# Guideline adherence and lost workdays for acute low back pain in the California workers’ compensation system

**DOI:** 10.1371/journal.pone.0253268

**Published:** 2021-06-17

**Authors:** Fraser W. Gaspar, Matthew S. Thiese, Kerri Wizner, Kurt Hegmann

**Affiliations:** 1 MDGuidelines, ReedGroup Ltd, Westminster, Colorado, United States of America; 2 Rocky Mountain Center for Occupational and Environmental Health, University of Utah, Salt Lake City, Utah, United States of America; University of Tennessee Health Science Center, UNITED STATES

## Abstract

**Context:**

The use of clinical-practice guidelines is a suggested method for improving health outcomes by the earlier provision of necessary and effective medical interventions.

**Objective:**

To quantify the influence of adherence to guideline-recommended interventions in the first week of treatment for an initial low back pain (LBP) injury on lost workdays.

**Methods:**

In a retrospective cohort of California’s workers’ compensation claims data from May 2009 to May 2018, 41 diagnostic and treatment interventions were abstracted from the medical claims for workers with acute LBP injuries and compared with guideline recommendations. Lost workdays within 1-year post-injury were compared by guideline adherence using quantile regressions.

**Results:**

Of the 59,656 workers who met the study inclusion criteria, 66.1% were male and the average (SD) age was 41 (12) years. The median number (IQR) of lost workdays was 27 (6–146) days. In the first week of treatment, 14.2% of workers received only recommended interventions, 14.6% received only non-recommended interventions, and 51.1% received both recommended and non-recommended interventions. Opioid prescriptions fell 86% from 2009 to 2018. Workers who received only guideline-recommended interventions experienced significantly fewer lost workdays (11.5 days; 95% CI: -13.9, -9.1), a 29.3% reduction, than workers who received only non-recommended interventions. The percentage of workers receiving only recommended interventions increased from 10.3% to 18.2% over the 9 years.

**Conclusion and relevance:**

When workers received guideline-recommended interventions, they typically returned to work in fewer days. The majority of workers received at least one non-recommended intervention, demonstrating the need for adherence to guideline recommendations. Fewer lost workdays and improved quality care are outcomes that strongly benefit injured workers.

## Introduction

Overtreatment and low-value care cost the U.S. healthcare system between $75.7 and $101.2 billion annually [1]. Despite the associated high cost, unnecessary or ineffective care appear to be on the rise [2]. One strategy to promote quality, value-based care is applying evidence-based medicine (EBM) to help guide treatment decisions. EBM integrates medical research with clinical expertise and patient values to support decision making based on the best available evidence [3].

In the U.S., state workers’ compensation (WC) systems have developed or adopted treatment guidelines to promote evidence-based care for occupational injuries. The most common occupational injury is back strain [4], and occupational stressors are thought to contribute to low back pain (LBP) [5, 6]. Considerable differences exist between evidence-based recommendations and current clinical practice for the treatment of LBP [7, 8]. An increasing number of studies in the literature suggest that following EBM guidelines improves outcomes and/or decreases costs [9–11]. For example, Owens et al. (2019) found that medical and total claim costs, which included payments for lost time (i.e., indemnity), decreased by $353 and $586, respectively, per unit of compliance with LBP guidelines in Workers’ Compensation Fund of Utah claims [11].

For occupational injuries and diseases, the majority of costs are related to a loss of functional capacity, including the inability to work [12]. Work absences not only reduce workers’ immediate earnings but may also increase the risk of future unemployment and loss of income [13]. In addition, both the employer and society experience negative effects from lost time, including reduced productivity and economic losses [14]. Therefore, the reduction of lost workdays and the promotion of effective, value-based care is an important area to investigate.

The aim of this study was to determine the influence of adherence to guideline recommendations on lost workdays for workers with acute LBP claims in California’s WC system.

## Materials and methods

### Study population

Workers’ Compensation claims reported to the California’s Department of Industrial Relations Workers’ Compensation Information System (WCIS) with a date of injury between May 2009 and May 2018 were used in this study. These dates were chosen to explore 9-year trends and capture at least one year of follow-up after the claim started. The data come from the 4010 and 5010 billing systems and include claims from employers with at least 150 employees, as smaller employers are not required to report to the WCIS. The data provided met the criteria for a limited-use data set under HIPAA and this study was not evaluated by an institutional review board because it did not meet the definition of human subjects research [15].

**Fig 1:**
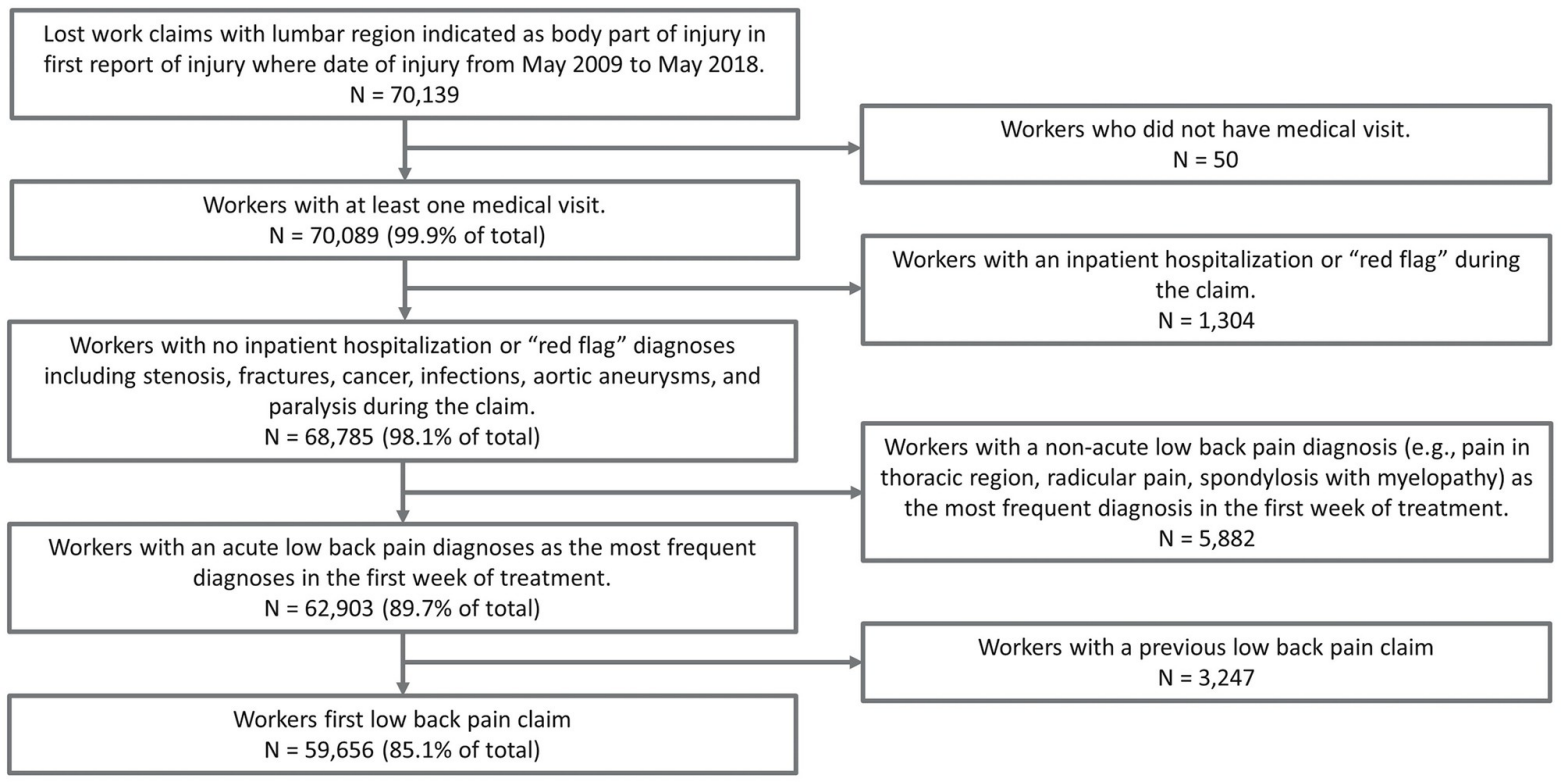
Flow chart depicting generation of the study population.

The study population consisted of workers with a lost time claim whose first report of injury indicated that the body part of injury was for the “low back area (lumbar and lumbosacral)” or the “lumbar and/or sacral vertebrae (vertebrae NOC trunk)” (Fig 1). Only workers with at least one medical visit were included. To focus on the treatment of uncomplicated LBP claims, claims were removed if they had an inpatient admission during the claim or a “red flag” diagnosis that may indicate treatment outside of guideline recommendations, such as fracture, cancer, infection, aortic aneurysm, and paralysis (S1 Table). Claims were excluded with stenosis diagnoses if they also had an accompanying surgical procedure (i.e., decompression, fusion, or adhesiolysis). Claims were also excluded if there had been a prior LBP claim to remove complicated clinical presentations due to LBP recurrences.

To confirm the LBP diagnosis, all primary medical diagnoses recorded within a week of the first medical visit were abstracted and at least 50% of the diagnoses had to be associated with acute LBP. Examples of back pain diagnoses not considered include pain in thoracic region, radicular pain, and spondylosis with myelopathy. Diagnoses included are listed in S2 Table.

### Guideline treatments

The American College of Occupational and Environmental Medicine (ACOEM)’s Low Back Disorders Practice Guideline was used as the source of guideline recommendations [16]. ACOEM’s treatment guidelines have been adopted in California’s Medical Treatment Utilization Schedule (MTUS), which determines what is reasonable and necessary medical care [17, 18]. Forty-one distinct treatments and diagnostic interventions within a week of the first medical visit were studied. The one-week treatment timeframe was *a priori* set to study, as the first appointment sets patient expectations and implementation of in/effective treatments begins, which are theorized to influence and set the trajectory of the LBP claim. Only treatments that could be reliably captured in a medical billing system were analyzed. Common Procedure Terminology^®^ and Healthcare Common Procedure Coding System codes were abstracted from the WCIS system to study diagnostic interventions and treatment procedures. National Drug Codes for the medications and topical creams were abstracted from the WCIS system and grouped using IBM Micromedex^®^ RED BOOK^®^ to evaluate the pharmaceutical interventions.

Workers were categorized into four distinct groups to study guideline adherence: 1) worker received only non-recommended interventions; 2) worker received only recommended interventions; 3) worker received recommended and non-recommended interventions; and 4) worker received either no intervention or other medical interventions. Other medical interventions could include treatment and diagnostic interventions that have no recommendation in the guideline. No recommendations are assigned when there is a lack of evidence and the balance of benefits, harms, and costs cannot be determined [19].

### Outcomes

Lost workdays were calculated as the number of workdays between the payment start and end dates for temporary total disability. For individuals receiving lump sum indemnity payments, the lump sum was divided by their daily wage. In California, injured workers receive two-thirds of their gross wages as payment, with minimum and maximum allowed weekly payments [20]. To calculate the temporary total disability daily payment per worker, the workers’ daily wages were calculated as their yearly income divided by the number of workdays in a typical year (261) and multiplied by two-thirds. If the weekly payments were below the minimum or above the maximum allowed weekly payments, they were adjusted up or down, respectively.

### Statistical analysis

Frequency and percent were calculated for all categorical variables used. Differences in the percent of workers receiving a type of medical intervention over time were tested using logistic regression models, with year of injury as the independent variable. Normality, homoscedasticity, and linearity of the continuous outcome variable, lost workdays, was assessed. Multiple variable quantile regression, an extension of linear regression, was used to test the influence of guideline adherence on lost workdays. In addition to the advantage of interpretability, quantile regressions were employed due to the right skewness of lost workdays. Covariates used in the multiple variable models included age (years), whether medical claims were present in 4010 billing system (yes/no), gender (male/female), whether the workers’ home was in a rural location (yes/no), yearly income (categorized), employment industry (categorized), employment status (regular employment vs. other), whether the worker had a previous WC claim (yes/no), time from injury to first medical visit (numeric days), medical complexity (numeric variables), year of injury, and the presence of at least one comorbidity (yes/no). Covariates were selected *a priori* and included if they were theoretical confounders or predictors of the outcome.

The industry of the patient’s employer was categorized using the North American Industry Classification System (NAICS) sectors, with any Standard Industrial Classification (SIC) codes mapped to 2017 NAICS codes using U.S. Census Bureau crosswalk tables [21]. When income was not available (11.4% of workers), it was imputed using the median income of the workers’ Standard Occupational Classification (SOC) or, if unavailable, the NAICS sector [22]. The workers’ SOC was determined by mapping the workers’ job description to the O*NET 24.2 database’s job descriptions [23]. Rurality of the worker was determined by mapping the worker’s zip code to rurality indicators by the Centers for Medicare & Medicaid Services [24]. Medical complexity was derived by counting the number of distinct medical visits and diagnoses in the first week of treatment, with higher counts indicating more complex cases. Comorbidities recorded in the first week were grouped using Quan et al.’s (2005) coding algorithm [25]. In addition, whether a worker smoked was determined using nicotine dependence diagnosis codes. Due to the limited capturing of comorbidities and smoking status, a single binary variable was developed to indicate if at least one comorbidity was present or if the worker smoked.

Missing variables (maximum percent missing = 16.5% for employment status) were imputed using the observed empirical distributions. As a sensitivity analysis, the quantile regression models were rerun to include 5,640 workers who had a primary LBP diagnosis that was considered unspecific and more general than the diagnoses used for the study population (S2 Table). Examples of unspecific LBP diagnosis include spondylosis of unspecified site, Schmorl’s nodes of unspecified region, and unspecified thoracic, thoracolumbar and lumbosacral intervertebral disc disorder. The sensitivity analysis was used to test the influence of our medical code inclusion criteria, as medical billers may use these unspecific codes when billing for LBP. A second sensitivity analysis was performed to test the influence of treatment bias using propensity score weights in the multiple variable quantile regression. Propensity score weights were calculated using multinomial predicted probabilities, where the dependent variable was treatment recommendation categories and the independent variables were a subset of the full model covariates hypothesized to be associated with treatment allocation. These independent variables included age, gender, rurality, number of comorbidities, and year of injury.

Due to the large sample size, statistical significance was *a priori* set at a p-value of < 0.01. Data management was performed in SQL Server 2012 and data cleaning and analyses were performed in R Version 3.6.1 [26].

## Results

A total of 59,656 workers met the study inclusion criteria and had an average (SD) age of 41 (12) years (Table 1). The workers tended to be male (66.1%), from an urban area (92.9%), and had regular employment (66.7%). The median yearly income was $32,404. The top three industry sectors were retail trade (15.2%), manufacturing (11.4%), and health care and social assistance (11.1%). The median number (interquartile range [IQR]) of lost workdays was 27 (6–146).

**Table 1:** Demographic characteristics of the study population (n = 59,656).

Categories	Number of workers (%)
**Gender**	
Female	19,991 (33.5%)
Male	39,433 (66.1%)
Unknown/missing	232 (0.4%)
**Age (years)**	
<25	5,348 (9.0%)
25 to 34	15,958 (26.8%)
35 to 44	15,191 (25.5%)
45 to 54	13,787 (23.1%)
55 to 64	8,095 (13.6%)
65 to 74	1,149 (1.9%)
Unknown/missing	128 (0.2%)
**Employment status**	
Regular employee	39,798 (66.7%)
Part-time employee	6,609 (11.1%)
Other (e.g., seasonal, apprenticeship)	3,475 (5.8%)
Unknown/missing	9,774 (16.4%)
**Annual income**	
<$25,000	19,170 (32.1%)
$25,000 to <$35,000	13,940 (23.4%)
$35,000 to <$45,000	8,738 (14.6%)
$45,000 to <$55,000	5,959 (10.0%)
$55,000 to <$65,000	3,412 (5.7%)
$65,000 to <$75,000	2,396 (4.0%)
≥$75,000	6,024 (10.1%)
Unknown/missing	17 (<0.1%)
**Rurality**	
Rural	3,786 (6.3%)
Urban	55,417 (92.9%)
Unknown/missing	453 (0.8%)

In the first week of treatment, 14.2% of workers received only recommended interventions, 14.6% received only non-recommended interventions, and 51.1% received both recommended and non-recommended interventions. Fewer than half of the workers received multiple (>1) recommended interventions (43.7%) or multiple non-recommended interventions (23.7%). The most common recommended interventions included prescriptions for nonsteroidal anti-inflammatory drugs (NSAIDs) (44.2%) and muscle relaxants (35.2%) (Table 2). The most common non-recommended interventions included x-rays (49.6%) and prescriptions for opioids (19.6%). Between the years 2009 and 2018, the percentage of workers receiving only recommended interventions increased from 10.3% to 18.2%, whereas the percentage receiving only non-recommended treatments increased at a slower rate from 12.1% to 15.4%.

**Table 2:** Frequency of interventions for acute low back pain in first week of treatment.

Intervention	Recommendation (strength of evidence)	% of study population ^a^
**Nonsteroidal anti-inflammatory drugs**	Strongly recommended (A)	44.2%
**Ketorolac injection**	Strongly recommended (A)	15.8%
**Muscle relaxant**	Moderately recommended (B)	35.2%
**Capsaicin**	Moderately recommended (B)	<0.1%
**Acetaminophen**	Recommended (C)	7.5%
**Tricyclic or serotonin norepinephrine reuptake inhibitor antidepressant**	Recommended (C)	0.1%
**Physical or occupational therapy** ^b^	Recommended (I)	16.6%
**Manipulation**	Recommended (I)	11.2%
**Manual therapy/massage**	Recommended (I)	5.5%
**Lidocaine and topical creams**	No recommendation (I)	10.3%
**Infrared therapy**	No recommendation (I)	3.4%
**Ultrasound (therapeutic)**	No recommendation (I)	2.0%
**Electrical stimulation**	Not recommended (I)	15.1%
**Carisoprodol**	Not recommended (I)	2.9%
**Magnetic resonance imaging or computed tomography**	Not recommended (I)	1.8%
**Traction**	Not recommended (I)	1.3%
**Hot or cold therapy at appointment** ^c^	Not recommended (I)	0.9%
**Acupuncture**	Not recommended (I)	0.2%
**Iontophoresis**	Not recommended (I)	0.2%
**Tender and trigger point injections**	Not recommended (I)	0.1%
**Electromyography**	Not recommended (I)	0.1%
**Functional capacity evaluation**	Not recommended (I)	<0.1%
**Aquatic therapy (including swimming)** ^d^	Not recommended (I)	<0.1%
**Selective serotonin reuptake inhibitor antidepressant**	Not recommended (I)	<0.1%
**Diagnostic facet joint injections (intraarticular and nerve blocks)**	Not recommended (I)	<0.1%
**Bone scans**	Not recommended (I)	<0.1%
**Intradiscal steroids**	Not recommended (I)	<0.1%
**Fluoroscopy**	Not recommended (I)	0%
**Thermography**	Not recommended (I)	0%
**Videofluoroscopy**	Not recommended (I)	0%
**Ultrasound (diagnostic)**	Not recommended (I)	0%
**Adhesiolysis**	Not recommended (I)	0%
**Implantable spinal cord stimulators**	Not recommended (I)	0%
**Diathermy**	Not recommended (C)	0.7%
**Anticonvulsants**	Not recommended (C)	0.2%
**Epidural injections**	Not recommended (C)	<0.1%
**X-ray**	Moderately not recommended (B)	49.6%
**Glucocorticoids**	Moderately not recommended (B)	3.2%
**Opioids**	Strongly not recommended (A)	19.6%
**Prolotherapy injections**	Strongly not recommended (A)	0.1%
**Discography**	Strongly not recommended (A)	<0.1%

^a^ Data are not mutually exclusive.

^b^ Specific types of exercise carry various A, B or C recommendation levels. For example, in the acute LBP setting, the favorable recommendations include progressive aerobic exercise (B) and directional stretching (C). This level of specificity was not available in the dataset.

^c^ Heat and cryotherapies are recommended for home use.

^d^ Aquatic therapy is recommended for select use in the subacute and chronic LBP patients for those with significant weight-bearing problems or other reasons to not engage in land-based exercise program.

**Fig 2:**
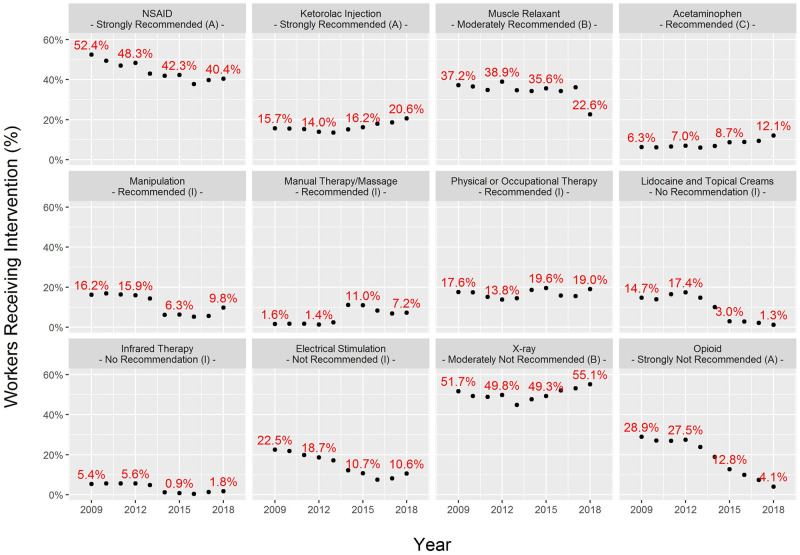
Percent of workers receiving intervention by year of injury.

For the most frequent interventions, there were statistically significant trends over the study years, some increasing and some decreasing (p-value < 0.01, Fig 2). Interventions that increased through the study period included ketorolac injections (strongly recommended), acetaminophen (recommended), manual therapy/massage (recommended), physical or occupational therapy (recommended), and x-rays (moderately not recommended). Interventions that decreased through the study years included NSAIDs (strongly recommended), muscle relaxants (moderately recommended), manipulation (recommended), lidocaine and topical creams (no recommendation), infrared therapy (no recommendation), electrical stimulation (not recommended), and opioids (strongly not recommended). Opioids had the largest decrease, with 28.9% of workers receiving a prescription in 2009 but only 4.1% receiving a prescription in 2018, an 86% reduction.

Workers receiving an x-ray had slightly longer lost workdays (median = 28 days) than workers not receiving an x-ray (median = 27 days) (Kolmogorov-Smirnov test p-value <0.001). Opioid prescriptions had more of an influence on lost workdays, as workers prescribed an opioid were typically out of work four days longer than workers not prescribed opioids (medians = 30 vs. 26 days) (Kolmogorov-Smirnov test p-value <0.001).

In multiple variable quantile regression models, workers who received only recommended interventions experienced 11.5 fewer lost workdays (95% CI: -13.9, -9.1), a 29.3% reduction, than workers who received only non-recommended interventions (Table 3 and S3 Table). Workers who received both recommended and non-recommended interventions experienced 7.9 fewer lost workdays (95% CI: -10.3, -5.5) than workers who received only non-recommended interventions. In addition, workers who received either no intervention or other medical interventions experienced more favorable outcomes than those who received non-recommended interventions (Beta = -7.7, 95% CI: -10.3, -5.1). The sensitivity analysis that included workers with primary LBP diagnoses that were less specific to acute LBP did not meaningfully change the results (S4 Table). In addition, propensity score weights in the quantile regressions did not meaningfully change the results (S5 Table).

**Table 3:** Univariate and multiple variable quantile regression results testing the influence of guideline adherence on lost workdays.

Reference group	Exposure group	Unadjusted models		Multiple variable models ^a^	
		Beta (95% CI)	p-value	Beta (95% CI)	p-value
Received only non-recommended interventions	Received only recommended interventions (No = 0, Yes = 1)	-17.0 (-19.8, -14.2)	<0.0001	-11.5 (-13.9, -9.1)	<0.0001
	Received recommended and non-recommended interventions (No = 0, Yes = 1)	-10.0 (-12.7, -7.3)	<0.0001	-7.9 (-10.3, -5.5)	<0.0001
	Received either no intervention or other medical interventions (No = 0, Yes = 1)	-8.0 (-11.0, -5.0)	<0.0001	-7.7 (-10.3, -5.1)	<0.0001

^a^ Multiple variable model control for age, gender, medical complexity, work industry, billing system, presence of a comorbidity, income, rurality, history of previous workers’ compensation claim, time to initial visit, year of injury, and employment status. Full results presented in S3 Table.

## Discussion

Workers receiving only recommended interventions incurred 11.5 fewer lost workdays, a 29.3% reduction, compared with those who received only non-recommended interventions in this large, retrospective cohort study. The majority of workers received at least one medical intervention that was not recommended by the ACOEM Low Back Disorders Practice Guideline. The percentage of workers receiving only recommended interventions increased from 10.3% to 18.2%. Opioid prescriptions fell 86% from 2009 to 2018. X-rays were a common non-recommended diagnostic intervention that increased in frequency throughout the study period.

According to the Institute of Medicine, the delay for incorporation of EBM into clinical practice approaches 17 years [27]. Identifying, digesting, and integrating research findings into clinical practice can be overwhelming for most clinicians, given that approximately 2 million papers are published annually and the number of published papers grows exponentially at 8–9% per year [28]. Even when limiting a research literature review to randomized control trials, a clinician needs to read approximately 48 papers a day to stay current with the literature [29]. The trends in changes in rates of prescriptions for specific treatments over nine years appear to support a long time horizon for the incorporation of practice changes. Reliance on high quality EBM treatment guidelines is important and will likely become more necessary in the future.

To address the disconnect between evidence and practice, it has been proposed that applying clinical decision-support systems and tools to bring guidelines to the point-of-care improves healthcare processes, such as facilitating preventive services, ordering clinical studies, and prescribing therapies [30]. In this spirit, as part of the Protecting Access to Medicare Act, the Centers for Medicare & Medicaid Services will require practitioners, or staff acting on their behalf, to consult a clinical decision support mechanism when ordering advanced diagnostic imaging services for Medicare beneficiaries starting on January 1, 2021. In 2018, the California Department of Industrial Relations began providing web-based access to ACOEM’s guidelines for all California workers’ compensation system providers. The expectation is that giving clinicians increased access to guidelines will better align treatment decisions between providers, workers, employers, and payers.

Changes in California’s workers’ compensation MTUS, including the adoption of an evidence-based drug formulary based on ACOEM’s guidelines (effective date January 1, 2018), were not considered in the analysis. Through the study period, four versions of ACOEM’s guidelines were published; however, the recommendations, especially for the most common treatments and diagnostic interventions, have not significantly through the study period (S6 Table). For purposes of workers’ compensation reimbursement, a significant change is to move from recommended to not recommended, or vice versa, as other changes (e.g., from Recommended C to Recommended B) do not change approvals and payments for services or treatments. For example, x-rays and opioids for acute low back pain, the two most common not recommended diagnostic intervention and treatment found in this study, have been not recommended in every version since 2008.

In the state of California, ACOEM’s guidelines are the presumptively correct guidelines that physicians must adhere to in treatment and diagnostic intervention decisions. Therefore, we did not consider the recommendations from other guidelines including the American College of Physicians (ACP) or the United Kingdoms’ National Institute of Health and Care Excellence (NICE) guidelines [31, 32]. In general, ACOEM guidelines tended to match NICE’s recommendations better than ACP’s guideline recommendations (summarized in S7 Table). For example, ACOEM and NICE do not recommend acupuncture for treatment of acute low back pain, whereas ACP does recommend acupuncture. The differences in recommendations between guideline may be important to consider when extrapolating the results of this study to other populations that may follow different treatment guidelines.

The strengths of this research include the use of a large, population-based integrated database to match medical and lost workdays information. The use of medical billing information allowed the investigation of 41 medical interventions; however, this study could not investigate interventions that were not tracked by the claims system, including over-the-counter NSAID use, bed rest, and modified duty. The coding also does not allow for detailed assessment of specific types of exercise, rather it is captured at the level of physical or occupational therapy. Confounders were identified and statistically controlled using multivariable regression. These included two medical complexity variables used to control for disease severity; however, additional metrics including standardized pain scales were not available. Additionally, temporality of the treatments is demonstrated by these data; therefore, the potentially causal link between recommended treatments and fewer lost workdays is plausible. Reverse causation in this instance is unlikely. Some variables known to influence lost workdays including race or psychosocial factors were not available and could not be controlled for in our analysis. Finally, the workers’ job requirements were not known, which may have influenced their ability to return to work and the total number of lost workdays, although this seems likely to have been non-differentially distributed and thus a possible bias towards the null.

## Conclusions

When workers received guideline-recommended interventions, they typically returned to work in fewer days. The majority of workers received at least one non-recommended intervention, demonstrating the need for adherence to guideline recommendations. Fewer lost workdays and improved quality care are outcomes that strongly benefit injured workers.

## Supporting information

S1 Table Red flag diagnoses used to exclude claims. Red flag diagnoses may support medical interventions outside of guideline recommendations and thus were *a priori* excluded.
(DOCX)

S2 Table Medical codes used to define acute low back pain (LBP) cases. Primary LBP codes are medical codes used to define the study population, whereas the unspecific LBP codes were used in the sensitivity analysis to explore the influence of the LBP definition.
(DOCX)

S3 Table Full results of the quantile regression model testing the influence of receiving only recommended, only non-recommended, and both recommended and non-recommended treatments on lost workdays. (DOCX)

S4 Table Sensitivity analysis including workers whose primary diagnosis was unspecific to the acute LPP diagnoses used in the main study population (n = 65,296) testing the influence of receiving only recommended, only non-recommended, and both recommended and non-recommended treatments on lost workdays. (DOCX)

S5 Table Sensitivity analysis including propensity score weights within the quantile regression testing the influence of receiving only recommended, only non-recommended, and both recommended and non-recommended treatments on lost workdays. (DOCX)

S6 Table ACOEM Guidelines’ acute low back pain recommendation changes over time for common treatments in study. (DOCX)

S7 Table Comparisons between select recommendation statements of the ACOEM, American College of Physicians (ACP) and United Kingdoms’ National Institute of Health and Care Excellence (NICE) guidelines for acute low back pain. (DOCX)
