# Supplementary material for: Guideline adherence and lost workdays for acute low back pain in the California workers’ compensation system
Source: PLoS One. 2021 Jun 17;16(6):e0253268. doi: 10.1371/journal.pone.0253268 (PMC8211224; doi:10.1371/journal.pone.0253268)
Supplement: S1 Table — Red flag diagnoses may support medical interventions outside of guideline recommendations and thus were a priori excluded. (DOCX) [file pone.0253268.s001.docx]

**Table S1. Red flag diagnoses used to exclude claims. Red flag diagnoses may support medical interventions outside of guideline recommendations and thus were *a priori* excluded.**

| **Medical Code** | **Medical Code Description** |
| --- | --- |
| 001 | Cholera |
| 001.0 | Cholera, Due to Vibrio cholerae |
| 001.1 | Cholera, Due to Vibrio cholerae el tor |
| 001.9 | Cholera, Unspecified |
| 002 | Typhoid and Paratyphoid Fevers |
| 002.0 | Typhoid Fever, Infection, Any Site |
| 002.1 | Paratyphoid Fever A |
| 002.2 | Paratyphoid Fever B |
| 002.3 | Paratyphoid Fever C |
| 002.9 | Paratyphoid Fever, Unspecified |
| 003 | Salmonella Infections, Other; Includes Infection or Food Poisoning by Salmonella |
| 003.0 | Salmonella Gastroenteritis, Salmonellosis |
| 003.1 | Salmonella Septicemia |
| 003.2 | Salmonella Infections, Localized |
| 003.20 | Salmonella Infection, Localized, Unspecified |
| 003.21 | Salmonella Infection, Localized, Salmonella Meningitis |
| 003.22 | Salmonella Infection, Localized, Salmonella Pneumonia |
| 003.23 | Salmonella Infection, Localized, Salmonella Arthritis |
| 003.24 | Salmonella Infection, Localized, Salmonella Osteomyelitis |
| 003.29 | Salmonella Infections, Localized, Other |
| 003.8 | Salmonella Infections, Other Specified |
| 003.9 | Salmonella Infection, Unspecified |
| 004 | Shigellosis; Includes Bacillary Dysentery |
| 004.0 | Shigella Dysenteriae, Infection by Group A Shigella (Schmitz) (Shiga) |
| 004.1 | Shigella Flexneri, Infection by Group B Shigella |
| 004.2 | Shigella Boydii, Infection by Group C Shigella |
| 004.3 | Shigella Sonnei, Infection by Group D Shigella |
| 004.8 | Shigella Infections, Other Specified |
| 004.9 | Shigellosis, Unspecified |
| 005 | Food Poisoning, Other, Bacterial |
| 005.0 | Staphylococcal Food Poisoning, Staphylococcal Toxemia Specified as Due to Food |
| 005.1 | Botulism; Botulism NOS; Food Poisoning Due to Clostridium Botulinum |
| 005.2 | Food Poisoning Due to Clostridium Perfringens; Enteritis Necroticans |
| 005.3 | Food Poisoning Due to Other Clostridia |
| 005.4 | Food Poisoning Due to Vibrio parahaemolyticus |
| 005.8 | Food Poisoning, Bacterial, Other |
| 005.81 | Food poisoning Due to Vibrio vulnificus |
| 005.89 | Food Poisoning, Other Bacterial; Food poisoning Due to Bacillus cereus |
| 005.9 | Food Poisoning, Unspecified |
| 006 | Amebiasis; Includes Infection Due to Entamoeba Histolytica |
| 006.0 | Amebic Dysentery, Acute, without Mention of Abscess; Acute Amebiasis |
| 006.1 | Amebiasis, Chronic Intestinal, without Mention of Abscess; Amebiasis, Chronic; Amebic Dysentery, Chronic |
| 006.2 | Amebic Nondysenteric Colitis |
| 006.3 | Amebic Liver Abscess; Hepatic Amebiasis |
| 006.4 | Amebic Lung Abscess; Amebic Abscess of Lung, Liver |
| 006.5 | Amebic Brain Abscess; Amebic Abscess of Brain, Liver, Lung |
| 006.6 | Amebic Skin Ulceration; Cutaneous Amebiasis |
| 006.8 | Amebic Infection of Other Sites; Amebic Appendicitis; Amebic Balanitis; Ameboma |
| 006.9 | Amebiasis, Unspecified; Amebiasis NOS |
| 007 | Protozoal Intestinal Diseases, Other; Includes Protozoal Colitis, Protozoal Diarrhea, Protozoal Dysentery |
| 007.0 | Protozoal Intestinal Diseases, Other; Balantidiasis; Infection by Balantidium coli |
| 007.1 | Protozoal Intestinal Diseases, Other; Giardiasis; Infection by Giardia Lamblia; Lambliasis |
| 007.2 | Protozoal Intestinal Diseases, Other; Coccidiosis; Infection by Isospora belli and Isospora hominis; Isosporiasis |
| 007.3 | Protozoal Intestinal Diseases, Other; Trichomoniasis, Intestinal |
| 007.4 | Protozoal Intestinal Diseases, Other; Cryptosporidiosis |
| 007.5 | Protozoal Intestinal Diseases, Other; Cyclosporiasis |
| 007.8 | Protozoal Intestinal Diseases, Other Specified; Amebiasis Due to Organisms Other than Entamoeba Histolytica |
| 007.9 | Protozoal Intestinal Disease, Unspecified; Flagellate Diarrhea; Protozoal Dysentery NOS |
| 008 | Intestinal Infections Due to Other Organisms |
| 008.0 | Intestinal Infections Due to Other Organisms; Escherichia coli (E. coli) |
| 008.00 | Intestinal Infections Due to Other Organisms; E. coli, Unspecified; E. coli enteritis NOS |
| 008.01 | Intestinal Infections Due to Other Organisms; Enteropathogenic E. coli |
| 008.02 | Intestinal Infections Due to Other Organisms; Enterotoxigenic E. coli |
| 008.03 | Intestinal Infections Due to Other Organisms; Enteroinvasive E. coli |
| 008.04 | Intestinal Infections Due to Other Organisms; Enterohemorrhagic E. coli |
| 008.09 | Intestinal Infections Due to Other Organisms; E. coli Infections, Other Intestinal |
| 008.1 | Intestinal Infections Due to Other Organisms; Arizona Group of Paracolon Bacilli |
| 008.2 | Intestinal Infections Due to Other Organisms; Aerobacter Aerogenes; Enterobacter Aerogenes |
| 008.3 | Intestinal Infections Due to Other Organisms; Proteus (Mirabilis) (Morganii) |
| 008.4 | Intestinal Infections Due to Other Specified Bacteria |
| 008.41 | Intestinal Infections Due to Other Specified Bacteria; Staphylococcus; Staphylococcal enterocolitis |
| 008.42 | Intestinal Infections Due to Other Specified Bacteria; Pseudomonas |
| 008.43 | Intestinal Infections Due to Other Specified Bacteria; Campylobacter |
| 008.44 | Intestinal Infections Due to Other Specified Bacteria; Yersinia enterocolitica |
| 008.45 | Intestinal Infections Due to Other Specified Bacteria; Clostridium difficile; Pseudomembranous colitis |
| 008.46 | Intestinal Infections Due to Other Specified Bacteria; Other Anaerobes; Anaerobic enteritis NOS; Bacteroides, Fragilis; Gram-negative Anaerobes |
| 008.47 | Intestinal Infections Due to Other Specified Bacteria; Gram-negative Bacteria, Other; Gram-negative Enteritis NOS |
| 008.49 | Intestinal Infections Due to Other Specified Bacteria, Other |
| 008.5 | Intestinal Infections Due to Other Organisms; Bacterial Enteritis, Unspecified |
| 008.6 | Intestinal Infections Due to Other Organisms; Enteritis Due to Specified Virus |
| 008.61 | Intestinal Infections Due to Other Organisms; Enteritis Due to Specified Virus; Rotavirus |
| 008.62 | Intestinal Infections Due to Other Organisms; Enteritis Due to Specified Virus; Adenovirus |
| 008.63 | Intestinal Infections Due to Other Organisms; Enteritis Due to Specified Virus; Norwalk Virus; Norwalk-like Agent; Norovirus |
| 008.64 | Intestinal Infections Due to Other Organisms; Enteritis Due to Specified Virus; Other Small Round Viruses; Small Round Virus NOS |
| 008.65 | Intestinal Infections Due to Other Organisms; Enteritis Due to Specified Virus; Calcivirus |
| 008.66 | Intestinal Infections Due to Other Organisms; Enteritis Due to Specified Virus; Astrovirus |
| 008.67 | Intestinal Infections Due to Other Organisms; Enteritis Due to Specified Virus; Enterovirus NEC; Coxsackie Virus; Echovirus |
| 008.69 | Intestinal Infections Due to Other Organisms; Enteritis Due to Specified Virus; Viral Enteritis, Other; Torovirus |
| 008.8 | Intestinal Infection due to Other Organism, NEC; Viral, Enteritis NOS, Gastroenteritis |
| 009 | Ill-defined Intestinal Infections |
| 009.0 | Ill-defined Intestinal Infections; Infectious Colitis, Enteritis, and Gastroenteritis; Colitis, Septic; Dysentery, NOS, Catarrhal, Hemorrhagic; Enteritis, Septic; Gastroenteritis, Septic |
| 009.1 | Ill-defined Intestinal Infections; Colitis, Enteritis, and Gastroenteritis of Presumed Infectious Origin |
| 009.2 | Ill-defined Intestinal Infections; Infectious Diarrhea, Dysenteric, Epidemic; Infectious Diarrheal Disease NOS |
| 009.3 | Ill-defined Intestinal Infections; Diarrhea of Presumed Infectious Origin |
| 010 | Primary Tuberculous Infection |
| 010.0 | Primary Tuberculous Infection |
| 010.00 | Primary Tuberculous Infection, Unspecified |
| 010.01 | Primary Tuberculous Infection, Bacteriological or Histological Examination Not Done |
| 010.02 | Primary Tuberculous Infection, Bacteriological or Histological Examination Unknown, at Present |
| 010.03 | Primary Tuberculous Infection, Tubercle Bacilli Found (in Sputum) by Microscopy |
| 010.04 | Primary Tuberculous Infection, Tubercle Bacilli Found by Bacterial Culture |
| 010.05 | Primary Tuberculous Infection, Tuberculosis Confirmed Histologically |
| 010.06 | Primary Tuberculous Infection, Tuberculous Confirmed by Other Methods, Inoculation of Animals |
| 010.1 | Tuberculous Pleurisy in Primary Progressive Tuberculosis |
| 010.10 | Tuberculous Pleurisy in Primary Progressive Tuberculosis, Unspecified |
| 010.11 | Tuberculous Pleurisy in Primary Progressive Tuberculosis, Bacterial or Histological Examination Not Done |
| 010.12 | Tuberculous Pleurisy in Primary Progressive Tuberculosis, Bacterial or Histological Examination Unknown |
| 010.13 | Tuberculous Pleurisy in Primary Progressive Tuberculosis, Tubercle Bacilli Found (in Sputum) by Microscopy |
| 010.14 | Tuberculous Pleurisy in Primary Progressive Tuberculosis, Tubercle Bacilli Found by Bacterial Culture |
| 010.15 | Tuberculous Pleurisy in Primary Progressive Tuberculosis, Tuberculosis Confirmed Histologically |
| 010.16 | Tuberculous Pleurisy in Primary Progressive Tuberculosis, Tubercle Bacilli Confirmed by Other Methods, Inoculation of Animals |
| 010.8 | Primary Progressive Tuberculosis, Other |
| 010.80 | Primary Progressive Tuberculosis, Other; Unspecified |
| 010.81 | Primary Progressive Tuberculosis, Other; Bacterial or Histological Examination Not Done |
| 010.82 | Primary Progressive Tuberculosis, Other; Bacterial or Histological Examination Unknown |
| 010.83 | Primary Progressive Tuberculosis, Other; Tubercle Bacilli Found (in Sputum) by Microscopy |
| 010.84 | Primary Progressive Tuberculosis, Other; Tubercle Bacilli Found by Bacterial Culture |
| 010.85 | Primary Progressive Tuberculosis, Other; Tuberculosis Confirmed Histologically |
| 010.86 | Primary Progressive Tuberculosis, Other; Tubercle Bacilli; Confirmed by Other Methods, Inoculation of Animals |
| 010.9 | Primary Tuberculous Infection, Unspecified |
| 010.90 | Primary Tuberculous Infection, Unspecified Type, Confirmation Unspecified |
| 010.91 | Primary Tuberculous Infection, Unspecified; Bacterial or Histological Examination Not Done |
| 010.92 | Primary Tuberculous Infection, Unspecified; Bacterial or Histological Examination Unknown |
| 010.93 | Primary Tuberculous Infection, Unspecified; Tubercle Bacilli Found (in Sputum) by Microscopy |
| 010.94 | Primary Tuberculous Infection, Unspecified; Tubercle Bacilli Found by Bacterial Culture |
| 010.95 | Primary Tuberculous Infection, Unspecified; Tuberculosis Confirmed Histologically |
| 010.96 | Primary Tuberculous Infection, Unspecified; Tubercle Bacilli Confirmed by Other Methods, Inoculation of Animals |
| 011 | Pulmonary Tuberculosis |
| 011.0 | Tuberculosis of Lung, Infiltrative |
| 011.00 | Tuberculosis of Lung, Infiltrative Unspecified |
| 011.01 | Tuberculosis of Lung, Infiltrative Bacterial or Histological Examination Not Done |
| 011.02 | Tuberculosis of Lung, Nodular, Bacterial or Histological Examination Unknown |
| 011.03 | Tuberculosis of Lung, Infiltrative Tubercle Bacilli Found (in Sputum) by Microscopy |
| 011.04 | Tuberculosis of Lung, Infiltrative Tubercle Bacilli Found by Bacterial Culture |
| 011.05 | Tuberculosis of Lung, Infiltrative Tuberculosis Confirmed Histologically |
| 011.06 | Tuberculosis of Lung, Infiltrative Tubercle Bacilli Confirmed by Other Methods, Inoculation of Animals |
| 011.1 | Tuberculosis of Lung, Nodular |
| 011.10 | Tuberculosis of Lung, Nodular, Unspecified |
| 011.11 | Tuberculosis of Lung, Nodular, Bacterial or Histological Examination Not Done |
| 011.12 | Tuberculosis of lung, nodular, bacteriological or histological examination unknown (at present) |
| 011.13 | Tuberculosis of Lung, Nodular, Tubercle Bacilli Found (in Sputum) by Microscopy |
| 011.14 | Tuberculosis of Lung, Nodular, Tubercle Bacilli Found by Bacterial Culture |
| 011.15 | Tuberculosis of Lung, Nodular, Tuberculosis Confirmed Histologically |
| 011.16 | Tuberculosis of Lung, Nodular, Tubercle Bacilli Confirmed by Other Methods, Inoculation of Animals |
| 011.2 | Tuberculosis of Lung with Cavitation |
| 011.20 | Tuberculosis of Lung with Cavitation, Unspecified |
| 011.21 | Tuberculosis of Lung with Cavitation, Bacterial or Histological Examination Not Done |
| 011.22 | Tuberculosis of Lung with Cavitation, Bacterial or Histological Examination Unknown |
| 011.23 | Tuberculosis of Lung with Cavitation, Tubercle Bacilli Found (in Sputum) by Microscopy |
| 011.24 | Tuberculosis of Lung with Cavitation, Tubercle Bacilli Found by Bacterial Culture |
| 011.25 | Tuberculosis of Lung with Cavitation, Tuberculosis Confirmed Histologically |
| 011.26 | Tuberculosis of Lung with Cavitation, Tubercle Bacilli Tuberculosis Confirmed by Other Methods [Inoculation of Animals] |
| 011.3 | Tuberculosis of Bronchus |
| 011.30 | Tuberculosis of Bronchus, Unspecified |
| 011.31 | Tuberculosis of Bronchus, Bacterial or Histological Examination Not Done |
| 011.32 | Tuberculosis of Bronchus, Bacterial or Histological Examination Unknown |
| 011.33 | Tuberculosis of Bronchus, Tubercle Bacilli Found (in Sputum) by Microscopy |
| 011.34 | Tuberculosis of Bronchus, Tubercle Bacilli Found by Bacterial Culture |
| 011.35 | Tuberculosis of Bronchus, Tuberculosis Confirmed Histologically |
| 011.36 | Tuberculosis of Bronchus, Tubercle Bacilli Tuberculosis Confirmed by Other Methods [Inoculation of Animals] |
| 011.4 | Tuberculous Fibrosis of Lung |
| 011.40 | Tuberculous Fibrosis of Lung, Unspecified |
| 011.41 | Tuberculous Fibrosis of Lung, Bacterial or Histological Examination Not Done |
| 011.42 | Tuberculous Fibrosis of Lung, Bacterial or Histological Examination Unknown |
| 011.43 | Tuberculous Fibrosis of Lung, Tubercle Bacilli Found (in Sputum) by Microscopy |
| 011.44 | Tuberculous Fibrosis of Lung, Tubercle Bacilli Found by Bacterial Culture |
| 011.45 | Tuberculous Fibrosis of Lung, Tuberculosis Confirmed Histologically |
| 011.46 | Tuberculous Fibrosis of Lung, Tubercle Bacilli Tuberculosis Confirmed by Other Methods [Inoculation of Animals] |
| 011.5 | Tuberculous Bronchiectasis |
| 011.50 | Tuberculous Bronchiectasis, Unspecified |
| 011.51 | Tuberculous Bronchiectasis, Bacterial or Histological Examination Not Done |
| 011.52 | Tuberculous Bronchiectasis, Bacterial or Histological Examination Unknown |
| 011.53 | Tuberculous Bronchiectasis, Tubercle Bacilli Found (in Sputum) by Microscopy |
| 011.54 | Tuberculous Bronchiectasis, Tubercle Bacilli Found by Bacterial Culture |
| 011.55 | Tuberculous Bronchiectasis, Tuberculosis Confirmed Histologically |
| 011.56 | Tuberculous Bronchiectasis, Tubercle Bacilli Tuberculosis Confirmed by Other Methods [Inoculation of Animals] |
| 011.6 | Tuberculous Pneumonia, Any Form |
| 011.60 | Tuberculous Pneumonia, Any Form; Unspecified |
| 011.61 | Tuberculous Pneumonia, Any Form; Bacterial or Histological Examination Not Done |
| 011.62 | Tuberculous Pneumonia, Any Form; Bacterial or Histological Examination Unknown |
| 011.63 | Tuberculous Pneumonia, Any Form; Tubercle Bacilli Found (in Sputum) by Microscopy |
| 011.64 | Tuberculous Pneumonia, Any Form; Tubercle Bacilli Found by Bacterial Culture |
| 011.65 | Tuberculous Pneumonia, Any Form; Tuberculosis Confirmed Histologically |
| 011.66 | Tuberculous Pneumonia, Any Form; Tubercle Bacilli Tuberculosis Confirmed by Other Methods [Inoculation of Animals] |
| 011.7 | Tuberculous Pneumothorax |
| 011.70 | Tuberculous Pneumothorax, Unspecified |
| 011.71 | Tuberculous Pneumothorax, Bacterial or Histological Examination Not Done |
| 011.72 | Tuberculous Pneumothorax, Bacterial or Histological Examination Unknown |
| 011.73 | Tuberculous Pneumothorax, Tubercle Bacilli Found (in Sputum) by Microscopy |
| 011.74 | Tuberculous Pneumothorax, Tubercle Bacilli Found by Bacterial Culture |
| 011.75 | Tuberculous Pneumothorax, Tuberculosis Confirmed Histologically |
| 011.76 | Tuberculous Pneumothorax, Tubercle Bacilli Tuberculosis Confirmed by Other Methods [Inoculation of Animals] |
| 011.8 | Pulmonary Tuberculosis, Other Specified Type |
| 011.80 | Pulmonary Tuberculous, Other Specified; Confirmation Unspecified |
| 011.81 | Pulmonary Tuberculous, Other Specified; Bacterial or Histological Examination Not Done |
| 011.82 | Pulmonary Tuberculous, Other Specified; Bacterial or Histological Examination Unknown |
| 011.83 | Pulmonary Tuberculous, Other Specified; Tubercle Bacilli Found (in Sputum) by Microscopy |
| 011.84 | Pulmonary Tuberculous, Other Specified; Tubercle Bacilli Found by Bacterial Culture |
| 011.85 | Pulmonary Tuberculous, Other Specified; Tuberculosis Confirmed Histologically |
| 011.86 | Pulmonary Tuberculous, Other Specified; Tubercle Bacilli Tuberculosis Confirmed by Other Methods [Inoculation of Animals] |
| 011.9 | Pulmonary Tuberculous, Unspecified; Respiratory Tuberculosis, NOS; Tuberculosis of Lung, NOS |
| 011.90 | Tuberculosis, Pulmonary, Unspecified; Respiratory Tuberculosis, NOS; Tuberculosis of Lung, NOS; Unspecified |
| 011.91 | Tuberculosis, Pulmonary, Unspecified; Respiratory Tuberculosis, NOS; Tuberculosis of Lung, NOS; Bacterial or Histological Examination Not Done |
| 011.92 | Tuberculosis, Pulmonary, Unspecified; Respiratory Tuberculosis, NOS; Tuberculosis of Lung, NOS; Bacterial or Histological Examination Unknown |
| 011.93 | Tuberculosis, Pulmonary, Unspecified; Respiratory Tuberculosis, NOS; Tuberculosis of Lung, NOS; Tubercle Bacilli Found (in Sputum) by Microscopy |
| 011.94 | Tuberculosis, Pulmonary, Unspecified; Respiratory Tuberculosis, NOS; Tuberculosis of Lung, NOS; Tubercle Bacilli Found by Bacterial Culture |
| 011.95 | Tuberculosis, Pulmonary, Unspecified; Respiratory Tuberculosis, NOS; Tuberculosis of Lung, NOS; Tuberculosis Confirmed Histologically |
| 011.96 | Tuberculosis, Pulmonary, Unspecified; Respiratory Tuberculosis, NOS; Tuberculosis of Lung, NOS; Tuberculosis Confirmed by Other Methods [Inoculation of Animals] |
| 012 | Tuberculosis, Respiratory, Other |
| 012.0 | Tuberculous Pleurisy; Tuberculosis of Pleura; Tuberculous Empyema; Tuberculous Hydrothorax |
| 012.00 | Tuberculous Pleurisy; Tuberculosis of Pleura; Tuberculous Empyema; Tuberculous Hydrothorax; Unspecified |
| 012.01 | Tuberculous Pleurisy; Tuberculosis of Pleura; Tuberculous Empyema; Tuberculous Hydrothorax; Bacterial or Histological Examination Not Done |
| 012.02 | Tuberculous Pleurisy, Bacterial or Histological Examination Unknown |
| 012.03 | Tuberculous Pleurisy; Tuberculosis of Pleura; Tuberculous Empyema; Tuberculous Hydrothorax; Tubercle Bacilli Found (in Sputum) by Microscopy |
| 012.04 | Tuberculous Pleurisy; Tuberculosis of Pleura; Tuberculous Empyema; Tuberculous Hydrothorax; Tubercle Bacilli Found by Bacterial Culture |
| 012.05 | Tuberculous Pleurisy; Tuberculosis of Pleura; Tuberculous Empyema; Tuberculous Hydrothorax; Tuberculosis Confirmed Histologically |
| 012.06 | Tuberculous Pleurisy; Tuberculosis of Pleura; Tuberculous Empyema; Tuberculous Hydrothorax; Tuberculosis Confirmed by Other Methods [Inoculation of Animals] |
| 012.1 | Tuberculosis of Intrathoracic Lymph Nodes; Tuberculosis of Lymph Nodes, Hilar, Mediastinal, Tracheobronchial; Tuberculous Tracheobronchial Adenopathy |
| 012.10 | Tuberculosis of Intrathoracic Lymph Nodes; Tuberculosis of Lymph Nodes, Hilar, Mediastinal, Tracheobronchial; Tuberculous Tracheobronchial Adenopathy; Unspecified |
| 012.11 | Tuberculosis of Intrathoracic Lymph Nodes; Tuberculosis of Lymph Nodes, Hilar, Mediastinal, Tracheobronchial; Tuberculous Tracheobronchial Adenopathy; Bacterial or Histological Examination Not Done |
| 012.12 | Tuberculosis of Intrathoracic Lymph Nodes; Tuberculosis of Lymph Nodes, Hilar, Mediastinal, Tracheobronchial; Tuberculous Tracheobronchial Adenopathy; Bacterial or Histological Examination Unknown |
| 012.13 | Tuberculosis of Intrathoracic Lymph Nodes; Tuberculosis of Lymph Nodes, Hilar, Mediastinal, Tracheobronchial; Tuberculous Tracheobronchial Adenopathy; Tubercle Bacilli Found (in Sputum) by Microscopy |
| 012.14 | Tuberculosis of Intrathoracic Lymph Nodes; Tuberculosis of Lymph Nodes, Hilar, Mediastinal, Tracheobronchial; Tuberculous Tracheobronchial Adenopathy; Tubercle Bacilli Found by Bacterial Culture |
| 012.15 | Tuberculosis of Intrathoracic Lymph Nodes; Tuberculosis of Lymph Nodes, Hilar, Mediastinal, Tracheobronchial; Tuberculous Tracheobronchial Adenopathy; Tuberculosis Confirmed Histologically |
| 012.16 | Tuberculosis of Intrathoracic Lymph Nodes; Tuberculosis of Lymph Nodes, Hilar, Mediastinal, Tracheobronchial; Tuberculous Tracheobronchial Adenopathy; Tuberculosis Confirmed by Other Methods [Inoculation of Animals] |
| 012.2 | Isolated Tracheal or Bronchial Tuberculosis |
| 012.20 | Isolated Tracheal or Bronchial Tuberculosis, Unspecified |
| 012.21 | Isolated Tracheal or Bronchial Tuberculosis, Bacterial or Histological Examination Not Done |
| 012.22 | Isolated Tracheal or Bronchial Tuberculosis, Bacterial or Histological Examination Unknown |
| 012.23 | Isolated Tracheal or Bronchial Tuberculosis, Tubercle Bacilli Found (in Sputum) by Microscopy |
| 012.24 | Isolated Tracheal or Bronchial Tuberculosis, Tubercle Bacilli Found by Bacterial Culture |
| 012.25 | Isolated Tracheal or Bronchial Tuberculosis, Tuberculosis Confirmed Histologically |
| 012.26 | Isolated Tracheal or Bronchial Tuberculosis; Tuberculosis Confirmed by Other Methods [Inoculation of Animals] |
| 012.3 | Tuberculous Laryngitis; Tuberculosis of Glottis |
| 012.30 | Tuberculous Laryngitis; Tuberculosis of Glottis, Unspecified |
| 012.31 | Tuberculous Laryngitis; Tuberculosis of Glottis; Bacterial or Histological Examination Not Done |
| 012.32 | Tuberculous Laryngitis; Tuberculosis of Glottis; Bacterial or Histological Examination Unknown |
| 012.33 | Tuberculous Laryngitis; Tuberculosis of Glottis; Tubercle Bacilli Found (in Sputum) by Microscopy |
| 012.34 | Tuberculous Laryngitis; Tuberculosis of Glottis; Tubercle Bacilli Found by Bacterial Culture |
| 012.35 | Tuberculous Laryngitis; Tuberculosis of Glottis; Tuberculosis Confirmed Histologically |
| 012.36 | Tuberculous Laryngitis; Tuberculosis of Glottis; Tuberculosis Confirmed by Other Methods [Inoculation of Animals] |
| 012.8 | Respiratory Tuberculosis, Other Specified; Tuberculosis of Mediastinum, Nasopharynx, Nose (Septum), Sinus, Any Nasal |
| 012.80 | Respiratory Tuberculosis, Other Specified; Tuberculosis of Mediastinum, Nasopharynx, Nose (Septum), Sinus, Any Nasal; Unspecified |
| 012.81 | Respiratory Tuberculosis, Other Specified; Tuberculosis of Mediastinum, Nasopharynx, Nose (Septum), Sinus, Any Nasal; Bacterial or Histological Examination Not Done |
| 012.82 | Respiratory Tuberculosis, Other Specified; Tuberculosis of Mediastinum, Nasopharynx, Nose (Septum), Sinus, Any Nasal; Bacterial or Histological Examination Unknown |
| 012.83 | Respiratory Tuberculosis, Other Specified; Tuberculosis of Mediastinum, Nasopharynx, Nose (Septum), Sinus, Any Nasal; Tubercle Bacilli Found (in Sputum) by Microscopy |
| 012.84 | Respiratory Tuberculosis, Other Specified; Tuberculosis of Mediastinum, Nasopharynx, Nose (Septum), Sinus, Any Nasal; Tubercle Bacilli Found by Bacterial Culture |
| 012.85 | Respiratory Tuberculosis, Other Specified; Tuberculosis of Mediastinum, Nasopharynx, Nose (Septum), Sinus, Any Nasal; Found by Tuberculosis Confirmed Histologically |
| 012.86 | Respiratory Tuberculosis, Other Specified; Tuberculosis of Mediastinum, Nasopharynx, Nose (Septum), Sinus, Any Nasal; Tuberculosis Confirmed by Other Methods [Inoculation of Animals] |
| 013 | Tuberculosis of Meninges and Central Nervous System |
| 013.0 | Tuberculous Meningitis; Tuberculosis of Meninges (Cerebral) (Spinal); Tuberculous, Leptomeningitis, Meningoencephalitis |
| 013.00 | Tuberculous Meningitis; Tuberculosis of Meninges (Cerebral) (Spinal); Tuberculous, Leptomeningitis, Meningoencephalitis; Unspecified |
| 013.01 | Tuberculous Meningitis; Tuberculosis of Meninges (Cerebral) (Spinal); Tuberculous, Leptomeningitis, Meningoencephalitis; Bacterial or Histological Examination Not Done |
| 013.02 | Tuberculous Meningitis; Tuberculosis of Meninges (Cerebral) (Spinal); Tuberculous, Leptomeningitis, Meningoencephalitis; Bacterial or Histological Examination Unknown |
| 013.03 | Tuberculous Meningitis; Tuberculosis of Meninges (Cerebral) (Spinal); Tuberculous, Leptomeningitis, Meningoencephalitis; Tubercle Bacilli Found (in Sputum) by Microscopy |
| 013.04 | Tuberculous Meningitis; Tuberculosis of Meninges (Cerebral) (Spinal); Tuberculous, Leptomeningitis, Meningoencephalitis; Tubercle Bacilli Found by Bacterial Culture |
| 013.05 | Tuberculous Meningitis; Tuberculosis of Meninges (Cerebral) (Spinal); Tuberculous, Leptomeningitis, Meningoencephalitis; Tuberculosis Confirmed Histologically |
| 013.06 | Tuberculous Meningitis; Tuberculosis of Meninges (Cerebral) (Spinal); Tuberculous, Leptomeningitis, Meningoencephalitis; Tuberculosis Confirmed by Other Methods [Inoculation of Animals] |
| 013.1 | Tuberculoma of Meninges |
| 013.10 | Tuberculoma of Meninges, Unspecified |
| 013.11 | Tuberculoma of Meninges, Bacterial or Histological Examination Not Done |
| 013.12 | Tuberculoma of Meninges, Bacterial or Histological Examination Unknown |
| 013.13 | Tuberculoma of Meninges, Tubercle Bacilli Found (in Sputum) by Microscopy |
| 013.14 | Tuberculoma of Meninges, Tubercle Bacilli Found by Bacterial Culture |
| 013.15 | Tuberculoma of Meninges, Tuberculosis Confirmed Histologically |
| 013.16 | Tuberculoma of Meninges, Tuberculosis Confirmed by Other Methods [Inoculation of Animals] |
| 013.2 | Tuberculoma of Brain; Tuberculosis of Brain, Current Disease |
| 013.20 | Tuberculoma of Brain; Tuberculosis of Brain, Current Disease; Unspecified |
| 013.21 | Tuberculoma of Brain; Tuberculosis of Brain, Current Disease; Bacterial or Histological Examination Not Done |
| 013.22 | Tuberculoma of Brain; Tuberculosis of Brain, Current Disease; Bacterial or Histological Examination Unknown |
| 013.23 | Tuberculoma of Brain; Tuberculosis of Brain, Current Disease; Tubercle Bacilli Found (in Sputum) by Microscopy |
| 013.24 | Tuberculoma of Brain; Tuberculosis of Brain, Current Disease; Tubercle Bacilli not Found by Bacterial Culture |
| 013.25 | Tuberculoma of Brain; Tuberculosis of Brain, Current Disease; Tuberculosis Confirmed Histologically |
| 013.26 | Tuberculoma of Brain; Tuberculosis of Brain, Current Disease; Tubercle Bacilli Tuberculosis Confirmed by Other Methods [Inoculation of Animals] |
| 013.3 | Tuberculous Abscess of Brain |
| 013.30 | Tuberculous Abscess of Brain, Unspecified |
| 013.31 | Tuberculous Abscess of Brain, Bacterial or Histological Examination Not Done |
| 013.32 | Tuberculous Abscess of Brain, Bacterial or Histological Examination Unknown |
| 013.33 | Tuberculous Abscess of Brain, Tubercle Bacilli Found (in Sputum) by Microscopy |
| 013.34 | Tuberculous Abscess of Brain, Tubercle Bacilli Found by Bacterial Culture |
| 013.35 | Tuberculous Abscess of Brain, Tuberculosis Confirmed Histologically |
| 013.36 | Tuberculous Abscess of Brain, Tubercle Bacilli Confirmed by Other Methods, Inoculation of Animals |
| 013.4 | Tuberculoma of Spinal Cord |
| 013.40 | Tuberculoma of Spinal Cord, Unspecified |
| 013.41 | Tuberculoma of Spinal Cord, Bacterial or Histological Examination Not Done |
| 013.42 | Tuberculoma of Spinal Cord, Bacterial or Histological Examination Unknown |
| 013.43 | Tuberculoma of Spinal Cord, Tubercle Bacilli Found (in Sputum) by Microscopy |
| 013.44 | Tuberculoma of Spinal Cord, Tubercle Bacilli Found by Bacterial Culture |
| 013.45 | Tuberculoma of Spinal Cord, Tuberculosis Confirmed Histologically |
| 013.46 | Tuberculoma of Spinal Cord, Tubercle Bacilli Tuberculosis Confirmed by Other Methods [Inoculation of Animals] |
| 013.5 | Tuberculous Abscess of Spinal Cord |
| 013.50 | Tuberculous Abscess of Spinal Cord, Unspecified |
| 013.51 | Tuberculous Abscess of Spinal Cord, Bacterial or Histological Examination Not Done |
| 013.52 | Tuberculous Abscess of Spinal Cord, Bacterial or Histological Examination Unknown |
| 013.53 | Tuberculous Abscess of Spinal Cord, Tubercle Bacilli Found (in Sputum) by Microscopy |
| 013.54 | Tuberculous Abscess of Spinal Cord, Tubercle Bacilli Found by Bacterial Culture |
| 013.55 | Tuberculous Abscess of Spinal Cord, Tuberculosis Confirmed Histologically |
| 013.56 | Tuberculous Abscess of Spinal Cord, Tuberculosis Confirmed by Other Methods [Inoculation of Animals] |
| 013.6 | Tuberculous Encephalitis or Myelitis |
| 013.60 | Tuberculous Encephalitis or Myelitis, Unspecified |
| 013.61 | Tuberculous Encephalitis or Myelitis, Bacterial or Histological Examination Not Done |
| 013.62 | Tuberculous Encephalitis or Myelitis, Bacterial or Histological Examination Unknown |
| 013.63 | Tuberculous Encephalitis or Myelitis, Tubercle Bacilli Found (in Sputum) by Microscopy |
| 013.64 | Tuberculous Encephalitis or Myelitis, Tubercle Bacilli Found by Bacterial Culture |
| 013.65 | Tuberculous Encephalitis or Myelitis, Tuberculosis Confirmed Histologically |
| 013.66 | Tuberculous Encephalitis or Myelitis, Tuberculosis Confirmed by Other Methods [Inoculation of Animals] |
| 013.8 | Tuberculosis of Central Nervous System, Other Specified |
| 013.80 | Tuberculosis of Central Nervous System, Other Specified; Confirmation Unspecified |
| 013.81 | Tuberculosis of Central Nervous System, Other Specified; Bacterial or Histological Examination Not Done |
| 013.82 | Tuberculosis of Central Nervous System, Other Specified; Bacterial or Histological Examination Unknown |
| 013.83 | Tuberculosis of Central Nervous System, Other Specified; Tubercle Bacilli Found (in Sputum) by Microscopy |
| 013.84 | Tuberculosis of Central Nervous System, Other Specified; Tubercle Bacilli Found by Bacterial Culture |
| 013.85 | Tuberculosis of Central Nervous System, Other Specified; Tuberculosis Confirmed Histologically |
| 013.86 | Tuberculosis of Central Nervous System, Other Specified; Tuberculosis Confirmed by Other Methods [Inoculation of Animals] |
| 013.9 | Tuberculosis of Central Nervous System, Unspecified; Tuberculosis of Central Nervous System, NOS |
| 013.90 | Tuberculosis of Central Nervous System, Unspecified; Tuberculosis of Central Nervous System, NOS; Confirmation Unspecified |
| 013.91 | Tuberculosis of Central Nervous System, Unspecified; Tuberculosis of Central Nervous System, NOS; Bacterial or Histological Examination Not Done |
| 013.92 | Tuberculosis of Central Nervous System, Unspecified; Tuberculosis of Central Nervous System, NOS; Bacterial or Histological Examination Unknown |
| 013.93 | Tuberculosis of Central Nervous System, Unspecified; Tuberculosis of Central Nervous System, NOS; Tubercle Bacilli Found (in Sputum) by Microscopy |
| 013.94 | Tuberculosis of Central Nervous System, Unspecified; Tuberculosis of Central Nervous System, NOS; Tubercle Bacilli Found by Bacterial Culture |
| 013.95 | Tuberculosis of Central Nervous System, Unspecified; Tuberculosis of Central Nervous System, NOS; Tubercle Bacilli Found Tuberculosis Confirmed Histologically |
| 013.96 | Tuberculosis of Central Nervous System, Unspecified; Tuberculosis of Central Nervous System, NOS; Tuberculosis Confirmed by Other Methods [Inoculation of Animals] |
| 014 | Tuberculosis of Intestines, Peritoneum, and Mesenteric Glands |
| 014.0 | Tuberculous Peritonitis; Tuberculous Ascites |
| 014.00 | Tuberculous Peritonitis; Tuberculous Ascites; Unspecified |
| 014.01 | Tuberculous Peritonitis; Tuberculous Ascites; Bacterial or Histological Examination Not Done |
| 014.02 | Tuberculous Peritonitis; Tuberculous Ascites; Bacterial or Histological Examination Unknown |
| 014.03 | Tuberculous Peritonitis; Tuberculous Ascites; Tubercle Bacilli Found (in Sputum) by Microscopy |
| 014.04 | Tuberculous Peritonitis; Tuberculous Ascites; Tubercle Bacilli Found by Bacterial Culture |
| 014.05 | Tuberculous Peritonitis; Tuberculous Ascites; Tuberculosis Confirmed Histologically |
| 014.06 | Tuberculous Peritonitis; Tuberculous Ascites; Tuberculosis Confirmed by Other Methods [Inoculation of Animals] |
| 014.8 | Tuberculosis of Intestines, Peritoneum, and Mesenteric Glands, Other; Tuberculosis of Anus, Intestine (Large) (Small), Mesenteric Glands, Rectum, Retroperitoneal (Lymph Nodes); Tuberculous Enteritis |
| 014.80 | Tuberculosis of Intestines, Peritoneum, and Mesenteric Glands, Other; Tuberculosis of Anus, Intestine (Large) (Small), Mesenteric Glands, Rectum, Retroperitoneal (Lymph Nodes); Tuberculous Enteritis; Unspecified |
| 014.81 | Tuberculosis of Intestines, Peritoneum, and Mesenteric Glands, Other; Tuberculosis of Anus, Intestine (Large) (Small), Mesenteric Glands, Rectum, Retroperitoneal (Lymph Nodes); Tuberculous Enteritis; Bacterial or Histological Examination Not Done |
| 014.82 | Tuberculosis of Intestines, Peritoneum, and Mesenteric Glands, Other; Tuberculosis of Anus, Intestine (Large) (Small), Mesenteric Glands, Rectum, Retroperitoneal (Lymph Nodes); Tuberculous Enteritis; Bacterial or Histological Examination Unknown |
| 014.83 | Tuberculosis of Intestines, Peritoneum, and Mesenteric Glands, Other; Tuberculosis of Anus, Intestine (Large) (Small), Mesenteric Glands, Rectum, Retroperitoneal (Lymph Nodes); Tuberculous Enteritis; Tubercle Bacilli Found (in Sputum) by Microscopy |
| 014.84 | Tuberculosis of Intestines, Peritoneum, and Mesenteric Glands, Other; Tuberculosis of Anus, Intestine (Large) (Small), Mesenteric Glands, Rectum, Retroperitoneal (Lymph Nodes); Tuberculous Enteritis; Tubercle Bacilli Found by Bacterial Culture |
| 014.85 | Tuberculosis of Intestines, Peritoneum, and Mesenteric Glands, Other; Tuberculosis of Anus, Intestine (Large) (Small), Mesenteric Glands, Rectum, Retroperitoneal (Lymph Nodes); Tuberculous Enteritis; Tuberculosis Confirmed Histologically |
| 014.86 | Tuberculosis of Intestines, Peritoneum, and Mesenteric Glands, Other; Tuberculosis of Anus, Intestine (Large) (Small), Mesenteric Glands, Rectum, Retroperitoneal (Lymph Nodes); Tuberculous Enteritis; Tuberculosis Confirmed by Other Methods [Inoculation of Animals] |
| 015 | Tuberculosis of Bones and Joints |
| 015.0 | Tuberculosis of Vertebral Column; Pott's Disease |
| 015.00 | Tuberculosis of Vertebral Column; Pott's Disease; Unspecified |
| 015.01 | Tuberculosis of Vertebral Column; Pott's Disease; Bacterial or Histological Examination Not Done |
| 015.02 | Tuberculosis of Vertebral Column; Pott's Disease; Bacterial or Histological Examination Unknown |
| 015.03 | Tuberculosis of Vertebral Column; Pott's Disease; Tubercle Bacilli Found (in Sputum) by Microscopy |
| 015.04 | Tuberculosis of Vertebral Column; Pott's Disease; Tubercle Bacilli Found by Bacterial Culture |
| 015.05 | Tuberculosis of Vertebral Column; Pott's Disease; Tuberculosis Confirmed Histologically |
| 015.06 | Tuberculosis of Vertebral Column; Pott's Disease; Tuberculosis Confirmed by Other Methods [Inoculation of Animals] |
| 015.1 | Tuberculosis of Hip |
| 015.10 | Tuberculosis of Hip, Unspecified |
| 015.11 | Tuberculosis of Hip, Bacterial or Histological Examination Not Done |
| 015.12 | Tuberculosis of Hip, Bacterial or Histological Examination Unknown |
| 015.13 | Tuberculosis of Hip, Tubercle Bacilli Found (in Sputum) by Microscopy |
| 015.14 | Tuberculosis of Hip, Tubercle Bacilli Found by Bacterial Culture |
| 015.15 | Tuberculosis of Hip, Tubercle Bacilli Found by Tuberculosis Confirmed Histologically |
| 015.16 | Tuberculosis of Hip, Tuberculosis Confirmed by Other Methods [Inoculation of Animals] |
| 015.2 | Tuberculosis of Knee |
| 015.20 | Tuberculosis of Knee, Unspecified |
| 015.21 | Tuberculosis of Knee, Bacterial or Histological Examination Not Done |
| 015.22 | Tuberculosis of Knee, Bacterial or Histological Examination Unknown |
| 015.23 | Tuberculosis of Knee, Tubercle Bacilli Found (in Sputum) by Microscopy |
| 015.24 | Tuberculosis of Knee, Tubercle Bacilli Found by Bacterial Culture |
| 015.25 | Tuberculosis of Knee, Tuberculosis Confirmed Histologically |
| 015.26 | Tuberculosis of Knee, Tuberculosis Confirmed by Other Methods [Inoculation of Animals] |
| 015.5 | Tuberculosis of Limb Bones; Tuberculous Dactylitis |
| 015.50 | Tuberculosis of Limb Bones; Tuberculous Dactylitis; Unspecified |
| 015.51 | Tuberculosis of Limb Bones; Tuberculous Dactylitis; Bacterial or Histological Examination Not Done |
| 015.52 | Tuberculosis of Limb Bones; Tuberculous Dactylitis; Bacterial or Histological Examination Unknown |
| 015.53 | Tuberculosis of Limb Bones; Tuberculous Dactylitis; Tubercle Bacilli Found (in Sputum) by Microscopy |
| 015.54 | Tuberculosis of Limb Bones; Tuberculous Dactylitis; Tubercle Bacilli Found by Bacterial Culture |
| 015.55 | Tuberculosis of Limb Bones; Tuberculous Dactylitis; Tuberculosis Confirmed Histologically |
| 015.56 | Tuberculosis of Limb Bones; Tuberculous Dactylitis; Tuberculosis Confirmed by Other Methods [Inoculation of Animals] |
| 015.6 | Tuberculosis of Mastoid; Tuberculous Mastoiditis |
| 015.60 | Tuberculosis of Mastoid; Tuberculous Mastoiditis; Unspecified |
| 015.61 | Tuberculosis of Mastoid; Tuberculous Mastoiditis; Bacterial or Histological Examination Not Done |
| 015.62 | Tuberculosis of Mastoid; Tuberculous Mastoiditis; Bacterial or Histological Examination Unknown |
| 015.63 | Tuberculosis of Mastoid; Tuberculous Mastoiditis; Tubercle Bacilli Found (in Sputum) by Microscopy |
| 015.64 | Tuberculosis of Mastoid; Tuberculous Mastoiditis; Tubercle Bacilli Found by Bacterial Culture |
| 015.65 | Tuberculosis of Mastoid; Tuberculous Mastoiditis; Tuberculosis Confirmed Histologically |
| 015.66 | Tuberculosis of Mastoid; Tuberculous Mastoiditis; Tuberculosis Confirmed by Other Methods [Inoculation of Animals] |
| 015.7 | Tuberculosis of Bones and Joints, Other Specified Bone |
| 015.70 | Tuberculosis of Bones and Joints, Other Specified Bone; Confirmation Unspecified |
| 015.71 | Tuberculosis of Bones and Joints, Other Specified Bone; Bacterial or Histological Examination Not Done |
| 015.72 | Tuberculosis of Bones and Joints, Other Specified Bone; Bacterial or Histological Examination Unknown |
| 015.73 | Tuberculosis of Bones and Joints, Other Specified Bone; Tubercle Bacilli Found (in Sputum) by Microscopy |
| 015.74 | Tuberculosis of Bones and Joints, Other Specified Bone; Tubercle Bacilli Found by Bacterial Culture |
| 015.75 | Tuberculosis of Bones and Joints; Other Specified Bone; Tuberculosis Confirmed Histologically |
| 015.76 | Tuberculosis of Bones and Joints; Other Specified Bone; Tuberculosis Confirmed by Other Methods [Inoculation of Animals] |
| 015.8 | Tuberculosis of Bones and Joints; Other Specified Joint |
| 015.80 | Tuberculosis of Bones and Joints; Other Specified Joint; Confirmation Unspecified |
| 015.81 | Tuberculosis of Bones and Joints; Other Specified Joint; Bacterial or Histological Examination Not Done |
| 015.82 | Tuberculosis of Bones and Joints; Other Specified Joint; Bacterial or Histological Examination Unknown |
| 015.83 | Tuberculosis of Bones and Joints; Other Specified Joint; Tubercle Bacilli Found (in Sputum) by Microscopy |
| 015.84 | Tuberculosis of Bones and Joints; Other Specified Joint; Tubercle Bacilli Found by Bacterial Culture |
| 015.85 | Tuberculosis of Bones and Joints; Other Specified Joint; Tuberculosis Confirmed Histologically |
| 015.86 | Tuberculosis of Bones and Joints; Other Specified Joint; Tuberculosis Confirmed by Other Methods [Inoculation of Animals] |
| 015.9 | Tuberculosis of Unspecified Bones and Joints |
| 015.90 | Tuberculosis of Unspecified Bones and Joints, Confirmation Unspecified |
| 015.91 | Tuberculosis of Unspecified Bones and Joints, Bacterial or Histological Examination Not Done |
| 015.92 | Tuberculosis of Unspecified Bones and Joints, Bacterial or Histological Examination Unknown |
| 015.93 | Tuberculosis of Unspecified Bones and Joints, Tubercle Bacilli Found (in Sputum) by Microscopy |
| 015.94 | Tuberculosis of Unspecified Bones and Joints, Tubercle Bacilli Found by Bacterial Culture |
| 015.95 | Tuberculosis of Unspecified Bones and Joints, Tuberculosis Confirmed Histologically |
| 015.96 | Tuberculosis of Unspecified Bones and Joints, Tuberculosis Confirmed by Other Methods [Inoculation of Animals] |
| 016 | Tuberculosis of Genitourinary System |
| 016.0 | Tuberculosis of Genitourinary system; Kidney; Renal Tuberculosis |
| 016.00 | Tuberculosis of Genitourinary System; Kidney; Renal Tuberculosis; Unspecified |
| 016.01 | Tuberculosis of Genitourinary System; Kidney; Renal Tuberculosis; Bacterial or Histological Examination Not Done |
| 016.02 | Tuberculosis of Genitourinary System; Kidney; Renal Tuberculosis; Bacterial or Histological Examination Unknown |
| 016.03 | Tuberculosis of Genitourinary System; Kidney; Renal Tuberculosis; Tubercle Bacilli Found (in Sputum) by Microscopy |
| 016.04 | Tuberculosis of Genitourinary System; Kidney; Renal Tuberculosis; Tubercle Bacilli Found by Bacterial Culture |
| 016.05 | Tuberculosis of Genitourinary System; Kidney; Renal Tuberculosis; Tuberculosis Confirmed Histologically |
| 016.06 | Tuberculosis of Genitourinary System; Kidney; Renal Tuberculosis; Tuberculosis Confirmed by Other Methods [Inoculation of Animals] |
| 016.1 | Tuberculosis of Bladder |
| 016.10 | Tuberculosis of Bladder, Unspecified |
| 016.11 | Tuberculosis of Bladder, Bacterial or Histological Examination Not Done |
| 016.12 | Tuberculosis of Bladder, Bacterial or Histological Examination Unknown |
| 016.13 | Tuberculosis of Bladder, Tubercle Bacilli Found (in Sputum) by Microscopy |
| 016.14 | Tuberculosis of Bladder, Tubercle Bacilli Found by Bacterial Culture |
| 016.15 | Tuberculosis of Bladder, Tuberculosis Confirmed Histologically |
| 016.16 | Tuberculosis of Bladder, Tuberculosis Confirmed by Other Methods [Inoculation of Animals] |
| 016.2 | Tuberculosis of Ureter |
| 016.20 | Tuberculosis of Ureter, Unspecified |
| 016.21 | Tuberculosis of Ureter, Bacterial or Histological Examination Not Done |
| 016.22 | Tuberculosis of Ureter, Bacterial or Histological Examination Unknown |
| 016.23 | Tuberculosis of Ureter, Tubercle Bacilli Found (in Sputum) by Microscopy |
| 016.24 | Tuberculosis of Ureter, Tubercle Bacilli Found by Bacterial Culture |
| 016.25 | Tuberculosis of Ureter, Tuberculosis Confirmed Histologically |
| 016.26 | Tuberculosis of Ureter, Tuberculosis Confirmed by Other Methods [Inoculation of Animals] |
| 016.3 | Tuberculosis of Genitourinary System, Other Urinary Organs |
| 016.30 | Tuberculosis of Genitourinary System, Other Urinary Organs; Unspecified |
| 016.31 | Tuberculosis of Genitourinary System, Other Urinary Organs; Bacterial or Histological Examination Not Done |
| 016.32 | Tuberculosis of Genitourinary System, Other Urinary Organs; Bacterial or Histological Examination Unknown |
| 016.33 | Tuberculosis of Genitourinary System, Other Urinary Organs; Tubercle Bacilli Found (in Sputum) by Microscopy |
| 016.34 | Tuberculosis of Genitourinary System, Other Urinary Organs; Tubercle Bacilli Found by Bacterial Culture |
| 016.35 | Tuberculosis of Genitourinary System, Other Urinary Organs; Tuberculosis Confirmed Histologically |
| 016.36 | Tuberculosis of Genitourinary System, Other Urinary Organs; Tuberculosis Confirmed by Other Methods [Inoculation of Animals] |
| 016.4 | Tuberculosis of Epididymis |
| 016.40 | Tuberculosis of Epididymis, Unspecified |
| 016.41 | Tuberculosis of Epididymis, Bacterial or Histological Examination Not Done |
| 016.42 | Tuberculosis of Epididymis, Bacterial or Histological Examination Unknown |
| 016.43 | Tuberculosis of Epididymis, Tubercle Bacilli Found (in Sputum) by Microscopy |
| 016.44 | Tuberculosis of Epididymis, Tubercle Bacilli Found by Bacterial Culture |
| 016.45 | Tuberculosis of Epididymis, Tuberculosis Confirmed Histologically |
| 016.46 | Tuberculosis of Epididymis, Tuberculosis Confirmed by Other Methods [Inoculation of Animals] |
| 016.5 | Tuberculosis of Genitourinary System, Other Male Genital Organs |
| 016.50 | Tuberculosis of Genitourinary System, Other Male Genital Organs; Unspecified |
| 016.51 | Tuberculosis of Genitourinary System, Other Male Genital Organs; Bacterial or Histological Examination Not Done |
| 016.52 | Tuberculosis of Genitourinary System, Other Male Genital Organs; Bacterial or Histological Examination Unknown |
| 016.53 | Tuberculosis of Genitourinary System, Other Male Genital Organs; Tubercle Bacilli Found (in Sputum) by Microscopy |
| 016.54 | Tuberculosis of Genitourinary System, Other Male Genital Organs; Tubercle Bacilli Found by Bacterial Culture |
| 016.55 | Tuberculosis of Genitourinary System, Other Male Genital Organs; Tuberculosis Confirmed Histologically |
| 016.56 | Tuberculosis of Genitourinary System, Other Male Genital Organs; Tuberculosis Confirmed by Other Methods [Inoculation of Animals] |
| 016.6 | Tuberculous Oophoritis and Salpingitis |
| 016.60 | Tuberculous Oophoritis and Salpingitis, Unspecified |
| 016.61 | Tuberculous Oophoritis and Salpingitis, Bacterial or Histological Examination Not Done |
| 016.62 | Tuberculous Oophoritis and Salpingitis, Bacterial or Histological Examination Unknown |
| 016.63 | Tuberculous Oophoritis and Salpingitis, Tuberculous Enteritis, Tubercle Bacilli Found (in Sputum) by Microscopy |
| 016.64 | Tuberculous Oophoritis and Salpingitis, Tubercle Bacilli Found by Bacterial Culture |
| 016.65 | Tuberculous Oophoritis and Salpingitis, Tuberculosis Confirmed Histologically |
| 016.66 | Tuberculous Oophoritis and Salpingitis, Tuberculosis Confirmed by Other Methods [Inoculation of Animals] |
| 016.7 | Tuberculosis of Genitourinary System, Other Female Genital Organs; Tuberculous Cervicitis, Endometritis |
| 016.70 | Tuberculosis of Genitourinary System, Other Female Genital Organs; Tuberculous Cervicitis, Endometritis; Unspecified |
| 016.71 | Tuberculosis of Genitourinary System, Other Female Genital Organs; Tuberculous Cervicitis, Endometritis; Bacterial or Histological Examination Not Done |
| 016.72 | Tuberculosis of Genitourinary System, Other Female Genital Organs; Tuberculous Cervicitis, Endometritis; Bacterial or Histological Examination Unknown |
| 016.73 | Tuberculosis of Genitourinary System, Other Female Genital Organs; Tuberculous Cervicitis, Endometritis; Tubercle Bacilli Found (in Sputum) by Microscopy |
| 016.74 | Tuberculosis of Genitourinary System, Other Female Genital Organs; Tuberculous Cervicitis, Endometritis; Tubercle Bacilli Found by Bacterial Culture |
| 016.75 | Tuberculosis of Genitourinary System, Other Female Genital Organs; Tuberculous Cervicitis, Endometritis; Tubercle Bacilli not Found by Bacteriological Examination, but Tuberculosis Confirmed Histologically |
| 016.76 | Tuberculosis of Genitourinary System, Other Female Genital Organs; Tuberculous Cervicitis, Endometritis; Tubercle Bacilli not Found by Bacteriological or Histological Examination, but Tuberculosis Confirmed by Other Methods [Inoculation of Animals] |
| 016.9 | Genitourinary Tuberculosis, Unspecified |
| 016.90 | Genitourinary Tuberculosis, Unspecified Type; Confirmation Unspecified |
| 016.91 | Genitourinary Tuberculosis, Unspecified; Bacterial or Histological Examination Not Done |
| 016.92 | Genitourinary Tuberculosis, Unspecified; Bacterial or Histological Examination Unknown |
| 016.93 | Genitourinary Tuberculosis, Unspecified; Tubercle Bacilli Found (in Sputum) by Microscopy |
| 016.94 | Genitourinary Tuberculosis, Unspecified; Tubercle Bacilli Found by Bacterial Culture |
| 016.95 | Genitourinary Tuberculosis, Unspecified; Tuberculosis Confirmed Histologically |
| 016.96 | Genitourinary Tuberculosis, Unspecified; Tuberculosis Confirmed by Other Methods [Inoculation of Animals] |
| 017 | Tuberculosis of Other Organs |
| 017.0 | Tuberculosis of Skin and Subcutaneous Cellular Tissue; Lupus Exedens, Vulgaris; Scrofuloderma; Tuberculosis Colliquativa, Cutis, Lichenoides, Papulonecrotica, Verrucosa Cutis |
| 017.00 | Tuberculosis of Skin and Subcutaneous Cellular Tissue; Lupus Exedens, Vulgaris; Scrofuloderma; Tuberculosis Colliquativa, Cutis, Lichenoides, Papulonecrotica, Verrucosa Cutis; Confirmation Unspecified |
| 017.01 | Tuberculosis of Skin and Subcutaneous Cellular Tissue; Lupus Exedens, Vulgaris; Scrofuloderma; Tuberculosis Colliquativa, Cutis, Lichenoides, Papulonecrotica, Verrucosa Cutis; Bacterial or Histological Examination Not Done |
| 017.02 | Tuberculosis of Skin and Subcutaneous Cellular Tissue; Lupus Exedens, Vulgaris; Scrofuloderma; Tuberculosis Colliquativa, Cutis, Lichenoides, Papulonecrotica, Verrucosa Cutis; Bacterial or Histological Examination Unknown |
| 017.03 | Tuberculosis of Skin and Subcutaneous Cellular Tissue; Lupus Exedens, Vulgaris; Scrofuloderma; Tuberculosis Colliquativa, Cutis, Lichenoides, Papulonecrotica, Verrucosa Cutis; Tubercle Bacilli Found (in Sputum) by Microscopy |
| 017.04 | Tuberculosis of Skin and Subcutaneous Cellular Tissue; Lupus Exedens, Vulgaris; Scrofuloderma; Tuberculosis Colliquativa, Cutis, Lichenoides, Papulonecrotica, Verrucosa Cutis; Tubercle Bacilli Found by Bacterial Culture |
| 017.05 | Tuberculosis of Skin and Subcutaneous Cellular Tissue; Lupus Exedens, Vulgaris; Scrofuloderma; Tuberculosis Colliquativa, Cutis, Lichenoides, Papulonecrotica, Verrucosa Cutis; Tuberculosis Confirmed Histologically |
| 017.06 | Tuberculosis of Skin and Subcutaneous Cellular Tissue; Lupus Exedens, Vulgaris; Scrofuloderma; Tuberculosis Colliquativa, Cutis, Lichenoides, Papulonecrotica, Verrucosa Cutis; Tuberculosis Confirmed by Other Methods [Inoculation of Animals] |
| 017.1 | Tuberculosis of Erythema Nodosum with Hypersensitivity Reaction in Tuberculosis; Bazin's Disease; Erythema Induratum, Nodosum, Tuberculous; Tuberculosis Indurativa |
| 017.10 | Tuberculosis of Erythema Nodosum with Hypersensitivity Reaction in Tuberculosis; Bazin's Disease; Erythema Induratum, Nodosum, Tuberculous; Tuberculosis Indurativa; Confirmation Unspecified |
| 017.11 | Tuberculosis of Erythema Nodosum with Hypersensitivity Reaction in Tuberculosis; Bazin's Disease; Erythema Induratum, Nodosum, Tuberculous; Tuberculosis Indurativa; Bacterial or Histological Examination Not Done |
| 017.12 | Tuberculosis of Erythema Nodosum with Hypersensitivity Reaction in Tuberculosis; Bazin's Disease; Erythema Induratum, Nodosum, Tuberculous; Tuberculosis Indurativa; Bacterial or Histological Examination Unknown |
| 017.13 | Tuberculosis of Erythema Nodosum with Hypersensitivity Reaction in Tuberculosis; Bazin's Disease; Erythema Induratum, Nodosum, Tuberculous; Tuberculosis Indurativa; Tubercle Bacilli Found (in Sputum) by Microscopy |
| 017.14 | Tuberculosis of Erythema Nodosum with Hypersensitivity Reaction in Tuberculosis; Bazin's Disease; Erythema Induratum, Nodosum, Tuberculous; Tuberculosis Indurativa; Tubercle Bacilli Found by Bacterial Culture |
| 017.15 | Tuberculosis of Erythema Nodosum with Hypersensitivity Reaction in Tuberculosis; Bazin's Disease; Erythema Induratum, Nodosum, Tuberculous; Tuberculosis Indurativa; Tuberculosis Confirmed Histologically |
| 017.16 | Tuberculosis of Erythema Nodosum with Hypersensitivity Reaction in Tuberculosis; Bazin's Disease; Erythema Induratum, Nodosum, Tuberculous; Tuberculosis Indurativa; Tuberculosis Confirmed by Other Methods [Inoculation of Animals] |
| 017.2 | Tuberculosis of Peripheral Lymph Nodes; Scrofula; Scrofulous Abscess; Tuberculous adenitis |
| 017.20 | Tuberculosis of Peripheral Lymph Nodes; Scrofula; Scrofulous Abscess; Tuberculous adenitis; Confirmation Unspecified |
| 017.21 | Tuberculosis of Peripheral Lymph Nodes; Scrofula; Scrofulous Abscess; Tuberculous adenitis; Bacterial or Histological Examination Not Done |
| 017.22 | Tuberculosis of Peripheral Lymph Nodes; Scrofula; Scrofulous Abscess; Tuberculous adenitis; Bacterial or Histological Examination Unknown |
| 017.23 | Tuberculosis of Peripheral Lymph Nodes; Scrofula; Scrofulous Abscess; Tuberculous adenitis; Tubercle Bacilli Found (in Sputum) by Microscopy |
| 017.24 | Tuberculosis of Peripheral Lymph Nodes; Scrofula; Scrofulous Abscess; Tuberculous adenitis; Tubercle Bacilli Found by Bacterial Culture |
| 017.25 | Tuberculosis of Peripheral Lymph Nodes; Scrofula; Scrofulous Abscess; Tuberculous adenitis; Tuberculosis Confirmed Histologically |
| 017.26 | Tuberculosis of Peripheral Lymph Nodes; Scrofula; Scrofulous Abscess; Tuberculous adenitis; Tuberculosis Confirmed by Other Methods [Inoculation of Animals] |
| 017.3 | Tuberculosis of Eye |
| 017.30 | Tuberculosis of Eye, Unspecified |
| 017.31 | Tuberculosis of Eye, Bacterial or Histological Examination Not Done |
| 017.32 | Tuberculosis of Eye, Bacterial or Histological Examination Unknown |
| 017.33 | Tuberculosis of Eye, Tubercle Bacilli Found (in Sputum) by Microscopy |
| 017.34 | Tuberculosis of Eye, Tubercle Bacilli Found by Bacterial Culture |
| 017.35 | Tuberculosis of Eye, Tuberculosis Confirmed Histologically |
| 017.36 | Tuberculosis of Eye, Tuberculosis Confirmed by Other Methods [Inoculation of Animals] |
| 017.4 | Tuberculosis of Ear |
| 017.40 | Tuberculosis of Ear; Tuberculous Otitis Media; Unspecified |
| 017.41 | Tuberculosis of Ear; Tuberculous Otitis Media; Bacterial or Histological Examination Not Done |
| 017.42 | Tuberculosis of Ear; Tuberculous Otitis Media; Bacterial or Histological Examination Unknown |
| 017.43 | Tuberculosis of Ear; Tuberculous Otitis Media; Tubercle Bacilli Found (in Sputum) by Microscopy |
| 017.44 | Tuberculosis of Ear; Tuberculous Otitis Media; Tubercle Bacilli Found by Bacterial Culture |
| 017.45 | Tuberculosis of Ear; Tuberculous Otitis Media; Tuberculosis Confirmed Histologically |
| 017.46 | Tuberculosis of Ear; Tuberculous Otitis Media; Tuberculosis Confirmed by Other Methods [Inoculation of Animals] |
| 017.5 | Tuberculosis of Thyroid Gland |
| 017.50 | Tuberculosis of Thyroid Gland, Unspecified |
| 017.51 | Tuberculosis of Thyroid Gland, Bacterial or Histological Examination Not Done |
| 017.52 | Tuberculosis of Thyroid Gland, Bacterial or Histological Examination Unknown |
| 017.53 | Tuberculosis of Thyroid Gland, Tubercle Bacilli Found (in Sputum) by Microscopy |
| 017.54 | Tuberculosis of Thyroid Gland, Tubercle Bacilli Found by Bacterial Culture |
| 017.55 | Tuberculosis of Thyroid Gland, Tuberculosis Confirmed Histologically |
| 017.56 | Tuberculosis of Thyroid Gland, Tuberculosis Confirmed by Other Methods [Inoculation of Animals] |
| 017.6 | Tuberculosis of Adrenal Glands; Addison's Disease, Tuberculous |
| 017.60 | Tuberculosis of Adrenal Glands; Addison's Disease, Tuberculous; Unspecified |
| 017.61 | Tuberculosis of Adrenal Glands; Addison's Disease, Tuberculous; Bacterial or Histological Examination Not Done |
| 017.62 | Tuberculosis of Adrenal Glands; Addison's Disease, Tuberculous; Bacterial or Histological Examination Unknown |
| 017.63 | Tuberculosis of Adrenal Glands; Addison's Disease, Tuberculous; Tubercle Bacilli Found (in Sputum) by Microscopy |
| 017.64 | Tuberculosis of Adrenal Glands; Addison's Disease, Tuberculous; Tubercle Bacilli Found by Bacterial Culture |
| 017.65 | Tuberculosis of Adrenal Glands; Addison's Disease, Tuberculous; Tuberculosis Confirmed Histologically |
| 017.66 | Tuberculosis of Adrenal Glands; Addison's Disease, Tuberculous; Tuberculosis Confirmed by Other Methods [Inoculation of Animals] |
| 017.7 | Tuberculosis of Spleen |
| 017.70 | Tuberculosis of Spleen, Unspecified |
| 017.71 | Tuberculosis of Spleen, Bacterial or Histological Examination Not Done |
| 017.72 | Tuberculosis of Spleen, Bacterial or Histological Examination Unknown |
| 017.73 | Tuberculosis of Spleen, Tubercle Bacilli Found (in Sputum) by Microscopy |
| 017.74 | Tuberculosis of Spleen, Tubercle Bacilli Found by Bacterial Culture |
| 017.75 | Tuberculosis of Spleen, Tuberculosis Confirmed Histologically |
| 017.76 | Tuberculosis of Spleen, Tuberculosis Confirmed by Other Methods [Inoculation of Animals] |
| 017.8 | Tuberculosis of Esophagus |
| 017.80 | Tuberculosis of Esophagus, Unspecified |
| 017.81 | Tuberculosis of Esophagus, Bacterial or Histological Examination Not Done |
| 017.82 | Tuberculosis of Esophagus, Bacterial or Histological Examination Unknown |
| 017.83 | Tuberculosis of Esophagus, Tubercle Bacilli Found (in Sputum) by Microscopy |
| 017.84 | Tuberculosis of Esophagus, Tubercle Bacilli Found by Bacterial Culture |
| 017.85 | Tuberculosis of Esophagus, Tuberculosis Confirmed Histologically |
| 017.86 | Tuberculosis of Esophagus, Tuberculosis Confirmed by Other Methods [Inoculation of Animals] |
| 017.9 | Tuberculosis of Other Specified Organs |
| 017.90 | Tuberculosis of Other Specified Organs, Unspecified |
| 017.91 | Tuberculosis of Other Specified Organs, Bacterial or Histological Examination Not Done |
| 017.92 | Tuberculosis of Other Specified Organs, Bacterial or Histological Examination Unknown |
| 017.93 | Tuberculosis of Other Specified Organs, Tubercle Bacilli Found (in Sputum) by Microscopy |
| 017.94 | Tuberculosis of Other Specified Organs, Tubercle Bacilli Found by Bacterial Culture |
| 017.95 | Tuberculosis of Other Specified Organs, Tuberculosis Confirmed Histologically |
| 017.96 | Tuberculosis of Other Specified Organs, Tuberculosis Confirmed by Other Methods [Inoculation of Animals] |
| 018 | Miliary Tuberculosis; Tuberculosis Disseminated, generalized; Tuberculosis, Miliary, Whether of a Single Specified Site, Multiple Sites, or Unspecified Site; Polyserositis |
| 018.0 | Acute Miliary Tuberculosis |
| 018.00 | Acute Miliary Tuberculosis, Unspecified |
| 018.01 | Acute Miliary Tuberculosis, Bacterial or Histological Examination Not Done |
| 018.02 | Acute Miliary Tuberculosis, Bacterial or Histological Examination Unknown |
| 018.03 | Acute Miliary Tuberculosis, Tubercle Bacilli Found (in Sputum) by Microscopy |
| 018.04 | Acute Miliary Tuberculosis, Tubercle Bacilli Found by Bacterial Culture |
| 018.05 | Acute Miliary Tuberculosis, Tuberculosis Confirmed Histologically |
| 018.06 | Acute Miliary Tuberculosis, Tuberculosis Confirmed by Other Methods [Inoculation of Animals] |
| 018.8 | Specified Miliary Tuberculosis, Other |
| 018.80 | Specified Miliary Tuberculosis, Other Type; Confirmation Unspecified |
| 018.81 | Specified Miliary Tuberculosis, Other; Bacterial or Histological Examination Not Done |
| 018.82 | Specified Miliary Tuberculosis, Other; Bacterial or Histological Examination Unknown |
| 018.83 | Specified Miliary Tuberculosis, Other; Tubercle Bacilli Found (in Sputum) by Microscopy |
| 018.84 | Specified Miliary Tuberculosis, Other; Tubercle Bacilli Found by Bacterial Culture |
| 018.85 | Specified Miliary Tuberculosis, Other; Tuberculosis Confirmed Histologically |
| 018.86 | Specified Miliary Tuberculosis, Other; Tuberculosis Confirmed by Other Methods [Inoculation of Animals] |
| 018.9 | Miliary Tuberculosis, Unspecified |
| 018.90 | Miliary Tuberculosis, Unspecified Type; Confirmation Unspecified |
| 018.91 | Miliary Tuberculosis, Unspecified; Bacterial or Histological Examination Not Done |
| 018.92 | Miliary Tuberculosis, Unspecified; Bacterial or Histological Examination Unknown |
| 018.93 | Miliary Tuberculosis, Unspecified; Tubercle Bacilli Found (in Sputum) by Microscopy |
| 018.94 | Miliary Tuberculosis, Unspecified; Tubercle Bacilli Found by Bacterial Culture |
| 018.95 | Miliary Tuberculosis, Unspecified; Tuberculosis Confirmed Histologically |
| 018.96 | Miliary Tuberculosis, Unspecified, Tuberculosis Confirmed by Other Methods [Inoculation of Animals] |
| 020 | Plague, Includes Infection by Yersinia pestis |
| 020.0 | Plague, Bubonic |
| 020.1 | Plague, Cellulocutaneous |
| 020.2 | Plague, Septicemic |
| 020.3 | Plague, Primary Pneumonic |
| 020.4 | Plague, Secondary Pneumonic |
| 020.5 | Plague, Pneumonic, Unspecified |
| 020.8 | Plague, Other Specific Types; Abortive plague; Ambulatory plague; Pestis minor |
| 020.9 | Plague, Unspecified |
| 021 | Tularemia; Includes Deerfly Fever; Rabbit Fever; Infection by Francisella Tularensis |
| 021.0 | Tularemia, Ulceroglandular |
| 021.1 | Tularemia, Enteric; Tularemia Cryptogenic, Intestinal, Typhoidal |
| 021.2 | Tularemia, Pulmonary; Bronchopneumonic Tularemia |
| 021.3 | Tularemia, Oculoglandular |
| 021.8 | Tularemia, Other Specified; Generalized or Disseminated Tularemia, Glandular |
| 021.9 | Tularemia, Unspecified |
| 022 | Anthrax |
| 022.0 | Anthrax, Cutaneous; Malignant Pustule |
| 022.1 | Anthrax, Pulmonary; Respiratory Anthrax; Wool-sorters' Disease |
| 022.2 | Anthrax, Gastrointestinal |
| 022.3 | Anthrax Septicemia |
| 022.8 | Anthrax, Other Specified Manifestations |
| 022.9 | Anthrax, Unspecified |
| 023 | Brucellosis, Includes Fever Malta, Mediterranean, Undulant |
| 023.0 | Brucella melitensis |
| 023.1 | Brucella abortus |
| 023.2 | Brucella suis |
| 023.3 | Brucella canis |
| 023.8 | Brucellosis, Other |
| 023.9 | Brucellosis, Unspecified |
| 024 | Glanders; Infection by Actinobacillus mallei, Malleomyces mallei, Pseudomonas mallei, Farcy, Malleus |
| 025 | Melioidosis; Infection by Malleomyces pseudomallei, Pseudomonas pseudomallei, Whitmore's bacillus; Pseudoglanders |
| 026 | Rat-bite Fever |
| 026.0 | Rat-bite Fever; Spirillary fever; Rat-bite fever Due to Spirillum minor [S. minus]; Sodoku |
| 026.1 | Rate-bite Fever; Streptobacillary Fever; Epidemic Arthritic Erythema; Haverhill Fever; Rat-bite Fever Due to Streptobacillus moniliformis |
| 026.9 | Rat-bite Fever, Unspecified |
| 027 | Zoonotic Bacterial Diseases, Other |
| 027.0 | Zoonotic Bacterial Diseases, Other; Listeriosis; Infection by Listeria Monocytogenes; Septicemia by Listeria Monocytogenes |
| 027.1 | Zoonotic Bacterial Diseases, Other; Erysipelothrix Infection, Erysipeloid (of Rosenbach), Infection of Erysipelothrix insidiosa, Septicemia by Erysipelothrix |
| 027.2 | Zoonotic Bacterial Diseases, Other Pasteurellosis; Pasteurella pseudotuberculosis Infection by Pasteurella multocida; Mesenteric adenitis by Pasteurella multocida; Septic Infection (Cat Bite) (Dog Bite) by Pasteurella multocida |
| 027.8 | Zoonotic Bacterial Diseases, Specified, Other |
| 027.9 | Zoonotic Bacterial Disease, Unspecified |
| 030 | Leprosy; Includes Hansen's Disease, Infection by Mycobacterium leprae |
| 030.0 | Leprosy; Lepromatous or Type L; Lepromatous Leprosy, Macular, Diffuse, Infiltrated, Nodular, Neuritic |
| 030.1 | Leprosy; Tuberculoid or Type T; Tuberculoid Leprosy, Macular, Maculoanesthetic, Major, Minor, Neuritic |
| 030.2 | Leprosy; Indeterminate or Group I; Indeterminate or Uncharacteristic Leprosy, Macular, Neuritic |
| 030.3 | Leprosy; Borderline or Group B; Borderline or Dimorphous Leprosy, Infiltrated, Neuritic |
| 030.8 | Leprosy, Specified, Other |
| 030.9 | Leprosy, Unspecified |
| 031 | Diseases Due to Other Mycobacteria |
| 031.0 | Diseases Due to Other Mycobacteria; Pulmonary; Infection by Mycobacterium, Avium, Intracellulare, Kansasii; Battey Disease |
| 031.1 | Diseases Due to Other Mycobacteria; Cutaneous; Buruli Ulcer; Infection by Mycobacterium, Marinum, Ulcerans |
| 031.2 | Diseases Due to Other Mycobacteria; Disseminated; Disseminated Mycobacterium Avium-intracellulare Complex (DMAC); Mycobacterium Avium-intracellulare Complex (MAC) Bacteremia |
| 031.8 | Diseases Due to Other Mycobacteria; Other Specified Mycobacterial Diseases |
| 031.9 | Diseases Due to Other Mycobacteria; Unspecified Diseases Due to Mycobacteria |
| 032 | Diphtheria, Includes Infection by Corynebacterium Diphtheriae |
| 032.0 | Diphtheria, Faucial; Membranous Angina, Diphtheritic |
| 032.1 | Diphtheria, Nasopharyngeal |
| 032.2 | Anterior Nasal Diphtheria |
| 032.3 | Diphtheria, Laryngeal; Laryngotracheitis, Diphtheritic |
| 032.8 | Diphtheria, Other Specified |
| 032.81 | Diphtheria, Conjunctival; Pseudomembranous Diphtheritic Conjunctivitis |
| 032.82 | Diphtheritic Myocarditis |
| 032.83 | Diphtheritic Peritonitis |
| 032.84 | Diphtheritic Cystitis |
| 032.85 | Cutaneous Diphtheria |
| 032.89 | Diphtheria, Other Specified, Other |
| 032.9 | Diphtheria, Unspecified |
| 033 | Whooping Cough, Includes Pertussis |
| 033.0 | Whooping Cough; Bordetella pertussis |
| 033.1 | Whooping Cough; Bordetella parapertussis |
| 033.8 | Whooping Cough Due to Other Specified Organism; Bordetella bronchiseptica |
| 033.9 | Whooping Cough, Unspecified Organism |
| 034 | Streptococcal Sore Throat and Scarlet Fever |
| 034.0 | Streptococcal Sore Throat; Septic Angina, Sore Throat; Streptococcal Angina, Laryngitis, Pharyngitis, Tonsillitis |
| 034.1 | Scarlet Fever; Scarlatina |
| 035 | Erysipelas |
| 036 | Meningococcal Infection |
| 036.0 | Meningococcal Meningitis; Cerebrospinal Fever, Meningococcal; Cerebrospinal, Epidemic |
| 036.1 | Meningococcal Encephalitis |
| 036.2 | Meningococcemia; Meningococcal Septicemia |
| 036.3 | Waterhouse-Friderichsen Syndrome, Meningococcal; Meningococcal Hemorrhagic Adrenalitis; Meningococcic Adrenal Syndrome; Waterhouse-Friderichsen Syndrome, NOS |
| 036.4 | Meningococcal Carditis |
| 036.40 | Meningococcal Carditis, Unspecified |
| 036.41 | Meningococcal Pericarditis |
| 036.42 | Meningococcal Endocarditis |
| 036.43 | Meningococcal Myocarditis |
| 036.8 | Meningococcal Infections, Specified, Other |
| 036.81 | Meningococcal Optic Neuritis |
| 036.82 | Meningococcal Arthropathy |
| 036.89 | Meningococcal Infections, Other Specified; Other |
| 036.9 | Meningococcal Infection, Unspecified; Meningococcal Infection, NOS |
| 037 | Tetanus |
| 038 | Septicemia |
| 038.0 | Streptococcal Septicemia |
| 038.1 | Staphylococcal Septicemia |
| 038.10 | Staphylococcal Septicemia, Unspecified |
| 038.11 | Methicillin susceptible Staphylococcus Aureus Septicemia; MSSA septicemia; Staphylococcus aureus septicemia NOS |
| 038.12 | Methicillin resistant Staphylococcus aureus septicemia |
| 038.19 | Staphylococcal Septicemia, Other |
| 038.2 | Pneumococcal Septicemia |
| 038.3 | Septicemia Due to Anaerobes; Septicemia Due to Bacteroides |
| 038.4 | Septicemia Due to Other Gram-negative Organisms |
| 038.40 | Septicemia Due to Other Gram-negative Organisms; Gram-negative organism, Unspecified; Gram-negative Septicemia NOS |
| 038.41 | Septicemia Due to Other Gram-negative Organisms; Hemophilus influenzae [H. influenzae] |
| 038.42 | Septicemia Due to Other Gram-negative Organisms; Escherichia coli [E. coli] |
| 038.43 | Septicemia Due to Other Gram-negative Organisms; Pseudomonas |
| 038.44 | Septicemia Due to Other Gram-negative Organisms; Serratia |
| 038.49 | Septicemia Due to Other Gram-negative Organisms, Other |
| 038.8 | Septicemias, Other Specified |
| 038.9 | Septicemia, Unspecified; Septicemia, NOS |
| 039 | Actinomycotic Infections |
| 039.0 | Actinomycotic Infection; Cutaneous, Erythrasma, Trichomycosis Axillaris |
| 039.1 | Actinomycotic Infection; Pulmonary, Thoracic Actinomycosis |
| 039.2 | Actinomycotic Infection, Abdominal |
| 039.3 | Actinomycotic Infection, Cervicofacial |
| 039.4 | Actinomycotic Infections; Madura Foot |
| 039.8 | Actinomycotic Infections, Other Specified Sites |
| 039.9 | Actinomycosis Infections, Unspecified Site; Actinomycosis NOS, Maduromycosis NOS, Nocardiosis NOS |
| 040 | Bacterial Diseases, Other |
| 040.0 | Bacterial Diseases, Other; Gas Gangrene; Gas Bacillus Infection or Gangrene; Infection by Clostridium, Histolyticum, Oedematiens, Perfringens, Septicum, Sordellii; Malignant Edema; Myonecrosis, Clostridial; Myositis, Clostridial |
| 040.1 | Bacterial Diseases, Other; Rhinoscleroma |
| 040.2 | Bacterial Diseases, Other; Whipple's Disease; Intestinal lipodystrophy |
| 040.3 | Bacterial Diseases, Other; Necrobacillosis |
| 040.4 | Bacterial Diseases, Other; Specified Botulism, Other, Non-Foodborne Intoxication Due to Toxins Of Clostridium Botulinum |
| 040.41 | Bacterial Diseases, Other; Infant Botulism |
| 040.42 | Bacterial Diseases, Other; Wound Botulism, Non-Foodborne Botulism NOS |
| 040.8 | Bacterial Diseases, Specific, Other |
| 040.81 | Bacterial Diseases, Specific, Other; Tropical Pyomyositis |
| 040.82 | Bacterial Diseases, Specific, Other; Toxic Shock Syndrome |
| 040.89 | Bacterial Diseases, Other Specific, Other |
| 041 | Bacterial Infection in Conditions Classified Elsewhere and of Unspecified Site |
| 041.0 | Streptococcus |
| 041.00 | Streptococcus, Unspecified |
| 041.01 | Streptococcus; Group A |
| 041.02 | Streptococcus; Group B |
| 041.03 | Streptococcus; Group C |
| 041.04 | Streptococcus; Group D [Enterococcus] |
| 041.05 | Streptococcus; Group G |
| 041.09 | Streptococcus, Other |
| 041.1 | Staphylococcus |
| 041.10 | Staphylococcus, Unspecified |
| 041.11 | Methicillin Susceptible Staphylococcus Aureus; MSSA; Staphylococcus aureus NOS |
| 041.12 | Methicillin Resistant Staphylococcus Aureus (MRSA) |
| 041.19 | Staphylococcus, Other |
| 041.2 | Pneumococcus |
| 041.3 | Klebsiella pneumoniae |
| 041.4 | Escherichia coli [E. Coli] |
| 041.41 | Shiga toxin-producing Escherichia coli [E. coli] (STEC) O157 |
| 041.42 | Other specified Shiga toxin-producing Escherichia coli [E. coli] (STEC) |
| 041.43 | Shiga toxin-producing Escherichia coli [E. coli] (STEC), unspecified |
| 041.49 | Other and unspecified Escherichia coli [E. coli] |
| 041.5 | Hemophilus influenzae [H. influenzae] |
| 041.6 | Proteus mirabilis, Proteus morganii |
| 041.7 | Pseudomonas |
| 041.8 | Bacterial Infections, Other Specified |
| 041.81 | Bacterial Infections, Other Specified; Mycoplasma; Eaton's Agent; Pleuropneumonia-like Organisms |
| 041.82 | Bacterial Infections, Other Specified; Bacteroides Fragilis |
| 041.83 | Bacterial Infections, Other Specified; Clostridium Perfringens |
| 041.84 | Bacterial Infections, Other Specified; Other Anaerobes; Gram-negative Anaerobes |
| 041.85 | Bacterial Infections, Other Specified; Other Gram-negative Organisms; Aerobacter Aerogenes; Mima Polymorpha; Serratia |
| 041.86 | Bacterial Infections, Other Specified; Helicobacter pylori (H. pylori) |
| 041.89 | Bacterial Infections, Other Specified; Bacterial, Other Specified |
| 041.9 | Bacterial Infection, Unspecified |
| 042 | Human Immunodeficiency Virus [HIV] Disease |
| 045 | Acute Poliomyelitis |
| 045.0 | Acute Paralytic Poliomyelitis Specified as Bulbar; Infantile Paralysis (Acute) Specified as Bulbar; Poliomyelitis (Acute) (Anterior) Specified as Bulbar; Polioencephalitis (Acute) (Bulbar); Polioencephalomyelitis (Acute) (Anterior) (Bulbar) |
| 045.00 | Acute Paralytic Poliomyelitis Specified as Bulbar; Infantile Paralysis (Acute) Specified as Bulbar; Poliomyelitis (Acute) (Anterior) Specified As Bulbar; Polioencephalitis (Acute) (Bulbar); Polioencephalomyelitis (Acute) (Anterior) (Bulbar); Poliovirus, Unspecified Type |
| 045.01 | Acute Paralytic Poliomyelitis Specified as Bulbar; Infantile Paralysis (Acute) Specified as Bulbar; Poliomyelitis (Acute) (Anterior) Specified as Bulbar; Polioencephalitis (Acute) (Bulbar); Polioencephalomyelitis (Acute) (Anterior) (Bulbar); Poliovirus Type I |
| 045.02 | Acute Paralytic Poliomyelitis Specified as Bulbar; Infantile Paralysis (Acute) Specified as Bulbar; Poliomyelitis (Acute) (Anterior) Specified as Bulbar; Polioencephalitis (Acute) (Bulbar); Polioencephalomyelitis (Acute) (Anterior) (Bulbar); Poliovirus Type II |
| 045.03 | Acute Paralytic Poliomyelitis Specified as Bulbar; Infantile Paralysis (acute) Specified as Bulbar; Poliomyelitis (Acute) (Anterior) specified as Bulbar; Polioencephalitis (Acute) (Bulbar); Polioencephalomyelitis (Acute) (Anterior) (Bulbar); Poliovirus Type III |
| 045.1 | Acute Poliomyelitis with Other Paralysis |
| 045.10 | Acute Poliomyelitis with Other Paralysis, Poliovirus, Unspecified Type |
| 045.11 | Acute Poliomyelitis with Other Paralysis, Poliovirus Type I |
| 045.12 | Acute Poliomyelitis with Other Paralysis, Poliovirus Type II |
| 045.13 | Acute Poliomyelitis with Other Paralysis, Poliovirus Type III |
| 045.2 | Acute Nonparalytic Poliomyelitis; Poliomyelitis (Acute) Specified as Nonparalytic; Anterior Specified as Nonparalytic; Epidemic Specified as Nonparalytic |
| 045.20 | Acute Nonparalytic Poliomyelitis; Poliomyelitis (Acute) Specified as Nonparalytic; Anterior Specified as Nonparalytic; Epidemic Specified as Nonparalytic; Unspecified Type |
| 045.21 | Acute Nonparalytic Poliomyelitis; Poliomyelitis (Acute) Specified as Nonparalytic; Anterior Specified as Nonparalytic; Epidemic Specified as Nonparalytic; Poliovirus Type I |
| 045.22 | Acute Nonparalytic Poliomyelitis; Poliomyelitis (Acute) Specified as Nonparalytic; Anterior Specified as Nonparalytic; Epidemic Specified as Nonparalytic; Poliovirus Type II |
| 045.23 | Acute Nonparalytic Poliomyelitis; Poliomyelitis (Acute) Specified as Nonparalytic; Anterior Specified as Nonparalytic; Epidemic Specified as Nonparalytic; Poliovirus Type III |
| 045.9 | Acute Poliomyelitis, Unspecified; Infantile Paralysis Unspecified Whether Paralytic or Nonparalytic; Poliomyelitis (Acute) Unspecified Whether Paralytic or Nonparalytic; Anterior Unspecified whether Paralytic or Nonparalytic |
| 045.90 | Acute Poliomyelitis, Unspecified; Infantile Paralysis Unspecified Whether Paralytic or Nonparalytic; Poliomyelitis (Acute) Unspecified Whether Paralytic or Nonparalytic; Anterior Unspecified whether Paralytic or Nonparalytic; Unspecified Type |
| 045.91 | Acute Poliomyelitis, Unspecified; Infantile Paralysis Unspecified Whether Paralytic or Nonparalytic; Poliomyelitis (Acute) Unspecified Whether Paralytic or Nonparalytic; Anterior Unspecified whether Paralytic or Nonparalytic; Poliovirus Type I |
| 045.92 | Acute Poliomyelitis, Unspecified; Infantile Paralysis Unspecified Whether Paralytic or Nonparalytic; Poliomyelitis (Acute) Unspecified Whether Paralytic or Nonparalytic; Anterior Unspecified whether Paralytic or Nonparalytic; Poliovirus Type II |
| 045.93 | Acute Poliomyelitis, Unspecified; Infantile Paralysis Unspecified Whether Paralytic or Nonparalytic; Poliomyelitis (Acute) Unspecified Whether Paralytic or Nonparalytic; Anterior Unspecified whether Paralytic or Nonparalytic; Poliovirus Type III |
| 046 | Slow Virus Infections and prion diseases of Central Nervous System |
| 046.0 | Slow Virus Infection of Central Nervous System; Kuru |
| 046.1 | Slow Virus Infections and prion diseases of Central Nervous System; Jakob-Creutzfeldt Disease |
| 046.11 | Slow Virus Infections and prion diseases of Central Nervous System; Variant Creutzfeldt-Jakob disease; vCJD |
| 046.19 | Slow Virus Infections and prion diseases of Central Nervous System; Other and unspecified Creutzfeldt-Jakob disease; CJD; Familial Creutzfeldt-Jakob disease; Iatrogenic Creutzfeldt-Jakob disease; Sporadic Creutzfeldt-Jakob disease; Subacute spongiform en |
| 046.2 | Slow Virus Infection of Central Nervous System; Subacute Sclerosing Panencephalitis; Dawson's Inclusion Body Encephalitis; Van Bogaert's Sclerosing Leukoencephalitis |
| 046.3 | Slow Virus Infection of Central Nervous System; Progressive Multifocal Leukoencephalopathy |
| 046.7 | Other specified prion diseases of central nervous system |
| 046.71 | Other specified prion diseases of central nervous system; Gerstmann-Other specified prion diseases of central nervous system; StrÃ¤ussler-Scheinker syndrome; GSS syndrome |
| 046.72 | Other specified prion diseases of central nervous system; Fatal familial insomnia; FFI |
| 046.79 | Other and unspecified prion disease of central nervous system |
| 046.8 | Slow Virus Infection of Central Nervous System, Other Specified |
| 046.9 | Slow Virus Infection of Central Nervous System, Unspecified |
| 047 | Meningitis Due to Enterovirus |
| 047.0 | Meningitis Due to Coxsackie Virus |
| 047.1 | Meningitis Due to ECHO Virus |
| 047.8 | Viral Meningitis, Other Specified |
| 047.9 | Viral Meningitis, Unspecified; Viral Meningitis, NOS |
| 048 | Enterovirus Diseases of Central Nervous System, Other; Boston Exanthem |
| 049 | Non-Arthropod-Borne Viral Diseases of Central Nervous System, Other |
| 049.0 | Non-arthropod-borne Viral Diseases of Central Nervous System, Other; Lymphocytic Choriomeningitis; Meningitis (Serous) (Benign); Meningoencephalitis (Serous) (Benign) |
| 049.1 | Non-Arthropod-Borne Viral Diseases Of Central Nervous System, Other; Meningitis Due to Adenovirus |
| 049.8 | Non-Arthropod-Borne Viral Diseases Of Central Nervous System, Other; Encephalitis, Acute, Inclusion Body, Necrotizing; Encephalitis, Epidemic, Lethargica, Rio Bravo, von Economo's Disease |
| 049.9 | Non-Arthropod-Borne Viral Diseases of Central Nervous System, Other, Unspecified: Viral Encephalitis NOS |
| 050 | Smallpox |
| 050.0 | Smallpox; Variola Major; Hemorrhagic (pustular) Smallpox; Malignant Smallpox; Purpura Variolosa |
| 050.1 | Smallpox; Alastrim; Variola Minor |
| 050.2 | Smallpox; Modified Smallpox; Varioloid |
| 050.9 | Smallpox, Unspecified |
| 051 | Cowpox and Paravaccinia |
| 051.0 | Cowpox and vaccinia not from vaccination |
| 051.01 | Cowpox |
| 051.02 | Vaccinia not from vaccination |
| 051.1 | Pseudocowpox; Milkers' Node |
| 051.2 | Cowpox and Paravaccinia; Contagious Pustular Dermatitis; Ecthyma Contagiosum; Orf |
| 051.9 | Paravaccinia, Unspecified |
| 052 | Chickenpox |
| 052.0 | Chickenpox; Postvaricella Encephalitis |
| 052.1 | Chickenpox; Varicella (Hemorrhagic) Pneumonitis |
| 052.2 | Chickenpox; Postvaricella Myelitis; Postchickenpox Myelitis |
| 052.7 | Chickenpox with Other Specified Complications |
| 052.8 | Chickenpox with Unspecified Complication |
| 052.9 | Chickenpox; Varicella without Mention of Complication; Chickenpox NOS; Varicella NOS |
| 053 | Herpes Zoster |
| 053.0 | Herpes Zoster with Meningitis |
| 053.1 | Herpes Zoster with Nervous System Complications, Other |
| 053.10 | Herpes Zoster, with Unspecified Nervous System Complication |
| 053.11 | Geniculate Herpes Zoster; Herpetic Geniculate Ganglionitis |
| 053.12 | Herpes Zoster with Nervous System Complications, Other; Postherpetic Trigeminal Neuralgia |
| 053.13 | Herpes Zoster with Nervous System Complications, Other; Postherpetic Polyneuropathy |
| 053.14 | Herpes Zoster with Nervous System Complications, Other; Herpes Zoster Myelitis |
| 053.19 | Herpes Zoster with Nervous System Complications, Other; Other |
| 053.2 | Herpes Zoster with Ophthalmic Complications |
| 053.20 | Herpes Zoster with Ophthalmic Complications; Herpes Zoster Dermatitis of Eyelid; Herpes Zoster Ophthalmicus |
| 053.21 | Herpes Zoster with Ophthalmic Complications; Herpes Zoster Keratoconjunctivitis |
| 053.22 | Herpes Zoster with Ophthalmic Complications; Herpes Zoster Iridocyclitis |
| 053.29 | Herpes Zoster with Ophthalmic Complications, Other |
| 053.7 | Herpes Zoster with Other Specified Complications |
| 053.71 | Herpes Zoster with Other Specified Complications; Otitis Externa Due to Herpes Zoster |
| 053.79 | h Other Specified Complications; Other |
| 053.8 | Herpes Zoster with Unspecified Complication |
| 053.9 | Herpes Zoster without Mention of Complication; Herpes Zoster NOS |
| 054 | Herpes Simplex |
| 054.0 | Herpes Simplex; Eczema Herpeticum; Kaposi's Varicelliform Eruption |
| 054.1 | Herpes Simplex; Genital Herpes |
| 054.10 | Herpes Simplex; Genital Herpes, Unspecified; Herpes Progenitalis |
| 054.11 | Herpes Simplex; Genital Herpes; Herpetic Vulvovaginitis |
| 054.12 | Herpes Simplex; Genital Herpes; Herpetic Ulceration of Vulva |
| 054.13 | Herpes Simplex; Genital Herpes; Herpetic Infection of Penis |
| 054.19 | Herpes Simplex; Genital Herpes; Other |
| 054.2 | Herpes Simplex; Herpetic Gingivostomatitis |
| 054.3 | Herpes Simplex; Herpetic Meningoencephalitis; Herpes Encephalitis, Simian B Disease |
| 054.4 | Herpes Simplex with Ophthalmic Complication |
| 054.40 | Herpes Simplex with Ophthalmic Complication, Unspecified |
| 054.41 | Herpes Simplex with Ophthalmic Complication; Herpes simplex dermatitis of eyelid |
| 054.42 | Herpes Simplex with Ophthalmic Complication; Dendritic Keratitis |
| 054.43 | Herpes Simplex with Ophthalmic Complication; Herpes Simplex Disciform Keratitis |
| 054.44 | Herpes Simplex with Ophthalmic Complication; Herpes simplex iridocyclitis |
| 054.49 | Herpes Simplex with Ophthalmic Complication, Other |
| 054.5 | Herpetic Septicemia |
| 054.6 | Herpetic Whitlow; Herpetic Felon |
| 054.7 | Herpes Simplex with Other Specified Complications |
| 054.71 | Visceral Herpes Simplex |
| 054.72 | Herpes Simplex Meningitis |
| 054.73 | Herpes Simplex Otitis Externa |
| 054.74 | Herpes Simplex Myelitis |
| 054.79 | Herpes Simplex with Other Specified, Other |
| 054.8 | Herpes Simplex with Unspecified Complication |
| 054.9 | Herpes Simplex without Mention of Complication |
| 055 | Measles |
| 055.0 | Postmeasles Encephalitis |
| 055.1 | Postmeasles Pneumonia |
| 055.2 | Postmeasles Otitis Media |
| 055.7 | Measles with Other Specified Complications |
| 055.71 | Measles keratoconjunctivitis; Measles keratitis |
| 055.79 | Measles with Other Specified Complications, Other |
| 055.8 | Measles with Unspecified Complication |
| 055.9 | Rubeola; Measles without Mention of Complications |
| 056 | Rubella |
| 056.0 | Rubella with Neurological Complications |
| 056.00 | Rubella with Unspecified Neurological Complication |
| 056.01 | Encephalomyelitis Due to Rubella; Encephalitis Due to Rubella; Meningoencephalitis Due to Rubella |
| 056.09 | Rubella with Neurological Complications, Other |
| 056.7 | Rubella with Other Specified Complications |
| 056.71 | Arthritis Due to Rubella |
| 056.79 | Rubella with Other Specified Complications, Other |
| 056.8 | Rubella with Unspecified Complications |
| 056.9 | Rubella without Mention of Complication |
| 057 | Viral exanthemata, Other |
| 057.0 | Viral exanthemata, Other; Erythema infectiosum [fifth disease] |
| 057.8 | Viral Exanthemata, Other Specified; Dukes (-Filatow) Disease; Exanthema Subitum [Sixth Disease]; Fourth Disease; Parascarlatina; Pseudoscarlatina; Roseola Infantum |
| 057.9 | Viral Exanthem, Unspecified |
| 058 | Human Herpesvirus, Other |
| 058.1 | Human Herpesvirus, Other; Roseola infantum, Exanthema subitum [Sixth Disease] |
| 058.10 | Human Herpesvirus, Other; Roseola infantum, Unspecified; Exanthema subitum, Unspecified |
| 058.11 | Human Herpesvirus, Other; Roseola infantum Due to Human Herpesvirus 6; Exanthema subitum Due to Human Herpesvirus 6 |
| 058.12 | Human Herpesvirus, Other; Roseola infantum Due to Human Herpesvirus 7; Exanthema subitum Due to Human Herpesvirus 7 |
| 058.2 | Human Herpesvirus, Other, Encephalitis |
| 058.21 | Human Herpesvirus, Other; Human Herpesvirus 6 encephalitis |
| 058.29 | Human Herpesvirus, Other, Encephalitis; Human Herpesvirus 7 encephalitis |
| 058.8 | Human Herpesvirus, Other Infections |
| 058.81 | Human Herpesvirus, Other; Human Herpesvirus 6 Infection |
| 058.82 | Human Herpesvirus, Other; Human Herpesvirus 7 Infection |
| 058.89 | Human Herpesvirus, Other; Human Herpesvirus 8 Infection, Kaposi's Sarcoma-Associated Herpesvirus Infection |
| 059 | Other poxvirus infections |
| 059.0 | Other orthopoxvirus infections |
| 059.00 | Orthopoxvirus infection, unspecified |
| 059.01 | Monkeypox |
| 059.09 | Other orthopoxvirus infections |
| 059.1 | Other parapoxvirus infections |
| 059.10 | Parapoxvirus infection, unspecified |
| 059.11 | Bovine stomatitis |
| 059.12 | Sealpox |
| 059.19 | Other parapoxvirus infections |
| 059.2 | Yatapoxvirus infections |
| 059.20 | Yatapoxvirus infection, unspecified |
| 059.21 | Tanapox |
| 059.22 | Yaba monkey tumor virus |
| 059.8 | Other poxvirus infections |
| 059.9 | Poxvirus infections, unspecified |
| 060 | Yellow Fever |
| 060.0 | Yellow Fever; Sylvatic; Jungle; Sylvan |
| 060.1 | Yellow Fever, Urban |
| 060.9 | Yellow Fever, Unspecified |
| 061 | Dengue Fever; Breakbone Fever |
| 062 | Mosquito-borne Viral Encephalitis |
| 062.0 | Mosquito-borne Viral Encephalitis; Japanese Encephalitis; Japanese B Encephalitis |
| 062.1 | Mosquito-borne Viral Encephalitis; Encephalitis, Western Equine |
| 062.2 | Mosquito-borne Viral Encephalitis; Encephalitis, Eastern Equine |
| 062.3 | Mosquito-borne Viral Encephalitis; Encephalitis, St. Louis |
| 062.4 | Mosquito-borne Viral Encephalitis; Australian Encephalitis; Australian Arboencephalitis; Australian X Disease; Murray Valley Encephalitis |
| 062.5 | Mosquito-borne Viral Encephalitis; California Virus Encephalitis; California; La Crosse; Tahyna Fever |
| 062.8 | Mosquito-borne Viral Encephalitis, Other; Encephalitis by Ilheus Virus |
| 062.9 | Mosquito Borne Viral Encephalitis, Unspecified |
| 063 | Tick-borne viral encephalitis |
| 063.0 | Tick-borne Viral Encephalitis; Russian Spring-summer [Taiga] Encephalitis |
| 063.1 | Tick-borne Viral Encephalitis; Louping Ill |
| 063.2 | Tick-borne Viral Encephalitis; Central European Encephalitis |
| 063.8 | Tick-borne Viral Encephalitis, Other Specific; Langat Encephalitis; Powassan Encephalitis |
| 063.9 | Tick-borne Viral Encephalitis, Unspecified |
| 064 | Encephalitis, Viral, Transmitted by Other and Unspecified Arthropods; Arthropod-borne Viral Encephalitis, Vector Unknown; Negishi Virus Encephalitis |
| 065 | Arthropod-borne Hemorrhagic Fever |
| 065.0 | Arthropod-borne Hemorrhagic Fever; Crimean Hemorrhagic Fever [CHF Congo Virus]; Central Asian Hemorrhagic Fever |
| 065.1 | Arthropod-borne Hemorrhagic Fever; Omsk Hemorrhagic Fever |
| 065.2 | Arthropod-borne Hemorrhagic Fever; Kyasanur Forest Disease |
| 065.3 | Arthropod-borne Hemorrhagic Fever; Other Tick-borne Hemorrhagic Fever |
| 065.4 | Mosquito-borne Hemorrhagic Fever; Chikungunya Hemorrhagic Fever; Dengue Hemorrhagic Fever |
| 065.8 | Arthropod-borne Hemorrhagic Fever, Other specified; Mite-borne Hemorrhagic Fever |
| 065.9 | Arthropod-borne Hemorrhagic Fever, Unspecified |
| 066 | Arthropod-borne Viral Diseases, Other |
| 066.0 | Arthropod-borne Viral Diseases, Other; Phlebotomus Fever; Changuinola Fever; Sandfly Fever |
| 066.1 | Arthropod-borne Viral Diseases, Other; Tick-Borne Fever; Nairobi Sheep Disease; Tick Fever, American Mountain, Colorado, Kemerovo, Quaranfil |
| 066.2 | Arthropod-borne Viral Diseases, Other; Venezuelan Equine Fever; Venezuelan Equine Encephalitis |
| 066.3 | Mosquito-borne Fever, Other; Bunyamwera; Bwamba; Chikungunya; Guama; Mayaro; Mucambo; O'Nyong-Nyong; Oropouche; Pixuna; Rift Valley; Ross River; Wesselsbron; Zika |
| 066.4 | Arthropod-borne Viral Diseases, Other; West Nile fever |
| 066.40 | Arthropod-borne Viral Diseases, Other; West Nile Fever, Unspecified; West Nile Fever NOS; West Nile Fever without Complications; West Nile Virus NOS |
| 066.41 | Arthropod-borne Viral Diseases, Other; West Nile Fever with Encephalitis; West Nile Encephalitis; West Nile Encephalomyelitis |
| 066.42 | Arthropod-Borne Viral Diseases, Other; West Nile Fever with other Neurologic Manifestation |
| 066.49 | Arthropod-Borne Viral Diseases, Other; West Nile fever with Other Complications |
| 066.8 | Arthropod-borne Viral Diseases, Other Specified; Chandipura Fever; Piry Fever |
| 066.9 | Arthropod-borne Viral Disease, Unspecified; Arbovirus Infection NOS |
| 070 | Viral Hepatitis |
| 070.0 | Hepatitis, Viral Type A with Hepatic Coma |
| 070.1 | Hepatitis, Viral Type A without Mention of Hepatic Coma |
| 070.2 | Hepatitis, Viral Type B with Hepatic Coma |
| 070.20 | Hepatitis, Viral Type B with Hepatic Coma, Acute or Unspecified, without Mention of Hepatitis delta |
| 070.21 | Hepatitis, Viral Type B with Hepatic Coma, Acute or Unspecified, with Hepatitis delta |
| 070.22 | Hepatitis, Viral Type B with Hepatic Coma, Chronic, without Mention of Hepatitis delta |
| 070.23 | Hepatitis, Viral Type B with Hepatic Coma, Chronic, with Hepatitis delta |
| 070.3 | Hepatitis, Viral Type B without Mention of Hepatic Coma |
| 070.30 | Hepatitis, Viral Type B without Mention of Hepatic Coma, Acute or Unspecified, without Mention of Hepatitis Delta |
| 070.31 | Hepatitis, Viral Type B without Mention of Hepatic Coma, Acute or Unspecified, with Hepatitis Delta |
| 070.32 | Hepatitis, Viral Type B without Mention of Hepatic Coma, Chronic, without Mention of Hepatitis Delta |
| 070.33 | Hepatitis, Viral Type B without Mention of Hepatic Coma, Chronic, with Hepatitis Delta |
| 070.4 | Hepatitis, Other Specified Viral with Hepatic Coma |
| 070.41 | Hepatitis, Viral Type C with Hepatic Coma, Other Specified |
| 070.42 | Hepatitis Delta without Mention of Active Hepatitis B Disease with Hepatic Coma; Hepatitis Delta with Hepatitis B Carrier State |
| 070.43 | Hepatitis E with Hepatic Coma |
| 070.44 | Chronic Hepatitis C with Hepatic Coma |
| 070.49 | Viral Hepatitis with Hepatic Coma, Other Specified |
| 070.5 | Hepatitis, Viral Type C without Mention of Hepatic Coma, Other Specified |
| 070.51 | Hepatitis, Viral Type C |
| 070.52 | Hepatitis Delta without Mention of Active Hepatitis B Disease or Hepatic Coma |
| 070.53 | Hepatitis E without Mention of Hepatic Coma |
| 070.54 | Chronic Hepatitis C without Mention of Hepatic Coma |
| 070.59 | Viral Hepatitis without Mention of Hepatic Coma, Other Specified |
| 070.6 | Viral Hepatitis with Hepatic Coma, Unspecified |
| 070.7 | Viral Hepatitis C, Unspecified |
| 070.70 | Viral Hepatitis C without Hepatic Coma, unspecified; Unspecified Viral Hepatitis C NOS |
| 070.71 | Viral Hepatitis C with Hepatic Coma, Unspecified |
| 070.9 | Hepatitis, Viral without Mention of Hepatic Coma, Unspecified; Viral hepatitis NOS |
| 071 | Rabies, Hydrophobia, Lyssa |
| 072 | Mumps |
| 072.0 | Mumps Orchitis |
| 072.1 | Mumps Meningitis |
| 072.2 | Mumps Encephalitis; Mumps Meningoencephalitis |
| 072.3 | Mumps Pancreatitis |
| 072.7 | Mumps with Other Specified Complications |
| 072.71 | Mumps with Other Specified Complications; Mumps hepatitis |
| 072.72 | Mumps with Other Specified Complications; Mumps Polyneuropathy |
| 072.79 | Mumps with Other Specified Complications, Other |
| 072.8 | Mumps with Unspecified Complication |
| 072.9 | Mumps without Mention of Complication; Epidemic Parotitis; Infectious Parotitis |
| 073 | Ornithosis |
| 073.0 | Ornithosis with Pneumonia; Lobular Pneumonitis Due to Ornithosis |
| 073.7 | Ornithosis with Other Specified Complications |
| 073.8 | Ornithosis with Unspecified Complication |
| 073.9 | Ornithosis, Unspecified |
| 074 | Specific Diseases Due to Coxsackie Virus |
| 074.0 | Specific Diseases Due to Coxsackie Virus; Herpangina; Vesicular Pharyngitis |
| 074.1 | Specific Diseases Due to Coxsackie Virus; Epidemic Pleurodynia; Bornholm Disease; Devil's Grip; Epidemic: Myalgia; Myositis |
| 074.2 | Coxsackie Carditis |
| 074.20 | Coxsackie Carditis, Unspecified |
| 074.21 | Coxsackie Pericarditis |
| 074.22 | Coxsackie Endocarditis |
| 074.23 | Coxsackie Myocarditis; Aseptic Myocarditis of Newborn |
| 074.3 | Specific Diseases Due to Coxsackie Virus; Hand, Foot, and Mouth Disease; Vesicular Stomatitis and Exanthem |
| 074.8 | Diseases Due to Coxsackie Virus, Other Specified; Acute lymphonodular pharyngitis |
| 075 | Infectious Mononucleosis; Glandular Fever, Monocytic Angina, Pfeiffer's Disease |
| 076 | Trachoma |
| 076.0 | Trachoma, Initial Stage; Trachoma Dubium |
| 076.1 | Trachoma, Active Stage; Granular Conjunctivitis; Trachomatous: Follicular Conjunctivitis; Pannus |
| 076.9 | Trachoma, Unspecified; Trachoma NOS |
| 077 | Diseases of Conjunctiva Due to Viruses and Chlamydiae, Other |
| 077.0 | Diseases of Conjunctiva Due to Viruses and Chlamydiae, Other; Inclusion Conjunctivitis; Paratrachoma; Swimming Pool Conjunctivitis |
| 077.1 | Diseases of Conjunctiva Due to Viruses and Chlamydiae, Other; Epidemic Keratoconjunctivitis; Shipyard Eye |
| 077.2 | Diseases of Conjunctiva Due to Viruses and Chlamydiae, Other; Pharyngoconjunctival Fever; Viral Pharyngoconjunctivitis |
| 077.3 | Diseases of Conjunctiva Due to Viruses and Chlamydiae, Other; Other Adenoviral Conjunctivitis; Acute Adenoviral Follicular Conjunctivitis |
| 077.4 | Diseases of Conjunctiva Due to Viruses and Chlamydiae, Other; Epidemic Hemorrhagic Conjunctivitis; Disease; Conjunctivitis Due to Enterovirus Type 70; Hemorrhagic Conjunctivitis (Acute) (Epidemic) |
| 077.8 | Diseases of Conjunctiva Due to Viruses and Chlamydiae, Other; Other Viral Conjunctivitis; Newcastle Conjunctivitis |
| 077.9 | Diseases of Conjunctiva Due to Viruses and Chlamydiae, Unspecified |
| 077.98 | Diseases of Conjunctiva Due to Viruses and Chlamydiae, Unspecified; Due to Chlamydiae |
| 077.99 | Diseases of Conjunctiva Due to Viruses and Chlamydiae, Unspecified; Due to Viruses; Viral Conjunctivitis NOS |
| 078 | Diseases Due to Viruses and Chlamydiae, Other |
| 078.0 | Diseases Due to Viruses and Chlamydiae, Other; Molluscum Contagiosum |
| 078.1 | Diseases Due to Viruses and Chlamydiae, Other; Viral Warts |
| 078.10 | Diseases Due to Viruses and Chlamydiae, Other; Viral Warts, Unspecified; Verruca |
| 078.11 | Diseases Due to Viruses and Chlamydiae, Other; Condyloma Acuminatum; Condyloma NOS; Genital warts NOS |
| 078.12 | Diseases Due to Viruses and Chlamydiae, Other; Plantar wart; Verruca plataris |
| 078.19 | Diseases Due to Viruses and Chlamydiae, Other; Viral warts, Other Specified; Common Wart; Flat wart; Verruca plana |
| 078.2 | Diseases Due to Viruses and Chlamydiae, Other; Sweating Fever; Miliary Fever; Sweating Disease |
| 078.3 | Diseases Due to Viruses and Chlamydiae, Other; Cat Scratch Disease; Benign Lymphoreticulosis (of Inoculation); Cat-Scratch Fever |
| 078.4 | Diseases Due to Viruses and Chlamydiae, Other; Foot and Mouth Disease; Aphthous Fever; Epizootic: Aphthae, Stomatitis |
| 078.5 | Diseases Due to Viruses and Chlamydiae, Other; Cytomegaloviral Disease; Cytomegalic Inclusion Disease; Salivary Gland Virus Disease |
| 078.6 | Diseases Due to Viruses and Chlamydiae, Other; Hemorrhagic nephrosonephritis; Hemorrhagic Fever: Epidemic, Korean, Russian with Renal Syndrome |
| 078.7 | Diseases Due to Viruses and Chlamydiae, Other; Arenaviral Fever; Hemorrhagic Fever: Argentine, Bolivian, Junin Virus, Machupo Virus |
| 078.8 | Diseases Due to Viruses and Chlamydiae; Other Specified |
| 078.81 | Diseases Due to Viruses and Chlamydiae; Other Specified; Epidemic Vertigo |
| 078.82 | Diseases Due to Viruses and Chlamydiae; Other Specified; Epidemic Vomiting Syndrome; Winter Vomiting Disease |
| 078.88 | Diseases Due to Viruses and Chlamydiae, Other Specified; Other Specified Diseases Due to Chlamydiae |
| 078.89 | Diseases Due to Viruses and Chlamydiae, Other Specified; Other Specified Diseases Due to Viruses, Epidemic Cervical Myalgia, Marburg Disease |
| 079 | Viral and Chlamydial Infection in Conditions Classified Elsewhere and of Unspecified Site |
| 079.0 | Viral and Chlamydial Infection in Conditions Classified Elsewhere and of Unspecified Site; Adenovirus |
| 079.1 | Viral and Chlamydial Infection in Conditions Classified Elsewhere and of Unspecified Site; ECHO Virus |
| 079.2 | Viral and Chlamydial Infection in Conditions Classified Elsewhere and of Unspecified Site; Coxsackie Virus |
| 079.3 | Viral and Chlamydial Infection in Conditions Classified Elsewhere and of Unspecified Site; Rhinovirus |
| 079.4 | Viral and Chlamydial Infection in Conditions Classified Elsewhere and of Unspecified Site; Human Papillomavirus |
| 079.5 | Viral and Chlamydial Infection in Conditions Classified Elsewhere and of Unspecified Site; Retrovirus |
| 079.50 | Viral and Chlamydial Infection in Conditions Classified Elsewhere and of Unspecified Site; Retrovirus, Unspecified |
| 079.51 | Viral and Chlamydial Infection in Conditions Classified Elsewhere and of Unspecified Site; Retrovirus; Human T-cell Lymphotrophic Virus, Type I [HTLV-I] |
| 079.52 | Viral and Chlamydial Infection in Conditions Classified Elsewhere and of Unspecified Site; Retrovirus; Human T-cell Lymphotrophic Virus, Type II [HTLV-II] |
| 079.53 | Viral and Chlamydial Infection in Conditions Classified Elsewhere and of Unspecified Site; Retrovirus; Human Immunodeficiency Virus, Type 2 [HIV-2] |
| 079.59 | Viral and Chlamydial Infection in Conditions Classified Elsewhere and of Unspecified Site; Retrovirus; Other Specified Retrovirus |
| 079.6 | Viral and Chlamydial Infection in Conditions Classified Elsewhere and of Unspecified Site; Respiratory Syncytial Virus (RSV) |
| 079.8 | Viral and Chlamydial Infection in Conditions Classified Elsewhere and of Unspecified Site; Specified Viral and Chlamydial Infections, Other |
| 079.81 | Viral and Chlamydial Infection in Conditions Classified Elsewhere and of Unspecified Site; Specified Viral and Chlamydial Infections, Other; Hantavirus |
| 079.82 | Viral and Chlamydial Infection in Conditions Classified Elsewhere and of Unspecified Site; Specified Viral and Chlamydial Infections, Other; SARS-associated Coronavirus |
| 079.83 | Viral and Chlamydial Infection in Conditions Classified Elsewhere and of Unspecified Site; Specified Viral and Chlamydial Infections, Other; Parvovirus B19, Human Parvovirus, Parvovirus NOS |
| 079.88 | Viral and Chlamydial Infection in Conditions Classified Elsewhere and of Unspecified Site; Chlamydial Infection, Other Specified |
| 079.89 | Viral and Chlamydial Infection in Conditions Classified Elsewhere and of Unspecified Site; Viral Infection, Other Specified |
| 079.9 | Viral and Chlamydial Infections, Unspecified |
| 079.98 | Chlamydial Infection, Unspecified; Chlamydial Infection NOS |
| 079.99 | Viral Infection, Unspecified; Viral Infection NOS |
| 080 | Typhus, Louse-borne; Typhus Fever: Classical, Epidemic, Exanthematic NOS, Louse-borne |
| 081 | Typhus, Other |
| 081.0 | Typhus, Other; Murine Typhus, Typhus: Endemic, Flea-borne |
| 081.1 | Typhus, Other; Brill's Disease; Brill-Zinsser Disease; Recrudescent Typhus (Fever) |
| 081.2 | Typhus, Scrub; Japanese River Fever, Kedani Fever, Mite-borne Typhus, Tsutsugamushi |
| 081.9 | Typhus Fever, Unspecified; Typhus NOS |
| 082 | Tick-borne Rickettsioses |
| 082.0 | Tick-borne Rickettsioses; Spotted Fevers, Rocky Mountain Spotted Fever, Sao Paulo Fever |
| 082.1 | Tick-borne Rickettsioses; Boutonneuse Fever; African Tick Typhus; India Tick Typhus; Kenya Tick Typhus; Marseilles Fever; Mediterranean Tick Fever |
| 082.2 | Tick-borne Rickettsioses; North Asian Tick Fever; Siberian Tick Typhus |
| 082.3 | Tick-borne Rickettsioses; Queensland Tick Typhus |
| 082.4 | Tick-borne Rickettsioses; Ehrlichiosis |
| 082.40 | Tick-borne Rickettsioses; Ehrlichiosis, Unspecified |
| 082.41 | Tick-borne Rickettsioses; Ehrlichiosis chaffeensis [E. chaffeensis] |
| 082.49 | Tick-borne Rickettsioses; Other Ehrlichiosis |
| 082.8 | Tick-borne Rickettsioses, Other Specified; Lone Star Fever |
| 082.9 | Tick-borne Rickettsiosis, Unspecified; Tick Borne Typhus NOS |
| 083 | Rickettsiosis, Other |
| 083.0 | Rickettsiosis, Other; Q fever |
| 083.1 | Rickettsiosis, Other; Trench Fever; Quintan Fever; Wolhynian Fever |
| 083.2 | Rickettsiosis, Other; Rickettsialpox; Vesicular Rickettsiosis |
| 083.8 | Rickettsioses, Other Specified |
| 083.9 | Rickettsiosis, Unspecified |
| 084 | Malaria |
| 084.0 | Malaria, Falciparum; Malaria by Plasmodium Falciparum, Subtertian |
| 084.1 | Malaria, Vivax (Benign Tertian); Malaria by Plasmodium Vivax |
| 084.2 | Malaria, Quartan; Malaria by Malariae; Malariae Malaria |
| 084.3 | Malaria, Ovale; Malaria by Plasmodium Ovale |
| 084.4 | Malaria, Other; Monkey Malaria |
| 084.5 | Malaria, Mixed; Malaria by More than One Parasite |
| 084.6 | Malaria, Unspecified; Malaria NOS |
| 084.7 | Malaria, Induced; Therapeutically Induced Malaria |
| 084.8 | Malaria; Blackwater Fever; Hemoglobinuric Fever (Bilious), Hemoglobinuric Malaria, Malarial Hemoglobinuria |
| 084.9 | Malaria, Other Pernicious Complications of Malaria; Algid Malaria; Cerebral Malaria |
| 085 | Leishmaniasis |
| 085.0 | Leishmaniasis; Visceral; Dumdum Fever; Infection by Leishmania: Donovani, Infantum: Leishmaniasis: Dermal, Post-Kala-Azar; Mediterranean; Visceral (Indian) |
| 085.1 | Leishmaniasis; Cutaneous, Urban; Aleppo Boil, Baghdad Boil, Delhi Boil, Infection by Leishmania Tropica (Minor); Leishmaniasis, Cutaneous: Dry Form, Late, Recurrent, Ulcerating; Oriental Sore |
| 085.2 | Leishmaniasis; Cutaneous, Asian desert; Infection by Leishmania Tropica Major; Leishmaniasis, Cutaneous: Acute Necrotizing, Rural, Wet Form, Zoonotic Form |
| 085.3 | Leishmaniasis; Cutaneous, Ethiopian; Infection by Leishmania ethiopica; Leishmaniasis, Cutaneous: Diffuse, Lepromatous |
| 085.4 | Leishmaniasis; Cutaneous, American; Chiclero Ulcer; Infection by Leishmania Mexicana; Leishmaniasis Tegumentaria Diffusa |
| 085.5 | Leishmaniasis; Mucocutaneous (American); Espundia; Infection by Leishmania braziliensis; Uta |
| 085.9 | Leishmaniasis, Unspecified |
| 086 | Trypanosomiasis |
| 086.0 | Trypanosomiasis; Chagas' Disease with Heart Involvement; American Trypanosomiasis with Heart Involvement; Infection by Trypanosoma cruzi with Heart Involvement |
| 086.1 | Trypanosomiasis; Chagas' Disease with Other Organ Involvement; American trypanosomiasis with Involvement of Organ Other than Heart; Infection by Trypanosoma cruzi with Involvement of Organ Other than Heart |
| 086.2 | Trypanosomiasis; Chagas' Disease without Mention of Organ Involvement; American Trypanosomiasis; Infection by Trypanosoma cruzi |
| 086.3 | Trypanosomiasis, Gambian; Gambian Sleeping Sickness; Infection by Trypanosoma Gambiense |
| 086.4 | Trypanosomiasis, Rhodesian; Infection by trypanosoma Rhodesiense; Rhodesian Sleeping Sickness |
| 086.5 | Trypanosomiasis, African, Unspecified; Sleeping Sickness NOS |
| 086.9 | Trypanosomiasis, Unspecified |
| 087 | Relapsing Fever |
| 087.0 | Relapsing Fever, Louse-Borne |
| 087.1 | Relapsing Fever, Tick-borne |
| 087.9 | Relapsing Fever, Unspecified |
| 088 | Arthropod-borne Diseases, Other |
| 088.0 | Arthropod-borne Diseases, Other Bartonellosis; CarriÃ³n's Disease; Oroya Fever; Verruga Peruana |
| 088.8 | Arthropod-borne Diseases, Other Specified |
| 088.81 | Arthropod-borne Diseases, Other Specified; Lyme Disease |
| 088.82 | Arthropod-borne Diseases, Other Specified; Babesiosis; Babesiasis |
| 088.89 | Arthropod-Borne Diseases, Other Specified; Other |
| 088.9 | Arthropod-borne Disease, Unspecified |
| 090 | Syphilis, Congenital |
| 090.0 | Early Congenital Syphilis, Symptomatic; Choroiditis; Coryza (Chronic); Hepatomegaly; Mucous Patches; Periostitis; Splenomegaly; Epiphysitis; Osteochondritis; Pemphigus; Any Congenital Syphilitic Condition Specified as Early or Manifest Less than Two Years after Birth |
| 090.1 | Early Congenital Syphilis, Latent; Congenital Syphilis without Clinical Manifestations, with Positive Serological Reaction and Negative Spinal Fluid Test, Less than Two Years after Birth |
| 090.2 | Early Congenital Syphilis, Unspecified; Congenital Syphilis NOS, Less than Two Years after Birth |
| 090.3 | Syphilis, Congenital; Syphilitic Interstitial Keratitis; Parenchymatous; Punctata Profunda |
| 090.4 | Juvenile Neurosyphilis |
| 090.40 | Juvenile Neurosyphilis, Unspecified; Congenital Neurosyphilis; Dementia Paralytica Juvenilis; Juvenile: General Paresis, Tabes, Taboparesis |
| 090.41 | Juvenile Neurosyphilis; Congenital Syphilitic Encephalitis |
| 090.42 | Juvenile Neurosyphilis; Congenital Syphilitic Meningitis |
| 090.49 | Juvenile Neurosyphilis, Other |
| 090.5 | Late Congenital Syphilis, Other; Symptomatic; Gumma Due to Congenital Syphilis; Hutchinson's Teeth; Syphilitic Saddle Nose; Any Congenital Syphilitic Condition Specified as Late or Manifest Two Years or More after Birth |
| 090.6 | Late Congenital Syphilis, Latent; Congenital Syphilis without Clinical Manifestations, with Positive Serological Reaction and Negative Spinal Fluid Test, Two Years or More after Birth |
| 090.7 | Late Congenital Syphilis, Unspecified; Congenital Syphilis NOS< Two Years or More after Birth |
| 090.9 | Congenital Syphilis, Unspecified |
| 091 | Early Syphilis, Symptomatic |
| 091.0 | Early Syphilis, Symptomatic; Syphilis, Genital (Primary); Genital Chancre |
| 091.1 | Early Syphilis, Symptomatic; Syphilis, Primary Anal |
| 091.2 | Early Syphilis, Symptomatic; Syphilis, Primary, Other; Primary Syphilis of Breast, Fingers, Lip, or Tonsils |
| 091.3 | Early Syphilis, Symptomatic; Syphilis of Skin or Mucous Membrane, Secondary; Condyloma Latum; Secondary Syphilis of Anus, Mouth, Pharynx, Skin, Tonsils, Vulva |
| 091.4 | Adenopathy Due to Secondary Syphilis; Syphilitic Adenopathy, Secondary; Syphilitic Lymphadenitis, Secondary |
| 091.5 | Uveitis Due to Secondary Syphilis |
| 091.50 | Syphilitic Uveitis, Unspecified |
| 091.51 | Uveitis Due to Secondary Syphilis; Syphilitic Chorioretinitis, Secondary |
| 091.52 | Uveitis Due to Secondary Syphilis; Syphilitic Iridocyclitis, Secondary |
| 091.6 | Syphilis, Secondary of Viscera and Bone |
| 091.61 | Secondary Syphilitic Periostitis |
| 091.62 | Secondary Syphilitic Hepatitis; Secondary Syphilis of Liver |
| 091.69 | Syphilis, Secondary of Other Viscera |
| 091.7 | Syphilis, Secondary, Relapse; Treated, Untreated |
| 091.8 | Syphilis, Secondary, Other Forms |
| 091.81 | Acute Syphilitic Meningitis, Secondary |
| 091.82 | Syphilitic Alopecia |
| 091.89 | Syphilis, Secondary, Other Forms, Other |
| 091.9 | Syphilis, Secondary, Unspecified |
| 092 | Syphilis, Early, Latent |
| 092.0 | Syphilis, Early, Latent, Serological Relapse after Treatment |
| 092.9 | Syphilis, Early, Latent, Unspecified |
| 093 | Cardiovascular Syphilis |
| 093.0 | Cardiovascular Syphilis; Aneurysm of Aorta, Specified As Syphilitic; Dilatation of Aorta, Specified as Syphilitic |
| 093.1 | Cardiovascular Syphilis; Syphilitic Aortitis |
| 093.2 | Cardiovascular Syphilis; Syphilitic Endocarditis |
| 093.20 | Cardiovascular Syphilis; Syphilitic Endocarditis of Valve, Unspecified; Syphilitic Ostial Coronary Disease |
| 093.21 | Cardiovascular Syphilis; Syphilitic Endocarditis; Mitral Valve |
| 093.22 | Cardiovascular Syphilis; Syphilitic Endocarditis; Aortic Valve; Syphilitic Aortic Incompetence or Stenosis |
| 093.23 | Cardiovascular Syphilis; Syphilitic Endocarditis; Tricuspid Valve |
| 093.24 | Cardiovascular Syphilis; Syphilitic Endocarditis; Pulmonary Valve |
| 093.8 | Cardiovascular Syphilis, Other Specified |
| 093.81 | Syphilitic Pericarditis |
| 093.82 | Syphilitic Myocarditis |
| 093.89 | Syphilis, Cardiovascular, Other Specified, Other |
| 093.9 | Syphilis, Cardiovascular, Unspecified |
| 094 | Neurosyphilis |
| 094.0 | Neurosyphilis, Tabes Dorsalis; Locomotor Ataxia, Progressive; Syphilitic Posterior Spinal Sclerosis |
| 094.1 | Neurosyphilis; General Paresis; Paretic Neurosyphilis, Dementia Paralytica, General Paralysis (of the Insane) (Progressive), Taboparesis |
| 094.2 | Neurosyphilis; Meningitis, Syphilitic; Meningovascular Syphilis |
| 094.3 | Neurosyphilis; Asymptomatic |
| 094.8 | Neurosyphilis, Other Specified |
| 094.81 | Neurosyphilis, Other Specified; Syphilitic Encephalitis |
| 094.82 | Neurosyphilis, Other Specified; Syphilitic Parkinsonism |
| 094.83 | Neurosyphilis, Other Specified; Syphilitic Disseminated Retinochoroiditis |
| 094.84 | Neurosyphilis, Other Specified; Syphilitic Optic Atrophy |
| 094.85 | Neurosyphilis, Other Specified; Syphilitic Retrobulbar Neuritis |
| 094.86 | Neurosyphilis, Other Specified; Syphilitic Acoustic Neuritis |
| 094.87 | Neurosyphilis, Other Specified; Syphilitic Ruptured Cerebral Aneurysm |
| 094.89 | Neurosyphilis, Other Specified; Other |
| 094.9 | Neurosyphilis, Unspecified; Gumma of Central Nervous System NOS; Syphilis of Central Nervous System NOS; Syphiloma of Central Nervous System NOS |
| 095 | Late Syphilis, with Symptoms, Other Forms |
| 095.0 | Syphilitic Episcleritis |
| 095.1 | Syphilis of Lung |
| 095.2 | Syphilitic Peritonitis |
| 095.3 | Syphilis of Liver |
| 095.4 | Syphilis of Kidney |
| 095.5 | Syphilis of Bone |
| 095.6 | Syphilis of muscle; Syphilitic myositis |
| 095.7 | Syphilis of Synovium, Tendon, and Bursa; Syphilitic: Bursitis, Synovitis |
| 095.8 | Syphilis, Other Specified Forms of Late Symptomatic |
| 095.9 | Syphilis, Late Symptomatic, Unspecified |
| 096 | Syphilis, Late, Latent; Syphilis without Clinical Manifestations, with Positive Serological Reaction and Negative Spinal Fluid Test, Two Years or More after Infection |
| 097 | Syphilis, Other and Unspecified |
| 097.0 | Late Syphilis, Unspecified |
| 097.1 | Latent Syphilis, Unspecified; Positive Serological Reaction for Syphilis |
| 097.9 | Syphilis, Unspecified; Syphilis, Acquired, NOS |
| 098 | Gonococcal Infections |
| 098.0 | Gonococcal Infection, Acute, Lower Genitourinary Tract; Gonococcal: Bartholinitis, Acute; Urethritis, Acute; Vulvovaginitis, Acute: Gonorrhea (Acute), NOS, Genitourinary NOS |
| 098.1 | Gonococcal Infection; Acute, Upper Genitourinary Tract |
| 098.10 | Gonococcal Infection; Acute, Upper Genitourinary Tract; Site Unspecified |
| 098.11 | Gonococcal Infection; Acute, Upper Genitourinary Tract; Gonorrhea (Acute) of Bladder |
| 098.12 | Gonococcal Infection; Acute, Upper Genitourinary Tract; Gonococcal Prostatitis (Acute) |
| 098.13 | Gonococcal Infection; Acute, Upper Genitourinary Tract; Gonococcal Epididymo-orchitis, Acute |
| 098.14 | Gonococcal Infection; Acute, Upper Genitourinary Tract; Gonococcal Seminal Vesiculitis (Acute); Gonorrhea (Acute) of Seminal Vesicle |
| 098.15 | Gonococcal Infection; Acute, Upper Genitourinary Tract; Gonococcal Cervicitis (Acute); Gonorrhea (Acute) of Cervix |
| 098.16 | Gonococcal Infection; Acute, Upper Genitourinary Tract; Gonococcal Endometritis (Acute); Gonorrhea (Acute) of Uterus |
| 098.17 | Gonococcal Infection; Acute, Upper Genitourinary Tract; Gonococcal Salpingitis, Specified as Acute |
| 098.19 | Gonococcal Infection, Acute, Upper Genitourinary Tract, Other |
| 098.2 | Gonococcal Infection; Chronic, Lower Genitourinary Tract |
| 098.3 | Gonococcal Infection; Chronic, Upper Genitourinary Tract |
| 098.30 | Chronic Gonococcal Infection of Upper Genitourinary Tract, Site Unspecified |
| 098.31 | Gonococcal Cystitis, Chronic; Gonorrhea of Bladder, Chronic |
| 098.32 | Gonococcal prostatitis, chronic |
| 098.33 | Gonococcal Epididymo-orchitis, Chronic; Gonococcal Orchitis, Chronic |
| 098.34 | Gonococcal Seminal Vesiculitis, Chronic; Gonorrhea of Seminal Vesicle, Chronic |
| 098.35 | Gonococcal Cervicitis, Chronic; Gonorrhea of Cervix, Chronic |
| 098.36 | Gonococcal Endometritis, Chronic |
| 098.37 | Gonococcal Salpingitis (Chronic) |
| 098.39 | Gonococcal Infection, Chronic, Upper Genitourinary Tract, Other |
| 098.4 | Gonococcal Infection of Eye |
| 098.40 | Gonococcal Conjunctivitis, Neonatorum; Gonococcal Ophthalmia, Neonatorum |
| 098.41 | Gonococcal Iridocyclitis |
| 098.42 | Gonococcal Endophthalmia |
| 098.43 | Gonococcal Keratitis |
| 098.49 | Gonococcal Infection of Eye, Other |
| 098.5 | Gonococcal Infection of Joint |
| 098.50 | Gonococcal Arthritis; Gonococcal Infection of Joint NOS |
| 098.51 | Gonococcal Synovitis and Tenosynovitis |
| 098.52 | Gonococcal Bursitis |
| 098.53 | Gonococcal Spondylitis |
| 098.59 | Gonococcal Infection of Joint, Other; Gonococcal rheumatism |
| 098.6 | Gonococcal Infection of Pharynx |
| 098.7 | Gonococcal Infection of Anus and Rectum; Gonococcal Proctitis |
| 098.8 | Gonococcal Infection of Other Specified Sites |
| 098.81 | Gonococcal Keratosis, Blennorrhagica |
| 098.82 | Gonococcal Meningitis |
| 098.83 | Gonococcal Pericarditis |
| 098.84 | Gonococcal Endocarditis |
| 098.85 | Gonococcal Infection of Other Specified Sites, Other Gonococcal Heart Disease |
| 098.86 | Gonococcal Peritonitis |
| 098.89 | Gonococcal Infection of Other Specified Sites, Other; Gonococcemia |
| 099 | Venereal Diseases, Other |
| 099.0 | Venereal Diseases, Other; Chancroid; Bubo (Inguinal), Chancroidal, Due to Hemophilus ducreyi; Chancre, Ducrey's Simple Soft, Ulcus molle (Cutis) (Skin) |
| 099.1 | Venereal Diseases, Other; Lymphogranuloma venereum; Climatic or Tropical Bubo; (Durand-) Nicolas-Favre Disease; Esthiomene; Lymphogranuloma Inguinale |
| 099.2 | Venereal Diseases, Other; Granuloma Inguinale; Donovanosis; Granuloma Pudendi (Ulcerating); Granuloma Venereum; Pudendal Ulcer |
| 099.3 | Venereal Diseases, Other; Reiter's Syndrome (Disease) |
| 099.4 | Venereal Diseases, Other; Urethritis, Other Nongonococcal |
| 099.40 | Venereal Diseases, Other; Urethritis, Other Nongonococcal, Unspecified |
| 099.41 | Venereal Diseases, Other; Nongonococcal Urethritis Due to Chlamydia Trachomatis |
| 099.49 | Venereal Diseases, Other; Nongonococcal Urethritis Due to Other Specified Organism |
| 099.5 | Venereal Diseases Due to Chlamydia Trachomatis, Other |
| 099.50 | Venereal Diseases Due to Chlamydia Trachomatis, Other; Unspecified site |
| 099.51 | Venereal Diseases Due to Chlamydia Trachomatis, Other; Pharynx |
| 099.52 | Venereal Diseases Due to Chlamydia Trachomatis, Other; Anus and rectum |
| 099.53 | Venereal Diseases Due to Chlamydia Trachomatis, Other; Lower Genitourinary Sites |
| 099.54 | Venereal Diseases Due to Chlamydia Trachomatis, Other; Other Genitourinary Sites |
| 099.55 | Venereal Diseases Due to Chlamydia Trachomatis, Other; Unspecified Genitourinary Site |
| 099.56 | Venereal Diseases Due to Chlamydia Trachomatis, Other; Peritoneum; Perihepatitis |
| 099.59 | Venereal Diseases Due to Chlamydia Trachomatis, Other; Other specified site |
| 099.8 | Venereal Disease, Other Specified |
| 099.9 | Venereal Disease, Unspecified |
| 100 | Leptospirosis |
| 100.0 | Leptospirosis Icterohemorrhagica; Leptospiral or Spirochetal Jaundice (Hemorrhagic); Weil's Disease |
| 100.8 | Leptospiral Infections, Other Specified |
| 100.81 | Leptospiral Infections, Other Specified; Leptospiral Meningitis (Aseptic) |
| 100.89 | Leptospiral Infections, Other Specified; Other; Fever, Fort Bragg, Pretibial, Swamp; Infection by Leptospira, australis, bataviae, pyrogenes |
| 100.9 | Leptospirosis, Unspecified |
| 101 | Vincent's Angina; Acute Necrotizing Ulcerative, Gingivitis, Stomatitis; Fusospirochetal Pharyngitis; Spirochetal Stomatitis; Trench Mouth; Vincent's Gingivitis; Vincent's Infection [Any Site] |
| 102 | Yaws |
| 102.0 | Yaws; Initial Lesions; Chancre of Yaws; Frambesia, Initial or Primary; Initial Frambesial Ulcer; Mother Yaw |
| 102.1 | Yaws; Multiple Papillomata and Wet Crab Yaws; Butter Yaws; Frambesioma; Pianoma; Plantar or Palmar Papilloma of Yaws |
| 102.2 | Yaws; Other Early Skin Lesions; Early Yaws (Cutaneous) (Macular) (Papular) (Maculopapular) (Micropapular); Frambeside of Early Yaws; ; Cutaneous Yaws, Less than Five Years after Infection |
| 102.3 | Yaws; Hyperkeratosis; Ghoul Hand; Hyperkeratosis, Palmar or Plantar (Early) (Late) Due to Yaws; Worm-eaten Soles |
| 102.4 | Yaws; Gummata and Ulcers; Nodular Late Yaws (Ulcerated); Gummatous Frambeside |
| 102.5 | Yaws; Gangosa; Rhinopharyngitis Mutilans |
| 102.6 | Yaws; Bone and Joint Lesions; Goundou of Yaws (Late); Gumma, Bone of Yaws (Late); Gummatous Osteitis or Periostitis of Yaws (Late); Hydrarthrosis of Yaws (Early) (Late); Osteitis of Yaws (Early) (Late); Periostitis (Hypertrophic) of Yaws (Early) (Late) |
| 102.7 | Yaws; Other Manifestations; Juxta-Articular Nodules of Yaws; Mucosal Yaws |
| 102.8 | Latent Yaws; Yaws without Clinical Manifestations, with Positive Serology |
| 102.9 | Yaws, unspecified |
| 103 | Pinta |
| 103.0 | Pinta; Primary lesions; Chancre (primary) of Pinta [carate]; Papule (primary) of Pinta [carate]; Pintid of Pinta [carate] |
| 103.1 | Pinta; Intermediate Lesions; Erythematous Plaques of Pinta [Carate]; Hyperchromic Lesions of Pinta [Carate]; Hyperkeratosis of Pinta [Carate] |
| 103.2 | Pinta; Late Lesions; Cardiovascular Lesions of Pinta [Carate]; Achromic of Pinta [Carate]; Cicatricial of Pinta [Carate]; Dyschromic of Pinta [Carate]; Vitiligo of Pinta [Carate] |
| 103.3 | Pinta; Mixed Lesions; Achromic and Hyperchromic Skin Lesions of Pinta [Carate] |
| 103.9 | Pinta, Unspecified |
| 104 | Spirochetal Infection, Other |
| 104.0 | Spirochetal Infection, Other ; Nonvenereal Endemic Syphilis; Bejel; Njovera |
| 104.8 | Spirochetal Infection, Other ; Other Specified Spirochetal Infections |
| 104.9 | Spirochetal Infection, Unspecified |
| 110 | Dermatophytosis |
| 110.0 | Dermatophytosis, Scalp and Beard; Kerion; Sycosis, Mycotic; Trichophytic Tinea, Scalp |
| 110.1 | Dermatophytosis, Nail; Dermatophytic Onychia; Onychomycosis; Tinea Unguium |
| 110.2 | Dermatophytosis, Hand; Tinea Manuum |
| 110.3 | Dermatophytosis, Groin and Perianal Area; Dhobie Itch; Eczema Marginatum; Tinea Cruris |
| 110.4 | Dermatophytosis, Foot; Athlete's Foot; Tinea Pedis |
| 110.5 | Dermatophytosis, Body; Herpes Circinatus; Tinea Imbricata |
| 110.6 | Dermatophytosis, Deep Seated; Granuloma Trichophyticum; Majocchi's Granuloma |
| 110.8 | Dermatophytosis, Specified Sites |
| 110.9 | Dermatophytosis, Unspecified Site; Favus NOS; Microsporic Tinea NOS; Ringworm NOS |
| 111 | Dermatomycosis, Other and Unspecified |
| 111.0 | Dermatomycosis, Other and Unspecified; Pityriasis Versicolor; Infection by Malassezia [Pityrosporum] Furfur; Tinea Flava; Tinea Versicolor |
| 111.1 | Dermatomycosis, Other and Unspecified; Tinea Nigra; Infection by Cladosporium Species; Keratomycosis Nigricans; Microsporosis Nigra; Pityriasis Nigra; Tinea Palmaris Nigra |
| 111.2 | Dermatomycosis, Other and Unspecified; Tinea Blanca; Infection by Trichosporon (Beigelii) Cutaneum; White Piedra |
| 111.3 | Dermatomycosis, Other and Unspecified; Black Piedra; Infection by Piedraia Hortai |
| 111.8 | Dermatomycosis, Other Specified |
| 111.9 | Dermatomycosis, Unspecified |
| 112 | Candidiasis |
| 112.0 | Candidiasis, Mouth; Thrush (Oral) |
| 112.1 | Candidiasis, Vulva and Vagina; Candidal Vulvovaginitis; Monilial Vulvovaginitis |
| 112.2 | Candidiasis, Other Urogenital Sites; Candidal Balanitis |
| 112.3 | Candidiasis, Skin and Nails; Candidal Intertrigo; Candidal Onychia; Candidal Perionyxis [Paronychia] |
| 112.4 | Candidiasis, Lung; Candidal Pneumonia |
| 112.5 | Candidiasis, Disseminated; Systemic Candidiasis |
| 112.8 | Candidiasis, Other Specified Sites |
| 112.81 | Candidal Endocarditis |
| 112.82 | Candidal Otitis Externa; Otomycosis in Moniliasis |
| 112.83 | Candidal Meningitis |
| 112.84 | Candidal Esophagitis |
| 112.85 | Candidal Enteritis |
| 112.89 | Candidiasis, Other Specified Sites; Other |
| 112.9 | Candidiasis, Unspecified Site |
| 114 | Coccidioidomycosis; Posada Wernicke Disease |
| 114.0 | Coccidioidomycosis, Primary Pulmonary; Acute Pulmonary Coccidioidomycosis; Coccidioidomycotic Pneumonitis; Desert Rheumatism; Pulmonary Coccidioidomycosis; San Joaquin Valley Fever |
| 114.1 | Coccidioidomycosis, Primary Extrapulmonary; Chancriform Syndrome; Primary Cutaneous Coccidioidomycosis |
| 114.2 | Coccidioidal Meningitis |
| 114.3 | Coccidioidomycosis, Other Forms of Progressive; Coccidioidal Granuloma; Disseminated Coccidioidomycosis |
| 114.4 | Coccidioidomycosis, Chronic Pulmonary |
| 114.5 | Coccidioidomycosis, Unspecified Pulmonary |
| 114.9 | Coccidioidomycosis, Unspecified |
| 115 | Histoplasmosis |
| 115.0 | Histoplasma capsulatum; American Histoplasmosis; Darling's Disease; Reticuloendothelial Cytomycosis, Small Form Histoplasmosis |
| 115.00 | Histoplasma capsulatum; American Histoplasmosis; Darling's Disease; Reticuloendothelial Cytomycosis, Small Form Histoplasmosis; without Mention of Manifestation |
| 115.01 | Histoplasma capsulatum; American Histoplasmosis; Darling's Disease; Reticuloendothelial Cytomycosis, Small Form Histoplasmosis; Meningitis |
| 115.02 | Histoplasma capsulatum; American Histoplasmosis; Darling's Disease; Reticuloendothelial Cytomycosis, Small Form Histoplasmosis; Retinitis |
| 115.03 | Histoplasma capsulatum; American Histoplasmosis; Darling's Disease; Reticuloendothelial Cytomycosis, Small Form Histoplasmosis; Pericarditis |
| 115.04 | Histoplasma capsulatum; American Histoplasmosis; Darling's Disease; Reticuloendothelial Cytomycosis, Small Form Histoplasmosis; Endocarditis |
| 115.05 | Histoplasma capsulatum; American Histoplasmosis; Darling's Disease; Reticuloendothelial Cytomycosis, Small Form Histoplasmosis; Pneumonia |
| 115.09 | Histoplasma capsulatum; American Histoplasmosis; Darling's Disease; Reticuloendothelial Cytomycosis, Small Form Histoplasmosis; Other |
| 115.1 | Infection by Histoplasma duboisii; African Histoplasmosis; Large Form Histoplasmosis |
| 115.10 | Infection by Histoplasma duboisii; African Histoplasmosis; Large Form Histoplasmosis; without Mention of Manifestation |
| 115.11 | Infection by Histoplasma duboisii; African Histoplasmosis; Large Form Histoplasmosis; Meningitis |
| 115.12 | Infection by Histoplasma duboisii; African Histoplasmosis; Large Form Histoplasmosis; Retinitis |
| 115.13 | Infection by Histoplasma duboisii; African Histoplasmosis; Large Form Histoplasmosis; Pericarditis |
| 115.14 | Infection by Histoplasma duboisii; African Histoplasmosis; Large Form Histoplasmosis; Endocarditis |
| 115.15 | Infection by Histoplasma duboisii; African Histoplasmosis; Large Form Histoplasmosis; Pneumonia |
| 115.19 | Infection by Histoplasma duboisii; African Histoplasmosis; Large Form Histoplasmosis; Other |
| 115.9 | Histoplasmosis, Unspecified; Histoplasmosis NOS |
| 115.90 | Histoplasmosis, Unspecified; Histoplasmosis NOS; without Mention of Manifestation |
| 115.91 | Histoplasmosis, Unspecified; Histoplasmosis NOS; Meningitis |
| 115.92 | Histoplasmosis, Unspecified; Histoplasmosis NOS; Retinitis |
| 115.93 | Histoplasmosis, Unspecified; Histoplasmosis NOS; Pericarditis |
| 115.94 | Histoplasmosis, Unspecified; Histoplasmosis NOS; Endocarditis |
| 115.95 | Histoplasmosis, Unspecified; Histoplasmosis NOS; Pneumonia |
| 115.99 | Histoplasmosis, Unspecified; Histoplasmosis NOS; Other |
| 116 | Blastomycotic Infection |
| 116.0 | Blastomycosis; Blastomycotic Dermatitis; Chicago Disease; Cutaneous Blastomycosis; Disseminated Blastomycosis; Gilchrist's Disease; Infection by Blastomyces Dermatitidis; North American Blastomycosis; Primary Pulmonary Blastomycosis |
| 116.1 | Paracoccidioidomycosis; Brazilian blastomycosis; Infection by Paracoccidioides [Blastomyces] brasiliensis; Lutz-Splendore-Almeida disease; Mucocutaneous-lymphangitic paracoccidioidomycosis; Pulmonary paracoccidioidomycosis; South American blastomycosis; Visceral paracoccidioidomycosis |
| 116.2 | Lobomycosis; Infections by Loboa [Blastomyces] loboi; Keloidal blastomycosis; Lobo's disease |
| 117 | Mycoses, Other |
| 117.0 | Rhinosporidiosis; Infection by Rhinosporidium seeberi |
| 117.1 | Sporotrichosis; Cutaneous Sporotrichosis; Disseminated Sporotrichosis; Infection by Sporothrix [Sporotrichum] Schenckii; Lymphocutaneous Sporotrichosis; Pulmonary Sporotrichosis; Sporotrichosis of the Bones |
| 117.2 | Chromoblastomycosis; Chromomycosis; Infection by Cladosporidium carrionii, Fonsecaea compactum, Fonsecaea pedrosoi, Phialophora verrucosa |
| 117.3 | Aspergillosis; Infection by Aspergillus species, mainly A. fumigatus, A. flavus Group, A. terreus Group |
| 117.4 | Mycotic Mycetomas; Infection by Various Genera and Species of Ascomycetes and Deuteromycetes, such as Acremonium [Cephalosporium] falciforme, Neotestudina rosatii, Madurella grisea, Madurella mycetomii, Pyrenochaeta romeroi, Zopfia [Leptosphaeria] senegalensis; Madura Foot, Mycotic; Maduromycosis, Mycotic |
| 117.5 | Cryptococcosis; Busse-Buschke's disease; European Cryptococcosis; Infection by Cryptococcus neoformans; Pulmonary Cryptococcosis; Systemic Cryptococcosis Torula |
| 117.6 | Allescheriosis [Petriellidosis]; Infections by Allescheria [Petriellidium] boydii [Monosporium apiospermum] |
| 117.7 | Zygomycosis [Phycomycosis or Mucormycosis]; Infection by Species of Absidia, Basidiobolus, Conidiobolus, Cunninghamella, Entomophthora, Mucor, Rhizopus, Saksenaea |
| 117.8 | Infection by Dematiacious Fungi [Phaehyphomycosis]; Infection by Dematiacious Fungi, Such as Cladosporium trichoides [bantianum], Dreschlera hawaiiensis, Phialophora gougerotii, Phialophora jeanselmi |
| 117.9 | Mycoses, Other and Unspecified |
| 118 | Opportunistic Mycoses; Infection of Skin, Subcutaneous Tissues, and/or Organs by a Wide Variety of Fungi Generally Considered to be Pathogenic to Compromised Hosts Only (e.g., Infection by Species of Alternaria, Dreschlera, Fusarium) |
| 120 | Schistosomiasis [bilharziasis] |
| 120.0 | Schistosoma haematobium; Vesical Schistosomiasis NOS |
| 120.1 | Schistosoma mansoni; Intestinal Schistosomiasis NOS |
| 120.2 | Schistosoma japonicum; Asiatic Schistosomiasis NOS; Katayama Disease or Fever |
| 120.3 | Cutaneous; Cercarial Dermatitis; Infection by Cercariae of Schistosoma; Schistosome Dermatitis; Swimmers' Itch |
| 120.8 | Schistosomiasis, Other Specified; Bovis; Intercalatum; Mattheii; Infection by Schistosoma Spindale; Schistosomiasis Chestermani |
| 120.9 | Schistosomiasis, Unspecified; Blood Flukes NOS; Hemic Distomiasis |
| 121 | Trematode Infections, Other |
| 121.0 | Opisthorchiasis; Cat Liver Fluke; Opisthorchis (Felineus) (Tenuicollis) (Viverrini) |
| 121.1 | Clonorchiasis; Biliary Cirrhosis Due to Clonorchiasis; Chinese Liver Fluke Disease; Hepatic Distomiasis Due to Clonorchis Sinensis; Oriental Liver Fluke Disease |
| 121.2 | Paragonimiasis; Infection by Paragonimus; Lung Fluke Disease (Oriental); Pulmonary Distomiasis |
| 121.3 | Fascioliasis; Infection by Fasciola: gigantica, Hepatica; Liver Flukes NOS; Sheep Liver Fluke Infection |
| 121.4 | Fasciolopsiasis; Infection by Fasciolopsis (buski); Intestinal Distomiasis |
| 121.5 | Metagonimiasis; Infection by Metagonimus Yokogawai |
| 121.6 | Heterophyiasis; Heterophyes Heterophyes; Stellantchasmus Falcatus |
| 121.8 | Trematode Infections, Other Specified; Dicrocoelium dendriticum; Echinostoma ilocanum; Gastrodiscoides hominis |
| 121.9 | Trematode Infection, Unspecified; Distomiasis NOS; Fluke Disease NOS |
| 122 | Echinococcosis |
| 122.0 | Echinococcus Granulosus Infection of Liver |
| 122.1 | Echinococcus Granulosus Infection of Lung |
| 122.2 | Echinococcus Granulosus Infection of Thyroid |
| 122.3 | Echinococcus Granulosus Infection, Other |
| 122.4 | Echinococcus Granulosus Infection, Unspecified |
| 122.5 | Echinococcus Multilocularis Infection of Liver |
| 122.6 | Echinococcus Multilocularis Infection, Other |
| 122.7 | Echinococcus Multilocularis Infection, Unspecified |
| 122.8 | Echinococcus, Unspecified, of Liver |
| 122.9 | Echinococcus, Other and Unspecified |
| 123 | Infection, Other |
| 123.0 | Taenia solium Infection, Intestinal Form; Pork Tapeworm (Adult) (Infection) |
| 123.1 | Cysticercosis; Cysticerciasis; Infection by Cysticercus Cellulosae [Larval Form of Taenia solium] |
| 123.2 | Taenia saginata Infection; Beef Tapeworm (Infection); Infection by Taeniarhynchus saginatus |
| 123.3 | Taeniasis, Unspecified |
| 123.4 | Diphyllobothriasis, Intestinal; Diphyllobothrium (Adult) (Latum) (Pacificum) Infection; Fish Tapeworm (Infection) |
| 123.5 | Sparganosis [Larval Diphyllobothriasis]; Diphyllobothrium Larvae; Sparganum (Mansoni) (Proliferum); Spirometra Larvae |
| 123.6 | Hymenolepiasis; Dwarf Tapeworm (Infection); Hymenolepis (Diminuta) (Nana) Infection; Rat Tapeworm (Infection) |
| 123.8 | Cestode Infection, Other Specified; Diplogonoporus (Grandis) Infection; Dipylidium (Caninum) Infection; Dog Tapeworm (Infection) |
| 123.9 | Cestode Infection, Unspecified; Tapeworm, Infection, NOS |
| 124 | Trichinosis; Trichinella spiralis Infection; Trichinellosis; Trichiniasis |
| 125 | Filarial Infection and Dracontiasis |
| 125.0 | Bancroftian Filariasis; Chyluria Due to Wuchereria bancrofti; Elephantiasis Due to Wuchereria bancrofti; Infection Due to Wuchereria bancrofti; Lymphadenitis Due to Wuchereria bancrofti; Wuchereriasis |
| 125.1 | Malayan filariasis; Brugia filariasis Due to Brugia [Wuchereria] malayi; Chyluria Due to Brugia [Wuchereria] malayi; Elephantiasis Due to Brugia [Wuchereria] malayi; Infection Due to Brugia [Wuchereria] malayi; Lymphadenitis Due to Brugia [Wuchereria] malayi; Lymphangitis Due to Brugia [Wuchereria] malayi |
| 125.2 | Loiasis; Eyeworm Disease of Africa; Loa loa Infection |
| 125.3 | Onchocerciasis; Onchocerca Volvulus Infection; Onchocercosis |
| 125.4 | Dipetalonemiasis; Acanthocheilonema perstans; Dipetalonema perstans |
| 125.5 | Mansonella ozzardi Infection; Filariasis ozzardi |
| 125.6 | Filariasis, Other Specified; Dirofilaria Infection; Infection by: Acanthocheilonema streptocerca, Dipetalonema streptocerca |
| 125.7 | Dracontiasis; Guinea-worm infection; Infection by Dracunculus medinensis |
| 125.9 | Filariasis, Unspecified |
| 126 | Ancylostomiasis and Necatoriasis |
| 126.0 | Ancylostomiasis and Necatoriasis; Ancylostoma duodenale |
| 126.1 | Ancylostomiasis and Necatoriasis; Necator americanus |
| 126.2 | Ancylostomiasis and Necatoriasis; Ancylostoma braziliense |
| 126.3 | Ancylostomiasis and Necatoriasis; Ancylostoma ceylanicum |
| 126.8 | Ancylostoma, Other Specified |
| 126.9 | Ancylostomiasis and Necatoriasis, Unspecified |
| 127 | Intestinal Helminthiases, Other |
| 127.0 | Ascariasis; Ascaridiasis; Infection by Ascaris lumbricoides; Roundworm infection |
| 127.1 | Anisakiasis; Infection by Anisakis Larva |
| 127.2 | Strongyloidiasis; Infection by Strongyloides stercoralis |
| 127.3 | Trichuriasis; Infection by Trichuris trichiuria; Trichocephaliasis; Whipworm (Disease) (Infection) |
| 127.4 | Enterobiasis; Infection by Enterobius vermicularis; Oxyuriasis; Oxyuris vermicularis Infection; Pinworm (Disease) (Infection); Threadworm Infection |
| 127.5 | Capillariasis; Infection by Capillaria philippinensis |
| 127.6 | Trichostrongyliasis; Infection by Trichostrongylus Species |
| 127.7 | Intestinal Helminthiasis, Other Specified; Oesophagostomum Apiostomum and Related Species; Ternidens Diminutus; Other Specified Intestinal Helminth; Physalopteriasis |
| 127.8 | Mixed Intestinal Helminthiasis |
| 127.9 | Intestinal Helminthiasis, Unspecified |
| 128 | Helminthiases, Other and Unspecified |
| 128.0 | Toxocariasis; Larva migrans visceralis; Toxocara (Canis) (Cati) Infection; Visceral larva migrans Syndrome |
| 128.1 | Gnathostomiasis; Infection by Gnathostoma spinigerum and Related Species |
| 128.8 | Helminthiasis, Other Specified; Angiostrongylus Cantonensis; Capillaria Hepatica; Other Specified Helminth |
| 128.9 | Helminth Infection, Unspecified; Helminthiasis NOS; Worms NOS |
| 129 | Intestinal Parasitism, Unspecified |
| 130 | Toxoplasmosis |
| 130.0 | Meningoencephalitis Due to Toxoplasmosis; Encephalitis Due to Acquired Toxoplasmosis |
| 130.1 | Conjunctivitis Due to Toxoplasmosis |
| 130.2 | Chorioretinitis Due to Toxoplasmosis; Focal retinochoroiditis Due to Acquired Toxoplasmosis |
| 130.3 | Myocarditis Due to Toxoplasmosis |
| 130.4 | Pneumonitis Due to Toxoplasmosis |
| 130.5 | Hepatitis Due to Toxoplasmosis |
| 130.7 | Toxoplasmosis of Other Unspecified Sites |
| 130.8 | Multisystemic Disseminated Toxoplasmosis; Toxoplasmosis of Multiple Sites |
| 130.9 | Toxoplasmosis, Unspecified |
| 131 | Trichomoniasis |
| 131.0 | Trichomoniasis, Urogenital |
| 131.00 | Trichomoniasis, Unspecified Urogenital; Fluor (Vaginalis) Trichomonal or Due to Trichomonas (Vaginalis); Leukorrhea (Vaginalis) Trichomonal or Due to Trichomonas (Vaginalis) |
| 131.01 | Trichomonal Vulvovaginitis; Vaginitis, Trichomonal or Due to Trichomonas |
| 131.02 | Trichomonal Urethritis |
| 131.03 | Trichomonal Prostatitis |
| 131.09 | Trichomoniasis, Urogenital, Other |
| 131.8 | Trichomoniasis, Other Specified Sites |
| 131.9 | Trichomoniasis, Unspecified |
| 132 | Pediculosis and Phthirus Infestation |
| 132.0 | Pediculus capitis [Head Louse] |
| 132.1 | Pediculus corporis [Body Louse] |
| 132.2 | Phthirus pubis [Pubic Louse]; Pediculus pubis |
| 132.3 | Pediculosis and Phthirus Infestation; Mixed Infestation |
| 132.9 | Pediculosis, Unspecified |
| 133 | Acariasis |
| 133.0 | Scabies; Infestation by Sarcoptes scabiei; Norwegian Scabies; Sarcoptic Itch |
| 133.8 | Acariasis, Other; Chiggers; Demodex Folliculorum; Trombicula |
| 133.9 | Acariasis, unspecified; Infestation by Mites NOS |
| 134 | Infestation, Other |
| 134.0 | Myiasis; Dermatobia (hominis); Fly Larvae; Gasterophilus (intestinalis); Maggots; Oestrus ovis |
| 134.1 | Arthropod Infestation, Other; Chigoe; Sand Flea; Tunga Penetrans; Jigger Disease; Scarabiasis; Tungiasis |
| 134.2 | Hirudiniasis; Hirudiniasis (External) (Internal); Leeches (Aquatic) (Land) |
| 134.8 | Infestations, Other Specific |
| 134.9 | Infestation, Unspecified; Infestation (Skin) NOS; Skin Parasites NOS |
| 135 | Sarcoidosis; Besnier-Boeck-Schaumann Disease; Lupoid (Miliary) of Boeck; Lupus pernio (Besnier); Lymphogranulomatosis, Benign (Schaumann's); Sarcoid (Any Site): NOS, Boeck, Darier-Roussy; Uveoparotid Fever |
| 136 | Infectious and Parasitic Diseases, Other and Unspecified |
| 136.0 | Ainhum; Dactylolysis spontanea |
| 136.1 | BehÃ§et's Syndrome |
| 136.2 | Specific Infections by Free-living Amebae |
| 136.21 | Specific infection due to acanthamoeba |
| 136.29 | Other specific infections by free-living amebae; Meningoencephalitis due to Naegleria |
| 136.3 | Pneumocystosis; Pneumonia Due to Pneumocystis carinii; Pneumonia Due to Pneumocystis jiroveci |
| 136.4 | Psorospermiasis |
| 136.5 | Sarcosporidiosis; Infection by Sarcocystis lindemanni |
| 136.8 | Infectious and Parasitic Diseases, Other Specified; Candiru Infestation |
| 136.9 | Infectious and Parasitic Diseases, Unspecified; Infectious Disease NOS; Parasitic Disease NOS |
| 137 | Late Effects of Tuberculosis |
| 137.0 | Late Effects of Respiratory or Unspecified Tuberculosis |
| 137.1 | Late Effects of Central Nervous System Tuberculosis |
| 137.2 | Late Effects of Genitourinary Tuberculosis |
| 137.3 | Late Effects of Tuberculosis of Bones and Joints |
| 137.4 | Late Effects of Tuberculosis of Other Specified Organs |
| 138 | Late Effects of Acute Poliomyelitis |
| 139 | Late Effects of Other Infectious and Parasitic Diseases |
| 139.0 | Late Effects of Viral Encephalitis |
| 139.1 | Late Effects of Trachoma |
| 139.8 | Late Effects of Other and Unspecified Infectious and Parasitic Diseases |
| 140 | Malignant Neoplasm of Lip |
| 140.0 | Malignant Neoplasm of Lip; Upper Lip, Vermilion Border; Upper Lip: NOS, External, Lipstick Area |
| 140.1 | Malignant Neoplasm of Lip; Lower Lip, Vermilion Border; Lower Lip: NOS, External; Lipstick Area |
| 140.3 | Malignant Neoplasm of Lip; Upper Lip, Inner Aspect; Upper Lip: Buccal Aspect, Frenulum; Mucosa, Oral Aspect |
| 140.4 | Malignant Neoplasm of Lip; Lower Lip, Inner Aspect; Buccal Aspect; Frenulum; Mucosa; Oral Aspect |
| 140.5 | Malignant Neoplasm of Lip; Lip, Unspecified, Inner aspect; Buccal aspect; Frenulum; Mucosa; Oral Aspect |
| 140.6 | Malignant Neoplasm of Lip; Commissure of Lip; Labial Commissure |
| 140.8 | Malignant Neoplasm of Lip; Other Sites of Lip; Malignant Neoplasm of Contiguous or Overlapping Sites of Lip Whose Point of Origin Cannot be Determined |
| 140.9 | Malignant Neoplasm of Lip; Lip, Unspecified, Vermilion Border; External; Lipstick Area |
| 141 | Malignant Neoplasm of Tongue |
| 141.0 | Malignant Neoplasm of Tongue; Base of Tongue; Dorsal Surface of Base of Tongue; Fixed Part of Tongue NOS |
| 141.1 | Malignant Neoplasm of Tongue; Dorsal Surface of Tongue; Anterior Two-thirds of Tongue, Dorsal Surface; Dorsal Tongue NOS; Midline of Tongue |
| 141.2 | Malignant Neoplasm of Tongue; Tip and Lateral Border of Tongue |
| 141.3 | Malignant Neoplasm of Tongue; Ventral Surface of Tongue; Anterior Two-thirds of Tongue, Ventral Surface; Frenulum Linguae |
| 141.4 | Malignant Neoplasm of Tongue; Anterior Two-thirds of Tongue, Malignant, Part Unspecified; Mobile Part of Tongue NOS |
| 141.5 | Malignant Neoplasm of Tongue; Junctional Zone |
| 141.6 | Neoplasm, Lingual Tonsil, Malignant |
| 141.8 | Neoplasm, Other Sites of Tongue, Malignant |
| 141.9 | Malignant Neoplasm of Tongue; Tongue, Unspecified; Tongue NOS |
| 142 | Neoplasm, Major Salivary Glands, Malignant |
| 142.0 | Malignant Neoplasm, Parotid Gland |
| 142.1 | Malignant Neoplasm, Submandibular Gland; Submaxillary Gland |
| 142.2 | Malignant Neoplasm, Sublingual Gland |
| 142.8 | Malignant Neoplasm, Other Major Salivary Glands; Malignant Neoplasm of Contiguous or Overlapping Sites of Salivary Glands and Ducts Whose Point of Origin Cannot be Determined |
| 142.9 | Malignant Neoplasm, Salivary Gland, Unspecified |
| 143 | Neoplasm, Gum, Malignant |
| 143.0 | Malignant Neoplasm, Upper Gum |
| 143.1 | Malignant Neoplasm, Lower Gum |
| 143.8 | Malignant Neoplasm, Other Sites of Gum; Malignant Neoplasm of Contiguous or Overlapping Sites of Gum Whose Point of Origin Cannot Be Determined |
| 143.9 | Malignant Neoplasm, Gum, Unspecified |
| 144 | Neoplasm, Floor of Mouth, Malignant |
| 144.0 | Malignant Neoplasm, Anterior Portion; Anterior to the Premolar-Canine Junction |
| 144.1 | Malignant Neoplasm, Lateral Portion |
| 144.8 | Malignant Neoplasm, Other Sites of Floor of Mouth; Malignant Neoplasm of Contiguous or Overlapping Sites of Floor of Mouth Whose Point of Origin Cannot Be Determined |
| 144.9 | Malignant Neoplasm, Floor of Mouth, Part Unspecified |
| 145 | Neoplasm, Other and Unspecified Parts of Mouth, Malignant |
| 145.0 | Malignant Neoplasm, Cheek Mucosa; Buccal Mucosa; Cheek, Inner Aspect |
| 145.1 | Neoplasm, Other and Unspecified Parts of Mouth, Malignant; Vestibule of mouth; Buccal sulcus (Upper) (Lower); Labial sulcus (Upper) (Lower) |
| 145.2 | Neoplasm, Other and Unspecified Parts of Mouth, Malignant; Hard Palate |
| 145.3 | Neoplasm, Other and Unspecified Parts of Mouth, Malignant; Soft Palate |
| 145.4 | Neoplasm, Other and Unspecified Parts of Mouth, Malignant; Uvula |
| 145.5 | Neoplasm, Palate, Malignant, Unspecified; Unction of Hard and Soft Palate; Roof of Mouth |
| 145.6 | Neoplasm, Other and Unspecified Parts of Mouth, Malignant; Retromolar Area |
| 145.8 | Malignant Neoplasm, Other Specified Parts of Mouth; Malignant Neoplasm of Contiguous or Overlapping Sites of Mouth Whose Point of Origin Cannot be Determined |
| 145.9 | Malignant Neoplasm, Mouth; Unspecified Site, Buccal Cavity NOS, Minor Salivary Gland Unspecified Site, Oral Cavity NOS |
| 146 | Neoplasm, Oropharynx, Malignant |
| 146.0 | Neoplasm, Tonsil, Malignant, NOS, Faucial, Palatine |
| 146.1 | Neoplasm, Tonsillar Fossa, Malignant |
| 146.2 | Neoplasm, Tonsillar Pillars, Malignant; Faucial Pillar; Glossopalatine Fold; Palatoglossal Arch; Palatopharyngeal Arch |
| 146.3 | Neoplasm, Vallecula, Malignant |
| 146.4 | Neoplasm, Anterior Aspect of Epiglottis, Malignant; Epiglottis, Free border; Glossoepiglottic Fold(s) |
| 146.5 | Neoplasm, Junctional Region, Malignant |
| 146.6 | Neoplasm, Lateral Wall of Oropharynx, Malignant |
| 146.7 | Neoplasm, Posterior Wall of Oropharynx, Malignant |
| 146.8 | Neoplasm, Other Specified Sites of Oropharynx, Malignant; Branchial Cleft |
| 146.9 | Malignant Neoplasm, Oropharynx, Unspecified |
| 147 | Neoplasm, Nasopharynx, Malignant |
| 147.0 | Malignant Neoplasm, Superior Wall; Roof of Nasopharynx |
| 147.1 | Malignant Neoplasm, Posterior Wall; Adenoid; Pharyngeal Tonsil |
| 147.2 | Malignant Neoplasm, Lateral Wall; Fossa of RosenmÃ¼ller; Opening of Auditory Tube; Pharyngeal Recess |
| 147.3 | Malignant Neoplasm, Anterior Wall; Floor of Nasopharynx; Nasopharyngeal [Posterior] [Superior] Surface of Soft palate; Posterior Margin of Nasal Septum and Choanae |
| 147.8 | Malignant Neoplasm, Other Specified Sites of Nasopharynx; Malignant Neoplasm of Contiguous or Overlapping Sites of Nasopharynx Whose Point of Origin Cannot be Determined |
| 147.9 | Malignant Neoplasm, Nasopharynx, Unspecified |
| 148 | Neoplasm, Hypopharynx, Malignant |
| 148.0 | Malignant Neoplasm, Postcricoid Region |
| 148.1 | Malignant Neoplasm, Pyriform Sinus; Pyriform Fossa |
| 148.2 | Malignant Neoplasm, Aryepiglottic Fold, Hypopharyngeal Aspect; Aryepiglottic Fold or Interarytenoid Fold: NOS, Marginal Zone |
| 148.3 | Malignant Neoplasm, Posterior Hypopharyngeal Wall |
| 148.8 | Malignant Neoplasm, Other Specified Sites of Hypopharynx; Malignant Neoplasm of Contiguous or Overlapping Sites of Hypopharynx Whose Point of Origin Cannot Be Determined |
| 148.9 | Malignant Neoplasm, Hypopharynx, Unspecified |
| 149 | Neoplasm, Other and Ill-defined Sites within the Lip, Oral Cavity, and Pharynx, Malignant |
| 149.0 | Malignant Neoplasm, Pharynx, Unspecified |
| 149.1 | Malignant Neoplasm, Waldeyer's Ring |
| 149.8 | Malignant Neoplasm, Other |
| 149.9 | Malignant Neoplasm, Ill-defined |
| 150 | Neoplasm, Esophagus, Malignant |
| 150.0 | Neoplasm, Cervical Esophagus, Malignant |
| 150.1 | Neoplasm, Thoracic Esophagus, Malignant |
| 150.2 | Neoplasm, Abdominal Esophagus, Malignant |
| 150.3 | Neoplasm, Upper Third of Esophagus, Malignant |
| 150.4 | Neoplasm, Middle Third of Esophagus, Malignant |
| 150.5 | Neoplasm, Lower Third of Esophagus, Malignant |
| 150.8 | Neoplasm, Other Specified Part of Esophagus, Malignant |
| 150.9 | Malignant Neoplasm, Esophagus, Unspecified |
| 151 | Malignant Neoplasm of Stomach |
| 151.0 | Neoplasm, Cardia, Malignant; Cardiac Orifice; Cardio-esophageal Junction |
| 151.1 | Neoplasm, Pylorus, Malignant; Prepylorus; Pyloric Canal |
| 151.2 | Neoplasm, Pyloric Antrum, Malignant ; Antrum of Stomach NOS |
| 151.3 | Neoplasm, Fundus of Stomach, Malignant |
| 151.4 | Neoplasm, Body of Stomach, Malignant |
| 151.5 | Neoplasm, Lesser Curvature, Unspecified, Malignant |
| 151.6 | Neoplasm, Greater Curvature, Unspecified, Malignant |
| 151.8 | Neoplasm, Other Specified Sites of Stomach, Malignant |
| 151.9 | Cancer, Stomach, Unspecified Site; Carcinoma Ventriculi, Gastric Cancer |
| 152 | Neoplasm, Small Intestine (Include Duodenum), Malignant |
| 152.0 | Neoplasm, Duodenum, Malignant |
| 152.1 | Neoplasm, Jejunum, Malignant |
| 152.2 | Neoplasm, Ileum, Malignant |
| 152.3 | Neoplasm, Meckel's Diverticulum, Malignant |
| 152.8 | Neoplasm, Other Specified Sites of Small Intestine, Malignant |
| 152.9 | Neoplasm, Small Intestine, Unspecified Site, Malignant |
| 153 | Neoplasm, Colon, Malignant |
| 153.0 | Neoplasm, Hepatic Flexure of Colon, Malignant |
| 153.1 | Neoplasm, Transverse Colon, Malignant |
| 153.2 | Neoplasm, Descending Colon, Malignant; Left Colon |
| 153.3 | Neoplasm, Sigmoid Colon, Malignant; Sigmoid Flexure |
| 153.4 | Neoplasm, Cecum, Malignant |
| 153.5 | Neoplasm, Appendix, Malignant |
| 153.6 | Malignant Neoplasm, Ascending Colon, Right Colon |
| 153.7 | Neoplasm, Splenic Flexure of Colon, Malignant |
| 153.8 | Neoplasm, Other Specified Sites of Large Intestine, Malignant |
| 153.9 | Neoplasm, Colon, Malignant, Unspecified Site; Large Intestine NOS |
| 154 | Neoplasm, Rectum, Rectosigmoid Junction and Anus, Malignant |
| 154.0 | Malignant Neoplasm of Rectum, Rectosigmoid Junction, and Anus; Rectosigmoid Junction; Colon with Rectum; Rectosigmoid |
| 154.1 | Malignant Neoplasm of Rectum, Rectosigmoid Junction, and Anus; Rectosigmoid Junction; Rectum; Rectal Ampulla |
| 154.2 | Malignant Neoplasm of Rectum, Rectosigmoid Junction, and Anus; Rectosigmoid Junction; Anal Canal; Anal Sphincter |
| 154.3 | Neoplasm, Anus, Malignant, Unspecified |
| 154.8 | Neoplasm, Other Sites of Rectum, Rectosigmoid Junction, and Anus, Malignant |
| 155 | Neoplasm, Liver and Intrahepatic Bile Ducts, Malignant |
| 155.0 | Malignant Neoplasm of Liver and Intrahepatic Bile Ducts; Liver, Primary: Carcinoma: Liver, Specified as Primary; Hepatocellular; Liver Cell; Hepatoblastoma |
| 155.1 | Malignant Neoplasm of Liver and Intrahepatic Bile Ducts; Intrahepatic Bile Ducts; Canaliculi Biliferi; Interlobular: Bile Ducts, Biliary Canals; Intrahepatic: Biliary Passages, Canaliculi, Gall Duct |
| 155.2 | Angiosarcoma, Hepatic |
| 156 | Malignant Neoplasm of Gallbladder and Extrahepatic Bile Ducts |
| 156.0 | Gallbladder |
| 156.1 | Extrahepatic Bile Ducts; Biliary Duct or Passage; Common Bile Duct; Cystic Duct; Hepatic Duct; Sphincter of Oddi |
| 156.2 | Ampulla of Vater |
| 156.8 | Other Specified Sites of Gallbladder and Extrahepatic Bile Ducts; Malignant Neoplasm of Contiguous or Overlapping Sites of Gallbladder and Extrahepatic Bile Ducts whose Point of Origin Cannot be Determined |
| 156.9 | Biliary Tract, Part Unspecified; Malignant Neoplasm Involving both Intrahepatic and Extrahepatic Bile Ducts |
| 157 | Cancer, Pancreas |
| 157.0 | Neoplasm, Head of Pancreas, Malignant |
| 157.1 | Neoplasm, Body of Pancreas, Malignant |
| 157.2 | Neoplasm, Tail of Pancreas, Malignant |
| 157.3 | Malignant Neoplasm of Pancreas; Pancreatic Duct; Duct Of: Santorini, Wirsung |
| 157.4 | Malignant Neoplasm of Pancreas; Islets of Langerhans; Islets of Langerhans, Any Part of Pancreas |
| 157.8 | Malignant Neoplasm of Pancreas; Other Specified Sites of Pancreas; Ectopic Pancreatic Tissue; Malignant Neoplasm of Contiguous or Overlapping Sites of Pancreas Whose Point of Origin Cannot be Determined |
| 157.9 | Cancer, Pancreas, Part Unspecified |
| 158 | Malignant Neoplasm of Retroperitoneum and Peritoneum |
| 158.0 | Retroperitoneum; Periadrenal Tissue; Perinephric Tissue; Perirenal Tissue; Retrocecal Tissue |
| 158.8 | Specified Parts of Peritoneum; Cul-de-sac (of Douglas); Mesentery; Mesocolon; Omentum; Parietal; Pelvic; Rectouterine Pouch; Malignant Neoplasm of Contiguous or Overlapping Sites of Retroperitoneum and Peritoneum whose Point of Origin Cannot be Determined |
| 158.9 | Peritoneum, Unspecified |
| 159 | Malignant Neoplasm of Other and Ill-defined Sites within the Digestive Organs and Peritoneum |
| 159.0 | Intestinal Tract, Part Unspecified |
| 159.1 | Spleen, Not Elsewhere Classified; Angiosarcoma of Spleen; Fibrosarcoma of Spleen |
| 159.8 | Other Sites of Digestive System and Intra-abdominal Organs; |
| 159.9 | Malignant Neoplasm of Retroperitoneum and Peritoneum, Ill-defined |
| 160 | Malignant Neoplasm of nasal Cavities, Middle Ear, and Accessory Sinuses |
| 160.0 | Nasal Cavities; Cartilage of Nose; Conchae, Nasal; Internal Nose; Septum of Nose; Vestibule of Nose |
| 160.1 | Auditory Tube, Middle Ear, and Mastoid Air Cells; Antrum Tympanicum; Eustachian Tube; Tympanic Cavity |
| 160.2 | Maxillary Sinus; Antrum (Highmore) (Maxillary) |
| 160.3 | Ethmoidal Sinus |
| 160.4 | Frontal Sinus |
| 160.5 | Sphenoidal Sinus |
| 160.8 | Nasal Cavities, Other; Malignant Neoplasm of Contiguous or Overlapping Sites of Nasal Cavities, Middle Ear, and Accessory Sinuses whose Point of Origin Cannot be Determined |
| 160.9 | Accessory Sinus, Unspecified |
| 161 | Malignant Neoplasm of Larynx |
| 161.0 | Malignant Neoplasm of Larynx, Glottis; Intrinsic Larynx; Laryngeal Commissure (Anterior) (Posterior); True Vocal Cord; Vocal Cord NOS |
| 161.1 | Malignant Neoplasm of Larynx, Supraglottis; Aryepiglottic Fold or Interarytenoid Fold, Laryngeal Aspect; Extrinsic Larynx; False Vocal Cords; Posterior (Laryngeal) Surface of Epiglottis; Ventricular Bands |
| 161.2 | Malignant Neoplasm of Larynx, Subglottis |
| 161.3 | Malignant Neoplasm of Larynx, Laryngeal Cartilages; Cartilage: Arytenoid, Cricoid, Cuneiform, Thyroid |
| 161.8 | Malignant Neoplasm of Larynx, Other Specified Sites of Larynx; Malignant Neoplasm of Contiguous or Overlapping Sites of Larynx whose Point of Origin Cannot be Determined |
| 161.9 | Malignant Neoplasm of Larynx, Larynx, Unspecified |
| 162 | Malignant Neoplasm of Trachea, Bronchus, and Lung |
| 162.0 | Malignant Neoplasm of Trachea, Bronchus, and Lung; Trachea; Cartilage of Trachea; Mucosa of Trachea |
| 162.2 | Malignant Neoplasm of Trachea, Bronchus, and Lung; Trachea; Main Bronchus; Carina; Hilus of Lung |
| 162.3 | Neoplasm, Upper Lobe, Bronchus or Lung, Malignant |
| 162.4 | Neoplasm, Middle Lobe, Bronchus or Lung, Malignant |
| 162.5 | Neoplasm, Lower Lobe, Bronchus or Lung, Malignant |
| 162.8 | Neoplasm, Other Parts of Bronchus or Lung, Malignant |
| 162.9 | Malignant Neoplasm of Trachea, Bronchus, and Lung; Bronchus and Lung, Unspecified |
| 163 | Neoplasm, Pleura, Malignant |
| 163.0 | Neoplasm, Parietal Pleura, Malignant |
| 163.1 | Neoplasm, Visceral Pleura, Malignant |
| 163.8 | Neoplasm, Other Specified Sites of Pleura, Malignant |
| 163.9 | Neoplasm, Pleura, Malignant, Unspecified Site |
| 164 | Malignant Neoplasm of Thymus, Heart, and Mediastinum |
| 164.0 | Malignant Neoplasm of Thymus, Heart, and Mediastinum, Thymus |
| 164.1 | Malignant Neoplasm of Thymus, Heart, and Mediastinum, Heart; Endocardium; Epicardium; Myocardium; Pericardium |
| 164.2 | Malignant Neoplasm of Thymus, Heart, and Mediastinum, Anterior Mediastinum |
| 164.3 | Malignant Neoplasm of Thymus, Heart, and Mediastinum, Posterior Mediastinum |
| 164.8 | Malignant Neoplasm of Thymus, Heart, and Mediastinum, Other; Malignant Neoplasm of Contiguous or Overlapping Sites of Thymus, Heart, and Mediastinum Whose Point of Origin Cannot be Determined |
| 164.9 | Malignant Neoplasm of Thymus, Heart, and Mediastinum, Mediastinum, Part Unspecified |
| 165 | Malignant Neoplasm of Other and Ill-defined Sites within the Respiratory System and Intrathoracic Organs |
| 165.0 | Upper Respiratory Tract, Part Unspecified |
| 165.8 | Malignant Neoplasm of Other and Ill-defined Sites within the Respiratory System and Intrathoracic Organs, Other |
| 165.9 | Ill-defined Sites within the Respiratory System |
| 170 | Malignant Neoplasm of Bone and Articular Cartilage |
| 170.0 | Malignant Neoplasm of Bone and Articular Cartilage; Bone: Ethmoid, Frontal, Malar, Nasal, Occipital, Orbital, Parietal, Sphenoid, Temporal, Zygomatic, Maxilla (Superior), Turbinate, Upper Jaw Bone, Vomer |
| 170.1 | Malignant Neoplasm of Bone and Articular Cartilage; Mandible; Inferior Maxilla; Jaw Bone NOS; Lower Jaw Bone |
| 170.2 | Malignant Neoplasm of Bone and Articular Cartilage; Mandible; Vertebral Column, Excluding Sacrum and Coccyx; Spinal Column; Spine: Vertebra |
| 170.3 | Malignant Neoplasm of Bone and Articular Cartilage; Ribs, Sternum, and Clavicle; Costal Cartilage; Costovertebral Joint; Xiphoid Process |
| 170.4 | Malignant Neoplasm of Bone and Articular Cartilage; Scapula and Long Bones of Upper Limb; Acromion; Bones NOS of Upper Limb; Humerus; Radius; Ulna |
| 170.5 | Malignant Neoplasm of Bone and Articular Cartilage; Short Bones of Upper Limb; Carpal; Cuneiform, Wrist; Metacarpal; Navicular, of Hand; Phalanges of Hand; Pisiform; Scaphoid (of Hand); Semilunar or Lunate; Trapezium; Trapezoid; Unciform |
| 170.6 | Malignant Neoplasm of Bone and Articular Cartilage; Pelvic Bones, Sacrum, and Coccyx; Coccygeal Vertebra; Ilium; Ischium; Pubic Bone; Sacral Vertebra |
| 170.7 | Malignant Neoplasm of Bone and Articular Cartilage; Bones NOS of Lower Limb; Femur; Fibula; Tibia |
| 170.8 | Malignant Neoplasm of Bone and Articular Cartilage; Short Bones of Lower Limb; Astragalus [Talus]; Calcaneus; Cuboid; Cuneiform, Ankle; Metatarsal; Navicular (of Ankle); Patella; Phalanges of Foot; Tarsal |
| 170.9 | Malignant Neoplasm of Bone and Articular Cartilage; Bone and Articular Cartilage, Site Unspecified |
| 171 | Malignant Neoplasm of Connective and Other Soft Tissue; Bursa; Fascia; Fat; Ligament, except Uterine; Muscle; Peripheral, Sympathetic, and Parasympathetic Nerves and Ganglia; Synovia; Tendon (Sheath) |
| 171.0 | Malignant Neoplasm of Connective and Other Soft Tissue; Bursa; Fascia; Fat; Ligament, except Uterine; Muscle; Peripheral, Sympathetic, and Parasympathetic Nerves and Ganglia; Synovia; Tendon (Sheath), Head, Face, and Neck; Ear; Eyelid |
| 171.2 | Malignant Neoplasm of Connective and Other Soft Tissue; Bursa; Fascia; Fat; Ligament, except Uterine; Muscle; Peripheral, Sympathetic, and Parasympathetic Nerves and Ganglia; Synovia; Tendon (Sheath), Upper Limb, Including Shoulder; Arm; Finger; Forearm; Hand |
| 171.3 | Malignant Neoplasm of Connective and Other Soft Tissue; Bursa; Fascia; Fat; Ligament, except Uterine; Muscle; Peripheral, Sympathetic, and Parasympathetic Nerves and Ganglia; Synovia; Tendon (Sheath), Lower Limb, Including Hip; Foot; Leg; Popliteal Space; Thigh; Toe |
| 171.4 | Malignant Neoplasm of Connective and Other Soft Tissue; Bursa; Fascia; Fat; Ligament, except Uterine; Muscle; Peripheral, Sympathetic, and Parasympathetic Nerves and Ganglia; Synovia; Tendon (Sheath), Thorax; Axilla; Diaphragm; Great Vessels |
| 171.5 | Malignant Neoplasm of Connective and Other Soft Tissue; Bursa; Fascia; Fat; Ligament, except Uterine; Muscle; Peripheral, Sympathetic, and Parasympathetic Nerves and Ganglia; Synovia; Tendon (Sheath), Abdomen; Abdominal Wall; Hypochondrium |
| 171.6 | Malignant Neoplasm of Connective and Other Soft Tissue; Bursa; Fascia; Fat; Ligament, except Uterine; Muscle; Peripheral, Sympathetic, and Parasympathetic Nerves and Ganglia; Synovia; Tendon (Sheath), Pelvis; Buttock; Groin; Inguinal Region; Perineum |
| 171.7 | Malignant Neoplasm of Connective and Other Soft Tissue; Bursa; Fascia; Fat; Ligament, except Uterine; Muscle; Peripheral, Sympathetic, and Parasympathetic Nerves and Ganglia; Synovia; Tendon (Sheath); Trunk, Unspecified; Back NOS; Flank NOS |
| 171.8 | Other Specified Sites of Connective and Other Soft Tissue; Malignant Neoplasm of Contiguous or Overlapping Sites of Connective Tissue whose Point of Origin Cannot be Determined |
| 171.9 | Malignant Neoplasm, Connective and Other Soft Tissue, Site Unspecified |
| 172 | Malignant Melanoma of Skin |
| 172.0 | Malignant Melanoma of Lip |
| 172.1 | Malignant Melanoma of Eyelid, Including Canthus |
| 172.2 | Malignant Melanoma of Ear and External Auditory Canal; Auricle (Ear); Auricular Canal, External; External [Acoustic] Meatus; Pinna |
| 172.3 | Malignant Melanoma of Other and Unspecified Parts of Face, Cheek (External), Chin, Eyebrow, Forehead, Nose (External), Temple |
| 172.4 | Malignant Melanoma of Scalp and Neck |
| 172.5 | Malignant Melanoma of Trunk, except Scrotum; Axilla, Breast, Buttock Groin, Perianal Skin, Perineum, Umbilicus |
| 172.6 | Malignant Melanoma of Upper Limb, Including Shoulder; Arm, Finger, Forearm, Hand |
| 172.7 | Malignant Melanoma of Lower Limb, Including Hip; Ankle, Foot, Heel, Knee, Leg, Popliteal Area, Thigh, Toe |
| 172.8 | Malignant Melanoma of Other Specified Sites of Skin |
| 172.9 | Melanoma of Skin, Site Unspecified |
| 173 | Other and Unspecified Malignant Neoplasm of Skin of Lip |
| 173.0 | Malignant Neoplasm of Skin, Other; Skin of Lip |
| 173.00 | Unspecified malignant neoplasm of skin of lip |
| 173.01 | Basal cell carcinoma of skin of lip |
| 173.02 | Squamous cell carcinoma of skin of lip |
| 173.09 | Other specified malignant neoplasm of skin of lip |
| 173.1 | Malignant Neoplasm of Skin, Other; Eyelid, Including Canthus |
| 173.10 | Unspecified malignant neoplasm of eyelid, including canthus |
| 173.11 | Basal cell carcinoma of eyelid, including canthus |
| 173.12 | Squamous cell carcinoma of eyelid, including canthus |
| 173.19 | Other specified malignant neoplasm of eyelid, including canthus |
| 173.2 | Malignant Neoplasm of Skin, Other; Skin of Ear and External Auditory Canal, Auricle, Auricular Canal (External), External Meatus, Pinna |
| 173.20 | Unspecified malignant neoplasm of skin of ear and external auditory canal |
| 173.21 | Basal cell carcinoma of skin of ear and external auditory canal |
| 173.22 | Squamous cell carcinoma of skin of ear and external auditory canal |
| 173.29 | Other specified malignant neoplasm of skin of ear and external auditory canal |
| 173.3 | Malignant Neoplasm of Skin, Other; Skin of Other and Unspecified Parts of Face, Cheek (External), Chin, Eyebrow, Forehead, Nose (External), Temple |
| 173.30 | Unspecified malignant neoplasm of skin of other and unspecified parts of face |
| 173.31 | Basal cell carcinoma of skin of other and unspecified parts of face |
| 173.32 | Squamous cell carcinoma of skin of other and unspecified parts of face |
| 173.39 | Other specified malignant neoplasm of skin of other and unspecified parts of face |
| 173.4 | Malignant Neoplasm of Skin, Other; Scalp and Skin of Neck |
| 173.40 | Unspecified malignant neoplasm of scalp and skin of neck |
| 173.41 | Basal cell carcinoma of scalp and skin of neck |
| 173.42 | Squamous cell carcinoma of scalp and skin of neck |
| 173.49 | Other specified malignant neoplasm of scalp and skin of neck |
| 173.5 | Malignant Neoplasm of Skin, Other; Skin of Trunk, except Scrotum; Axillary fold; Perianal Skin; Skin of Abdominal Wall, Anus, Back, Breast, Buttock, Chest Wall, Groin, Perineum; Umbilicus |
| 173.50 | Unspecified malignant neoplasm of skin of trunk, except scrotum |
| 173.51 | Basal cell carcinoma of skin of trunk, except scrotum |
| 173.52 | Squamous cell carcinoma of skin of trunk, except scrotum |
| 173.59 | Other specified malignant neoplasm of skin of trunk, except scrotum |
| 173.6 | Malignant Neoplasm of Skin, Other; Skin of Upper Limb, Including Shoulder, Arm, finger, Forearm, Hand |
| 173.60 | Unspecified malignant neoplasm of skin of upper limb, including shoulder |
| 173.61 | Basal cell carcinoma of skin of upper limb, including shoulder |
| 173.62 | Squamous cell carcinoma of skin of upper limb, including shoulder |
| 173.69 | Other specified malignant neoplasm of skin of upper limb, including shoulder |
| 173.7 | Malignant Neoplasm of Skin, Other; Skin of Lower Limb, Including Hip, Ankle, Foot, Heel, Knee, Leg, Popliteal Area, Thigh, Toe |
| 173.70 | Unspecified malignant neoplasm of skin of lower limb, including hip |
| 173.71 | Basal cell carcinoma of skin of lower limb, including hip |
| 173.72 | Squamous cell carcinoma of skin of lower limb, including hip |
| 173.79 | Other specified malignant neoplasm of skin of lower limb, including hip |
| 173.8 | Other and unspecified malignant neoplasm of other specified sites of skin; Malignant neoplasm of contiguous or overlapping sites of skin whose point of origin cannot be determined |
| 173.80 | Unspecified malignant neoplasm of other specified sites of skin |
| 173.81 | Basal cell carcinoma of other specified sites of skin |
| 173.82 | Squamous cell carcinoma of other specified sites of skin |
| 173.89 | Other specified malignant neoplasm of other specified sites of skin |
| 173.9 | Malignant Neoplasm of Skin, Other; Site Unspecified |
| 173.90 | Unspecified malignant neoplasm of skin, site unspecified |
| 173.91 | Basal cell carcinoma of skin, site unspecified |
| 173.92 | Squamous cell carcinoma of skin, site unspecified |
| 173.99 | Other specified malignant neoplasm of skin, site unspecified |
| 174 | Malignant Neoplasm of Female Breast |
| 174.0 | Malignant Neoplasm of Female Breast; Nipple and Areola |
| 174.1 | Malignant Neoplasm of Female Breast; Central Portion |
| 174.2 | Malignant Neoplasm of Female Breast; Upper-inner Quadrant |
| 174.3 | Malignant Neoplasm of Female Breast; Lower-inner Quadrant |
| 174.4 | Malignant Neoplasm of Female Breast; Upper-outer Quadrant |
| 174.5 | Malignant Neoplasm of Female Breast; Lower-outer Quadrant |
| 174.6 | Malignant Neoplasm of Female Breast; Axillary Tail |
| 174.8 | Malignant Neoplasm of Female Breast; Other Specified Sites of Female Breast, Ectopic Sites, Inner breast, Lower Breast, Midline of Breast, Outer Breast, Upper Breast |
| 174.9 | Malignant Neoplasm of Female Breast, Unspecified |
| 175 | Malignant Neoplasm of Male Breast |
| 175.0 | Malignant Neoplasm of Male Breast; Nipple and Areola |
| 175.9 | Malignant Neoplasm of Male Breast; Other and Unspecified Sites of Male Breast; Ectopic Breast Tissue, Male |
| 176 | Kaposi's Sarcoma |
| 176.0 | Kaposi's Sarcoma, Skin |
| 176.1 | Kaposi's Sarcoma, Soft Tissue |
| 176.2 | Kaposi's Sarcoma, Palate |
| 176.3 | Kaposi's Sarcoma, Gastrointestinal Sites |
| 176.4 | Kaposi's Sarcoma, Lung |
| 176.5 | Kaposi's Sarcoma, Lymph Nodes |
| 176.8 | Kaposi's Sarcoma, Other Specified Sites; Oral Cavity NEC |
| 176.9 | Kaposi's Sarcoma, Unspecified; Viscera NOS |
| 179 | Malignant Neoplasm of Uterus, Part Unspecified |
| 180 | Malignant Neoplasm of Cervix Uteri |
| 180.0 | Malignant Neoplasm of Cervix Uteri; Endocervix; Cervical Canal NOS; Endocervical Canal; Endocervical Gland |
| 180.1 | Malignant Neoplasm of Cervix Uteri; Exocervix |
| 180.8 | Neoplasm, Other Specified Sites of Cervix, Malignant |
| 180.9 | Cancer, Cervix Uteri, Unspecified Site |
| 181 | Malignant Neoplasm of Placenta; Choriocarinoma NOS; Chorioepithelioma NOS |
| 182 | Malignant Neoplasm of Body of Uterus |
| 182.0 | Malignant Neoplasm of Body of Uterus; Corpus Uteri, except Isthmus; Cornu; Endometrium; Fundus; Myometrium |
| 182.1 | Malignant Neoplasm of Body of Uterus; Isthmus, Lower Uterine Segment |
| 182.8 | Malignant Neoplasm of Body of Uterus; Other Specified Sites of Body of Uterus |
| 183 | Malignant Neoplasm of Ovary and Other Uterine Adnexa |
| 183.0 | Malignant Neoplasm of Ovary and Other Uterine Adnexa; Ovary |
| 183.2 | Malignant Neoplasm of Ovary and Other Uterine Adnexa; Fallopian tube; Oviduct; Uterine Tube |
| 183.3 | Malignant Neoplasm of Ovary and Other Uterine Adnexa; Broad Ligament; Mesovarium; Parovarian Region |
| 183.4 | Malignant Neoplasm of Ovary and Other Uterine Adnexa; Parametrium; Uterine Ligament NOS; Uterosacral Ligament |
| 183.5 | Malignant Neoplasm of Ovary and Other Uterine Adnexa; Round Ligament |
| 183.8 | Malignant Neoplasm of Ovary and Other Uterine Adnexa; Other Specified Sites of Uterine Adnexa; Tubo-ovarian; Utero-ovarian |
| 183.9 | Malignant Neoplasm of Ovary and Other Uterine Adnexa; Uterine Adnexa, Unspecified |
| 184 | Malignant Neoplasm of Other and Unspecified Female Genital Organs |
| 184.0 | Malignant Neoplasm of Other and Unspecified Female Genital Organs, Vagina; Gartner's Duct; Vaginal Vault |
| 184.1 | Malignant Neoplasm of Other and Unspecified Female Genital Organs, Labia Majora; Greater Vestibular [Bartholin's] Gland |
| 184.2 | Malignant Neoplasm of Other and Unspecified Female Genital Organs, Labia Minora |
| 184.3 | Malignant Neoplasm of Other and Unspecified Female Genital Organs, Clitoris |
| 184.4 | Malignant Neoplasm of Other and Unspecified Female Genital Organs, Vulva, Unspecified; External Female Genitalia NOS; Pudendum |
| 184.8 | Malignant Neoplasm of Other and Unspecified Female Genital Organs, Other Specified Sites of Female Genital Organs; Malignant Neoplasm of Contiguous or Overlapping Sites of Female Genital Organs whose Point of Origin Cannot be Determined |
| 184.9 | Malignant Neoplasm of Other and Unspecified Female Genital Organs; Female Genital Organ, Site Unspecified; Female Genitourinary Tract NOS |
| 185 | Malignant Neoplasm of Prostate |
| 186 | Malignant Neoplasm of Testis |
| 186.0 | Malignant Neoplasm of Testis; Undescended Testis, Malignant; Ectopic Testis; Retained Testis |
| 186.9 | Malignant Neoplasm of Testis; Other and Unspecified Testis; Testis: NOS, Descended, Scrotal |
| 187 | Malignant Neoplasm of Penis and Other Male Genital Organs |
| 187.1 | Malignant Neoplasm of Penis and Other Male Genital Organs, Prepuce; Foreskin |
| 187.2 | Malignant Neoplasm of Penis and Other Male Genital Organs, Glans Penis |
| 187.3 | Malignant Neoplasm of Penis and Other Male Genital Organs, Body of Penis; Corpus cavernosum |
| 187.4 | Malignant Neoplasm of Penis and Other Male Genital Organs, Penis, Part Unspecified; Skin of Penis NOS |
| 187.5 | Malignant Neoplasm of Penis and Other Male Genital Organs, Epididymis |
| 187.6 | Malignant Neoplasm of Penis and Other Male Genital Organs, Spermatic Cord; Vas Deferens |
| 187.7 | Malignant Neoplasm of Penis and Other Male Genital Organs, Scrotum; Skin of Scrotum |
| 187.8 | Malignant Neoplasm of Penis and Other Male Genital Organs, Other Specified Sites of Male Genital Organs; Seminal Vesicle; Tunica Vaginalis |
| 187.9 | Malignant Neoplasm of Penis and Other Male Genital Organs; Male Genital Organ, Site Unspecified; Male Genital Organ or Tract NOS |
| 188 | Neoplasm, Bladder, Malignant |
| 188.0 | Neoplasm, Trigone of Urinary Bladder, Malignant |
| 188.1 | Neoplasm, Dome of Urinary Bladder, Malignant |
| 188.2 | Neoplasm, Lateral Wall of Urinary Bladder, Malignant |
| 188.3 | Neoplasm, Anterior Wall of Urinary Bladder, Malignant |
| 188.4 | Neoplasm, Posterior Wall of Urinary Bladder, Malignant |
| 188.5 | Malignant Neoplasm of Bladder; Bladder Neck, Internal Urethral Orifice |
| 188.6 | Neoplasm, Ureteric Orifice, Malignant |
| 188.7 | Neoplasm, Urachus, Malignant |
| 188.8 | Neoplasm, Other Specified Sites of Bladder, Malignant |
| 188.9 | Malignant Neoplasm of Bladder, Part Unspecified; Bladder Wall NOS |
| 189 | Malignant Neoplasm of Kidney and Other and Unspecified Urinary Organs |
| 189.0 | Malignant Neoplasm of Kidney and Other and Unspecified Urinary Organs; Kidney, except Pelvis; Kidney NOS; Kidney Parenchyma |
| 189.1 | Malignant Neoplasm of Kidney and Other and Unspecified Urinary Organs; Renal Pelvis; Renal Calyces; Ureteropelvic Junction |
| 189.2 | Malignant Neoplasm of Kidney and Other and Unspecified Urinary Organs; Ureter |
| 189.3 | Malignant Neoplasm of Kidney and Other and Unspecified Urinary Organs; Urethra |
| 189.4 | Malignant Neoplasm of Kidney and Other and Unspecified Urinary Organs; Paraurethral Glands |
| 189.8 | Malignant Neoplasm of Kidney and Other and Unspecified Urinary Organs; Other Specified Sites of Urinary Organs |
| 189.9 | Malignant Neoplasm of Kidney and Other and Unspecified Urinary Organs Urinary Organ, Site Unspecified; Urinary System NOS |
| 190 | Malignant Neoplasm of Eye |
| 190.0 | Malignant Neoplasm of Eye, Eyeball, except Conjunctiva, Cornea, Retina, and Choroid; Ciliary Body; Crystalline Lens; Iris; Sclera; Uveal Tract |
| 190.1 | Malignant Neoplasm of Eye, Orbit; Connective Tissue of Orbit; Extraocular Muscle; Retrobulbar |
| 190.2 | Malignant Neoplasm of Eye, Lacrimal Gland |
| 190.3 | Malignant Neoplasm of Eye, Conjunctiva |
| 190.4 | Malignant Neoplasm of Eye, Cornea |
| 190.5 | Malignant Neoplasm of Eye, Retina |
| 190.6 | Malignant Neoplasm of Eye, Choroid |
| 190.7 | Malignant Neoplasm of Eye, Lacrimal Duct; Lacrimal Sac; Nasolacrimal Duct |
| 190.8 | Malignant Neoplasm of Eye, Other Specified Sites of Eye; Malignant Neoplasm of Contiguous or Overlapping Sites of Eye whose Point of Origin Cannot be Determined |
| 190.9 | Malignant Neoplasm of Eye, Eye, Part Unspecified |
| 191 | Malignant Neoplasm of Brain |
| 191.0 | Malignant Neoplasm of Brain; Cerebrum, except Lobes and Ventricles; Basal Ganglia; Cerebral Cortex; Corpus Striatum; Globus Pallidus; Hypothalamus; Thalamus |
| 191.1 | Malignant Neoplasm of Brain; Frontal Lobe |
| 191.2 | Malignant Neoplasm of Brain; Temporal Lobe, Hippocampus, Uncus |
| 191.3 | Malignant Neoplasm of Brain; Parietal Lobe |
| 191.4 | Malignant Neoplasm of Brain; Occipital Lobe |
| 191.5 | Malignant Neoplasm of Brain; Ventricles, Choroid Plexus, Floor of Ventricle |
| 191.6 | Malignant Neoplasm of Brain; Cerebellum NOS, Cerebellopontine Angle |
| 191.7 | Malignant Neoplasm of Brain; Brain Stem; Cerebral Peduncle; Medulla Oblongata; Midbrain; Pons |
| 191.8 | Malignant Neoplasm of Brain; Other Parts of Brain; Corpus Callosum; Tapetum |
| 191.9 | Brain, Unspecified; Cranial Fossa NOS |
| 192 | Neoplasm, Other and Unspecified Parts of Nervous System, Malignant |
| 192.0 | Neoplasm, Other and Unspecified Parts of Nervous System, Malignant, Cranial Nerves; Olfactory Bulb |
| 192.1 | Neoplasm, Other and Unspecified Parts of Nervous System, Malignant, Meningioma |
| 192.2 | Neoplasm, Other and Unspecified Parts of Nervous System, Malignant, Spinal Cord; Cauda Equina |
| 192.3 | Neoplasm, Other and Unspecified Parts of Nervous System, Malignant, Spinal Meninges |
| 192.8 | Neoplasm, Other Specified Sites of Nervous System, Malignant |
| 192.9 | Neoplasm, Nervous System, Malignant, Part Unspecified |
| 193 | Cancer, Thyroid Gland |
| 194 | Malignant Neoplasm of Other Endocrine Glands and Related Structures, Other Endocrine Glands and Related Structures |
| 194.0 | Malignant Neoplasm of Other Endocrine Glands and Related Structures, Adrenal Gland; Adrenal Cortex; Adrenal Medulla; Suprarenal Gland |
| 194.1 | Malignant Neoplasm of other Endocrine Glands and Related Structures, Parathyroid Gland |
| 194.3 | Malignant Neoplasm of Other Endocrine Glands and Related Structures, Pituitary Gland and Craniopharyngeal Duct; Craniobuccal Pouch; Hypophysis; Rathke's Pouch; Sella Turcica |
| 194.4 | Malignant Neoplasm of other Endocrine Glands and Related Structures, Pineal Gland, Malignant |
| 194.5 | Malignant Neoplasm of other Endocrine Glands and Related Structures, Carotid Body |
| 194.6 | Malignant Neoplasm of other Endocrine Glands and Related Structures, Aortic Body and Other Paraganglia; Coccygeal Body; Glomus Jugulare; Para-aortic Body |
| 194.8 | Malignant Neoplasm of other Endocrine Glands and Related Structures, Other |
| 194.9 | Malignant Neoplasm of Other Endocrine Glands and Related Structures, Endocrine Gland, Site Unspecified |
| 195 | Malignant Neoplasm of Other and Ill-defined Sites, Head, Face, and Neck |
| 195.0 | Malignant Neoplasm of Other and Ill-defined Sites, Head, Face, and Neck; Cheek NOS, Jaw NOS, Nose NOS, Supraclavicular Region NOS |
| 195.1 | Malignant Neoplasm of Other and Ill-defined Sites, Thorax; Axilla; Chest (Wall) NOS; Intrathoracic Nos |
| 195.2 | Malignant Neoplasm of Other and Ill-defined Sites, Abdomen; Intra-abdominal NOS |
| 195.3 | Malignant Neoplasm of Other and Ill-defined Sites, Pelvis; Groin; Presacral Region; Sacrococcygeal Region; Rectovaginal (Septum); Rectovesical (Septum) |
| 195.4 | Malignant Neoplasm of Other and Ill-defined Sites, Upper Limb |
| 195.5 | Malignant Neoplasm of Other and Ill-defined Sites, Lower Limb |
| 195.8 | Malignant Neoplasm of Other and Ill-defined sites, Other Specified Sites |
| 196 | Secondary and Unspecified Malignant Neoplasm of Lymph Nodes |
| 196.0 | Lymph Nodes of Head, Face, and Neck; Cervical; Cervicofacial; Scalene; Supraclavicular |
| 196.1 | Intrathoracic Lymph Nodes; Bronchopulmonary; Intercostal; Mediastinal; Tracheobronchial |
| 196.2 | Intra-abdominal Lymph Nodes; Intestinal; Mesenteric; Retroperitoneal |
| 196.3 | Lymph Nodes of Axilla and Upper Limb; Brachial; Epitrochlear; Infraclavicular; Pectoral |
| 196.5 | Lymph Nodes of Inguinal Region and Lower Limb; Femoral; Groin; Popliteal; Tibial |
| 196.6 | Intrapelvic Lymph Nodes; Hypogastric; Iliac; Obturator; Parametrial |
| 196.8 | Lymph Nodes of Multiple Sites |
| 196.9 | Secondary and Unspecified Malignant Neoplasm of Lymph Nodes; Site Unspecified; Lymph Nodes NOS |
| 197 | Secondary Malignant Neoplasm of Respiratory and Digestive Systems |
| 197.0 | Secondary Malignant Neoplasm of Respiratory and Digestive Systems; Lung, Bronchus |
| 197.1 | Secondary Malignant Neoplasm of Respiratory and Digestive Systems; Mediastinum |
| 197.2 | Secondary Malignant Neoplasm of Respiratory and Digestive Systems; Pleura |
| 197.3 | Secondary Malignant Neoplasm of Respiratory and Digestive Systems; Other Respiratory Organs; Trachea |
| 197.4 | Secondary Malignant Neoplasm of Respiratory and Digestive Systems; Small Intestine, Including Duodenum |
| 197.5 | Secondary Malignant Neoplasm of Respiratory and Digestive Systems; Large Intestine and Rectum |
| 197.6 | Secondary Malignant Neoplasm of Respiratory and Digestive Systems; Retroperitoneum and Peritoneum |
| 197.7 | Secondary Malignant Neoplasm of Respiratory and Digestive Systems; Liver, Specified as Secondary |
| 197.8 | Secondary Malignant Neoplasm of Respiratory and Digestive Systems; Other Digestive Organs and Spleen |
| 198 | Secondary Malignant Neoplasm of Other Specified Sites |
| 198.0 | Secondary Malignant Neoplasm of Other Specified Sites; Kidney |
| 198.1 | Secondary Malignant Neoplasm of Other Specified Sites; Other Urinary Organs |
| 198.2 | Secondary Malignant Neoplasm of Other Specified Sites; Skin; Skin Of Breast |
| 198.3 | Secondary Malignant Neoplasm of other Specified Sites; Brain and Spinal Cord |
| 198.4 | Secondary Malignant Neoplasm of Other Specified Sites; Other Parts of Nervous System; Meninges |
| 198.5 | Secondary Malignant Neoplasm of Other Specified Sites; Bone and Bone Marrow |
| 198.6 | Secondary Malignant Neoplasm of Other Specified Sites; Ovary |
| 198.7 | Secondary Malignant Neoplasm of Other Specified sites; Adrenal Gland; Suprarenal gland |
| 198.8 | Secondary malignant neoplasm of Other Specified Sites; Other Specified Sites |
| 198.81 | Secondary Malignant Neoplasm of Other Specified Sites; Other Specified Sites, Breast |
| 198.82 | Secondary Malignant Neoplasm of Other Specified Sites; Other Specified Sites, Genital Organs |
| 198.89 | Secondary Malignant Neoplasm of Other Specified Sites; Other Specified Sites, Other |
| 199 | Malignant Neoplasm without Specification of Site |
| 199.0 | Malignant Neoplasm without Specification of Site; Disseminated; Carcinomatosis Unspecified Site (Primary) (Secondary); Cancer Unspecified Site (Primary) (Secondary); Malignancy Unspecified Site (Primary) (Secondary); Multiple Cancer Unspecified Site (Primary) (Secondary) |
| 199.1 | Malignant Neoplasm without Specification of Site; Other; Cancer Unspecified Site (Primary) (Secondary); Carcinoma Unspecified Site (Primary) (Secondary); Malignancy Unspecified Site (Primary) (Secondary) |
| 199.2 | Malignant neoplasm associated with transplant organ |
| 200 | Lymphosarcoma and Reticulosarcoma, Other Specified Malignant Tumors of Lymphatic Tissue |
| 200.0 | Reticulosarcoma; Nodular; Pleomorphic Cell Type; Reticulum Cell Type; Pleomorphic Cell Type |
| 200.00 | Reticulosarcoma; Nodular; Pleomorphic Cell Type; Reticulum Cell Type; Pleomorphic Cell Type; Unspecified Site, Extranodal and Solid Organ Sites |
| 200.01 | Reticulosarcoma; Nodular; Pleomorphic Cell Type; Reticulum Cell Type; Pleomorphic Cell Type; Lymph Nodes of Head, Face, and Neck |
| 200.02 | Reticulosarcoma; Nodular; Pleomorphic Cell Type; Reticulum Cell Type; Pleomorphic Cell Type; Intrathoracic Lymph Nodes |
| 200.03 | Reticulosarcoma; Nodular; Pleomorphic Cell Type; Reticulum Cell Type; Pleomorphic Cell Type; Intra-abdominal Lymph Nodes |
| 200.04 | Reticulosarcoma; Nodular; Pleomorphic Cell Type; Reticulum Cell Type; Pleomorphic Cell Type; Lymph Nodes of Axilla and Upper Limb |
| 200.05 | Reticulosarcoma; Nodular; Pleomorphic Cell Type; Reticulum Cell Type; Pleomorphic Cell Type; Lymph Nodes of Inguinal Region and Lower Limb |
| 200.06 | Reticulosarcoma; Nodular; Pleomorphic Cell Type; Reticulum Cell Type; Pleomorphic Cell Type; Intrapelvic Lymph Nodes |
| 200.07 | Reticulosarcoma; Nodular; Pleomorphic Cell Type; Reticulum Cell Type; Pleomorphic Cell Type; Spleen |
| 200.08 | Reticulosarcoma; Nodular; Pleomorphic Cell Type; Reticulum Cell Type; Pleomorphic Cell Type; Lymph Nodes of Multiple Sites |
| 200.1 | Lymphosarcoma; Hodgkin's Disease |
| 200.10 | Lymphosarcoma; Lymphoblastoma (Diffuse); Lymphoma (Malignant); Lymphosarcoma NOS; Unspecified Site, Extranodal and Solid Organ Sites |
| 200.11 | Lymphosarcoma; Lymphoblastoma (Diffuse); Lymphoma (Malignant); Lymphosarcoma NOS; Lymph Nodes of Head, Face, and Neck |
| 200.12 | Lymphosarcoma; Lymphoblastoma (Diffuse); Lymphoma (Malignant); Lymphosarcoma NOS; Intrathoracic Lymph Nodes |
| 200.13 | Lymphosarcoma; Lymphoblastoma (Diffuse); Lymphoma (Malignant); Lymphosarcoma NOS; Intra-Abdominal Lymph Nodes |
| 200.14 | Lymphosarcoma; Lymphoblastoma (Diffuse); Lymphoma (Malignant); Lymphosarcoma NOS; Lymph Nodes of Axilla and Upper Limb |
| 200.15 | Lymphosarcoma; Lymphoblastoma (Diffuse); Lymphoma (Malignant); Lymphosarcoma NOS; Lymph Nodes of Inguinal Region and Lower Limb |
| 200.16 | Lymphosarcoma; Lymphoblastoma (Diffuse); Lymphoma (Malignant); Lymphosarcoma NOS; Intrapelvic Lymph Nodes |
| 200.17 | Lymphosarcoma; Lymphoblastoma (Diffuse); Lymphoma (Malignant); Lymphosarcoma NOS; Spleen |
| 200.18 | Lymphosarcoma; Lymphoblastoma (Diffuse); Lymphoma (Malignant); Lymphosarcoma NOS; Lymph Nodes of Multiple Sites |
| 200.2 | Burkitt's Tumor or Lymphoma; Malignant Lymphoma Burkitt's Type |
| 200.20 | Burkitt's Tumor or Lymphoma; Malignant Lymphoma Burkitt's Type; Unspecified Site, Extranodal and Solid Organ Sites |
| 200.21 | Burkitt's Tumor or Lymphoma; Malignant Lymphoma Burkitt's Type; Lymph Nodes of Head, Face, and Neck |
| 200.22 | Burkitt's Tumor or Lymphoma; Malignant Lymphoma Burkitt's Type; Intrathoracic Lymph Nodes |
| 200.23 | Burkitt's Tumor or Lymphoma; Malignant Lymphoma Burkitt's Type; Intra-Abdominal Lymph Nodes |
| 200.24 | Burkitt's Tumor or Lymphoma; Malignant Lymphoma Burkitt's Type; Lymph Nodes of Axilla and Upper Limb |
| 200.25 | Burkitt's Tumor or Lymphoma; Malignant Lymphoma Burkitt's Type; Lymph Nodes of Inguinal Region and Lower Limb |
| 200.26 | Burkitt's Tumor or Lymphoma; Malignant Lymphoma Burkitt's Type; Intrapelvic Lymph Nodes |
| 200.27 | Burkitt's Tumor or Lymphoma; Malignant Lymphoma Burkitt's Type; Spleen |
| 200.28 | Burkitt's Tumor or Lymphoma; Malignant Lymphoma Burkitt's Type; Lymph Nodes of Multiple Sites |
| 200.3 | Marginal Zone Lymphoma; Extranodal Marginal Zone B Cell Lymphoma, Mucosa Associated Lymphoid Tissue, Nodal Marginal Zone B Cell Lymphoma, Splenic Marginal Zone B Cell Lymphoma |
| 200.30 | Marginal Zone Lymphoma; Extranodal Marginal Zone B Cell Lymphoma, Mucosa Associated Lymphoid Tissue, Nodal Marginal Zone B Cell Lymphoma, Splenic Marginal Zone B Cell Lymphoma; Unspecified Site, Extranodal and Solid Organ Sites |
| 200.31 | Marginal Zone Lymphoma; Extranodal Marginal Zone B Cell Lymphoma, Mucosa Associated Lymphoid Tissue, Nodal Marginal Zone B Cell Lymphoma, Splenic Marginal Zone B Cell Lymphoma; Lymph Nodes of Head, Face, and Neck |
| 200.32 | Marginal Zone Lymphoma; Extranodal Marginal Zone B Cell Lymphoma, Mucosa Associated Lymphoid Tissue, Nodal Marginal Zone B Cell Lymphoma, Splenic Marginal Zone B Cell Lymphoma; Intrathoracic Lymph Nodes |
| 200.33 | Marginal Zone Lymphoma; Extranodal Marginal Zone B Cell Lymphoma, Mucosa Associated Lymphoid Tissue, Nodal Marginal Zone B Cell Lymphoma, Splenic Marginal Zone B Cell Lymphoma; Intra-Abdominal Lymph Nodes |
| 200.34 | Marginal Zone Lymphoma; Extranodal Marginal Zone B Cell Lymphoma, Mucosa Associated Lymphoid Tissue, Nodal Marginal Zone B Cell Lymphoma, Splenic Marginal Zone B Cell Lymphoma; Lymph Nodes of Axilla and Upper Limb |
| 200.35 | Marginal Zone Lymphoma; Extranodal Marginal Zone B Cell Lymphoma, Mucosa Associated Lymphoid Tissue, Nodal Marginal Zone B Cell Lymphoma, Splenic Marginal Zone B Cell Lymphoma; Lymph Nodes of Inguinal Region and Lower Limb |
| 200.36 | Marginal Zone Lymphoma; Extranodal Marginal Zone B Cell Lymphoma, Mucosa Associated Lymphoid Tissue, Nodal Marginal Zone B Cell Lymphoma, Splenic Marginal Zone B Cell Lymphoma; Intrapelvic Lymph Nodes |
| 200.37 | Marginal Zone Lymphoma; Extranodal Marginal Zone B Cell Lymphoma, Mucosa Associated Lymphoid Tissue, Nodal Marginal Zone B Cell Lymphoma, Splenic Marginal Zone B Cell Lymphoma; Spleen |
| 200.38 | Marginal Zone Lymphoma; Extranodal Marginal Zone B Cell Lymphoma, Mucosa Associated Lymphoid Tissue, Nodal Marginal Zone B Cell Lymphoma, Splenic Marginal Zone B Cell Lymphoma; Lymph Nodes of Multiple Sites |
| 200.4 | Mantle Cell Lymphoma |
| 200.40 | Mantle Cell Lymphoma; Unspecified Site, Extranodal and Solid Organ Sites |
| 200.41 | Mantle Cell Lymphoma; Lymph Nodes of Head, Face, and Neck |
| 200.42 | Mantle Cell Lymphoma; Intrathoracic Lymph Nodes |
| 200.43 | Mantle Cell Lymphoma; Intra-abdominal Lymph Nodes |
| 200.44 | Mantle Cell Lymphoma; Lymph Nodes of Axilla and Upper Limb |
| 200.45 | Mantle Cell Lymphoma; Lymph Nodes of Inguinal Region and Lower Limb |
| 200.46 | Mantle Cell Lymphoma; Intrapelvic Lymph Nodes |
| 200.47 | Mantle Cell Lymphoma; Spleen |
| 200.48 | Mantle Cell Lymphoma; Lymph Nodes of Multiple Sites |
| 200.5 | Primary Central Nervous System Lymphoma |
| 200.50 | Primary Central Nervous System Lymphoma; Unspecified Site, Extranodal and Solid Organ Sites |
| 200.51 | Primary Central Nervous System Lymphoma; Lymph Nodes of Head, Face, and Neck |
| 200.52 | Primary Central Nervous System Lymphoma; Intrathoracic Lymph Nodes |
| 200.53 | Primary Central Nervous System Lymphoma; Intra-abdominal Lymph Nodes |
| 200.54 | Primary Central Nervous System Lymphoma; Lymph Nodes of Axilla and Upper Limb |
| 200.55 | Primary Central Nervous System Lymphoma; Lymph Nodes of Inguinal Region and Lower Limb |
| 200.56 | Primary Central Nervous System Lymphoma; Intrapelvic Lymph Nodes |
| 200.57 | Primary Central Nervous System Lymphoma; Spleen |
| 200.58 | Primary Central Nervous System Lymphoma; Lymph Nodes of Multiple Sites |
| 200.6 | Anaplastic Large Cell Lymphoma |
| 200.60 | Anaplastic Large Cell Lymphoma; Unspecified Site, Extranodal and Solid Organ Sites |
| 200.61 | Anaplastic Large Cell Lymphoma; Lymph Nodes of Head, Face, and Neck |
| 200.62 | Anaplastic Large Cell Lymphoma; Intrathoracic Lymph Nodes |
| 200.63 | Anaplastic Large Cell Lymphoma; Intra-abdominal Lymph Nodes |
| 200.64 | Anaplastic Large Cell Lymphoma; Lymph Nodes of Axilla and Upper Limb |
| 200.65 | Anaplastic Large Cell Lymphoma; Lymph Nodes of Inguinal Region and Lower Limb |
| 200.66 | Anaplastic Large Cell Lymphoma; Intrapelvic Lymph Nodes |
| 200.67 | Anaplastic Large Cell Lymphoma; Spleen |
| 200.68 | Anaplastic Large Cell Lymphoma; Lymph Nodes of Multiple Sites |
| 200.7 | Large Cell Lymphoma |
| 200.70 | Large Cell Lymphoma; Unspecified Site, Extranodal and Solid Organ Sites |
| 200.71 | Large Cell Lymphoma; Lymph Nodes of Head, Face, and Neck |
| 200.72 | Large Cell Lymphoma; Intrathoracic Lymph Nodes |
| 200.73 | Large Cell Lymphoma; Intra-Abdominal Lymph Nodes |
| 200.74 | Large Cell Lymphoma; Lymph Nodes of Axilla and Upper Limb |
| 200.75 | Large Cell Lymphoma; Lymph Nodes of Inguinal Region and Lower Limb |
| 200.76 | Large Cell Lymphoma; Intrapelvic Lymph Nodes |
| 200.77 | Large Cell Lymphoma; Spleen |
| 200.78 | Large Cell Lymphoma; Lymph Nodes of Multiple Sites |
| 200.8 | Lymphoma, Other Named Variants; Lymphoma (Malignant): Lymphoplasmacytoid Type, Mixed Lymphocytic-histiocytic (Diffuse); Lymphosarcoma, Mixed Cell Type (Diffuse); Reticulolymphosarcoma (Diffuse) |
| 200.80 | Lymphoma, Other Named Variants; Lymphoma (Malignant): Lymphoplasmacytoid Type, Mixed Lymphocytic-histiocytic (Diffuse); Lymphosarcoma, Mixed Cell Type (Diffuse); Reticulolymphosarcoma (Diffuse); Unspecified Site, Extranodal and Solid Organ Sites |
| 200.81 | Lymphoma, Other Named Variants; Lymphoma (Malignant): Lymphoplasmacytoid Type, Mixed Lymphocytic-histiocytic (Diffuse); Lymphosarcoma, Mixed Cell Type (Diffuse); Reticulolymphosarcoma (Diffuse); Lymph Nodes of Head, Face, and Neck |
| 200.82 | Lymphoma, Other Named Variants; Lymphoma (Malignant): Lymphoplasmacytoid Type, Mixed Lymphocytic-histiocytic (Diffuse); Lymphosarcoma, Mixed Cell Type (Diffuse); Reticulolymphosarcoma (Diffuse); Intrathoracic Lymph Nodes |
| 200.83 | Lymphoma, Other Named Variants; Lymphoma (Malignant): Lymphoplasmacytoid Type, Mixed Lymphocytic-histiocytic (Diffuse); Lymphosarcoma, Mixed Cell Type (Diffuse); Reticulolymphosarcoma (Diffuse); Intra-Abdominal Lymph Nodes |
| 200.84 | Lymphoma, Other Named Variants; Lymphoma (Malignant): Lymphoplasmacytoid Type, Mixed Lymphocytic-histiocytic (Diffuse); Lymphosarcoma, Mixed Cell Type (Diffuse); Reticulolymphosarcoma (Diffuse); Lymph Nodes of Axilla and Upper Limb |
| 200.85 | Lymphoma, Other Named Variants; Lymphoma (Malignant): Lymphoplasmacytoid Type, Mixed Lymphocytic-histiocytic (Diffuse); Lymphosarcoma, Mixed Cell Type (Diffuse); Reticulolymphosarcoma (Diffuse); Lymph Nodes of Inguinal Region and Lower Limb |
| 200.86 | Lymphoma, Other Named Variants; Lymphoma (Malignant): Lymphoplasmacytoid Type, Mixed Lymphocytic-histiocytic (Diffuse); Lymphosarcoma, Mixed Cell Type (Diffuse); Reticulolymphosarcoma (Diffuse); Intrapelvic Lymph Nodes |
| 200.87 | Lymphoma, Other Named Variants; Lymphoma (Malignant): Lymphoplasmacytoid Type, Mixed Lymphocytic-histiocytic (Diffuse); Lymphosarcoma, Mixed Cell Type (Diffuse); Reticulolymphosarcoma (Diffuse); Spleen |
| 200.88 | Lymphoma, Other Named Variants; Lymphoma (Malignant): Lymphoplasmacytoid Type, Mixed Lymphocytic-histiocytic (Diffuse); Lymphosarcoma, Mixed Cell Type (Diffuse); Reticulolymphosarcoma (Diffuse); Lymph Nodes of Multiple Sites |
| 201 | Hodgkin's Disease; Lymphoma |
| 201.0 | Hodgkin's Paragranuloma |
| 201.00 | Hodgkin's Paragranuloma, Unspecified Site |
| 201.01 | Hodgkin's Paragranuloma; Lymph Nodes of Head, Face, and Neck |
| 201.02 | Hodgkin's Paragranuloma; Intrathoracic Lymph Nodes |
| 201.03 | Hodgkin's Paragranuloma; Intra-abdominal Lymph Nodes |
| 201.04 | Hodgkin's Paragranuloma; Lymph Nodes of Axilla and Upper Limb |
| 201.05 | Hodgkin's Paragranuloma; Lymph Nodes of Inguinal Region and Lower Limb |
| 201.06 | Hodgkin's Paragranuloma; Intrapelvic Lymph Nodes |
| 201.07 | Hodgkin's Paragranuloma; Spleen |
| 201.08 | Hodgkin's Paragranuloma, Lymph Nodes of Multiple Sites |
| 201.1 | Hodgkin's Granuloma |
| 201.10 | Hodgkin's Granuloma, Unspecified Site |
| 201.11 | Hodgkin's Granuloma; Lymph Nodes of Head, Face, and Neck |
| 201.12 | Hodgkin's Granuloma; Intrathoracic Lymph Nodes |
| 201.13 | Hodgkin's Granuloma; Intra-Abdominal Lymph Nodes |
| 201.14 | Hodgkin's Granuloma; Lymph Nodes of Axilla and Upper Limb |
| 201.15 | Hodgkin's Granuloma; Lymph Nodes of Inguinal Region and Lower Limb |
| 201.16 | Hodgkin's Granuloma; Intrapelvic Lymph Nodes |
| 201.17 | Hodgkin's Granuloma; Spleen |
| 201.18 | Hodgkin's Granuloma, Lymph Nodes of Multiple Sites |
| 201.2 | Hodgkin's Sarcoma |
| 201.20 | Hodgkin's Sarcoma, Unspecified Site |
| 201.21 | Hodgkin's Sarcoma; Lymph Nodes of Head, Face, and Neck |
| 201.22 | Hodgkin's Sarcoma; Intrathoracic Lymph Nodes |
| 201.23 | Hodgkin's Sarcoma; Intra-Abdominal Lymph Nodes |
| 201.24 | Hodgkin's Sarcoma; Lymph Nodes of Axilla and Upper Limb |
| 201.25 | Hodgkin's Sarcoma; Lymph Nodes of Inguinal Region and Lower Limb |
| 201.26 | Hodgkin's Sarcoma; Intrapelvic Lymph Nodes |
| 201.27 | Hodgkin's Sarcoma; Spleen |
| 201.28 | Hodgkin's Sarcoma, Lymph Nodes of Multiple Sites |
| 201.4 | Hodgkin's Disease, Lymphocytic-histiocytic Predominance |
| 201.40 | Hodgkin's Disease, Lymphocytic-histiocytic Predominance, Unspecified Site |
| 201.41 | Hodgkin's Disease, Lymphocytic-histiocytic Predominance; Lymph Nodes of Head, Face, and Neck |
| 201.42 | Hodgkin's Disease, Lymphocytic-histiocytic Predominance; Intrathoracic Lymph Nodes |
| 201.43 | Hodgkin's Disease, Lymphocytic-histiocytic Predominance; Intra-Abdominal Lymph Nodes |
| 201.44 | Hodgkin's Disease, Lymphocytic-histiocytic Predominance; Lymph Nodes of Axilla and Upper Limb |
| 201.45 | Hodgkin's Disease, Lymphocytic-histiocytic Predominance; Lymph Nodes of Inguinal Region and Lower Limb |
| 201.46 | Hodgkin's Disease, Lymphocytic-histiocytic Predominance; Intrapelvic Lymph Nodes |
| 201.47 | Hodgkin's Disease, Lymphocytic-histiocytic Predominance; Spleen |
| 201.48 | Hodgkin's Disease, Lymphocytic-histiocytic Predominance, Lymph Nodes of Multiple Sites |
| 201.5 | Hodgkin's Disease, Nodular Sclerosis |
| 201.50 | Hodgkin's Disease, Nodular Sclerosis, Unspecified Site |
| 201.51 | Lymph Nodes of Head, Face, and Neck |
| 201.52 | Hodgkin's Disease, Nodular Sclerosis; NOS, Cellular Phase; Intrathoracic Lymph Nodes |
| 201.53 | Hodgkin's Disease, Nodular Sclerosis; NOS, Cellular Phase; Intra-Abdominal Lymph Nodes |
| 201.54 | Hodgkin's Disease, Nodular Sclerosis; NOS, Cellular Phase; Lymph Nodes of Axilla and Upper Limb |
| 201.55 | Hodgkin's Disease, Nodular Sclerosis; NOS, Cellular Phase; Lymph Nodes of Inguinal Region and Lower Limb |
| 201.56 | Hodgkin's Disease, Nodular Sclerosis; NOS, Cellular Phase; Intrapelvic Lymph Nodes |
| 201.57 | Hodgkin's Disease, Nodular Sclerosis; NOS, Cellular Phase; Spleen |
| 201.58 | Hodgkin's Disease, Nodular Sclerosis, Lymph Nodes of Multiple Sites |
| 201.6 | Hodgkin's Disease, Mixed Cellularity |
| 201.60 | Hodgkin's Disease, Mixed Cellularity, Unspecified Site |
| 201.61 | Hodgkin's Disease, Mixed Cellularity; Lymph Nodes of Head, Face, and Neck |
| 201.62 | Hodgkin's Disease, Mixed Cellularity; Intrathoracic Lymph Nodes |
| 201.63 | Hodgkin's Disease, Mixed Cellularity; Intra-Abdominal Lymph Nodes |
| 201.64 | Hodgkin's Disease, Mixed Cellularity; Lymph Nodes of Axilla and Upper Limb |
| 201.65 | Hodgkin's Disease, Mixed Cellularity; Lymph Nodes of Inguinal Region and Lower Limb |
| 201.66 | Hodgkin's Disease, Mixed Cellularity; Intrapelvic Lymph Nodes |
| 201.67 | Hodgkin's Disease, Mixed Cellularity; Spleen |
| 201.68 | Hodgkin's Disease, Mixed Cellularity, Lymph Nodes of Multiple Sites |
| 201.7 | Hodgkin's Disease, Lymphocytic Depletion |
| 201.70 | Hodgkin's Disease, Lymphocytic Depletion, Unspecified Site |
| 201.71 | Hodgkin's Disease, Lymphocytic Depletion; NOS, Diffuse Fibrosis, Reticular Type; Lymph Nodes of Head, Face, and Neck |
| 201.72 | Hodgkin's Disease, Lymphocytic Depletion; NOS, Diffuse Fibrosis, Reticular Type; Intrathoracic Lymph Nodes |
| 201.73 | Hodgkin's Disease, Lymphocytic Depletion; NOS, Diffuse Fibrosis, Reticular Type; Intra-Abdominal Lymph Nodes |
| 201.74 | Lymph Nodes of Axilla and Upper Limb |
| 201.75 | Hodgkin's Disease, Lymphocytic Depletion; NOS, Diffuse Fibrosis, Reticular Type; Lymph Nodes of Inguinal Region and Lower Limb |
| 201.76 | Hodgkin's Disease, Lymphocytic Depletion; NOS, Diffuse Fibrosis, Reticular Type; Intrapelvic Lymph Nodes |
| 201.77 | Hodgkin's Disease, Lymphocytic Depletion; NOS, Diffuse Fibrosis, Reticular Type; Spleen |
| 201.78 | Hodgkin's Disease, Lymphocytic Depletion, Lymph Nodes of Multiple Sites |
| 201.9 | Hodgkin's Disease, Unspecified |
| 201.90 | Hodgkin's Disease, Unspecified, Unspecified Site |
| 201.91 | Hodgkin's Disease, Unspecified; NOS, Lymphoma NOS; Malignant: Lymphogranuloma, Lymphogranulomatosis; Lymph Nodes of Head, Face, and Neck |
| 201.92 | Hodgkin's Disease, Unspecified; NOS, Lymphoma NOS; Malignant: Lymphogranuloma, Lymphogranulomatosis; Intrathoracic Lymph Nodes |
| 201.93 | Hodgkin's Disease, Unspecified; NOS, Lymphoma NOS; Malignant: Lymphogranuloma, Lymphogranulomatosis; Intra-Abdominal Lymph Nodes |
| 201.94 | Hodgkin's Disease, Unspecified; NOS, Lymphoma NOS; Malignant: Lymphogranuloma, Lymphogranulomatosis; Lymph Nodes of Axilla and Upper Limb |
| 201.95 | Hodgkin's Disease, Unspecified; NOS, Lymphoma NOS; Malignant: Lymphogranuloma, Lymphogranulomatosis; Lymph Nodes of Inguinal Region and Lower Limb |
| 201.96 | Hodgkin's Disease, Unspecified; NOS, Lymphoma NOS; Malignant: Lymphogranuloma, Lymphogranulomatosis; Intrapelvic Lymph Nodes |
| 201.97 | Hodgkin's Disease, Unspecified; NOS, Lymphoma NOS; Malignant: Lymphogranuloma, Lymphogranulomatosis; Spleen |
| 201.98 | Hodgkin's Disease, Unspecified, Lymph Nodes of Multiple Sites |
| 202 | Neoplasms of Lymphoid and Histiocytic Tissue, Malignant |
| 202.0 | Nodular Lymphoma; Brill-Symmers Disease; Follicular (Giant)(Large cell); Lymphocytic, Nodular; Follicular (Giant)(Large cell); Nodular; Reticulosarcoma, Follicular or Nodular |
| 202.00 | Nodular Lymphoma; Brill-Symmers Disease; Follicular (Giant); Lymphocytic, Nodular; Follicular (Giant); Nodular; Reticulosarcoma, Follicular or Nodular; Unspecified Site, Extranodal and Solid Organ Sites |
| 202.01 | Nodular Lymphoma; Brill-Symmers Disease; Follicular (Giant); Lymphocytic, Nodular; Follicular (Giant); Nodular; Reticulosarcoma, Follicular or Nodular; Lymph Nodes of Head, Face, and Neck |
| 202.02 | Nodular Lymphoma; Brill-Symmers Disease; Follicular (Giant); Lymphocytic, Nodular; Follicular (Giant); Nodular; Reticulosarcoma, Follicular or Nodular; Intrathoracic Lymph Nodes |
| 202.03 | Nodular Lymphoma; Brill-Symmers Disease; Follicular (Giant); Lymphocytic, Nodular; Follicular (Giant); Nodular; Reticulosarcoma, Follicular or Nodular; Intra-Abdominal Lymph Nodes |
| 202.04 | Nodular Lymphoma; Brill-Symmers Disease; Follicular (Giant); Lymphocytic, Nodular; Follicular (Giant); Nodular; Reticulosarcoma, Follicular or Nodular; Lymph Nodes of Axilla and Upper Limb |
| 202.05 | Nodular Lymphoma; Brill-Symmers Disease; Follicular (Giant); Lymphocytic, Nodular; Follicular (Giant); Nodular; Reticulosarcoma, Follicular or Nodular; Lymph Nodes of Inguinal Region and Lower Limb |
| 202.06 | Nodular Lymphoma; Brill-Symmers Disease; Follicular (Giant); Lymphocytic, Nodular; Follicular (Giant); Nodular; Reticulosarcoma, Follicular or Nodular; Intrapelvic Lymph Nodes |
| 202.07 | Nodular Lymphoma; Brill-Symmers Disease; Follicular (Giant); Lymphocytic, Nodular; Follicular (Giant); Nodular; Reticulosarcoma, Follicular or Nodular; Spleen |
| 202.08 | Nodular Lymphoma; Brill-Symmers Disease; Follicular (Giant); Lymphocytic, Nodular; Follicular (Giant); Nodular; Reticulosarcoma, Follicular or Nodular; Lymph Nodes of Multiple Sites |
| 202.1 | Mycosis Fungoides; Rare Chronic Inflammatory Malignant Disease Affecting the Superficial and Deep Layers of the Skin |
| 202.10 | Mycosis Fungoides; Unspecified Site, Extranodal and Solid Organ Sites |
| 202.11 | Mycosis Fungoides; Lymph Nodes of Head, Face, and Neck |
| 202.12 | Mycosis Fungoides; Intrathoracic Lymph Nodes |
| 202.13 | Mycosis Fungoides; Intra-Abdominal Lymph Nodes |
| 202.14 | Mycosis Fungoides; Lymph Nodes of Axilla and Upper Limb |
| 202.15 | Mycosis Fungoides; Lymph Nodes of Inguinal Region and Lower Limb |
| 202.16 | Mycosis Fungoides; Intrapelvic Lymph Nodes |
| 202.17 | Mycosis Fungoides; Spleen |
| 202.18 | Mycosis Fungoides; Lymph Nodes of Multiple Sites |
| 202.2 | SÃ©zary's Disease |
| 202.20 | SÃ©zary's Disease; Unspecified Site, Extranodal and Solid Organ Sites |
| 202.21 | SÃ©zary's Disease; Lymph Nodes of Head, Face, and Neck |
| 202.22 | SÃ©zary's Disease; Intrathoracic Lymph Nodes |
| 202.23 | SÃ©zary's Disease; Intra-abdominal Lymph Nodes |
| 202.24 | SÃ©zary's Disease; Lymph Nodes of Axilla and Upper Limb |
| 202.25 | SÃ©zary's Disease; Lymph Nodes of Inguinal Region and Lower Limb |
| 202.26 | SÃ©zary's Disease; Intrapelvic Lymph Nodes |
| 202.27 | SÃ©zary's Disease; Spleen |
| 202.28 | SÃ©zary's Disease; Lymph Nodes of Multiple Sites |
| 202.3 | Malignant Histiocytosis; Histiocytic Medullary Reticulosis; Reticuloendotheliosis; Reticulosis |
| 202.30 | Malignant Histiocytosis; Histiocytic Medullary Reticulosis; Reticuloendotheliosis; Reticulosis; Unspecified Site, Extranodal and Solid Organ Sites |
| 202.31 | Malignant Histiocytosis; Histiocytic Medullary Reticulosis; Reticuloendotheliosis; Reticulosis; Lymph Nodes of Head, Face, and Neck |
| 202.32 | Malignant Histiocytosis; Histiocytic Medullary Reticulosis; Reticuloendotheliosis; Reticulosis; Intrathoracic Lymph Nodes |
| 202.33 | Malignant Histiocytosis; Histiocytic Medullary Reticulosis; Reticuloendotheliosis; Reticulosis; Intra-abdominal Lymph Nodes |
| 202.34 | Malignant Histiocytosis; Histiocytic Medullary Reticulosis; Reticuloendotheliosis; Reticulosis; Lymph Nodes of Axilla and Upper Limb |
| 202.35 | Malignant Histiocytosis; Histiocytic Medullary Reticulosis; Reticuloendotheliosis; Reticulosis; Lymph Nodes of Inguinal Region and Lower Limb |
| 202.36 | Malignant Histiocytosis; Histiocytic Medullary Reticulosis; Reticuloendotheliosis; Reticulosis; Intrapelvic Lymph Nodes |
| 202.37 | Malignant Histiocytosis; Histiocytic Medullary Reticulosis; Reticuloendotheliosis; Reticulosis; Spleen |
| 202.38 | Malignant Histiocytosis; Histiocytic Medullary Reticulosis; Reticuloendotheliosis; Reticulosis; Lymph Nodes of Multiple Sites |
| 202.4 | Leukemic Reticuloendotheliosis; Hairy-cell Leukemia |
| 202.40 | Leukemic Reticuloendotheliosis; Hairy-Cell Leukemia; Unspecified Site, Extranodal and Solid Organ Sites |
| 202.41 | Leukemic Reticuloendotheliosis; Hairy-Cell Leukemia; Lymph Nodes of Head, Face, and Neck |
| 202.42 | Leukemic Reticuloendotheliosis; Hairy-Cell Leukemia; Intrathoracic Lymph Nodes |
| 202.43 | Leukemic Reticuloendotheliosis; Hairy-Cell Leukemia; Intra-Abdominal Lymph Nodes |
| 202.44 | Leukemic Reticuloendotheliosis; Hairy-Cell Leukemia; Lymph Nodes of Axilla and Upper Limb |
| 202.45 | Leukemic Reticuloendotheliosis; Hairy-Cell Leukemia; Lymph Nodes of Inguinal Region and Lower Limb |
| 202.46 | Leukemic Reticuloendotheliosis; Hairy-Cell Leukemia; Intrapelvic Lymph Nodes |
| 202.47 | Leukemic Reticuloendotheliosis; Hairy-Cell Leukemia; Spleen |
| 202.48 | Leukemic Reticuloendotheliosis; Hairy-Cell Leukemia; Lymph Nodes of Multiple Sites |
| 202.5 | Letterer-Siwe Disease; Differentiated Progressive Histiocytosis; Histiocytosis X (Progressive); Infantile Reticuloendotheliosis; Reticulosis of Infancy |
| 202.50 | Letterer-Siwe Disease; Differentiated Progressive Histiocytosis; Histiocytosis X (Progressive); Infantile Reticuloendotheliosis; Reticulosis of Infancy; Unspecified Site, Extranodal and Solid Organ Sites |
| 202.51 | Letterer-Siwe Disease; Differentiated Progressive Histiocytosis; Histiocytosis X (Progressive); Infantile Reticuloendotheliosis; Reticulosis of Infancy; Lymph Nodes of Head, Face, and Neck |
| 202.52 | Letterer-Siwe Disease; Differentiated Progressive Histiocytosis; Histiocytosis X (Progressive); Infantile Reticuloendotheliosis; Reticulosis of Infancy; Intrathoracic Lymph Nodes |
| 202.53 | Letterer-Siwe Disease; Differentiated Progressive Histiocytosis; Histiocytosis X (Progressive); Infantile Reticuloendotheliosis; Reticulosis of Infancy; Intra-abdominal Lymph Nodes |
| 202.54 | Letterer-Siwe Disease; Differentiated Progressive Histiocytosis; Histiocytosis X (Progressive); Infantile Reticuloendotheliosis; Reticulosis of Infancy; Lymph Nodes of Axilla and Upper Limb |
| 202.55 | Letterer-Siwe Disease; Differentiated Progressive Histiocytosis; Histiocytosis X (Progressive); Infantile Reticuloendotheliosis; Reticulosis of Infancy; Lymph Nodes of Inguinal Region and Lower Limb |
| 202.56 | Letterer-Siwe Disease; Differentiated Progressive Histiocytosis; Histiocytosis X (Progressive); Infantile Reticuloendotheliosis; Reticulosis of Infancy; Intrapelvic Lymph Nodes |
| 202.57 | Letterer-Siwe Disease; Differentiated Progressive Histiocytosis; Histiocytosis X (Progressive); Infantile Reticuloendotheliosis; Reticulosis of Infancy; Spleen |
| 202.58 | Letterer-Siwe Disease; Differentiated Progressive Histiocytosis; Histiocytosis X (Progressive); Infantile Reticuloendotheliosis; Reticulosis of Infancy; Lymph Nodes of Multiple Sites |
| 202.6 | Malignant Mast Cell Tumors; Mastocytoma; Mastocytosis; Mast Cell Sarcoma; Systemic Tissue Mast Cell Disease |
| 202.60 | Malignant Mast Cell Tumors; Mastocytoma, Mastocytosis; Mast Cell Sarcoma; Systemic Tissue Mast Cell Disease; Unspecified Site, Extranodal and Solid Organ Sites |
| 202.61 | Malignant Mast Cell Tumors; Mastocytoma, Mastocytosis; Mast Cell Sarcoma; Systemic Tissue Mast Cell Disease; Lymph Nodes of Head, Face, and Neck |
| 202.62 | Malignant Mast Cell Tumors; Mastocytoma, Mastocytosis; Mast Cell Sarcoma; Systemic Tissue Mast Cell Disease; Intrathoracic Lymph Nodes |
| 202.63 | Malignant Mast Cell Tumors; Mastocytoma, Mastocytosis; Mast Cell Sarcoma; Systemic Tissue Mast Cell Disease; Intra-abdominal Lymph Nodes |
| 202.64 | Malignant Mast Cell Tumors; Mastocytoma, Mastocytosis; Mast Cell Sarcoma; Systemic Tissue Mast Cell Disease; Lymph Nodes of Axilla and Upper Limb |
| 202.65 | Malignant Mast Cell Tumors; Mastocytoma, Mastocytosis; Mast Cell Sarcoma; Systemic Tissue Mast Cell Disease; Lymph Nodes of Inguinal Region and Lower Limb |
| 202.66 | Malignant Mast Cell Tumors; Mastocytoma, Mastocytosis; Mast Cell Sarcoma; Systemic Tissue Mast Cell Disease; Intrapelvic Lymph Nodes |
| 202.67 | Malignant Mast Cell Tumors; Mastocytoma, Mastocytosis; Mast Cell Sarcoma; Systemic Tissue Mast Cell Disease; Spleen |
| 202.68 | Malignant Mast Cell Tumors; Mastocytoma, Mastocytosis; Mast Cell Sarcoma; Systemic Tissue Mast Cell Disease; Lymph Nodes of Multiple Sites |
| 202.7 | Peripheral T cell lymphoma |
| 202.70 | Peripheral T cell lymphoma, unspecified site, extranodal and solid organ sites |
| 202.71 | Peripheral T cell lymphoma, lymph nodes of head, face, and neck |
| 202.72 | Peripheral T cell lymphoma, intrathoracic lymph nodes |
| 202.73 | Peripheral T cell lymphoma, intra-abdominal lymph nodes |
| 202.74 | Peripheral T cell lymphoma, lymph nodes of axilla and upper limb |
| 202.75 | Peripheral T cell lymphoma, lymph nodes of inguinal region and lower limb |
| 202.76 | Peripheral T cell lymphoma, intrapelvic lymph nodes |
| 202.77 | Peripheral T cell lymphoma, spleen |
| 202.78 | Peripheral T cell lymphoma, lymph nodes of multiple sites |
| 202.8 | Non-Hodgkin's Lymphoma |
| 202.80 | Lymphoma, Other; Lymphoma (Malignant) NOS, Diffuse; Unspecified Site, Extranodal and Solid Organ Sites |
| 202.81 | Lymphoma, Other; Lymphoma (Malignant) NOS, Diffuse; Lymph Nodes of Head, Face, and Neck |
| 202.82 | Lymphoma, Other; Lymphoma (Malignant) NOS, Diffuse; Intrathoracic Lymph Nodes |
| 202.83 | Lymphoma, Other; Lymphoma (Malignant) NOS, Diffuse; Intra-Abdominal Lymph Nodes |
| 202.84 | Lymphoma, Other; Lymphoma (Malignant) NOS, Diffuse; Lymph Nodes of Axilla and Upper Limb |
| 202.85 | Lymphoma, Other; Lymphoma (Malignant) NOS, Diffuse; Lymph Nodes of Inguinal Region and Lower Limb |
| 202.86 | Lymphoma, Other; Lymphoma (Malignant) NOS, Diffuse; Intrapelvic Lymph Nodes |
| 202.87 | Lymphoma, Other; Lymphoma (Malignant) NOS, Diffuse; Spleen |
| 202.88 | Lymphoma, Other; Lymphoma (Malignant) NOS, Diffuse; Lymph Nodes of Multiple Sites |
| 202.9 | Malignant Neoplasms of Lymphoid and Histiocytic Tissue, Other and Unspecified; Follicular Dendritic Cell Sarcoma; Interdigitating Dendritic Cell Sarcoma; Langerhans Cell Sarcoma; Malignant Neoplasm of Bone Marrow NOS |
| 202.90 | Malignant Neoplasms of Lymphoid and Histiocytic Tissue, Other and Unspecified; Follicular Dendritic Cell Sarcoma; Interdigitating Dendritic Cell Sarcoma; Langerhans Cell Sarcoma; Malignant Neoplasm of Bone Marrow NOS; Unspecified Site, Extranodal and Solid Organ Sites |
| 202.91 | Malignant Neoplasms of Lymphoid and Histiocytic Tissue, Other and Unspecified; Follicular Dendritic Cell Sarcoma; Interdigitating Dendritic Cell Sarcoma; Langerhans Cell Sarcoma; Malignant Neoplasm of Bone Marrow NOS; Lymph Nodes of Head, Face, and Neck |
| 202.92 | Malignant Neoplasms of Lymphoid and Histiocytic Tissue, Other and Unspecified; Follicular Dendritic Cell Sarcoma; Interdigitating Dendritic Cell Sarcoma; Langerhans Cell Sarcoma; Malignant Neoplasm of Bone Marrow NOS; Intrathoracic Lymph Nodes |
| 202.93 | Malignant Neoplasms of Lymphoid and Histiocytic Tissue, Other and Unspecified; Follicular Dendritic Cell Sarcoma; Interdigitating Dendritic Cell Sarcoma; Langerhans Cell Sarcoma; Malignant Neoplasm of Bone Marrow NOS; Intra-abdominal Lymph Nodes |
| 202.94 | Malignant Neoplasms of Lymphoid and Histiocytic Tissue, Other and Unspecified; Follicular Dendritic Cell Sarcoma; Interdigitating Dendritic Cell Sarcoma; Langerhans Cell Sarcoma; Malignant Neoplasm of Bone Marrow NOS; Lymph Nodes of Axilla and Upper Limb |
| 202.95 | Malignant Neoplasms of Lymphoid and Histiocytic Tissue, Other and Unspecified; Follicular Dendritic Cell Sarcoma; Interdigitating Dendritic Cell Sarcoma; Langerhans Cell Sarcoma; Malignant Neoplasm of Bone Marrow NOS; Lymph Nodes of Inguinal Region and Lower Limb |
| 202.96 | Malignant Neoplasms of Lymphoid and Histiocytic Tissue, Other and Unspecified; Follicular Dendritic Cell Sarcoma; Interdigitating Dendritic Cell Sarcoma; Langerhans Cell Sarcoma; Malignant Neoplasm of Bone Marrow NOS; Intrapelvic Lymph Nodes |
| 202.97 | Malignant Neoplasms of Lymphoid and Histiocytic Tissue, Other and Unspecified; Follicular Dendritic Cell Sarcoma; Interdigitating Dendritic Cell Sarcoma; Langerhans Cell Sarcoma; Malignant Neoplasm of Bone Marrow NOS; Spleen |
| 202.98 | Malignant Neoplasms of Lymphoid and Histiocytic Tissue, Other and Unspecified; Follicular Dendritic Cell Sarcoma; Interdigitating Dendritic Cell Sarcoma; Langerhans Cell Sarcoma; Malignant Neoplasm of Bone Marrow NOS; Lymph Nodes of Multiple Sites |
| 203 | Multiple Myeloma and Immunoproliferative Neoplasms |
| 203.0 | Multiple Myeloma |
| 203.00 | Multiple Myeloma without Mention of Having Achieved Remission; Failed Remission |
| 203.01 | Multiple Myeloma; Kahler's Disease, Myelomatosis; in Remission |
| 203.02 | Multiple myeloma, in relapse |
| 203.1 | Plasma Cell Leukemia; Plasmacytic Leukemia |
| 203.10 | Plasma Cell Leukemia; Plasmacytic Leukemia; without Mention of Having Achieved Remission; Failed Remission |
| 203.11 | Plasma Cell Leukemia; Plasmacytic Leukemia; in Remission |
| 203.12 | Plasma cell leukemia, in relapse |
| 203.8 | Immunoproliferative Neoplasms, Other |
| 203.80 | Immunoproliferative Neoplasms, Other; without Mention of Having Achieved Remission; Failed Remission |
| 203.81 | Immunoproliferative Neoplasms, Other; in Remission |
| 203.82 | Other immunoproliferative neoplasms, in relapse |
| 204 | Lymphoid Leukemia |
| 204.0 | Lymphoid Leukemia; Acute |
| 204.00 | Lymphoid Leukemia; Acute; without Mention of Having Achieved Remission; Failed Remission |
| 204.01 | Lymphoid Leukemia; in Remission |
| 204.02 | Acute lymphoid leukemia, in relapse |
| 204.1 | Lymphoid Leukemia; Chronic |
| 204.10 | Lymphoid Leukemia; Chronic; without Mention of Having Achieved Remission; Failed Remission |
| 204.11 | Lymphoid Leukemia; Chronic; in Remission |
| 204.12 | Chronic lymphoid leukemia, in relapse |
| 204.2 | Lymphoid Leukemia; Subacute |
| 204.20 | Lymphoid Leukemia; Subacute; without Mention of Having Achieved Remission; Failed Remission |
| 204.21 | Lymphoid Leukemia; Subacute; in Remission |
| 204.22 | Subacute lymphoid leukemia, in relapse |
| 204.8 | Lymphoid Leukemia; Other Lymphoid Leukemia; Aleukemic Leukemia: Lymphatic, Lymphocytic, Lymphoid |
| 204.80 | Lymphoid Leukemia; Other Lymphoid Leukemia; Aleukemic Leukemia: Lymphatic, Lymphocytic, Lymphoid; without Mention of Having Achieved Remission; Failed Remission |
| 204.81 | Lymphoid Leukemia; Other Lymphoid Leukemia; Aleukemic Leukemia: Lymphatic, Lymphocytic, Lymphoid; in Remission |
| 204.82 | Other lymphoid leukemia, in relapse |
| 204.9 | Lymphoid Leukemia, Unspecified; without Mention of Having Achieved Remission; Failed Remission |
| 204.90 | Lymphoid Leukemia, Unspecified; without Mention of Having Achieved Remission; Failed Remission |
| 204.91 | Lymphoid Leukemia, Unspecified, in Remission |
| 204.92 | Unspecified lymphoid leukemia, in relapse |
| 205 | Myeloid Leukemia |
| 205.0 | Myeloid Leukemia; Acute |
| 205.00 | Myeloid Leukemia; Acute; without Mention of Having Achieved Remission; Failed Remission |
| 205.01 | Myeloid Leukemia; Acute; in Remission |
| 205.02 | Acute myeloid leukemia, in relapse |
| 205.1 | Myeloid Leukemia; Chronic, Eosinophilic Leukemia, Neutrophilic Leukemia |
| 205.10 | Myeloid Leukemia; Chronic, Eosinophilic Leukemia, Neutrophilic Leukemia; without Mention of Having Achieved Remission; Failed Remission |
| 205.11 | Myeloid Leukemia; Chronic, Eosinophilic Leukemia, Neutrophilic Leukemia; in Remission |
| 205.12 | Chronic myeloid leukemia, in relapse |
| 205.2 | Myeloid Leukemia; Subacute |
| 205.20 | Myeloid Leukemia; Subacute; without Mention of Having Achieved Remission; Failed Remission |
| 205.21 | Myeloid Leukemia; Subacute; in Remission |
| 205.22 | Subacute myeloid leukemia, in relapse |
| 205.3 | Myeloid Leukemia; Myeloid Sarcoma; Chloroma; Granulocytic Sarcoma |
| 205.30 | Myeloid Leukemia; Myeloid Sarcoma; Chloroma; Granulocytic Sarcoma; without Mention of Having Achieved Remission; Failed Remission |
| 205.31 | Myeloid Leukemia; Myeloid Sarcoma; Chloroma; Granulocytic Sarcoma; IN Remission |
| 205.32 | Myeloid sarcoma, in relapse |
| 205.8 | Myeloid Leukemia; Other Myeloid Leukemia; Aleukemic Leukemia: Granulocytic, Myelogenous, Myeloid; Aleukemic Myelosis |
| 205.80 | Myeloid Leukemia; Other Myeloid Leukemia; Aleukemic Leukemia: Granulocytic, Myelogenous, Myeloid; Aleukemic Myelosis; without Mention of Having Achieved Remission; Failed Remission |
| 205.81 | Myeloid Leukemia; Other Myeloid Leukemia; Aleukemic Leukemia: Granulocytic, Myelogenous, Myeloid; Aleukemic Myelosis; in Remission |
| 205.82 | Other myeloid leukemia, in relapse |
| 205.9 | Myeloid Leukemia, Unspecified |
| 205.90 | Myeloid Leukemia, Unspecified; without Mention of Having Achieved Remission; Failed Remission |
| 205.91 | Myeloid Leukemia, Unspecified; in Remission |
| 205.92 | Unspecified myeloid leukemia, in relapse |
| 206 | Monocytic Leukemia |
| 206.0 | Monocytic Leukemia; Acute |
| 206.00 | Monocytic Leukemia; Acute; without Mention of Having Achieved Remission; Failed Remission |
| 206.01 | Monocytic Leukemia; Acute; in Remission |
| 206.02 | Acute monocytic leukemia, in relapse |
| 206.1 | Monocytic Leukemia; Chronic |
| 206.10 | Monocytic Leukemia; Chronic; without Mention of Having Achieved Remission; Failed Remission |
| 206.11 | Monocytic Leukemia; Chronic; in Remission |
| 206.12 | Chronic monocytic leukemia, in relapse |
| 206.2 | Monocytic Leukemia; Subacute |
| 206.20 | Monocytic Leukemia; Subacute; without Mention of Having Achieved Remission; Failed Remission |
| 206.21 | Monocytic Leukemia; Subacute; in Remission |
| 206.22 | Subacute monocytic leukemia, in relapse |
| 206.8 | Monocytic Leukemia, Other; Aleukemic: Monocytic Leukemia, Monocytoid Leukemia |
| 206.80 | Monocytic Leukemia, Other; Aleukemic: Monocytic Leukemia, Monocytoid Leukemia; without Mention of Having Achieved Remission; Failed Remission |
| 206.81 | Monocytic Leukemia, Other; Aleukemic: Monocytic Leukemia, Monocytoid Leukemia; in Remission |
| 206.82 | Other monocytic leukemia, in relapse |
| 206.9 | Monocytic Leukemia, Unspecified |
| 206.90 | Monocytic Leukemia, Unspecified; without Mention of Having Achieved Remission; Failed Remission |
| 206.91 | Monocytic Leukemia, Unspecified; in Remission |
| 206.92 | Unspecified monocytic leukemia, in relapse |
| 207 | Leukemia, Other Specified |
| 207.0 | Leukemia, Other Specified; Acute Erythremia and Erythroleukemia; Acute Erythremic Myelosis; Di Guglielmo's Disease; Erythremic Myelosis |
| 207.00 | Leukemia, Other Specified; Acute Erythremia and Erythroleukemia; Acute Erythremic Myelosis; Di Guglielmo's Disease; Erythremic Myelosis; without Mention of Having Achieved Remission; Failed Remission |
| 207.01 | Leukemia, Other Specified; Acute Erythremia and Erythroleukemia; Acute Erythremic Myelosis; Di Guglielmo's Disease; Erythremic Myelosis; in Remission |
| 207.02 | Acute erythremia and erythroleukemia, in relapse |
| 207.1 | Leukemia, Other Specified; Chronic Erythremia; Heilmeyer-SchÃ¶ner Disease |
| 207.10 | Leukemia, Other Specified; Chronic Erythremia; Heilmeyer-SchÃ¶ner Disease; without Mention of Having Achieved Remission; Failed Remission |
| 207.11 | Leukemia, Other Specified; Chronic Erythremia; Heilmeyer-SchÃ¶ner Disease; in Remission |
| 207.12 | Chronic erythremia, in relapse |
| 207.2 | Leukemia, Other Specified; Megakaryocytic Leukemia; Megakaryocytic Myelosis; Thrombocytic Leukemia |
| 207.20 | Leukemia, Other Specified; Megakaryocytic Leukemia; Megakaryocytic Myelosis; Thrombocytic Leukemia; without Mention of Having Achieved Remission; Failed Remission |
| 207.21 | Leukemia, Other Specified; Megakaryocytic Leukemia; Megakaryocytic Myelosis; Thrombocytic Leukemia; in Remission |
| 207.22 | Megakaryocytic leukemia, in relapse |
| 207.8 | Leukemia, Other Specified; Other Specified Leukemia; Lymphosarcoma Cell Leukemia |
| 207.80 | Leukemia, Other Specified; Other Specified Leukemia; Lymphosarcoma Cell Leukemia; without Mention of Having Achieved Remission; Failed Remission |
| 207.81 | Leukemia, Other Specified; Other Specified Leukemia; Lymphosarcoma Cell Leukemia; in Remission |
| 207.82 | Other specified leukemia, in relapse |
| 208 | Leukemia, Unspecified Cell Type |
| 208.0 | Leukemia, Acute, Unspecified Cell Type |
| 208.00 | Acute Leukemia of Unspecified Cell Type, without Mention of Having Achieved Remission; Failed Remission |
| 208.01 | Leukemia, Unspecified Cell Type, Acute; in Remission |
| 208.02 | Acute leukemia of unspecified cell type, in relapse |
| 208.1 | Leukemia, Chronic, Unspecified Cell Type |
| 208.10 | Leukemia, Chronic, without Mention of Having Achieved Remission; Failed Remission |
| 208.11 | Leukemia, Chronic, with Remission |
| 208.12 | Chronic leukemia of unspecified cell type, in relapse |
| 208.2 | Leukemia, Subacute, Unspecified Cell Type |
| 208.20 | Leukemia, Subacute, without Mention of Having Achieved Remission; Failed Remission |
| 208.21 | Leukemia, Subacute, with Remission |
| 208.22 | Subacute leukemia of unspecified cell type, in relapse |
| 208.8 | Leukemia, Other, Unspecified Cell Type |
| 208.80 | Leukemia, Unspecified Cell Type, Other; without Mention of Having Achieved Remission; Failed Remission |
| 208.81 | Leukemia, Unspecified Cell Type, Other; in Remission |
| 208.82 | Other leukemia of unspecified cell type, in relapse |
| 208.9 | Leukemia, Unspecified |
| 208.90 | Unspecified Leukemia, without Mention of Having Achieved Remission; Failed Remission |
| 208.91 | Leukemia, Unspecified; Leukemia NOS; in Remission |
| 208.92 | Unspecified leukemia, in relapse |
| 209 | Neuroendocrine Tumors |
| 209.0 | Neuroendocrine Tumors, Malignant Carcinoid Tumors of the Small Intestine |
| 209.00 | Neuroendocrine Tumors, Malignant Carcinoid Tumor of the Small Intestine, Unspecified Portion |
| 209.01 | Neuroendocrine Tumors, Malignant Carcinoid Tumor of the Duodenum |
| 209.02 | Neuroendocrine Tumors, Malignant Carcinoid Tumor of the Jejunum |
| 209.03 | Neuroendocrine Tumors, Malignant Carcinoid Tumor of the Ileum |
| 209.1 | Neuroendocrine Tumors, Malignant Carcinoid Tumors of the Appendix, Large Intestine, and Rectum |
| 209.10 | Neuroendocrine Tumors, Malignant Carcinoid Tumor of the Large Intestine, Unspecified Portion |
| 209.11 | Neuroendocrine Tumors, Malignant Carcinoid Tumor of the Appendix |
| 209.12 | Neuroendocrine Tumors, Malignant Carcinoid Tumor of the Cecum |
| 209.13 | Neuroendocrine Tumors, Malignant Carcinoid Tumor of the Ascending Colon |
| 209.14 | Neuroendocrine Tumors, Malignant Carcinoid Tumor of the Transverse Colon |
| 209.15 | Neuroendocrine Tumors, Malignant Carcinoid Tumor of the Descending Colon |
| 209.16 | Neuroendocrine Tumors, Malignant Carcinoid Tumor of the Sigmoid Colon |
| 209.17 | Neuroendocrine Tumors, Malignant Carcinoid Tumor of the Rectum |
| 209.2 | Neuroendocrine Tumors, Malignant Carcinoid Tumors of Other and Unspecified Sites |
| 209.20 | Malignant carcinoid tumor of unknown primary site |
| 209.21 | Neuroendocrine Tumors, Malignant Carcinoid Tumor of the Bronchus and Lung |
| 209.22 | Neuroendocrine Tumors, Malignant Carcinoid Tumor of the Thymus |
| 209.23 | Neuroendocrine Tumors, Malignant Carcinoid Tumor of the Stomach |
| 209.24 | Neuroendocrine Tumors, Malignant Carcinoid Tumor of the Kidney |
| 209.25 | Neuroendocrine Tumors, Malignant Carcinoid Tumor of the Foregut NOS |
| 209.26 | Neuroendocrine Tumors, Malignant Carcinoid Tumor of the Midgut NOS |
| 209.27 | Neuroendocrine Tumors, Malignant Carcinoid Tumor of the Hindgut NOS |
| 209.29 | Neuroendocrine Tumors, Malignant Carcinoid Tumors of Other Sites |
| 209.3 | Neuroendocrine Tumors, Malignant Poorly Differentiated Neuroendocrine Tumors |
| 209.30 | Neuroendocrine Tumors, Malignant Poorly Differentiated Neuroendocrine Carcinoma, Any Site; High Grade Neuroendocrine Carcinoma, Any Site; Malignant Poorly Differentiated Neuroendocrine Tumor NOS |
| 209.31 | Neuroendocrine Tumors, Merkel Cell Carcinoma of the Face; Ear; Eyelid, Including Canthus; Lip |
| 209.32 | Neuroendocrine Tumors, Merkel Cell Carcinoma of the Scalp and Neck |
| 209.33 | Neuroendocrine Tumors, Merkel Cell Carcinoma of the Upper Limb |
| 209.34 | Neuroendocrine Tumors, Merkel Cell Carcinoma of the Lower Limb |
| 209.35 | Neuroendocrine Tumors, Merkel Cell Carcinoma of the Trunk |
| 209.36 | Neuroendocrine Tumors, Merkel Cell Carcinoma of Other Sites; Buttock; Genitals; NOS |
| 209.4 | Neuroendocrine Tumors, Benign Carcinoid Tumors of the Small Intestine |
| 209.40 | Neuroendocrine Tumors, Benign Carcinoid Tumor of the Small Intestine, Unspecified Portion |
| 209.41 | Neuroendocrine Tumors, Benign Carcinoid Tumor of the Duodenum |
| 209.42 | Neuroendocrine Tumors, Benign Carcinoid Tumor of the Jejunum |
| 209.43 | Neuroendocrine Tumors, Benign Carcinoid Tumor of the Ileum |
| 209.5 | Neuroendocrine Tumors, Benign Carcinoid Tumors of the Appendix, Large Intestine, and Rectum |
| 209.50 | Neuroendocrine Tumors, Benign Carcinoid Tumor of the Large Intestine, Unspecified Portion; Colon NOS |
| 209.51 | Neuroendocrine Tumors, Benign Carcinoid Tumor of the Appendix |
| 209.52 | Neuroendocrine Tumors, Benign Carcinoid Tumor of the Cecum |
| 209.53 | Neuroendocrine Tumors, Benign Carcinoid Tumor of the Ascending Colon |
| 209.54 | Neuroendocrine Tumors, Benign Carcinoid Tumor of the Transverse Colon |
| 209.55 | Neuroendocrine Tumors, Benign Carcinoid Tumor of the Descending Colon |
| 209.56 | Neuroendocrine Tumors, Benign Carcinoid Tumor of the Sigmoid Colon |
| 209.57 | Neuroendocrine Tumors, Benign Carcinoid Tumor of the Rectum |
| 209.6 | Neuroendocrine Tumors, Benign Carcinoid Tumors of Other and Unspecified Sites |
| 209.60 | Neuroendocrine Tumors, Benign Carcinoid Tumor of Unknown Primary Site; Carcinoid Tumor NOS; Neuroendocrine Tumor NOS |
| 209.61 | Neuroendocrine Tumors, Benign Carcinoid Tumor of the Bronchus and Lung |
| 209.62 | Neuroendocrine Tumors, Benign Carcinoid Tumor of the Thymus |
| 209.63 | Neuroendocrine Tumors, Benign Carcinoid Tumor of the Stomach |
| 209.64 | Neuroendocrine Tumors, Benign Carcinoid Tumor of the Kidney |
| 209.65 | Neuroendocrine Tumors, Benign Carcinoid Tumor of the Foregut NOS |
| 209.66 | Neuroendocrine Tumors, Benign Carcinoid Tumor of the Midgut NOS |
| 209.67 | Neuroendocrine Tumors, Benign Carcinoid Tumor of the Hindgut NOS |
| 209.69 | Neuroendocrine Tumors, Benign Carcinoid Tumors of Other Sites |
| 209.7 | Neuroendocrine Tumors, Secondary Neuroendocrine Tumors; Secondary Carcinoid Tumors |
| 209.70 | Neuroendocrine Tumors, Secondary Neuroendocrine Tumor, Unspecified Site |
| 209.71 | Neuroendocrine Tumors, Secondary Neuroendocrine Tumor of Distant Lymph Nodes; Mesentery Metastasis of Neuroendocrine Tumor |
| 209.72 | Neuroendocrine Tumors, Secondary Neuroendocrine Tumor of Liver |
| 209.73 | Neuroendocrine Tumors, Secondary Neuroendocrine Tumor of Bone |
| 209.74 | Neuroendocrine Tumors, Secondary Neuroendocrine Tumor of Peritoneum |
| 209.75 | Neuroendocrine Tumors, Secondary Merkel Cell Carcinoma; Merkel Cell Carcinoma Nodal Presentation; Merkel Cell Carcinoma Visceral Metastatic Presentation; Secondary Merkel Cell Carcinoma, Any Site |
| 209.79 | Neuroendocrine Tumors, Secondary Neuroendocrine Tumor of Other Sites |
| 210 | Neoplasm, Lip, Oral Cavity, and Pharynx, Benign |
| 210.0 | Benign Neoplasm of Lip, Oral Cavity, and Pharynx; Lip; Frenulum labii; Lip (Inner Aspect) (Mucosa) (Vermilion Border) |
| 210.1 | Benign Neoplasm of Lip, Oral Cavity, and Pharynx; Tongue; Lingual Tonsil |
| 210.2 | Benign Neoplasm of Major Salivary Glands |
| 210.3 | Benign Neoplasm of Lip, Oral Cavity, and Pharynx; Floor of Mouth |
| 210.4 | Other and Unspecified Parts of Mouth; Gingiva; Gum (Upper) (Lower); Labial Commissure; Oral Mucosa; Palate (Hard) (Soft); Uvula |
| 210.5 | Benign Neoplasm of Lip, Oral Cavity, and Pharynx; Tonsil; Tonsil (Faucial) (Palatine) |
| 210.6 | Benign Neoplasm of Lip, Oral Cavity, and Pharynx; Other Parts of Oropharynx; Branchial Cleft or Vestiges; Epiglottis, Anterior Aspect; Fossa; Pillars; Vallecula |
| 210.7 | Benign Neoplasm of Lip, Oral Cavity, and Pharynx; Nasopharynx; Adenoid Tissue; Lymphadenoid Tissue; Pharyngeal Tonsil; Posterior Nasal Septum |
| 210.8 | Benign Neoplasm of Lip, Oral Cavity, and Pharynx; Hypopharynx; Arytenoid Fold; Laryngopharynx; Postcricoid Region; Pyriform Fossa |
| 210.9 | Benign Neoplasm of Lip, Oral Cavity, and Pharynx; Pharynx, Unspecified |
| 211 | Neoplasm, Other Parts of Digestive System, Benign |
| 211.0 | Benign Neoplasm of Other Parts of Digestive System; Esophagus |
| 211.1 | Benign Neoplasm of Other Parts of Digestive System; Stomach; Body of Stomach; Cardia of Stomach; Fundus of Stomach; Cardiac Orifice; Pylorus |
| 211.2 | Benign Neoplasm of Other Parts of Digestive System; Duodenum, Jejunum, and Ileum |
| 211.3 | Polyp of Colon |
| 211.4 | Benign Neoplasm of Other Parts of Digestive System; Rectum and Anal Canal; Anal Canal or Sphincter; Rectosigmoid Junction |
| 211.5 | Benign Neoplasm of Other Parts of Digestive System; Liver and Biliary Passages; Ampulla of Vater; Common Bile Duct; Cystic Duct; Gallbladder; Hepatic Duct; Sphincter of Oddi |
| 211.6 | Benign Neoplasm of Other Parts of Digestive System; Pancreas, except Islets of Langerhans |
| 211.7 | Benign Neoplasm of Other Parts of Digestive System; Islets of Langerhans; Islet Cell Tumor; Use Additional Code to Identify Any Functional Activity |
| 211.8 | Benign Neoplasm of Other Parts of Digestive System; Retroperitoneum and Peritoneum; Mesentery; Mesocolon; Omentum; Retroperitoneal Tissue |
| 211.9 | Benign Neoplasm of Other Parts of Digestive System; Other and Unspecified Site; Alimentary Tract NOS; Digestive System NOS; Gastrointestinal Tract NOS; Intestinal Tract NOS; Intestine NOS; Spleen, Not Elsewhere Classified |
| 212 | Neoplasm, Respiratory and Intrathoracic Organs, Benign |
| 212.0 | Neoplasm, Respiratory and Intrathoracic Organs, Benign, Nasal Cavities, Middle Ear, and Accessory Sinuses; Cartilage of Nose; Eustachian Tube; Nares; Septum of Nose; Ethmoidal; Frontal; Maxillary; Sphenoidal |
| 212.1 | Neoplasm, Respiratory and Intrathoracic Organs, Benign, Larynx; Arytenoid; Cricoid; Cuneiform; Thyroid; Glottis; Vocal Cords (False) (True) |
| 212.2 | Neoplasm, Respiratory and Intrathoracic Organs, Benign, Trachea |
| 212.3 | Neoplasm, Respiratory and Intrathoracic Organs, Benign, Bronchus and Lung; Carina; Hilus of Lung |
| 212.4 | Neoplasm, Respiratory and Intrathoracic Organs, Benign, Pleura |
| 212.5 | Neoplasm, Respiratory and Intrathoracic Organs, Benign, Mediastinum |
| 212.6 | Neoplasm, Respiratory and Intrathoracic Organs, Benign, Thymus |
| 212.7 | Neoplasm, Respiratory and Intrathoracic Organs, Benign, Heart |
| 212.8 | Neoplasm, Respiratory and Intrathoracic Organs, Benign, Other specified sites |
| 212.9 | Neoplasm, Respiratory and Intrathoracic Organs, Benign, Site unspecified |
| 213 | Benign Neoplasm of Bone and Articular Cartilage |
| 213.0 | Neoplasm, Bones of Skull and Face, Benign |
| 213.1 | Neoplasm, Lower Jaw Bone, Benign |
| 213.2 | Neoplasm, Vertebral Column, Excluding Sacrum and Coccyx, Benign |
| 213.3 | Neoplasm, Ribs, Sternum, and Clavicle, Benign |
| 213.4 | Neoplasm, Scapula and Long Bones of Upper Limb, Benign |
| 213.5 | Neoplasm, Short Bones of Upper Limb, Benign |
| 213.6 | Neoplasm, Pelvic Bones, Sacrum, and Coccyx, Benign |
| 213.7 | Neoplasm, Long Bones of Lower Limb, Benign |
| 213.8 | Neoplasm, Short Bones of Lower Limb, Benign |
| 213.9 | Neoplasm, Bone and Articular Cartilage, Benign, Site Unspecified |
| 214 | Lipoma |
| 214.0 | Lipoma; Skin and Subcutaneous Tissue of Face |
| 214.1 | Lipoma; Skin and Subcutaneous Tissue, Other |
| 214.2 | Lipoma; Intrathoracic Organs |
| 214.3 | Lipoma; Intra-abdominal Organs |
| 214.4 | Lipoma; Spermatic Cord |
| 214.8 | Lipoma; Specified Sites, Other |
| 214.9 | Lipoma, Unspecified Site |
| 215 | Benign Neoplasm of Connective and Other Soft Tissue, Other |
| 215.0 | Benign Neoplasm of Connective and Other Soft Tissue, Other; Head, Face and Neck |
| 215.2 | Benign Neoplasm of Connective and Other Soft Tissue, Other; Upper Limb, Including Shoulder |
| 215.3 | Benign Neoplasm of Connective and Other Soft Tissue, Other; Lower Limb, Including Hip |
| 215.4 | Benign Neoplasm of Connective and Other Soft Tissue, Other; Thorax |
| 215.5 | Benign Neoplasm of Connective and Other Soft Tissue, Other; Abdomen, Abdominal Wall, Benign Stromal Tumors of Abdomen, Hypochondrium |
| 215.6 | Benign Neoplasm of Connective and Other Soft Tissue, Other; Pelvis, Buttock, Groin, Inguinal Region, Perineum |
| 215.7 | Benign Neoplasm of Connective and Other Soft Tissue, Other; Trunk, Unspecified; Back NOS; Flank NOS |
| 215.8 | Benign Neoplasm of Connective and Other Soft Tissue, Other; Specified Sites, Other |
| 215.9 | Benign Neoplasm of Connective and Other Soft Tissue, Other; Site Unspecified |
| 216 | Benign Neoplasm of Skin |
| 216.0 | Benign Neoplasm of Skin, Skin of Lip |
| 216.1 | Benign Neoplasm of Skin, Eyelid, including Canthus |
| 216.2 | Benign Neoplasm of Skin, Ear and External Auditory Canal; Auricle (Ear); Auricular Canal, External; External Meatus; Pinna |
| 216.3 | Benign Neoplasm of Skin, Skin of Other and Unspecified Parts of Face; Cheek, External; Eyebrow; Nose, External; Temple |
| 216.4 | Benign Neoplasm of Skin, Scalp and Skin of Neck |
| 216.5 | Benign Neoplasm of Skin, Skin of Trunk, except Scrotum; Axillary Fold; Perianal Skin; Abdominal Wall; Anus; Back; Breast; Buttock; Chest Wall; Groin; Perineum; Umbilicus |
| 216.6 | Benign Neoplasm of Skin, Skin of Upper Limb, including Shoulder |
| 216.7 | Skin of Lower Limb, Including Hip |
| 216.8 | Sites of Skin, Other Specified |
| 216.9 | Skin, Site Unspecified |
| 217 | Neoplasm, Benign Breast Nipple |
| 218 | Uterine Leiomyoma |
| 218.0 | Uterine Leiomyoma; Submucous Leiomyoma of Uterus |
| 218.1 | Uterine Leiomyoma; Intramural Leiomyoma of Uterus; Interstitial Leiomyoma of Uterus |
| 218.2 | Uterine Leiomyoma; Subserous Leiomyoma of Uterus; Subperitoneal Leiomyoma of Uterus |
| 218.9 | Uterine Leiomyoma, Unspecified |
| 219 | Neoplasm, Uterus, Other Benign |
| 219.0 | Neoplasm, Uterus, Other Benign, Cervix Uteri |
| 219.1 | Neoplasm, Uterus, Other Benign, Corpus Uteri; Endometrium; Fundus; Myometrium |
| 219.8 | Neoplasm, Uterus, Other Benign, Other Specified Parts of Uterus |
| 219.9 | Neoplasm, Uterus, Other Benign, Uterus, Part Unspecified |
| 220 | Benign Neoplasm of Ovary |
| 221 | Benign Neoplasm of Other Female Genital Organs |
| 221.0 | Benign Neoplasm of Other Female Genital Organs; Fallopian Tube and Uterine Ligaments; Oviduct; Parametrium; Uterine Ligament (Broad) (Round) (Uterosacral); Uterine Tube |
| 221.1 | Benign Neoplasm of Other Female Genital Organs; Vagina |
| 221.2 | Benign Neoplasm of Other Female Genital Organs; Vulva; Clitoris; Greater Vestibular [Bartholin's] Gland; Labia (Majora) (Minora); Pudendum |
| 221.8 | Benign Neoplasm of Other Female Genital Organs; Other Specified Sites of Female Genital Organs |
| 221.9 | Benign Neoplasm of Other Female Genital Organs; Female Genital Organ, Site Unspecified |
| 222 | Benign Neoplasm of Male Genital Organs |
| 222.0 | Benign Neoplasm of Male Genital Organs, Testis |
| 222.1 | Benign Neoplasm of Male Genital Organs, Penis; Corpus Cavernosum; Glans Penis; Prepuce |
| 222.2 | Benign Neoplasm of Male Genital Organs, Prostate |
| 222.3 | Benign Neoplasm of Male Genital Organs, Epididymis |
| 222.4 | Benign Neoplasm of Male Genital Organs, Scrotum; Skin of Scrotum |
| 222.8 | Benign Neoplasm of Male Genital Organs, Other Specified Sites of Male Genital Organs; Seminal Vesicle; Spermatic Cord |
| 222.9 | Benign Neoplasm of Male Genital Organs, Male Genital Organ, Site Unspecified |
| 223 | Benign Neoplasm of Kidney and Other Urinary Organs |
| 223.0 | Benign Neoplasm of Kidney and Other Urinary Organs; Kidney, except Pelvis |
| 223.1 | Benign Neoplasm of Kidney and Other Urinary Organs; Renal Pelvis |
| 223.2 | Benign Neoplasm of Kidney and Other Urinary Organs; Ureter |
| 223.3 | Benign Neoplasm of Kidney and Other Urinary Organs; Bladder |
| 223.8 | Benign Neoplasm of Kidney and Other Urinary Organs; Specified Sites of Urinary Organs, Other |
| 223.81 | Benign Neoplasm of Kidney and Other Urinary Organs; Specified Sites of Urinary Organs, Other; Urethra |
| 223.89 | Benign Neoplasm of Kidney and Other Urinary Organs; Specified Sites of Urinary Organs, Other; Other |
| 223.9 | Benign Neoplasm of Kidney and Other Urinary Organs; Urinary Organ, Site Unspecified; Urinary System NOS |
| 224 | Benign Neoplasm of Eye |
| 224.0 | Benign Neoplasm of Eye, Eyeball, except Conjunctiva, Cornea, Retina, and Choroid; Ciliary Body; Iris; Sclera; Uveal Tract |
| 224.1 | Benign Neoplasm of Eye, Orbit |
| 224.2 | Benign Neoplasm of Eye, Lacrimal Gland |
| 224.3 | Benign Neoplasm of Eye, Conjunctiva |
| 224.4 | Benign Neoplasm of Eye, Cornea |
| 224.5 | Benign Neoplasm of Eye, Retina |
| 224.6 | Benign Neoplasm of Eye, Choroid |
| 224.7 | Benign Neoplasm of Eye, Lacrimal Duct; Lacrimal Sac; Nasolacrimal Duct |
| 224.8 | Benign Neoplasm of Eye, Other Specified Parts of Eye |
| 224.9 | Benign Neoplasm of Eye, Part Unspecified |
| 225 | Benign Neoplasm of Brain and Other Parts of Nervous System |
| 225.0 | Benign Neoplasm of Brain and Other Parts of Nervous System; Brain |
| 225.1 | Benign Neoplasm of Brain and Other Parts of Nervous System; Cranial Nerves |
| 225.2 | Benign Neoplasm of Brain and Other Parts of Nervous System; Cerebral Meninges; Meninges NOS; Meningioma, Cerebral |
| 225.3 | Benign Neoplasm of Brain and Other Parts of Nervous System; Spinal Cord; Cauda Equina |
| 225.4 | Benign Neoplasm of Brain and Other Parts of Nervous System; Spinal Meninges; Spinal Meningioma |
| 225.8 | Benign Neoplasm of Brain and Other Parts of Nervous System; Other Specified Sites of Nervous System |
| 225.9 | Benign Neoplasm of Brain and Other Parts of Nervous System; Nervous System, Part Unspecified; Nervous System (Central), NOS |
| 226 | Benign Neoplasm of Thyroid Glands |
| 227 | Benign Neoplasm of Other Endocrine Glands and Related Structures |
| 227.0 | Benign Neoplasm of Other Endocrine Glands and Related Structures; Adrenal Gland; Suprarenal Gland |
| 227.1 | Benign Neoplasm of Other Endocrine Glands and Related Structures; Parathyroid Gland |
| 227.3 | Benign Neoplasm of Other Endocrine Glands and Related Structures; Pituitary Gland and Craniopharyngeal Duct; Craniobuccal Pouch; Hypophysis; Rathke's Pouch; Sella Turcica |
| 227.4 | Benign Neoplasm of Other Endocrine Glands and Related Structures; Pineal Gland; Pineal Body |
| 227.5 | Benign Neoplasm of Other Endocrine Glands and Related Structures; Carotid Body |
| 227.6 | Benign Neoplasm of Other Endocrine Glands and Related Structures; Aortic Body and Other Paraganglia; Coccygeal Body; Glomus Jugulare; Para-aortic Body |
| 227.8 | Benign Neoplasm of Other Endocrine Glands and Related Structures; Other |
| 227.9 | Benign Neoplasm of Other Endocrine Glands and Related Structures; Endocrine Gland, Site Unspecified |
| 228 | Hemangioma and Lymphangioma, Any Site |
| 228.0 | Hemangioma, Any Site |
| 228.00 | Hemangioma, Any Site, of Unspecified Site |
| 228.01 | Hemangioma, Any Site, of Skin and Subcutaneous Tissue |
| 228.02 | Hemangioma, Any Site, of Intracranial Structures |
| 228.03 | Hemangioma, Any Site, of Retina |
| 228.04 | Hemangioma, Any Site, of Intra-abdominal Structures; Peritoneum; Retroperitoneal Tissue |
| 228.09 | Hemangioma, Any Site, of Other Sites; Systemic Angiomatosis |
| 228.1 | Lymphangioma, Any Site; Congenital Lymphangioma; Lymphatic Nevus |
| 229 | Neoplasm, Other and Unspecified Sites, Benign |
| 229.0 | Benign Neoplasm of Other and Unspecified Sites; Lymph Nodes |
| 229.8 | Benign Neoplasm of Other and Unspecified Sites; Other Specified Sites; Intrathoracic NOS; Thoracic NOS |
| 229.9 | Neoplasm, Benign; Site Unspecified |
| 230 | Carcinoma in Situ of Digestive Organs |
| 230.0 | Carcinoma in Situ of Lip, Oral Cavity, Oropharynx, Nasopharynx, Salivary Gland, Tongue |
| 230.1 | Carcinoma in Situ of Esophagus |
| 230.2 | Carcinoma in Situ of Stomach |
| 230.3 | Carcinoma in Situ of Colon, Appendix, Cecum, Ileocecal Valve, Large Intestine NOS |
| 230.4 | Carcinoma in Situ of Rectum, Rectosigmoid Junction |
| 230.5 | Carcinoma in Situ of Digestive Organs, Anal Canal; Anal Sphincter |
| 230.6 | Carcinoma in Situ of Digestive Organs, Anus, Unspecified |
| 230.7 | Carcinoma in Situ of Other and Unspecified Parts of Intestine, Duodenum, Ileum, Jejunum, Small Intestine NOS |
| 230.8 | Carcinoma in Situ of Liver and Biliary System, Ampulla of Vater, Common Bile Duct, Cystic Duct, Gallbladder, Hepatic Duct, Sphincter of Oddi |
| 230.9 | Carcinoma in Situ of Other and Unspecified Digestive Organs; Digestive Organ NOS; Gastrointestinal Tract NOS; Pancreas; Spleen |
| 231 | Carcinoma in Situ of Respiratory System |
| 231.0 | Larynx; Arytenoid; Cricoid; Cuneiform; Thyroid; Posterior Surface; Suprahyoid Portion; Vocal Cords (False) (True) |
| 231.1 | Carcinoma in Situ of Respiratory System, Trachea |
| 231.2 | Carcinoma in Situ of Bronchus and Lung; Carina, Hilus of Lung |
| 231.8 | Other Specified Parts of Respiratory System; Accessory Sinuses; Middle Ear; Nasal Cavities; Pleura |
| 231.9 | Respiratory System, Part Unspecified |
| 232 | Carcinoma in Situ of Skin |
| 232.0 | Carcinoma in Situ of Skin of Lip |
| 232.1 | Carcinoma in Situ of Eyelid, Including Canthus |
| 232.2 | Carcinoma in Situ of Ear and External Auditory Canal |
| 232.3 | Carcinoma in Situ of Skin of Other and Unspecified Parts of Face |
| 232.4 | Carcinoma in Situ of Scalp and Skin of Neck |
| 232.5 | Carcinoma in Situ of Skin of Trunk, except Scrotum; Anus, Margin; Axillary Fold; Perianal Skin; Skin of: Abdominal Wall, Anus, Back, Breast, Buttock, Chest Wall, Groin, Perineum; Umbilicus |
| 232.6 | Carcinoma in Situ of Skin of Upper Limb, Including Shoulder |
| 232.7 | Carcinoma in Situ of Skin of Lower Limb, Including Hip |
| 232.8 | Carcinoma in Situ of Other Specified Sites of Skin |
| 232.9 | Carcinoma in Situ of Skin, Site Unspecified |
| 233 | Carcinoma in Situ of Breast and Genitourinary System |
| 233.0 | Carcinoma in Situ of the Breast |
| 233.1 | Carcinoma in Situ of Cervix Uteri; Adenocarcinoma in Situ of Cervix; Cervical Intraepithelial Glandular Neoplasia, grade III; Cervical Intraepithelial Neoplasia III [CIN III]; Severe Dysplasia of Cervix |
| 233.2 | Carcinoma in Situ of Other and Unspecified Parts of Uterus |
| 233.3 | Carcinoma in Situ of Other and Unspecified Female Genital Organs |
| 233.30 | Carcinoma in Situ; Other and Unspecified Female Genital Organs; Unspecified Female Genital Organ |
| 233.31 | Carcinoma in Situ; Other and Unspecified Female Genital Organs; Vagina; Severe Dysplasia of Vagina, Vaginal Intraepithelial Neoplasia [VAIN III] |
| 233.32 | Carcinoma in Situ; Other and Unspecified Female Genital Organs; Vulva; Severe Dysplasia of Vulva, Vulvar Intraepithelial Neoplasia [VIN III] |
| 233.39 | Carcinoma in Situ; Other and Unspecified Female Genital Organs; Other Female Genital Organ |
| 233.4 | Carcinoma in Situ of Prostate |
| 233.5 | Carcinoma in Situ of Breast and Genitourinary System, Penis |
| 233.6 | Carcinoma in Situ of Other and Unspecified Male Genital Organs |
| 233.7 | Carcinoma in Situ of Bladder |
| 233.9 | Carcinoma in Situ of Other and Unspecified Urinary Organs |
| 234 | Carcinoma in Situ of Other and Unspecified Sites |
| 234.0 | Carcinoma in Situ of Other and Unspecified Sites, Eye |
| 234.8 | Carcinoma in Situ of Other and Unspecified Sites, Specified Sites, Other; Endocrine Gland, Any |
| 234.9 | Carcinoma in Situ of Other and Unspecified Sites, Site unspecified; Carcinoma in Situ NOS |
| 235 | Neoplasm of Uncertain Behavior of Digestive and Respiratory Systems |
| 235.0 | Major Salivary Glands; Parotid; Sublingual; Submandibular |
| 235.1 | Neoplasm of Uncertain Behavior of Lip, Oral Cavity and Pharynx; Gingiva, Hypopharynx, Minor Salivary Glands, Mouth, Nasopharynx, Oropharynx, Tongue |
| 235.2 | Neoplasm of Uncertain Behavior of Stomach, Intestines and Rectum |
| 235.3 | Liver and biliary passages; Ampulla of Vater; Bile Ducts [Any]; Gallbladder; Liver |
| 235.4 | Retroperitoneum and Peritoneum |
| 235.5 | Neoplasm of Uncertain Behavior of Other and Unspecified Digestive Organs; Anal, Canal, Sphincter; Anus NOS; Esophagus; Pancreas; Spleen |
| 235.6 | Neoplasm of Uncertain Behavior of Digestive and Respiratory Systems, Larynx |
| 235.7 | Trachea, Bronchus, and Lung |
| 235.8 | Neoplasm of Uncertain Behavior of Pleura, Thymus and Mediastinum |
| 235.9 | Other and Unspecified Respiratory Organs; Accessory Sinuses; Middle Ear; Nasal Cavities |
| 236 | Neoplasm of Uncertain Behavior of Genitourinary Organs |
| 236.0 | Neoplasm of Uncertain Behavior of Genitourinary Organs, Uterus |
| 236.1 | Placenta; Chorioadenoma (Destruens); Invasive Mole; Malignant Hydatid(Iform) Mole |
| 236.2 | Neoplasm of Uncertain Behavior of Genitourinary Organs, Ovary |
| 236.3 | Neoplasm of Uncertain Behavior of Genitourinary Organs, Other and Unspecified Female Genital Organs |
| 236.4 | Neoplasm of Uncertain Behavior of Genitourinary Organs, Testis |
| 236.5 | Neoplasm of Uncertain Behavior of Genitourinary Organs, Prostate |
| 236.6 | Neoplasm of Uncertain Behavior of Genitourinary Organs; Other and unspecified male genital organs |
| 236.7 | Neoplasm of Uncertain Behavior of Genitourinary Organs; Bladder Polyps |
| 236.9 | Neoplasm of Uncertain Behavior of Genitourinary Organs, Other and Unspecified Urinary Organs |
| 236.90 | Neoplasms of Uncertain Behavior or Genitourinary Organs, Urinary Organ, Unspecified |
| 236.91 | Neoplasm of Uncertain Behavior of Genitourinary Organs; Kidney and Ureter |
| 236.99 | Neoplasm of Uncertain Behavior of Genitourinary Organs, Other and Unspecified Urinary Organs, Other |
| 237 | Neoplasm of Uncertain Behavior of Endocrine Glands and Nervous System |
| 237.0 | Neoplasm of Uncertain Behavior of Endocrine Glands and Nervous System; Pituitary Gland and Craniopharyngeal Duct |
| 237.1 | Neoplasm of Uncertain Behavior of Endocrine Glands and Nervous System, Pineal Gland Neoplasm |
| 237.2 | Neoplasm of Uncertain Behavior of Endocrine Glands and Nervous System; Adrenal Gland; Suprarenal Gland |
| 237.3 | Neoplasm of Uncertain Behavior of Endocrine Glands and Nervous System; Paraganglia; Aortic body; Carotid body; Coccygeal Body; Glomus Jugulare |
| 237.4 | Other and Unspecified Endocrine Glands; Parathyroid Gland; Thyroid Gland |
| 237.5 | Neoplasm of Uncertain Behavior of Endocrine Glands and Nervous System, Brain and Spinal Cord |
| 237.6 | Neoplasm of Uncertain Behavior of Endocrine Glands and Nervous System, Meninges, NOS, Cerebral, Spinal |
| 237.7 | Neurofibromatosis |
| 237.70 | Neurofibromatosis, Unspecified |
| 237.71 | Neurofibromatosis, Type I; von Recklinghausen's Disease |
| 237.72 | Neurofibromatosis, Type II; Acoustic Neurofibromatosis |
| 237.73 | Schwannomatosis |
| 237.79 | Other Neurofibromatosis |
| 237.9 | Other and Unspecified Parts of Nervous System; Cranial Nerves |
| 238 | Neoplasm of Uncertain Behavior of Other and Unspecified Sites and Tissues |
| 238.0 | Neoplasm of Uncertain Behavior of Bone and Articular Cartilage |
| 238.1 | Neoplasm of Uncertain Behavior of Other and Unspecified Sites and Tissues; Connective and Other Soft Tissue; Peripheral, Sympathetic, and Parasympathetic Nerves and Ganglia |
| 238.2 | Neoplasm of Uncertain Behavior, Skin |
| 238.3 | Neoplasm of Uncertain Behavior of Other and Unspecified Sites and Tissues, Breast |
| 238.4 | Neoplasm of Uncertain Behavior, Polycythemia Vera |
| 238.5 | Neoplasm of Uncertain Behavior of Other and Unspecified Sites and Tissues; Histiocytic and Mast Cells; Mast Cell Tumor NOS; Mastocytoma NOS |
| 238.6 | Neoplasm of Uncertain Behavior of Other and Unspecified Sites and Tissues; Plasma Cells; Plasmacytoma NOS; Solitary Myeloma |
| 238.7 | Neoplasm of Uncertain Behavior of Other and Unspecified Sites and Tissues, Other Lymphatic and Hematopoietic Tissues |
| 238.71 | Essential Thrombocythemia |
| 238.72 | Low Grade Myelodysplastic Syndrome Lesions; Refractory anemia with excess blasts-1(RAEB-1) |
| 238.73 | High Grade Myelodysplastic Syndrome Lesions |
| 238.74 | Myelodysplastic Syndrome with 5q Deletion |
| 238.75 | Myelodysplastic Syndrome, Unspecified |
| 238.76 | Myelofibrosis with Myeloid Metaplasia |
| 238.77 | Post-transplant lymphoproliferative disorder (PTLD) |
| 238.79 | Other Lymphatic and Hematopoietic Tissues |
| 238.8 | Neoplasm of Uncertain Behavior of Other and Unspecified Sites and Tissues; Other specified sites; Eye; Heart |
| 238.9 | Neoplasm of Uncertain Behavior of Other and Unspecified Sites and Tissues; Site unspecified |
| 239 | Neoplasms of Unspecified Nature |
| 239.0 | Neoplasms of Unspecified Nature of Digestive System |
| 239.1 | Neoplasm of Unspecified Nature of Respiratory System |
| 239.2 | Neoplasms of Unspecified Nature of Bone, Soft Tissue, and Skin |
| 239.3 | Neoplasm of Unspecified Nature; Breast |
| 239.4 | Neoplasm of Unspecified Nature, Bladder |
| 239.5 | Neoplasm of Other Genitourinary Organs |
| 239.6 | Neoplasm of Unspecified Nature of Brain |
| 239.7 | Neoplasm of Unspecified Nature, Endocrine Glands and Other Parts of Nervous System |
| 239.8 | Neoplasms of Unspecified Nature; Other specified sites |
| 239.81 | Neoplasms of unspecified nature, retina and choroid |
| 239.89 | Neoplasms of unspecified nature, other specified sites |
| 239.9 | Neoplasms of Unspecified Nature; Site unspecified |
| 344 | Paralytic Syndromes, Other; |
| 344.0 | Quadriplegia and Quadriparesis |
| 344.00 | Quadriplegia, Unspecified |
| 344.01 | Quadriplegia C1-C4, Complete |
| 344.02 | Quadriplegia C1-C4, Incomplete |
| 344.03 | Quadriplegia C5-C7, Complete |
| 344.04 | Quadriplegia C5-C7, Incomplete |
| 344.09 | Quadriplegia and Quadriparesis, Other |
| 344.1 | Paraplegia |
| 344.2 | Diplegia of Upper Limbs; Diplegia (Upper); Paralysis of Both Upper Limbs |
| 344.3 | Monoplegia of Lower Limb; Paralysis of Lower Limb |
| 344.30 | Monoplegia of Lower Limb, Affecting Unspecified Side |
| 344.31 | Monoplegia of Lower Limb, Affecting Dominant Side |
| 344.32 | Monoplegia of Lower Limb, Affecting Nondominant Side |
| 344.4 | Monoplegia of Upper Limb; Paralysis of Upper Limb |
| 344.40 | Monoplegia of Upper Limb, Affecting Unspecified Side |
| 344.41 | Monoplegia of Upper Limb, Affecting Dominant Side |
| 344.42 | Monoplegia of Upper Limb, Affecting Nondominant Side |
| 344.5 | Monoplegia of Upper Limb, Unspecified Monoplegia |
| 344.6 | Cauda Equina Syndrome |
| 344.60 | Cauda Equina Syndrome without Mention of Neurogenic Bladder |
| 344.61 | Cauda Equina Syndrome with Neurogenic Bladder |
| 344.8 | Paralytic Syndromes, Other Specified |
| 344.81 | Paralytic Syndromes, Other Specified; Locked-in State |
| 344.89 | Paralytic Syndrome, Other Specified |
| 344.9 | Paralysis, Paraplegia, and Quadriplegia |
| 353 | Nerve Root and Plexus Disorder |
| 353.0 | Brachial Plexus Lesions; Cervical Rib Syndrome; Costoclavicular Syndrome; Scalenus Anticus Syndrome; Thoracic Outlet Syndrome |
| 353.1 | Lumbosacral plexus lesions |
| 353.2 | Cervical Root Lesions, Not Elsewhere Classified |
| 353.3 | Thoracic Root Lesions, Not Elsewhere Classified |
| 353.4 | Lumbosacral Root Lesions, Not Elsewhere Classified |
| 353.5 | Neuralgic Amyotrophy; Parsonage-Aldren-Turner Syndrome |
| 353.6 | Phantom Limb (Syndrome) |
| 353.8 | Nerve Root and Plexus Disorders, Other |
| 353.9 | Nerve Root and Plexus Disorder, Unspecified |
| 441.0 | Dissection of Aorta |
| 441.00 | Dissection of Aorta, Unspecified Site |
| 441.01 | Dissection of Aorta, Thoracic |
| 441.02 | Dissection of Aorta, Abdominal |
| 441.03 | Dissection of Aorta, Thoracoabdominal |
| 441.1 | Thoracic Aneurysm, Ruptured |
| 441.2 | Thoracic Aneurysm without Mention of Rupture |
| 441.3 | Abdominal Aneurysm, Ruptured |
| 441.4 | Abdominal Aneurysm without Mention of Rupture |
| 441.5 | Aortic Aneurysm of Unspecified Site, Ruptured |
| 441.6 | Thoracoabdominal Aneurysm, Ruptured, Ruptured |
| 441.7 | Thoracoabdominal Aneurysm |
| 441.9 | Aortic Aneurysm of Unspecified Site without Mention of Rupture |
| 442.0 | Aneurysm of Artery of Upper Extremity, Other |
| 442.1 | Aneurysm of Renal Artery, Other |
| 442.2 | Aneurysm of Iliac Artery, Other |
| 442.3 | Aneurysm of Artery of Lower Extremity, Other; Femoral Artery, Popliteal Artery |
| 442.81 | Aneurysm of Other Specified Artery, Other; Artery of Neck; Aneurysm of Carotid Artery (Common) (External) (Internal, Extracranial portion) |
| 442.82 | Aneurysm of Other Specified Artery, Other; Subclavian Artery |
| 442.83 | Aneurysm of Other Specified Artery, Other; Splenic Artery |
| 442.84 | Aneurysm of Other Specified Artery, Other; Other Visceral Artery; Celiac Artery; Gastroduodenal Artery; Gastroepiploic Artery; Hepatic Artery; Pancreaticoduodenal Artery; Superior Mesenteric Artery |
| 442.89 | Aneurysm of Other Specified Artery, Other; Other; Mediastinal Artery; Spinal Artery |
| 442.9 | Aneurysm of Other Specified Artery, Other; Of Unspecified Site |
| 443.21 | Dissection of Carotid Artery |
| 443.22 | Dissection of Iliac Artery |
| 443.23 | Dissection of Renal Artery |
| 443.24 | Dissection of Vertebral Artery |
| 443.29 | Dissection of Other Artery |
| 447.70 | Aortic Ectasia, Unspecified Site |
| 447.71 | Thoracic Aortic Ectasia |
| 447.72 | Abdominal Aortic Ectasia |
| 447.73 | Thoracoabdominal Aortic Ectasia |
| 720 | Ankylosing Spondylitis and Other Inflammatory Spondylopathies |
| 720.0 | Ankylosing Spondylitis |
| 720.1 | Spinal Enthesopathy; Disorder of Peripheral Ligamentous or Muscular Attachments of Spine; Romanus Lesion |
| 720.2 | Sacroiliitis, Not Elsewhere Classified; Inflammation of Sacroiliac Joint NOS |
| 720.8 | Other Inflammatory Spondylopathies |
| 720.81 | Inflammatory Spondylopathies in Diseases Classified Elsewhere |
| 720.89 | Other Inflammatory Spondylopathies, Other |
| 720.9 | Unspecified Inflammatory Spondylopathy; Spondylitis NOS |
| 733 | Other Disorders of Bone and Cartilage |
| 733.0 | Osteoporosis |
| 733.00 | Osteoporosis, Unspecified |
| 733.01 | Senile Osteoporosis; Postmenopausal Osteoporosis |
| 733.02 | Idiopathic Osteoporosis |
| 733.03 | Disuse Osteoporosis |
| 733.09 | Osteoporosis, Other; Drug-induced Osteoporosis |
| 733.1 | Pathologic Fracture; Spontaneous Fracture; Chronic fracture |
| 733.10 | Pathologic Fracture, Unspecified Site |
| 733.11 | Pathologic Fracture of Humerus |
| 733.12 | Pathologic Fracture of Distal Radius and Ulna; Wrist NOS |
| 733.13 | Pathological Fracture of Vertebra; Collapse of Vertebra, NOS |
| 733.14 | Pathologic Fracture of Neck of Femur; Femur NOS; Hip NOS |
| 733.15 | Pathologic Fracture of Other Specified Part of Femur |
| 733.16 | Pathologic Fracture of Tibia and Fibula, Ankle NOS |
| 733.19 | Pathologic Fracture of Other Specified Site |
| 733.2 | Cyst of Bone |
| 733.20 | Cyst of Bone (Localized), Unspecified |
| 733.21 | Solitary Bone Cyst; Unicameral Bone Cyst |
| 733.22 | Aneurysmal Bone Cyst |
| 733.29 | Cyst of Bone, Other; Fibrous Dysplasia (Monostotic) |
| 733.3 | Hyperostosis of Skull; Hyperostosis Interna Frontalis; Leontiasis Ossium |
| 733.4 | Aseptic Necrosis of Bone |
| 733.40 | Aseptic Necrosis of Bone, Site Unspecified |
| 733.41 | Aseptic Necrosis; Head of Humerus |
| 733.42 | Aseptic Necrosis; Head and Neck of Femur; Femur NOS |
| 733.43 | Aseptic Necrosis; Medial Femoral Condyle |
| 733.44 | Aseptic Necrosis; Talus |
| 733.45 | Aseptic Necrosis; Jaw |
| 733.49 | Aseptic Necrosis of Bone, Other |
| 733.5 | Osteitis Condensans; Piriform Sclerosis of Ilium |
| 733.6 | Tietze's Disease; Costochondral Junction Syndrome; Costochondritis |
| 733.7 | Algoneurodystrophy; Disuse Atrophy Of Bone; Sudeck's Atrophy |
| 733.8 | Malunion and Nonunion of Fracture |
| 733.81 | Malunion of Fracture |
| 733.82 | Nonunion of Fracture, Psedoarthrosis (Bone) |
| 733.9 | Other and Unspecified Disorders of Bone and Cartilage |
| 733.90 | Disorder of Bone and Cartilage, Unspecified |
| 733.91 | Arrest of Bone Development or Growth; Epiphyseal Arrest |
| 733.92 | Chondromalacia; Chondromalacia: NOS, Localized, Except Patella, Systemic, Tibial Plateau |
| 733.93 | Stress Fracture of Tibia or Fibula; Stress Reaction of Tibia or Fibula |
| 733.94 | Stress Fracture of The Metatarsals; Stress Reaction of Metatarsals |
| 733.95 | Stress Fracture of Other Bone; Stress Reaction of Other Bone |
| 733.96 | Stress fracture of femoral neck |
| 733.97 | Stress fracture of shaft of femur |
| 733.98 | Stress fracture of pelvis |
| 733.99 | Other and Unspecified Disorders of Bone and Cartilage, Other; Diaphysitis; Hypertrophy of Bone; Relapsing Polychondritis |
| 738 | Other Acquired Deformity |
| 738.0 | Acquired Deformity of Nose; Deformity Of Nose (Acquired); Overdevelopment of Nasal Bones |
| 738.1 | Other Acquired Deformity of Head |
| 738.10 | Unspecified Deformity |
| 738.11 | Zygomatic Hyperplasia |
| 738.12 | Zygomatic Hypoplasia |
| 738.19 | Other Specified Deformity |
| 738.2 | Acquired Deformity of Neck |
| 738.3 | Acquired Deformity of Chest and Rib; Deformity: Chest (Acquired), Rib (Acquired); Pectus: Carinatum, Acquired, Excavatum, Acquired |
| 738.4 | Acquired Spondylolisthesis; Degenerative Spondylolisthesis; Spondylolysis, Acquired |
| 738.5 | Other Acquired Deformity of Back or Spine; Deformity of Spine NOS |
| 738.6 | Acquired Deformity of Pelvis; Pelvic Obliquity |
| 738.7 | Cauliflower Ear |
| 738.8 | Acquired Deformity of Other Specified Site; Deformity of Clavicle |
| 738.9 | Acquired Deformity of Unspecified Site |
| 742.59 | Other Specified Anomalies of Spinal Cord; Other; Amyelia; Atelomyelia; Congenital Anomaly of Spinal Meninges; Defective Development of Cauda Equina; Hypoplasia of Spinal Cord; Myelatelia; Myelodysplasia |
| 767.4 | Birth Trauma; Injury to Spine and Spinal Cord; Dislocation of Spine or Spinal Cord Due to Birth Trauma; Fracture of Spine or Spinal Cord Due to Birth Trauma; Laceration of Spine or Spinal Cord Due to Birth Trauma; Rupture of Spine or Spinal Cord Due to Birth Trauma |
| 805 | Fracture of Vertebral Column without Mention of Spinal Cord Injury |
| 805.0 | Closed Fracture of Cervical Spine without Mention of Spinal Cord Injury |
| 805.00 | Closed Fracture of Cervical Spine, Unspecified Level, without Mention of Spinal Cord Injury |
| 805.01 | Closed Fracture of First Cervical Vertebra without Mention of Spinal Cord Injury |
| 805.02 | Closed Fracture of Second Cervical Vertebra without Mention of Spinal Cord Injury |
| 805.03 | Closed Fracture of Third Cervical Vertebra without Mention of Spinal Cord Injury |
| 805.04 | Closed Fracture of Fourth Cervical Vertebra without Mention of Spinal Cord Injury |
| 805.05 | Closed Fracture of Fifth Cervical Vertebra without Mention of Spinal Cord Injury |
| 805.06 | Closed Fracture of Sixth Cervical Vertebra without Mention of Spinal Cord Injury, Closed |
| 805.07 | Closed Fracture of Seventh Cervical Vertebra without Mention of Spinal Cord Injury, Closed |
| 805.08 | Closed Fracture of Multiple Cervical Vertebra without Mention of Spinal Cord Injury |
| 805.1 | Open Fracture of Cervical Spine without Mention of Spinal Cord Injury |
| 805.10 | Open Fracture of Cervical Spine, Unspecified Level, without Mention of Spinal Cord Injury |
| 805.11 | Open Fracture of First Cervical Vertebra without Mention of Spinal Cord Injury |
| 805.12 | Open Fracture of Second Cervical Vertebra without Mention of Spinal Cord Injury |
| 805.13 | Open Fracture of Third Cervical Vertebra without Mention of Spinal Cord Injury |
| 805.14 | Open Fracture of Fourth Cervical Vertebra without Mention of Spinal Cord Injury |
| 805.15 | Open Fracture of Fifth Cervical Vertebra without Mention of Spinal Cord Injury |
| 805.16 | Open Fracture of Sixth Cervical Vertebra without Mention of Spinal Cord Injury |
| 805.17 | Open Fracture of Seventh Cervical Vertebra without Mention of Spinal Cord Injury |
| 805.18 | Open Fracture of Multiple Cervical Vertebra without Mention of Spinal Cord Injury |
| 805.2 | Closed Fracture of Thoracic Spine without Mention of Spinal Cord Injury |
| 805.3 | Open Fracture of Thoracic Spine without Mention of Spinal Cord Injury |
| 805.4 | Closed Fracture of Lumbar Spine without Mention of Spinal Cord Injury |
| 805.5 | Open Fracture of Lumbar Spine without Mention of Spinal Cord Injury |
| 805.6 | Closed Fracture of Sacrum and Coccyx without Mention of Spinal Cord Injury |
| 805.7 | Open Fracture of Sacrum and Coccyx without Mention of Spinal Cord Injury |
| 805.8 | Closed Fracture of Vertebral Column, Unspecified, without Mention of Spinal Cord Injury |
| 805.9 | Open Fracture of Vertebral Column, Unspecified, without Mention of Spinal Cord Injury |
| 806 | Fracture of Vertebral Column with Spinal Cord Injury |
| 806.0 | Closed Fracture of Cervical Spine with Spinal Cord Injury |
| 806.00 | Closed Fracture of C1-C4 Level of Vertebral Column with Unspecified Spinal Cord Injury |
| 806.01 | Closed Fracture of C1-C4 Level of Vertebral Column with Complete Lesion of Cord |
| 806.02 | Closed Fracture of C1-C4 Level of Vertebral Column with Anterior Cord Syndrome |
| 806.03 | Closed Fracture of C1-C4 Level of Vertebral Column with Central Cord Syndrome |
| 806.04 | Closed Fracture of C1-C4 Level of Vertebral Column with Other Specified Spinal Cord Injury |
| 806.05 | Closed Fracture of C1-C4 Level of Vertebral Column with Unspecified Spinal Cord Injury |
| 806.06 | Closed Fracture of C5-C7 Level of Vertebral Column with Complete Lesion of Cord |
| 806.07 | Closed Fracture of C5-C7 Level of Vertebral Column with Anterior Cord Syndrome |
| 806.08 | Closed Fracture of C5-C7 Level of Vertebral Column with Central Cord Syndrome |
| 806.09 | Closed Fracture of C5-C7 Level of Vertebral Column with Other Specified Spinal Cord Injury |
| 806.1 | Open Fracture of Cervical Spine with Spinal Cord Injury |
| 806.10 | Open Fracture of C1-C4 Level of Vertebral Column with Unspecified Spinal Cord Injury |
| 806.11 | Open Fracture of C1-C4 Level of Vertebral Column with Complete Lesion of Cord |
| 806.12 | Open Fracture of C1-C4 Level of Vertebral Column with Anterior Cord Syndrome |
| 806.13 | Open Fracture of C1-C4 Level of Vertebral Column with Central Cord Syndrome |
| 806.14 | Open Fracture of C1-C4 Level of Vertebral Column with Other Specified Spinal Cord Injury |
| 806.15 | Open Fracture of C1-C4 Level of Vertebral Column with Unspecified Spinal Cord Injury |
| 806.16 | Open Fracture of C5-C7 Level of Vertebral Column with Complete Lesion of Cord |
| 806.17 | Open Fracture of C5-C7 Level of Vertebral Column with Anterior Cord Syndrome |
| 806.18 | Open Fracture of C5-C7 Level of Vertebral Column with Central Cord Syndrome |
| 806.19 | Open Fracture of C5-C7 Level of Vertebral Column with Other Specified Spinal Cord Injury |
| 806.2 | Closed Fracture of Thoracic Spine with Spinal Cord Injury |
| 806.20 | Closed Fracture of T1-T6 Level of Vertebral Column with Unspecified Spinal Cord Injury |
| 806.21 | Closed Fracture of T1-T6 Level of Vertebral Column with Complete Lesion of Cord |
| 806.22 | Closed Fracture of T1-T6 Level of Vertebral Column with Anterior Cord Syndrome |
| 806.23 | Closed Fracture of T1-T6 Level of Vertebral Column with Central Cord Syndrome |
| 806.24 | Closed Fracture of T1-T6 Level of Vertebral Column with Other Specified Spinal Cord Injury |
| 806.25 | Closed Fracture of T7-T12 Level of Vertebral Column with Unspecified Spinal Cord Injury |
| 806.26 | Closed Fracture of T7-T12 Level of Vertebral Column with Complete Lesion of Cord |
| 806.27 | Closed Fracture of T7-T12 Level of Vertebral Column with Anterior Cord Syndrome |
| 806.28 | Closed Fracture of T7-T12 Level of Vertebral Column with Central Cord Syndrome |
| 806.29 | Closed Fracture of T7-T12 Level of Vertebral Column with Other Specified Spinal Cord Injury |
| 806.3 | Open Fracture of Thoracic Spine with Spinal Cord Injury |
| 806.30 | Open Fracture of T1-T6 Level of Vertebral Column with Unspecified Spinal Cord Injury |
| 806.31 | Open Fracture of T1-T6 Level of Vertebral Column with Complete Lesion of Cord |
| 806.32 | Open Fracture of T1-T6 Level of Vertebral Column with Anterior Cord Syndrome |
| 806.33 | Open Fracture of T1-T6 Level of Vertebral Column with Central Cord Syndrome |
| 806.34 | Open Fracture of T1-T6 Level of Vertebral Column with Other Specified Spinal Cord Injury |
| 806.35 | Open Fracture of T7-T12 Level of Vertebral Column with Unspecified Spinal Cord Injury |
| 806.36 | Open Fracture of T7-T12 Level of Vertebral Column with Complete Lesion of Cord |
| 806.37 | Open Fracture of T7-T12 Level of Vertebral Column with Anterior Cord Syndrome |
| 806.38 | Open Fracture of T7-T12 Level of Vertebral Column with Central Cord Syndrome |
| 806.39 | Fracture, T7-T12 Level of Vertebral Column with Other Specified Spinal Cord Injury, Open |
| 806.4 | Closed Fracture of Lumbar Spine with Spinal Cord Injury |
| 806.5 | Open Fracture of Lumbar Spine with Spinal Cord Injury |
| 806.6 | Closed Fracture of Sacrum and Coccyx with Spinal Cord Injury |
| 806.60 | Closed Fracture of Sacrum and Coccyx with Unspecified Spinal Cord Injury |
| 806.61 | Closed Fracture of Sacrum and Coccyx with Complete Cauda Equina Lesion |
| 806.62 | Closed Fracture of Sacrum and Coccyx with Other Cauda Equina Lesion |
| 806.69 | Closed Fracture of Sacrum and Coccyx with Other Spinal Cord Injury |
| 806.7 | Open Fracture of Sacrum and Coccyx with Spinal Cord Injury |
| 806.70 | Open Fracture of Sacrum and Coccyx with Unspecified Spinal Cord Injury |
| 806.71 | Open Fracture of Sacrum and Coccyx with Complete Cauda Equina Lesion |
| 806.72 | Open Fracture of Sacrum and Coccyx with Other Cauda Equina Lesion |
| 806.79 | Open Fracture of Sacrum and Coccyx with Other Spinal Cord Injury |
| 806.8 | Closed Fracture of Vertebral Column with Spinal Cord Injury, Unspecified |
| 806.9 | Open Fracture of Vertebral Column with Spinal Cord Injury, Unspecified |
| 811.03 | Closed Fracture of Glenoid Cavity and Neck of Scapula |
| 811.13 | Open Fracture of Glenoid Cavity and Neck of Scapula |
| 812.01 | Closed Fracture of Surgical Neck of Humerus |
| 812.02 | Closed Fracture of Anatomical Neck of Humerus |
| 812.11 | Open Fracture of Surgical Neck of Humerus |
| 812.12 | Open Fracture of Anatomical Neck of Humerus |
| 813.06 | Closed Fracture of Neck of Radius |
| 813.16 | Open Fracture of Neck of Radius |
| 815.04 | Closed Fracture of Neck of Metacarpal Bone(s) |
| 815.14 | Open Fracture of Neck of Metacarpal Bone(s) |
| 820 | Fracture of Neck of Femur |
| 820.0 | Closed Transcervical Fracture of Neck of Femur |
| 820.00 | Closed Fracture of Intracapsular Section of Neck of Femur, Unspecified |
| 820.01 | Closed Fracture of Epiphysis of Neck of Femur |
| 820.02 | Closed Fracture of Mid-cervical Section of Neck of Femur |
| 820.03 | Closed Fracture of Base of Neck of Femur, Cervicotrochanteric Section |
| 820.09 | Closed Transcervical Fracture of Neck of Femur, Other |
| 820.1 | Open Transcervical Fracture of Neck of Femur |
| 820.10 | Open Fracture of Unspecified Intracapsular Section of Neck of Femur |
| 820.11 | Open Fracture of Epiphysis of Neck of Femur |
| 820.12 | Open Fracture of Mid-cervical Section of Neck of Femur |
| 820.13 | Open Fracture of Base of Neck of Femur |
| 820.19 | Open Transcervical Fracture of Femur, Other |
| 820.8 | Closed Fracture of Neck of Femur, Unspecified Part |
| 820.9 | Open Fracture of Neck of Femur, Unspecified Part |
| 839 | Dislocations, Other, Multiple, and Ill-defined |
| 839.0 | Dislocation of Cervical Vertebra, Closed; Cervical Spine; Neck |
| 839.00 | Dislocation of Cervical Vertebra, Closed; Cervical Spine; Neck, Cervical Vertebra, Unspecified |
| 839.01 | Dislocation of Cervical Vertebra, Closed; Cervical Spine; Neck, First Cervical Vertebra |
| 839.02 | Dislocation of Cervical Vertebra, Closed; Cervical Spine; Neck, Second Cervical Vertebra |
| 839.03 | Dislocation of Cervical Vertebra, Closed; Cervical Spine; Neck, Third Cervical Vertebra |
| 839.04 | Dislocation of Cervical Vertebra, Closed; Cervical Spine; Neck, Fourth Cervical Vertebra |
| 839.05 | Dislocation of Cervical Vertebra, Closed; Cervical Spine; Neck, Fifth Cervical Vertebra |
| 839.06 | Dislocation of Cervical Vertebra, Closed; Cervical Spine; Neck, Sixth Cervical Vertebra |
| 839.07 | Dislocation of Cervical Vertebra, Closed; Cervical Spine; Neck, Seventh Cervical Vertebra |
| 839.08 | Dislocation of Cervical Vertebra, Closed; Cervical Spine; Neck, Multiple Cervical Vertebrae |
| 839.1 | Dislocation of Cervical Vertebra, Open; Cervical Spine; Neck |
| 839.10 | Dislocation of Cervical Vertebra, Open; Cervical Spine; Neck, Cervical Vertebra, Unspecified |
| 839.11 | Dislocation of Cervical Vertebra, Open; Cervical Spine; Neck, First Cervical Vertebra |
| 839.12 | Dislocation of Cervical Vertebra, Open; Cervical Spine; Neck, Second Cervical Vertebra |
| 839.13 | Dislocation of Cervical Vertebra, Open; Cervical Spine; Neck, Third Cervical Vertebra |
| 839.14 | Dislocation of Cervical Vertebra, Open; Cervical Spine; Neck, Fourth Cervical Vertebra |
| 839.15 | Dislocation of Cervical Vertebra, Open; Cervical Spine; Neck, Fifth Cervical Vertebra |
| 839.16 | Dislocation of Cervical Vertebra, Open; Cervical Spine; Neck, Sixth Cervical Vertebra |
| 839.17 | Dislocation of Cervical Vertebra, Open; Cervical Spine; Neck, Seventh Cervical Vertebra |
| 839.18 | Dislocation of Cervical Vertebra, Open; Cervical Spine; Neck, Multiple Cervical Vertebrae |
| 839.2 | Dislocation of Cervical Vertebra, Thoracic and Lumbar Vertebra, Closed |
| 839.20 | Dislocation of Cervical Vertebra, Lumbar Vertebra, Closed |
| 839.21 | Dislocation of Cervical Vertebra, Thoracic Vertebra, Closed; Dorsal [Thoracic] Vertebra |
| 839.3 | Dislocation of Cervical Vertebra, Thoracic and Lumbar Vertebra, Open |
| 839.30 | Dislocation of Cervical Vertebra, Lumbar Vertebra, Open |
| 839.31 | Dislocation of Cervical Vertebra, Thoracic Vertebra, Open |
| 839.4 | Dislocations, Other, Multiple, and Ill-defined, Other Vertebra, Closed |
| 839.40 | Dislocations, Other, Multiple, and Ill-defined, Other Vertebra, Closed, Unspecified Site; Spine NOS |
| 839.41 | Dislocations, Other, Multiple, and Ill-defined, Other Vertebra, Closed, Coccyx |
| 839.42 | Dislocations, Other, Multiple, and Ill-defined, Other Vertebra, Closed, Sacrum; Sacroiliac (Joint) |
| 839.49 | Dislocations, Other, Multiple, and Ill-defined, Other vertebra, Closed, Other |
| 839.5 | Dislocations, Other, Multiple, and Ill-defined, Other Vertebra, Open |
| 839.50 | Dislocations, Other, Multiple, and Ill-defined, Other Vertebra, Open, Unspecified Site |
| 839.51 | Dislocations, Other, Multiple, and Ill-defined, Other Vertebra, Open, Coccyx |
| 839.52 | Dislocations, Other, Multiple, and Ill-defined, Other Vertebra, Open, Sacrum |
| 839.59 | Dislocations, Other, Multiple, and Ill-defined, Other Vertebra, Open, Other |
| 839.6 | Dislocations, Other, Multiple, and Ill-defined, Other Vertebra, Open, Other Location, Closed |
| 839.61 | Dislocations, Other, Multiple, and Ill-defined, Other Vertebra, Open, Other Location, Closed, Sternoclavicular Joint |
| 839.69 | Dislocations, Other, Multiple, and Ill-defined, Other Vertebra, Open, Other Location, Closed; Pelvis |
| 839.7 | Dislocations, Other, Multiple, and Ill-defined, Other Location, Open |
| 839.71 | Dislocations, Other, Multiple, and Ill-defined, Other Location, Open, Sternum |
| 839.79 | Dislocations, Other, Multiple, and Ill-defined, Other Location, Open, Other |
| 839.8 | Dislocation, Multiple and Ill-defined Sites, Closed; Arm; Back; Hand; Multiple Locations, except Fingers or Toes Alone |
| 839.9 | Dislocation, Multiple and Ill-defined Sites, Open |
| 876 | Open Wound, Back |
| 876.0 | Open Wound, Back without Mention of Complication |
| 876.1 | Open Wound, Back, Complicated |
| 877 | Open Wound, Buttock |
| 877.0 | Open Wound, Buttock, without Mention of Complication |
| 877.1 | Open Wound, Buttock, Complicated |
| 905.1 | Late Effect Of Fracture Of Spine And Trunk Without Mention Of Spinal Cord Lesion |
| 905.3 | Late Effect of Fracture of Neck of Femur |
| 926 | Crushing Injury of Trunk |
| 926.0 | Crushing Injury of External Genitalia; Labium (Majus) (Minus); Penis; Scrotum; Testis; Vulva |
| 926.1 | Crushing Injury of Other Specified Sites of Trunk |
| 926.11 | Crushing Injury of Back |
| 926.12 | Crushing Injury of Buttock |
| 926.19 | Crushing Injury of Trunk, Other |
| 926.8 | Crushing Injury of Multiple Sites of Trunk |
| 926.9 | Crushing Injury of Trunk, Unspecified Site |
| 952 | Spinal Cord Injury, without Evidence of Spinal Bone Injury |
| 952.0 | Cervical Spinal Cord Injury without Evidence of Spinal Bone Injury |
| 952.00 | Spinal Cord Injury, without Evidence of Spinal Bone Injury; C1-C4 Level with Unspecified Spinal Cord Injury |
| 952.01 | Spinal Cord Injury, without Evidence of Spinal Bone Injury; C1-C4 Level with Complete Lesion of Spinal Cord |
| 952.02 | Spinal Cord Injury, Cervical Spine with Anterior Cord Syndrome |
| 952.03 | Spinal Cord Injury, without Evidence of Spinal Bone Injury; C1-C4 level with Central Cord Syndrome |
| 952.04 | Spinal Cord Injury, without Evidence of Spinal Bone Injury; C1-C4 level with Other Specified Spinal Cord Injury |
| 952.05 | Spinal Cord Injury, without Evidence of Spinal Bone Injury; C5-C7 level with Unspecified Spinal Cord Injury |
| 952.06 | Spinal Cord Injury, without Evidence of Spinal Bone Injury; C5-C7 level with Complete Lesion Of Spinal Cord |
| 952.07 | Spinal Cord Injury, without Evidence of Spinal Bone Injury; C5-C7 level with Anterior Cord Syndrome |
| 952.08 | Spinal Cord Injury, without Evidence of Spinal Bone Injury; C5-C7 level with Central Cord Syndrome |
| 952.09 | Spinal Cord Injury, without Evidence of Spinal Bone Injury;C5-C7 level with Other Specified Spinal Cord Injury |
| 952.1 | Thoracic Spinal Cord Injury without Evidence of Spinal Bone Injury |
| 952.10 | Thoracic Spinal Cord Injury without Evidence of Spinal Bone Injury; T1-T6 Level with Unspecified Spinal Cord Injury |
| 952.11 | Thoracic Spinal Cord Injury without Evidence of Spinal Bone Injury; T1-T6 Level with Complete Lesion of Spinal Cord |
| 952.12 | Thoracic Spinal Cord Injury without Evidence of Spinal Bone Injury; T1-T6 Level with Anterior Cord Syndrome |
| 952.13 | Thoracic Spinal Cord Injury without Evidence of Spinal Bone Injury; T1-T6 Level with Central Cord Syndrome |
| 952.14 | Thoracic Spinal Cord Injury without Evidence of Spinal Bone Injury; T1-T6 Level with Other Specified Spinal Cord Injury |
| 952.15 | Thoracic Spinal Cord Injury without Evidence of Spinal Bone Injury; T7-T12 Level with Unspecified Spinal Cord Injury |
| 952.16 | Thoracic Spinal Cord Injury without Evidence of Spinal Bone Injury; T7-T12 Level with Complete Lesion of Spinal Cord |
| 952.17 | Thoracic Spinal Cord Injury without Evidence of Spinal Bone Injury; T7-T12 Level with Anterior Cord Syndrome |
| 952.18 | Thoracic Spinal Cord Injury without Evidence of Spinal Bone Injury; T7-T12 Level with Central Cord Syndrome |
| 952.19 | Thoracic Spinal Cord Injury without Evidence of Spinal Bone Injury; T7-T12 Level with Other Specified Spinal Cord Injury |
| 952.2 | Lumbar Spinal Cord Injury without Evidence of Spinal Bone Injury |
| 952.3 | Sacral Spinal Cord Injury without Evidence of Spinal Bone Injury |
| 952.4 | Injury to Nerves and Spinal Cord, Cauda Equina |
| 952.8 | Spinal Cord Injury without Evidence of Spinal Bone Injury, Multiple Sites |
| 952.9 | Spinal Cord Injury, Unspecified |
| 953 | Injury to Nerve Roots and Spinal Plexus |
| 953.0 | Injury to Nerve Roots and Spinal Plexus; Cervical Root |
| 953.1 | Injury to Nerve Roots and Spinal Plexus; Dorsal Root |
| 953.2 | Injury to Nerve Roots and Spinal Plexus; Lumbar Root |
| 953.3 | Injury to Nerve Roots and Spinal Plexus; Sacral Root |
| 953.4 | Injury to Nerve Roots and Spinal Plexus; Brachial Plexus Injury |
| 953.5 | Injury to Nerve Roots and Spinal Plexus; Lumbosacral Plexus |
| 953.8 | Injury to Nerve Roots and Spinal Plexus; Multiple Sites |
| 953.9 | Injury to Nerve Roots and Spinal Plexus; Unspecified Site |
| A00 | Cholera |
| A00.0 | Cholera due to Vibrio cholerae 01, biovar cholerae |
| A00.1 | Cholera due to Vibrio cholerae 01, biovar eltor |
| A00.9 | Cholera, unspecified |
| A01 | Typhoid and paratyphoid fevers |
| A01.0 | Typhoid fever |
| A01.00 | Typhoid fever, unspecified |
| A01.01 | Typhoid meningitis |
| A01.02 | Typhoid fever with heart involvement |
| A01.03 | Typhoid pneumonia |
| A01.04 | Typhoid arthritis |
| A01.05 | Typhoid osteomyelitis |
| A01.09 | Typhoid fever with other complications |
| A01.1 | Paratyphoid fever A |
| A01.2 | Paratyphoid fever B |
| A01.3 | Paratyphoid fever C |
| A01.4 | Paratyphoid fever, unspecified |
| A02 | Other salmonella infections |
| A02.0 | Salmonella enteritis |
| A02.1 | Salmonella sepsis |
| A02.2 | Localized salmonella infections |
| A02.20 | Localized salmonella infection, unspecified |
| A02.21 | Salmonella meningitis |
| A02.22 | Salmonella pneumonia |
| A02.23 | Salmonella arthritis |
| A02.24 | Salmonella osteomyelitis |
| A02.25 | Salmonella pyelonephritis |
| A02.29 | Salmonella with other localized infection |
| A02.8 | Other specified salmonella infections |
| A02.9 | Salmonella infection, unspecified |
| A03 | Shigellosis |
| A03.0 | Shigellosis due to Shigella dysenteriae |
| A03.1 | Shigellosis due to Shigella flexneri |
| A03.2 | Shigellosis due to Shigella boydii |
| A03.3 | Shigellosis due to Shigella sonnei |
| A03.8 | Other shigellosis |
| A03.9 | Shigellosis, unspecified |
| A04 | Other bacterial intestinal infections |
| A04.0 | Enteropathogenic Escherichia coli infection |
| A04.1 | Enterotoxigenic Escherichia coli infection |
| A04.2 | Enteroinvasive Escherichia coli infection |
| A04.3 | Enterohemorrhagic Escherichia coli infection |
| A04.4 | Other intestinal Escherichia coli infections |
| A04.5 | Campylobacter enteritis |
| A04.6 | Enteritis due to Yersinia enterocolitica |
| A04.7 | Enterocolitis due to Clostridium difficile |
| A04.71 | Enterocolitis due to Clostridium difficile, recurrent |
| A04.72 | Enterocolitis due to Clostridium difficile, not specified as recurrent |
| A04.8 | Other specified bacterial intestinal infections |
| A04.9 | Bacterial intestinal infection, unspecified |
| A05 | Other bacterial foodborne intoxications, not elsewhere classified |
| A05.0 | Foodborne staphylococcal intoxication |
| A05.1 | Botulism food poisoning |
| A05.2 | Foodborne Clostridium perfringens [Clostridium welchii] intoxication |
| A05.3 | Foodborne Vibrio parahaemolyticus intoxication |
| A05.4 | Foodborne Bacillus cereus intoxication |
| A05.5 | Foodborne Vibrio vulnificus intoxication |
| A05.8 | Other specified bacterial foodborne intoxications |
| A05.9 | Bacterial foodborne intoxication, unspecified |
| A06 | Amebiasis |
| A06.0 | Acute amebic dysentery |
| A06.1 | Chronic intestinal amebiasis |
| A06.2 | Amebic nondysenteric colitis |
| A06.3 | Ameboma of intestine |
| A06.4 | Amebic liver abscess |
| A06.5 | Amebic lung abscess |
| A06.6 | Amebic brain abscess |
| A06.7 | Cutaneous amebiasis |
| A06.8 | Amebic infection of other sites |
| A06.81 | Amebic cystitis |
| A06.82 | Other amebic genitourinary infections |
| A06.89 | Other amebic infections |
| A06.9 | Amebiasis, unspecified |
| A07 | Other protozoal intestinal diseases |
| A07.0 | Balantidiasis |
| A07.1 | Giardiasis [lambliasis] |
| A07.2 | Cryptosporidiosis |
| A07.3 | Isosporiasis |
| A07.4 | Cyclosporiasis |
| A07.8 | Other specified protozoal intestinal diseases |
| A07.9 | Protozoal intestinal disease, unspecified |
| A08 | Viral and other specified intestinal infections |
| A08.0 | Rotaviral enteritis |
| A08.1 | Acute gastroenteropathy due to Norwalk agent and other small round viruses |
| A08.11 | Acute gastroenteropathy due to Norwalk agent |
| A08.19 | Acute gastroenteropathy due to other small round viruses |
| A08.2 | Adenoviral enteritis |
| A08.3 | Other viral enteritis |
| A08.31 | Calicivirus enteritis |
| A08.32 | Astrovirus enteritis |
| A08.39 | Other viral enteritis |
| A08.4 | Viral intestinal infection, unspecified |
| A08.8 | Other specified intestinal infections |
| A09 | Infectious gastroenteritis and colitis, unspecified |
| A15 | Respiratory tuberculosis |
| A15.0 | Tuberculosis of lung |
| A15.4 | Tuberculosis of intrathoracic lymph nodes |
| A15.5 | Tuberculosis of larynx, trachea and bronchus |
| A15.6 | Tuberculous pleurisy |
| A15.7 | Primary respiratory tuberculosis |
| A15.8 | Other respiratory tuberculosis |
| A15.9 | Respiratory tuberculosis unspecified |
| A17 | Tuberculosis of nervous system |
| A17.0 | Tuberculous meningitis |
| A17.1 | Meningeal tuberculoma |
| A17.8 | Other tuberculosis of nervous system |
| A17.81 | Tuberculoma of brain and spinal cord |
| A17.82 | Tuberculous meningoencephalitis |
| A17.83 | Tuberculous neuritis |
| A17.89 | Other tuberculosis of nervous system |
| A17.9 | Tuberculosis of nervous system, unspecified |
| A18 | Tuberculosis of other organs |
| A18.0 | Tuberculosis of bones and joints |
| A18.01 | Tuberculosis of spine |
| A18.02 | Tuberculous arthritis of other joints |
| A18.03 | Tuberculosis of other bones |
| A18.09 | Other musculoskeletal tuberculosis |
| A18.1 | Tuberculosis of genitourinary system |
| A18.10 | Tuberculosis of genitourinary system, unspecified |
| A18.11 | Tuberculosis of kidney and ureter |
| A18.12 | Tuberculosis of bladder |
| A18.13 | Tuberculosis of other urinary organs |
| A18.14 | Tuberculosis of prostate |
| A18.15 | Tuberculosis of other male genital organs |
| A18.16 | Tuberculosis of cervix |
| A18.17 | Tuberculous female pelvic inflammatory disease |
| A18.18 | Tuberculosis of other female genital organs |
| A18.2 | Tuberculous peripheral lymphadenopathy |
| A18.3 | Tuberculosis of intestines, peritoneum and mesenteric glands |
| A18.31 | Tuberculous peritonitis |
| A18.32 | Tuberculous enteritis |
| A18.39 | Retroperitoneal tuberculosis |
| A18.4 | Tuberculosis of skin and subcutaneous tissue |
| A18.5 | Tuberculosis of eye |
| A18.50 | Tuberculosis of eye, unspecified |
| A18.51 | Tuberculous episcleritis |
| A18.52 | Tuberculous keratitis |
| A18.53 | Tuberculous chorioretinitis |
| A18.54 | Tuberculous iridocyclitis |
| A18.59 | Other tuberculosis of eye |
| A18.6 | Tuberculosis of (inner) (middle) ear |
| A18.7 | Tuberculosis of adrenal glands |
| A18.8 | Tuberculosis of other specified organs |
| A18.81 | Tuberculosis of thyroid gland |
| A18.82 | Tuberculosis of other endocrine glands |
| A18.83 | Tuberculosis of digestive tract organs, not elsewhere classified |
| A18.84 | Tuberculosis of heart |
| A18.85 | Tuberculosis of spleen |
| A18.89 | Tuberculosis of other sites |
| A19 | Miliary tuberculosis |
| A19.0 | Acute miliary tuberculosis of a single specified site |
| A19.1 | Acute miliary tuberculosis of multiple sites |
| A19.2 | Acute miliary tuberculosis, unspecified |
| A19.8 | Other miliary tuberculosis |
| A19.9 | Miliary tuberculosis, unspecified |
| A20 | Plague |
| A20.0 | Bubonic plague |
| A20.1 | Cellulocutaneous plague |
| A20.2 | Pneumonic plague |
| A20.3 | Plague meningitis |
| A20.7 | Septicemic plague |
| A20.8 | Other forms of plague |
| A20.9 | Plague, unspecified |
| A21 | Tularemia |
| A21.0 | Ulceroglandular tularemia |
| A21.1 | Oculoglandular tularemia |
| A21.2 | Pulmonary tularemia |
| A21.3 | Gastrointestinal tularemia |
| A21.7 | Generalized tularemia |
| A21.8 | Other forms of tularemia |
| A21.9 | Tularemia, unspecified |
| A22 | Anthrax |
| A22.0 | Cutaneous anthrax |
| A22.1 | Pulmonary anthrax |
| A22.2 | Gastrointestinal anthrax |
| A22.7 | Anthrax sepsis |
| A22.8 | Other forms of anthrax |
| A22.9 | Anthrax, unspecified |
| A23 | Brucellosis |
| A23.0 | Brucellosis due to Brucella melitensis |
| A23.1 | Brucellosis due to Brucella abortus |
| A23.2 | Brucellosis due to Brucella suis |
| A23.3 | Brucellosis due to Brucella canis |
| A23.8 | Other brucellosis |
| A23.9 | Brucellosis, unspecified |
| A24 | Glanders and melioidosis |
| A24.0 | Glanders |
| A24.1 | Acute and fulminating melioidosis |
| A24.2 | Subacute and chronic melioidosis |
| A24.3 | Other melioidosis |
| A24.9 | Melioidosis, unspecified |
| A25 | Rat-bite fevers |
| A25.0 | Spirillosis |
| A25.1 | Streptobacillosis |
| A25.9 | Rat-bite fever, unspecified |
| A26 | Erysipeloid |
| A26.0 | Cutaneous erysipeloid |
| A26.7 | Erysipelothrix sepsis |
| A26.8 | Other forms of erysipeloid |
| A26.9 | Erysipeloid, unspecified |
| A27 | Leptospirosis |
| A27.0 | Leptospirosis icterohemorrhagica |
| A27.8 | Other forms of leptospirosis |
| A27.81 | Aseptic meningitis in leptospirosis |
| A27.89 | Other forms of leptospirosis |
| A27.9 | Leptospirosis, unspecified |
| A28 | Other zoonotic bacterial diseases, not elsewhere classified |
| A28.0 | Pasteurellosis |
| A28.1 | Cat-scratch disease |
| A28.2 | Extraintestinal yersiniosis |
| A28.8 | Other specified zoonotic bacterial diseases, not elsewhere classified |
| A28.9 | Zoonotic bacterial disease, unspecified |
| A30 | Leprosy [Hansen's disease] |
| A30.0 | Indeterminate leprosy |
| A30.1 | Tuberculoid leprosy |
| A30.2 | Borderline tuberculoid leprosy |
| A30.3 | Borderline leprosy |
| A30.4 | Borderline lepromatous leprosy |
| A30.5 | Lepromatous leprosy |
| A30.8 | Other forms of leprosy |
| A30.9 | Leprosy, unspecified |
| A31 | Infection due to other mycobacteria |
| A31.0 | Pulmonary mycobacterial infection |
| A31.1 | Cutaneous mycobacterial infection |
| A31.2 | Disseminated mycobacterium avium-intracellulare complex (DMAC) |
| A31.8 | Other mycobacterial infections |
| A31.9 | Mycobacterial infection, unspecified |
| A32 | Listeriosis |
| A32.0 | Cutaneous listeriosis |
| A32.1 | Listerial meningitis and meningoencephalitis |
| A32.11 | Listerial meningitis |
| A32.12 | Listerial meningoencephalitis |
| A32.7 | Listerial sepsis |
| A32.8 | Other forms of listeriosis |
| A32.81 | Oculoglandular listeriosis |
| A32.82 | Listerial endocarditis |
| A32.89 | Other forms of listeriosis |
| A32.9 | Listeriosis, unspecified |
| A33 | Tetanus neonatorum |
| A34 | Obstetrical tetanus |
| A35 | Other tetanus |
| A36 | Diphtheria |
| A36.0 | Pharyngeal diphtheria |
| A36.1 | Nasopharyngeal diphtheria |
| A36.2 | Laryngeal diphtheria |
| A36.3 | Cutaneous diphtheria |
| A36.8 | Other diphtheria |
| A36.81 | Diphtheritic cardiomyopathy |
| A36.82 | Diphtheritic radiculomyelitis |
| A36.83 | Diphtheritic polyneuritis |
| A36.84 | Diphtheritic tubulo-interstitial nephropathy |
| A36.85 | Diphtheritic cystitis |
| A36.86 | Diphtheritic conjunctivitis |
| A36.89 | Other diphtheritic complications |
| A36.9 | Diphtheria, unspecified |
| A37 | Whooping cough |
| A37.0 | Whooping cough due to Bordetella pertussis |
| A37.00 | Whooping cough due to Bordetella pertussis without pneumonia |
| A37.01 | Whooping cough due to Bordetella pertussis with pneumonia |
| A37.1 | Whooping cough due to Bordetella parapertussis |
| A37.10 | Whooping cough due to Bordetella parapertussis without pneumonia |
| A37.11 | Whooping cough due to Bordetella parapertussis with pneumonia |
| A37.8 | Whooping cough due to other Bordetella species |
| A37.80 | Whooping cough due to other Bordetella species without pneumonia |
| A37.81 | Whooping cough due to other Bordetella species with pneumonia |
| A37.9 | Whooping cough, unspecified species |
| A37.90 | Whooping cough, unspecified species without pneumonia |
| A37.91 | Whooping cough, unspecified species with pneumonia |
| A38 | Scarlet fever |
| A38.0 | Scarlet fever with otitis media |
| A38.1 | Scarlet fever with myocarditis |
| A38.8 | Scarlet fever with other complications |
| A38.9 | Scarlet fever, uncomplicated |
| A39 | Meningococcal infection |
| A39.0 | Meningococcal meningitis |
| A39.1 | Waterhouse-Friderichsen syndrome |
| A39.2 | Acute meningococcemia |
| A39.3 | Chronic meningococcemia |
| A39.4 | Meningococcemia, unspecified |
| A39.5 | Meningococcal heart disease |
| A39.50 | Meningococcal carditis, unspecified |
| A39.51 | Meningococcal endocarditis |
| A39.52 | Meningococcal myocarditis |
| A39.53 | Meningococcal pericarditis |
| A39.8 | Other meningococcal infections |
| A39.81 | Meningococcal encephalitis |
| A39.82 | Meningococcal retrobulbar neuritis |
| A39.83 | Meningococcal arthritis |
| A39.84 | Postmeningococcal arthritis |
| A39.89 | Other meningococcal infections |
| A39.9 | Meningococcal infection, unspecified |
| A40 | Streptococcal sepsis |
| A40.0 | Sepsis due to streptococcus, group A |
| A40.1 | Sepsis due to streptococcus, group B |
| A40.3 | Sepsis due to Streptococcus pneumoniae |
| A40.8 | Other streptococcal sepsis |
| A40.9 | Streptococcal sepsis, unspecified |
| A41 | Other sepsis |
| A41.0 | Sepsis due to Staphylococcus aureus |
| A41.01 | Sepsis due to Methicillin susceptible Staphylococcus aureus |
| A41.02 | Sepsis due to Methicillin resistant Staphylococcus aureus |
| A41.1 | Sepsis due to other specified staphylococcus |
| A41.2 | Sepsis due to unspecified staphylococcus |
| A41.3 | Sepsis due to Hemophilus influenzae |
| A41.4 | Sepsis due to anaerobes |
| A41.5 | Sepsis due to other Gram-negative organisms |
| A41.50 | Gram-negative sepsis, unspecified |
| A41.51 | Sepsis due to Escherichia coli [E. coli] |
| A41.52 | Sepsis due to Pseudomonas |
| A41.53 | Sepsis due to Serratia |
| A41.59 | Other Gram-negative sepsis |
| A41.8 | Other specified sepsis |
| A41.81 | Sepsis due to Enterococcus |
| A41.89 | Other specified sepsis |
| A41.9 | Sepsis, unspecified organism |
| A42 | Actinomycosis |
| A42.0 | Pulmonary actinomycosis |
| A42.1 | Abdominal actinomycosis |
| A42.2 | Cervicofacial actinomycosis |
| A42.7 | Actinomycotic sepsis |
| A42.8 | Other forms of actinomycosis |
| A42.81 | Actinomycotic meningitis |
| A42.82 | Actinomycotic encephalitis |
| A42.89 | Other forms of actinomycosis |
| A42.9 | Actinomycosis, unspecified |
| A43 | Nocardiosis |
| A43.0 | Pulmonary nocardiosis |
| A43.1 | Cutaneous nocardiosis |
| A43.8 | Other forms of nocardiosis |
| A43.9 | Nocardiosis, unspecified |
| A44 | Bartonellosis |
| A44.0 | Systemic bartonellosis |
| A44.1 | Cutaneous and mucocutaneous bartonellosis |
| A44.8 | Other forms of bartonellosis |
| A44.9 | Bartonellosis, unspecified |
| A46 | Erysipelas |
| A48 | Other bacterial diseases, not elsewhere classified |
| A48.0 | Gas gangrene |
| A48.1 | Legionnaires' disease |
| A48.2 | Nonpneumonic Legionnaires' disease [Pontiac fever] |
| A48.3 | Toxic shock syndrome |
| A48.4 | Brazilian purpuric fever |
| A48.5 | Other specified botulism |
| A48.51 | Infant botulism |
| A48.52 | Wound botulism |
| A48.8 | Other specified bacterial diseases |
| A49 | Bacterial infection of unspecified site |
| A49.0 | Staphylococcal infection, unspecified site |
| A49.01 | Methicillin susceptible Staphylococcus aureus infection, unspecified site |
| A49.02 | Methicillin resistant Staphylococcus aureus infection, unspecified site |
| A49.1 | Streptococcal infection, unspecified site |
| A49.2 | Hemophilus influenzae infection, unspecified site |
| A49.3 | Mycoplasma infection, unspecified site |
| A49.8 | Other bacterial infections of unspecified site |
| A49.9 | Bacterial infection, unspecified |
| A50 | Congenital syphilis |
| A50.0 | Early congenital syphilis, symptomatic |
| A50.01 | Early congenital syphilitic oculopathy |
| A50.02 | Early congenital syphilitic osteochondropathy |
| A50.03 | Early congenital syphilitic pharyngitis |
| A50.04 | Early congenital syphilitic pneumonia |
| A50.05 | Early congenital syphilitic rhinitis |
| A50.06 | Early cutaneous congenital syphilis |
| A50.07 | Early mucocutaneous congenital syphilis |
| A50.08 | Early visceral congenital syphilis |
| A50.09 | Other early congenital syphilis, symptomatic |
| A50.1 | Early congenital syphilis, latent |
| A50.2 | Early congenital syphilis, unspecified |
| A50.3 | Late congenital syphilitic oculopathy |
| A50.30 | Late congenital syphilitic oculopathy, unspecified |
| A50.31 | Late congenital syphilitic interstitial keratitis |
| A50.32 | Late congenital syphilitic chorioretinitis |
| A50.39 | Other late congenital syphilitic oculopathy |
| A50.4 | Late congenital neurosyphilis [juvenile neurosyphilis] |
| A50.40 | Late congenital neurosyphilis, unspecified |
| A50.41 | Late congenital syphilitic meningitis |
| A50.42 | Late congenital syphilitic encephalitis |
| A50.43 | Late congenital syphilitic polyneuropathy |
| A50.44 | Late congenital syphilitic optic nerve atrophy |
| A50.45 | Juvenile general paresis |
| A50.49 | Other late congenital neurosyphilis |
| A50.5 | Other late congenital syphilis, symptomatic |
| A50.51 | Clutton's joints |
| A50.52 | Hutchinson's teeth |
| A50.53 | Hutchinson's triad |
| A50.54 | Late congenital cardiovascular syphilis |
| A50.55 | Late congenital syphilitic arthropathy |
| A50.56 | Late congenital syphilitic osteochondropathy |
| A50.57 | Syphilitic saddle nose |
| A50.59 | Other late congenital syphilis, symptomatic |
| A50.6 | Late congenital syphilis, latent |
| A50.7 | Late congenital syphilis, unspecified |
| A50.9 | Congenital syphilis, unspecified |
| A51 | Early syphilis |
| A51.0 | Primary genital syphilis |
| A51.1 | Primary anal syphilis |
| A51.2 | Primary syphilis of other sites |
| A51.3 | Secondary syphilis of skin and mucous membranes |
| A51.31 | Condyloma latum |
| A51.32 | Syphilitic alopecia |
| A51.39 | Other secondary syphilis of skin |
| A51.4 | Other secondary syphilis |
| A51.41 | Secondary syphilitic meningitis |
| A51.42 | Secondary syphilitic female pelvic disease |
| A51.43 | Secondary syphilitic oculopathy |
| A51.44 | Secondary syphilitic nephritis |
| A51.45 | Secondary syphilitic hepatitis |
| A51.46 | Secondary syphilitic osteopathy |
| A51.49 | Other secondary syphilitic conditions |
| A51.5 | Early syphilis, latent |
| A51.9 | Early syphilis, unspecified |
| A52 | Late syphilis |
| A52.0 | Cardiovascular and cerebrovascular syphilis |
| A52.00 | Cardiovascular syphilis, unspecified |
| A52.01 | Syphilitic aneurysm of aorta |
| A52.02 | Syphilitic aortitis |
| A52.03 | Syphilitic endocarditis |
| A52.04 | Syphilitic cerebral arteritis |
| A52.05 | Other cerebrovascular syphilis |
| A52.06 | Other syphilitic heart involvement |
| A52.09 | Other cardiovascular syphilis |
| A52.1 | Symptomatic neurosyphilis |
| A52.10 | Symptomatic neurosyphilis, unspecified |
| A52.11 | Tabes dorsalis |
| A52.12 | Other cerebrospinal syphilis |
| A52.13 | Late syphilitic meningitis |
| A52.14 | Late syphilitic encephalitis |
| A52.15 | Late syphilitic neuropathy |
| A52.16 | Charcot's arthropathy (tabetic) |
| A52.17 | General paresis |
| A52.19 | Other symptomatic neurosyphilis |
| A52.2 | Asymptomatic neurosyphilis |
| A52.3 | Neurosyphilis, unspecified |
| A52.7 | Other symptomatic late syphilis |
| A52.71 | Late syphilitic oculopathy |
| A52.72 | Syphilis of lung and bronchus |
| A52.73 | Symptomatic late syphilis of other respiratory organs |
| A52.74 | Syphilis of liver and other viscera |
| A52.75 | Syphilis of kidney and ureter |
| A52.76 | Other genitourinary symptomatic late syphilis |
| A52.77 | Syphilis of bone and joint |
| A52.78 | Syphilis of other musculoskeletal tissue |
| A52.79 | Other symptomatic late syphilis |
| A52.8 | Late syphilis, latent |
| A52.9 | Late syphilis, unspecified |
| A53 | Other and unspecified syphilis |
| A53.0 | Latent syphilis, unspecified as early or late |
| A53.9 | Syphilis, unspecified |
| A54 | Gonococcal infection |
| A54.0 | Gonococcal infection of lower genitourinary tract without periurethral or accessory gland abscess |
| A54.00 | Gonococcal infection of lower genitourinary tract, unspecified |
| A54.01 | Gonococcal cystitis and urethritis, unspecified |
| A54.02 | Gonococcal vulvovaginitis, unspecified |
| A54.03 | Gonococcal cervicitis, unspecified |
| A54.09 | Other gonococcal infection of lower genitourinary tract |
| A54.1 | Gonococcal infection of lower genitourinary tract with periurethral and accessory gland abscess |
| A54.2 | Gonococcal pelviperitonitis and other gonococcal genitourinary infection |
| A54.21 | Gonococcal infection of kidney and ureter |
| A54.22 | Gonococcal prostatitis |
| A54.23 | Gonococcal infection of other male genital organs |
| A54.24 | Gonococcal female pelvic inflammatory disease |
| A54.29 | Other gonococcal genitourinary infections |
| A54.3 | Gonococcal infection of eye |
| A54.30 | Gonococcal infection of eye, unspecified |
| A54.31 | Gonococcal conjunctivitis |
| A54.32 | Gonococcal iridocyclitis |
| A54.33 | Gonococcal keratitis |
| A54.39 | Other gonococcal eye infection |
| A54.4 | Gonococcal infection of musculoskeletal system |
| A54.40 | Gonococcal infection of musculoskeletal system, unspecified |
| A54.41 | Gonococcal spondylopathy |
| A54.42 | Gonococcal arthritis |
| A54.43 | Gonococcal osteomyelitis |
| A54.49 | Gonococcal infection of other musculoskeletal tissue |
| A54.5 | Gonococcal pharyngitis |
| A54.6 | Gonococcal infection of anus and rectum |
| A54.8 | Other gonococcal infections |
| A54.81 | Gonococcal meningitis |
| A54.82 | Gonococcal brain abscess |
| A54.83 | Gonococcal heart infection |
| A54.84 | Gonococcal pneumonia |
| A54.85 | Gonococcal peritonitis |
| A54.86 | Gonococcal sepsis |
| A54.89 | Other gonococcal infections |
| A54.9 | Gonococcal infection, unspecified |
| A55 | Chlamydial lymphogranuloma (venereum) |
| A56 | Other sexually transmitted chlamydial diseases |
| A56.0 | Chlamydial infection of lower genitourinary tract |
| A56.00 | Chlamydial infection of lower genitourinary tract, unspecified |
| A56.01 | Chlamydial cystitis and urethritis |
| A56.02 | Chlamydial vulvovaginitis |
| A56.09 | Other chlamydial infection of lower genitourinary tract |
| A56.1 | Chlamydial infection of pelviperitoneum and other genitourinary organs |
| A56.11 | Chlamydial female pelvic inflammatory disease |
| A56.19 | Other chlamydial genitourinary infection |
| A56.2 | Chlamydial infection of genitourinary tract, unspecified |
| A56.3 | Chlamydial infection of anus and rectum |
| A56.4 | Chlamydial infection of pharynx |
| A56.8 | Sexually transmitted chlamydial infection of other sites |
| A57 | Chancroid |
| A58 | Granuloma inguinale |
| A59 | Trichomoniasis |
| A59.0 | Urogenital trichomoniasis |
| A59.00 | Urogenital trichomoniasis, unspecified |
| A59.01 | Trichomonal vulvovaginitis |
| A59.02 | Trichomonal prostatitis |
| A59.03 | Trichomonal cystitis and urethritis |
| A59.09 | Other urogenital trichomoniasis |
| A59.8 | Trichomoniasis of other sites |
| A59.9 | Trichomoniasis, unspecified |
| A60 | Anogenital herpesviral [herpes simplex] infections |
| A60.0 | Herpesviral infection of genitalia and urogenital tract |
| A60.00 | Herpesviral infection of urogenital system, unspecified |
| A60.01 | Herpesviral infection of penis |
| A60.02 | Herpesviral infection of other male genital organs |
| A60.03 | Herpesviral cervicitis |
| A60.04 | Herpesviral vulvovaginitis |
| A60.09 | Herpesviral infection of other urogenital tract |
| A60.1 | Herpesviral infection of perianal skin and rectum |
| A60.9 | Anogenital herpesviral infection, unspecified |
| A63 | Other predominantly sexually transmitted diseases, not elsewhere classified |
| A63.0 | Anogenital (venereal) warts |
| A63.8 | Other specified predominantly sexually transmitted diseases |
| A64 | Unspecified sexually transmitted disease |
| A65 | Nonvenereal syphilis |
| A66 | Yaws |
| A66.0 | Initial lesions of yaws |
| A66.1 | Multiple papillomata and wet crab yaws |
| A66.2 | Other early skin lesions of yaws |
| A66.3 | Hyperkeratosis of yaws |
| A66.4 | Gummata and ulcers of yaws |
| A66.5 | Gangosa |
| A66.6 | Bone and joint lesions of yaws |
| A66.7 | Other manifestations of yaws |
| A66.8 | Latent yaws |
| A66.9 | Yaws, unspecified |
| A67 | Pinta [carate] |
| A67.0 | Primary lesions of pinta |
| A67.1 | Intermediate lesions of pinta |
| A67.2 | Late lesions of pinta |
| A67.3 | Mixed lesions of pinta |
| A67.9 | Pinta, unspecified |
| A68 | Relapsing fevers |
| A68.0 | Louse-borne relapsing fever |
| A68.1 | Tick-borne relapsing fever |
| A68.9 | Relapsing fever, unspecified |
| A69 | Other spirochetal infections |
| A69.0 | Necrotizing ulcerative stomatitis |
| A69.1 | Other Vincent's infections |
| A69.2 | Lyme disease |
| A69.20 | Lyme disease, unspecified |
| A69.21 | Meningitis due to Lyme disease |
| A69.22 | Other neurologic disorders in Lyme disease |
| A69.23 | Arthritis due to Lyme disease |
| A69.29 | Other conditions associated with Lyme disease |
| A69.8 | Other specified spirochetal infections |
| A69.9 | Spirochetal infection, unspecified |
| A70 | Chlamydia psittaci infections |
| A71 | Trachoma |
| A71.0 | Initial stage of trachoma |
| A71.1 | Active stage of trachoma |
| A71.9 | Trachoma, unspecified |
| A74 | Other diseases caused by chlamydiae |
| A74.0 | Chlamydial conjunctivitis |
| A74.8 | Other chlamydial diseases |
| A74.81 | Chlamydial peritonitis |
| A74.89 | Other chlamydial diseases |
| A74.9 | Chlamydial infection, unspecified |
| A75 | Typhus fever |
| A75.0 | Epidemic louse-borne typhus fever due to Rickettsia prowazekii |
| A75.1 | Recrudescent typhus [Brill's disease] |
| A75.2 | Typhus fever due to Rickettsia typhi |
| A75.3 | Typhus fever due to Rickettsia tsutsugamushi |
| A75.9 | Typhus fever, unspecified |
| A77 | Spotted fever [tick-borne rickettsioses] |
| A77.0 | Spotted fever due to Rickettsia rickettsii |
| A77.1 | Spotted fever due to Rickettsia conorii |
| A77.2 | Spotted fever due to Rickettsia siberica |
| A77.3 | Spotted fever due to Rickettsia australis |
| A77.4 | Ehrlichiosis |
| A77.40 | Ehrlichiosis, unspecified |
| A77.41 | Ehrlichiosis chafeensis [E. chafeensis] |
| A77.49 | Other ehrlichiosis |
| A77.8 | Other spotted fevers |
| A77.9 | Spotted fever, unspecified |
| A78 | Q fever |
| A79 | Other rickettsioses |
| A79.0 | Trench fever |
| A79.1 | Rickettsialpox due to Rickettsia akari |
| A79.8 | Other specified rickettsioses |
| A79.81 | Rickettsiosis due to Ehrlichia sennetsu |
| A79.89 | Other specified rickettsioses |
| A79.9 | Rickettsiosis, unspecified |
| A80 | Acute poliomyelitis |
| A80.0 | Acute paralytic poliomyelitis, vaccine-associated |
| A80.1 | Acute paralytic poliomyelitis, wild virus, imported |
| A80.2 | Acute paralytic poliomyelitis, wild virus, indigenous |
| A80.3 | Acute paralytic poliomyelitis, other and unspecified |
| A80.30 | Acute paralytic poliomyelitis, unspecified |
| A80.39 | Other acute paralytic poliomyelitis |
| A80.4 | Acute nonparalytic poliomyelitis |
| A80.9 | Acute poliomyelitis, unspecified |
| A81 | Atypical virus infections of central nervous system |
| A81.0 | Creutzfeldt-Jakob disease |
| A81.00 | Creutzfeldt-Jakob disease, unspecified |
| A81.01 | Variant Creutzfeldt-Jakob disease |
| A81.09 | Other Creutzfeldt-Jakob disease |
| A81.1 | Subacute sclerosing panencephalitis |
| A81.2 | Progressive multifocal leukoencephalopathy |
| A81.8 | Other atypical virus infections of central nervous system |
| A81.81 | Kuru |
| A81.82 | Gerstmann-Straussler-Scheinker syndrome |
| A81.83 | Fatal familial insomnia |
| A81.89 | Other atypical virus infections of central nervous system |
| A81.9 | Atypical virus infection of central nervous system, unspecified |
| A82 | Rabies |
| A82.0 | Sylvatic rabies |
| A82.1 | Urban rabies |
| A82.9 | Rabies, unspecified |
| A83 | Mosquito-borne viral encephalitis |
| A83.0 | Japanese encephalitis |
| A83.1 | Western equine encephalitis |
| A83.2 | Eastern equine encephalitis |
| A83.3 | St Louis encephalitis |
| A83.4 | Australian encephalitis |
| A83.5 | California encephalitis |
| A83.6 | Rocio virus disease |
| A83.8 | Other mosquito-borne viral encephalitis |
| A83.9 | Mosquito-borne viral encephalitis, unspecified |
| A84 | Tick-borne viral encephalitis |
| A84.0 | Far Eastern tick-borne encephalitis [Russian spring-summer encephalitis] |
| A84.1 | Central European tick-borne encephalitis |
| A84.8 | Other tick-borne viral encephalitis |
| A84.9 | Tick-borne viral encephalitis, unspecified |
| A85 | Other viral encephalitis, not elsewhere classified |
| A85.0 | Enteroviral encephalitis |
| A85.1 | Adenoviral encephalitis |
| A85.2 | Arthropod-borne viral encephalitis, unspecified |
| A85.8 | Other specified viral encephalitis |
| A86 | Unspecified viral encephalitis |
| A87 | Viral meningitis |
| A87.0 | Enteroviral meningitis |
| A87.1 | Adenoviral meningitis |
| A87.2 | Lymphocytic choriomeningitis |
| A87.8 | Other viral meningitis |
| A87.9 | Viral meningitis, unspecified |
| A88 | Other viral infections of central nervous system, not elsewhere classified |
| A88.0 | Enteroviral exanthematous fever [Boston exanthem] |
| A88.1 | Epidemic vertigo |
| A88.8 | Other specified viral infections of central nervous system |
| A89 | Unspecified viral infection of central nervous system |
| A90 | Dengue fever [classical dengue] |
| A91 | Dengue hemorrhagic fever |
| A92 | Other mosquito-borne viral fevers |
| A92.0 | Chikungunya virus disease |
| A92.1 | O'nyong-nyong fever |
| A92.2 | Venezuelan equine fever |
| A92.3 | West Nile virus infection |
| A92.30 | West Nile virus infection, unspecified |
| A92.31 | West Nile virus infection with encephalitis |
| A92.32 | West Nile virus infection with other neurologic manifestation |
| A92.39 | West Nile virus infection with other complications |
| A92.4 | Rift Valley fever |
| A92.5 | Zika virus disease |
| A92.8 | Other specified mosquito-borne viral fevers |
| A92.9 | Mosquito-borne viral fever, unspecified |
| A93 | Other arthropod-borne viral fevers, not elsewhere classified |
| A93.0 | Oropouche virus disease |
| A93.1 | Sandfly fever |
| A93.2 | Colorado tick fever |
| A93.8 | Other specified arthropod-borne viral fevers |
| A94 | Unspecified arthropod-borne viral fever |
| A95 | Yellow fever |
| A95.0 | Sylvatic yellow fever |
| A95.1 | Urban yellow fever |
| A95.9 | Yellow fever, unspecified |
| A96 | Arenaviral hemorrhagic fever |
| A96.0 | Junin hemorrhagic fever |
| A96.1 | Machupo hemorrhagic fever |
| A96.2 | Lassa fever |
| A96.8 | Other arenaviral hemorrhagic fevers |
| A96.9 | Arenaviral hemorrhagic fever, unspecified |
| A98 | Other viral hemorrhagic fevers, not elsewhere classified |
| A98.0 | Crimean-Congo hemorrhagic fever |
| A98.1 | Omsk hemorrhagic fever |
| A98.2 | Kyasanur Forest disease |
| A98.3 | Marburg virus disease |
| A98.4 | Ebola virus disease |
| A98.5 | Hemorrhagic fever with renal syndrome |
| A98.8 | Other specified viral hemorrhagic fevers |
| A99 | Unspecified viral hemorrhagic fever |
| B00 | Herpesviral [herpes simplex] infections |
| B00.0 | Eczema herpeticum |
| B00.1 | Herpesviral vesicular dermatitis |
| B00.2 | Herpesviral gingivostomatitis and pharyngotonsillitis |
| B00.3 | Herpesviral meningitis |
| B00.4 | Herpesviral encephalitis |
| B00.5 | Herpesviral ocular disease |
| B00.50 | Herpesviral ocular disease, unspecified |
| B00.51 | Herpesviral iridocyclitis |
| B00.52 | Herpesviral keratitis |
| B00.53 | Herpesviral conjunctivitis |
| B00.59 | Other herpesviral disease of eye |
| B00.7 | Disseminated herpesviral disease |
| B00.8 | Other forms of herpesviral infections |
| B00.81 | Herpesviral hepatitis |
| B00.82 | Herpes simplex myelitis |
| B00.89 | Other herpesviral infection |
| B00.9 | Herpesviral infection, unspecified |
| B01 | Varicella [chickenpox] |
| B01.0 | Varicella meningitis |
| B01.1 | Varicella encephalitis, myelitis and encephalomyelitis |
| B01.11 | Varicella encephalitis and encephalomyelitis |
| B01.12 | Varicella myelitis |
| B01.2 | Varicella pneumonia |
| B01.8 | Varicella with other complications |
| B01.81 | Varicella keratitis |
| B01.89 | Other varicella complications |
| B01.9 | Varicella without complication |
| B02 | Zoster [herpes zoster] |
| B02.0 | Zoster encephalitis |
| B02.1 | Zoster meningitis |
| B02.2 | Zoster with other nervous system involvement |
| B02.21 | Postherpetic geniculate ganglionitis |
| B02.22 | Postherpetic trigeminal neuralgia |
| B02.23 | Postherpetic polyneuropathy |
| B02.24 | Postherpetic myelitis |
| B02.29 | Other postherpetic nervous system involvement |
| B02.3 | Zoster ocular disease |
| B02.30 | Zoster ocular disease, unspecified |
| B02.31 | Zoster conjunctivitis |
| B02.32 | Zoster iridocyclitis |
| B02.33 | Zoster keratitis |
| B02.34 | Zoster scleritis |
| B02.39 | Other herpes zoster eye disease |
| B02.7 | Disseminated zoster |
| B02.8 | Zoster with other complications |
| B02.9 | Zoster without complications |
| B03 | Smallpox |
| B04 | Monkeypox |
| B05 | Measles |
| B05.0 | Measles complicated by encephalitis |
| B05.1 | Measles complicated by meningitis |
| B05.2 | Measles complicated by pneumonia |
| B05.3 | Measles complicated by otitis media |
| B05.4 | Measles with intestinal complications |
| B05.8 | Measles with other complications |
| B05.81 | Measles keratitis and keratoconjunctivitis |
| B05.89 | Other measles complications |
| B05.9 | Measles without complication |
| B06 | Rubella [German measles] |
| B06.0 | Rubella with neurological complications |
| B06.00 | Rubella with neurological complication, unspecified |
| B06.01 | Rubella encephalitis |
| B06.02 | Rubella meningitis |
| B06.09 | Other neurological complications of rubella |
| B06.8 | Rubella with other complications |
| B06.81 | Rubella pneumonia |
| B06.82 | Rubella arthritis |
| B06.89 | Other rubella complications |
| B06.9 | Rubella without complication |
| B07 | Viral warts |
| B07.0 | Plantar wart |
| B07.8 | Other viral warts |
| B07.9 | Viral wart, unspecified |
| B08 | Other viral infections characterized by skin and mucous membrane lesions, not elsewhere classified |
| B08.0 | Other orthopoxvirus infections |
| B08.01 | Cowpox and vaccinia not from vaccine |
| B08.010 | Cowpox |
| B08.011 | Vaccinia not from vaccine |
| B08.02 | Orf virus disease |
| B08.03 | Pseudocowpox [milker's node] |
| B08.04 | Paravaccinia, unspecified |
| B08.09 | Other orthopoxvirus infections |
| B08.1 | Molluscum contagiosum |
| B08.2 | Exanthema subitum [sixth disease] |
| B08.20 | Exanthema subitum [sixth disease], unspecified |
| B08.21 | Exanthema subitum [sixth disease] due to human herpesvirus 6 |
| B08.22 | Exanthema subitum [sixth disease] due to human herpesvirus 7 |
| B08.3 | Erythema infectiosum [fifth disease] |
| B08.4 | Enteroviral vesicular stomatitis with exanthem |
| B08.5 | Enteroviral vesicular pharyngitis |
| B08.6 | Parapoxvirus infections |
| B08.60 | Parapoxvirus infection, unspecified |
| B08.61 | Bovine stomatitis |
| B08.62 | Sealpox |
| B08.69 | Other parapoxvirus infections |
| B08.7 | Yatapoxvirus infections |
| B08.70 | Yatapoxvirus infection, unspecified |
| B08.71 | Tanapox virus disease |
| B08.72 | Yaba pox virus disease |
| B08.79 | Other yatapoxvirus infections |
| B08.8 | Other specified viral infections characterized by skin and mucous membrane lesions |
| B09 | Unspecified viral infection characterized by skin and mucous membrane lesions |
| B10 | Other human herpesviruses |
| B10.0 | Other human herpesvirus encephalitis |
| B10.01 | Human herpesvirus 6 encephalitis |
| B10.09 | Other human herpesvirus encephalitis |
| B10.8 | Other human herpesvirus infection |
| B10.81 | Human herpesvirus 6 infection |
| B10.82 | Human herpesvirus 7 infection |
| B10.89 | Other human herpesvirus infection |
| B15 | Acute hepatitis A |
| B15.0 | Hepatitis A with hepatic coma |
| B15.9 | Hepatitis A without hepatic coma |
| B16 | Acute hepatitis B |
| B16.0 | Acute hepatitis B with delta-agent with hepatic coma |
| B16.1 | Acute hepatitis B with delta-agent without hepatic coma |
| B16.2 | Acute hepatitis B without delta-agent with hepatic coma |
| B16.9 | Acute hepatitis B without delta-agent and without hepatic coma |
| B17 | Other acute viral hepatitis |
| B17.0 | Acute delta-(super) infection of hepatitis B carrier |
| B17.1 | Acute hepatitis C |
| B17.10 | Acute hepatitis C without hepatic coma |
| B17.11 | Acute hepatitis C with hepatic coma |
| B17.2 | Acute hepatitis E |
| B17.8 | Other specified acute viral hepatitis |
| B17.9 | Acute viral hepatitis, unspecified |
| B18 | Chronic viral hepatitis |
| B18.0 | Chronic viral hepatitis B with delta-agent |
| B18.1 | Chronic viral hepatitis B without delta-agent |
| B18.2 | Chronic viral hepatitis C |
| B18.8 | Other chronic viral hepatitis |
| B18.9 | Chronic viral hepatitis, unspecified |
| B19 | Unspecified viral hepatitis |
| B19.0 | Unspecified viral hepatitis with hepatic coma |
| B19.1 | Unspecified viral hepatitis B |
| B19.10 | Unspecified viral hepatitis B without hepatic coma |
| B19.11 | Unspecified viral hepatitis B with hepatic coma |
| B19.2 | Unspecified viral hepatitis C |
| B19.20 | Unspecified viral hepatitis C without hepatic coma |
| B19.21 | Unspecified viral hepatitis C with hepatic coma |
| B19.9 | Unspecified viral hepatitis without hepatic coma |
| B20 | Human immunodeficiency virus [HIV] disease |
| B25 | Cytomegaloviral disease |
| B25.0 | Cytomegaloviral pneumonitis |
| B25.1 | Cytomegaloviral hepatitis |
| B25.2 | Cytomegaloviral pancreatitis |
| B25.8 | Other cytomegaloviral diseases |
| B25.9 | Cytomegaloviral disease, unspecified |
| B26 | Mumps |
| B26.0 | Mumps orchitis |
| B26.1 | Mumps meningitis |
| B26.2 | Mumps encephalitis |
| B26.3 | Mumps pancreatitis |
| B26.8 | Mumps with other complications |
| B26.81 | Mumps hepatitis |
| B26.82 | Mumps myocarditis |
| B26.83 | Mumps nephritis |
| B26.84 | Mumps polyneuropathy |
| B26.85 | Mumps arthritis |
| B26.89 | Other mumps complications |
| B26.9 | Mumps without complication |
| B27 | Infectious mononucleosis |
| B27.0 | Gammaherpesviral mononucleosis |
| B27.00 | Gammaherpesviral mononucleosis without complication |
| B27.01 | Gammaherpesviral mononucleosis with polyneuropathy |
| B27.02 | Gammaherpesviral mononucleosis with meningitis |
| B27.09 | Gammaherpesviral mononucleosis with other complications |
| B27.1 | Cytomegaloviral mononucleosis |
| B27.10 | Cytomegaloviral mononucleosis without complications |
| B27.11 | Cytomegaloviral mononucleosis with polyneuropathy |
| B27.12 | Cytomegaloviral mononucleosis with meningitis |
| B27.19 | Cytomegaloviral mononucleosis with other complication |
| B27.8 | Other infectious mononucleosis |
| B27.80 | Other infectious mononucleosis without complication |
| B27.81 | Other infectious mononucleosis with polyneuropathy |
| B27.82 | Other infectious mononucleosis with meningitis |
| B27.89 | Other infectious mononucleosis with other complication |
| B27.9 | Infectious mononucleosis, unspecified |
| B27.90 | Infectious mononucleosis, unspecified without complication |
| B27.91 | Infectious mononucleosis, unspecified with polyneuropathy |
| B27.92 | Infectious mononucleosis, unspecified with meningitis |
| B27.99 | Infectious mononucleosis, unspecified with other complication |
| B30 | Viral conjunctivitis |
| B30.0 | Keratoconjunctivitis due to adenovirus |
| B30.1 | Conjunctivitis due to adenovirus |
| B30.2 | Viral pharyngoconjunctivitis |
| B30.3 | Acute epidemic hemorrhagic conjunctivitis (enteroviral) |
| B30.8 | Other viral conjunctivitis |
| B30.9 | Viral conjunctivitis, unspecified |
| B33 | Other viral diseases, not elsewhere classified |
| B33.0 | Epidemic myalgia |
| B33.1 | Ross River disease |
| B33.2 | Viral carditis |
| B33.20 | Viral carditis, unspecified |
| B33.21 | Viral endocarditis |
| B33.22 | Viral myocarditis |
| B33.23 | Viral pericarditis |
| B33.24 | Viral cardiomyopathy |
| B33.3 | Retrovirus infections, not elsewhere classified |
| B33.4 | Hantavirus (cardio)-pulmonary syndrome [HPS] [HCPS] |
| B33.8 | Other specified viral diseases |
| B34 | Viral infection of unspecified site |
| B34.0 | Adenovirus infection, unspecified |
| B34.1 | Enterovirus infection, unspecified |
| B34.2 | Coronavirus infection, unspecified |
| B34.3 | Parvovirus infection, unspecified |
| B34.4 | Papovavirus infection, unspecified |
| B34.8 | Other viral infections of unspecified site |
| B34.9 | Viral infection, unspecified |
| B35 | Dermatophytosis |
| B35.0 | Tinea barbae and tinea capitis |
| B35.1 | Tinea unguium |
| B35.2 | Tinea manuum |
| B35.3 | Tinea pedis |
| B35.4 | Tinea corporis |
| B35.5 | Tinea imbricata |
| B35.6 | Tinea cruris |
| B35.8 | Other dermatophytoses |
| B35.9 | Dermatophytosis, unspecified |
| B36 | Other superficial mycoses |
| B36.0 | Pityriasis versicolor |
| B36.1 | Tinea nigra |
| B36.2 | White piedra |
| B36.3 | Black piedra |
| B36.8 | Other specified superficial mycoses |
| B36.9 | Superficial mycosis, unspecified |
| B37 | Candidiasis |
| B37.0 | Candidal stomatitis |
| B37.1 | Pulmonary candidiasis |
| B37.2 | Candidiasis of skin and nail |
| B37.3 | Candidiasis of vulva and vagina |
| B37.4 | Candidiasis of other urogenital sites |
| B37.41 | Candidal cystitis and urethritis |
| B37.42 | Candidal balanitis |
| B37.49 | Other urogenital candidiasis |
| B37.5 | Candidal meningitis |
| B37.6 | Candidal endocarditis |
| B37.7 | Candidal sepsis |
| B37.8 | Candidiasis of other sites |
| B37.81 | Candidal esophagitis |
| B37.82 | Candidal enteritis |
| B37.83 | Candidal cheilitis |
| B37.84 | Candidal otitis externa |
| B37.89 | Other sites of candidiasis |
| B37.9 | Candidiasis, unspecified |
| B38 | Coccidioidomycosis |
| B38.0 | Acute pulmonary coccidioidomycosis |
| B38.1 | Chronic pulmonary coccidioidomycosis |
| B38.2 | Pulmonary coccidioidomycosis, unspecified |
| B38.3 | Cutaneous coccidioidomycosis |
| B38.4 | Coccidioidomycosis meningitis |
| B38.7 | Disseminated coccidioidomycosis |
| B38.8 | Other forms of coccidioidomycosis |
| B38.81 | Prostatic coccidioidomycosis |
| B38.89 | Other forms of coccidioidomycosis |
| B38.9 | Coccidioidomycosis, unspecified |
| B39 | Histoplasmosis |
| B39.0 | Acute pulmonary histoplasmosis capsulati |
| B39.1 | Chronic pulmonary histoplasmosis capsulati |
| B39.2 | Pulmonary histoplasmosis capsulati, unspecified |
| B39.3 | Disseminated histoplasmosis capsulati |
| B39.4 | Histoplasmosis capsulati, unspecified |
| B39.5 | Histoplasmosis duboisii |
| B39.9 | Histoplasmosis, unspecified |
| B40 | Blastomycosis |
| B40.0 | Acute pulmonary blastomycosis |
| B40.1 | Chronic pulmonary blastomycosis |
| B40.2 | Pulmonary blastomycosis, unspecified |
| B40.3 | Cutaneous blastomycosis |
| B40.7 | Disseminated blastomycosis |
| B40.8 | Other forms of blastomycosis |
| B40.81 | Blastomycotic meningoencephalitis |
| B40.89 | Other forms of blastomycosis |
| B40.9 | Blastomycosis, unspecified |
| B41 | Paracoccidioidomycosis |
| B41.0 | Pulmonary paracoccidioidomycosis |
| B41.7 | Disseminated paracoccidioidomycosis |
| B41.8 | Other forms of paracoccidioidomycosis |
| B41.9 | Paracoccidioidomycosis, unspecified |
| B42 | Sporotrichosis |
| B42.0 | Pulmonary sporotrichosis |
| B42.1 | Lymphocutaneous sporotrichosis |
| B42.7 | Disseminated sporotrichosis |
| B42.8 | Other forms of sporotrichosis |
| B42.81 | Cerebral sporotrichosis |
| B42.82 | Sporotrichosis arthritis |
| B42.89 | Other forms of sporotrichosis |
| B42.9 | Sporotrichosis, unspecified |
| B43 | Chromomycosis and pheomycotic abscess |
| B43.0 | Cutaneous chromomycosis |
| B43.1 | Pheomycotic brain abscess |
| B43.2 | Subcutaneous pheomycotic abscess and cyst |
| B43.8 | Other forms of chromomycosis |
| B43.9 | Chromomycosis, unspecified |
| B44 | Aspergillosis |
| B44.0 | Invasive pulmonary aspergillosis |
| B44.1 | Other pulmonary aspergillosis |
| B44.2 | Tonsillar aspergillosis |
| B44.7 | Disseminated aspergillosis |
| B44.8 | Other forms of aspergillosis |
| B44.81 | Allergic bronchopulmonary aspergillosis |
| B44.89 | Other forms of aspergillosis |
| B44.9 | Aspergillosis, unspecified |
| B45 | Cryptococcosis |
| B45.0 | Pulmonary cryptococcosis |
| B45.1 | Cerebral cryptococcosis |
| B45.2 | Cutaneous cryptococcosis |
| B45.3 | Osseous cryptococcosis |
| B45.7 | Disseminated cryptococcosis |
| B45.8 | Other forms of cryptococcosis |
| B45.9 | Cryptococcosis, unspecified |
| B46 | Zygomycosis |
| B46.0 | Pulmonary mucormycosis |
| B46.1 | Rhinocerebral mucormycosis |
| B46.2 | Gastrointestinal mucormycosis |
| B46.3 | Cutaneous mucormycosis |
| B46.4 | Disseminated mucormycosis |
| B46.5 | Mucormycosis, unspecified |
| B46.8 | Other zygomycoses |
| B46.9 | Zygomycosis, unspecified |
| B47 | Mycetoma |
| B47.0 | Eumycetoma |
| B47.1 | Actinomycetoma |
| B47.9 | Mycetoma, unspecified |
| B48 | Other mycoses, not elsewhere classified |
| B48.0 | Lobomycosis |
| B48.1 | Rhinosporidiosis |
| B48.2 | Allescheriasis |
| B48.3 | Geotrichosis |
| B48.4 | Penicillosis |
| B48.8 | Other specified mycoses |
| B49 | Unspecified mycosis |
| B50 | Plasmodium falciparum malaria |
| B50.0 | Plasmodium falciparum malaria with cerebral complications |
| B50.8 | Other severe and complicated Plasmodium falciparum malaria |
| B50.9 | Plasmodium falciparum malaria, unspecified |
| B51 | Plasmodium vivax malaria |
| B51.0 | Plasmodium vivax malaria with rupture of spleen |
| B51.8 | Plasmodium vivax malaria with other complications |
| B51.9 | Plasmodium vivax malaria without complication |
| B52 | Plasmodium malariae malaria |
| B52.0 | Plasmodium malariae malaria with nephropathy |
| B52.8 | Plasmodium malariae malaria with other complications |
| B52.9 | Plasmodium malariae malaria without complication |
| B53 | Other specified malaria |
| B53.0 | Plasmodium ovale malaria |
| B53.1 | Malaria due to simian plasmodia |
| B53.8 | Other malaria, not elsewhere classified |
| B54 | Unspecified malaria |
| B55 | Leishmaniasis |
| B55.0 | Visceral leishmaniasis |
| B55.1 | Cutaneous leishmaniasis |
| B55.2 | Mucocutaneous leishmaniasis |
| B55.9 | Leishmaniasis, unspecified |
| B56 | African trypanosomiasis |
| B56.0 | Gambiense trypanosomiasis |
| B56.1 | Rhodesiense trypanosomiasis |
| B56.9 | African trypanosomiasis, unspecified |
| B57 | Chagas' disease |
| B57.0 | Acute Chagas' disease with heart involvement |
| B57.1 | Acute Chagas' disease without heart involvement |
| B57.2 | Chagas' disease (chronic) with heart involvement |
| B57.3 | Chagas' disease (chronic) with digestive system involvement |
| B57.30 | Chagas' disease with digestive system involvement, unspecified |
| B57.31 | Megaesophagus in Chagas' disease |
| B57.32 | Megacolon in Chagas' disease |
| B57.39 | Other digestive system involvement in Chagas' disease |
| B57.4 | Chagas' disease (chronic) with nervous system involvement |
| B57.40 | Chagas' disease with nervous system involvement, unspecified |
| B57.41 | Meningitis in Chagas' disease |
| B57.42 | Meningoencephalitis in Chagas' disease |
| B57.49 | Other nervous system involvement in Chagas' disease |
| B57.5 | Chagas' disease (chronic) with other organ involvement |
| B58 | Toxoplasmosis |
| B58.0 | Toxoplasma oculopathy |
| B58.00 | Toxoplasma oculopathy, unspecified |
| B58.01 | Toxoplasma chorioretinitis |
| B58.09 | Other toxoplasma oculopathy |
| B58.1 | Toxoplasma hepatitis |
| B58.2 | Toxoplasma meningoencephalitis |
| B58.3 | Pulmonary toxoplasmosis |
| B58.8 | Toxoplasmosis with other organ involvement |
| B58.81 | Toxoplasma myocarditis |
| B58.82 | Toxoplasma myositis |
| B58.83 | Toxoplasma tubulo-interstitial nephropathy |
| B58.89 | Toxoplasmosis with other organ involvement |
| B58.9 | Toxoplasmosis, unspecified |
| B59 | Pneumocystosis |
| B60 | Other protozoal diseases, not elsewhere classified |
| B60.0 | Babesiosis |
| B60.1 | Acanthamebiasis |
| B60.10 | Acanthamebiasis, unspecified |
| B60.11 | Meningoencephalitis due to Acanthamoeba (culbertsoni) |
| B60.12 | Conjunctivitis due to Acanthamoeba |
| B60.13 | Keratoconjunctivitis due to Acanthamoeba |
| B60.19 | Other acanthamebic disease |
| B60.2 | Naegleriasis |
| B60.8 | Other specified protozoal diseases |
| B64 | Unspecified protozoal disease |
| B65 | Schistosomiasis [bilharziasis] |
| B65.0 | Schistosomiasis due to Schistosoma haematobium [urinary schistosomiasis] |
| B65.1 | Schistosomiasis due to Schistosoma mansoni [intestinal schistosomiasis] |
| B65.2 | Schistosomiasis due to Schistosoma japonicum |
| B65.3 | Cercarial dermatitis |
| B65.8 | Other schistosomiasis |
| B65.9 | Schistosomiasis, unspecified |
| B66 | Other fluke infections |
| B66.0 | Opisthorchiasis |
| B66.1 | Clonorchiasis |
| B66.2 | Dicroceliasis |
| B66.3 | Fascioliasis |
| B66.4 | Paragonimiasis |
| B66.5 | Fasciolopsiasis |
| B66.8 | Other specified fluke infections |
| B66.9 | Fluke infection, unspecified |
| B67 | Echinococcosis |
| B67.0 | Echinococcus granulosus infection of liver |
| B67.1 | Echinococcus granulosus infection of lung |
| B67.2 | Echinococcus granulosus infection of bone |
| B67.3 | Echinococcus granulosus infection, other and multiple sites |
| B67.31 | Echinococcus granulosus infection, thyroid gland |
| B67.32 | Echinococcus granulosus infection, multiple sites |
| B67.39 | Echinococcus granulosus infection, other sites |
| B67.4 | Echinococcus granulosus infection, unspecified |
| B67.5 | Echinococcus multilocularis infection of liver |
| B67.6 | Echinococcus multilocularis infection, other and multiple sites |
| B67.61 | Echinococcus multilocularis infection, multiple sites |
| B67.69 | Echinococcus multilocularis infection, other sites |
| B67.7 | Echinococcus multilocularis infection, unspecified |
| B67.8 | Echinococcosis, unspecified, of liver |
| B67.9 | Echinococcosis, other and unspecified |
| B67.90 | Echinococcosis, unspecified |
| B67.99 | Other echinococcosis |
| B68 | Taeniasis |
| B68.0 | Taenia solium taeniasis |
| B68.1 | Taenia saginata taeniasis |
| B68.9 | Taeniasis, unspecified |
| B69 | Cysticercosis |
| B69.0 | Cysticercosis of central nervous system |
| B69.1 | Cysticercosis of eye |
| B69.8 | Cysticercosis of other sites |
| B69.81 | Myositis in cysticercosis |
| B69.89 | Cysticercosis of other sites |
| B69.9 | Cysticercosis, unspecified |
| B70 | Diphyllobothriasis and sparganosis |
| B70.0 | Diphyllobothriasis |
| B70.1 | Sparganosis |
| B71 | Other cestode infections |
| B71.0 | Hymenolepiasis |
| B71.1 | Dipylidiasis |
| B71.8 | Other specified cestode infections |
| B71.9 | Cestode infection, unspecified |
| B72 | Dracunculiasis |
| B73 | Onchocerciasis |
| B73.0 | Onchocerciasis with eye disease |
| B73.00 | Onchocerciasis with eye involvement, unspecified |
| B73.01 | Onchocerciasis with endophthalmitis |
| B73.02 | Onchocerciasis with glaucoma |
| B73.09 | Onchocerciasis with other eye involvement |
| B73.1 | Onchocerciasis without eye disease |
| B74 | Filariasis |
| B74.0 | Filariasis due to Wuchereria bancrofti |
| B74.1 | Filariasis due to Brugia malayi |
| B74.2 | Filariasis due to Brugia timori |
| B74.3 | Loiasis |
| B74.4 | Mansonelliasis |
| B74.8 | Other filariases |
| B74.9 | Filariasis, unspecified |
| B75 | Trichinellosis |
| B76 | Hookworm diseases |
| B76.0 | Ancylostomiasis |
| B76.1 | Necatoriasis |
| B76.8 | Other hookworm diseases |
| B76.9 | Hookworm disease, unspecified |
| B77 | Ascariasis |
| B77.0 | Ascariasis with intestinal complications |
| B77.8 | Ascariasis with other complications |
| B77.81 | Ascariasis pneumonia |
| B77.89 | Ascariasis with other complications |
| B77.9 | Ascariasis, unspecified |
| B78 | Strongyloidiasis |
| B78.0 | Intestinal strongyloidiasis |
| B78.1 | Cutaneous strongyloidiasis |
| B78.7 | Disseminated strongyloidiasis |
| B78.9 | Strongyloidiasis, unspecified |
| B79 | Trichuriasis |
| B80 | Enterobiasis |
| B81 | Other intestinal helminthiases, not elsewhere classified |
| B81.0 | Anisakiasis |
| B81.1 | Intestinal capillariasis |
| B81.2 | Trichostrongyliasis |
| B81.3 | Intestinal angiostrongyliasis |
| B81.4 | Mixed intestinal helminthiases |
| B81.8 | Other specified intestinal helminthiases |
| B82 | Unspecified intestinal parasitism |
| B82.0 | Intestinal helminthiasis, unspecified |
| B82.9 | Intestinal parasitism, unspecified |
| B83 | Other helminthiases |
| B83.0 | Visceral larva migrans |
| B83.1 | Gnathostomiasis |
| B83.2 | Angiostrongyliasis due to Parastrongylus cantonensis |
| B83.3 | Syngamiasis |
| B83.4 | Internal hirudiniasis |
| B83.8 | Other specified helminthiases |
| B83.9 | Helminthiasis, unspecified |
| B85 | Pediculosis and phthiriasis |
| B85.0 | Pediculosis due to Pediculus humanus capitis |
| B85.1 | Pediculosis due to Pediculus humanus corporis |
| B85.2 | Pediculosis, unspecified |
| B85.3 | Phthiriasis |
| B85.4 | Mixed pediculosis and phthiriasis |
| B86 | Scabies |
| B87 | Myiasis |
| B87.0 | Cutaneous myiasis |
| B87.1 | Wound myiasis |
| B87.2 | Ocular myiasis |
| B87.3 | Nasopharyngeal myiasis |
| B87.4 | Aural myiasis |
| B87.8 | Myiasis of other sites |
| B87.81 | Genitourinary myiasis |
| B87.82 | Intestinal myiasis |
| B87.89 | Myiasis of other sites |
| B87.9 | Myiasis, unspecified |
| B88 | Other infestations |
| B88.0 | Other acariasis |
| B88.1 | Tungiasis [sandflea infestation] |
| B88.2 | Other arthropod infestations |
| B88.3 | External hirudiniasis |
| B88.8 | Other specified infestations |
| B88.9 | Infestation, unspecified |
| B89 | Unspecified parasitic disease |
| B90 | Sequelae of tuberculosis |
| B90.0 | Sequelae of central nervous system tuberculosis |
| B90.1 | Sequelae of genitourinary tuberculosis |
| B90.2 | Sequelae of tuberculosis of bones and joints |
| B90.8 | Sequelae of tuberculosis of other organs |
| B90.9 | Sequelae of respiratory and unspecified tuberculosis |
| B91 | Sequelae of poliomyelitis |
| B92 | Sequelae of leprosy |
| B94 | Sequelae of other and unspecified infectious and parasitic diseases |
| B94.0 | Sequelae of trachoma |
| B94.1 | Sequelae of viral encephalitis |
| B94.2 | Sequelae of viral hepatitis |
| B94.8 | Sequelae of other specified infectious and parasitic diseases |
| B94.9 | Sequelae of unspecified infectious and parasitic disease |
| B95 | Streptococcus, Staphylococcus, and Enterococcus as the cause of diseases classified elsewhere |
| B95.0 | Streptococcus, group A, as the cause of diseases classified elsewhere |
| B95.1 | Streptococcus, group B, as the cause of diseases classified elsewhere |
| B95.2 | Enterococcus as the cause of diseases classified elsewhere |
| B95.3 | Streptococcus pneumoniae as the cause of diseases classified elsewhere |
| B95.4 | Other streptococcus as the cause of diseases classified elsewhere |
| B95.5 | Unspecified streptococcus as the cause of diseases classified elsewhere |
| B95.6 | Staphylococcus aureus as the cause of diseases classified elsewhere |
| B95.61 | Methicillin susceptible Staphylococcus aureus infection as the cause of diseases classified elsewhere |
| B95.62 | Methicillin resistant Staphylococcus aureus infection as the cause of diseases classified elsewhere |
| B95.7 | Other staphylococcus as the cause of diseases classified elsewhere |
| B95.8 | Unspecified staphylococcus as the cause of diseases classified elsewhere |
| B96 | Other bacterial agents as the cause of diseases classified elsewhere |
| B96.0 | Mycoplasma pneumoniae [M. pneumoniae] as the cause of diseases classified elsewhere |
| B96.1 | Klebsiella pneumoniae [K. pneumoniae] as the cause of diseases classified elsewhere |
| B96.2 | Escherichia coli [E. coli ] as the cause of diseases classified elsewhere |
| B96.20 | Unspecified Escherichia coli [E. coli] as the cause of diseases classified elsewhere |
| B96.21 | Shiga toxin-producing Escherichia coli [E. coli] [STEC] O157 as the cause of diseases classified elsewhere |
| B96.22 | Other specified Shiga toxin-producing Escherichia coli [E. coli] [STEC] as the cause of diseases classified elsewhere |
| B96.23 | Unspecified Shiga toxin-producing Escherichia coli [E. coli] [STEC] as the cause of diseases classified elsewhere |
| B96.29 | Other Escherichia coli [E. coli] as the cause of diseases classified elsewhere |
| B96.3 | Hemophilus influenzae [H. influenzae] as the cause of diseases classified elsewhere |
| B96.4 | Proteus (mirabilis) (morganii) as the cause of diseases classified elsewhere |
| B96.5 | Pseudomonas (aeruginosa) (mallei) (pseudomallei) as the cause of diseases classified elsewhere |
| B96.6 | Bacteroides fragilis [B. fragilis] as the cause of diseases classified elsewhere |
| B96.7 | Clostridium perfringens [C. perfringens] as the cause of diseases classified elsewhere |
| B96.8 | Other specified bacterial agents as the cause of diseases classified elsewhere |
| B96.81 | Helicobacter pylori [H. pylori] as the cause of diseases classified elsewhere |
| B96.82 | Vibrio vulnificus as the cause of diseases classified elsewhere |
| B96.89 | Other specified bacterial agents as the cause of diseases classified elsewhere |
| B97 | Viral agents as the cause of diseases classified elsewhere |
| B97.0 | Adenovirus as the cause of diseases classified elsewhere |
| B97.1 | Enterovirus as the cause of diseases classified elsewhere |
| B97.10 | Unspecified enterovirus as the cause of diseases classified elsewhere |
| B97.11 | Coxsackievirus as the cause of diseases classified elsewhere |
| B97.12 | Echovirus as the cause of diseases classified elsewhere |
| B97.19 | Other enterovirus as the cause of diseases classified elsewhere |
| B97.2 | Coronavirus as the cause of diseases classified elsewhere |
| B97.21 | SARS-associated coronavirus as the cause of diseases classified elsewhere |
| B97.29 | Other coronavirus as the cause of diseases classified elsewhere |
| B97.3 | Retrovirus as the cause of diseases classified elsewhere |
| B97.30 | Unspecified retrovirus as the cause of diseases classified elsewhere |
| B97.31 | Lentivirus as the cause of diseases classified elsewhere |
| B97.32 | Oncovirus as the cause of diseases classified elsewhere |
| B97.33 | Human T-cell lymphotrophic virus, type I [HTLV-I] as the cause of diseases classified elsewhere |
| B97.34 | Human T-cell lymphotrophic virus, type II [HTLV-II] as the cause of diseases classified elsewhere |
| B97.35 | Human immunodeficiency virus, type 2 [HIV 2] as the cause of diseases classified elsewhere |
| B97.39 | Other retrovirus as the cause of diseases classified elsewhere |
| B97.4 | Respiratory syncytial virus as the cause of diseases classified elsewhere |
| B97.5 | Reovirus as the cause of diseases classified elsewhere |
| B97.6 | Parvovirus as the cause of diseases classified elsewhere |
| B97.7 | Papillomavirus as the cause of diseases classified elsewhere |
| B97.8 | Other viral agents as the cause of diseases classified elsewhere |
| B97.81 | Human metapneumovirus as the cause of diseases classified elsewhere |
| B97.89 | Other viral agents as the cause of diseases classified elsewhere |
| B99 | Other and unspecified infectious diseases |
| B99.8 | Other infectious disease |
| B99.9 | Unspecified infectious disease |
| C40 | Malignant neoplasm of bone and articular cartilage of limbs |
| C40.0 | Malignant neoplasm of scapula and long bones of upper limb |
| C40.00 | Malignant neoplasm of scapula and long bones of unspecified upper limb |
| C40.01 | Malignant neoplasm of scapula and long bones of right upper limb |
| C40.02 | Malignant neoplasm of scapula and long bones of left upper limb |
| C40.1 | Malignant neoplasm of short bones of upper limb |
| C40.10 | Malignant neoplasm of short bones of unspecified upper limb |
| C40.11 | Malignant neoplasm of short bones of right upper limb |
| C40.12 | Malignant neoplasm of short bones of left upper limb |
| C40.2 | Malignant neoplasm of long bones of lower limb |
| C40.20 | Malignant neoplasm of long bones of unspecified lower limb |
| C40.21 | Malignant neoplasm of long bones of right lower limb |
| C40.22 | Malignant neoplasm of long bones of left lower limb |
| C40.3 | Malignant neoplasm of short bones of lower limb |
| C40.30 | Malignant neoplasm of short bones of unspecified lower limb |
| C40.31 | Malignant neoplasm of short bones of right lower limb |
| C40.32 | Malignant neoplasm of short bones of left lower limb |
| C40.8 | Malignant neoplasm of overlapping sites of bone and articular cartilage of limb |
| C40.80 | Malignant neoplasm of overlapping sites of bone and articular cartilage of unspecified limb |
| C40.81 | Malignant neoplasm of overlapping sites of bone and articular cartilage of right limb |
| C40.82 | Malignant neoplasm of overlapping sites of bone and articular cartilage of left limb |
| C40.9 | Malignant neoplasm of unspecified bones and articular cartilage of limb |
| C40.90 | Malignant neoplasm of unspecified bones and articular cartilage of unspecified limb |
| C40.91 | Malignant neoplasm of unspecified bones and articular cartilage of right limb |
| C40.92 | Malignant neoplasm of unspecified bones and articular cartilage of left limb |
| C41 | Malignant neoplasm of bone and articular cartilage of other and unspecified sites |
| C41.0 | Malignant neoplasm of bones of skull and face |
| C41.1 | Malignant neoplasm of mandible |
| C41.2 | Malignant neoplasm of vertebral column |
| C41.3 | Malignant neoplasm of ribs, sternum and clavicle |
| C41.4 | Malignant neoplasm of pelvic bones, sacrum and coccyx |
| C41.9 | Malignant neoplasm of bone and articular cartilage, unspecified |
| C49 | Malignant neoplasm of other connective and soft tissue |
| C49.0 | Malignant neoplasm of connective and soft tissue of head, face and neck |
| C49.1 | Malignant neoplasm of connective and soft tissue of upper limb, including shoulder |
| C49.10 | Malignant neoplasm of connective and soft tissue of unspecified upper limb, including shoulder |
| C49.11 | Malignant neoplasm of connective and soft tissue of right upper limb, including shoulder |
| C49.12 | Malignant neoplasm of connective and soft tissue of left upper limb, including shoulder |
| C49.2 | Malignant neoplasm of connective and soft tissue of lower limb, including hip |
| C49.20 | Malignant neoplasm of connective and soft tissue of unspecified lower limb, including hip |
| C49.21 | Malignant neoplasm of connective and soft tissue of right lower limb, including hip |
| C49.22 | Malignant neoplasm of connective and soft tissue of left lower limb, including hip |
| C49.3 | Malignant neoplasm of connective and soft tissue of thorax |
| C49.4 | Malignant neoplasm of connective and soft tissue of abdomen |
| C49.5 | Malignant neoplasm of connective and soft tissue of pelvis |
| C49.6 | Malignant neoplasm of connective and soft tissue of trunk, unspecified |
| C49.8 | Malignant neoplasm of overlapping sites of connective and soft tissue |
| C49.9 | Malignant neoplasm of connective and soft tissue, unspecified |
| C49.A | Gastrointestinal stromal tumor |
| C49.A0 | Gastrointestinal stromal tumor, unspecified site |
| C49.A1 | Gastrointestinal stromal tumor of esophagus |
| C49.A2 | Gastrointestinal stromal tumor of stomach |
| C49.A3 | Gastrointestinal stromal tumor of small intestine |
| C49.A4 | Gastrointestinal stromal tumor of large intestine |
| C49.A5 | Gastrointestinal stromal tumor of rectum |
| C49.A9 | Gastrointestinal stromal tumor of other sites |
| C70 | Malignant neoplasm of meninges |
| C70.0 | Malignant neoplasm of cerebral meninges |
| C70.1 | Malignant neoplasm of spinal meninges |
| C70.9 | Malignant neoplasm of meninges, unspecified |
| C72 | Malignant neoplasm of spinal cord, cranial nerves and other parts of central nervous system |
| C72.0 | Malignant neoplasm of spinal cord |
| C72.1 | Malignant neoplasm of cauda equina |
| C72.2 | Malignant neoplasm of olfactory nerve |
| C72.20 | Malignant neoplasm of unspecified olfactory nerve |
| C72.21 | Malignant neoplasm of right olfactory nerve |
| C72.22 | Malignant neoplasm of left olfactory nerve |
| C72.3 | Malignant neoplasm of optic nerve |
| C72.30 | Malignant neoplasm of unspecified optic nerve |
| C72.31 | Malignant neoplasm of right optic nerve |
| C72.32 | Malignant neoplasm of left optic nerve |
| C72.4 | Malignant neoplasm of acoustic nerve |
| C72.40 | Malignant neoplasm of unspecified acoustic nerve |
| C72.41 | Malignant neoplasm of right acoustic nerve |
| C72.42 | Malignant neoplasm of left acoustic nerve |
| C72.5 | Malignant neoplasm of other and unspecified cranial nerves |
| C72.50 | Malignant neoplasm of unspecified cranial nerve |
| C72.59 | Malignant neoplasm of other cranial nerves |
| C72.9 | Malignant neoplasm of central nervous system, unspecified |
| C76 | Malignant neoplasm of other and ill-defined sites |
| C76.0 | Malignant neoplasm of head, face and neck |
| C76.1 | Malignant neoplasm of thorax |
| C76.2 | Malignant neoplasm of abdomen |
| C76.3 | Malignant neoplasm of pelvis |
| C76.4 | Malignant neoplasm of upper limb |
| C76.40 | Malignant neoplasm of unspecified upper limb |
| C76.41 | Malignant neoplasm of right upper limb |
| C76.42 | Malignant neoplasm of left upper limb |
| C76.5 | Malignant neoplasm of lower limb |
| C76.50 | Malignant neoplasm of unspecified lower limb |
| C76.51 | Malignant neoplasm of right lower limb |
| C76.52 | Malignant neoplasm of left lower limb |
| C76.8 | Malignant neoplasm of other specified ill-defined sites |
| C79 | Secondary malignant neoplasm of other and unspecified sites |
| C79.0 | Secondary malignant neoplasm of kidney and renal pelvis |
| C79.00 | Secondary malignant neoplasm of unspecified kidney and renal pelvis |
| C79.01 | Secondary malignant neoplasm of right kidney and renal pelvis |
| C79.02 | Secondary malignant neoplasm of left kidney and renal pelvis |
| C79.1 | Secondary malignant neoplasm of bladder and other and unspecified urinary organs |
| C79.10 | Secondary malignant neoplasm of unspecified urinary organs |
| C79.11 | Secondary malignant neoplasm of bladder |
| C79.19 | Secondary malignant neoplasm of other urinary organs |
| C79.2 | Secondary malignant neoplasm of skin |
| C79.3 | Secondary malignant neoplasm of brain and cerebral meninges |
| C79.31 | Secondary malignant neoplasm of brain |
| C79.32 | Secondary malignant neoplasm of cerebral meninges |
| C79.4 | Secondary malignant neoplasm of other and unspecified parts of nervous system |
| C79.40 | Secondary malignant neoplasm of unspecified part of nervous system |
| C79.49 | Secondary malignant neoplasm of other parts of nervous system |
| C79.5 | Secondary malignant neoplasm of bone and bone marrow |
| C79.51 | Secondary malignant neoplasm of bone |
| C79.52 | Secondary malignant neoplasm of bone marrow |
| C79.6 | Secondary malignant neoplasm of ovary |
| C79.60 | Secondary malignant neoplasm of unspecified ovary |
| C79.61 | Secondary malignant neoplasm of right ovary |
| C79.62 | Secondary malignant neoplasm of left ovary |
| C79.7 | Secondary malignant neoplasm of adrenal gland |
| C79.70 | Secondary malignant neoplasm of unspecified adrenal gland |
| C79.71 | Secondary malignant neoplasm of right adrenal gland |
| C79.72 | Secondary malignant neoplasm of left adrenal gland |
| C79.8 | Secondary malignant neoplasm of other specified sites |
| C79.81 | Secondary malignant neoplasm of breast |
| C79.82 | Secondary malignant neoplasm of genital organs |
| C79.89 | Secondary malignant neoplasm of other specified sites |
| C79.9 | Secondary malignant neoplasm of unspecified site |
| D00 | Carcinoma in situ of oral cavity, esophagus and stomach |
| D00.0 | Carcinoma in situ of lip, oral cavity and pharynx |
| D00.00 | Carcinoma in situ of oral cavity, unspecified site |
| D00.01 | Carcinoma in situ of labial mucosa and vermilion border |
| D00.02 | Carcinoma in situ of buccal mucosa |
| D00.03 | Carcinoma in situ of gingiva and edentulous alveolar ridge |
| D00.04 | Carcinoma in situ of soft palate |
| D00.05 | Carcinoma in situ of hard palate |
| D00.06 | Carcinoma in situ of floor of mouth |
| D00.07 | Carcinoma in situ of tongue |
| D00.08 | Carcinoma in situ of pharynx |
| D00.1 | Carcinoma in situ of esophagus |
| D00.2 | Carcinoma in situ of stomach |
| D01 | Carcinoma in situ of other and unspecified digestive organs |
| D01.0 | Carcinoma in situ of colon |
| D01.1 | Carcinoma in situ of rectosigmoid junction |
| D01.2 | Carcinoma in situ of rectum |
| D01.3 | Carcinoma in situ of anus and anal canal |
| D01.4 | Carcinoma in situ of other and unspecified parts of intestine |
| D01.40 | Carcinoma in situ of unspecified part of intestine |
| D01.49 | Carcinoma in situ of other parts of intestine |
| D01.5 | Carcinoma in situ of liver, gallbladder and bile ducts |
| D01.7 | Carcinoma in situ of other specified digestive organs |
| D01.9 | Carcinoma in situ of digestive organ, unspecified |
| D02 | Carcinoma in situ of middle ear and respiratory system |
| D02.0 | Carcinoma in situ of larynx |
| D02.1 | Carcinoma in situ of trachea |
| D02.2 | Carcinoma in situ of bronchus and lung |
| D02.20 | Carcinoma in situ of unspecified bronchus and lung |
| D02.21 | Carcinoma in situ of right bronchus and lung |
| D02.22 | Carcinoma in situ of left bronchus and lung |
| D02.3 | Carcinoma in situ of other parts of respiratory system |
| D02.4 | Carcinoma in situ of respiratory system, unspecified |
| D03 | Melanoma in situ |
| D03.0 | Melanoma in situ of lip |
| D03.1 | Melanoma in situ of eyelid, including canthus |
| D03.10 | Melanoma in situ of unspecified eyelid, including canthus |
| D03.11 | Melanoma in situ of right eyelid, including canthus |
| D03.111 | Melanoma in situ of right upper eyelid, including canthus |
| D03.112 | Melanoma in situ of right lower eyelid, including canthus |
| D03.12 | Melanoma in situ of left eyelid, including canthus |
| D03.121 | Melanoma in situ of left upper eyelid, including canthus |
| D03.122 | Melanoma in situ of left lower eyelid, including canthus |
| D03.2 | Melanoma in situ of ear and external auricular canal |
| D03.20 | Melanoma in situ of unspecified ear and external auricular canal |
| D03.21 | Melanoma in situ of right ear and external auricular canal |
| D03.22 | Melanoma in situ of left ear and external auricular canal |
| D03.3 | Melanoma in situ of other and unspecified parts of face |
| D03.30 | Melanoma in situ of unspecified part of face |
| D03.39 | Melanoma in situ of other parts of face |
| D03.4 | Melanoma in situ of scalp and neck |
| D03.5 | Melanoma in situ of trunk |
| D03.51 | Melanoma in situ of anal skin |
| D03.52 | Melanoma in situ of breast (skin) (soft tissue) |
| D03.59 | Melanoma in situ of other part of trunk |
| D03.6 | Melanoma in situ of upper limb, including shoulder |
| D03.60 | Melanoma in situ of unspecified upper limb, including shoulder |
| D03.61 | Melanoma in situ of right upper limb, including shoulder |
| D03.62 | Melanoma in situ of left upper limb, including shoulder |
| D03.7 | Melanoma in situ of lower limb, including hip |
| D03.70 | Melanoma in situ of unspecified lower limb, including hip |
| D03.71 | Melanoma in situ of right lower limb, including hip |
| D03.72 | Melanoma in situ of left lower limb, including hip |
| D03.8 | Melanoma in situ of other sites |
| D03.9 | Melanoma in situ, unspecified |
| D04 | Carcinoma in situ of skin |
| D04.0 | Carcinoma in situ of skin of lip |
| D04.1 | Carcinoma in situ of skin of eyelid, including canthus |
| D04.10 | Carcinoma in situ of skin of unspecified eyelid, including canthus |
| D04.11 | Carcinoma in situ of skin of right eyelid, including canthus |
| D04.111 | Carcinoma in situ of skin of right upper eyelid, including canthus |
| D04.112 | Carcinoma in situ of skin of right lower eyelid, including canthus |
| D04.12 | Carcinoma in situ of skin of left eyelid, including canthus |
| D04.121 | Carcinoma in situ of skin of left upper eyelid, including canthus |
| D04.122 | Carcinoma in situ of skin of left lower eyelid, including canthus |
| D04.2 | Carcinoma in situ of skin of ear and external auricular canal |
| D04.20 | Carcinoma in situ of skin of unspecified ear and external auricular canal |
| D04.21 | Carcinoma in situ of skin of right ear and external auricular canal |
| D04.22 | Carcinoma in situ of skin of left ear and external auricular canal |
| D04.3 | Carcinoma in situ of skin of other and unspecified parts of face |
| D04.30 | Carcinoma in situ of skin of unspecified part of face |
| D04.39 | Carcinoma in situ of skin of other parts of face |
| D04.4 | Carcinoma in situ of skin of scalp and neck |
| D04.5 | Carcinoma in situ of skin of trunk |
| D04.6 | Carcinoma in situ of skin of upper limb, including shoulder |
| D04.60 | Carcinoma in situ of skin of unspecified upper limb, including shoulder |
| D04.61 | Carcinoma in situ of skin of right upper limb, including shoulder |
| D04.62 | Carcinoma in situ of skin of left upper limb, including shoulder |
| D04.7 | Carcinoma in situ of skin of lower limb, including hip |
| D04.70 | Carcinoma in situ of skin of unspecified lower limb, including hip |
| D04.71 | Carcinoma in situ of skin of right lower limb, including hip |
| D04.72 | Carcinoma in situ of skin of left lower limb, including hip |
| D04.8 | Carcinoma in situ of skin of other sites |
| D04.9 | Carcinoma in situ of skin, unspecified |
| D05 | Carcinoma in situ of breast |
| D05.0 | Lobular carcinoma in situ of breast |
| D05.00 | Lobular carcinoma in situ of unspecified breast |
| D05.01 | Lobular carcinoma in situ of right breast |
| D05.02 | Lobular carcinoma in situ of left breast |
| D05.1 | Intraductal carcinoma in situ of breast |
| D05.10 | Intraductal carcinoma in situ of unspecified breast |
| D05.11 | Intraductal carcinoma in situ of right breast |
| D05.12 | Intraductal carcinoma in situ of left breast |
| D05.8 | Other specified type of carcinoma in situ of breast |
| D05.80 | Other specified type of carcinoma in situ of unspecified breast |
| D05.81 | Other specified type of carcinoma in situ of right breast |
| D05.82 | Other specified type of carcinoma in situ of left breast |
| D05.9 | Unspecified type of carcinoma in situ of breast |
| D05.90 | Unspecified type of carcinoma in situ of unspecified breast |
| D05.91 | Unspecified type of carcinoma in situ of right breast |
| D05.92 | Unspecified type of carcinoma in situ of left breast |
| D06 | Carcinoma in situ of cervix uteri |
| D06.0 | Carcinoma in situ of endocervix |
| D06.1 | Carcinoma in situ of exocervix |
| D06.7 | Carcinoma in situ of other parts of cervix |
| D06.9 | Carcinoma in situ of cervix, unspecified |
| D07 | Carcinoma in situ of other and unspecified genital organs |
| D07.0 | Carcinoma in situ of endometrium |
| D07.1 | Carcinoma in situ of vulva |
| D07.2 | Carcinoma in situ of vagina |
| D07.3 | Carcinoma in situ of other and unspecified female genital organs |
| D07.30 | Carcinoma in situ of unspecified female genital organs |
| D07.39 | Carcinoma in situ of other female genital organs |
| D07.4 | Carcinoma in situ of penis |
| D07.5 | Carcinoma in situ of prostate |
| D07.6 | Carcinoma in situ of other and unspecified male genital organs |
| D07.60 | Carcinoma in situ of unspecified male genital organs |
| D07.61 | Carcinoma in situ of scrotum |
| D07.69 | Carcinoma in situ of other male genital organs |
| D09 | Carcinoma in situ of other and unspecified sites |
| D09.0 | Carcinoma in situ of bladder |
| D09.1 | Carcinoma in situ of other and unspecified urinary organs |
| D09.10 | Carcinoma in situ of unspecified urinary organ |
| D09.19 | Carcinoma in situ of other urinary organs |
| D09.2 | Carcinoma in situ of eye |
| D09.20 | Carcinoma in situ of unspecified eye |
| D09.21 | Carcinoma in situ of right eye |
| D09.22 | Carcinoma in situ of left eye |
| D09.3 | Carcinoma in situ of thyroid and other endocrine glands |
| D09.8 | Carcinoma in situ of other specified sites |
| D09.9 | Carcinoma in situ, unspecified |
| D10 | Benign neoplasm of mouth and pharynx |
| D10.0 | Benign neoplasm of lip |
| D10.1 | Benign neoplasm of tongue |
| D10.2 | Benign neoplasm of floor of mouth |
| D10.3 | Benign neoplasm of other and unspecified parts of mouth |
| D10.30 | Benign neoplasm of unspecified part of mouth |
| D10.39 | Benign neoplasm of other parts of mouth |
| D10.4 | Benign neoplasm of tonsil |
| D10.5 | Benign neoplasm of other parts of oropharynx |
| D10.6 | Benign neoplasm of nasopharynx |
| D10.7 | Benign neoplasm of hypopharynx |
| D10.9 | Benign neoplasm of pharynx, unspecified |
| D11 | Benign neoplasm of major salivary glands |
| D11.0 | Benign neoplasm of parotid gland |
| D11.7 | Benign neoplasm of other major salivary glands |
| D11.9 | Benign neoplasm of major salivary gland, unspecified |
| D12 | Benign neoplasm of colon, rectum, anus and anal canal |
| D12.0 | Benign neoplasm of cecum |
| D12.1 | Benign neoplasm of appendix |
| D12.2 | Benign neoplasm of ascending colon |
| D12.3 | Benign neoplasm of transverse colon |
| D12.4 | Benign neoplasm of descending colon |
| D12.5 | Benign neoplasm of sigmoid colon |
| D12.6 | Benign neoplasm of colon, unspecified |
| D12.7 | Benign neoplasm of rectosigmoid junction |
| D12.8 | Benign neoplasm of rectum |
| D12.9 | Benign neoplasm of anus and anal canal |
| D13 | Benign neoplasm of other and ill-defined parts of digestive system |
| D13.0 | Benign neoplasm of esophagus |
| D13.1 | Benign neoplasm of stomach |
| D13.2 | Benign neoplasm of duodenum |
| D13.3 | Benign neoplasm of other and unspecified parts of small intestine |
| D13.30 | Benign neoplasm of unspecified part of small intestine |
| D13.39 | Benign neoplasm of other parts of small intestine |
| D13.4 | Benign neoplasm of liver |
| D13.5 | Benign neoplasm of extrahepatic bile ducts |
| D13.6 | Benign neoplasm of pancreas |
| D13.7 | Benign neoplasm of endocrine pancreas |
| D13.9 | Benign neoplasm of ill-defined sites within the digestive system |
| D14 | Benign neoplasm of middle ear and respiratory system |
| D14.0 | Benign neoplasm of middle ear, nasal cavity and accessory sinuses |
| D14.1 | Benign neoplasm of larynx |
| D14.2 | Benign neoplasm of trachea |
| D14.3 | Benign neoplasm of bronchus and lung |
| D14.30 | Benign neoplasm of unspecified bronchus and lung |
| D14.31 | Benign neoplasm of right bronchus and lung |
| D14.32 | Benign neoplasm of left bronchus and lung |
| D14.4 | Benign neoplasm of respiratory system, unspecified |
| D15 | Benign neoplasm of other and unspecified intrathoracic organs |
| D15.0 | Benign neoplasm of thymus |
| D15.1 | Benign neoplasm of heart |
| D15.2 | Benign neoplasm of mediastinum |
| D15.7 | Benign neoplasm of other specified intrathoracic organs |
| D15.9 | Benign neoplasm of intrathoracic organ, unspecified |
| D16 | Benign neoplasm of bone and articular cartilage |
| D16.0 | Benign neoplasm of scapula and long bones of upper limb |
| D16.00 | Benign neoplasm of scapula and long bones of unspecified upper limb |
| D16.01 | Benign neoplasm of scapula and long bones of right upper limb |
| D16.02 | Benign neoplasm of scapula and long bones of left upper limb |
| D16.1 | Benign neoplasm of short bones of upper limb |
| D16.10 | Benign neoplasm of short bones of unspecified upper limb |
| D16.11 | Benign neoplasm of short bones of right upper limb |
| D16.12 | Benign neoplasm of short bones of left upper limb |
| D16.2 | Benign neoplasm of long bones of lower limb |
| D16.20 | Benign neoplasm of long bones of unspecified lower limb |
| D16.21 | Benign neoplasm of long bones of right lower limb |
| D16.22 | Benign neoplasm of long bones of left lower limb |
| D16.3 | Benign neoplasm of short bones of lower limb |
| D16.30 | Benign neoplasm of short bones of unspecified lower limb |
| D16.31 | Benign neoplasm of short bones of right lower limb |
| D16.32 | Benign neoplasm of short bones of left lower limb |
| D16.4 | Benign neoplasm of bones of skull and face |
| D16.5 | Benign neoplasm of lower jaw bone |
| D16.6 | Benign neoplasm of vertebral column |
| D16.7 | Benign neoplasm of ribs, sternum and clavicle |
| D16.8 | Benign neoplasm of pelvic bones, sacrum and coccyx |
| D16.9 | Benign neoplasm of bone and articular cartilage, unspecified |
| D17 | Benign lipomatous neoplasm |
| D17.0 | Benign lipomatous neoplasm of skin and subcutaneous tissue of head, face and neck |
| D17.1 | Benign lipomatous neoplasm of skin and subcutaneous tissue of trunk |
| D17.2 | Benign lipomatous neoplasm of skin and subcutaneous tissue of limb |
| D17.20 | Benign lipomatous neoplasm of skin and subcutaneous tissue of unspecified limb |
| D17.21 | Benign lipomatous neoplasm of skin and subcutaneous tissue of right arm |
| D17.22 | Benign lipomatous neoplasm of skin and subcutaneous tissue of left arm |
| D17.23 | Benign lipomatous neoplasm of skin and subcutaneous tissue of right leg |
| D17.24 | Benign lipomatous neoplasm of skin and subcutaneous tissue of left leg |
| D17.3 | Benign lipomatous neoplasm of skin and subcutaneous tissue of other and unspecified sites |
| D17.30 | Benign lipomatous neoplasm of skin and subcutaneous tissue of unspecified sites |
| D17.39 | Benign lipomatous neoplasm of skin and subcutaneous tissue of other sites |
| D17.4 | Benign lipomatous neoplasm of intrathoracic organs |
| D17.5 | Benign lipomatous neoplasm of intra-abdominal organs |
| D17.6 | Benign lipomatous neoplasm of spermatic cord |
| D17.7 | Benign lipomatous neoplasm of other sites |
| D17.71 | Benign lipomatous neoplasm of kidney |
| D17.72 | Benign lipomatous neoplasm of other genitourinary organ |
| D17.79 | Benign lipomatous neoplasm of other sites |
| D17.9 | Benign lipomatous neoplasm, unspecified |
| D18 | Hemangioma and lymphangioma, any site |
| D18.0 | Hemangioma |
| D18.00 | Hemangioma unspecified site |
| D18.01 | Hemangioma of skin and subcutaneous tissue |
| D18.02 | Hemangioma of intracranial structures |
| D18.03 | Hemangioma of intra-abdominal structures |
| D18.09 | Hemangioma of other sites |
| D18.1 | Lymphangioma, any site |
| D19 | Benign neoplasm of mesothelial tissue |
| D19.0 | Benign neoplasm of mesothelial tissue of pleura |
| D19.1 | Benign neoplasm of mesothelial tissue of peritoneum |
| D19.7 | Benign neoplasm of mesothelial tissue of other sites |
| D19.9 | Benign neoplasm of mesothelial tissue, unspecified |
| D20 | Benign neoplasm of soft tissue of retroperitoneum and peritoneum |
| D20.0 | Benign neoplasm of soft tissue of retroperitoneum |
| D20.1 | Benign neoplasm of soft tissue of peritoneum |
| D21 | Other benign neoplasms of connective and other soft tissue |
| D21.0 | Benign neoplasm of connective and other soft tissue of head, face and neck |
| D21.1 | Benign neoplasm of connective and other soft tissue of upper limb, including shoulder |
| D21.10 | Benign neoplasm of connective and other soft tissue of unspecified upper limb, including shoulder |
| D21.11 | Benign neoplasm of connective and other soft tissue of right upper limb, including shoulder |
| D21.12 | Benign neoplasm of connective and other soft tissue of left upper limb, including shoulder |
| D21.2 | Benign neoplasm of connective and other soft tissue of lower limb, including hip |
| D21.20 | Benign neoplasm of connective and other soft tissue of unspecified lower limb, including hip |
| D21.21 | Benign neoplasm of connective and other soft tissue of right lower limb, including hip |
| D21.22 | Benign neoplasm of connective and other soft tissue of left lower limb, including hip |
| D21.3 | Benign neoplasm of connective and other soft tissue of thorax |
| D21.4 | Benign neoplasm of connective and other soft tissue of abdomen |
| D21.5 | Benign neoplasm of connective and other soft tissue of pelvis |
| D21.6 | Benign neoplasm of connective and other soft tissue of trunk, unspecified |
| D21.9 | Benign neoplasm of connective and other soft tissue, unspecified |
| D22 | Melanocytic nevi |
| D22.0 | Melanocytic nevi of lip |
| D22.1 | Melanocytic nevi of eyelid, including canthus |
| D22.10 | Melanocytic nevi of unspecified eyelid, including canthus |
| D22.11 | Melanocytic nevi of right eyelid, including canthus |
| D22.111 | Melanocytic nevi of right upper eyelid, including canthus |
| D22.112 | Melanocytic nevi of right lower eyelid, including canthus |
| D22.12 | Melanocytic nevi of left eyelid, including canthus |
| D22.121 | Melanocytic nevi of left upper eyelid, including canthus |
| D22.122 | Melanocytic nevi of left lower eyelid, including canthus |
| D22.2 | Melanocytic nevi of ear and external auricular canal |
| D22.20 | Melanocytic nevi of unspecified ear and external auricular canal |
| D22.21 | Melanocytic nevi of right ear and external auricular canal |
| D22.22 | Melanocytic nevi of left ear and external auricular canal |
| D22.3 | Melanocytic nevi of other and unspecified parts of face |
| D22.30 | Melanocytic nevi of unspecified part of face |
| D22.39 | Melanocytic nevi of other parts of face |
| D22.4 | Melanocytic nevi of scalp and neck |
| D22.5 | Melanocytic nevi of trunk |
| D22.6 | Melanocytic nevi of upper limb, including shoulder |
| D22.60 | Melanocytic nevi of unspecified upper limb, including shoulder |
| D22.61 | Melanocytic nevi of right upper limb, including shoulder |
| D22.62 | Melanocytic nevi of left upper limb, including shoulder |
| D22.7 | Melanocytic nevi of lower limb, including hip |
| D22.70 | Melanocytic nevi of unspecified lower limb, including hip |
| D22.71 | Melanocytic nevi of right lower limb, including hip |
| D22.72 | Melanocytic nevi of left lower limb, including hip |
| D22.9 | Melanocytic nevi, unspecified |
| D23 | Other benign neoplasms of skin |
| D23.0 | Other benign neoplasm of skin of lip |
| D23.1 | Other benign neoplasm of skin of eyelid, including canthus |
| D23.10 | Other benign neoplasm of skin of unspecified eyelid, including canthus |
| D23.11 | Other benign neoplasm of skin of right eyelid, including canthus |
| D23.111 | Other benign neoplasm of skin of right upper eyelid, including canthus |
| D23.112 | Other benign neoplasm of skin of right lower eyelid, including canthus |
| D23.12 | Other benign neoplasm of skin of left eyelid, including canthus |
| D23.121 | Other benign neoplasm of skin of left upper eyelid, including canthus |
| D23.122 | Other benign neoplasm of skin of left lower eyelid, including canthus |
| D23.2 | Other benign neoplasm of skin of ear and external auricular canal |
| D23.20 | Other benign neoplasm of skin of unspecified ear and external auricular canal |
| D23.21 | Other benign neoplasm of skin of right ear and external auricular canal |
| D23.22 | Other benign neoplasm of skin of left ear and external auricular canal |
| D23.3 | Other benign neoplasm of skin of other and unspecified parts of face |
| D23.30 | Other benign neoplasm of skin of unspecified part of face |
| D23.39 | Other benign neoplasm of skin of other parts of face |
| D23.4 | Other benign neoplasm of skin of scalp and neck |
| D23.5 | Other benign neoplasm of skin of trunk |
| D23.6 | Other benign neoplasm of skin of upper limb, including shoulder |
| D23.60 | Other benign neoplasm of skin of unspecified upper limb, including shoulder |
| D23.61 | Other benign neoplasm of skin of right upper limb, including shoulder |
| D23.62 | Other benign neoplasm of skin of left upper limb, including shoulder |
| D23.7 | Other benign neoplasm of skin of lower limb, including hip |
| D23.70 | Other benign neoplasm of skin of unspecified lower limb, including hip |
| D23.71 | Other benign neoplasm of skin of right lower limb, including hip |
| D23.72 | Other benign neoplasm of skin of left lower limb, including hip |
| D23.9 | Other benign neoplasm of skin, unspecified |
| D24 | Benign neoplasm of breast |
| D24.1 | Benign neoplasm of right breast |
| D24.2 | Benign neoplasm of left breast |
| D24.9 | Benign neoplasm of unspecified breast |
| D25 | Leiomyoma of uterus |
| D25.0 | Submucous leiomyoma of uterus |
| D25.1 | Intramural leiomyoma of uterus |
| D25.2 | Subserosal leiomyoma of uterus |
| D25.9 | Leiomyoma of uterus, unspecified |
| D26 | Other benign neoplasms of uterus |
| D26.0 | Other benign neoplasm of cervix uteri |
| D26.1 | Other benign neoplasm of corpus uteri |
| D26.7 | Other benign neoplasm of other parts of uterus |
| D26.9 | Other benign neoplasm of uterus, unspecified |
| D27 | Benign neoplasm of ovary |
| D27.0 | Benign neoplasm of right ovary |
| D27.1 | Benign neoplasm of left ovary |
| D27.9 | Benign neoplasm of unspecified ovary |
| D28 | Benign neoplasm of other and unspecified female genital organs |
| D28.0 | Benign neoplasm of vulva |
| D28.1 | Benign neoplasm of vagina |
| D28.2 | Benign neoplasm of uterine tubes and ligaments |
| D28.7 | Benign neoplasm of other specified female genital organs |
| D28.9 | Benign neoplasm of female genital organ, unspecified |
| D29 | Benign neoplasm of male genital organs |
| D29.0 | Benign neoplasm of penis |
| D29.1 | Benign neoplasm of prostate |
| D29.2 | Benign neoplasm of testis |
| D29.20 | Benign neoplasm of unspecified testis |
| D29.21 | Benign neoplasm of right testis |
| D29.22 | Benign neoplasm of left testis |
| D29.3 | Benign neoplasm of epididymis |
| D29.30 | Benign neoplasm of unspecified epididymis |
| D29.31 | Benign neoplasm of right epididymis |
| D29.32 | Benign neoplasm of left epididymis |
| D29.4 | Benign neoplasm of scrotum |
| D29.8 | Benign neoplasm of other specified male genital organs |
| D29.9 | Benign neoplasm of male genital organ, unspecified |
| D30 | Benign neoplasm of urinary organs |
| D30.0 | Benign neoplasm of kidney |
| D30.00 | Benign neoplasm of unspecified kidney |
| D30.01 | Benign neoplasm of right kidney |
| D30.02 | Benign neoplasm of left kidney |
| D30.1 | Benign neoplasm of renal pelvis |
| D30.10 | Benign neoplasm of unspecified renal pelvis |
| D30.11 | Benign neoplasm of right renal pelvis |
| D30.12 | Benign neoplasm of left renal pelvis |
| D30.2 | Benign neoplasm of ureter |
| D30.20 | Benign neoplasm of unspecified ureter |
| D30.21 | Benign neoplasm of right ureter |
| D30.22 | Benign neoplasm of left ureter |
| D30.3 | Benign neoplasm of bladder |
| D30.4 | Benign neoplasm of urethra |
| D30.8 | Benign neoplasm of other specified urinary organs |
| D30.9 | Benign neoplasm of urinary organ, unspecified |
| D31 | Benign neoplasm of eye and adnexa |
| D31.0 | Benign neoplasm of conjunctiva |
| D31.00 | Benign neoplasm of unspecified conjunctiva |
| D31.01 | Benign neoplasm of right conjunctiva |
| D31.02 | Benign neoplasm of left conjunctiva |
| D31.1 | Benign neoplasm of cornea |
| D31.10 | Benign neoplasm of unspecified cornea |
| D31.11 | Benign neoplasm of right cornea |
| D31.12 | Benign neoplasm of left cornea |
| D31.2 | Benign neoplasm of retina |
| D31.20 | Benign neoplasm of unspecified retina |
| D31.21 | Benign neoplasm of right retina |
| D31.22 | Benign neoplasm of left retina |
| D31.3 | Benign neoplasm of choroid |
| D31.30 | Benign neoplasm of unspecified choroid |
| D31.31 | Benign neoplasm of right choroid |
| D31.32 | Benign neoplasm of left choroid |
| D31.4 | Benign neoplasm of ciliary body |
| D31.40 | Benign neoplasm of unspecified ciliary body |
| D31.41 | Benign neoplasm of right ciliary body |
| D31.42 | Benign neoplasm of left ciliary body |
| D31.5 | Benign neoplasm of lacrimal gland and duct |
| D31.50 | Benign neoplasm of unspecified lacrimal gland and duct |
| D31.51 | Benign neoplasm of right lacrimal gland and duct |
| D31.52 | Benign neoplasm of left lacrimal gland and duct |
| D31.6 | Benign neoplasm of unspecified site of orbit |
| D31.60 | Benign neoplasm of unspecified site of unspecified orbit |
| D31.61 | Benign neoplasm of unspecified site of right orbit |
| D31.62 | Benign neoplasm of unspecified site of left orbit |
| D31.9 | Benign neoplasm of unspecified part of eye |
| D31.90 | Benign neoplasm of unspecified part of unspecified eye |
| D31.91 | Benign neoplasm of unspecified part of right eye |
| D31.92 | Benign neoplasm of unspecified part of left eye |
| D32 | Benign neoplasm of meninges |
| D32.0 | Benign neoplasm of cerebral meninges |
| D32.1 | Benign neoplasm of spinal meninges |
| D32.9 | Benign neoplasm of meninges, unspecified |
| D33 | Benign neoplasm of brain and other parts of central nervous system |
| D33.0 | Benign neoplasm of brain, supratentorial |
| D33.1 | Benign neoplasm of brain, infratentorial |
| D33.2 | Benign neoplasm of brain, unspecified |
| D33.3 | Benign neoplasm of cranial nerves |
| D33.4 | Benign neoplasm of spinal cord |
| D33.7 | Benign neoplasm of other specified parts of central nervous system |
| D33.9 | Benign neoplasm of central nervous system, unspecified |
| D34 | Benign neoplasm of thyroid gland |
| D35 | Benign neoplasm of other and unspecified endocrine glands |
| D35.0 | Benign neoplasm of adrenal gland |
| D35.00 | Benign neoplasm of unspecified adrenal gland |
| D35.01 | Benign neoplasm of right adrenal gland |
| D35.02 | Benign neoplasm of left adrenal gland |
| D35.1 | Benign neoplasm of parathyroid gland |
| D35.2 | Benign neoplasm of pituitary gland |
| D35.3 | Benign neoplasm of craniopharyngeal duct |
| D35.4 | Benign neoplasm of pineal gland |
| D35.5 | Benign neoplasm of carotid body |
| D35.6 | Benign neoplasm of aortic body and other paraganglia |
| D35.7 | Benign neoplasm of other specified endocrine glands |
| D35.9 | Benign neoplasm of endocrine gland, unspecified |
| D36 | Benign neoplasm of other and unspecified sites |
| D36.0 | Benign neoplasm of lymph nodes |
| D36.1 | Benign neoplasm of peripheral nerves and autonomic nervous system |
| D36.10 | Benign neoplasm of peripheral nerves and autonomic nervous system, unspecified |
| D36.11 | Benign neoplasm of peripheral nerves and autonomic nervous system of face, head, and neck |
| D36.12 | Benign neoplasm of peripheral nerves and autonomic nervous system, upper limb, including shoulder |
| D36.13 | Benign neoplasm of peripheral nerves and autonomic nervous system of lower limb, including hip |
| D36.14 | Benign neoplasm of peripheral nerves and autonomic nervous system of thorax |
| D36.15 | Benign neoplasm of peripheral nerves and autonomic nervous system of abdomen |
| D36.16 | Benign neoplasm of peripheral nerves and autonomic nervous system of pelvis |
| D36.17 | Benign neoplasm of peripheral nerves and autonomic nervous system of trunk, unspecified |
| D36.7 | Benign neoplasm of other specified sites |
| D36.9 | Benign neoplasm, unspecified site |
| D37 | Neoplasm of uncertain behavior of oral cavity and digestive organs |
| D37.0 | Neoplasm of uncertain behavior of lip, oral cavity and pharynx |
| D37.01 | Neoplasm of uncertain behavior of lip |
| D37.02 | Neoplasm of uncertain behavior of tongue |
| D37.03 | Neoplasm of uncertain behavior of the major salivary glands |
| D37.030 | Neoplasm of uncertain behavior of the parotid salivary glands |
| D37.031 | Neoplasm of uncertain behavior of the sublingual salivary glands |
| D37.032 | Neoplasm of uncertain behavior of the submandibular salivary glands |
| D37.039 | Neoplasm of uncertain behavior of the major salivary glands, unspecified |
| D37.04 | Neoplasm of uncertain behavior of the minor salivary glands |
| D37.05 | Neoplasm of uncertain behavior of pharynx |
| D37.09 | Neoplasm of uncertain behavior of other specified sites of the oral cavity |
| D37.1 | Neoplasm of uncertain behavior of stomach |
| D37.2 | Neoplasm of uncertain behavior of small intestine |
| D37.3 | Neoplasm of uncertain behavior of appendix |
| D37.4 | Neoplasm of uncertain behavior of colon |
| D37.5 | Neoplasm of uncertain behavior of rectum |
| D37.6 | Neoplasm of uncertain behavior of liver, gallbladder and bile ducts |
| D37.8 | Neoplasm of uncertain behavior of other specified digestive organs |
| D37.9 | Neoplasm of uncertain behavior of digestive organ, unspecified |
| D38 | Neoplasm of uncertain behavior of middle ear and respiratory and intrathoracic organs |
| D38.0 | Neoplasm of uncertain behavior of larynx |
| D38.1 | Neoplasm of uncertain behavior of trachea, bronchus and lung |
| D38.2 | Neoplasm of uncertain behavior of pleura |
| D38.3 | Neoplasm of uncertain behavior of mediastinum |
| D38.4 | Neoplasm of uncertain behavior of thymus |
| D38.5 | Neoplasm of uncertain behavior of other respiratory organs |
| D38.6 | Neoplasm of uncertain behavior of respiratory organ, unspecified |
| D39 | Neoplasm of uncertain behavior of female genital organs |
| D39.0 | Neoplasm of uncertain behavior of uterus |
| D39.1 | Neoplasm of uncertain behavior of ovary |
| D39.10 | Neoplasm of uncertain behavior of unspecified ovary |
| D39.11 | Neoplasm of uncertain behavior of right ovary |
| D39.12 | Neoplasm of uncertain behavior of left ovary |
| D39.2 | Neoplasm of uncertain behavior of placenta |
| D39.8 | Neoplasm of uncertain behavior of other specified female genital organs |
| D39.9 | Neoplasm of uncertain behavior of female genital organ, unspecified |
| D40 | Neoplasm of uncertain behavior of male genital organs |
| D40.0 | Neoplasm of uncertain behavior of prostate |
| D40.1 | Neoplasm of uncertain behavior of testis |
| D40.10 | Neoplasm of uncertain behavior of unspecified testis |
| D40.11 | Neoplasm of uncertain behavior of right testis |
| D40.12 | Neoplasm of uncertain behavior of left testis |
| D40.8 | Neoplasm of uncertain behavior of other specified male genital organs |
| D40.9 | Neoplasm of uncertain behavior of male genital organ, unspecified |
| D41 | Neoplasm of uncertain behavior of urinary organs |
| D41.0 | Neoplasm of uncertain behavior of kidney |
| D41.00 | Neoplasm of uncertain behavior of unspecified kidney |
| D41.01 | Neoplasm of uncertain behavior of right kidney |
| D41.02 | Neoplasm of uncertain behavior of left kidney |
| D41.1 | Neoplasm of uncertain behavior of renal pelvis |
| D41.10 | Neoplasm of uncertain behavior of unspecified renal pelvis |
| D41.11 | Neoplasm of uncertain behavior of right renal pelvis |
| D41.12 | Neoplasm of uncertain behavior of left renal pelvis |
| D41.2 | Neoplasm of uncertain behavior of ureter |
| D41.20 | Neoplasm of uncertain behavior of unspecified ureter |
| D41.21 | Neoplasm of uncertain behavior of right ureter |
| D41.22 | Neoplasm of uncertain behavior of left ureter |
| D41.3 | Neoplasm of uncertain behavior of urethra |
| D41.4 | Neoplasm of uncertain behavior of bladder |
| D41.8 | Neoplasm of uncertain behavior of other specified urinary organs |
| D41.9 | Neoplasm of uncertain behavior of unspecified urinary organ |
| D42 | Neoplasm of uncertain behavior of meninges |
| D42.0 | Neoplasm of uncertain behavior of cerebral meninges |
| D42.1 | Neoplasm of uncertain behavior of spinal meninges |
| D42.9 | Neoplasm of uncertain behavior of meninges, unspecified |
| D43 | Neoplasm of uncertain behavior of brain and central nervous system |
| D43.0 | Neoplasm of uncertain behavior of brain, supratentorial |
| D43.1 | Neoplasm of uncertain behavior of brain, infratentorial |
| D43.2 | Neoplasm of uncertain behavior of brain, unspecified |
| D43.3 | Neoplasm of uncertain behavior of cranial nerves |
| D43.4 | Neoplasm of uncertain behavior of spinal cord |
| D43.8 | Neoplasm of uncertain behavior of other specified parts of central nervous system |
| D43.9 | Neoplasm of uncertain behavior of central nervous system, unspecified |
| D44 | Neoplasm of uncertain behavior of endocrine glands |
| D44.0 | Neoplasm of uncertain behavior of thyroid gland |
| D44.1 | Neoplasm of uncertain behavior of adrenal gland |
| D44.10 | Neoplasm of uncertain behavior of unspecified adrenal gland |
| D44.11 | Neoplasm of uncertain behavior of right adrenal gland |
| D44.12 | Neoplasm of uncertain behavior of left adrenal gland |
| D44.2 | Neoplasm of uncertain behavior of parathyroid gland |
| D44.3 | Neoplasm of uncertain behavior of pituitary gland |
| D44.4 | Neoplasm of uncertain behavior of craniopharyngeal duct |
| D44.5 | Neoplasm of uncertain behavior of pineal gland |
| D44.6 | Neoplasm of uncertain behavior of carotid body |
| D44.7 | Neoplasm of uncertain behavior of aortic body and other paraganglia |
| D44.9 | Neoplasm of uncertain behavior of unspecified endocrine gland |
| D45 | Polycythemia vera |
| D46 | Myelodysplastic syndromes |
| D46.0 | Refractory anemia without ring sideroblasts, so stated |
| D46.1 | Refractory anemia with ring sideroblasts |
| D46.2 | Refractory anemia with excess of blasts [RAEB] |
| D46.20 | Refractory anemia with excess of blasts, unspecified |
| D46.21 | Refractory anemia with excess of blasts 1 |
| D46.22 | Refractory anemia with excess of blasts 2 |
| D46.4 | Refractory anemia, unspecified |
| D46.9 | Myelodysplastic syndrome, unspecified |
| D46.A | Refractory cytopenia with multilineage dysplasia |
| D46.B | Refractory cytopenia with multilineage dysplasia and ring sideroblasts |
| D46.C | Myelodysplastic syndrome with isolated del(5q) chromosomal abnormality |
| D46.Z | Other myelodysplastic syndromes |
| D47 | Other neoplasms of uncertain behavior of lymphoid, hematopoietic and related tissue |
| D47.0 | Mast cell neoplasms of uncertain behavior |
| D47.01 | Cutaneous mastocytosis |
| D47.02 | Systemic mastocytosis |
| D47.09 | Other mast cell neoplasms of uncertain behavior |
| D47.1 | Chronic myeloproliferative disease |
| D47.2 | Monoclonal gammopathy |
| D47.3 | Essential (hemorrhagic) thrombocythemia |
| D47.4 | Osteomyelofibrosis |
| D47.9 | Neoplasm of uncertain behavior of lymphoid, hematopoietic and related tissue, unspecified |
| D47.Z | Other specified neoplasms of uncertain behavior of lymphoid, hematopoietic and related tissue |
| D47.Z1 | Post-transplant lymphoproliferative disorder (PTLD) |
| D47.Z2 | Castleman disease |
| D47.Z9 | Other specified neoplasms of uncertain behavior of lymphoid, hematopoietic and related tissue |
| D48 | Neoplasm of uncertain behavior of other and unspecified sites |
| D48.0 | Neoplasm of uncertain behavior of bone and articular cartilage |
| D48.1 | Neoplasm of uncertain behavior of connective and other soft tissue |
| D48.2 | Neoplasm of uncertain behavior of peripheral nerves and autonomic nervous system |
| D48.3 | Neoplasm of uncertain behavior of retroperitoneum |
| D48.4 | Neoplasm of uncertain behavior of peritoneum |
| D48.5 | Neoplasm of uncertain behavior of skin |
| D48.6 | Neoplasm of uncertain behavior of breast |
| D48.60 | Neoplasm of uncertain behavior of unspecified breast |
| D48.61 | Neoplasm of uncertain behavior of right breast |
| D48.62 | Neoplasm of uncertain behavior of left breast |
| D48.7 | Neoplasm of uncertain behavior of other specified sites |
| D48.9 | Neoplasm of uncertain behavior, unspecified |
| D49 | Neoplasms of unspecified behavior |
| D49.0 | Neoplasm of unspecified behavior of digestive system |
| D49.1 | Neoplasm of unspecified behavior of respiratory system |
| D49.2 | Neoplasm of unspecified behavior of bone, soft tissue, and skin |
| D49.3 | Neoplasm of unspecified behavior of breast |
| D49.4 | Neoplasm of unspecified behavior of bladder |
| D49.5 | Neoplasm of unspecified behavior of other genitourinary organs |
| D49.51 | Neoplasm of unspecified behavior of kidney |
| D49.511 | Neoplasm of unspecified behavior of right kidney |
| D49.512 | Neoplasm of unspecified behavior of left kidney |
| D49.519 | Neoplasm of unspecified behavior of unspecified kidney |
| D49.59 | Neoplasm of unspecified behavior of other genitourinary organ |
| D49.6 | Neoplasm of unspecified behavior of brain |
| D49.7 | Neoplasm of unspecified behavior of endocrine glands and other parts of nervous system |
| D49.8 | Neoplasm of unspecified behavior of other specified sites |
| D49.81 | Neoplasm of unspecified behavior of retina and choroid |
| D49.89 | Neoplasm of unspecified behavior of other specified sites |
| D49.9 | Neoplasm of unspecified behavior of unspecified site |
| G54 | Nerve root and plexus disorders |
| G54.0 | Brachial plexus disorders |
| G54.1 | Lumbosacral plexus disorders |
| G54.2 | Cervical root disorders, not elsewhere classified |
| G54.3 | Thoracic root disorders, not elsewhere classified |
| G54.4 | Lumbosacral root disorders, not elsewhere classified |
| G54.5 | Neuralgic amyotrophy |
| G54.6 | Phantom limb syndrome with pain |
| G54.7 | Phantom limb syndrome without pain |
| G54.8 | Other nerve root and plexus disorders |
| G54.9 | Nerve root and plexus disorder, unspecified |
| G83 | Other paralytic syndromes |
| G83.0 | Diplegia of upper limbs |
| G83.1 | Monoplegia of lower limb |
| G83.10 | Monoplegia of lower limb affecting unspecified side |
| G83.11 | Monoplegia of lower limb affecting right dominant side |
| G83.12 | Monoplegia of lower limb affecting left dominant side |
| G83.13 | Monoplegia of lower limb affecting right nondominant side |
| G83.14 | Monoplegia of lower limb affecting left nondominant side |
| G83.2 | Monoplegia of upper limb |
| G83.20 | Monoplegia of upper limb affecting unspecified side |
| G83.21 | Monoplegia of upper limb affecting right dominant side |
| G83.22 | Monoplegia of upper limb affecting left dominant side |
| G83.23 | Monoplegia of upper limb affecting right nondominant side |
| G83.24 | Monoplegia of upper limb affecting left nondominant side |
| G83.3 | Monoplegia, unspecified |
| G83.30 | Monoplegia, unspecified affecting unspecified side |
| G83.31 | Monoplegia, unspecified affecting right dominant side |
| G83.32 | Monoplegia, unspecified affecting left dominant side |
| G83.33 | Monoplegia, unspecified affecting right nondominant side |
| G83.34 | Monoplegia, unspecified affecting left nondominant side |
| G83.4 | Cauda equina syndrome |
| G83.5 | Locked-in state |
| G83.8 | Other specified paralytic syndromes |
| G83.81 | Brown-Sequard syndrome |
| G83.82 | Anterior cord syndrome |
| G83.83 | Posterior cord syndrome |
| G83.84 | Todd's paralysis (postepileptic) |
| G83.89 | Other specified paralytic syndromes |
| G83.9 | Paralytic syndrome, unspecified |
| I71.1 | Thoracic aortic aneurysm, ruptured |
| I71.2 | Thoracic aortic aneurysm, without rupture |
| I71.3 | Abdominal aortic aneurysm, ruptured |
| I71.4 | Abdominal aortic aneurysm, without rupture |
| I71.5 | Thoracoabdominal aortic aneurysm, ruptured |
| I71.6 | Thoracoabdominal aortic aneurysm, without rupture |
| I71.8 | Aortic aneurysm of unspecified site, ruptured |
| I71.9 | Aortic aneurysm of unspecified site, without rupture |
| I72.0 | Aneurysm of carotid artery |
| I72.1 | Aneurysm of artery of upper extremity |
| I72.2 | Aneurysm of renal artery |
| I72.3 | Aneurysm of iliac artery |
| I72.4 | Aneurysm of artery of lower extremity |
| I72.5 | Aneurysm of other precerebral arteries |
| I72.6 | Aneurysm of vertebral artery |
| I72.8 | Aneurysm of other specified arteries |
| I72.9 | Aneurysm of unspecified site |
| I79.0 | Aneurysm of aorta in diseases classified elsewhere |
| M45 | Ankylosing spondylitis |
| M45.0 | Ankylosing spondylitis of multiple sites in spine |
| M45.1 | Ankylosing spondylitis of occipito-atlanto-axial region |
| M45.2 | Ankylosing spondylitis of cervical region |
| M45.3 | Ankylosing spondylitis of cervicothoracic region |
| M45.4 | Ankylosing spondylitis of thoracic region |
| M45.5 | Ankylosing spondylitis of thoracolumbar region |
| M45.6 | Ankylosing spondylitis lumbar region |
| M45.7 | Ankylosing spondylitis of lumbosacral region |
| M45.8 | Ankylosing spondylitis sacral and sacrococcygeal region |
| M45.9 | Ankylosing spondylitis of unspecified sites in spine |
| M46 | Other inflammatory spondylopathies |
| M46.0 | Spinal enthesopathy |
| M46.00 | Spinal enthesopathy, site unspecified |
| M46.01 | Spinal enthesopathy, occipito-atlanto-axial region |
| M46.02 | Spinal enthesopathy, cervical region |
| M46.03 | Spinal enthesopathy, cervicothoracic region |
| M46.04 | Spinal enthesopathy, thoracic region |
| M46.05 | Spinal enthesopathy, thoracolumbar region |
| M46.06 | Spinal enthesopathy, lumbar region |
| M46.07 | Spinal enthesopathy, lumbosacral region |
| M46.08 | Spinal enthesopathy, sacral and sacrococcygeal region |
| M46.09 | Spinal enthesopathy, multiple sites in spine |
| M46.1 | Sacroiliitis, not elsewhere classified |
| M46.2 | Osteomyelitis of vertebra |
| M46.20 | Osteomyelitis of vertebra, site unspecified |
| M46.21 | Osteomyelitis of vertebra, occipito-atlanto-axial region |
| M46.22 | Osteomyelitis of vertebra, cervical region |
| M46.23 | Osteomyelitis of vertebra, cervicothoracic region |
| M46.24 | Osteomyelitis of vertebra, thoracic region |
| M46.25 | Osteomyelitis of vertebra, thoracolumbar region |
| M46.26 | Osteomyelitis of vertebra, lumbar region |
| M46.27 | Osteomyelitis of vertebra, lumbosacral region |
| M46.28 | Osteomyelitis of vertebra, sacral and sacrococcygeal region |
| M46.3 | Infection of intervertebral disc (pyogenic) |
| M46.30 | Infection of intervertebral disc (pyogenic), site unspecified |
| M46.31 | Infection of intervertebral disc (pyogenic), occipito-atlanto-axial region |
| M46.32 | Infection of intervertebral disc (pyogenic), cervical region |
| M46.33 | Infection of intervertebral disc (pyogenic), cervicothoracic region |
| M46.34 | Infection of intervertebral disc (pyogenic), thoracic region |
| M46.35 | Infection of intervertebral disc (pyogenic), thoracolumbar region |
| M46.36 | Infection of intervertebral disc (pyogenic), lumbar region |
| M46.37 | Infection of intervertebral disc (pyogenic), lumbosacral region |
| M46.38 | Infection of intervertebral disc (pyogenic), sacral and sacrococcygeal region |
| M46.39 | Infection of intervertebral disc (pyogenic), multiple sites in spine |
| M46.4 | Discitis, unspecified |
| M46.40 | Discitis, unspecified, site unspecified |
| M46.41 | Discitis, unspecified, occipito-atlanto-axial region |
| M46.42 | Discitis, unspecified, cervical region |
| M46.43 | Discitis, unspecified, cervicothoracic region |
| M46.44 | Discitis, unspecified, thoracic region |
| M46.45 | Discitis, unspecified, thoracolumbar region |
| M46.46 | Discitis, unspecified, lumbar region |
| M46.47 | Discitis, unspecified, lumbosacral region |
| M46.48 | Discitis, unspecified, sacral and sacrococcygeal region |
| M46.49 | Discitis, unspecified, multiple sites in spine |
| M46.5 | Other infective spondylopathies |
| M46.50 | Other infective spondylopathies, site unspecified |
| M46.51 | Other infective spondylopathies, occipito-atlanto-axial region |
| M46.52 | Other infective spondylopathies, cervical region |
| M46.53 | Other infective spondylopathies, cervicothoracic region |
| M46.54 | Other infective spondylopathies, thoracic region |
| M46.55 | Other infective spondylopathies, thoracolumbar region |
| M46.56 | Other infective spondylopathies, lumbar region |
| M46.57 | Other infective spondylopathies, lumbosacral region |
| M46.58 | Other infective spondylopathies, sacral and sacrococcygeal region |
| M46.59 | Other infective spondylopathies, multiple sites in spine |
| M46.8 | Other specified inflammatory spondylopathies |
| M46.80 | Other specified inflammatory spondylopathies, site unspecified |
| M46.81 | Other specified inflammatory spondylopathies, occipito-atlanto-axial region |
| M46.82 | Other specified inflammatory spondylopathies, cervical region |
| M46.83 | Other specified inflammatory spondylopathies, cervicothoracic region |
| M46.84 | Other specified inflammatory spondylopathies, thoracic region |
| M46.85 | Other specified inflammatory spondylopathies, thoracolumbar region |
| M46.86 | Other specified inflammatory spondylopathies, lumbar region |
| M46.87 | Other specified inflammatory spondylopathies, lumbosacral region |
| M46.88 | Other specified inflammatory spondylopathies, sacral and sacrococcygeal region |
| M46.89 | Other specified inflammatory spondylopathies, multiple sites in spine |
| M46.9 | Unspecified inflammatory spondylopathy |
| M46.90 | Unspecified inflammatory spondylopathy, site unspecified |
| M46.91 | Unspecified inflammatory spondylopathy, occipito-atlanto-axial region |
| M46.92 | Unspecified inflammatory spondylopathy, cervical region |
| M46.93 | Unspecified inflammatory spondylopathy, cervicothoracic region |
| M46.94 | Unspecified inflammatory spondylopathy, thoracic region |
| M46.95 | Unspecified inflammatory spondylopathy, thoracolumbar region |
| M46.96 | Unspecified inflammatory spondylopathy, lumbar region |
| M46.97 | Unspecified inflammatory spondylopathy, lumbosacral region |
| M46.98 | Unspecified inflammatory spondylopathy, sacral and sacrococcygeal region |
| M46.99 | Unspecified inflammatory spondylopathy, multiple sites in spine |
| M48.4 | Fatigue fracture of vertebra |
| M48.40 | Fatigue fracture of vertebra, site unspecified |
| M48.40XA | Fatigue fracture of vertebra, site unspecified, initial encounter for fracture |
| M48.40XD | Fatigue fracture of vertebra, site unspecified, subsequent encounter for fracture with routine healing |
| M48.40XG | Fatigue fracture of vertebra, site unspecified, subsequent encounter for fracture with delayed healing |
| M48.40XS | Fatigue fracture of vertebra, site unspecified, sequela of fracture |
| M48.41 | Fatigue fracture of vertebra, occipito-atlanto-axial region |
| M48.41XA | Fatigue fracture of vertebra, occipito-atlanto-axial region, initial encounter for fracture |
| M48.41XD | Fatigue fracture of vertebra, occipito-atlanto-axial region, subsequent encounter for fracture with routine healing |
| M48.41XG | Fatigue fracture of vertebra, occipito-atlanto-axial region, subsequent encounter for fracture with delayed healing |
| M48.41XS | Fatigue fracture of vertebra, occipito-atlanto-axial region, sequela of fracture |
| M48.42 | Fatigue fracture of vertebra, cervical region |
| M48.42XA | Fatigue fracture of vertebra, cervical region, initial encounter for fracture |
| M48.42XD | Fatigue fracture of vertebra, cervical region, subsequent encounter for fracture with routine healing |
| M48.42XG | Fatigue fracture of vertebra, cervical region, subsequent encounter for fracture with delayed healing |
| M48.42XS | Fatigue fracture of vertebra, cervical region, sequela of fracture |
| M48.43 | Fatigue fracture of vertebra, cervicothoracic region |
| M48.43XA | Fatigue fracture of vertebra, cervicothoracic region, initial encounter for fracture |
| M48.43XD | Fatigue fracture of vertebra, cervicothoracic region, subsequent encounter for fracture with routine healing |
| M48.43XG | Fatigue fracture of vertebra, cervicothoracic region, subsequent encounter for fracture with delayed healing |
| M48.43XS | Fatigue fracture of vertebra, cervicothoracic region, sequela of fracture |
| M48.44 | Fatigue fracture of vertebra, thoracic region |
| M48.44XA | Fatigue fracture of vertebra, thoracic region, initial encounter for fracture |
| M48.44XD | Fatigue fracture of vertebra, thoracic region, subsequent encounter for fracture with routine healing |
| M48.44XG | Fatigue fracture of vertebra, thoracic region, subsequent encounter for fracture with delayed healing |
| M48.44XS | Fatigue fracture of vertebra, thoracic region, sequela of fracture |
| M48.45 | Fatigue fracture of vertebra, thoracolumbar region |
| M48.45XA | Fatigue fracture of vertebra, thoracolumbar region, initial encounter for fracture |
| M48.45XD | Fatigue fracture of vertebra, thoracolumbar region, subsequent encounter for fracture with routine healing |
| M48.45XG | Fatigue fracture of vertebra, thoracolumbar region, subsequent encounter for fracture with delayed healing |
| M48.45XS | Fatigue fracture of vertebra, thoracolumbar region, sequela of fracture |
| M48.46 | Fatigue fracture of vertebra, lumbar region |
| M48.46XA | Fatigue fracture of vertebra, lumbar region, initial encounter for fracture |
| M48.46XD | Fatigue fracture of vertebra, lumbar region, subsequent encounter for fracture with routine healing |
| M48.46XG | Fatigue fracture of vertebra, lumbar region, subsequent encounter for fracture with delayed healing |
| M48.46XS | Fatigue fracture of vertebra, lumbar region, sequela of fracture |
| M48.47 | Fatigue fracture of vertebra, lumbosacral region |
| M48.47XA | Fatigue fracture of vertebra, lumbosacral region, initial encounter for fracture |
| M48.47XD | Fatigue fracture of vertebra, lumbosacral region, subsequent encounter for fracture with routine healing |
| M48.47XG | Fatigue fracture of vertebra, lumbosacral region, subsequent encounter for fracture with delayed healing |
| M48.47XS | Fatigue fracture of vertebra, lumbosacral region, sequela of fracture |
| M48.48 | Fatigue fracture of vertebra, sacral and sacrococcygeal region |
| M48.48XA | Fatigue fracture of vertebra, sacral and sacrococcygeal region, initial encounter for fracture |
| M48.48XD | Fatigue fracture of vertebra, sacral and sacrococcygeal region, subsequent encounter for fracture with routine healing |
| M48.48XG | Fatigue fracture of vertebra, sacral and sacrococcygeal region, subsequent encounter for fracture with delayed healing |
| M48.48XS | Fatigue fracture of vertebra, sacral and sacrococcygeal region, sequela of fracture |
| M48.50XA | Collapsed vertebra, not elsewhere classified, site unspecified, initial encounter for fracture |
| M48.50XD | Collapsed vertebra, not elsewhere classified, site unspecified, subsequent encounter for fracture with routine healing |
| M48.50XG | Collapsed vertebra, not elsewhere classified, site unspecified, subsequent encounter for fracture with delayed healing |
| M48.50XS | Collapsed vertebra, not elsewhere classified, site unspecified, sequela of fracture |
| M48.51XA | Collapsed vertebra, not elsewhere classified, occipito-atlanto-axial region, initial encounter for fracture |
| M48.51XD | Collapsed vertebra, not elsewhere classified, occipito-atlanto-axial region, subsequent encounter for fracture with routine healing |
| M48.51XG | Collapsed vertebra, not elsewhere classified, occipito-atlanto-axial region, subsequent encounter for fracture with delayed healing |
| M48.51XS | Collapsed vertebra, not elsewhere classified, occipito-atlanto-axial region, sequela of fracture |
| M48.52XA | Collapsed vertebra, not elsewhere classified, cervical region, initial encounter for fracture |
| M48.52XD | Collapsed vertebra, not elsewhere classified, cervical region, subsequent encounter for fracture with routine healing |
| M48.52XG | Collapsed vertebra, not elsewhere classified, cervical region, subsequent encounter for fracture with delayed healing |
| M48.52XS | Collapsed vertebra, not elsewhere classified, cervical region, sequela of fracture |
| M48.53XA | Collapsed vertebra, not elsewhere classified, cervicothoracic region, initial encounter for fracture |
| M48.53XD | Collapsed vertebra, not elsewhere classified, cervicothoracic region, subsequent encounter for fracture with routine healing |
| M48.53XG | Collapsed vertebra, not elsewhere classified, cervicothoracic region, subsequent encounter for fracture with delayed healing |
| M48.53XS | Collapsed vertebra, not elsewhere classified, cervicothoracic region, sequela of fracture |
| M48.54XA | Collapsed vertebra, not elsewhere classified, thoracic region, initial encounter for fracture |
| M48.54XD | Collapsed vertebra, not elsewhere classified, thoracic region, subsequent encounter for fracture with routine healing |
| M48.54XG | Collapsed vertebra, not elsewhere classified, thoracic region, subsequent encounter for fracture with delayed healing |
| M48.54XS | Collapsed vertebra, not elsewhere classified, thoracic region, sequela of fracture |
| M48.55XA | Collapsed vertebra, not elsewhere classified, thoracolumbar region, initial encounter for fracture |
| M48.55XD | Collapsed vertebra, not elsewhere classified, thoracolumbar region, subsequent encounter for fracture with routine healing |
| M48.55XG | Collapsed vertebra, not elsewhere classified, thoracolumbar region, subsequent encounter for fracture with delayed healing |
| M48.55XS | Collapsed vertebra, not elsewhere classified, thoracolumbar region, sequela of fracture |
| M48.56XA | Collapsed vertebra, not elsewhere classified, lumbar region, initial encounter for fracture |
| M48.56XD | Collapsed vertebra, not elsewhere classified, lumbar region, subsequent encounter for fracture with routine healing |
| M48.56XG | Collapsed vertebra, not elsewhere classified, lumbar region, subsequent encounter for fracture with delayed healing |
| M48.56XS | Collapsed vertebra, not elsewhere classified, lumbar region, sequela of fracture |
| M48.57XA | Collapsed vertebra, not elsewhere classified, lumbosacral region, initial encounter for fracture |
| M48.57XD | Collapsed vertebra, not elsewhere classified, lumbosacral region, subsequent encounter for fracture with routine healing |
| M48.57XG | Collapsed vertebra, not elsewhere classified, lumbosacral region, subsequent encounter for fracture with delayed healing |
| M48.57XS | Collapsed vertebra, not elsewhere classified, lumbosacral region, sequela of fracture |
| M48.58XA | Collapsed vertebra, not elsewhere classified, sacral and sacrococcygeal region, initial encounter for fracture |
| M48.58XD | Collapsed vertebra, not elsewhere classified, sacral and sacrococcygeal region, subsequent encounter for fracture with routine healing |
| M48.58XG | Collapsed vertebra, not elsewhere classified, sacral and sacrococcygeal region, subsequent encounter for fracture with delayed healing |
| M48.58XS | Collapsed vertebra, not elsewhere classified, sacral and sacrococcygeal region, sequela of fracture |
| M80 | Osteoporosis with current pathological fracture |
| M80.0 | Age-related osteoporosis with current pathological fracture |
| M80.00 | Age-related osteoporosis with current pathological fracture, unspecified site |
| M80.00XA | Age-related osteoporosis with current pathological fracture, unspecified site, initial encounter for fracture |
| M80.00XD | Age-related osteoporosis with current pathological fracture, unspecified site, subsequent encounter for fracture with routine healing |
| M80.00XG | Age-related osteoporosis with current pathological fracture, unspecified site, subsequent encounter for fracture with delayed healing |
| M80.00XK | Age-related osteoporosis with current pathological fracture, unspecified site, subsequent encounter for fracture with nonunion |
| M80.00XP | Age-related osteoporosis with current pathological fracture, unspecified site, subsequent encounter for fracture with malunion |
| M80.00XS | Age-related osteoporosis with current pathological fracture, unspecified site, sequela |
| M80.01 | Age-related osteoporosis with current pathological fracture, shoulder |
| M80.011 | Age-related osteoporosis with current pathological fracture, right shoulder |
| M80.011A | Age-related osteoporosis with current pathological fracture, right shoulder, initial encounter for fracture |
| M80.011D | Age-related osteoporosis with current pathological fracture, right shoulder, subsequent encounter for fracture with routine healing |
| M80.011G | Age-related osteoporosis with current pathological fracture, right shoulder, subsequent encounter for fracture with delayed healing |
| M80.011K | Age-related osteoporosis with current pathological fracture, right shoulder, subsequent encounter for fracture with nonunion |
| M80.011P | Age-related osteoporosis with current pathological fracture, right shoulder, subsequent encounter for fracture with malunion |
| M80.011S | Age-related osteoporosis with current pathological fracture, right shoulder, sequela |
| M80.012 | Age-related osteoporosis with current pathological fracture, left shoulder |
| M80.012A | Age-related osteoporosis with current pathological fracture, left shoulder, initial encounter for fracture |
| M80.012D | Age-related osteoporosis with current pathological fracture, left shoulder, subsequent encounter for fracture with routine healing |
| M80.012G | Age-related osteoporosis with current pathological fracture, left shoulder, subsequent encounter for fracture with delayed healing |
| M80.012K | Age-related osteoporosis with current pathological fracture, left shoulder, subsequent encounter for fracture with nonunion |
| M80.012P | Age-related osteoporosis with current pathological fracture, left shoulder, subsequent encounter for fracture with malunion |
| M80.012S | Age-related osteoporosis with current pathological fracture, left shoulder, sequela |
| M80.019 | Age-related osteoporosis with current pathological fracture, unspecified shoulder |
| M80.019A | Age-related osteoporosis with current pathological fracture, unspecified shoulder, initial encounter for fracture |
| M80.019D | Age-related osteoporosis with current pathological fracture, unspecified shoulder, subsequent encounter for fracture with routine healing |
| M80.019G | Age-related osteoporosis with current pathological fracture, unspecified shoulder, subsequent encounter for fracture with delayed healing |
| M80.019K | Age-related osteoporosis with current pathological fracture, unspecified shoulder, subsequent encounter for fracture with nonunion |
| M80.019P | Age-related osteoporosis with current pathological fracture, unspecified shoulder, subsequent encounter for fracture with malunion |
| M80.019S | Age-related osteoporosis with current pathological fracture, unspecified shoulder, sequela |
| M80.02 | Age-related osteoporosis with current pathological fracture, humerus |
| M80.021 | Age-related osteoporosis with current pathological fracture, right humerus |
| M80.021A | Age-related osteoporosis with current pathological fracture, right humerus, initial encounter for fracture |
| M80.021D | Age-related osteoporosis with current pathological fracture, right humerus, subsequent encounter for fracture with routine healing |
| M80.021G | Age-related osteoporosis with current pathological fracture, right humerus, subsequent encounter for fracture with delayed healing |
| M80.021K | Age-related osteoporosis with current pathological fracture, right humerus, subsequent encounter for fracture with nonunion |
| M80.021P | Age-related osteoporosis with current pathological fracture, right humerus, subsequent encounter for fracture with malunion |
| M80.021S | Age-related osteoporosis with current pathological fracture, right humerus, sequela |
| M80.022 | Age-related osteoporosis with current pathological fracture, left humerus |
| M80.022A | Age-related osteoporosis with current pathological fracture, left humerus, initial encounter for fracture |
| M80.022D | Age-related osteoporosis with current pathological fracture, left humerus, subsequent encounter for fracture with routine healing |
| M80.022G | Age-related osteoporosis with current pathological fracture, left humerus, subsequent encounter for fracture with delayed healing |
| M80.022K | Age-related osteoporosis with current pathological fracture, left humerus, subsequent encounter for fracture with nonunion |
| M80.022P | Age-related osteoporosis with current pathological fracture, left humerus, subsequent encounter for fracture with malunion |
| M80.022S | Age-related osteoporosis with current pathological fracture, left humerus, sequela |
| M80.029 | Age-related osteoporosis with current pathological fracture, unspecified humerus |
| M80.029A | Age-related osteoporosis with current pathological fracture, unspecified humerus, initial encounter for fracture |
| M80.029D | Age-related osteoporosis with current pathological fracture, unspecified humerus, subsequent encounter for fracture with routine healing |
| M80.029G | Age-related osteoporosis with current pathological fracture, unspecified humerus, subsequent encounter for fracture with delayed healing |
| M80.029K | Age-related osteoporosis with current pathological fracture, unspecified humerus, subsequent encounter for fracture with nonunion |
| M80.029P | Age-related osteoporosis with current pathological fracture, unspecified humerus, subsequent encounter for fracture with malunion |
| M80.029S | Age-related osteoporosis with current pathological fracture, unspecified humerus, sequela |
| M80.03 | Age-related osteoporosis with current pathological fracture, forearm |
| M80.031 | Age-related osteoporosis with current pathological fracture, right forearm |
| M80.031A | Age-related osteoporosis with current pathological fracture, right forearm, initial encounter for fracture |
| M80.031D | Age-related osteoporosis with current pathological fracture, right forearm, subsequent encounter for fracture with routine healing |
| M80.031G | Age-related osteoporosis with current pathological fracture, right forearm, subsequent encounter for fracture with delayed healing |
| M80.031K | Age-related osteoporosis with current pathological fracture, right forearm, subsequent encounter for fracture with nonunion |
| M80.031P | Age-related osteoporosis with current pathological fracture, right forearm, subsequent encounter for fracture with malunion |
| M80.031S | Age-related osteoporosis with current pathological fracture, right forearm, sequela |
| M80.032 | Age-related osteoporosis with current pathological fracture, left forearm |
| M80.032A | Age-related osteoporosis with current pathological fracture, left forearm, initial encounter for fracture |
| M80.032D | Age-related osteoporosis with current pathological fracture, left forearm, subsequent encounter for fracture with routine healing |
| M80.032G | Age-related osteoporosis with current pathological fracture, left forearm, subsequent encounter for fracture with delayed healing |
| M80.032K | Age-related osteoporosis with current pathological fracture, left forearm, subsequent encounter for fracture with nonunion |
| M80.032P | Age-related osteoporosis with current pathological fracture, left forearm, subsequent encounter for fracture with malunion |
| M80.032S | Age-related osteoporosis with current pathological fracture, left forearm, sequela |
| M80.039 | Age-related osteoporosis with current pathological fracture, unspecified forearm |
| M80.039A | Age-related osteoporosis with current pathological fracture, unspecified forearm, initial encounter for fracture |
| M80.039D | Age-related osteoporosis with current pathological fracture, unspecified forearm, subsequent encounter for fracture with routine healing |
| M80.039G | Age-related osteoporosis with current pathological fracture, unspecified forearm, subsequent encounter for fracture with delayed healing |
| M80.039K | Age-related osteoporosis with current pathological fracture, unspecified forearm, subsequent encounter for fracture with nonunion |
| M80.039P | Age-related osteoporosis with current pathological fracture, unspecified forearm, subsequent encounter for fracture with malunion |
| M80.039S | Age-related osteoporosis with current pathological fracture, unspecified forearm, sequela |
| M80.04 | Age-related osteoporosis with current pathological fracture, hand |
| M80.041 | Age-related osteoporosis with current pathological fracture, right hand |
| M80.041A | Age-related osteoporosis with current pathological fracture, right hand, initial encounter for fracture |
| M80.041D | Age-related osteoporosis with current pathological fracture, right hand, subsequent encounter for fracture with routine healing |
| M80.041G | Age-related osteoporosis with current pathological fracture, right hand, subsequent encounter for fracture with delayed healing |
| M80.041K | Age-related osteoporosis with current pathological fracture, right hand, subsequent encounter for fracture with nonunion |
| M80.041P | Age-related osteoporosis with current pathological fracture, right hand, subsequent encounter for fracture with malunion |
| M80.041S | Age-related osteoporosis with current pathological fracture, right hand, sequela |
| M80.042 | Age-related osteoporosis with current pathological fracture, left hand |
| M80.042A | Age-related osteoporosis with current pathological fracture, left hand, initial encounter for fracture |
| M80.042D | Age-related osteoporosis with current pathological fracture, left hand, subsequent encounter for fracture with routine healing |
| M80.042G | Age-related osteoporosis with current pathological fracture, left hand, subsequent encounter for fracture with delayed healing |
| M80.042K | Age-related osteoporosis with current pathological fracture, left hand, subsequent encounter for fracture with nonunion |
| M80.042P | Age-related osteoporosis with current pathological fracture, left hand, subsequent encounter for fracture with malunion |
| M80.042S | Age-related osteoporosis with current pathological fracture, left hand, sequela |
| M80.049 | Age-related osteoporosis with current pathological fracture, unspecified hand |
| M80.049A | Age-related osteoporosis with current pathological fracture, unspecified hand, initial encounter for fracture |
| M80.049D | Age-related osteoporosis with current pathological fracture, unspecified hand, subsequent encounter for fracture with routine healing |
| M80.049G | Age-related osteoporosis with current pathological fracture, unspecified hand, subsequent encounter for fracture with delayed healing |
| M80.049K | Age-related osteoporosis with current pathological fracture, unspecified hand, subsequent encounter for fracture with nonunion |
| M80.049P | Age-related osteoporosis with current pathological fracture, unspecified hand, subsequent encounter for fracture with malunion |
| M80.049S | Age-related osteoporosis with current pathological fracture, unspecified hand, sequela |
| M80.05 | Age-related osteoporosis with current pathological fracture, femur |
| M80.051 | Age-related osteoporosis with current pathological fracture, right femur |
| M80.051A | Age-related osteoporosis with current pathological fracture, right femur, initial encounter for fracture |
| M80.051D | Age-related osteoporosis with current pathological fracture, right femur, subsequent encounter for fracture with routine healing |
| M80.051G | Age-related osteoporosis with current pathological fracture, right femur, subsequent encounter for fracture with delayed healing |
| M80.051K | Age-related osteoporosis with current pathological fracture, right femur, subsequent encounter for fracture with nonunion |
| M80.051P | Age-related osteoporosis with current pathological fracture, right femur, subsequent encounter for fracture with malunion |
| M80.051S | Age-related osteoporosis with current pathological fracture, right femur, sequela |
| M80.052 | Age-related osteoporosis with current pathological fracture, left femur |
| M80.052A | Age-related osteoporosis with current pathological fracture, left femur, initial encounter for fracture |
| M80.052D | Age-related osteoporosis with current pathological fracture, left femur, subsequent encounter for fracture with routine healing |
| M80.052G | Age-related osteoporosis with current pathological fracture, left femur, subsequent encounter for fracture with delayed healing |
| M80.052K | Age-related osteoporosis with current pathological fracture, left femur, subsequent encounter for fracture with nonunion |
| M80.052P | Age-related osteoporosis with current pathological fracture, left femur, subsequent encounter for fracture with malunion |
| M80.052S | Age-related osteoporosis with current pathological fracture, left femur, sequela |
| M80.059 | Age-related osteoporosis with current pathological fracture, unspecified femur |
| M80.059A | Age-related osteoporosis with current pathological fracture, unspecified femur, initial encounter for fracture |
| M80.059D | Age-related osteoporosis with current pathological fracture, unspecified femur, subsequent encounter for fracture with routine healing |
| M80.059G | Age-related osteoporosis with current pathological fracture, unspecified femur, subsequent encounter for fracture with delayed healing |
| M80.059K | Age-related osteoporosis with current pathological fracture, unspecified femur, subsequent encounter for fracture with nonunion |
| M80.059P | Age-related osteoporosis with current pathological fracture, unspecified femur, subsequent encounter for fracture with malunion |
| M80.059S | Age-related osteoporosis with current pathological fracture, unspecified femur, sequela |
| M80.06 | Age-related osteoporosis with current pathological fracture, lower leg |
| M80.061 | Age-related osteoporosis with current pathological fracture, right lower leg |
| M80.061A | Age-related osteoporosis with current pathological fracture, right lower leg, initial encounter for fracture |
| M80.061D | Age-related osteoporosis with current pathological fracture, right lower leg, subsequent encounter for fracture with routine healing |
| M80.061G | Age-related osteoporosis with current pathological fracture, right lower leg, subsequent encounter for fracture with delayed healing |
| M80.061K | Age-related osteoporosis with current pathological fracture, right lower leg, subsequent encounter for fracture with nonunion |
| M80.061P | Age-related osteoporosis with current pathological fracture, right lower leg, subsequent encounter for fracture with malunion |
| M80.061S | Age-related osteoporosis with current pathological fracture, right lower leg, sequela |
| M80.062 | Age-related osteoporosis with current pathological fracture, left lower leg |
| M80.062A | Age-related osteoporosis with current pathological fracture, left lower leg, initial encounter for fracture |
| M80.062D | Age-related osteoporosis with current pathological fracture, left lower leg, subsequent encounter for fracture with routine healing |
| M80.062G | Age-related osteoporosis with current pathological fracture, left lower leg, subsequent encounter for fracture with delayed healing |
| M80.062K | Age-related osteoporosis with current pathological fracture, left lower leg, subsequent encounter for fracture with nonunion |
| M80.062P | Age-related osteoporosis with current pathological fracture, left lower leg, subsequent encounter for fracture with malunion |
| M80.062S | Age-related osteoporosis with current pathological fracture, left lower leg, sequela |
| M80.069 | Age-related osteoporosis with current pathological fracture, unspecified lower leg |
| M80.069A | Age-related osteoporosis with current pathological fracture, unspecified lower leg, initial encounter for fracture |
| M80.069D | Age-related osteoporosis with current pathological fracture, unspecified lower leg, subsequent encounter for fracture with routine healing |
| M80.069G | Age-related osteoporosis with current pathological fracture, unspecified lower leg, subsequent encounter for fracture with delayed healing |
| M80.069K | Age-related osteoporosis with current pathological fracture, unspecified lower leg, subsequent encounter for fracture with nonunion |
| M80.069P | Age-related osteoporosis with current pathological fracture, unspecified lower leg, subsequent encounter for fracture with malunion |
| M80.069S | Age-related osteoporosis with current pathological fracture, unspecified lower leg, sequela |
| M80.07 | Age-related osteoporosis with current pathological fracture, ankle and foot |
| M80.071 | Age-related osteoporosis with current pathological fracture, right ankle and foot |
| M80.071A | Age-related osteoporosis with current pathological fracture, right ankle and foot, initial encounter for fracture |
| M80.071D | Age-related osteoporosis with current pathological fracture, right ankle and foot, subsequent encounter for fracture with routine healing |
| M80.071G | Age-related osteoporosis with current pathological fracture, right ankle and foot, subsequent encounter for fracture with delayed healing |
| M80.071K | Age-related osteoporosis with current pathological fracture, right ankle and foot, subsequent encounter for fracture with nonunion |
| M80.071P | Age-related osteoporosis with current pathological fracture, right ankle and foot, subsequent encounter for fracture with malunion |
| M80.071S | Age-related osteoporosis with current pathological fracture, right ankle and foot, sequela |
| M80.072 | Age-related osteoporosis with current pathological fracture, left ankle and foot |
| M80.072A | Age-related osteoporosis with current pathological fracture, left ankle and foot, initial encounter for fracture |
| M80.072D | Age-related osteoporosis with current pathological fracture, left ankle and foot, subsequent encounter for fracture with routine healing |
| M80.072G | Age-related osteoporosis with current pathological fracture, left ankle and foot, subsequent encounter for fracture with delayed healing |
| M80.072K | Age-related osteoporosis with current pathological fracture, left ankle and foot, subsequent encounter for fracture with nonunion |
| M80.072P | Age-related osteoporosis with current pathological fracture, left ankle and foot, subsequent encounter for fracture with malunion |
| M80.072S | Age-related osteoporosis with current pathological fracture, left ankle and foot, sequela |
| M80.079 | Age-related osteoporosis with current pathological fracture, unspecified ankle and foot |
| M80.079A | Age-related osteoporosis with current pathological fracture, unspecified ankle and foot, initial encounter for fracture |
| M80.079D | Age-related osteoporosis with current pathological fracture, unspecified ankle and foot, subsequent encounter for fracture with routine healing |
| M80.079G | Age-related osteoporosis with current pathological fracture, unspecified ankle and foot, subsequent encounter for fracture with delayed healing |
| M80.079K | Age-related osteoporosis with current pathological fracture, unspecified ankle and foot, subsequent encounter for fracture with nonunion |
| M80.079P | Age-related osteoporosis with current pathological fracture, unspecified ankle and foot, subsequent encounter for fracture with malunion |
| M80.079S | Age-related osteoporosis with current pathological fracture, unspecified ankle and foot, sequela |
| M80.08 | Age-related osteoporosis with current pathological fracture, vertebra(e) |
| M80.08XA | Age-related osteoporosis with current pathological fracture, vertebra(e), initial encounter for fracture |
| M80.08XD | Age-related osteoporosis with current pathological fracture, vertebra(e), subsequent encounter for fracture with routine healing |
| M80.08XG | Age-related osteoporosis with current pathological fracture, vertebra(e), subsequent encounter for fracture with delayed healing |
| M80.08XK | Age-related osteoporosis with current pathological fracture, vertebra(e), subsequent encounter for fracture with nonunion |
| M80.08XP | Age-related osteoporosis with current pathological fracture, vertebra(e), subsequent encounter for fracture with malunion |
| M80.08XS | Age-related osteoporosis with current pathological fracture, vertebra(e), sequela |
| M80.8 | Other osteoporosis with current pathological fracture |
| M80.80 | Other osteoporosis with current pathological fracture, unspecified site |
| M80.80XA | Other osteoporosis with current pathological fracture, unspecified site, initial encounter for fracture |
| M80.80XD | Other osteoporosis with current pathological fracture, unspecified site, subsequent encounter for fracture with routine healing |
| M80.80XG | Other osteoporosis with current pathological fracture, unspecified site, subsequent encounter for fracture with delayed healing |
| M80.80XK | Other osteoporosis with current pathological fracture, unspecified site, subsequent encounter for fracture with nonunion |
| M80.80XP | Other osteoporosis with current pathological fracture, unspecified site, subsequent encounter for fracture with malunion |
| M80.80XS | Other osteoporosis with current pathological fracture, unspecified site, sequela |
| M80.81 | Other osteoporosis with pathological fracture, shoulder |
| M80.811 | Other osteoporosis with current pathological fracture, right shoulder |
| M80.811A | Other osteoporosis with current pathological fracture, right shoulder, initial encounter for fracture |
| M80.811D | Other osteoporosis with current pathological fracture, right shoulder, subsequent encounter for fracture with routine healing |
| M80.811G | Other osteoporosis with current pathological fracture, right shoulder, subsequent encounter for fracture with delayed healing |
| M80.811K | Other osteoporosis with current pathological fracture, right shoulder, subsequent encounter for fracture with nonunion |
| M80.811P | Other osteoporosis with current pathological fracture, right shoulder, subsequent encounter for fracture with malunion |
| M80.811S | Other osteoporosis with current pathological fracture, right shoulder, sequela |
| M80.812 | Other osteoporosis with current pathological fracture, left shoulder |
| M80.812A | Other osteoporosis with current pathological fracture, left shoulder, initial encounter for fracture |
| M80.812D | Other osteoporosis with current pathological fracture, left shoulder, subsequent encounter for fracture with routine healing |
| M80.812G | Other osteoporosis with current pathological fracture, left shoulder, subsequent encounter for fracture with delayed healing |
| M80.812K | Other osteoporosis with current pathological fracture, left shoulder, subsequent encounter for fracture with nonunion |
| M80.812P | Other osteoporosis with current pathological fracture, left shoulder, subsequent encounter for fracture with malunion |
| M80.812S | Other osteoporosis with current pathological fracture, left shoulder, sequela |
| M80.819 | Other osteoporosis with current pathological fracture, unspecified shoulder |
| M80.819A | Other osteoporosis with current pathological fracture, unspecified shoulder, initial encounter for fracture |
| M80.819D | Other osteoporosis with current pathological fracture, unspecified shoulder, subsequent encounter for fracture with routine healing |
| M80.819G | Other osteoporosis with current pathological fracture, unspecified shoulder, subsequent encounter for fracture with delayed healing |
| M80.819K | Other osteoporosis with current pathological fracture, unspecified shoulder, subsequent encounter for fracture with nonunion |
| M80.819P | Other osteoporosis with current pathological fracture, unspecified shoulder, subsequent encounter for fracture with malunion |
| M80.819S | Other osteoporosis with current pathological fracture, unspecified shoulder, sequela |
| M80.82 | Other osteoporosis with current pathological fracture, humerus |
| M80.821 | Other osteoporosis with current pathological fracture, right humerus |
| M80.821A | Other osteoporosis with current pathological fracture, right humerus, initial encounter for fracture |
| M80.821D | Other osteoporosis with current pathological fracture, right humerus, subsequent encounter for fracture with routine healing |
| M80.821G | Other osteoporosis with current pathological fracture, right humerus, subsequent encounter for fracture with delayed healing |
| M80.821K | Other osteoporosis with current pathological fracture, right humerus, subsequent encounter for fracture with nonunion |
| M80.821P | Other osteoporosis with current pathological fracture, right humerus, subsequent encounter for fracture with malunion |
| M80.821S | Other osteoporosis with current pathological fracture, right humerus, sequela |
| M80.822 | Other osteoporosis with current pathological fracture, left humerus |
| M80.822A | Other osteoporosis with current pathological fracture, left humerus, initial encounter for fracture |
| M80.822D | Other osteoporosis with current pathological fracture, left humerus, subsequent encounter for fracture with routine healing |
| M80.822G | Other osteoporosis with current pathological fracture, left humerus, subsequent encounter for fracture with delayed healing |
| M80.822K | Other osteoporosis with current pathological fracture, left humerus, subsequent encounter for fracture with nonunion |
| M80.822P | Other osteoporosis with current pathological fracture, left humerus, subsequent encounter for fracture with malunion |
| M80.822S | Other osteoporosis with current pathological fracture, left humerus, sequela |
| M80.829 | Other osteoporosis with current pathological fracture, unspecified humerus |
| M80.829A | Other osteoporosis with current pathological fracture, unspecified humerus, initial encounter for fracture |
| M80.829D | Other osteoporosis with current pathological fracture, unspecified humerus, subsequent encounter for fracture with routine healing |
| M80.829G | Other osteoporosis with current pathological fracture, unspecified humerus, subsequent encounter for fracture with delayed healing |
| M80.829K | Other osteoporosis with current pathological fracture, unspecified humerus, subsequent encounter for fracture with nonunion |
| M80.829P | Other osteoporosis with current pathological fracture, unspecified humerus, subsequent encounter for fracture with malunion |
| M80.829S | Other osteoporosis with current pathological fracture, unspecified humerus, sequela |
| M80.83 | Other osteoporosis with current pathological fracture, forearm |
| M80.831 | Other osteoporosis with current pathological fracture, right forearm |
| M80.831A | Other osteoporosis with current pathological fracture, right forearm, initial encounter for fracture |
| M80.831D | Other osteoporosis with current pathological fracture, right forearm, subsequent encounter for fracture with routine healing |
| M80.831G | Other osteoporosis with current pathological fracture, right forearm, subsequent encounter for fracture with delayed healing |
| M80.831K | Other osteoporosis with current pathological fracture, right forearm, subsequent encounter for fracture with nonunion |
| M80.831P | Other osteoporosis with current pathological fracture, right forearm, subsequent encounter for fracture with malunion |
| M80.831S | Other osteoporosis with current pathological fracture, right forearm, sequela |
| M80.832 | Other osteoporosis with current pathological fracture, left forearm |
| M80.832A | Other osteoporosis with current pathological fracture, left forearm, initial encounter for fracture |
| M80.832D | Other osteoporosis with current pathological fracture, left forearm, subsequent encounter for fracture with routine healing |
| M80.832G | Other osteoporosis with current pathological fracture, left forearm, subsequent encounter for fracture with delayed healing |
| M80.832K | Other osteoporosis with current pathological fracture, left forearm, subsequent encounter for fracture with nonunion |
| M80.832P | Other osteoporosis with current pathological fracture, left forearm, subsequent encounter for fracture with malunion |
| M80.832S | Other osteoporosis with current pathological fracture, left forearm, sequela |
| M80.839 | Other osteoporosis with current pathological fracture, unspecified forearm |
| M80.839A | Other osteoporosis with current pathological fracture, unspecified forearm, initial encounter for fracture |
| M80.839D | Other osteoporosis with current pathological fracture, unspecified forearm, subsequent encounter for fracture with routine healing |
| M80.839G | Other osteoporosis with current pathological fracture, unspecified forearm, subsequent encounter for fracture with delayed healing |
| M80.839K | Other osteoporosis with current pathological fracture, unspecified forearm, subsequent encounter for fracture with nonunion |
| M80.839P | Other osteoporosis with current pathological fracture, unspecified forearm, subsequent encounter for fracture with malunion |
| M80.839S | Other osteoporosis with current pathological fracture, unspecified forearm, sequela |
| M80.84 | Other osteoporosis with current pathological fracture, hand |
| M80.841 | Other osteoporosis with current pathological fracture, right hand |
| M80.841A | Other osteoporosis with current pathological fracture, right hand, initial encounter for fracture |
| M80.841D | Other osteoporosis with current pathological fracture, right hand, subsequent encounter for fracture with routine healing |
| M80.841G | Other osteoporosis with current pathological fracture, right hand, subsequent encounter for fracture with delayed healing |
| M80.841K | Other osteoporosis with current pathological fracture, right hand, subsequent encounter for fracture with nonunion |
| M80.841P | Other osteoporosis with current pathological fracture, right hand, subsequent encounter for fracture with malunion |
| M80.841S | Other osteoporosis with current pathological fracture, right hand, sequela |
| M80.842 | Other osteoporosis with current pathological fracture, left hand |
| M80.842A | Other osteoporosis with current pathological fracture, left hand, initial encounter for fracture |
| M80.842D | Other osteoporosis with current pathological fracture, left hand, subsequent encounter for fracture with routine healing |
| M80.842G | Other osteoporosis with current pathological fracture, left hand, subsequent encounter for fracture with delayed healing |
| M80.842K | Other osteoporosis with current pathological fracture, left hand, subsequent encounter for fracture with nonunion |
| M80.842P | Other osteoporosis with current pathological fracture, left hand, subsequent encounter for fracture with malunion |
| M80.842S | Other osteoporosis with current pathological fracture, left hand, sequela |
| M80.849 | Other osteoporosis with current pathological fracture, unspecified hand |
| M80.849A | Other osteoporosis with current pathological fracture, unspecified hand, initial encounter for fracture |
| M80.849D | Other osteoporosis with current pathological fracture, unspecified hand, subsequent encounter for fracture with routine healing |
| M80.849G | Other osteoporosis with current pathological fracture, unspecified hand, subsequent encounter for fracture with delayed healing |
| M80.849K | Other osteoporosis with current pathological fracture, unspecified hand, subsequent encounter for fracture with nonunion |
| M80.849P | Other osteoporosis with current pathological fracture, unspecified hand, subsequent encounter for fracture with malunion |
| M80.849S | Other osteoporosis with current pathological fracture, unspecified hand, sequela |
| M80.85 | Other osteoporosis with current pathological fracture, femur |
| M80.851 | Other osteoporosis with current pathological fracture, right femur |
| M80.851A | Other osteoporosis with current pathological fracture, right femur, initial encounter for fracture |
| M80.851D | Other osteoporosis with current pathological fracture, right femur, subsequent encounter for fracture with routine healing |
| M80.851G | Other osteoporosis with current pathological fracture, right femur, subsequent encounter for fracture with delayed healing |
| M80.851K | Other osteoporosis with current pathological fracture, right femur, subsequent encounter for fracture with nonunion |
| M80.851P | Other osteoporosis with current pathological fracture, right femur, subsequent encounter for fracture with malunion |
| M80.851S | Other osteoporosis with current pathological fracture, right femur, sequela |
| M80.852 | Other osteoporosis with current pathological fracture, left femur |
| M80.852A | Other osteoporosis with current pathological fracture, left femur, initial encounter for fracture |
| M80.852D | Other osteoporosis with current pathological fracture, left femur, subsequent encounter for fracture with routine healing |
| M80.852G | Other osteoporosis with current pathological fracture, left femur, subsequent encounter for fracture with delayed healing |
| M80.852K | Other osteoporosis with current pathological fracture, left femur, subsequent encounter for fracture with nonunion |
| M80.852P | Other osteoporosis with current pathological fracture, left femur, subsequent encounter for fracture with malunion |
| M80.852S | Other osteoporosis with current pathological fracture, left femur, sequela |
| M80.859 | Other osteoporosis with current pathological fracture, unspecified femur |
| M80.859A | Other osteoporosis with current pathological fracture, unspecified femur, initial encounter for fracture |
| M80.859D | Other osteoporosis with current pathological fracture, unspecified femur, subsequent encounter for fracture with routine healing |
| M80.859G | Other osteoporosis with current pathological fracture, unspecified femur, subsequent encounter for fracture with delayed healing |
| M80.859K | Other osteoporosis with current pathological fracture, unspecified femur, subsequent encounter for fracture with nonunion |
| M80.859P | Other osteoporosis with current pathological fracture, unspecified femur, subsequent encounter for fracture with malunion |
| M80.859S | Other osteoporosis with current pathological fracture, unspecified femur, sequela |
| M80.86 | Other osteoporosis with current pathological fracture, lower leg |
| M80.861 | Other osteoporosis with current pathological fracture, right lower leg |
| M80.861A | Other osteoporosis with current pathological fracture, right lower leg, initial encounter for fracture |
| M80.861D | Other osteoporosis with current pathological fracture, right lower leg, subsequent encounter for fracture with routine healing |
| M80.861G | Other osteoporosis with current pathological fracture, right lower leg, subsequent encounter for fracture with delayed healing |
| M80.861K | Other osteoporosis with current pathological fracture, right lower leg, subsequent encounter for fracture with nonunion |
| M80.861P | Other osteoporosis with current pathological fracture, right lower leg, subsequent encounter for fracture with malunion |
| M80.861S | Other osteoporosis with current pathological fracture, right lower leg, sequela |
| M80.862 | Other osteoporosis with current pathological fracture, left lower leg |
| M80.862A | Other osteoporosis with current pathological fracture, left lower leg, initial encounter for fracture |
| M80.862D | Other osteoporosis with current pathological fracture, left lower leg, subsequent encounter for fracture with routine healing |
| M80.862G | Other osteoporosis with current pathological fracture, left lower leg, subsequent encounter for fracture with delayed healing |
| M80.862K | Other osteoporosis with current pathological fracture, left lower leg, subsequent encounter for fracture with nonunion |
| M80.862P | Other osteoporosis with current pathological fracture, left lower leg, subsequent encounter for fracture with malunion |
| M80.862S | Other osteoporosis with current pathological fracture, left lower leg, sequela |
| M80.869 | Other osteoporosis with current pathological fracture, unspecified lower leg |
| M80.869A | Other osteoporosis with current pathological fracture, unspecified lower leg, initial encounter for fracture |
| M80.869D | Other osteoporosis with current pathological fracture, unspecified lower leg, subsequent encounter for fracture with routine healing |
| M80.869G | Other osteoporosis with current pathological fracture, unspecified lower leg, subsequent encounter for fracture with delayed healing |
| M80.869K | Other osteoporosis with current pathological fracture, unspecified lower leg, subsequent encounter for fracture with nonunion |
| M80.869P | Other osteoporosis with current pathological fracture, unspecified lower leg, subsequent encounter for fracture with malunion |
| M80.869S | Other osteoporosis with current pathological fracture, unspecified lower leg, sequela |
| M80.87 | Other osteoporosis with current pathological fracture, ankle and foot |
| M80.871 | Other osteoporosis with current pathological fracture, right ankle and foot |
| M80.871A | Other osteoporosis with current pathological fracture, right ankle and foot, initial encounter for fracture |
| M80.871D | Other osteoporosis with current pathological fracture, right ankle and foot, subsequent encounter for fracture with routine healing |
| M80.871G | Other osteoporosis with current pathological fracture, right ankle and foot, subsequent encounter for fracture with delayed healing |
| M80.871K | Other osteoporosis with current pathological fracture, right ankle and foot, subsequent encounter for fracture with nonunion |
| M80.871P | Other osteoporosis with current pathological fracture, right ankle and foot, subsequent encounter for fracture with malunion |
| M80.871S | Other osteoporosis with current pathological fracture, right ankle and foot, sequela |
| M80.872 | Other osteoporosis with current pathological fracture, left ankle and foot |
| M80.872A | Other osteoporosis with current pathological fracture, left ankle and foot, initial encounter for fracture |
| M80.872D | Other osteoporosis with current pathological fracture, left ankle and foot, subsequent encounter for fracture with routine healing |
| M80.872G | Other osteoporosis with current pathological fracture, left ankle and foot, subsequent encounter for fracture with delayed healing |
| M80.872K | Other osteoporosis with current pathological fracture, left ankle and foot, subsequent encounter for fracture with nonunion |
| M80.872P | Other osteoporosis with current pathological fracture, left ankle and foot, subsequent encounter for fracture with malunion |
| M80.872S | Other osteoporosis with current pathological fracture, left ankle and foot, sequela |
| M80.879 | Other osteoporosis with current pathological fracture, unspecified ankle and foot |
| M80.879A | Other osteoporosis with current pathological fracture, unspecified ankle and foot, initial encounter for fracture |
| M80.879D | Other osteoporosis with current pathological fracture, unspecified ankle and foot, subsequent encounter for fracture with routine healing |
| M80.879G | Other osteoporosis with current pathological fracture, unspecified ankle and foot, subsequent encounter for fracture with delayed healing |
| M80.879K | Other osteoporosis with current pathological fracture, unspecified ankle and foot, subsequent encounter for fracture with nonunion |
| M80.879P | Other osteoporosis with current pathological fracture, unspecified ankle and foot, subsequent encounter for fracture with malunion |
| M80.879S | Other osteoporosis with current pathological fracture, unspecified ankle and foot, sequela |
| M80.88 | Other osteoporosis with current pathological fracture, vertebra(e) |
| M80.88XA | Other osteoporosis with current pathological fracture, vertebra(e), initial encounter for fracture |
| M80.88XD | Other osteoporosis with current pathological fracture, vertebra(e), subsequent encounter for fracture with routine healing |
| M80.88XG | Other osteoporosis with current pathological fracture, vertebra(e), subsequent encounter for fracture with delayed healing |
| M80.88XK | Other osteoporosis with current pathological fracture, vertebra(e), subsequent encounter for fracture with nonunion |
| M80.88XP | Other osteoporosis with current pathological fracture, vertebra(e), subsequent encounter for fracture with malunion |
| M80.88XS | Other osteoporosis with current pathological fracture, vertebra(e), sequela |
| M81 | Osteoporosis without current pathological fracture |
| M81.0 | Age-related osteoporosis without current pathological fracture |
| M81.6 | Localized osteoporosis [Lequesne] |
| M81.8 | Other osteoporosis without current pathological fracture |
| M84 | Disorder of continuity of bone |
| M84.3 | Stress fracture |
| M84.30 | Stress fracture, unspecified site |
| M84.30XA | Stress fracture, unspecified site, initial encounter for fracture |
| M84.30XD | Stress fracture, unspecified site, subsequent encounter for fracture with routine healing |
| M84.30XG | Stress fracture, unspecified site, subsequent encounter for fracture with delayed healing |
| M84.30XK | Stress fracture, unspecified site, subsequent encounter for fracture with nonunion |
| M84.30XP | Stress fracture, unspecified site, subsequent encounter for fracture with malunion |
| M84.30XS | Stress fracture, unspecified site, sequela |
| M84.31 | Stress fracture, shoulder |
| M84.311 | Stress fracture, right shoulder |
| M84.311A | Stress fracture, right shoulder, initial encounter for fracture |
| M84.311D | Stress fracture, right shoulder, subsequent encounter for fracture with routine healing |
| M84.311G | Stress fracture, right shoulder, subsequent encounter for fracture with delayed healing |
| M84.311K | Stress fracture, right shoulder, subsequent encounter for fracture with nonunion |
| M84.311P | Stress fracture, right shoulder, subsequent encounter for fracture with malunion |
| M84.311S | Stress fracture, right shoulder, sequela |
| M84.312 | Stress fracture, left shoulder |
| M84.312A | Stress fracture, left shoulder, initial encounter for fracture |
| M84.312D | Stress fracture, left shoulder, subsequent encounter for fracture with routine healing |
| M84.312G | Stress fracture, left shoulder, subsequent encounter for fracture with delayed healing |
| M84.312K | Stress fracture, left shoulder, subsequent encounter for fracture with nonunion |
| M84.312P | Stress fracture, left shoulder, subsequent encounter for fracture with malunion |
| M84.312S | Stress fracture, left shoulder, sequela |
| M84.319 | Stress fracture, unspecified shoulder |
| M84.319A | Stress fracture, unspecified shoulder, initial encounter for fracture |
| M84.319D | Stress fracture, unspecified shoulder, subsequent encounter for fracture with routine healing |
| M84.319G | Stress fracture, unspecified shoulder, subsequent encounter for fracture with delayed healing |
| M84.319K | Stress fracture, unspecified shoulder, subsequent encounter for fracture with nonunion |
| M84.319P | Stress fracture, unspecified shoulder, subsequent encounter for fracture with malunion |
| M84.319S | Stress fracture, unspecified shoulder, sequela |
| M84.32 | Stress fracture, humerus |
| M84.321 | Stress fracture, right humerus |
| M84.321A | Stress fracture, right humerus, initial encounter for fracture |
| M84.321D | Stress fracture, right humerus, subsequent encounter for fracture with routine healing |
| M84.321G | Stress fracture, right humerus, subsequent encounter for fracture with delayed healing |
| M84.321K | Stress fracture, right humerus, subsequent encounter for fracture with nonunion |
| M84.321P | Stress fracture, right humerus, subsequent encounter for fracture with malunion |
| M84.321S | Stress fracture, right humerus, sequela |
| M84.322 | Stress fracture, left humerus |
| M84.322A | Stress fracture, left humerus, initial encounter for fracture |
| M84.322D | Stress fracture, left humerus, subsequent encounter for fracture with routine healing |
| M84.322G | Stress fracture, left humerus, subsequent encounter for fracture with delayed healing |
| M84.322K | Stress fracture, left humerus, subsequent encounter for fracture with nonunion |
| M84.322P | Stress fracture, left humerus, subsequent encounter for fracture with malunion |
| M84.322S | Stress fracture, left humerus, sequela |
| M84.329 | Stress fracture, unspecified humerus |
| M84.329A | Stress fracture, unspecified humerus, initial encounter for fracture |
| M84.329D | Stress fracture, unspecified humerus, subsequent encounter for fracture with routine healing |
| M84.329G | Stress fracture, unspecified humerus, subsequent encounter for fracture with delayed healing |
| M84.329K | Stress fracture, unspecified humerus, subsequent encounter for fracture with nonunion |
| M84.329P | Stress fracture, unspecified humerus, subsequent encounter for fracture with malunion |
| M84.329S | Stress fracture, unspecified humerus, sequela |
| M84.33 | Stress fracture, ulna and radius |
| M84.331 | Stress fracture, right ulna |
| M84.331A | Stress fracture, right ulna, initial encounter for fracture |
| M84.331D | Stress fracture, right ulna, subsequent encounter for fracture with routine healing |
| M84.331G | Stress fracture, right ulna, subsequent encounter for fracture with delayed healing |
| M84.331K | Stress fracture, right ulna, subsequent encounter for fracture with nonunion |
| M84.331P | Stress fracture, right ulna, subsequent encounter for fracture with malunion |
| M84.331S | Stress fracture, right ulna, sequela |
| M84.332 | Stress fracture, left ulna |
| M84.332A | Stress fracture, left ulna, initial encounter for fracture |
| M84.332D | Stress fracture, left ulna, subsequent encounter for fracture with routine healing |
| M84.332G | Stress fracture, left ulna, subsequent encounter for fracture with delayed healing |
| M84.332K | Stress fracture, left ulna, subsequent encounter for fracture with nonunion |
| M84.332P | Stress fracture, left ulna, subsequent encounter for fracture with malunion |
| M84.332S | Stress fracture, left ulna, sequela |
| M84.333 | Stress fracture, right radius |
| M84.333A | Stress fracture, right radius, initial encounter for fracture |
| M84.333D | Stress fracture, right radius, subsequent encounter for fracture with routine healing |
| M84.333G | Stress fracture, right radius, subsequent encounter for fracture with delayed healing |
| M84.333K | Stress fracture, right radius, subsequent encounter for fracture with nonunion |
| M84.333P | Stress fracture, right radius, subsequent encounter for fracture with malunion |
| M84.333S | Stress fracture, right radius, sequela |
| M84.334 | Stress fracture, left radius |
| M84.334A | Stress fracture, left radius, initial encounter for fracture |
| M84.334D | Stress fracture, left radius, subsequent encounter for fracture with routine healing |
| M84.334G | Stress fracture, left radius, subsequent encounter for fracture with delayed healing |
| M84.334K | Stress fracture, left radius, subsequent encounter for fracture with nonunion |
| M84.334P | Stress fracture, left radius, subsequent encounter for fracture with malunion |
| M84.334S | Stress fracture, left radius, sequela |
| M84.339 | Stress fracture, unspecified ulna and radius |
| M84.339A | Stress fracture, unspecified ulna and radius, initial encounter for fracture |
| M84.339D | Stress fracture, unspecified ulna and radius, subsequent encounter for fracture with routine healing |
| M84.339G | Stress fracture, unspecified ulna and radius, subsequent encounter for fracture with delayed healing |
| M84.339K | Stress fracture, unspecified ulna and radius, subsequent encounter for fracture with nonunion |
| M84.339P | Stress fracture, unspecified ulna and radius, subsequent encounter for fracture with malunion |
| M84.339S | Stress fracture, unspecified ulna and radius, sequela |
| M84.34 | Stress fracture, hand and fingers |
| M84.341 | Stress fracture, right hand |
| M84.341A | Stress fracture, right hand, initial encounter for fracture |
| M84.341D | Stress fracture, right hand, subsequent encounter for fracture with routine healing |
| M84.341G | Stress fracture, right hand, subsequent encounter for fracture with delayed healing |
| M84.341K | Stress fracture, right hand, subsequent encounter for fracture with nonunion |
| M84.341P | Stress fracture, right hand, subsequent encounter for fracture with malunion |
| M84.341S | Stress fracture, right hand, sequela |
| M84.342 | Stress fracture, left hand |
| M84.342A | Stress fracture, left hand, initial encounter for fracture |
| M84.342D | Stress fracture, left hand, subsequent encounter for fracture with routine healing |
| M84.342G | Stress fracture, left hand, subsequent encounter for fracture with delayed healing |
| M84.342K | Stress fracture, left hand, subsequent encounter for fracture with nonunion |
| M84.342P | Stress fracture, left hand, subsequent encounter for fracture with malunion |
| M84.342S | Stress fracture, left hand, sequela |
| M84.343 | Stress fracture, unspecified hand |
| M84.343A | Stress fracture, unspecified hand, initial encounter for fracture |
| M84.343D | Stress fracture, unspecified hand, subsequent encounter for fracture with routine healing |
| M84.343G | Stress fracture, unspecified hand, subsequent encounter for fracture with delayed healing |
| M84.343K | Stress fracture, unspecified hand, subsequent encounter for fracture with nonunion |
| M84.343P | Stress fracture, unspecified hand, subsequent encounter for fracture with malunion |
| M84.343S | Stress fracture, unspecified hand, sequela |
| M84.344 | Stress fracture, right finger(s) |
| M84.344A | Stress fracture, right finger(s), initial encounter for fracture |
| M84.344D | Stress fracture, right finger(s), subsequent encounter for fracture with routine healing |
| M84.344G | Stress fracture, right finger(s), subsequent encounter for fracture with delayed healing |
| M84.344K | Stress fracture, right finger(s), subsequent encounter for fracture with nonunion |
| M84.344P | Stress fracture, right finger(s), subsequent encounter for fracture with malunion |
| M84.344S | Stress fracture, right finger(s), sequela |
| M84.345 | Stress fracture, left finger(s) |
| M84.345A | Stress fracture, left finger(s), initial encounter for fracture |
| M84.345D | Stress fracture, left finger(s), subsequent encounter for fracture with routine healing |
| M84.345G | Stress fracture, left finger(s), subsequent encounter for fracture with delayed healing |
| M84.345K | Stress fracture, left finger(s), subsequent encounter for fracture with nonunion |
| M84.345P | Stress fracture, left finger(s), subsequent encounter for fracture with malunion |
| M84.345S | Stress fracture, left finger(s), sequela |
| M84.346 | Stress fracture, unspecified finger(s) |
| M84.346A | Stress fracture, unspecified finger(s), initial encounter for fracture |
| M84.346D | Stress fracture, unspecified finger(s), subsequent encounter for fracture with routine healing |
| M84.346G | Stress fracture, unspecified finger(s), subsequent encounter for fracture with delayed healing |
| M84.346K | Stress fracture, unspecified finger(s), subsequent encounter for fracture with nonunion |
| M84.346P | Stress fracture, unspecified finger(s), subsequent encounter for fracture with malunion |
| M84.346S | Stress fracture, unspecified finger(s), sequela |
| M84.35 | Stress fracture, pelvis and femur |
| M84.350 | Stress fracture, pelvis |
| M84.350A | Stress fracture, pelvis, initial encounter for fracture |
| M84.350D | Stress fracture, pelvis, subsequent encounter for fracture with routine healing |
| M84.350G | Stress fracture, pelvis, subsequent encounter for fracture with delayed healing |
| M84.350K | Stress fracture, pelvis, subsequent encounter for fracture with nonunion |
| M84.350P | Stress fracture, pelvis, subsequent encounter for fracture with malunion |
| M84.350S | Stress fracture, pelvis, sequela |
| M84.351 | Stress fracture, right femur |
| M84.351A | Stress fracture, right femur, initial encounter for fracture |
| M84.351D | Stress fracture, right femur, subsequent encounter for fracture with routine healing |
| M84.351G | Stress fracture, right femur, subsequent encounter for fracture with delayed healing |
| M84.351K | Stress fracture, right femur, subsequent encounter for fracture with nonunion |
| M84.351P | Stress fracture, right femur, subsequent encounter for fracture with malunion |
| M84.351S | Stress fracture, right femur, sequela |
| M84.352 | Stress fracture, left femur |
| M84.352A | Stress fracture, left femur, initial encounter for fracture |
| M84.352D | Stress fracture, left femur, subsequent encounter for fracture with routine healing |
| M84.352G | Stress fracture, left femur, subsequent encounter for fracture with delayed healing |
| M84.352K | Stress fracture, left femur, subsequent encounter for fracture with nonunion |
| M84.352P | Stress fracture, left femur, subsequent encounter for fracture with malunion |
| M84.352S | Stress fracture, left femur, sequela |
| M84.353 | Stress fracture, unspecified femur |
| M84.353A | Stress fracture, unspecified femur, initial encounter for fracture |
| M84.353D | Stress fracture, unspecified femur, subsequent encounter for fracture with routine healing |
| M84.353G | Stress fracture, unspecified femur, subsequent encounter for fracture with delayed healing |
| M84.353K | Stress fracture, unspecified femur, subsequent encounter for fracture with nonunion |
| M84.353P | Stress fracture, unspecified femur, subsequent encounter for fracture with malunion |
| M84.353S | Stress fracture, unspecified femur, sequela |
| M84.359 | Stress fracture, hip, unspecified |
| M84.359A | Stress fracture, hip, unspecified, initial encounter for fracture |
| M84.359D | Stress fracture, hip, unspecified, subsequent encounter for fracture with routine healing |
| M84.359G | Stress fracture, hip, unspecified, subsequent encounter for fracture with delayed healing |
| M84.359K | Stress fracture, hip, unspecified, subsequent encounter for fracture with nonunion |
| M84.359P | Stress fracture, hip, unspecified, subsequent encounter for fracture with malunion |
| M84.359S | Stress fracture, hip, unspecified, sequela |
| M84.36 | Stress fracture, tibia and fibula |
| M84.361 | Stress fracture, right tibia |
| M84.361A | Stress fracture, right tibia, initial encounter for fracture |
| M84.361D | Stress fracture, right tibia, subsequent encounter for fracture with routine healing |
| M84.361G | Stress fracture, right tibia, subsequent encounter for fracture with delayed healing |
| M84.361K | Stress fracture, right tibia, subsequent encounter for fracture with nonunion |
| M84.361P | Stress fracture, right tibia, subsequent encounter for fracture with malunion |
| M84.361S | Stress fracture, right tibia, sequela |
| M84.362 | Stress fracture, left tibia |
| M84.362A | Stress fracture, left tibia, initial encounter for fracture |
| M84.362D | Stress fracture, left tibia, subsequent encounter for fracture with routine healing |
| M84.362G | Stress fracture, left tibia, subsequent encounter for fracture with delayed healing |
| M84.362K | Stress fracture, left tibia, subsequent encounter for fracture with nonunion |
| M84.362P | Stress fracture, left tibia, subsequent encounter for fracture with malunion |
| M84.362S | Stress fracture, left tibia, sequela |
| M84.363 | Stress fracture, right fibula |
| M84.363A | Stress fracture, right fibula, initial encounter for fracture |
| M84.363D | Stress fracture, right fibula, subsequent encounter for fracture with routine healing |
| M84.363G | Stress fracture, right fibula, subsequent encounter for fracture with delayed healing |
| M84.363K | Stress fracture, right fibula, subsequent encounter for fracture with nonunion |
| M84.363P | Stress fracture, right fibula, subsequent encounter for fracture with malunion |
| M84.363S | Stress fracture, right fibula, sequela |
| M84.364 | Stress fracture, left fibula |
| M84.364A | Stress fracture, left fibula, initial encounter for fracture |
| M84.364D | Stress fracture, left fibula, subsequent encounter for fracture with routine healing |
| M84.364G | Stress fracture, left fibula, subsequent encounter for fracture with delayed healing |
| M84.364K | Stress fracture, left fibula, subsequent encounter for fracture with nonunion |
| M84.364P | Stress fracture, left fibula, subsequent encounter for fracture with malunion |
| M84.364S | Stress fracture, left fibula, sequela |
| M84.369 | Stress fracture, unspecified tibia and fibula |
| M84.369A | Stress fracture, unspecified tibia and fibula, initial encounter for fracture |
| M84.369D | Stress fracture, unspecified tibia and fibula, subsequent encounter for fracture with routine healing |
| M84.369G | Stress fracture, unspecified tibia and fibula, subsequent encounter for fracture with delayed healing |
| M84.369K | Stress fracture, unspecified tibia and fibula, subsequent encounter for fracture with nonunion |
| M84.369P | Stress fracture, unspecified tibia and fibula, subsequent encounter for fracture with malunion |
| M84.369S | Stress fracture, unspecified tibia and fibula, sequela |
| M84.37 | Stress fracture, ankle, foot and toes |
| M84.371 | Stress fracture, right ankle |
| M84.371A | Stress fracture, right ankle, initial encounter for fracture |
| M84.371D | Stress fracture, right ankle, subsequent encounter for fracture with routine healing |
| M84.371G | Stress fracture, right ankle, subsequent encounter for fracture with delayed healing |
| M84.371K | Stress fracture, right ankle, subsequent encounter for fracture with nonunion |
| M84.371P | Stress fracture, right ankle, subsequent encounter for fracture with malunion |
| M84.371S | Stress fracture, right ankle, sequela |
| M84.372 | Stress fracture, left ankle |
| M84.372A | Stress fracture, left ankle, initial encounter for fracture |
| M84.372D | Stress fracture, left ankle, subsequent encounter for fracture with routine healing |
| M84.372G | Stress fracture, left ankle, subsequent encounter for fracture with delayed healing |
| M84.372K | Stress fracture, left ankle, subsequent encounter for fracture with nonunion |
| M84.372P | Stress fracture, left ankle, subsequent encounter for fracture with malunion |
| M84.372S | Stress fracture, left ankle, sequela |
| M84.373 | Stress fracture, unspecified ankle |
| M84.373A | Stress fracture, unspecified ankle, initial encounter for fracture |
| M84.373D | Stress fracture, unspecified ankle, subsequent encounter for fracture with routine healing |
| M84.373G | Stress fracture, unspecified ankle, subsequent encounter for fracture with delayed healing |
| M84.373K | Stress fracture, unspecified ankle, subsequent encounter for fracture with nonunion |
| M84.373P | Stress fracture, unspecified ankle, subsequent encounter for fracture with malunion |
| M84.373S | Stress fracture, unspecified ankle, sequela |
| M84.374 | Stress fracture, right foot |
| M84.374A | Stress fracture, right foot, initial encounter for fracture |
| M84.374D | Stress fracture, right foot, subsequent encounter for fracture with routine healing |
| M84.374G | Stress fracture, right foot, subsequent encounter for fracture with delayed healing |
| M84.374K | Stress fracture, right foot, subsequent encounter for fracture with nonunion |
| M84.374P | Stress fracture, right foot, subsequent encounter for fracture with malunion |
| M84.374S | Stress fracture, right foot, sequela |
| M84.375 | Stress fracture, left foot |
| M84.375A | Stress fracture, left foot, initial encounter for fracture |
| M84.375D | Stress fracture, left foot, subsequent encounter for fracture with routine healing |
| M84.375G | Stress fracture, left foot, subsequent encounter for fracture with delayed healing |
| M84.375K | Stress fracture, left foot, subsequent encounter for fracture with nonunion |
| M84.375P | Stress fracture, left foot, subsequent encounter for fracture with malunion |
| M84.375S | Stress fracture, left foot, sequela |
| M84.376 | Stress fracture, unspecified foot |
| M84.376A | Stress fracture, unspecified foot, initial encounter for fracture |
| M84.376D | Stress fracture, unspecified foot, subsequent encounter for fracture with routine healing |
| M84.376G | Stress fracture, unspecified foot, subsequent encounter for fracture with delayed healing |
| M84.376K | Stress fracture, unspecified foot, subsequent encounter for fracture with nonunion |
| M84.376P | Stress fracture, unspecified foot, subsequent encounter for fracture with malunion |
| M84.376S | Stress fracture, unspecified foot, sequela |
| M84.377 | Stress fracture, right toe(s) |
| M84.377A | Stress fracture, right toe(s), initial encounter for fracture |
| M84.377D | Stress fracture, right toe(s), subsequent encounter for fracture with routine healing |
| M84.377G | Stress fracture, right toe(s), subsequent encounter for fracture with delayed healing |
| M84.377K | Stress fracture, right toe(s), subsequent encounter for fracture with nonunion |
| M84.377P | Stress fracture, right toe(s), subsequent encounter for fracture with malunion |
| M84.377S | Stress fracture, right toe(s), sequela |
| M84.378 | Stress fracture, left toe(s) |
| M84.378A | Stress fracture, left toe(s), initial encounter for fracture |
| M84.378D | Stress fracture, left toe(s), subsequent encounter for fracture with routine healing |
| M84.378G | Stress fracture, left toe(s), subsequent encounter for fracture with delayed healing |
| M84.378K | Stress fracture, left toe(s), subsequent encounter for fracture with nonunion |
| M84.378P | Stress fracture, left toe(s), subsequent encounter for fracture with malunion |
| M84.378S | Stress fracture, left toe(s), sequela |
| M84.379 | Stress fracture, unspecified toe(s) |
| M84.379A | Stress fracture, unspecified toe(s), initial encounter for fracture |
| M84.379D | Stress fracture, unspecified toe(s), subsequent encounter for fracture with routine healing |
| M84.379G | Stress fracture, unspecified toe(s), subsequent encounter for fracture with delayed healing |
| M84.379K | Stress fracture, unspecified toe(s), subsequent encounter for fracture with nonunion |
| M84.379P | Stress fracture, unspecified toe(s), subsequent encounter for fracture with malunion |
| M84.379S | Stress fracture, unspecified toe(s), sequela |
| M84.38 | Stress fracture, other site |
| M84.38XA | Stress fracture, other site, initial encounter for fracture |
| M84.38XD | Stress fracture, other site, subsequent encounter for fracture with routine healing |
| M84.38XG | Stress fracture, other site, subsequent encounter for fracture with delayed healing |
| M84.38XK | Stress fracture, other site, subsequent encounter for fracture with nonunion |
| M84.38XP | Stress fracture, other site, subsequent encounter for fracture with malunion |
| M84.38XS | Stress fracture, other site, sequela |
| M84.4 | Pathological fracture, not elsewhere classified |
| M84.40 | Pathological fracture, unspecified site |
| M84.40XA | Pathological fracture, unspecified site, initial encounter for fracture |
| M84.40XD | Pathological fracture, unspecified site, subsequent encounter for fracture with routine healing |
| M84.40XG | Pathological fracture, unspecified site, subsequent encounter for fracture with delayed healing |
| M84.40XK | Pathological fracture, unspecified site, subsequent encounter for fracture with nonunion |
| M84.40XP | Pathological fracture, unspecified site, subsequent encounter for fracture with malunion |
| M84.40XS | Pathological fracture, unspecified site, sequela |
| M84.41 | Pathological fracture, shoulder |
| M84.411 | Pathological fracture, right shoulder |
| M84.411A | Pathological fracture, right shoulder, initial encounter for fracture |
| M84.411D | Pathological fracture, right shoulder, subsequent encounter for fracture with routine healing |
| M84.411G | Pathological fracture, right shoulder, subsequent encounter for fracture with delayed healing |
| M84.411K | Pathological fracture, right shoulder, subsequent encounter for fracture with nonunion |
| M84.411P | Pathological fracture, right shoulder, subsequent encounter for fracture with malunion |
| M84.411S | Pathological fracture, right shoulder, sequela |
| M84.412 | Pathological fracture, left shoulder |
| M84.412A | Pathological fracture, left shoulder, initial encounter for fracture |
| M84.412D | Pathological fracture, left shoulder, subsequent encounter for fracture with routine healing |
| M84.412G | Pathological fracture, left shoulder, subsequent encounter for fracture with delayed healing |
| M84.412K | Pathological fracture, left shoulder, subsequent encounter for fracture with nonunion |
| M84.412P | Pathological fracture, left shoulder, subsequent encounter for fracture with malunion |
| M84.412S | Pathological fracture, left shoulder, sequela |
| M84.419 | Pathological fracture, unspecified shoulder |
| M84.419A | Pathological fracture, unspecified shoulder, initial encounter for fracture |
| M84.419D | Pathological fracture, unspecified shoulder, subsequent encounter for fracture with routine healing |
| M84.419G | Pathological fracture, unspecified shoulder, subsequent encounter for fracture with delayed healing |
| M84.419K | Pathological fracture, unspecified shoulder, subsequent encounter for fracture with nonunion |
| M84.419P | Pathological fracture, unspecified shoulder, subsequent encounter for fracture with malunion |
| M84.419S | Pathological fracture, unspecified shoulder, sequela |
| M84.42 | Pathological fracture, humerus |
| M84.421 | Pathological fracture, right humerus |
| M84.421A | Pathological fracture, right humerus, initial encounter for fracture |
| M84.421D | Pathological fracture, right humerus, subsequent encounter for fracture with routine healing |
| M84.421G | Pathological fracture, right humerus, subsequent encounter for fracture with delayed healing |
| M84.421K | Pathological fracture, right humerus, subsequent encounter for fracture with nonunion |
| M84.421P | Pathological fracture, right humerus, subsequent encounter for fracture with malunion |
| M84.421S | Pathological fracture, right humerus, sequela |
| M84.422 | Pathological fracture, left humerus |
| M84.422A | Pathological fracture, left humerus, initial encounter for fracture |
| M84.422D | Pathological fracture, left humerus, subsequent encounter for fracture with routine healing |
| M84.422G | Pathological fracture, left humerus, subsequent encounter for fracture with delayed healing |
| M84.422K | Pathological fracture, left humerus, subsequent encounter for fracture with nonunion |
| M84.422P | Pathological fracture, left humerus, subsequent encounter for fracture with malunion |
| M84.422S | Pathological fracture, left humerus, sequela |
| M84.429 | Pathological fracture, unspecified humerus |
| M84.429A | Pathological fracture, unspecified humerus, initial encounter for fracture |
| M84.429D | Pathological fracture, unspecified humerus, subsequent encounter for fracture with routine healing |
| M84.429G | Pathological fracture, unspecified humerus, subsequent encounter for fracture with delayed healing |
| M84.429K | Pathological fracture, unspecified humerus, subsequent encounter for fracture with nonunion |
| M84.429P | Pathological fracture, unspecified humerus, subsequent encounter for fracture with malunion |
| M84.429S | Pathological fracture, unspecified humerus, sequela |
| M84.43 | Pathological fracture, ulna and radius |
| M84.431 | Pathological fracture, right ulna |
| M84.431A | Pathological fracture, right ulna, initial encounter for fracture |
| M84.431D | Pathological fracture, right ulna, subsequent encounter for fracture with routine healing |
| M84.431G | Pathological fracture, right ulna, subsequent encounter for fracture with delayed healing |
| M84.431K | Pathological fracture, right ulna, subsequent encounter for fracture with nonunion |
| M84.431P | Pathological fracture, right ulna, subsequent encounter for fracture with malunion |
| M84.431S | Pathological fracture, right ulna, sequela |
| M84.432 | Pathological fracture, left ulna |
| M84.432A | Pathological fracture, left ulna, initial encounter for fracture |
| M84.432D | Pathological fracture, left ulna, subsequent encounter for fracture with routine healing |
| M84.432G | Pathological fracture, left ulna, subsequent encounter for fracture with delayed healing |
| M84.432K | Pathological fracture, left ulna, subsequent encounter for fracture with nonunion |
| M84.432P | Pathological fracture, left ulna, subsequent encounter for fracture with malunion |
| M84.432S | Pathological fracture, left ulna, sequela |
| M84.433 | Pathological fracture, right radius |
| M84.433A | Pathological fracture, right radius, initial encounter for fracture |
| M84.433D | Pathological fracture, right radius, subsequent encounter for fracture with routine healing |
| M84.433G | Pathological fracture, right radius, subsequent encounter for fracture with delayed healing |
| M84.433K | Pathological fracture, right radius, subsequent encounter for fracture with nonunion |
| M84.433P | Pathological fracture, right radius, subsequent encounter for fracture with malunion |
| M84.433S | Pathological fracture, right radius, sequela |
| M84.434 | Pathological fracture, left radius |
| M84.434A | Pathological fracture, left radius, initial encounter for fracture |
| M84.434D | Pathological fracture, left radius, subsequent encounter for fracture with routine healing |
| M84.434G | Pathological fracture, left radius, subsequent encounter for fracture with delayed healing |
| M84.434K | Pathological fracture, left radius, subsequent encounter for fracture with nonunion |
| M84.434P | Pathological fracture, left radius, subsequent encounter for fracture with malunion |
| M84.434S | Pathological fracture, left radius, sequela |
| M84.439 | Pathological fracture, unspecified ulna and radius |
| M84.439A | Pathological fracture, unspecified ulna and radius, initial encounter for fracture |
| M84.439D | Pathological fracture, unspecified ulna and radius, subsequent encounter for fracture with routine healing |
| M84.439G | Pathological fracture, unspecified ulna and radius, subsequent encounter for fracture with delayed healing |
| M84.439K | Pathological fracture, unspecified ulna and radius, subsequent encounter for fracture with nonunion |
| M84.439P | Pathological fracture, unspecified ulna and radius, subsequent encounter for fracture with malunion |
| M84.439S | Pathological fracture, unspecified ulna and radius, sequela |
| M84.44 | Pathological fracture, hand and fingers |
| M84.441 | Pathological fracture, right hand |
| M84.441A | Pathological fracture, right hand, initial encounter for fracture |
| M84.441D | Pathological fracture, right hand, subsequent encounter for fracture with routine healing |
| M84.441G | Pathological fracture, right hand, subsequent encounter for fracture with delayed healing |
| M84.441K | Pathological fracture, right hand, subsequent encounter for fracture with nonunion |
| M84.441P | Pathological fracture, right hand, subsequent encounter for fracture with malunion |
| M84.441S | Pathological fracture, right hand, sequela |
| M84.442 | Pathological fracture, left hand |
| M84.442A | Pathological fracture, left hand, initial encounter for fracture |
| M84.442D | Pathological fracture, left hand, subsequent encounter for fracture with routine healing |
| M84.442G | Pathological fracture, left hand, subsequent encounter for fracture with delayed healing |
| M84.442K | Pathological fracture, left hand, subsequent encounter for fracture with nonunion |
| M84.442P | Pathological fracture, left hand, subsequent encounter for fracture with malunion |
| M84.442S | Pathological fracture, left hand, sequela |
| M84.443 | Pathological fracture, unspecified hand |
| M84.443A | Pathological fracture, unspecified hand, initial encounter for fracture |
| M84.443D | Pathological fracture, unspecified hand, subsequent encounter for fracture with routine healing |
| M84.443G | Pathological fracture, unspecified hand, subsequent encounter for fracture with delayed healing |
| M84.443K | Pathological fracture, unspecified hand, subsequent encounter for fracture with nonunion |
| M84.443P | Pathological fracture, unspecified hand, subsequent encounter for fracture with malunion |
| M84.443S | Pathological fracture, unspecified hand, sequela |
| M84.444 | Pathological fracture, right finger(s) |
| M84.444A | Pathological fracture, right finger(s), initial encounter for fracture |
| M84.444D | Pathological fracture, right finger(s), subsequent encounter for fracture with routine healing |
| M84.444G | Pathological fracture, right finger(s), subsequent encounter for fracture with delayed healing |
| M84.444K | Pathological fracture, right finger(s), subsequent encounter for fracture with nonunion |
| M84.444P | Pathological fracture, right finger(s), subsequent encounter for fracture with malunion |
| M84.444S | Pathological fracture, right finger(s), sequela |
| M84.445 | Pathological fracture, left finger(s) |
| M84.445A | Pathological fracture, left finger(s), initial encounter for fracture |
| M84.445D | Pathological fracture, left finger(s), subsequent encounter for fracture with routine healing |
| M84.445G | Pathological fracture, left finger(s), subsequent encounter for fracture with delayed healing |
| M84.445K | Pathological fracture, left finger(s), subsequent encounter for fracture with nonunion |
| M84.445P | Pathological fracture, left finger(s), subsequent encounter for fracture with malunion |
| M84.445S | Pathological fracture, left finger(s), sequela |
| M84.446 | Pathological fracture, unspecified finger(s) |
| M84.446A | Pathological fracture, unspecified finger(s), initial encounter for fracture |
| M84.446D | Pathological fracture, unspecified finger(s), subsequent encounter for fracture with routine healing |
| M84.446G | Pathological fracture, unspecified finger(s), subsequent encounter for fracture with delayed healing |
| M84.446K | Pathological fracture, unspecified finger(s), subsequent encounter for fracture with nonunion |
| M84.446P | Pathological fracture, unspecified finger(s), subsequent encounter for fracture with malunion |
| M84.446S | Pathological fracture, unspecified finger(s), sequela |
| M84.45 | Pathological fracture, femur and pelvis |
| M84.451 | Pathological fracture, right femur |
| M84.451A | Pathological fracture, right femur, initial encounter for fracture |
| M84.451D | Pathological fracture, right femur, subsequent encounter for fracture with routine healing |
| M84.451G | Pathological fracture, right femur, subsequent encounter for fracture with delayed healing |
| M84.451K | Pathological fracture, right femur, subsequent encounter for fracture with nonunion |
| M84.451P | Pathological fracture, right femur, subsequent encounter for fracture with malunion |
| M84.451S | Pathological fracture, right femur, sequela |
| M84.452 | Pathological fracture, left femur |
| M84.452A | Pathological fracture, left femur, initial encounter for fracture |
| M84.452D | Pathological fracture, left femur, subsequent encounter for fracture with routine healing |
| M84.452G | Pathological fracture, left femur, subsequent encounter for fracture with delayed healing |
| M84.452K | Pathological fracture, left femur, subsequent encounter for fracture with nonunion |
| M84.452P | Pathological fracture, left femur, subsequent encounter for fracture with malunion |
| M84.452S | Pathological fracture, left femur, sequela |
| M84.453 | Pathological fracture, unspecified femur |
| M84.453A | Pathological fracture, unspecified femur, initial encounter for fracture |
| M84.453D | Pathological fracture, unspecified femur, subsequent encounter for fracture with routine healing |
| M84.453G | Pathological fracture, unspecified femur, subsequent encounter for fracture with delayed healing |
| M84.453K | Pathological fracture, unspecified femur, subsequent encounter for fracture with nonunion |
| M84.453P | Pathological fracture, unspecified femur, subsequent encounter for fracture with malunion |
| M84.453S | Pathological fracture, unspecified femur, sequela |
| M84.454 | Pathological fracture, pelvis |
| M84.454A | Pathological fracture, pelvis, initial encounter for fracture |
| M84.454D | Pathological fracture, pelvis, subsequent encounter for fracture with routine healing |
| M84.454G | Pathological fracture, pelvis, subsequent encounter for fracture with delayed healing |
| M84.454K | Pathological fracture, pelvis, subsequent encounter for fracture with nonunion |
| M84.454P | Pathological fracture, pelvis, subsequent encounter for fracture with malunion |
| M84.454S | Pathological fracture, pelvis, sequela |
| M84.459 | Pathological fracture, hip, unspecified |
| M84.459A | Pathological fracture, hip, unspecified, initial encounter for fracture |
| M84.459D | Pathological fracture, hip, unspecified, subsequent encounter for fracture with routine healing |
| M84.459G | Pathological fracture, hip, unspecified, subsequent encounter for fracture with delayed healing |
| M84.459K | Pathological fracture, hip, unspecified, subsequent encounter for fracture with nonunion |
| M84.459P | Pathological fracture, hip, unspecified, subsequent encounter for fracture with malunion |
| M84.459S | Pathological fracture, hip, unspecified, sequela |
| M84.46 | Pathological fracture, tibia and fibula |
| M84.461 | Pathological fracture, right tibia |
| M84.461A | Pathological fracture, right tibia, initial encounter for fracture |
| M84.461D | Pathological fracture, right tibia, subsequent encounter for fracture with routine healing |
| M84.461G | Pathological fracture, right tibia, subsequent encounter for fracture with delayed healing |
| M84.461K | Pathological fracture, right tibia, subsequent encounter for fracture with nonunion |
| M84.461P | Pathological fracture, right tibia, subsequent encounter for fracture with malunion |
| M84.461S | Pathological fracture, right tibia, sequela |
| M84.462 | Pathological fracture, left tibia |
| M84.462A | Pathological fracture, left tibia, initial encounter for fracture |
| M84.462D | Pathological fracture, left tibia, subsequent encounter for fracture with routine healing |
| M84.462G | Pathological fracture, left tibia, subsequent encounter for fracture with delayed healing |
| M84.462K | Pathological fracture, left tibia, subsequent encounter for fracture with nonunion |
| M84.462P | Pathological fracture, left tibia, subsequent encounter for fracture with malunion |
| M84.462S | Pathological fracture, left tibia, sequela |
| M84.463 | Pathological fracture, right fibula |
| M84.463A | Pathological fracture, right fibula, initial encounter for fracture |
| M84.463D | Pathological fracture, right fibula, subsequent encounter for fracture with routine healing |
| M84.463G | Pathological fracture, right fibula, subsequent encounter for fracture with delayed healing |
| M84.463K | Pathological fracture, right fibula, subsequent encounter for fracture with nonunion |
| M84.463P | Pathological fracture, right fibula, subsequent encounter for fracture with malunion |
| M84.463S | Pathological fracture, right fibula, sequela |
| M84.464 | Pathological fracture, left fibula |
| M84.464A | Pathological fracture, left fibula, initial encounter for fracture |
| M84.464D | Pathological fracture, left fibula, subsequent encounter for fracture with routine healing |
| M84.464G | Pathological fracture, left fibula, subsequent encounter for fracture with delayed healing |
| M84.464K | Pathological fracture, left fibula, subsequent encounter for fracture with nonunion |
| M84.464P | Pathological fracture, left fibula, subsequent encounter for fracture with malunion |
| M84.464S | Pathological fracture, left fibula, sequela |
| M84.469 | Pathological fracture, unspecified tibia and fibula |
| M84.469A | Pathological fracture, unspecified tibia and fibula, initial encounter for fracture |
| M84.469D | Pathological fracture, unspecified tibia and fibula, subsequent encounter for fracture with routine healing |
| M84.469G | Pathological fracture, unspecified tibia and fibula, subsequent encounter for fracture with delayed healing |
| M84.469K | Pathological fracture, unspecified tibia and fibula, subsequent encounter for fracture with nonunion |
| M84.469P | Pathological fracture, unspecified tibia and fibula, subsequent encounter for fracture with malunion |
| M84.469S | Pathological fracture, unspecified tibia and fibula, sequela |
| M84.47 | Pathological fracture, ankle, foot and toes |
| M84.471 | Pathological fracture, right ankle |
| M84.471A | Pathological fracture, right ankle, initial encounter for fracture |
| M84.471D | Pathological fracture, right ankle, subsequent encounter for fracture with routine healing |
| M84.471G | Pathological fracture, right ankle, subsequent encounter for fracture with delayed healing |
| M84.471K | Pathological fracture, right ankle, subsequent encounter for fracture with nonunion |
| M84.471P | Pathological fracture, right ankle, subsequent encounter for fracture with malunion |
| M84.471S | Pathological fracture, right ankle, sequela |
| M84.472 | Pathological fracture, left ankle |
| M84.472A | Pathological fracture, left ankle, initial encounter for fracture |
| M84.472D | Pathological fracture, left ankle, subsequent encounter for fracture with routine healing |
| M84.472G | Pathological fracture, left ankle, subsequent encounter for fracture with delayed healing |
| M84.472K | Pathological fracture, left ankle, subsequent encounter for fracture with nonunion |
| M84.472P | Pathological fracture, left ankle, subsequent encounter for fracture with malunion |
| M84.472S | Pathological fracture, left ankle, sequela |
| M84.473 | Pathological fracture, unspecified ankle |
| M84.473A | Pathological fracture, unspecified ankle, initial encounter for fracture |
| M84.473D | Pathological fracture, unspecified ankle, subsequent encounter for fracture with routine healing |
| M84.473G | Pathological fracture, unspecified ankle, subsequent encounter for fracture with delayed healing |
| M84.473K | Pathological fracture, unspecified ankle, subsequent encounter for fracture with nonunion |
| M84.473P | Pathological fracture, unspecified ankle, subsequent encounter for fracture with malunion |
| M84.473S | Pathological fracture, unspecified ankle, sequela |
| M84.474 | Pathological fracture, right foot |
| M84.474A | Pathological fracture, right foot, initial encounter for fracture |
| M84.474D | Pathological fracture, right foot, subsequent encounter for fracture with routine healing |
| M84.474G | Pathological fracture, right foot, subsequent encounter for fracture with delayed healing |
| M84.474K | Pathological fracture, right foot, subsequent encounter for fracture with nonunion |
| M84.474P | Pathological fracture, right foot, subsequent encounter for fracture with malunion |
| M84.474S | Pathological fracture, right foot, sequela |
| M84.475 | Pathological fracture, left foot |
| M84.475A | Pathological fracture, left foot, initial encounter for fracture |
| M84.475D | Pathological fracture, left foot, subsequent encounter for fracture with routine healing |
| M84.475G | Pathological fracture, left foot, subsequent encounter for fracture with delayed healing |
| M84.475K | Pathological fracture, left foot, subsequent encounter for fracture with nonunion |
| M84.475P | Pathological fracture, left foot, subsequent encounter for fracture with malunion |
| M84.475S | Pathological fracture, left foot, sequela |
| M84.476 | Pathological fracture, unspecified foot |
| M84.476A | Pathological fracture, unspecified foot, initial encounter for fracture |
| M84.476D | Pathological fracture, unspecified foot, subsequent encounter for fracture with routine healing |
| M84.476G | Pathological fracture, unspecified foot, subsequent encounter for fracture with delayed healing |
| M84.476K | Pathological fracture, unspecified foot, subsequent encounter for fracture with nonunion |
| M84.476P | Pathological fracture, unspecified foot, subsequent encounter for fracture with malunion |
| M84.476S | Pathological fracture, unspecified foot, sequela |
| M84.477 | Pathological fracture, right toe(s) |
| M84.477A | Pathological fracture, right toe(s), initial encounter for fracture |
| M84.477D | Pathological fracture, right toe(s), subsequent encounter for fracture with routine healing |
| M84.477G | Pathological fracture, right toe(s), subsequent encounter for fracture with delayed healing |
| M84.477K | Pathological fracture, right toe(s), subsequent encounter for fracture with nonunion |
| M84.477P | Pathological fracture, right toe(s), subsequent encounter for fracture with malunion |
| M84.477S | Pathological fracture, right toe(s), sequela |
| M84.478 | Pathological fracture, left toe(s) |
| M84.478A | Pathological fracture, left toe(s), initial encounter for fracture |
| M84.478D | Pathological fracture, left toe(s), subsequent encounter for fracture with routine healing |
| M84.478G | Pathological fracture, left toe(s), subsequent encounter for fracture with delayed healing |
| M84.478K | Pathological fracture, left toe(s), subsequent encounter for fracture with nonunion |
| M84.478P | Pathological fracture, left toe(s), subsequent encounter for fracture with malunion |
| M84.478S | Pathological fracture, left toe(s), sequela |
| M84.479 | Pathological fracture, unspecified toe(s) |
| M84.479A | Pathological fracture, unspecified toe(s), initial encounter for fracture |
| M84.479D | Pathological fracture, unspecified toe(s), subsequent encounter for fracture with routine healing |
| M84.479G | Pathological fracture, unspecified toe(s), subsequent encounter for fracture with delayed healing |
| M84.479K | Pathological fracture, unspecified toe(s), subsequent encounter for fracture with nonunion |
| M84.479P | Pathological fracture, unspecified toe(s), subsequent encounter for fracture with malunion |
| M84.479S | Pathological fracture, unspecified toe(s), sequela |
| M84.48 | Pathological fracture, other site |
| M84.48XA | Pathological fracture, other site, initial encounter for fracture |
| M84.48XD | Pathological fracture, other site, subsequent encounter for fracture with routine healing |
| M84.48XG | Pathological fracture, other site, subsequent encounter for fracture with delayed healing |
| M84.48XK | Pathological fracture, other site, subsequent encounter for fracture with nonunion |
| M84.48XP | Pathological fracture, other site, subsequent encounter for fracture with malunion |
| M84.48XS | Pathological fracture, other site, sequela |
| M84.5 | Pathological fracture in neoplastic disease |
| M84.50 | Pathological fracture in neoplastic disease, unspecified site |
| M84.50XA | Pathological fracture in neoplastic disease, unspecified site, initial encounter for fracture |
| M84.50XD | Pathological fracture in neoplastic disease, unspecified site, subsequent encounter for fracture with routine healing |
| M84.50XG | Pathological fracture in neoplastic disease, unspecified site, subsequent encounter for fracture with delayed healing |
| M84.50XK | Pathological fracture in neoplastic disease, unspecified site, subsequent encounter for fracture with nonunion |
| M84.50XP | Pathological fracture in neoplastic disease, unspecified site, subsequent encounter for fracture with malunion |
| M84.50XS | Pathological fracture in neoplastic disease, unspecified site, sequela |
| M84.51 | Pathological fracture in neoplastic disease, shoulder |
| M84.511 | Pathological fracture in neoplastic disease, right shoulder |
| M84.511A | Pathological fracture in neoplastic disease, right shoulder, initial encounter for fracture |
| M84.511D | Pathological fracture in neoplastic disease, right shoulder, subsequent encounter for fracture with routine healing |
| M84.511G | Pathological fracture in neoplastic disease, right shoulder, subsequent encounter for fracture with delayed healing |
| M84.511K | Pathological fracture in neoplastic disease, right shoulder, subsequent encounter for fracture with nonunion |
| M84.511P | Pathological fracture in neoplastic disease, right shoulder, subsequent encounter for fracture with malunion |
| M84.511S | Pathological fracture in neoplastic disease, right shoulder, sequela |
| M84.512 | Pathological fracture in neoplastic disease, left shoulder |
| M84.512A | Pathological fracture in neoplastic disease, left shoulder, initial encounter for fracture |
| M84.512D | Pathological fracture in neoplastic disease, left shoulder, subsequent encounter for fracture with routine healing |
| M84.512G | Pathological fracture in neoplastic disease, left shoulder, subsequent encounter for fracture with delayed healing |
| M84.512K | Pathological fracture in neoplastic disease, left shoulder, subsequent encounter for fracture with nonunion |
| M84.512P | Pathological fracture in neoplastic disease, left shoulder, subsequent encounter for fracture with malunion |
| M84.512S | Pathological fracture in neoplastic disease, left shoulder, sequela |
| M84.519 | Pathological fracture in neoplastic disease, unspecified shoulder |
| M84.519A | Pathological fracture in neoplastic disease, unspecified shoulder, initial encounter for fracture |
| M84.519D | Pathological fracture in neoplastic disease, unspecified shoulder, subsequent encounter for fracture with routine healing |
| M84.519G | Pathological fracture in neoplastic disease, unspecified shoulder, subsequent encounter for fracture with delayed healing |
| M84.519K | Pathological fracture in neoplastic disease, unspecified shoulder, subsequent encounter for fracture with nonunion |
| M84.519P | Pathological fracture in neoplastic disease, unspecified shoulder, subsequent encounter for fracture with malunion |
| M84.519S | Pathological fracture in neoplastic disease, unspecified shoulder, sequela |
| M84.52 | Pathological fracture in neoplastic disease, humerus |
| M84.521 | Pathological fracture in neoplastic disease, right humerus |
| M84.521A | Pathological fracture in neoplastic disease, right humerus, initial encounter for fracture |
| M84.521D | Pathological fracture in neoplastic disease, right humerus, subsequent encounter for fracture with routine healing |
| M84.521G | Pathological fracture in neoplastic disease, right humerus, subsequent encounter for fracture with delayed healing |
| M84.521K | Pathological fracture in neoplastic disease, right humerus, subsequent encounter for fracture with nonunion |
| M84.521P | Pathological fracture in neoplastic disease, right humerus, subsequent encounter for fracture with malunion |
| M84.521S | Pathological fracture in neoplastic disease, right humerus, sequela |
| M84.522 | Pathological fracture in neoplastic disease, left humerus |
| M84.522A | Pathological fracture in neoplastic disease, left humerus, initial encounter for fracture |
| M84.522D | Pathological fracture in neoplastic disease, left humerus, subsequent encounter for fracture with routine healing |
| M84.522G | Pathological fracture in neoplastic disease, left humerus, subsequent encounter for fracture with delayed healing |
| M84.522K | Pathological fracture in neoplastic disease, left humerus, subsequent encounter for fracture with nonunion |
| M84.522P | Pathological fracture in neoplastic disease, left humerus, subsequent encounter for fracture with malunion |
| M84.522S | Pathological fracture in neoplastic disease, left humerus, sequela |
| M84.529 | Pathological fracture in neoplastic disease, unspecified humerus |
| M84.529A | Pathological fracture in neoplastic disease, unspecified humerus, initial encounter for fracture |
| M84.529D | Pathological fracture in neoplastic disease, unspecified humerus, subsequent encounter for fracture with routine healing |
| M84.529G | Pathological fracture in neoplastic disease, unspecified humerus, subsequent encounter for fracture with delayed healing |
| M84.529K | Pathological fracture in neoplastic disease, unspecified humerus, subsequent encounter for fracture with nonunion |
| M84.529P | Pathological fracture in neoplastic disease, unspecified humerus, subsequent encounter for fracture with malunion |
| M84.529S | Pathological fracture in neoplastic disease, unspecified humerus, sequela |
| M84.53 | Pathological fracture in neoplastic disease, ulna and radius |
| M84.531 | Pathological fracture in neoplastic disease, right ulna |
| M84.531A | Pathological fracture in neoplastic disease, right ulna, initial encounter for fracture |
| M84.531D | Pathological fracture in neoplastic disease, right ulna, subsequent encounter for fracture with routine healing |
| M84.531G | Pathological fracture in neoplastic disease, right ulna, subsequent encounter for fracture with delayed healing |
| M84.531K | Pathological fracture in neoplastic disease, right ulna, subsequent encounter for fracture with nonunion |
| M84.531P | Pathological fracture in neoplastic disease, right ulna, subsequent encounter for fracture with malunion |
| M84.531S | Pathological fracture in neoplastic disease, right ulna, sequela |
| M84.532 | Pathological fracture in neoplastic disease, left ulna |
| M84.532A | Pathological fracture in neoplastic disease, left ulna, initial encounter for fracture |
| M84.532D | Pathological fracture in neoplastic disease, left ulna, subsequent encounter for fracture with routine healing |
| M84.532G | Pathological fracture in neoplastic disease, left ulna, subsequent encounter for fracture with delayed healing |
| M84.532K | Pathological fracture in neoplastic disease, left ulna, subsequent encounter for fracture with nonunion |
| M84.532P | Pathological fracture in neoplastic disease, left ulna, subsequent encounter for fracture with malunion |
| M84.532S | Pathological fracture in neoplastic disease, left ulna, sequela |
| M84.533 | Pathological fracture in neoplastic disease, right radius |
| M84.533A | Pathological fracture in neoplastic disease, right radius, initial encounter for fracture |
| M84.533D | Pathological fracture in neoplastic disease, right radius, subsequent encounter for fracture with routine healing |
| M84.533G | Pathological fracture in neoplastic disease, right radius, subsequent encounter for fracture with delayed healing |
| M84.533K | Pathological fracture in neoplastic disease, right radius, subsequent encounter for fracture with nonunion |
| M84.533P | Pathological fracture in neoplastic disease, right radius, subsequent encounter for fracture with malunion |
| M84.533S | Pathological fracture in neoplastic disease, right radius, sequela |
| M84.534 | Pathological fracture in neoplastic disease, left radius |
| M84.534A | Pathological fracture in neoplastic disease, left radius, initial encounter for fracture |
| M84.534D | Pathological fracture in neoplastic disease, left radius, subsequent encounter for fracture with routine healing |
| M84.534G | Pathological fracture in neoplastic disease, left radius, subsequent encounter for fracture with delayed healing |
| M84.534K | Pathological fracture in neoplastic disease, left radius, subsequent encounter for fracture with nonunion |
| M84.534P | Pathological fracture in neoplastic disease, left radius, subsequent encounter for fracture with malunion |
| M84.534S | Pathological fracture in neoplastic disease, left radius, sequela |
| M84.539 | Pathological fracture in neoplastic disease, unspecified ulna and radius |
| M84.539A | Pathological fracture in neoplastic disease, unspecified ulna and radius, initial encounter for fracture |
| M84.539D | Pathological fracture in neoplastic disease, unspecified ulna and radius, subsequent encounter for fracture with routine healing |
| M84.539G | Pathological fracture in neoplastic disease, unspecified ulna and radius, subsequent encounter for fracture with delayed healing |
| M84.539K | Pathological fracture in neoplastic disease, unspecified ulna and radius, subsequent encounter for fracture with nonunion |
| M84.539P | Pathological fracture in neoplastic disease, unspecified ulna and radius, subsequent encounter for fracture with malunion |
| M84.539S | Pathological fracture in neoplastic disease, unspecified ulna and radius, sequela |
| M84.54 | Pathological fracture in neoplastic disease, hand |
| M84.541 | Pathological fracture in neoplastic disease, right hand |
| M84.541A | Pathological fracture in neoplastic disease, right hand, initial encounter for fracture |
| M84.541D | Pathological fracture in neoplastic disease, right hand, subsequent encounter for fracture with routine healing |
| M84.541G | Pathological fracture in neoplastic disease, right hand, subsequent encounter for fracture with delayed healing |
| M84.541K | Pathological fracture in neoplastic disease, right hand, subsequent encounter for fracture with nonunion |
| M84.541P | Pathological fracture in neoplastic disease, right hand, subsequent encounter for fracture with malunion |
| M84.541S | Pathological fracture in neoplastic disease, right hand, sequela |
| M84.542 | Pathological fracture in neoplastic disease, left hand |
| M84.542A | Pathological fracture in neoplastic disease, left hand, initial encounter for fracture |
| M84.542D | Pathological fracture in neoplastic disease, left hand, subsequent encounter for fracture with routine healing |
| M84.542G | Pathological fracture in neoplastic disease, left hand, subsequent encounter for fracture with delayed healing |
| M84.542K | Pathological fracture in neoplastic disease, left hand, subsequent encounter for fracture with nonunion |
| M84.542P | Pathological fracture in neoplastic disease, left hand, subsequent encounter for fracture with malunion |
| M84.542S | Pathological fracture in neoplastic disease, left hand, sequela |
| M84.549 | Pathological fracture in neoplastic disease, unspecified hand |
| M84.549A | Pathological fracture in neoplastic disease, unspecified hand, initial encounter for fracture |
| M84.549D | Pathological fracture in neoplastic disease, unspecified hand, subsequent encounter for fracture with routine healing |
| M84.549G | Pathological fracture in neoplastic disease, unspecified hand, subsequent encounter for fracture with delayed healing |
| M84.549K | Pathological fracture in neoplastic disease, unspecified hand, subsequent encounter for fracture with nonunion |
| M84.549P | Pathological fracture in neoplastic disease, unspecified hand, subsequent encounter for fracture with malunion |
| M84.549S | Pathological fracture in neoplastic disease, unspecified hand, sequela |
| M84.55 | Pathological fracture in neoplastic disease, pelvis and femur |
| M84.550 | Pathological fracture in neoplastic disease, pelvis |
| M84.550A | Pathological fracture in neoplastic disease, pelvis, initial encounter for fracture |
| M84.550D | Pathological fracture in neoplastic disease, pelvis, subsequent encounter for fracture with routine healing |
| M84.550G | Pathological fracture in neoplastic disease, pelvis, subsequent encounter for fracture with delayed healing |
| M84.550K | Pathological fracture in neoplastic disease, pelvis, subsequent encounter for fracture with nonunion |
| M84.550P | Pathological fracture in neoplastic disease, pelvis, subsequent encounter for fracture with malunion |
| M84.550S | Pathological fracture in neoplastic disease, pelvis, sequela |
| M84.551 | Pathological fracture in neoplastic disease, right femur |
| M84.551A | Pathological fracture in neoplastic disease, right femur, initial encounter for fracture |
| M84.551D | Pathological fracture in neoplastic disease, right femur, subsequent encounter for fracture with routine healing |
| M84.551G | Pathological fracture in neoplastic disease, right femur, subsequent encounter for fracture with delayed healing |
| M84.551K | Pathological fracture in neoplastic disease, right femur, subsequent encounter for fracture with nonunion |
| M84.551P | Pathological fracture in neoplastic disease, right femur, subsequent encounter for fracture with malunion |
| M84.551S | Pathological fracture in neoplastic disease, right femur, sequela |
| M84.552 | Pathological fracture in neoplastic disease, left femur |
| M84.552A | Pathological fracture in neoplastic disease, left femur, initial encounter for fracture |
| M84.552D | Pathological fracture in neoplastic disease, left femur, subsequent encounter for fracture with routine healing |
| M84.552G | Pathological fracture in neoplastic disease, left femur, subsequent encounter for fracture with delayed healing |
| M84.552K | Pathological fracture in neoplastic disease, left femur, subsequent encounter for fracture with nonunion |
| M84.552P | Pathological fracture in neoplastic disease, left femur, subsequent encounter for fracture with malunion |
| M84.552S | Pathological fracture in neoplastic disease, left femur, sequela |
| M84.553 | Pathological fracture in neoplastic disease, unspecified femur |
| M84.553A | Pathological fracture in neoplastic disease, unspecified femur, initial encounter for fracture |
| M84.553D | Pathological fracture in neoplastic disease, unspecified femur, subsequent encounter for fracture with routine healing |
| M84.553G | Pathological fracture in neoplastic disease, unspecified femur, subsequent encounter for fracture with delayed healing |
| M84.553K | Pathological fracture in neoplastic disease, unspecified femur, subsequent encounter for fracture with nonunion |
| M84.553P | Pathological fracture in neoplastic disease, unspecified femur, subsequent encounter for fracture with malunion |
| M84.553S | Pathological fracture in neoplastic disease, unspecified femur, sequela |
| M84.559 | Pathological fracture in neoplastic disease, hip, unspecified |
| M84.559A | Pathological fracture in neoplastic disease, hip, unspecified, initial encounter for fracture |
| M84.559D | Pathological fracture in neoplastic disease, hip, unspecified, subsequent encounter for fracture with routine healing |
| M84.559G | Pathological fracture in neoplastic disease, hip, unspecified, subsequent encounter for fracture with delayed healing |
| M84.559K | Pathological fracture in neoplastic disease, hip, unspecified, subsequent encounter for fracture with nonunion |
| M84.559P | Pathological fracture in neoplastic disease, hip, unspecified, subsequent encounter for fracture with malunion |
| M84.559S | Pathological fracture in neoplastic disease, hip, unspecified, sequela |
| M84.56 | Pathological fracture in neoplastic disease, tibia and fibula |
| M84.561 | Pathological fracture in neoplastic disease, right tibia |
| M84.561A | Pathological fracture in neoplastic disease, right tibia, initial encounter for fracture |
| M84.561D | Pathological fracture in neoplastic disease, right tibia, subsequent encounter for fracture with routine healing |
| M84.561G | Pathological fracture in neoplastic disease, right tibia, subsequent encounter for fracture with delayed healing |
| M84.561K | Pathological fracture in neoplastic disease, right tibia, subsequent encounter for fracture with nonunion |
| M84.561P | Pathological fracture in neoplastic disease, right tibia, subsequent encounter for fracture with malunion |
| M84.561S | Pathological fracture in neoplastic disease, right tibia, sequela |
| M84.562 | Pathological fracture in neoplastic disease, left tibia |
| M84.562A | Pathological fracture in neoplastic disease, left tibia, initial encounter for fracture |
| M84.562D | Pathological fracture in neoplastic disease, left tibia, subsequent encounter for fracture with routine healing |
| M84.562G | Pathological fracture in neoplastic disease, left tibia, subsequent encounter for fracture with delayed healing |
| M84.562K | Pathological fracture in neoplastic disease, left tibia, subsequent encounter for fracture with nonunion |
| M84.562P | Pathological fracture in neoplastic disease, left tibia, subsequent encounter for fracture with malunion |
| M84.562S | Pathological fracture in neoplastic disease, left tibia, sequela |
| M84.563 | Pathological fracture in neoplastic disease, right fibula |
| M84.563A | Pathological fracture in neoplastic disease, right fibula, initial encounter for fracture |
| M84.563D | Pathological fracture in neoplastic disease, right fibula, subsequent encounter for fracture with routine healing |
| M84.563G | Pathological fracture in neoplastic disease, right fibula, subsequent encounter for fracture with delayed healing |
| M84.563K | Pathological fracture in neoplastic disease, right fibula, subsequent encounter for fracture with nonunion |
| M84.563P | Pathological fracture in neoplastic disease, right fibula, subsequent encounter for fracture with malunion |
| M84.563S | Pathological fracture in neoplastic disease, right fibula, sequela |
| M84.564 | Pathological fracture in neoplastic disease, left fibula |
| M84.564A | Pathological fracture in neoplastic disease, left fibula, initial encounter for fracture |
| M84.564D | Pathological fracture in neoplastic disease, left fibula, subsequent encounter for fracture with routine healing |
| M84.564G | Pathological fracture in neoplastic disease, left fibula, subsequent encounter for fracture with delayed healing |
| M84.564K | Pathological fracture in neoplastic disease, left fibula, subsequent encounter for fracture with nonunion |
| M84.564P | Pathological fracture in neoplastic disease, left fibula, subsequent encounter for fracture with malunion |
| M84.564S | Pathological fracture in neoplastic disease, left fibula, sequela |
| M84.569 | Pathological fracture in neoplastic disease, unspecified tibia and fibula |
| M84.569A | Pathological fracture in neoplastic disease, unspecified tibia and fibula, initial encounter for fracture |
| M84.569D | Pathological fracture in neoplastic disease, unspecified tibia and fibula, subsequent encounter for fracture with routine healing |
| M84.569G | Pathological fracture in neoplastic disease, unspecified tibia and fibula, subsequent encounter for fracture with delayed healing |
| M84.569K | Pathological fracture in neoplastic disease, unspecified tibia and fibula, subsequent encounter for fracture with nonunion |
| M84.569P | Pathological fracture in neoplastic disease, unspecified tibia and fibula, subsequent encounter for fracture with malunion |
| M84.569S | Pathological fracture in neoplastic disease, unspecified tibia and fibula, sequela |
| M84.57 | Pathological fracture in neoplastic disease, ankle and foot |
| M84.571 | Pathological fracture in neoplastic disease, right ankle |
| M84.571A | Pathological fracture in neoplastic disease, right ankle, initial encounter for fracture |
| M84.571D | Pathological fracture in neoplastic disease, right ankle, subsequent encounter for fracture with routine healing |
| M84.571G | Pathological fracture in neoplastic disease, right ankle, subsequent encounter for fracture with delayed healing |
| M84.571K | Pathological fracture in neoplastic disease, right ankle, subsequent encounter for fracture with nonunion |
| M84.571P | Pathological fracture in neoplastic disease, right ankle, subsequent encounter for fracture with malunion |
| M84.571S | Pathological fracture in neoplastic disease, right ankle, sequela |
| M84.572 | Pathological fracture in neoplastic disease, left ankle |
| M84.572A | Pathological fracture in neoplastic disease, left ankle, initial encounter for fracture |
| M84.572D | Pathological fracture in neoplastic disease, left ankle, subsequent encounter for fracture with routine healing |
| M84.572G | Pathological fracture in neoplastic disease, left ankle, subsequent encounter for fracture with delayed healing |
| M84.572K | Pathological fracture in neoplastic disease, left ankle, subsequent encounter for fracture with nonunion |
| M84.572P | Pathological fracture in neoplastic disease, left ankle, subsequent encounter for fracture with malunion |
| M84.572S | Pathological fracture in neoplastic disease, left ankle, sequela |
| M84.573 | Pathological fracture in neoplastic disease, unspecified ankle |
| M84.573A | Pathological fracture in neoplastic disease, unspecified ankle, initial encounter for fracture |
| M84.573D | Pathological fracture in neoplastic disease, unspecified ankle, subsequent encounter for fracture with routine healing |
| M84.573G | Pathological fracture in neoplastic disease, unspecified ankle, subsequent encounter for fracture with delayed healing |
| M84.573K | Pathological fracture in neoplastic disease, unspecified ankle, subsequent encounter for fracture with nonunion |
| M84.573P | Pathological fracture in neoplastic disease, unspecified ankle, subsequent encounter for fracture with malunion |
| M84.573S | Pathological fracture in neoplastic disease, unspecified ankle, sequela |
| M84.574 | Pathological fracture in neoplastic disease, right foot |
| M84.574A | Pathological fracture in neoplastic disease, right foot, initial encounter for fracture |
| M84.574D | Pathological fracture in neoplastic disease, right foot, subsequent encounter for fracture with routine healing |
| M84.574G | Pathological fracture in neoplastic disease, right foot, subsequent encounter for fracture with delayed healing |
| M84.574K | Pathological fracture in neoplastic disease, right foot, subsequent encounter for fracture with nonunion |
| M84.574P | Pathological fracture in neoplastic disease, right foot, subsequent encounter for fracture with malunion |
| M84.574S | Pathological fracture in neoplastic disease, right foot, sequela |
| M84.575 | Pathological fracture in neoplastic disease, left foot |
| M84.575A | Pathological fracture in neoplastic disease, left foot, initial encounter for fracture |
| M84.575D | Pathological fracture in neoplastic disease, left foot, subsequent encounter for fracture with routine healing |
| M84.575G | Pathological fracture in neoplastic disease, left foot, subsequent encounter for fracture with delayed healing |
| M84.575K | Pathological fracture in neoplastic disease, left foot, subsequent encounter for fracture with nonunion |
| M84.575P | Pathological fracture in neoplastic disease, left foot, subsequent encounter for fracture with malunion |
| M84.575S | Pathological fracture in neoplastic disease, left foot, sequela |
| M84.576 | Pathological fracture in neoplastic disease, unspecified foot |
| M84.576A | Pathological fracture in neoplastic disease, unspecified foot, initial encounter for fracture |
| M84.576D | Pathological fracture in neoplastic disease, unspecified foot, subsequent encounter for fracture with routine healing |
| M84.576G | Pathological fracture in neoplastic disease, unspecified foot, subsequent encounter for fracture with delayed healing |
| M84.576K | Pathological fracture in neoplastic disease, unspecified foot, subsequent encounter for fracture with nonunion |
| M84.576P | Pathological fracture in neoplastic disease, unspecified foot, subsequent encounter for fracture with malunion |
| M84.576S | Pathological fracture in neoplastic disease, unspecified foot, sequela |
| M84.58 | Pathological fracture in neoplastic disease, other specified site |
| M84.58XA | Pathological fracture in neoplastic disease, other specified site, initial encounter for fracture |
| M84.58XD | Pathological fracture in neoplastic disease, other specified site, subsequent encounter for fracture with routine healing |
| M84.58XG | Pathological fracture in neoplastic disease, other specified site, subsequent encounter for fracture with delayed healing |
| M84.58XK | Pathological fracture in neoplastic disease, other specified site, subsequent encounter for fracture with nonunion |
| M84.58XP | Pathological fracture in neoplastic disease, other specified site, subsequent encounter for fracture with malunion |
| M84.58XS | Pathological fracture in neoplastic disease, other specified site, sequela |
| M84.6 | Pathological fracture in other disease |
| M84.60 | Pathological fracture in other disease, unspecified site |
| M84.60XA | Pathological fracture in other disease, unspecified site, initial encounter for fracture |
| M84.60XD | Pathological fracture in other disease, unspecified site, subsequent encounter for fracture with routine healing |
| M84.60XG | Pathological fracture in other disease, unspecified site, subsequent encounter for fracture with delayed healing |
| M84.60XK | Pathological fracture in other disease, unspecified site, subsequent encounter for fracture with nonunion |
| M84.60XP | Pathological fracture in other disease, unspecified site, subsequent encounter for fracture with malunion |
| M84.60XS | Pathological fracture in other disease, unspecified site, sequela |
| M84.61 | Pathological fracture in other disease, shoulder |
| M84.611 | Pathological fracture in other disease, right shoulder |
| M84.611A | Pathological fracture in other disease, right shoulder, initial encounter for fracture |
| M84.611D | Pathological fracture in other disease, right shoulder, subsequent encounter for fracture with routine healing |
| M84.611G | Pathological fracture in other disease, right shoulder, subsequent encounter for fracture with delayed healing |
| M84.611K | Pathological fracture in other disease, right shoulder, subsequent encounter for fracture with nonunion |
| M84.611P | Pathological fracture in other disease, right shoulder, subsequent encounter for fracture with malunion |
| M84.611S | Pathological fracture in other disease, right shoulder, sequela |
| M84.612 | Pathological fracture in other disease, left shoulder |
| M84.612A | Pathological fracture in other disease, left shoulder, initial encounter for fracture |
| M84.612D | Pathological fracture in other disease, left shoulder, subsequent encounter for fracture with routine healing |
| M84.612G | Pathological fracture in other disease, left shoulder, subsequent encounter for fracture with delayed healing |
| M84.612K | Pathological fracture in other disease, left shoulder, subsequent encounter for fracture with nonunion |
| M84.612P | Pathological fracture in other disease, left shoulder, subsequent encounter for fracture with malunion |
| M84.612S | Pathological fracture in other disease, left shoulder, sequela |
| M84.619 | Pathological fracture in other disease, unspecified shoulder |
| M84.619A | Pathological fracture in other disease, unspecified shoulder, initial encounter for fracture |
| M84.619D | Pathological fracture in other disease, unspecified shoulder, subsequent encounter for fracture with routine healing |
| M84.619G | Pathological fracture in other disease, unspecified shoulder, subsequent encounter for fracture with delayed healing |
| M84.619K | Pathological fracture in other disease, unspecified shoulder, subsequent encounter for fracture with nonunion |
| M84.619P | Pathological fracture in other disease, unspecified shoulder, subsequent encounter for fracture with malunion |
| M84.619S | Pathological fracture in other disease, unspecified shoulder, sequela |
| M84.62 | Pathological fracture in other disease, humerus |
| M84.621 | Pathological fracture in other disease, right humerus |
| M84.621A | Pathological fracture in other disease, right humerus, initial encounter for fracture |
| M84.621D | Pathological fracture in other disease, right humerus, subsequent encounter for fracture with routine healing |
| M84.621G | Pathological fracture in other disease, right humerus, subsequent encounter for fracture with delayed healing |
| M84.621K | Pathological fracture in other disease, right humerus, subsequent encounter for fracture with nonunion |
| M84.621P | Pathological fracture in other disease, right humerus, subsequent encounter for fracture with malunion |
| M84.621S | Pathological fracture in other disease, right humerus, sequela |
| M84.622 | Pathological fracture in other disease, left humerus |
| M84.622A | Pathological fracture in other disease, left humerus, initial encounter for fracture |
| M84.622D | Pathological fracture in other disease, left humerus, subsequent encounter for fracture with routine healing |
| M84.622G | Pathological fracture in other disease, left humerus, subsequent encounter for fracture with delayed healing |
| M84.622K | Pathological fracture in other disease, left humerus, subsequent encounter for fracture with nonunion |
| M84.622P | Pathological fracture in other disease, left humerus, subsequent encounter for fracture with malunion |
| M84.622S | Pathological fracture in other disease, left humerus, sequela |
| M84.629 | Pathological fracture in other disease, unspecified humerus |
| M84.629A | Pathological fracture in other disease, unspecified humerus, initial encounter for fracture |
| M84.629D | Pathological fracture in other disease, unspecified humerus, subsequent encounter for fracture with routine healing |
| M84.629G | Pathological fracture in other disease, unspecified humerus, subsequent encounter for fracture with delayed healing |
| M84.629K | Pathological fracture in other disease, unspecified humerus, subsequent encounter for fracture with nonunion |
| M84.629P | Pathological fracture in other disease, unspecified humerus, subsequent encounter for fracture with malunion |
| M84.629S | Pathological fracture in other disease, unspecified humerus, sequela |
| M84.63 | Pathological fracture in other disease, ulna and radius |
| M84.631 | Pathological fracture in other disease, right ulna |
| M84.631A | Pathological fracture in other disease, right ulna, initial encounter for fracture |
| M84.631D | Pathological fracture in other disease, right ulna, subsequent encounter for fracture with routine healing |
| M84.631G | Pathological fracture in other disease, right ulna, subsequent encounter for fracture with delayed healing |
| M84.631K | Pathological fracture in other disease, right ulna, subsequent encounter for fracture with nonunion |
| M84.631P | Pathological fracture in other disease, right ulna, subsequent encounter for fracture with malunion |
| M84.631S | Pathological fracture in other disease, right ulna, sequela |
| M84.632 | Pathological fracture in other disease, left ulna |
| M84.632A | Pathological fracture in other disease, left ulna, initial encounter for fracture |
| M84.632D | Pathological fracture in other disease, left ulna, subsequent encounter for fracture with routine healing |
| M84.632G | Pathological fracture in other disease, left ulna, subsequent encounter for fracture with delayed healing |
| M84.632K | Pathological fracture in other disease, left ulna, subsequent encounter for fracture with nonunion |
| M84.632P | Pathological fracture in other disease, left ulna, subsequent encounter for fracture with malunion |
| M84.632S | Pathological fracture in other disease, left ulna, sequela |
| M84.633 | Pathological fracture in other disease, right radius |
| M84.633A | Pathological fracture in other disease, right radius, initial encounter for fracture |
| M84.633D | Pathological fracture in other disease, right radius, subsequent encounter for fracture with routine healing |
| M84.633G | Pathological fracture in other disease, right radius, subsequent encounter for fracture with delayed healing |
| M84.633K | Pathological fracture in other disease, right radius, subsequent encounter for fracture with nonunion |
| M84.633P | Pathological fracture in other disease, right radius, subsequent encounter for fracture with malunion |
| M84.633S | Pathological fracture in other disease, right radius, sequela |
| M84.634 | Pathological fracture in other disease, left radius |
| M84.634A | Pathological fracture in other disease, left radius, initial encounter for fracture |
| M84.634D | Pathological fracture in other disease, left radius, subsequent encounter for fracture with routine healing |
| M84.634G | Pathological fracture in other disease, left radius, subsequent encounter for fracture with delayed healing |
| M84.634K | Pathological fracture in other disease, left radius, subsequent encounter for fracture with nonunion |
| M84.634P | Pathological fracture in other disease, left radius, subsequent encounter for fracture with malunion |
| M84.634S | Pathological fracture in other disease, left radius, sequela |
| M84.639 | Pathological fracture in other disease, unspecified ulna and radius |
| M84.639A | Pathological fracture in other disease, unspecified ulna and radius, initial encounter for fracture |
| M84.639D | Pathological fracture in other disease, unspecified ulna and radius, subsequent encounter for fracture with routine healing |
| M84.639G | Pathological fracture in other disease, unspecified ulna and radius, subsequent encounter for fracture with delayed healing |
| M84.639K | Pathological fracture in other disease, unspecified ulna and radius, subsequent encounter for fracture with nonunion |
| M84.639P | Pathological fracture in other disease, unspecified ulna and radius, subsequent encounter for fracture with malunion |
| M84.639S | Pathological fracture in other disease, unspecified ulna and radius, sequela |
| M84.64 | Pathological fracture in other disease, hand |
| M84.641 | Pathological fracture in other disease, right hand |
| M84.641A | Pathological fracture in other disease, right hand, initial encounter for fracture |
| M84.641D | Pathological fracture in other disease, right hand, subsequent encounter for fracture with routine healing |
| M84.641G | Pathological fracture in other disease, right hand, subsequent encounter for fracture with delayed healing |
| M84.641K | Pathological fracture in other disease, right hand, subsequent encounter for fracture with nonunion |
| M84.641P | Pathological fracture in other disease, right hand, subsequent encounter for fracture with malunion |
| M84.641S | Pathological fracture in other disease, right hand, sequela |
| M84.642 | Pathological fracture in other disease, left hand |
| M84.642A | Pathological fracture in other disease, left hand, initial encounter for fracture |
| M84.642D | Pathological fracture in other disease, left hand, subsequent encounter for fracture with routine healing |
| M84.642G | Pathological fracture in other disease, left hand, subsequent encounter for fracture with delayed healing |
| M84.642K | Pathological fracture in other disease, left hand, subsequent encounter for fracture with nonunion |
| M84.642P | Pathological fracture in other disease, left hand, subsequent encounter for fracture with malunion |
| M84.642S | Pathological fracture in other disease, left hand, sequela |
| M84.649 | Pathological fracture in other disease, unspecified hand |
| M84.649A | Pathological fracture in other disease, unspecified hand, initial encounter for fracture |
| M84.649D | Pathological fracture in other disease, unspecified hand, subsequent encounter for fracture with routine healing |
| M84.649G | Pathological fracture in other disease, unspecified hand, subsequent encounter for fracture with delayed healing |
| M84.649K | Pathological fracture in other disease, unspecified hand, subsequent encounter for fracture with nonunion |
| M84.649P | Pathological fracture in other disease, unspecified hand, subsequent encounter for fracture with malunion |
| M84.649S | Pathological fracture in other disease, unspecified hand, sequela |
| M84.65 | Pathological fracture in other disease, pelvis and femur |
| M84.650 | Pathological fracture in other disease, pelvis |
| M84.650A | Pathological fracture in other disease, pelvis, initial encounter for fracture |
| M84.650D | Pathological fracture in other disease, pelvis, subsequent encounter for fracture with routine healing |
| M84.650G | Pathological fracture in other disease, pelvis, subsequent encounter for fracture with delayed healing |
| M84.650K | Pathological fracture in other disease, pelvis, subsequent encounter for fracture with nonunion |
| M84.650P | Pathological fracture in other disease, pelvis, subsequent encounter for fracture with malunion |
| M84.650S | Pathological fracture in other disease, pelvis, sequela |
| M84.651 | Pathological fracture in other disease, right femur |
| M84.651A | Pathological fracture in other disease, right femur, initial encounter for fracture |
| M84.651D | Pathological fracture in other disease, right femur, subsequent encounter for fracture with routine healing |
| M84.651G | Pathological fracture in other disease, right femur, subsequent encounter for fracture with delayed healing |
| M84.651K | Pathological fracture in other disease, right femur, subsequent encounter for fracture with nonunion |
| M84.651P | Pathological fracture in other disease, right femur, subsequent encounter for fracture with malunion |
| M84.651S | Pathological fracture in other disease, right femur, sequela |
| M84.652 | Pathological fracture in other disease, left femur |
| M84.652A | Pathological fracture in other disease, left femur, initial encounter for fracture |
| M84.652D | Pathological fracture in other disease, left femur, subsequent encounter for fracture with routine healing |
| M84.652G | Pathological fracture in other disease, left femur, subsequent encounter for fracture with delayed healing |
| M84.652K | Pathological fracture in other disease, left femur, subsequent encounter for fracture with nonunion |
| M84.652P | Pathological fracture in other disease, left femur, subsequent encounter for fracture with malunion |
| M84.652S | Pathological fracture in other disease, left femur, sequela |
| M84.653 | Pathological fracture in other disease, unspecified femur |
| M84.653A | Pathological fracture in other disease, unspecified femur, initial encounter for fracture |
| M84.653D | Pathological fracture in other disease, unspecified femur, subsequent encounter for fracture with routine healing |
| M84.653G | Pathological fracture in other disease, unspecified femur, subsequent encounter for fracture with delayed healing |
| M84.653K | Pathological fracture in other disease, unspecified femur, subsequent encounter for fracture with nonunion |
| M84.653P | Pathological fracture in other disease, unspecified femur, subsequent encounter for fracture with malunion |
| M84.653S | Pathological fracture in other disease, unspecified femur, sequela |
| M84.659 | Pathological fracture in other disease, hip, unspecified |
| M84.659A | Pathological fracture in other disease, hip, unspecified, initial encounter for fracture |
| M84.659D | Pathological fracture in other disease, hip, unspecified, subsequent encounter for fracture with routine healing |
| M84.659G | Pathological fracture in other disease, hip, unspecified, subsequent encounter for fracture with delayed healing |
| M84.659K | Pathological fracture in other disease, hip, unspecified, subsequent encounter for fracture with nonunion |
| M84.659P | Pathological fracture in other disease, hip, unspecified, subsequent encounter for fracture with malunion |
| M84.659S | Pathological fracture in other disease, hip, unspecified, sequela |
| M84.66 | Pathological fracture in other disease, tibia and fibula |
| M84.661 | Pathological fracture in other disease, right tibia |
| M84.661A | Pathological fracture in other disease, right tibia, initial encounter for fracture |
| M84.661D | Pathological fracture in other disease, right tibia, subsequent encounter for fracture with routine healing |
| M84.661G | Pathological fracture in other disease, right tibia, subsequent encounter for fracture with delayed healing |
| M84.661K | Pathological fracture in other disease, right tibia, subsequent encounter for fracture with nonunion |
| M84.661P | Pathological fracture in other disease, right tibia, subsequent encounter for fracture with malunion |
| M84.661S | Pathological fracture in other disease, right tibia, sequela |
| M84.662 | Pathological fracture in other disease, left tibia |
| M84.662A | Pathological fracture in other disease, left tibia, initial encounter for fracture |
| M84.662D | Pathological fracture in other disease, left tibia, subsequent encounter for fracture with routine healing |
| M84.662G | Pathological fracture in other disease, left tibia, subsequent encounter for fracture with delayed healing |
| M84.662K | Pathological fracture in other disease, left tibia, subsequent encounter for fracture with nonunion |
| M84.662P | Pathological fracture in other disease, left tibia, subsequent encounter for fracture with malunion |
| M84.662S | Pathological fracture in other disease, left tibia, sequela |
| M84.663 | Pathological fracture in other disease, right fibula |
| M84.663A | Pathological fracture in other disease, right fibula, initial encounter for fracture |
| M84.663D | Pathological fracture in other disease, right fibula, subsequent encounter for fracture with routine healing |
| M84.663G | Pathological fracture in other disease, right fibula, subsequent encounter for fracture with delayed healing |
| M84.663K | Pathological fracture in other disease, right fibula, subsequent encounter for fracture with nonunion |
| M84.663P | Pathological fracture in other disease, right fibula, subsequent encounter for fracture with malunion |
| M84.663S | Pathological fracture in other disease, right fibula, sequela |
| M84.664 | Pathological fracture in other disease, left fibula |
| M84.664A | Pathological fracture in other disease, left fibula, initial encounter for fracture |
| M84.664D | Pathological fracture in other disease, left fibula, subsequent encounter for fracture with routine healing |
| M84.664G | Pathological fracture in other disease, left fibula, subsequent encounter for fracture with delayed healing |
| M84.664K | Pathological fracture in other disease, left fibula, subsequent encounter for fracture with nonunion |
| M84.664P | Pathological fracture in other disease, left fibula, subsequent encounter for fracture with malunion |
| M84.664S | Pathological fracture in other disease, left fibula, sequela |
| M84.669 | Pathological fracture in other disease, unspecified tibia and fibula |
| M84.669A | Pathological fracture in other disease, unspecified tibia and fibula, initial encounter for fracture |
| M84.669D | Pathological fracture in other disease, unspecified tibia and fibula, subsequent encounter for fracture with routine healing |
| M84.669G | Pathological fracture in other disease, unspecified tibia and fibula, subsequent encounter for fracture with delayed healing |
| M84.669K | Pathological fracture in other disease, unspecified tibia and fibula, subsequent encounter for fracture with nonunion |
| M84.669P | Pathological fracture in other disease, unspecified tibia and fibula, subsequent encounter for fracture with malunion |
| M84.669S | Pathological fracture in other disease, unspecified tibia and fibula, sequela |
| M84.67 | Pathological fracture in other disease, ankle and foot |
| M84.671 | Pathological fracture in other disease, right ankle |
| M84.671A | Pathological fracture in other disease, right ankle, initial encounter for fracture |
| M84.671D | Pathological fracture in other disease, right ankle, subsequent encounter for fracture with routine healing |
| M84.671G | Pathological fracture in other disease, right ankle, subsequent encounter for fracture with delayed healing |
| M84.671K | Pathological fracture in other disease, right ankle, subsequent encounter for fracture with nonunion |
| M84.671P | Pathological fracture in other disease, right ankle, subsequent encounter for fracture with malunion |
| M84.671S | Pathological fracture in other disease, right ankle, sequela |
| M84.672 | Pathological fracture in other disease, left ankle |
| M84.672A | Pathological fracture in other disease, left ankle, initial encounter for fracture |
| M84.672D | Pathological fracture in other disease, left ankle, subsequent encounter for fracture with routine healing |
| M84.672G | Pathological fracture in other disease, left ankle, subsequent encounter for fracture with delayed healing |
| M84.672K | Pathological fracture in other disease, left ankle, subsequent encounter for fracture with nonunion |
| M84.672P | Pathological fracture in other disease, left ankle, subsequent encounter for fracture with malunion |
| M84.672S | Pathological fracture in other disease, left ankle, sequela |
| M84.673 | Pathological fracture in other disease, unspecified ankle |
| M84.673A | Pathological fracture in other disease, unspecified ankle, initial encounter for fracture |
| M84.673D | Pathological fracture in other disease, unspecified ankle, subsequent encounter for fracture with routine healing |
| M84.673G | Pathological fracture in other disease, unspecified ankle, subsequent encounter for fracture with delayed healing |
| M84.673K | Pathological fracture in other disease, unspecified ankle, subsequent encounter for fracture with nonunion |
| M84.673P | Pathological fracture in other disease, unspecified ankle, subsequent encounter for fracture with malunion |
| M84.673S | Pathological fracture in other disease, unspecified ankle, sequela |
| M84.674 | Pathological fracture in other disease, right foot |
| M84.674A | Pathological fracture in other disease, right foot, initial encounter for fracture |
| M84.674D | Pathological fracture in other disease, right foot, subsequent encounter for fracture with routine healing |
| M84.674G | Pathological fracture in other disease, right foot, subsequent encounter for fracture with delayed healing |
| M84.674K | Pathological fracture in other disease, right foot, subsequent encounter for fracture with nonunion |
| M84.674P | Pathological fracture in other disease, right foot, subsequent encounter for fracture with malunion |
| M84.674S | Pathological fracture in other disease, right foot, sequela |
| M84.675 | Pathological fracture in other disease, left foot |
| M84.675A | Pathological fracture in other disease, left foot, initial encounter for fracture |
| M84.675D | Pathological fracture in other disease, left foot, subsequent encounter for fracture with routine healing |
| M84.675G | Pathological fracture in other disease, left foot, subsequent encounter for fracture with delayed healing |
| M84.675K | Pathological fracture in other disease, left foot, subsequent encounter for fracture with nonunion |
| M84.675P | Pathological fracture in other disease, left foot, subsequent encounter for fracture with malunion |
| M84.675S | Pathological fracture in other disease, left foot, sequela |
| M84.676 | Pathological fracture in other disease, unspecified foot |
| M84.676A | Pathological fracture in other disease, unspecified foot, initial encounter for fracture |
| M84.676D | Pathological fracture in other disease, unspecified foot, subsequent encounter for fracture with routine healing |
| M84.676G | Pathological fracture in other disease, unspecified foot, subsequent encounter for fracture with delayed healing |
| M84.676K | Pathological fracture in other disease, unspecified foot, subsequent encounter for fracture with nonunion |
| M84.676P | Pathological fracture in other disease, unspecified foot, subsequent encounter for fracture with malunion |
| M84.676S | Pathological fracture in other disease, unspecified foot, sequela |
| M84.68 | Pathological fracture in other disease, other site |
| M84.68XA | Pathological fracture in other disease, other site, initial encounter for fracture |
| M84.68XD | Pathological fracture in other disease, other site, subsequent encounter for fracture with routine healing |
| M84.68XG | Pathological fracture in other disease, other site, subsequent encounter for fracture with delayed healing |
| M84.68XK | Pathological fracture in other disease, other site, subsequent encounter for fracture with nonunion |
| M84.68XP | Pathological fracture in other disease, other site, subsequent encounter for fracture with malunion |
| M84.68XS | Pathological fracture in other disease, other site, sequela |
| M84.7 | Nontraumatic fracture, not elsewhere classified |
| M84.75 | Atypical femoral fracture |
| M84.750 | Atypical femoral fracture, unspecified |
| M84.750A | Atypical femoral fracture, unspecified, initial encounter for fracture |
| M84.750D | Atypical femoral fracture, unspecified, subsequent encounter for fracture with routine healing |
| M84.750G | Atypical femoral fracture, unspecified, subsequent encounter for fracture with delayed healing |
| M84.750K | Atypical femoral fracture, unspecified, subsequent encounter for fracture with nonunion |
| M84.750P | Atypical femoral fracture, unspecified, subsequent encounter for fracture with malunion |
| M84.750S | Atypical femoral fracture, unspecified, sequela |
| M84.751 | Incomplete atypical femoral fracture, right leg |
| M84.751A | Incomplete atypical femoral fracture, right leg, initial encounter for fracture |
| M84.751D | Incomplete atypical femoral fracture, right leg, subsequent encounter for fracture with routine healing |
| M84.751G | Incomplete atypical femoral fracture, right leg, subsequent encounter for fracture with delayed healing |
| M84.751K | Incomplete atypical femoral fracture, right leg, subsequent encounter for fracture with nonunion |
| M84.751P | Incomplete atypical femoral fracture, right leg, subsequent encounter for fracture with malunion |
| M84.751S | Incomplete atypical femoral fracture, right leg, sequela |
| M84.752 | Incomplete atypical femoral fracture, left leg |
| M84.752A | Incomplete atypical femoral fracture, left leg, initial encounter for fracture |
| M84.752D | Incomplete atypical femoral fracture, left leg, subsequent encounter for fracture with routine healing |
| M84.752G | Incomplete atypical femoral fracture, left leg, subsequent encounter for fracture with delayed healing |
| M84.752K | Incomplete atypical femoral fracture, left leg, subsequent encounter for fracture with nonunion |
| M84.752P | Incomplete atypical femoral fracture, left leg, subsequent encounter for fracture with malunion |
| M84.752S | Incomplete atypical femoral fracture, left leg, sequela |
| M84.753 | Incomplete atypical femoral fracture, unspecified leg |
| M84.753A | Incomplete atypical femoral fracture, unspecified leg, initial encounter for fracture |
| M84.753D | Incomplete atypical femoral fracture, unspecified leg, subsequent encounter for fracture with routine healing |
| M84.753G | Incomplete atypical femoral fracture, unspecified leg, subsequent encounter for fracture with delayed healing |
| M84.753K | Incomplete atypical femoral fracture, unspecified leg, subsequent encounter for fracture with nonunion |
| M84.753P | Incomplete atypical femoral fracture, unspecified leg, subsequent encounter for fracture with malunion |
| M84.753S | Incomplete atypical femoral fracture, unspecified leg, sequela |
| M84.754 | Complete transverse atypical femoral fracture, right leg |
| M84.754A | Complete transverse atypical femoral fracture, right leg, initial encounter for fracture |
| M84.754D | Complete transverse atypical femoral fracture, right leg, subsequent encounter for fracture with routine healing |
| M84.754G | Complete transverse atypical femoral fracture, right leg, subsequent encounter for fracture with delayed healing |
| M84.754K | Complete transverse atypical femoral fracture, right leg, subsequent encounter for fracture with nonunion |
| M84.754P | Complete transverse atypical femoral fracture, right leg, subsequent encounter for fracture with malunion |
| M84.754S | Complete transverse atypical femoral fracture, right leg, sequela |
| M84.755 | Complete transverse atypical femoral fracture, left leg |
| M84.755A | Complete transverse atypical femoral fracture, left leg, initial encounter for fracture |
| M84.755D | Complete transverse atypical femoral fracture, left leg, subsequent encounter for fracture with routine healing |
| M84.755G | Complete transverse atypical femoral fracture, left leg, subsequent encounter for fracture with delayed healing |
| M84.755K | Complete transverse atypical femoral fracture, left leg, subsequent encounter for fracture with nonunion |
| M84.755P | Complete transverse atypical femoral fracture, left leg, subsequent encounter for fracture with malunion |
| M84.755S | Complete transverse atypical femoral fracture, left leg, sequela |
| M84.756 | Complete transverse atypical femoral fracture, unspecified leg |
| M84.756A | Complete transverse atypical femoral fracture, unspecified leg, initial encounter for fracture |
| M84.756D | Complete transverse atypical femoral fracture, unspecified leg, subsequent encounter for fracture with routine healing |
| M84.756G | Complete transverse atypical femoral fracture, unspecified leg, subsequent encounter for fracture with delayed healing |
| M84.756K | Complete transverse atypical femoral fracture, unspecified leg, subsequent encounter for fracture with nonunion |
| M84.756P | Complete transverse atypical femoral fracture, unspecified leg, subsequent encounter for fracture with malunion |
| M84.756S | Complete transverse atypical femoral fracture, unspecified leg, sequela |
| M84.757 | Complete oblique atypical femoral fracture, right leg |
| M84.757A | Complete oblique atypical femoral fracture, right leg, initial encounter for fracture |
| M84.757D | Complete oblique atypical femoral fracture, right leg, subsequent encounter for fracture with routine healing |
| M84.757G | Complete oblique atypical femoral fracture, right leg, subsequent encounter for fracture with delayed healing |
| M84.757K | Complete oblique atypical femoral fracture, right leg, subsequent encounter for fracture with nonunion |
| M84.757P | Complete oblique atypical femoral fracture, right leg, subsequent encounter for fracture with malunion |
| M84.757S | Complete oblique atypical femoral fracture, right leg, sequela |
| M84.758 | Complete oblique atypical femoral fracture, left leg |
| M84.758A | Complete oblique atypical femoral fracture, left leg, initial encounter for fracture |
| M84.758D | Complete oblique atypical femoral fracture, left leg, subsequent encounter for fracture with routine healing |
| M84.758G | Complete oblique atypical femoral fracture, left leg, subsequent encounter for fracture with delayed healing |
| M84.758K | Complete oblique atypical femoral fracture, left leg, subsequent encounter for fracture with nonunion |
| M84.758P | Complete oblique atypical femoral fracture, left leg, subsequent encounter for fracture with malunion |
| M84.758S | Complete oblique atypical femoral fracture, left leg, sequela |
| M84.759 | Complete oblique atypical femoral fracture, unspecified leg |
| M84.759A | Complete oblique atypical femoral fracture, unspecified leg, initial encounter for fracture |
| M84.759D | Complete oblique atypical femoral fracture, unspecified leg, subsequent encounter for fracture with routine healing |
| M84.759G | Complete oblique atypical femoral fracture, unspecified leg, subsequent encounter for fracture with delayed healing |
| M84.759K | Complete oblique atypical femoral fracture, unspecified leg, subsequent encounter for fracture with nonunion |
| M84.759P | Complete oblique atypical femoral fracture, unspecified leg, subsequent encounter for fracture with malunion |
| M84.759S | Complete oblique atypical femoral fracture, unspecified leg, sequela |
| M84.8 | Other disorders of continuity of bone |
| M84.80 | Other disorders of continuity of bone, unspecified site |
| M84.81 | Other disorders of continuity of bone, shoulder |
| M84.811 | Other disorders of continuity of bone, right shoulder |
| M84.812 | Other disorders of continuity of bone, left shoulder |
| M84.819 | Other disorders of continuity of bone, unspecified shoulder |
| M84.82 | Other disorders of continuity of bone, humerus |
| M84.821 | Other disorders of continuity of bone, right humerus |
| M84.822 | Other disorders of continuity of bone, left humerus |
| M84.829 | Other disorders of continuity of bone, unspecified humerus |
| M84.83 | Other disorders of continuity of bone, ulna and radius |
| M84.831 | Other disorders of continuity of bone, right ulna |
| M84.832 | Other disorders of continuity of bone, left ulna |
| M84.833 | Other disorders of continuity of bone, right radius |
| M84.834 | Other disorders of continuity of bone, left radius |
| M84.839 | Other disorders of continuity of bone, unspecified ulna and radius |
| M84.84 | Other disorders of continuity of bone, hand |
| M84.841 | Other disorders of continuity of bone, right hand |
| M84.842 | Other disorders of continuity of bone, left hand |
| M84.849 | Other disorders of continuity of bone, unspecified hand |
| M84.85 | Other disorders of continuity of bone, pelvic region and thigh |
| M84.851 | Other disorders of continuity of bone, right pelvic region and thigh |
| M84.852 | Other disorders of continuity of bone, left pelvic region and thigh |
| M84.859 | Other disorders of continuity of bone, unspecified pelvic region and thigh |
| M84.86 | Other disorders of continuity of bone, tibia and fibula |
| M84.861 | Other disorders of continuity of bone, right tibia |
| M84.862 | Other disorders of continuity of bone, left tibia |
| M84.863 | Other disorders of continuity of bone, right fibula |
| M84.864 | Other disorders of continuity of bone, left fibula |
| M84.869 | Other disorders of continuity of bone, unspecified tibia and fibula |
| M84.87 | Other disorders of continuity of bone, ankle and foot |
| M84.871 | Other disorders of continuity of bone, right ankle and foot |
| M84.872 | Other disorders of continuity of bone, left ankle and foot |
| M84.879 | Other disorders of continuity of bone, unspecified ankle and foot |
| M84.88 | Other disorders of continuity of bone, other site |
| M84.9 | Disorder of continuity of bone, unspecified |
| M85 | Other disorders of bone density and structure |
| M85.0 | Fibrous dysplasia (monostotic) |
| M85.00 | Fibrous dysplasia (monostotic), unspecified site |
| M85.01 | Fibrous dysplasia (monostotic), shoulder |
| M85.011 | Fibrous dysplasia (monostotic), right shoulder |
| M85.012 | Fibrous dysplasia (monostotic), left shoulder |
| M85.019 | Fibrous dysplasia (monostotic), unspecified shoulder |
| M85.02 | Fibrous dysplasia (monostotic), upper arm |
| M85.021 | Fibrous dysplasia (monostotic), right upper arm |
| M85.022 | Fibrous dysplasia (monostotic), left upper arm |
| M85.029 | Fibrous dysplasia (monostotic), unspecified upper arm |
| M85.03 | Fibrous dysplasia (monostotic), forearm |
| M85.031 | Fibrous dysplasia (monostotic), right forearm |
| M85.032 | Fibrous dysplasia (monostotic), left forearm |
| M85.039 | Fibrous dysplasia (monostotic), unspecified forearm |
| M85.04 | Fibrous dysplasia (monostotic), hand |
| M85.041 | Fibrous dysplasia (monostotic), right hand |
| M85.042 | Fibrous dysplasia (monostotic), left hand |
| M85.049 | Fibrous dysplasia (monostotic), unspecified hand |
| M85.05 | Fibrous dysplasia (monostotic), thigh |
| M85.051 | Fibrous dysplasia (monostotic), right thigh |
| M85.052 | Fibrous dysplasia (monostotic), left thigh |
| M85.059 | Fibrous dysplasia (monostotic), unspecified thigh |
| M85.06 | Fibrous dysplasia (monostotic), lower leg |
| M85.061 | Fibrous dysplasia (monostotic), right lower leg |
| M85.062 | Fibrous dysplasia (monostotic), left lower leg |
| M85.069 | Fibrous dysplasia (monostotic), unspecified lower leg |
| M85.07 | Fibrous dysplasia (monostotic), ankle and foot |
| M85.071 | Fibrous dysplasia (monostotic), right ankle and foot |
| M85.072 | Fibrous dysplasia (monostotic), left ankle and foot |
| M85.079 | Fibrous dysplasia (monostotic), unspecified ankle and foot |
| M85.08 | Fibrous dysplasia (monostotic), other site |
| M85.09 | Fibrous dysplasia (monostotic), multiple sites |
| M85.1 | Skeletal fluorosis |
| M85.10 | Skeletal fluorosis, unspecified site |
| M85.11 | Skeletal fluorosis, shoulder |
| M85.111 | Skeletal fluorosis, right shoulder |
| M85.112 | Skeletal fluorosis, left shoulder |
| M85.119 | Skeletal fluorosis, unspecified shoulder |
| M85.12 | Skeletal fluorosis, upper arm |
| M85.121 | Skeletal fluorosis, right upper arm |
| M85.122 | Skeletal fluorosis, left upper arm |
| M85.129 | Skeletal fluorosis, unspecified upper arm |
| M85.13 | Skeletal fluorosis, forearm |
| M85.131 | Skeletal fluorosis, right forearm |
| M85.132 | Skeletal fluorosis, left forearm |
| M85.139 | Skeletal fluorosis, unspecified forearm |
| M85.14 | Skeletal fluorosis, hand |
| M85.141 | Skeletal fluorosis, right hand |
| M85.142 | Skeletal fluorosis, left hand |
| M85.149 | Skeletal fluorosis, unspecified hand |
| M85.15 | Skeletal fluorosis, thigh |
| M85.151 | Skeletal fluorosis, right thigh |
| M85.152 | Skeletal fluorosis, left thigh |
| M85.159 | Skeletal fluorosis, unspecified thigh |
| M85.16 | Skeletal fluorosis, lower leg |
| M85.161 | Skeletal fluorosis, right lower leg |
| M85.162 | Skeletal fluorosis, left lower leg |
| M85.169 | Skeletal fluorosis, unspecified lower leg |
| M85.17 | Skeletal fluorosis, ankle and foot |
| M85.171 | Skeletal fluorosis, right ankle and foot |
| M85.172 | Skeletal fluorosis, left ankle and foot |
| M85.179 | Skeletal fluorosis, unspecified ankle and foot |
| M85.18 | Skeletal fluorosis, other site |
| M85.19 | Skeletal fluorosis, multiple sites |
| M85.2 | Hyperostosis of skull |
| M85.3 | Osteitis condensans |
| M85.30 | Osteitis condensans, unspecified site |
| M85.31 | Osteitis condensans, shoulder |
| M85.311 | Osteitis condensans, right shoulder |
| M85.312 | Osteitis condensans, left shoulder |
| M85.319 | Osteitis condensans, unspecified shoulder |
| M85.32 | Osteitis condensans, upper arm |
| M85.321 | Osteitis condensans, right upper arm |
| M85.322 | Osteitis condensans, left upper arm |
| M85.329 | Osteitis condensans, unspecified upper arm |
| M85.33 | Osteitis condensans, forearm |
| M85.331 | Osteitis condensans, right forearm |
| M85.332 | Osteitis condensans, left forearm |
| M85.339 | Osteitis condensans, unspecified forearm |
| M85.34 | Osteitis condensans, hand |
| M85.341 | Osteitis condensans, right hand |
| M85.342 | Osteitis condensans, left hand |
| M85.349 | Osteitis condensans, unspecified hand |
| M85.35 | Osteitis condensans, thigh |
| M85.351 | Osteitis condensans, right thigh |
| M85.352 | Osteitis condensans, left thigh |
| M85.359 | Osteitis condensans, unspecified thigh |
| M85.36 | Osteitis condensans, lower leg |
| M85.361 | Osteitis condensans, right lower leg |
| M85.362 | Osteitis condensans, left lower leg |
| M85.369 | Osteitis condensans, unspecified lower leg |
| M85.37 | Osteitis condensans, ankle and foot |
| M85.371 | Osteitis condensans, right ankle and foot |
| M85.372 | Osteitis condensans, left ankle and foot |
| M85.379 | Osteitis condensans, unspecified ankle and foot |
| M85.38 | Osteitis condensans, other site |
| M85.39 | Osteitis condensans, multiple sites |
| M85.4 | Solitary bone cyst |
| M85.40 | Solitary bone cyst, unspecified site |
| M85.41 | Solitary bone cyst, shoulder |
| M85.411 | Solitary bone cyst, right shoulder |
| M85.412 | Solitary bone cyst, left shoulder |
| M85.419 | Solitary bone cyst, unspecified shoulder |
| M85.42 | Solitary bone cyst, humerus |
| M85.421 | Solitary bone cyst, right humerus |
| M85.422 | Solitary bone cyst, left humerus |
| M85.429 | Solitary bone cyst, unspecified humerus |
| M85.43 | Solitary bone cyst, ulna and radius |
| M85.431 | Solitary bone cyst, right ulna and radius |
| M85.432 | Solitary bone cyst, left ulna and radius |
| M85.439 | Solitary bone cyst, unspecified ulna and radius |
| M85.44 | Solitary bone cyst, hand |
| M85.441 | Solitary bone cyst, right hand |
| M85.442 | Solitary bone cyst, left hand |
| M85.449 | Solitary bone cyst, unspecified hand |
| M85.45 | Solitary bone cyst, pelvis |
| M85.451 | Solitary bone cyst, right pelvis |
| M85.452 | Solitary bone cyst, left pelvis |
| M85.459 | Solitary bone cyst, unspecified pelvis |
| M85.46 | Solitary bone cyst, tibia and fibula |
| M85.461 | Solitary bone cyst, right tibia and fibula |
| M85.462 | Solitary bone cyst, left tibia and fibula |
| M85.469 | Solitary bone cyst, unspecified tibia and fibula |
| M85.47 | Solitary bone cyst, ankle and foot |
| M85.471 | Solitary bone cyst, right ankle and foot |
| M85.472 | Solitary bone cyst, left ankle and foot |
| M85.479 | Solitary bone cyst, unspecified ankle and foot |
| M85.48 | Solitary bone cyst, other site |
| M85.5 | Aneurysmal bone cyst |
| M85.50 | Aneurysmal bone cyst, unspecified site |
| M85.51 | Aneurysmal bone cyst, shoulder |
| M85.511 | Aneurysmal bone cyst, right shoulder |
| M85.512 | Aneurysmal bone cyst, left shoulder |
| M85.519 | Aneurysmal bone cyst, unspecified shoulder |
| M85.52 | Aneurysmal bone cyst, upper arm |
| M85.521 | Aneurysmal bone cyst, right upper arm |
| M85.522 | Aneurysmal bone cyst, left upper arm |
| M85.529 | Aneurysmal bone cyst, unspecified upper arm |
| M85.53 | Aneurysmal bone cyst, forearm |
| M85.531 | Aneurysmal bone cyst, right forearm |
| M85.532 | Aneurysmal bone cyst, left forearm |
| M85.539 | Aneurysmal bone cyst, unspecified forearm |
| M85.54 | Aneurysmal bone cyst, hand |
| M85.541 | Aneurysmal bone cyst, right hand |
| M85.542 | Aneurysmal bone cyst, left hand |
| M85.549 | Aneurysmal bone cyst, unspecified hand |
| M85.55 | Aneurysmal bone cyst, thigh |
| M85.551 | Aneurysmal bone cyst, right thigh |
| M85.552 | Aneurysmal bone cyst, left thigh |
| M85.559 | Aneurysmal bone cyst, unspecified thigh |
| M85.56 | Aneurysmal bone cyst, lower leg |
| M85.561 | Aneurysmal bone cyst, right lower leg |
| M85.562 | Aneurysmal bone cyst, left lower leg |
| M85.569 | Aneurysmal bone cyst, unspecified lower leg |
| M85.57 | Aneurysmal bone cyst, ankle and foot |
| M85.571 | Aneurysmal bone cyst, right ankle and foot |
| M85.572 | Aneurysmal bone cyst, left ankle and foot |
| M85.579 | Aneurysmal bone cyst, unspecified ankle and foot |
| M85.58 | Aneurysmal bone cyst, other site |
| M85.59 | Aneurysmal bone cyst, multiple sites |
| M85.6 | Other cyst of bone |
| M85.60 | Other cyst of bone, unspecified site |
| M85.61 | Other cyst of bone, shoulder |
| M85.611 | Other cyst of bone, right shoulder |
| M85.612 | Other cyst of bone, left shoulder |
| M85.619 | Other cyst of bone, unspecified shoulder |
| M85.62 | Other cyst of bone, upper arm |
| M85.621 | Other cyst of bone, right upper arm |
| M85.622 | Other cyst of bone, left upper arm |
| M85.629 | Other cyst of bone, unspecified upper arm |
| M85.63 | Other cyst of bone, forearm |
| M85.631 | Other cyst of bone, right forearm |
| M85.632 | Other cyst of bone, left forearm |
| M85.639 | Other cyst of bone, unspecified forearm |
| M85.64 | Other cyst of bone, hand |
| M85.641 | Other cyst of bone, right hand |
| M85.642 | Other cyst of bone, left hand |
| M85.649 | Other cyst of bone, unspecified hand |
| M85.65 | Other cyst of bone, thigh |
| M85.651 | Other cyst of bone, right thigh |
| M85.652 | Other cyst of bone, left thigh |
| M85.659 | Other cyst of bone, unspecified thigh |
| M85.66 | Other cyst of bone, lower leg |
| M85.661 | Other cyst of bone, right lower leg |
| M85.662 | Other cyst of bone, left lower leg |
| M85.669 | Other cyst of bone, unspecified lower leg |
| M85.67 | Other cyst of bone, ankle and foot |
| M85.671 | Other cyst of bone, right ankle and foot |
| M85.672 | Other cyst of bone, left ankle and foot |
| M85.679 | Other cyst of bone, unspecified ankle and foot |
| M85.68 | Other cyst of bone, other site |
| M85.69 | Other cyst of bone, multiple sites |
| M85.8 | Other specified disorders of bone density and structure |
| M85.80 | Other specified disorders of bone density and structure, unspecified site |
| M85.81 | Other specified disorders of bone density and structure, shoulder |
| M85.811 | Other specified disorders of bone density and structure, right shoulder |
| M85.812 | Other specified disorders of bone density and structure, left shoulder |
| M85.819 | Other specified disorders of bone density and structure, unspecified shoulder |
| M85.82 | Other specified disorders of bone density and structure, upper arm |
| M85.821 | Other specified disorders of bone density and structure, right upper arm |
| M85.822 | Other specified disorders of bone density and structure, left upper arm |
| M85.829 | Other specified disorders of bone density and structure, unspecified upper arm |
| M85.83 | Other specified disorders of bone density and structure, forearm |
| M85.831 | Other specified disorders of bone density and structure, right forearm |
| M85.832 | Other specified disorders of bone density and structure, left forearm |
| M85.839 | Other specified disorders of bone density and structure, unspecified forearm |
| M85.84 | Other specified disorders of bone density and structure, hand |
| M85.841 | Other specified disorders of bone density and structure, right hand |
| M85.842 | Other specified disorders of bone density and structure, left hand |
| M85.849 | Other specified disorders of bone density and structure, unspecified hand |
| M85.85 | Other specified disorders of bone density and structure, thigh |
| M85.851 | Other specified disorders of bone density and structure, right thigh |
| M85.852 | Other specified disorders of bone density and structure, left thigh |
| M85.859 | Other specified disorders of bone density and structure, unspecified thigh |
| M85.86 | Other specified disorders of bone density and structure, lower leg |
| M85.861 | Other specified disorders of bone density and structure, right lower leg |
| M85.862 | Other specified disorders of bone density and structure, left lower leg |
| M85.869 | Other specified disorders of bone density and structure, unspecified lower leg |
| M85.87 | Other specified disorders of bone density and structure, ankle and foot |
| M85.871 | Other specified disorders of bone density and structure, right ankle and foot |
| M85.872 | Other specified disorders of bone density and structure, left ankle and foot |
| M85.879 | Other specified disorders of bone density and structure, unspecified ankle and foot |
| M85.88 | Other specified disorders of bone density and structure, other site |
| M85.89 | Other specified disorders of bone density and structure, multiple sites |
| M85.9 | Disorder of bone density and structure, unspecified |
| M87 | Osteonecrosis |
| M87.0 | Idiopathic aseptic necrosis of bone |
| M87.00 | Idiopathic aseptic necrosis of unspecified bone |
| M87.01 | Idiopathic aseptic necrosis of shoulder |
| M87.011 | Idiopathic aseptic necrosis of right shoulder |
| M87.012 | Idiopathic aseptic necrosis of left shoulder |
| M87.019 | Idiopathic aseptic necrosis of unspecified shoulder |
| M87.02 | Idiopathic aseptic necrosis of humerus |
| M87.021 | Idiopathic aseptic necrosis of right humerus |
| M87.022 | Idiopathic aseptic necrosis of left humerus |
| M87.029 | Idiopathic aseptic necrosis of unspecified humerus |
| M87.03 | Idiopathic aseptic necrosis of radius, ulna and carpus |
| M87.031 | Idiopathic aseptic necrosis of right radius |
| M87.032 | Idiopathic aseptic necrosis of left radius |
| M87.033 | Idiopathic aseptic necrosis of unspecified radius |
| M87.034 | Idiopathic aseptic necrosis of right ulna |
| M87.035 | Idiopathic aseptic necrosis of left ulna |
| M87.036 | Idiopathic aseptic necrosis of unspecified ulna |
| M87.037 | Idiopathic aseptic necrosis of right carpus |
| M87.038 | Idiopathic aseptic necrosis of left carpus |
| M87.039 | Idiopathic aseptic necrosis of unspecified carpus |
| M87.04 | Idiopathic aseptic necrosis of hand and fingers |
| M87.041 | Idiopathic aseptic necrosis of right hand |
| M87.042 | Idiopathic aseptic necrosis of left hand |
| M87.043 | Idiopathic aseptic necrosis of unspecified hand |
| M87.044 | Idiopathic aseptic necrosis of right finger(s) |
| M87.045 | Idiopathic aseptic necrosis of left finger(s) |
| M87.046 | Idiopathic aseptic necrosis of unspecified finger(s) |
| M87.05 | Idiopathic aseptic necrosis of pelvis and femur |
| M87.050 | Idiopathic aseptic necrosis of pelvis |
| M87.051 | Idiopathic aseptic necrosis of right femur |
| M87.052 | Idiopathic aseptic necrosis of left femur |
| M87.059 | Idiopathic aseptic necrosis of unspecified femur |
| M87.06 | Idiopathic aseptic necrosis of tibia and fibula |
| M87.061 | Idiopathic aseptic necrosis of right tibia |
| M87.062 | Idiopathic aseptic necrosis of left tibia |
| M87.063 | Idiopathic aseptic necrosis of unspecified tibia |
| M87.064 | Idiopathic aseptic necrosis of right fibula |
| M87.065 | Idiopathic aseptic necrosis of left fibula |
| M87.066 | Idiopathic aseptic necrosis of unspecified fibula |
| M87.07 | Idiopathic aseptic necrosis of ankle, foot and toes |
| M87.071 | Idiopathic aseptic necrosis of right ankle |
| M87.072 | Idiopathic aseptic necrosis of left ankle |
| M87.073 | Idiopathic aseptic necrosis of unspecified ankle |
| M87.074 | Idiopathic aseptic necrosis of right foot |
| M87.075 | Idiopathic aseptic necrosis of left foot |
| M87.076 | Idiopathic aseptic necrosis of unspecified foot |
| M87.077 | Idiopathic aseptic necrosis of right toe(s) |
| M87.078 | Idiopathic aseptic necrosis of left toe(s) |
| M87.079 | Idiopathic aseptic necrosis of unspecified toe(s) |
| M87.08 | Idiopathic aseptic necrosis of bone, other site |
| M87.09 | Idiopathic aseptic necrosis of bone, multiple sites |
| M87.1 | Osteonecrosis due to drugs |
| M87.10 | Osteonecrosis due to drugs, unspecified bone |
| M87.11 | Osteonecrosis due to drugs, shoulder |
| M87.111 | Osteonecrosis due to drugs, right shoulder |
| M87.112 | Osteonecrosis due to drugs, left shoulder |
| M87.119 | Osteonecrosis due to drugs, unspecified shoulder |
| M87.12 | Osteonecrosis due to drugs, humerus |
| M87.121 | Osteonecrosis due to drugs, right humerus |
| M87.122 | Osteonecrosis due to drugs, left humerus |
| M87.129 | Osteonecrosis due to drugs, unspecified humerus |
| M87.13 | Osteonecrosis due to drugs of radius, ulna and carpus |
| M87.131 | Osteonecrosis due to drugs of right radius |
| M87.132 | Osteonecrosis due to drugs of left radius |
| M87.133 | Osteonecrosis due to drugs of unspecified radius |
| M87.134 | Osteonecrosis due to drugs of right ulna |
| M87.135 | Osteonecrosis due to drugs of left ulna |
| M87.136 | Osteonecrosis due to drugs of unspecified ulna |
| M87.137 | Osteonecrosis due to drugs of right carpus |
| M87.138 | Osteonecrosis due to drugs of left carpus |
| M87.139 | Osteonecrosis due to drugs of unspecified carpus |
| M87.14 | Osteonecrosis due to drugs, hand and fingers |
| M87.141 | Osteonecrosis due to drugs, right hand |
| M87.142 | Osteonecrosis due to drugs, left hand |
| M87.143 | Osteonecrosis due to drugs, unspecified hand |
| M87.144 | Osteonecrosis due to drugs, right finger(s) |
| M87.145 | Osteonecrosis due to drugs, left finger(s) |
| M87.146 | Osteonecrosis due to drugs, unspecified finger(s) |
| M87.15 | Osteonecrosis due to drugs, pelvis and femur |
| M87.150 | Osteonecrosis due to drugs, pelvis |
| M87.151 | Osteonecrosis due to drugs, right femur |
| M87.152 | Osteonecrosis due to drugs, left femur |
| M87.159 | Osteonecrosis due to drugs, unspecified femur |
| M87.16 | Osteonecrosis due to drugs, tibia and fibula |
| M87.161 | Osteonecrosis due to drugs, right tibia |
| M87.162 | Osteonecrosis due to drugs, left tibia |
| M87.163 | Osteonecrosis due to drugs, unspecified tibia |
| M87.164 | Osteonecrosis due to drugs, right fibula |
| M87.165 | Osteonecrosis due to drugs, left fibula |
| M87.166 | Osteonecrosis due to drugs, unspecified fibula |
| M87.17 | Osteonecrosis due to drugs, ankle, foot and toes |
| M87.171 | Osteonecrosis due to drugs, right ankle |
| M87.172 | Osteonecrosis due to drugs, left ankle |
| M87.173 | Osteonecrosis due to drugs, unspecified ankle |
| M87.174 | Osteonecrosis due to drugs, right foot |
| M87.175 | Osteonecrosis due to drugs, left foot |
| M87.176 | Osteonecrosis due to drugs, unspecified foot |
| M87.177 | Osteonecrosis due to drugs, right toe(s) |
| M87.178 | Osteonecrosis due to drugs, left toe(s) |
| M87.179 | Osteonecrosis due to drugs, unspecified toe(s) |
| M87.18 | Osteonecrosis due to drugs, other site |
| M87.180 | Osteonecrosis due to drugs, jaw |
| M87.188 | Osteonecrosis due to drugs, other site |
| M87.19 | Osteonecrosis due to drugs, multiple sites |
| M87.2 | Osteonecrosis due to previous trauma |
| M87.20 | Osteonecrosis due to previous trauma, unspecified bone |
| M87.21 | Osteonecrosis due to previous trauma, shoulder |
| M87.211 | Osteonecrosis due to previous trauma, right shoulder |
| M87.212 | Osteonecrosis due to previous trauma, left shoulder |
| M87.219 | Osteonecrosis due to previous trauma, unspecified shoulder |
| M87.22 | Osteonecrosis due to previous trauma, humerus |
| M87.221 | Osteonecrosis due to previous trauma, right humerus |
| M87.222 | Osteonecrosis due to previous trauma, left humerus |
| M87.229 | Osteonecrosis due to previous trauma, unspecified humerus |
| M87.23 | Osteonecrosis due to previous trauma of radius, ulna and carpus |
| M87.231 | Osteonecrosis due to previous trauma of right radius |
| M87.232 | Osteonecrosis due to previous trauma of left radius |
| M87.233 | Osteonecrosis due to previous trauma of unspecified radius |
| M87.234 | Osteonecrosis due to previous trauma of right ulna |
| M87.235 | Osteonecrosis due to previous trauma of left ulna |
| M87.236 | Osteonecrosis due to previous trauma of unspecified ulna |
| M87.237 | Osteonecrosis due to previous trauma of right carpus |
| M87.238 | Osteonecrosis due to previous trauma of left carpus |
| M87.239 | Osteonecrosis due to previous trauma of unspecified carpus |
| M87.24 | Osteonecrosis due to previous trauma, hand and fingers |
| M87.241 | Osteonecrosis due to previous trauma, right hand |
| M87.242 | Osteonecrosis due to previous trauma, left hand |
| M87.243 | Osteonecrosis due to previous trauma, unspecified hand |
| M87.244 | Osteonecrosis due to previous trauma, right finger(s) |
| M87.245 | Osteonecrosis due to previous trauma, left finger(s) |
| M87.246 | Osteonecrosis due to previous trauma, unspecified finger(s) |
| M87.25 | Osteonecrosis due to previous trauma, pelvis and femur |
| M87.250 | Osteonecrosis due to previous trauma, pelvis |
| M87.251 | Osteonecrosis due to previous trauma, right femur |
| M87.252 | Osteonecrosis due to previous trauma, left femur |
| M87.256 | Osteonecrosis due to previous trauma, unspecified femur |
| M87.26 | Osteonecrosis due to previous trauma, tibia and fibula |
| M87.261 | Osteonecrosis due to previous trauma, right tibia |
| M87.262 | Osteonecrosis due to previous trauma, left tibia |
| M87.263 | Osteonecrosis due to previous trauma, unspecified tibia |
| M87.264 | Osteonecrosis due to previous trauma, right fibula |
| M87.265 | Osteonecrosis due to previous trauma, left fibula |
| M87.266 | Osteonecrosis due to previous trauma, unspecified fibula |
| M87.27 | Osteonecrosis due to previous trauma, ankle, foot and toes |
| M87.271 | Osteonecrosis due to previous trauma, right ankle |
| M87.272 | Osteonecrosis due to previous trauma, left ankle |
| M87.273 | Osteonecrosis due to previous trauma, unspecified ankle |
| M87.274 | Osteonecrosis due to previous trauma, right foot |
| M87.275 | Osteonecrosis due to previous trauma, left foot |
| M87.276 | Osteonecrosis due to previous trauma, unspecified foot |
| M87.277 | Osteonecrosis due to previous trauma, right toe(s) |
| M87.278 | Osteonecrosis due to previous trauma, left toe(s) |
| M87.279 | Osteonecrosis due to previous trauma, unspecified toe(s) |
| M87.28 | Osteonecrosis due to previous trauma, other site |
| M87.29 | Osteonecrosis due to previous trauma, multiple sites |
| M87.3 | Other secondary osteonecrosis |
| M87.30 | Other secondary osteonecrosis, unspecified bone |
| M87.31 | Other secondary osteonecrosis, shoulder |
| M87.311 | Other secondary osteonecrosis, right shoulder |
| M87.312 | Other secondary osteonecrosis, left shoulder |
| M87.319 | Other secondary osteonecrosis, unspecified shoulder |
| M87.32 | Other secondary osteonecrosis, humerus |
| M87.321 | Other secondary osteonecrosis, right humerus |
| M87.322 | Other secondary osteonecrosis, left humerus |
| M87.329 | Other secondary osteonecrosis, unspecified humerus |
| M87.33 | Other secondary osteonecrosis of radius, ulna and carpus |
| M87.331 | Other secondary osteonecrosis of right radius |
| M87.332 | Other secondary osteonecrosis of left radius |
| M87.333 | Other secondary osteonecrosis of unspecified radius |
| M87.334 | Other secondary osteonecrosis of right ulna |
| M87.335 | Other secondary osteonecrosis of left ulna |
| M87.336 | Other secondary osteonecrosis of unspecified ulna |
| M87.337 | Other secondary osteonecrosis of right carpus |
| M87.338 | Other secondary osteonecrosis of left carpus |
| M87.339 | Other secondary osteonecrosis of unspecified carpus |
| M87.34 | Other secondary osteonecrosis, hand and fingers |
| M87.341 | Other secondary osteonecrosis, right hand |
| M87.342 | Other secondary osteonecrosis, left hand |
| M87.343 | Other secondary osteonecrosis, unspecified hand |
| M87.344 | Other secondary osteonecrosis, right finger(s) |
| M87.345 | Other secondary osteonecrosis, left finger(s) |
| M87.346 | Other secondary osteonecrosis, unspecified finger(s) |
| M87.35 | Other secondary osteonecrosis, pelvis and femur |
| M87.350 | Other secondary osteonecrosis, pelvis |
| M87.351 | Other secondary osteonecrosis, right femur |
| M87.352 | Other secondary osteonecrosis, left femur |
| M87.353 | Other secondary osteonecrosis, unspecified femur |
| M87.36 | Other secondary osteonecrosis, tibia and fibula |
| M87.361 | Other secondary osteonecrosis, right tibia |
| M87.362 | Other secondary osteonecrosis, left tibia |
| M87.363 | Other secondary osteonecrosis, unspecified tibia |
| M87.364 | Other secondary osteonecrosis, right fibula |
| M87.365 | Other secondary osteonecrosis, left fibula |
| M87.366 | Other secondary osteonecrosis, unspecified fibula |
| M87.37 | Other secondary osteonecrosis, ankle and foot |
| M87.371 | Other secondary osteonecrosis, right ankle |
| M87.372 | Other secondary osteonecrosis, left ankle |
| M87.373 | Other secondary osteonecrosis, unspecified ankle |
| M87.374 | Other secondary osteonecrosis, right foot |
| M87.375 | Other secondary osteonecrosis, left foot |
| M87.376 | Other secondary osteonecrosis, unspecified foot |
| M87.377 | Other secondary osteonecrosis, right toe(s) |
| M87.378 | Other secondary osteonecrosis, left toe(s) |
| M87.379 | Other secondary osteonecrosis, unspecified toe(s) |
| M87.38 | Other secondary osteonecrosis, other site |
| M87.39 | Other secondary osteonecrosis, multiple sites |
| M87.8 | Other osteonecrosis |
| M87.80 | Other osteonecrosis, unspecified bone |
| M87.81 | Other osteonecrosis, shoulder |
| M87.811 | Other osteonecrosis, right shoulder |
| M87.812 | Other osteonecrosis, left shoulder |
| M87.819 | Other osteonecrosis, unspecified shoulder |
| M87.82 | Other osteonecrosis, humerus |
| M87.821 | Other osteonecrosis, right humerus |
| M87.822 | Other osteonecrosis, left humerus |
| M87.829 | Other osteonecrosis, unspecified humerus |
| M87.83 | Other osteonecrosis of radius, ulna and carpus |
| M87.831 | Other osteonecrosis of right radius |
| M87.832 | Other osteonecrosis of left radius |
| M87.833 | Other osteonecrosis of unspecified radius |
| M87.834 | Other osteonecrosis of right ulna |
| M87.835 | Other osteonecrosis of left ulna |
| M87.836 | Other osteonecrosis of unspecified ulna |
| M87.837 | Other osteonecrosis of right carpus |
| M87.838 | Other osteonecrosis of left carpus |
| M87.839 | Other osteonecrosis of unspecified carpus |
| M87.84 | Other osteonecrosis, hand and fingers |
| M87.841 | Other osteonecrosis, right hand |
| M87.842 | Other osteonecrosis, left hand |
| M87.843 | Other osteonecrosis, unspecified hand |
| M87.844 | Other osteonecrosis, right finger(s) |
| M87.845 | Other osteonecrosis, left finger(s) |
| M87.849 | Other osteonecrosis, unspecified finger(s) |
| M87.85 | Other osteonecrosis, pelvis and femur |
| M87.850 | Other osteonecrosis, pelvis |
| M87.851 | Other osteonecrosis, right femur |
| M87.852 | Other osteonecrosis, left femur |
| M87.859 | Other osteonecrosis, unspecified femur |
| M87.86 | Other osteonecrosis, tibia and fibula |
| M87.861 | Other osteonecrosis, right tibia |
| M87.862 | Other osteonecrosis, left tibia |
| M87.863 | Other osteonecrosis, unspecified tibia |
| M87.864 | Other osteonecrosis, right fibula |
| M87.865 | Other osteonecrosis, left fibula |
| M87.869 | Other osteonecrosis, unspecified fibula |
| M87.87 | Other osteonecrosis, ankle, foot and toes |
| M87.871 | Other osteonecrosis, right ankle |
| M87.872 | Other osteonecrosis, left ankle |
| M87.873 | Other osteonecrosis, unspecified ankle |
| M87.874 | Other osteonecrosis, right foot |
| M87.875 | Other osteonecrosis, left foot |
| M87.876 | Other osteonecrosis, unspecified foot |
| M87.877 | Other osteonecrosis, right toe(s) |
| M87.878 | Other osteonecrosis, left toe(s) |
| M87.879 | Other osteonecrosis, unspecified toe(s) |
| M87.88 | Other osteonecrosis, other site |
| M87.89 | Other osteonecrosis, multiple sites |
| M87.9 | Osteonecrosis, unspecified |
| M89 | Other disorders of bone |
| M89.0 | Algoneurodystrophy |
| M89.00 | Algoneurodystrophy, unspecified site |
| M89.01 | Algoneurodystrophy, shoulder |
| M89.011 | Algoneurodystrophy, right shoulder |
| M89.012 | Algoneurodystrophy, left shoulder |
| M89.019 | Algoneurodystrophy, unspecified shoulder |
| M89.02 | Algoneurodystrophy, upper arm |
| M89.021 | Algoneurodystrophy, right upper arm |
| M89.022 | Algoneurodystrophy, left upper arm |
| M89.029 | Algoneurodystrophy, unspecified upper arm |
| M89.03 | Algoneurodystrophy, forearm |
| M89.031 | Algoneurodystrophy, right forearm |
| M89.032 | Algoneurodystrophy, left forearm |
| M89.039 | Algoneurodystrophy, unspecified forearm |
| M89.04 | Algoneurodystrophy, hand |
| M89.041 | Algoneurodystrophy, right hand |
| M89.042 | Algoneurodystrophy, left hand |
| M89.049 | Algoneurodystrophy, unspecified hand |
| M89.05 | Algoneurodystrophy, thigh |
| M89.051 | Algoneurodystrophy, right thigh |
| M89.052 | Algoneurodystrophy, left thigh |
| M89.059 | Algoneurodystrophy, unspecified thigh |
| M89.06 | Algoneurodystrophy, lower leg |
| M89.061 | Algoneurodystrophy, right lower leg |
| M89.062 | Algoneurodystrophy, left lower leg |
| M89.069 | Algoneurodystrophy, unspecified lower leg |
| M89.07 | Algoneurodystrophy, ankle and foot |
| M89.071 | Algoneurodystrophy, right ankle and foot |
| M89.072 | Algoneurodystrophy, left ankle and foot |
| M89.079 | Algoneurodystrophy, unspecified ankle and foot |
| M89.08 | Algoneurodystrophy, other site |
| M89.09 | Algoneurodystrophy, multiple sites |
| M89.1 | Physeal arrest |
| M89.12 | Physeal arrest, humerus |
| M89.121 | Complete physeal arrest, right proximal humerus |
| M89.122 | Complete physeal arrest, left proximal humerus |
| M89.123 | Partial physeal arrest, right proximal humerus |
| M89.124 | Partial physeal arrest, left proximal humerus |
| M89.125 | Complete physeal arrest, right distal humerus |
| M89.126 | Complete physeal arrest, left distal humerus |
| M89.127 | Partial physeal arrest, right distal humerus |
| M89.128 | Partial physeal arrest, left distal humerus |
| M89.129 | Physeal arrest, humerus, unspecified |
| M89.13 | Physeal arrest, forearm |
| M89.131 | Complete physeal arrest, right distal radius |
| M89.132 | Complete physeal arrest, left distal radius |
| M89.133 | Partial physeal arrest, right distal radius |
| M89.134 | Partial physeal arrest, left distal radius |
| M89.138 | Other physeal arrest of forearm |
| M89.139 | Physeal arrest, forearm, unspecified |
| M89.15 | Physeal arrest, femur |
| M89.151 | Complete physeal arrest, right proximal femur |
| M89.152 | Complete physeal arrest, left proximal femur |
| M89.153 | Partial physeal arrest, right proximal femur |
| M89.154 | Partial physeal arrest, left proximal femur |
| M89.155 | Complete physeal arrest, right distal femur |
| M89.156 | Complete physeal arrest, left distal femur |
| M89.157 | Partial physeal arrest, right distal femur |
| M89.158 | Partial physeal arrest, left distal femur |
| M89.159 | Physeal arrest, femur, unspecified |
| M89.16 | Physeal arrest, lower leg |
| M89.160 | Complete physeal arrest, right proximal tibia |
| M89.161 | Complete physeal arrest, left proximal tibia |
| M89.162 | Partial physeal arrest, right proximal tibia |
| M89.163 | Partial physeal arrest, left proximal tibia |
| M89.164 | Complete physeal arrest, right distal tibia |
| M89.165 | Complete physeal arrest, left distal tibia |
| M89.166 | Partial physeal arrest, right distal tibia |
| M89.167 | Partial physeal arrest, left distal tibia |
| M89.168 | Other physeal arrest of lower leg |
| M89.169 | Physeal arrest, lower leg, unspecified |
| M89.18 | Physeal arrest, other site |
| M89.2 | Other disorders of bone development and growth |
| M89.20 | Other disorders of bone development and growth, unspecified site |
| M89.21 | Other disorders of bone development and growth, shoulder |
| M89.211 | Other disorders of bone development and growth, right shoulder |
| M89.212 | Other disorders of bone development and growth, left shoulder |
| M89.219 | Other disorders of bone development and growth, unspecified shoulder |
| M89.22 | Other disorders of bone development and growth, humerus |
| M89.221 | Other disorders of bone development and growth, right humerus |
| M89.222 | Other disorders of bone development and growth, left humerus |
| M89.229 | Other disorders of bone development and growth, unspecified humerus |
| M89.23 | Other disorders of bone development and growth, ulna and radius |
| M89.231 | Other disorders of bone development and growth, right ulna |
| M89.232 | Other disorders of bone development and growth, left ulna |
| M89.233 | Other disorders of bone development and growth, right radius |
| M89.234 | Other disorders of bone development and growth, left radius |
| M89.239 | Other disorders of bone development and growth, unspecified ulna and radius |
| M89.24 | Other disorders of bone development and growth, hand |
| M89.241 | Other disorders of bone development and growth, right hand |
| M89.242 | Other disorders of bone development and growth, left hand |
| M89.249 | Other disorders of bone development and growth, unspecified hand |
| M89.25 | Other disorders of bone development and growth, femur |
| M89.251 | Other disorders of bone development and growth, right femur |
| M89.252 | Other disorders of bone development and growth, left femur |
| M89.259 | Other disorders of bone development and growth, unspecified femur |
| M89.26 | Other disorders of bone development and growth, tibia and fibula |
| M89.261 | Other disorders of bone development and growth, right tibia |
| M89.262 | Other disorders of bone development and growth, left tibia |
| M89.263 | Other disorders of bone development and growth, right fibula |
| M89.264 | Other disorders of bone development and growth, left fibula |
| M89.269 | Other disorders of bone development and growth, unspecified lower leg |
| M89.27 | Other disorders of bone development and growth, ankle and foot |
| M89.271 | Other disorders of bone development and growth, right ankle and foot |
| M89.272 | Other disorders of bone development and growth, left ankle and foot |
| M89.279 | Other disorders of bone development and growth, unspecified ankle and foot |
| M89.28 | Other disorders of bone development and growth, other site |
| M89.29 | Other disorders of bone development and growth, multiple sites |
| M89.3 | Hypertrophy of bone |
| M89.30 | Hypertrophy of bone, unspecified site |
| M89.31 | Hypertrophy of bone, shoulder |
| M89.311 | Hypertrophy of bone, right shoulder |
| M89.312 | Hypertrophy of bone, left shoulder |
| M89.319 | Hypertrophy of bone, unspecified shoulder |
| M89.32 | Hypertrophy of bone, humerus |
| M89.321 | Hypertrophy of bone, right humerus |
| M89.322 | Hypertrophy of bone, left humerus |
| M89.329 | Hypertrophy of bone, unspecified humerus |
| M89.33 | Hypertrophy of bone, ulna and radius |
| M89.331 | Hypertrophy of bone, right ulna |
| M89.332 | Hypertrophy of bone, left ulna |
| M89.333 | Hypertrophy of bone, right radius |
| M89.334 | Hypertrophy of bone, left radius |
| M89.339 | Hypertrophy of bone, unspecified ulna and radius |
| M89.34 | Hypertrophy of bone, hand |
| M89.341 | Hypertrophy of bone, right hand |
| M89.342 | Hypertrophy of bone, left hand |
| M89.349 | Hypertrophy of bone, unspecified hand |
| M89.35 | Hypertrophy of bone, femur |
| M89.351 | Hypertrophy of bone, right femur |
| M89.352 | Hypertrophy of bone, left femur |
| M89.359 | Hypertrophy of bone, unspecified femur |
| M89.36 | Hypertrophy of bone, tibia and fibula |
| M89.361 | Hypertrophy of bone, right tibia |
| M89.362 | Hypertrophy of bone, left tibia |
| M89.363 | Hypertrophy of bone, right fibula |
| M89.364 | Hypertrophy of bone, left fibula |
| M89.369 | Hypertrophy of bone, unspecified tibia and fibula |
| M89.37 | Hypertrophy of bone, ankle and foot |
| M89.371 | Hypertrophy of bone, right ankle and foot |
| M89.372 | Hypertrophy of bone, left ankle and foot |
| M89.379 | Hypertrophy of bone, unspecified ankle and foot |
| M89.38 | Hypertrophy of bone, other site |
| M89.39 | Hypertrophy of bone, multiple sites |
| M89.4 | Other hypertrophic osteoarthropathy |
| M89.40 | Other hypertrophic osteoarthropathy, unspecified site |
| M89.41 | Other hypertrophic osteoarthropathy, shoulder |
| M89.411 | Other hypertrophic osteoarthropathy, right shoulder |
| M89.412 | Other hypertrophic osteoarthropathy, left shoulder |
| M89.419 | Other hypertrophic osteoarthropathy, unspecified shoulder |
| M89.42 | Other hypertrophic osteoarthropathy, upper arm |
| M89.421 | Other hypertrophic osteoarthropathy, right upper arm |
| M89.422 | Other hypertrophic osteoarthropathy, left upper arm |
| M89.429 | Other hypertrophic osteoarthropathy, unspecified upper arm |
| M89.43 | Other hypertrophic osteoarthropathy, forearm |
| M89.431 | Other hypertrophic osteoarthropathy, right forearm |
| M89.432 | Other hypertrophic osteoarthropathy, left forearm |
| M89.439 | Other hypertrophic osteoarthropathy, unspecified forearm |
| M89.44 | Other hypertrophic osteoarthropathy, hand |
| M89.441 | Other hypertrophic osteoarthropathy, right hand |
| M89.442 | Other hypertrophic osteoarthropathy, left hand |
| M89.449 | Other hypertrophic osteoarthropathy, unspecified hand |
| M89.45 | Other hypertrophic osteoarthropathy, thigh |
| M89.451 | Other hypertrophic osteoarthropathy, right thigh |
| M89.452 | Other hypertrophic osteoarthropathy, left thigh |
| M89.459 | Other hypertrophic osteoarthropathy, unspecified thigh |
| M89.46 | Other hypertrophic osteoarthropathy, lower leg |
| M89.461 | Other hypertrophic osteoarthropathy, right lower leg |
| M89.462 | Other hypertrophic osteoarthropathy, left lower leg |
| M89.469 | Other hypertrophic osteoarthropathy, unspecified lower leg |
| M89.47 | Other hypertrophic osteoarthropathy, ankle and foot |
| M89.471 | Other hypertrophic osteoarthropathy, right ankle and foot |
| M89.472 | Other hypertrophic osteoarthropathy, left ankle and foot |
| M89.479 | Other hypertrophic osteoarthropathy, unspecified ankle and foot |
| M89.48 | Other hypertrophic osteoarthropathy, other site |
| M89.49 | Other hypertrophic osteoarthropathy, multiple sites |
| M89.5 | Osteolysis |
| M89.50 | Osteolysis, unspecified site |
| M89.51 | Osteolysis, shoulder |
| M89.511 | Osteolysis, right shoulder |
| M89.512 | Osteolysis, left shoulder |
| M89.519 | Osteolysis, unspecified shoulder |
| M89.52 | Osteolysis, upper arm |
| M89.521 | Osteolysis, right upper arm |
| M89.522 | Osteolysis, left upper arm |
| M89.529 | Osteolysis, unspecified upper arm |
| M89.53 | Osteolysis, forearm |
| M89.531 | Osteolysis, right forearm |
| M89.532 | Osteolysis, left forearm |
| M89.539 | Osteolysis, unspecified forearm |
| M89.54 | Osteolysis, hand |
| M89.541 | Osteolysis, right hand |
| M89.542 | Osteolysis, left hand |
| M89.549 | Osteolysis, unspecified hand |
| M89.55 | Osteolysis, thigh |
| M89.551 | Osteolysis, right thigh |
| M89.552 | Osteolysis, left thigh |
| M89.559 | Osteolysis, unspecified thigh |
| M89.56 | Osteolysis, lower leg |
| M89.561 | Osteolysis, right lower leg |
| M89.562 | Osteolysis, left lower leg |
| M89.569 | Osteolysis, unspecified lower leg |
| M89.57 | Osteolysis, ankle and foot |
| M89.571 | Osteolysis, right ankle and foot |
| M89.572 | Osteolysis, left ankle and foot |
| M89.579 | Osteolysis, unspecified ankle and foot |
| M89.58 | Osteolysis, other site |
| M89.59 | Osteolysis, multiple sites |
| M89.6 | Osteopathy after poliomyelitis |
| M89.60 | Osteopathy after poliomyelitis, unspecified site |
| M89.61 | Osteopathy after poliomyelitis, shoulder |
| M89.611 | Osteopathy after poliomyelitis, right shoulder |
| M89.612 | Osteopathy after poliomyelitis, left shoulder |
| M89.619 | Osteopathy after poliomyelitis, unspecified shoulder |
| M89.62 | Osteopathy after poliomyelitis, upper arm |
| M89.621 | Osteopathy after poliomyelitis, right upper arm |
| M89.622 | Osteopathy after poliomyelitis, left upper arm |
| M89.629 | Osteopathy after poliomyelitis, unspecified upper arm |
| M89.63 | Osteopathy after poliomyelitis, forearm |
| M89.631 | Osteopathy after poliomyelitis, right forearm |
| M89.632 | Osteopathy after poliomyelitis, left forearm |
| M89.639 | Osteopathy after poliomyelitis, unspecified forearm |
| M89.64 | Osteopathy after poliomyelitis, hand |
| M89.641 | Osteopathy after poliomyelitis, right hand |
| M89.642 | Osteopathy after poliomyelitis, left hand |
| M89.649 | Osteopathy after poliomyelitis, unspecified hand |
| M89.65 | Osteopathy after poliomyelitis, thigh |
| M89.651 | Osteopathy after poliomyelitis, right thigh |
| M89.652 | Osteopathy after poliomyelitis, left thigh |
| M89.659 | Osteopathy after poliomyelitis, unspecified thigh |
| M89.66 | Osteopathy after poliomyelitis, lower leg |
| M89.661 | Osteopathy after poliomyelitis, right lower leg |
| M89.662 | Osteopathy after poliomyelitis, left lower leg |
| M89.669 | Osteopathy after poliomyelitis, unspecified lower leg |
| M89.67 | Osteopathy after poliomyelitis, ankle and foot |
| M89.671 | Osteopathy after poliomyelitis, right ankle and foot |
| M89.672 | Osteopathy after poliomyelitis, left ankle and foot |
| M89.679 | Osteopathy after poliomyelitis, unspecified ankle and foot |
| M89.68 | Osteopathy after poliomyelitis, other site |
| M89.69 | Osteopathy after poliomyelitis, multiple sites |
| M89.7 | Major osseous defect |
| M89.70 | Major osseous defect, unspecified site |
| M89.71 | Major osseous defect, shoulder region |
| M89.711 | Major osseous defect, right shoulder region |
| M89.712 | Major osseous defect, left shoulder region |
| M89.719 | Major osseous defect, unspecified shoulder region |
| M89.72 | Major osseous defect, humerus |
| M89.721 | Major osseous defect, right humerus |
| M89.722 | Major osseous defect, left humerus |
| M89.729 | Major osseous defect, unspecified humerus |
| M89.73 | Major osseous defect, forearm |
| M89.731 | Major osseous defect, right forearm |
| M89.732 | Major osseous defect, left forearm |
| M89.739 | Major osseous defect, unspecified forearm |
| M89.74 | Major osseous defect, hand |
| M89.741 | Major osseous defect, right hand |
| M89.742 | Major osseous defect, left hand |
| M89.749 | Major osseous defect, unspecified hand |
| M89.75 | Major osseous defect, pelvic region and thigh |
| M89.751 | Major osseous defect, right pelvic region and thigh |
| M89.752 | Major osseous defect, left pelvic region and thigh |
| M89.759 | Major osseous defect, unspecified pelvic region and thigh |
| M89.76 | Major osseous defect, lower leg |
| M89.761 | Major osseous defect, right lower leg |
| M89.762 | Major osseous defect, left lower leg |
| M89.769 | Major osseous defect, unspecified lower leg |
| M89.77 | Major osseous defect, ankle and foot |
| M89.771 | Major osseous defect, right ankle and foot |
| M89.772 | Major osseous defect, left ankle and foot |
| M89.779 | Major osseous defect, unspecified ankle and foot |
| M89.78 | Major osseous defect, other site |
| M89.79 | Major osseous defect, multiple sites |
| M89.8 | Other specified disorders of bone |
| M89.8X | Other specified disorders of bone |
| M89.8X0 | Other specified disorders of bone, multiple sites |
| M89.8X1 | Other specified disorders of bone, shoulder |
| M89.8X2 | Other specified disorders of bone, upper arm |
| M89.8X3 | Other specified disorders of bone, forearm |
| M89.8X4 | Other specified disorders of bone, hand |
| M89.8X5 | Other specified disorders of bone, thigh |
| M89.8X6 | Other specified disorders of bone, lower leg |
| M89.8X7 | Other specified disorders of bone, ankle and foot |
| M89.8X8 | Other specified disorders of bone, other site |
| M89.8X9 | Other specified disorders of bone, unspecified site |
| M89.9 | Disorder of bone, unspecified |
| M94 | Other disorders of cartilage |
| M94.0 | Chondrocostal junction syndrome [Tietze] |
| M94.1 | Relapsing polychondritis |
| M94.2 | Chondromalacia |
| M94.20 | Chondromalacia, unspecified site |
| M94.21 | Chondromalacia, shoulder |
| M94.211 | Chondromalacia, right shoulder |
| M94.212 | Chondromalacia, left shoulder |
| M94.219 | Chondromalacia, unspecified shoulder |
| M94.22 | Chondromalacia, elbow |
| M94.221 | Chondromalacia, right elbow |
| M94.222 | Chondromalacia, left elbow |
| M94.229 | Chondromalacia, unspecified elbow |
| M94.23 | Chondromalacia, wrist |
| M94.231 | Chondromalacia, right wrist |
| M94.232 | Chondromalacia, left wrist |
| M94.239 | Chondromalacia, unspecified wrist |
| M94.24 | Chondromalacia, joints of hand |
| M94.241 | Chondromalacia, joints of right hand |
| M94.242 | Chondromalacia, joints of left hand |
| M94.249 | Chondromalacia, joints of unspecified hand |
| M94.25 | Chondromalacia, hip |
| M94.251 | Chondromalacia, right hip |
| M94.252 | Chondromalacia, left hip |
| M94.259 | Chondromalacia, unspecified hip |
| M94.26 | Chondromalacia, knee |
| M94.261 | Chondromalacia, right knee |
| M94.262 | Chondromalacia, left knee |
| M94.269 | Chondromalacia, unspecified knee |
| M94.27 | Chondromalacia, ankle and joints of foot |
| M94.271 | Chondromalacia, right ankle and joints of right foot |
| M94.272 | Chondromalacia, left ankle and joints of left foot |
| M94.279 | Chondromalacia, unspecified ankle and joints of foot |
| M94.28 | Chondromalacia, other site |
| M94.29 | Chondromalacia, multiple sites |
| M94.3 | Chondrolysis |
| M94.35 | Chondrolysis, hip |
| M94.351 | Chondrolysis, right hip |
| M94.352 | Chondrolysis, left hip |
| M94.359 | Chondrolysis, unspecified hip |
| M94.8 | Other specified disorders of cartilage |
| M94.8X | Other specified disorders of cartilage |
| M94.8X0 | Other specified disorders of cartilage, multiple sites |
| M94.8X1 | Other specified disorders of cartilage, shoulder |
| M94.8X2 | Other specified disorders of cartilage, upper arm |
| M94.8X3 | Other specified disorders of cartilage, forearm |
| M94.8X4 | Other specified disorders of cartilage, hand |
| M94.8X5 | Other specified disorders of cartilage, thigh |
| M94.8X6 | Other specified disorders of cartilage, lower leg |
| M94.8X7 | Other specified disorders of cartilage, ankle and foot |
| M94.8X8 | Other specified disorders of cartilage, other site |
| M94.8X9 | Other specified disorders of cartilage, unspecified sites |
| M94.9 | Disorder of cartilage, unspecified |
| M95 | Other acquired deformities of musculoskeletal system and connective tissue |
| M95.0 | Acquired deformity of nose |
| M95.1 | Cauliflower ear |
| M95.10 | Cauliflower ear, unspecified ear |
| M95.11 | Cauliflower ear, right ear |
| M95.12 | Cauliflower ear, left ear |
| M95.2 | Other acquired deformity of head |
| M95.3 | Acquired deformity of neck |
| M95.4 | Acquired deformity of chest and rib |
| M95.5 | Acquired deformity of pelvis |
| M95.8 | Other specified acquired deformities of musculoskeletal system |
| M95.9 | Acquired deformity of musculoskeletal system, unspecified |
| Q06.3 | Other congenital cauda equina malformations |
| S12 | Fracture of cervical vertebra and other parts of neck |
| S12.0 | Fracture of first cervical vertebra |
| S12.00 | Unspecified fracture of first cervical vertebra |
| S12.000 | Unspecified displaced fracture of first cervical vertebra |
| S12.000A | Unspecified displaced fracture of first cervical vertebra, initial encounter for closed fracture |
| S12.000B | Unspecified displaced fracture of first cervical vertebra, initial encounter for open fracture |
| S12.000D | Unspecified displaced fracture of first cervical vertebra, subsequent encounter for fracture with routine healing |
| S12.000G | Unspecified displaced fracture of first cervical vertebra, subsequent encounter for fracture with delayed healing |
| S12.000K | Unspecified displaced fracture of first cervical vertebra, subsequent encounter for fracture with nonunion |
| S12.000S | Unspecified displaced fracture of first cervical vertebra, sequela |
| S12.001 | Unspecified nondisplaced fracture of first cervical vertebra |
| S12.001A | Unspecified nondisplaced fracture of first cervical vertebra, initial encounter for closed fracture |
| S12.001B | Unspecified nondisplaced fracture of first cervical vertebra, initial encounter for open fracture |
| S12.001D | Unspecified nondisplaced fracture of first cervical vertebra, subsequent encounter for fracture with routine healing |
| S12.001G | Unspecified nondisplaced fracture of first cervical vertebra, subsequent encounter for fracture with delayed healing |
| S12.001K | Unspecified nondisplaced fracture of first cervical vertebra, subsequent encounter for fracture with nonunion |
| S12.001S | Unspecified nondisplaced fracture of first cervical vertebra, sequela |
| S12.01 | Stable burst fracture of first cervical vertebra |
| S12.01XA | Stable burst fracture of first cervical vertebra, initial encounter for closed fracture |
| S12.01XB | Stable burst fracture of first cervical vertebra, initial encounter for open fracture |
| S12.01XD | Stable burst fracture of first cervical vertebra, subsequent encounter for fracture with routine healing |
| S12.01XG | Stable burst fracture of first cervical vertebra, subsequent encounter for fracture with delayed healing |
| S12.01XK | Stable burst fracture of first cervical vertebra, subsequent encounter for fracture with nonunion |
| S12.01XS | Stable burst fracture of first cervical vertebra, sequela |
| S12.02 | Unstable burst fracture of first cervical vertebra |
| S12.02XA | Unstable burst fracture of first cervical vertebra, initial encounter for closed fracture |
| S12.02XB | Unstable burst fracture of first cervical vertebra, initial encounter for open fracture |
| S12.02XD | Unstable burst fracture of first cervical vertebra, subsequent encounter for fracture with routine healing |
| S12.02XG | Unstable burst fracture of first cervical vertebra, subsequent encounter for fracture with delayed healing |
| S12.02XK | Unstable burst fracture of first cervical vertebra, subsequent encounter for fracture with nonunion |
| S12.02XS | Unstable burst fracture of first cervical vertebra, sequela |
| S12.03 | Posterior arch fracture of first cervical vertebra |
| S12.030 | Displaced posterior arch fracture of first cervical vertebra |
| S12.030A | Displaced posterior arch fracture of first cervical vertebra, initial encounter for closed fracture |
| S12.030B | Displaced posterior arch fracture of first cervical vertebra, initial encounter for open fracture |
| S12.030D | Displaced posterior arch fracture of first cervical vertebra, subsequent encounter for fracture with routine healing |
| S12.030G | Displaced posterior arch fracture of first cervical vertebra, subsequent encounter for fracture with delayed healing |
| S12.030K | Displaced posterior arch fracture of first cervical vertebra, subsequent encounter for fracture with nonunion |
| S12.030S | Displaced posterior arch fracture of first cervical vertebra, sequela |
| S12.031 | Nondisplaced posterior arch fracture of first cervical vertebra |
| S12.031A | Nondisplaced posterior arch fracture of first cervical vertebra, initial encounter for closed fracture |
| S12.031B | Nondisplaced posterior arch fracture of first cervical vertebra, initial encounter for open fracture |
| S12.031D | Nondisplaced posterior arch fracture of first cervical vertebra, subsequent encounter for fracture with routine healing |
| S12.031G | Nondisplaced posterior arch fracture of first cervical vertebra, subsequent encounter for fracture with delayed healing |
| S12.031K | Nondisplaced posterior arch fracture of first cervical vertebra, subsequent encounter for fracture with nonunion |
| S12.031S | Nondisplaced posterior arch fracture of first cervical vertebra, sequela |
| S12.04 | Lateral mass fracture of first cervical vertebra |
| S12.040 | Displaced lateral mass fracture of first cervical vertebra |
| S12.040A | Displaced lateral mass fracture of first cervical vertebra, initial encounter for closed fracture |
| S12.040B | Displaced lateral mass fracture of first cervical vertebra, initial encounter for open fracture |
| S12.040D | Displaced lateral mass fracture of first cervical vertebra, subsequent encounter for fracture with routine healing |
| S12.040G | Displaced lateral mass fracture of first cervical vertebra, subsequent encounter for fracture with delayed healing |
| S12.040K | Displaced lateral mass fracture of first cervical vertebra, subsequent encounter for fracture with nonunion |
| S12.040S | Displaced lateral mass fracture of first cervical vertebra, sequela |
| S12.041 | Nondisplaced lateral mass fracture of first cervical vertebra |
| S12.041A | Nondisplaced lateral mass fracture of first cervical vertebra, initial encounter for closed fracture |
| S12.041B | Nondisplaced lateral mass fracture of first cervical vertebra, initial encounter for open fracture |
| S12.041D | Nondisplaced lateral mass fracture of first cervical vertebra, subsequent encounter for fracture with routine healing |
| S12.041G | Nondisplaced lateral mass fracture of first cervical vertebra, subsequent encounter for fracture with delayed healing |
| S12.041K | Nondisplaced lateral mass fracture of first cervical vertebra, subsequent encounter for fracture with nonunion |
| S12.041S | Nondisplaced lateral mass fracture of first cervical vertebra, sequela |
| S12.09 | Other fracture of first cervical vertebra |
| S12.090 | Other displaced fracture of first cervical vertebra |
| S12.090A | Other displaced fracture of first cervical vertebra, initial encounter for closed fracture |
| S12.090B | Other displaced fracture of first cervical vertebra, initial encounter for open fracture |
| S12.090D | Other displaced fracture of first cervical vertebra, subsequent encounter for fracture with routine healing |
| S12.090G | Other displaced fracture of first cervical vertebra, subsequent encounter for fracture with delayed healing |
| S12.090K | Other displaced fracture of first cervical vertebra, subsequent encounter for fracture with nonunion |
| S12.090S | Other displaced fracture of first cervical vertebra, sequela |
| S12.091 | Other nondisplaced fracture of first cervical vertebra |
| S12.091A | Other nondisplaced fracture of first cervical vertebra, initial encounter for closed fracture |
| S12.091B | Other nondisplaced fracture of first cervical vertebra, initial encounter for open fracture |
| S12.091D | Other nondisplaced fracture of first cervical vertebra, subsequent encounter for fracture with routine healing |
| S12.091G | Other nondisplaced fracture of first cervical vertebra, subsequent encounter for fracture with delayed healing |
| S12.091K | Other nondisplaced fracture of first cervical vertebra, subsequent encounter for fracture with nonunion |
| S12.091S | Other nondisplaced fracture of first cervical vertebra, sequela |
| S12.1 | Fracture of second cervical vertebra |
| S12.10 | Unspecified fracture of second cervical vertebra |
| S12.100 | Unspecified displaced fracture of second cervical vertebra |
| S12.100A | Unspecified displaced fracture of second cervical vertebra, initial encounter for closed fracture |
| S12.100B | Unspecified displaced fracture of second cervical vertebra, initial encounter for open fracture |
| S12.100D | Unspecified displaced fracture of second cervical vertebra, subsequent encounter for fracture with routine healing |
| S12.100G | Unspecified displaced fracture of second cervical vertebra, subsequent encounter for fracture with delayed healing |
| S12.100K | Unspecified displaced fracture of second cervical vertebra, subsequent encounter for fracture with nonunion |
| S12.100S | Unspecified displaced fracture of second cervical vertebra, sequela |
| S12.101 | Unspecified nondisplaced fracture of second cervical vertebra |
| S12.101A | Unspecified nondisplaced fracture of second cervical vertebra, initial encounter for closed fracture |
| S12.101B | Unspecified nondisplaced fracture of second cervical vertebra, initial encounter for open fracture |
| S12.101D | Unspecified nondisplaced fracture of second cervical vertebra, subsequent encounter for fracture with routine healing |
| S12.101G | Unspecified nondisplaced fracture of second cervical vertebra, subsequent encounter for fracture with delayed healing |
| S12.101K | Unspecified nondisplaced fracture of second cervical vertebra, subsequent encounter for fracture with nonunion |
| S12.101S | Unspecified nondisplaced fracture of second cervical vertebra, sequela |
| S12.11 | Type II dens fracture |
| S12.110 | Anterior displaced Type II dens fracture |
| S12.110A | Anterior displaced Type II dens fracture, initial encounter for closed fracture |
| S12.110B | Anterior displaced Type II dens fracture, initial encounter for open fracture |
| S12.110D | Anterior displaced Type II dens fracture, subsequent encounter for fracture with routine healing |
| S12.110G | Anterior displaced Type II dens fracture, subsequent encounter for fracture with delayed healing |
| S12.110K | Anterior displaced Type II dens fracture, subsequent encounter for fracture with nonunion |
| S12.110S | Anterior displaced Type II dens fracture, sequela |
| S12.111 | Posterior displaced Type II dens fracture |
| S12.111A | Posterior displaced Type II dens fracture, initial encounter for closed fracture |
| S12.111B | Posterior displaced Type II dens fracture, initial encounter for open fracture |
| S12.111D | Posterior displaced Type II dens fracture, subsequent encounter for fracture with routine healing |
| S12.111G | Posterior displaced Type II dens fracture, subsequent encounter for fracture with delayed healing |
| S12.111K | Posterior displaced Type II dens fracture, subsequent encounter for fracture with nonunion |
| S12.111S | Posterior displaced Type II dens fracture, sequela |
| S12.112 | Nondisplaced Type II dens fracture |
| S12.112A | Nondisplaced Type II dens fracture, initial encounter for closed fracture |
| S12.112B | Nondisplaced Type II dens fracture, initial encounter for open fracture |
| S12.112D | Nondisplaced Type II dens fracture, subsequent encounter for fracture with routine healing |
| S12.112G | Nondisplaced Type II dens fracture, subsequent encounter for fracture with delayed healing |
| S12.112K | Nondisplaced Type II dens fracture, subsequent encounter for fracture with nonunion |
| S12.112S | Nondisplaced Type II dens fracture, sequela |
| S12.12 | Other dens fracture |
| S12.120 | Other displaced dens fracture |
| S12.120A | Other displaced dens fracture, initial encounter for closed fracture |
| S12.120B | Other displaced dens fracture, initial encounter for open fracture |
| S12.120D | Other displaced dens fracture, subsequent encounter for fracture with routine healing |
| S12.120G | Other displaced dens fracture, subsequent encounter for fracture with delayed healing |
| S12.120K | Other displaced dens fracture, subsequent encounter for fracture with nonunion |
| S12.120S | Other displaced dens fracture, sequela |
| S12.121 | Other nondisplaced dens fracture |
| S12.121A | Other nondisplaced dens fracture, initial encounter for closed fracture |
| S12.121B | Other nondisplaced dens fracture, initial encounter for open fracture |
| S12.121D | Other nondisplaced dens fracture, subsequent encounter for fracture with routine healing |
| S12.121G | Other nondisplaced dens fracture, subsequent encounter for fracture with delayed healing |
| S12.121K | Other nondisplaced dens fracture, subsequent encounter for fracture with nonunion |
| S12.121S | Other nondisplaced dens fracture, sequela |
| S12.13 | Unspecified traumatic spondylolisthesis of second cervical vertebra |
| S12.130 | Unspecified traumatic displaced spondylolisthesis of second cervical vertebra |
| S12.130A | Unspecified traumatic displaced spondylolisthesis of second cervical vertebra, initial encounter for closed fracture |
| S12.130B | Unspecified traumatic displaced spondylolisthesis of second cervical vertebra, initial encounter for open fracture |
| S12.130D | Unspecified traumatic displaced spondylolisthesis of second cervical vertebra, subsequent encounter for fracture with routine healing |
| S12.130G | Unspecified traumatic displaced spondylolisthesis of second cervical vertebra, subsequent encounter for fracture with delayed healing |
| S12.130K | Unspecified traumatic displaced spondylolisthesis of second cervical vertebra, subsequent encounter for fracture with nonunion |
| S12.130S | Unspecified traumatic displaced spondylolisthesis of second cervical vertebra, sequela |
| S12.131 | Unspecified traumatic nondisplaced spondylolisthesis of second cervical vertebra |
| S12.131A | Unspecified traumatic nondisplaced spondylolisthesis of second cervical vertebra, initial encounter for closed fracture |
| S12.131B | Unspecified traumatic nondisplaced spondylolisthesis of second cervical vertebra, initial encounter for open fracture |
| S12.131D | Unspecified traumatic nondisplaced spondylolisthesis of second cervical vertebra, subsequent encounter for fracture with routine healing |
| S12.131G | Unspecified traumatic nondisplaced spondylolisthesis of second cervical vertebra, subsequent encounter for fracture with delayed healing |
| S12.131K | Unspecified traumatic nondisplaced spondylolisthesis of second cervical vertebra, subsequent encounter for fracture with nonunion |
| S12.131S | Unspecified traumatic nondisplaced spondylolisthesis of second cervical vertebra, sequela |
| S12.14 | Type III traumatic spondylolisthesis of second cervical vertebra |
| S12.14XA | Type III traumatic spondylolisthesis of second cervical vertebra, initial encounter for closed fracture |
| S12.14XB | Type III traumatic spondylolisthesis of second cervical vertebra, initial encounter for open fracture |
| S12.14XD | Type III traumatic spondylolisthesis of second cervical vertebra, subsequent encounter for fracture with routine healing |
| S12.14XG | Type III traumatic spondylolisthesis of second cervical vertebra, subsequent encounter for fracture with delayed healing |
| S12.14XK | Type III traumatic spondylolisthesis of second cervical vertebra, subsequent encounter for fracture with nonunion |
| S12.14XS | Type III traumatic spondylolisthesis of second cervical vertebra, sequela |
| S12.15 | Other traumatic spondylolisthesis of second cervical vertebra |
| S12.150 | Other traumatic displaced spondylolisthesis of second cervical vertebra |
| S12.150A | Other traumatic displaced spondylolisthesis of second cervical vertebra, initial encounter for closed fracture |
| S12.150B | Other traumatic displaced spondylolisthesis of second cervical vertebra, initial encounter for open fracture |
| S12.150D | Other traumatic displaced spondylolisthesis of second cervical vertebra, subsequent encounter for fracture with routine healing |
| S12.150G | Other traumatic displaced spondylolisthesis of second cervical vertebra, subsequent encounter for fracture with delayed healing |
| S12.150K | Other traumatic displaced spondylolisthesis of second cervical vertebra, subsequent encounter for fracture with nonunion |
| S12.150S | Other traumatic displaced spondylolisthesis of second cervical vertebra, sequela |
| S12.151 | Other traumatic nondisplaced spondylolisthesis of second cervical vertebra |
| S12.151A | Other traumatic nondisplaced spondylolisthesis of second cervical vertebra, initial encounter for closed fracture |
| S12.151B | Other traumatic nondisplaced spondylolisthesis of second cervical vertebra, initial encounter for open fracture |
| S12.151D | Other traumatic nondisplaced spondylolisthesis of second cervical vertebra, subsequent encounter for fracture with routine healing |
| S12.151G | Other traumatic nondisplaced spondylolisthesis of second cervical vertebra, subsequent encounter for fracture with delayed healing |
| S12.151K | Other traumatic nondisplaced spondylolisthesis of second cervical vertebra, subsequent encounter for fracture with nonunion |
| S12.151S | Other traumatic nondisplaced spondylolisthesis of second cervical vertebra, sequela |
| S12.19 | Other fracture of second cervical vertebra |
| S12.190 | Other displaced fracture of second cervical vertebra |
| S12.190A | Other displaced fracture of second cervical vertebra, initial encounter for closed fracture |
| S12.190B | Other displaced fracture of second cervical vertebra, initial encounter for open fracture |
| S12.190D | Other displaced fracture of second cervical vertebra, subsequent encounter for fracture with routine healing |
| S12.190G | Other displaced fracture of second cervical vertebra, subsequent encounter for fracture with delayed healing |
| S12.190K | Other displaced fracture of second cervical vertebra, subsequent encounter for fracture with nonunion |
| S12.190S | Other displaced fracture of second cervical vertebra, sequela |
| S12.191 | Other nondisplaced fracture of second cervical vertebra |
| S12.191A | Other nondisplaced fracture of second cervical vertebra, initial encounter for closed fracture |
| S12.191B | Other nondisplaced fracture of second cervical vertebra, initial encounter for open fracture |
| S12.191D | Other nondisplaced fracture of second cervical vertebra, subsequent encounter for fracture with routine healing |
| S12.191G | Other nondisplaced fracture of second cervical vertebra, subsequent encounter for fracture with delayed healing |
| S12.191K | Other nondisplaced fracture of second cervical vertebra, subsequent encounter for fracture with nonunion |
| S12.191S | Other nondisplaced fracture of second cervical vertebra, sequela |
| S12.2 | Fracture of third cervical vertebra |
| S12.20 | Unspecified fracture of third cervical vertebra |
| S12.200 | Unspecified displaced fracture of third cervical vertebra |
| S12.200A | Unspecified displaced fracture of third cervical vertebra, initial encounter for closed fracture |
| S12.200B | Unspecified displaced fracture of third cervical vertebra, initial encounter for open fracture |
| S12.200D | Unspecified displaced fracture of third cervical vertebra, subsequent encounter for fracture with routine healing |
| S12.200G | Unspecified displaced fracture of third cervical vertebra, subsequent encounter for fracture with delayed healing |
| S12.200K | Unspecified displaced fracture of third cervical vertebra, subsequent encounter for fracture with nonunion |
| S12.200S | Unspecified displaced fracture of third cervical vertebra, sequela |
| S12.201 | Unspecified nondisplaced fracture of third cervical vertebra |
| S12.201A | Unspecified nondisplaced fracture of third cervical vertebra, initial encounter for closed fracture |
| S12.201B | Unspecified nondisplaced fracture of third cervical vertebra, initial encounter for open fracture |
| S12.201D | Unspecified nondisplaced fracture of third cervical vertebra, subsequent encounter for fracture with routine healing |
| S12.201G | Unspecified nondisplaced fracture of third cervical vertebra, subsequent encounter for fracture with delayed healing |
| S12.201K | Unspecified nondisplaced fracture of third cervical vertebra, subsequent encounter for fracture with nonunion |
| S12.201S | Unspecified nondisplaced fracture of third cervical vertebra, sequela |
| S12.23 | Unspecified traumatic spondylolisthesis of third cervical vertebra |
| S12.230 | Unspecified traumatic displaced spondylolisthesis of third cervical vertebra |
| S12.230A | Unspecified traumatic displaced spondylolisthesis of third cervical vertebra, initial encounter for closed fracture |
| S12.230B | Unspecified traumatic displaced spondylolisthesis of third cervical vertebra, initial encounter for open fracture |
| S12.230D | Unspecified traumatic displaced spondylolisthesis of third cervical vertebra, subsequent encounter for fracture with routine healing |
| S12.230G | Unspecified traumatic displaced spondylolisthesis of third cervical vertebra, subsequent encounter for fracture with delayed healing |
| S12.230K | Unspecified traumatic displaced spondylolisthesis of third cervical vertebra, subsequent encounter for fracture with nonunion |
| S12.230S | Unspecified traumatic displaced spondylolisthesis of third cervical vertebra, sequela |
| S12.231 | Unspecified traumatic nondisplaced spondylolisthesis of third cervical vertebra |
| S12.231A | Unspecified traumatic nondisplaced spondylolisthesis of third cervical vertebra, initial encounter for closed fracture |
| S12.231B | Unspecified traumatic nondisplaced spondylolisthesis of third cervical vertebra, initial encounter for open fracture |
| S12.231D | Unspecified traumatic nondisplaced spondylolisthesis of third cervical vertebra, subsequent encounter for fracture with routine healing |
| S12.231G | Unspecified traumatic nondisplaced spondylolisthesis of third cervical vertebra, subsequent encounter for fracture with delayed healing |
| S12.231K | Unspecified traumatic nondisplaced spondylolisthesis of third cervical vertebra, subsequent encounter for fracture with nonunion |
| S12.231S | Unspecified traumatic nondisplaced spondylolisthesis of third cervical vertebra, sequela |
| S12.24 | Type III traumatic spondylolisthesis of third cervical vertebra |
| S12.24XA | Type III traumatic spondylolisthesis of third cervical vertebra, initial encounter for closed fracture |
| S12.24XB | Type III traumatic spondylolisthesis of third cervical vertebra, initial encounter for open fracture |
| S12.24XD | Type III traumatic spondylolisthesis of third cervical vertebra, subsequent encounter for fracture with routine healing |
| S12.24XG | Type III traumatic spondylolisthesis of third cervical vertebra, subsequent encounter for fracture with delayed healing |
| S12.24XK | Type III traumatic spondylolisthesis of third cervical vertebra, subsequent encounter for fracture with nonunion |
| S12.24XS | Type III traumatic spondylolisthesis of third cervical vertebra, sequela |
| S12.25 | Other traumatic spondylolisthesis of third cervical vertebra |
| S12.250 | Other traumatic displaced spondylolisthesis of third cervical vertebra |
| S12.250A | Other traumatic displaced spondylolisthesis of third cervical vertebra, initial encounter for closed fracture |
| S12.250B | Other traumatic displaced spondylolisthesis of third cervical vertebra, initial encounter for open fracture |
| S12.250D | Other traumatic displaced spondylolisthesis of third cervical vertebra, subsequent encounter for fracture with routine healing |
| S12.250G | Other traumatic displaced spondylolisthesis of third cervical vertebra, subsequent encounter for fracture with delayed healing |
| S12.250K | Other traumatic displaced spondylolisthesis of third cervical vertebra, subsequent encounter for fracture with nonunion |
| S12.250S | Other traumatic displaced spondylolisthesis of third cervical vertebra, sequela |
| S12.251 | Other traumatic nondisplaced spondylolisthesis of third cervical vertebra |
| S12.251A | Other traumatic nondisplaced spondylolisthesis of third cervical vertebra, initial encounter for closed fracture |
| S12.251B | Other traumatic nondisplaced spondylolisthesis of third cervical vertebra, initial encounter for open fracture |
| S12.251D | Other traumatic nondisplaced spondylolisthesis of third cervical vertebra, subsequent encounter for fracture with routine healing |
| S12.251G | Other traumatic nondisplaced spondylolisthesis of third cervical vertebra, subsequent encounter for fracture with delayed healing |
| S12.251K | Other traumatic nondisplaced spondylolisthesis of third cervical vertebra, subsequent encounter for fracture with nonunion |
| S12.251S | Other traumatic nondisplaced spondylolisthesis of third cervical vertebra, sequela |
| S12.29 | Other fracture of third cervical vertebra |
| S12.290 | Other displaced fracture of third cervical vertebra |
| S12.290A | Other displaced fracture of third cervical vertebra, initial encounter for closed fracture |
| S12.290B | Other displaced fracture of third cervical vertebra, initial encounter for open fracture |
| S12.290D | Other displaced fracture of third cervical vertebra, subsequent encounter for fracture with routine healing |
| S12.290G | Other displaced fracture of third cervical vertebra, subsequent encounter for fracture with delayed healing |
| S12.290K | Other displaced fracture of third cervical vertebra, subsequent encounter for fracture with nonunion |
| S12.290S | Other displaced fracture of third cervical vertebra, sequela |
| S12.291 | Other nondisplaced fracture of third cervical vertebra |
| S12.291A | Other nondisplaced fracture of third cervical vertebra, initial encounter for closed fracture |
| S12.291B | Other nondisplaced fracture of third cervical vertebra, initial encounter for open fracture |
| S12.291D | Other nondisplaced fracture of third cervical vertebra, subsequent encounter for fracture with routine healing |
| S12.291G | Other nondisplaced fracture of third cervical vertebra, subsequent encounter for fracture with delayed healing |
| S12.291K | Other nondisplaced fracture of third cervical vertebra, subsequent encounter for fracture with nonunion |
| S12.291S | Other nondisplaced fracture of third cervical vertebra, sequela |
| S12.3 | Fracture of fourth cervical vertebra |
| S12.30 | Unspecified fracture of fourth cervical vertebra |
| S12.300 | Unspecified displaced fracture of fourth cervical vertebra |
| S12.300A | Unspecified displaced fracture of fourth cervical vertebra, initial encounter for closed fracture |
| S12.300B | Unspecified displaced fracture of fourth cervical vertebra, initial encounter for open fracture |
| S12.300D | Unspecified displaced fracture of fourth cervical vertebra, subsequent encounter for fracture with routine healing |
| S12.300G | Unspecified displaced fracture of fourth cervical vertebra, subsequent encounter for fracture with delayed healing |
| S12.300K | Unspecified displaced fracture of fourth cervical vertebra, subsequent encounter for fracture with nonunion |
| S12.300S | Unspecified displaced fracture of fourth cervical vertebra, sequela |
| S12.301 | Unspecified nondisplaced fracture of fourth cervical vertebra |
| S12.301A | Unspecified nondisplaced fracture of fourth cervical vertebra, initial encounter for closed fracture |
| S12.301B | Unspecified nondisplaced fracture of fourth cervical vertebra, initial encounter for open fracture |
| S12.301D | Unspecified nondisplaced fracture of fourth cervical vertebra, subsequent encounter for fracture with routine healing |
| S12.301G | Unspecified nondisplaced fracture of fourth cervical vertebra, subsequent encounter for fracture with delayed healing |
| S12.301K | Unspecified nondisplaced fracture of fourth cervical vertebra, subsequent encounter for fracture with nonunion |
| S12.301S | Unspecified nondisplaced fracture of fourth cervical vertebra, sequela |
| S12.33 | Unspecified traumatic spondylolisthesis of fourth cervical vertebra |
| S12.330 | Unspecified traumatic displaced spondylolisthesis of fourth cervical vertebra |
| S12.330A | Unspecified traumatic displaced spondylolisthesis of fourth cervical vertebra, initial encounter for closed fracture |
| S12.330B | Unspecified traumatic displaced spondylolisthesis of fourth cervical vertebra, initial encounter for open fracture |
| S12.330D | Unspecified traumatic displaced spondylolisthesis of fourth cervical vertebra, subsequent encounter for fracture with routine healing |
| S12.330G | Unspecified traumatic displaced spondylolisthesis of fourth cervical vertebra, subsequent encounter for fracture with delayed healing |
| S12.330K | Unspecified traumatic displaced spondylolisthesis of fourth cervical vertebra, subsequent encounter for fracture with nonunion |
| S12.330S | Unspecified traumatic displaced spondylolisthesis of fourth cervical vertebra, sequela |
| S12.331 | Unspecified traumatic nondisplaced spondylolisthesis of fourth cervical vertebra |
| S12.331A | Unspecified traumatic nondisplaced spondylolisthesis of fourth cervical vertebra, initial encounter for closed fracture |
| S12.331B | Unspecified traumatic nondisplaced spondylolisthesis of fourth cervical vertebra, initial encounter for open fracture |
| S12.331D | Unspecified traumatic nondisplaced spondylolisthesis of fourth cervical vertebra, subsequent encounter for fracture with routine healing |
| S12.331G | Unspecified traumatic nondisplaced spondylolisthesis of fourth cervical vertebra, subsequent encounter for fracture with delayed healing |
| S12.331K | Unspecified traumatic nondisplaced spondylolisthesis of fourth cervical vertebra, subsequent encounter for fracture with nonunion |
| S12.331S | Unspecified traumatic nondisplaced spondylolisthesis of fourth cervical vertebra, sequela |
| S12.34 | Type III traumatic spondylolisthesis of fourth cervical vertebra |
| S12.34XA | Type III traumatic spondylolisthesis of fourth cervical vertebra, initial encounter for closed fracture |
| S12.34XB | Type III traumatic spondylolisthesis of fourth cervical vertebra, initial encounter for open fracture |
| S12.34XD | Type III traumatic spondylolisthesis of fourth cervical vertebra, subsequent encounter for fracture with routine healing |
| S12.34XG | Type III traumatic spondylolisthesis of fourth cervical vertebra, subsequent encounter for fracture with delayed healing |
| S12.34XK | Type III traumatic spondylolisthesis of fourth cervical vertebra, subsequent encounter for fracture with nonunion |
| S12.34XS | Type III traumatic spondylolisthesis of fourth cervical vertebra, sequela |
| S12.35 | Other traumatic spondylolisthesis of fourth cervical vertebra |
| S12.350 | Other traumatic displaced spondylolisthesis of fourth cervical vertebra |
| S12.350A | Other traumatic displaced spondylolisthesis of fourth cervical vertebra, initial encounter for closed fracture |
| S12.350B | Other traumatic displaced spondylolisthesis of fourth cervical vertebra, initial encounter for open fracture |
| S12.350D | Other traumatic displaced spondylolisthesis of fourth cervical vertebra, subsequent encounter for fracture with routine healing |
| S12.350G | Other traumatic displaced spondylolisthesis of fourth cervical vertebra, subsequent encounter for fracture with delayed healing |
| S12.350K | Other traumatic displaced spondylolisthesis of fourth cervical vertebra, subsequent encounter for fracture with nonunion |
| S12.350S | Other traumatic displaced spondylolisthesis of fourth cervical vertebra, sequela |
| S12.351 | Other traumatic nondisplaced spondylolisthesis of fourth cervical vertebra |
| S12.351A | Other traumatic nondisplaced spondylolisthesis of fourth cervical vertebra, initial encounter for closed fracture |
| S12.351B | Other traumatic nondisplaced spondylolisthesis of fourth cervical vertebra, initial encounter for open fracture |
| S12.351D | Other traumatic nondisplaced spondylolisthesis of fourth cervical vertebra, subsequent encounter for fracture with routine healing |
| S12.351G | Other traumatic nondisplaced spondylolisthesis of fourth cervical vertebra, subsequent encounter for fracture with delayed healing |
| S12.351K | Other traumatic nondisplaced spondylolisthesis of fourth cervical vertebra, subsequent encounter for fracture with nonunion |
| S12.351S | Other traumatic nondisplaced spondylolisthesis of fourth cervical vertebra, sequela |
| S12.39 | Other fracture of fourth cervical vertebra |
| S12.390 | Other displaced fracture of fourth cervical vertebra |
| S12.390A | Other displaced fracture of fourth cervical vertebra, initial encounter for closed fracture |
| S12.390B | Other displaced fracture of fourth cervical vertebra, initial encounter for open fracture |
| S12.390D | Other displaced fracture of fourth cervical vertebra, subsequent encounter for fracture with routine healing |
| S12.390G | Other displaced fracture of fourth cervical vertebra, subsequent encounter for fracture with delayed healing |
| S12.390K | Other displaced fracture of fourth cervical vertebra, subsequent encounter for fracture with nonunion |
| S12.390S | Other displaced fracture of fourth cervical vertebra, sequela |
| S12.391 | Other nondisplaced fracture of fourth cervical vertebra |
| S12.391A | Other nondisplaced fracture of fourth cervical vertebra, initial encounter for closed fracture |
| S12.391B | Other nondisplaced fracture of fourth cervical vertebra, initial encounter for open fracture |
| S12.391D | Other nondisplaced fracture of fourth cervical vertebra, subsequent encounter for fracture with routine healing |
| S12.391G | Other nondisplaced fracture of fourth cervical vertebra, subsequent encounter for fracture with delayed healing |
| S12.391K | Other nondisplaced fracture of fourth cervical vertebra, subsequent encounter for fracture with nonunion |
| S12.391S | Other nondisplaced fracture of fourth cervical vertebra, sequela |
| S12.4 | Fracture of fifth cervical vertebra |
| S12.40 | Unspecified fracture of fifth cervical vertebra |
| S12.400 | Unspecified displaced fracture of fifth cervical vertebra |
| S12.400A | Unspecified displaced fracture of fifth cervical vertebra, initial encounter for closed fracture |
| S12.400B | Unspecified displaced fracture of fifth cervical vertebra, initial encounter for open fracture |
| S12.400D | Unspecified displaced fracture of fifth cervical vertebra, subsequent encounter for fracture with routine healing |
| S12.400G | Unspecified displaced fracture of fifth cervical vertebra, subsequent encounter for fracture with delayed healing |
| S12.400K | Unspecified displaced fracture of fifth cervical vertebra, subsequent encounter for fracture with nonunion |
| S12.400S | Unspecified displaced fracture of fifth cervical vertebra, sequela |
| S12.401 | Unspecified nondisplaced fracture of fifth cervical vertebra |
| S12.401A | Unspecified nondisplaced fracture of fifth cervical vertebra, initial encounter for closed fracture |
| S12.401B | Unspecified nondisplaced fracture of fifth cervical vertebra, initial encounter for open fracture |
| S12.401D | Unspecified nondisplaced fracture of fifth cervical vertebra, subsequent encounter for fracture with routine healing |
| S12.401G | Unspecified nondisplaced fracture of fifth cervical vertebra, subsequent encounter for fracture with delayed healing |
| S12.401K | Unspecified nondisplaced fracture of fifth cervical vertebra, subsequent encounter for fracture with nonunion |
| S12.401S | Unspecified nondisplaced fracture of fifth cervical vertebra, sequela |
| S12.43 | Unspecified traumatic spondylolisthesis of fifth cervical vertebra |
| S12.430 | Unspecified traumatic displaced spondylolisthesis of fifth cervical vertebra |
| S12.430A | Unspecified traumatic displaced spondylolisthesis of fifth cervical vertebra, initial encounter for closed fracture |
| S12.430B | Unspecified traumatic displaced spondylolisthesis of fifth cervical vertebra, initial encounter for open fracture |
| S12.430D | Unspecified traumatic displaced spondylolisthesis of fifth cervical vertebra, subsequent encounter for fracture with routine healing |
| S12.430G | Unspecified traumatic displaced spondylolisthesis of fifth cervical vertebra, subsequent encounter for fracture with delayed healing |
| S12.430K | Unspecified traumatic displaced spondylolisthesis of fifth cervical vertebra, subsequent encounter for fracture with nonunion |
| S12.430S | Unspecified traumatic displaced spondylolisthesis of fifth cervical vertebra, sequela |
| S12.431 | Unspecified traumatic nondisplaced spondylolisthesis of fifth cervical vertebra |
| S12.431A | Unspecified traumatic nondisplaced spondylolisthesis of fifth cervical vertebra, initial encounter for closed fracture |
| S12.431B | Unspecified traumatic nondisplaced spondylolisthesis of fifth cervical vertebra, initial encounter for open fracture |
| S12.431D | Unspecified traumatic nondisplaced spondylolisthesis of fifth cervical vertebra, subsequent encounter for fracture with routine healing |
| S12.431G | Unspecified traumatic nondisplaced spondylolisthesis of fifth cervical vertebra, subsequent encounter for fracture with delayed healing |
| S12.431K | Unspecified traumatic nondisplaced spondylolisthesis of fifth cervical vertebra, subsequent encounter for fracture with nonunion |
| S12.431S | Unspecified traumatic nondisplaced spondylolisthesis of fifth cervical vertebra, sequela |
| S12.44 | Type III traumatic spondylolisthesis of fifth cervical vertebra |
| S12.44XA | Type III traumatic spondylolisthesis of fifth cervical vertebra, initial encounter for closed fracture |
| S12.44XB | Type III traumatic spondylolisthesis of fifth cervical vertebra, initial encounter for open fracture |
| S12.44XD | Type III traumatic spondylolisthesis of fifth cervical vertebra, subsequent encounter for fracture with routine healing |
| S12.44XG | Type III traumatic spondylolisthesis of fifth cervical vertebra, subsequent encounter for fracture with delayed healing |
| S12.44XK | Type III traumatic spondylolisthesis of fifth cervical vertebra, subsequent encounter for fracture with nonunion |
| S12.44XS | Type III traumatic spondylolisthesis of fifth cervical vertebra, sequela |
| S12.45 | Other traumatic spondylolisthesis of fifth cervical vertebra |
| S12.450 | Other traumatic displaced spondylolisthesis of fifth cervical vertebra |
| S12.450A | Other traumatic displaced spondylolisthesis of fifth cervical vertebra, initial encounter for closed fracture |
| S12.450B | Other traumatic displaced spondylolisthesis of fifth cervical vertebra, initial encounter for open fracture |
| S12.450D | Other traumatic displaced spondylolisthesis of fifth cervical vertebra, subsequent encounter for fracture with routine healing |
| S12.450G | Other traumatic displaced spondylolisthesis of fifth cervical vertebra, subsequent encounter for fracture with delayed healing |
| S12.450K | Other traumatic displaced spondylolisthesis of fifth cervical vertebra, subsequent encounter for fracture with nonunion |
| S12.450S | Other traumatic displaced spondylolisthesis of fifth cervical vertebra, sequela |
| S12.451 | Other traumatic nondisplaced spondylolisthesis of fifth cervical vertebra |
| S12.451A | Other traumatic nondisplaced spondylolisthesis of fifth cervical vertebra, initial encounter for closed fracture |
| S12.451B | Other traumatic nondisplaced spondylolisthesis of fifth cervical vertebra, initial encounter for open fracture |
| S12.451D | Other traumatic nondisplaced spondylolisthesis of fifth cervical vertebra, subsequent encounter for fracture with routine healing |
| S12.451G | Other traumatic nondisplaced spondylolisthesis of fifth cervical vertebra, subsequent encounter for fracture with delayed healing |
| S12.451K | Other traumatic nondisplaced spondylolisthesis of fifth cervical vertebra, subsequent encounter for fracture with nonunion |
| S12.451S | Other traumatic nondisplaced spondylolisthesis of fifth cervical vertebra, sequela |
| S12.49 | Other fracture of fifth cervical vertebra |
| S12.490 | Other displaced fracture of fifth cervical vertebra |
| S12.490A | Other displaced fracture of fifth cervical vertebra, initial encounter for closed fracture |
| S12.490B | Other displaced fracture of fifth cervical vertebra, initial encounter for open fracture |
| S12.490D | Other displaced fracture of fifth cervical vertebra, subsequent encounter for fracture with routine healing |
| S12.490G | Other displaced fracture of fifth cervical vertebra, subsequent encounter for fracture with delayed healing |
| S12.490K | Other displaced fracture of fifth cervical vertebra, subsequent encounter for fracture with nonunion |
| S12.490S | Other displaced fracture of fifth cervical vertebra, sequela |
| S12.491 | Other nondisplaced fracture of fifth cervical vertebra |
| S12.491A | Other nondisplaced fracture of fifth cervical vertebra, initial encounter for closed fracture |
| S12.491B | Other nondisplaced fracture of fifth cervical vertebra, initial encounter for open fracture |
| S12.491D | Other nondisplaced fracture of fifth cervical vertebra, subsequent encounter for fracture with routine healing |
| S12.491G | Other nondisplaced fracture of fifth cervical vertebra, subsequent encounter for fracture with delayed healing |
| S12.491K | Other nondisplaced fracture of fifth cervical vertebra, subsequent encounter for fracture with nonunion |
| S12.491S | Other nondisplaced fracture of fifth cervical vertebra, sequela |
| S12.5 | Fracture of sixth cervical vertebra |
| S12.50 | Unspecified fracture of sixth cervical vertebra |
| S12.500 | Unspecified displaced fracture of sixth cervical vertebra |
| S12.500A | Unspecified displaced fracture of sixth cervical vertebra, initial encounter for closed fracture |
| S12.500B | Unspecified displaced fracture of sixth cervical vertebra, initial encounter for open fracture |
| S12.500D | Unspecified displaced fracture of sixth cervical vertebra, subsequent encounter for fracture with routine healing |
| S12.500G | Unspecified displaced fracture of sixth cervical vertebra, subsequent encounter for fracture with delayed healing |
| S12.500K | Unspecified displaced fracture of sixth cervical vertebra, subsequent encounter for fracture with nonunion |
| S12.500S | Unspecified displaced fracture of sixth cervical vertebra, sequela |
| S12.501 | Unspecified nondisplaced fracture of sixth cervical vertebra |
| S12.501A | Unspecified nondisplaced fracture of sixth cervical vertebra, initial encounter for closed fracture |
| S12.501B | Unspecified nondisplaced fracture of sixth cervical vertebra, initial encounter for open fracture |
| S12.501D | Unspecified nondisplaced fracture of sixth cervical vertebra, subsequent encounter for fracture with routine healing |
| S12.501G | Unspecified nondisplaced fracture of sixth cervical vertebra, subsequent encounter for fracture with delayed healing |
| S12.501K | Unspecified nondisplaced fracture of sixth cervical vertebra, subsequent encounter for fracture with nonunion |
| S12.501S | Unspecified nondisplaced fracture of sixth cervical vertebra, sequela |
| S12.53 | Unspecified traumatic spondylolisthesis of sixth cervical vertebra |
| S12.530 | Unspecified traumatic displaced spondylolisthesis of sixth cervical vertebra |
| S12.530A | Unspecified traumatic displaced spondylolisthesis of sixth cervical vertebra, initial encounter for closed fracture |
| S12.530B | Unspecified traumatic displaced spondylolisthesis of sixth cervical vertebra, initial encounter for open fracture |
| S12.530D | Unspecified traumatic displaced spondylolisthesis of sixth cervical vertebra, subsequent encounter for fracture with routine healing |
| S12.530G | Unspecified traumatic displaced spondylolisthesis of sixth cervical vertebra, subsequent encounter for fracture with delayed healing |
| S12.530K | Unspecified traumatic displaced spondylolisthesis of sixth cervical vertebra, subsequent encounter for fracture with nonunion |
| S12.530S | Unspecified traumatic displaced spondylolisthesis of sixth cervical vertebra, sequela |
| S12.531 | Unspecified traumatic nondisplaced spondylolisthesis of sixth cervical vertebra |
| S12.531A | Unspecified traumatic nondisplaced spondylolisthesis of sixth cervical vertebra, initial encounter for closed fracture |
| S12.531B | Unspecified traumatic nondisplaced spondylolisthesis of sixth cervical vertebra, initial encounter for open fracture |
| S12.531D | Unspecified traumatic nondisplaced spondylolisthesis of sixth cervical vertebra, subsequent encounter for fracture with routine healing |
| S12.531G | Unspecified traumatic nondisplaced spondylolisthesis of sixth cervical vertebra, subsequent encounter for fracture with delayed healing |
| S12.531K | Unspecified traumatic nondisplaced spondylolisthesis of sixth cervical vertebra, subsequent encounter for fracture with nonunion |
| S12.531S | Unspecified traumatic nondisplaced spondylolisthesis of sixth cervical vertebra, sequela |
| S12.54 | Type III traumatic spondylolisthesis of sixth cervical vertebra |
| S12.54XA | Type III traumatic spondylolisthesis of sixth cervical vertebra, initial encounter for closed fracture |
| S12.54XB | Type III traumatic spondylolisthesis of sixth cervical vertebra, initial encounter for open fracture |
| S12.54XD | Type III traumatic spondylolisthesis of sixth cervical vertebra, subsequent encounter for fracture with routine healing |
| S12.54XG | Type III traumatic spondylolisthesis of sixth cervical vertebra, subsequent encounter for fracture with delayed healing |
| S12.54XK | Type III traumatic spondylolisthesis of sixth cervical vertebra, subsequent encounter for fracture with nonunion |
| S12.54XS | Type III traumatic spondylolisthesis of sixth cervical vertebra, sequela |
| S12.55 | Other traumatic spondylolisthesis of sixth cervical vertebra |
| S12.550 | Other traumatic displaced spondylolisthesis of sixth cervical vertebra |
| S12.550A | Other traumatic displaced spondylolisthesis of sixth cervical vertebra, initial encounter for closed fracture |
| S12.550B | Other traumatic displaced spondylolisthesis of sixth cervical vertebra, initial encounter for open fracture |
| S12.550D | Other traumatic displaced spondylolisthesis of sixth cervical vertebra, subsequent encounter for fracture with routine healing |
| S12.550G | Other traumatic displaced spondylolisthesis of sixth cervical vertebra, subsequent encounter for fracture with delayed healing |
| S12.550K | Other traumatic displaced spondylolisthesis of sixth cervical vertebra, subsequent encounter for fracture with nonunion |
| S12.550S | Other traumatic displaced spondylolisthesis of sixth cervical vertebra, sequela |
| S12.551 | Other traumatic nondisplaced spondylolisthesis of sixth cervical vertebra |
| S12.551A | Other traumatic nondisplaced spondylolisthesis of sixth cervical vertebra, initial encounter for closed fracture |
| S12.551B | Other traumatic nondisplaced spondylolisthesis of sixth cervical vertebra, initial encounter for open fracture |
| S12.551D | Other traumatic nondisplaced spondylolisthesis of sixth cervical vertebra, subsequent encounter for fracture with routine healing |
| S12.551G | Other traumatic nondisplaced spondylolisthesis of sixth cervical vertebra, subsequent encounter for fracture with delayed healing |
| S12.551K | Other traumatic nondisplaced spondylolisthesis of sixth cervical vertebra, subsequent encounter for fracture with nonunion |
| S12.551S | Other traumatic nondisplaced spondylolisthesis of sixth cervical vertebra, sequela |
| S12.59 | Other fracture of sixth cervical vertebra |
| S12.590 | Other displaced fracture of sixth cervical vertebra |
| S12.590A | Other displaced fracture of sixth cervical vertebra, initial encounter for closed fracture |
| S12.590B | Other displaced fracture of sixth cervical vertebra, initial encounter for open fracture |
| S12.590D | Other displaced fracture of sixth cervical vertebra, subsequent encounter for fracture with routine healing |
| S12.590G | Other displaced fracture of sixth cervical vertebra, subsequent encounter for fracture with delayed healing |
| S12.590K | Other displaced fracture of sixth cervical vertebra, subsequent encounter for fracture with nonunion |
| S12.590S | Other displaced fracture of sixth cervical vertebra, sequela |
| S12.591 | Other nondisplaced fracture of sixth cervical vertebra |
| S12.591A | Other nondisplaced fracture of sixth cervical vertebra, initial encounter for closed fracture |
| S12.591B | Other nondisplaced fracture of sixth cervical vertebra, initial encounter for open fracture |
| S12.591D | Other nondisplaced fracture of sixth cervical vertebra, subsequent encounter for fracture with routine healing |
| S12.591G | Other nondisplaced fracture of sixth cervical vertebra, subsequent encounter for fracture with delayed healing |
| S12.591K | Other nondisplaced fracture of sixth cervical vertebra, subsequent encounter for fracture with nonunion |
| S12.591S | Other nondisplaced fracture of sixth cervical vertebra, sequela |
| S12.6 | Fracture of seventh cervical vertebra |
| S12.60 | Unspecified fracture of seventh cervical vertebra |
| S12.600 | Unspecified displaced fracture of seventh cervical vertebra |
| S12.600A | Unspecified displaced fracture of seventh cervical vertebra, initial encounter for closed fracture |
| S12.600B | Unspecified displaced fracture of seventh cervical vertebra, initial encounter for open fracture |
| S12.600D | Unspecified displaced fracture of seventh cervical vertebra, subsequent encounter for fracture with routine healing |
| S12.600G | Unspecified displaced fracture of seventh cervical vertebra, subsequent encounter for fracture with delayed healing |
| S12.600K | Unspecified displaced fracture of seventh cervical vertebra, subsequent encounter for fracture with nonunion |
| S12.600S | Unspecified displaced fracture of seventh cervical vertebra, sequela |
| S12.601 | Unspecified nondisplaced fracture of seventh cervical vertebra |
| S12.601A | Unspecified nondisplaced fracture of seventh cervical vertebra, initial encounter for closed fracture |
| S12.601B | Unspecified nondisplaced fracture of seventh cervical vertebra, initial encounter for open fracture |
| S12.601D | Unspecified nondisplaced fracture of seventh cervical vertebra, subsequent encounter for fracture with routine healing |
| S12.601G | Unspecified nondisplaced fracture of seventh cervical vertebra, subsequent encounter for fracture with delayed healing |
| S12.601K | Unspecified nondisplaced fracture of seventh cervical vertebra, subsequent encounter for fracture with nonunion |
| S12.601S | Unspecified nondisplaced fracture of seventh cervical vertebra, sequela |
| S12.63 | Unspecified traumatic spondylolisthesis of seventh cervical vertebra |
| S12.630 | Unspecified traumatic displaced spondylolisthesis of seventh cervical vertebra |
| S12.630A | Unspecified traumatic displaced spondylolisthesis of seventh cervical vertebra, initial encounter for closed fracture |
| S12.630B | Unspecified traumatic displaced spondylolisthesis of seventh cervical vertebra, initial encounter for open fracture |
| S12.630D | Unspecified traumatic displaced spondylolisthesis of seventh cervical vertebra, subsequent encounter for fracture with routine healing |
| S12.630G | Unspecified traumatic displaced spondylolisthesis of seventh cervical vertebra, subsequent encounter for fracture with delayed healing |
| S12.630K | Unspecified traumatic displaced spondylolisthesis of seventh cervical vertebra, subsequent encounter for fracture with nonunion |
| S12.630S | Unspecified traumatic displaced spondylolisthesis of seventh cervical vertebra, sequela |
| S12.631 | Unspecified traumatic nondisplaced spondylolisthesis of seventh cervical vertebra |
| S12.631A | Unspecified traumatic nondisplaced spondylolisthesis of seventh cervical vertebra, initial encounter for closed fracture |
| S12.631B | Unspecified traumatic nondisplaced spondylolisthesis of seventh cervical vertebra, initial encounter for open fracture |
| S12.631D | Unspecified traumatic nondisplaced spondylolisthesis of seventh cervical vertebra, subsequent encounter for fracture with routine healing |
| S12.631G | Unspecified traumatic nondisplaced spondylolisthesis of seventh cervical vertebra, subsequent encounter for fracture with delayed healing |
| S12.631K | Unspecified traumatic nondisplaced spondylolisthesis of seventh cervical vertebra, subsequent encounter for fracture with nonunion |
| S12.631S | Unspecified traumatic nondisplaced spondylolisthesis of seventh cervical vertebra, sequela |
| S12.64 | Type III traumatic spondylolisthesis of seventh cervical vertebra |
| S12.64XA | Type III traumatic spondylolisthesis of seventh cervical vertebra, initial encounter for closed fracture |
| S12.64XB | Type III traumatic spondylolisthesis of seventh cervical vertebra, initial encounter for open fracture |
| S12.64XD | Type III traumatic spondylolisthesis of seventh cervical vertebra, subsequent encounter for fracture with routine healing |
| S12.64XG | Type III traumatic spondylolisthesis of seventh cervical vertebra, subsequent encounter for fracture with delayed healing |
| S12.64XK | Type III traumatic spondylolisthesis of seventh cervical vertebra, subsequent encounter for fracture with nonunion |
| S12.64XS | Type III traumatic spondylolisthesis of seventh cervical vertebra, sequela |
| S12.65 | Other traumatic spondylolisthesis of seventh cervical vertebra |
| S12.650 | Other traumatic displaced spondylolisthesis of seventh cervical vertebra |
| S12.650A | Other traumatic displaced spondylolisthesis of seventh cervical vertebra, initial encounter for closed fracture |
| S12.650B | Other traumatic displaced spondylolisthesis of seventh cervical vertebra, initial encounter for open fracture |
| S12.650D | Other traumatic displaced spondylolisthesis of seventh cervical vertebra, subsequent encounter for fracture with routine healing |
| S12.650G | Other traumatic displaced spondylolisthesis of seventh cervical vertebra, subsequent encounter for fracture with delayed healing |
| S12.650K | Other traumatic displaced spondylolisthesis of seventh cervical vertebra, subsequent encounter for fracture with nonunion |
| S12.650S | Other traumatic displaced spondylolisthesis of seventh cervical vertebra, sequela |
| S12.651 | Other traumatic nondisplaced spondylolisthesis of seventh cervical vertebra |
| S12.651A | Other traumatic nondisplaced spondylolisthesis of seventh cervical vertebra, initial encounter for closed fracture |
| S12.651B | Other traumatic nondisplaced spondylolisthesis of seventh cervical vertebra, initial encounter for open fracture |
| S12.651D | Other traumatic nondisplaced spondylolisthesis of seventh cervical vertebra, subsequent encounter for fracture with routine healing |
| S12.651G | Other traumatic nondisplaced spondylolisthesis of seventh cervical vertebra, subsequent encounter for fracture with delayed healing |
| S12.651K | Other traumatic nondisplaced spondylolisthesis of seventh cervical vertebra, subsequent encounter for fracture with nonunion |
| S12.651S | Other traumatic nondisplaced spondylolisthesis of seventh cervical vertebra, sequela |
| S12.69 | Other fracture of seventh cervical vertebra |
| S12.690 | Other displaced fracture of seventh cervical vertebra |
| S12.690A | Other displaced fracture of seventh cervical vertebra, initial encounter for closed fracture |
| S12.690B | Other displaced fracture of seventh cervical vertebra, initial encounter for open fracture |
| S12.690D | Other displaced fracture of seventh cervical vertebra, subsequent encounter for fracture with routine healing |
| S12.690G | Other displaced fracture of seventh cervical vertebra, subsequent encounter for fracture with delayed healing |
| S12.690K | Other displaced fracture of seventh cervical vertebra, subsequent encounter for fracture with nonunion |
| S12.690S | Other displaced fracture of seventh cervical vertebra, sequela |
| S12.691 | Other nondisplaced fracture of seventh cervical vertebra |
| S12.691A | Other nondisplaced fracture of seventh cervical vertebra, initial encounter for closed fracture |
| S12.691B | Other nondisplaced fracture of seventh cervical vertebra, initial encounter for open fracture |
| S12.691D | Other nondisplaced fracture of seventh cervical vertebra, subsequent encounter for fracture with routine healing |
| S12.691G | Other nondisplaced fracture of seventh cervical vertebra, subsequent encounter for fracture with delayed healing |
| S12.691K | Other nondisplaced fracture of seventh cervical vertebra, subsequent encounter for fracture with nonunion |
| S12.691S | Other nondisplaced fracture of seventh cervical vertebra, sequela |
| S12.8 | Fracture of other parts of neck |
| S12.8XXA | Fracture of other parts of neck, initial encounter |
| S12.8XXD | Fracture of other parts of neck, subsequent encounter |
| S12.8XXS | Fracture of other parts of neck, sequela |
| S12.9 | Fracture of neck, unspecified |
| S12.9XXA | Fracture of neck, unspecified, initial encounter |
| S12.9XXD | Fracture of neck, unspecified, subsequent encounter |
| S12.9XXS | Fracture of neck, unspecified, sequela |
| S13 | Dislocation and sprain of joints and ligaments at neck level |
| S13.0 | Traumatic rupture of cervical intervertebral disc |
| S13.0XXA | Traumatic rupture of cervical intervertebral disc, initial encounter |
| S13.0XXD | Traumatic rupture of cervical intervertebral disc, subsequent encounter |
| S13.0XXS | Traumatic rupture of cervical intervertebral disc, sequela |
| S13.1 | Subluxation and dislocation of cervical vertebrae |
| S13.10 | Subluxation and dislocation of unspecified cervical vertebrae |
| S13.100 | Subluxation of unspecified cervical vertebrae |
| S13.100A | Subluxation of unspecified cervical vertebrae, initial encounter |
| S13.100D | Subluxation of unspecified cervical vertebrae, subsequent encounter |
| S13.100S | Subluxation of unspecified cervical vertebrae, sequela |
| S13.101 | Dislocation of unspecified cervical vertebrae |
| S13.101A | Dislocation of unspecified cervical vertebrae, initial encounter |
| S13.101D | Dislocation of unspecified cervical vertebrae, subsequent encounter |
| S13.101S | Dislocation of unspecified cervical vertebrae, sequela |
| S13.11 | Subluxation and dislocation of C0/C1 cervical vertebrae |
| S13.110 | Subluxation of C0/C1 cervical vertebrae |
| S13.110A | Subluxation of C0/C1 cervical vertebrae, initial encounter |
| S13.110D | Subluxation of C0/C1 cervical vertebrae, subsequent encounter |
| S13.110S | Subluxation of C0/C1 cervical vertebrae, sequela |
| S13.111 | Dislocation of C0/C1 cervical vertebrae |
| S13.111A | Dislocation of C0/C1 cervical vertebrae, initial encounter |
| S13.111D | Dislocation of C0/C1 cervical vertebrae, subsequent encounter |
| S13.111S | Dislocation of C0/C1 cervical vertebrae, sequela |
| S13.12 | Subluxation and dislocation of C1/C2 cervical vertebrae |
| S13.120 | Subluxation of C1/C2 cervical vertebrae |
| S13.120A | Subluxation of C1/C2 cervical vertebrae, initial encounter |
| S13.120D | Subluxation of C1/C2 cervical vertebrae, subsequent encounter |
| S13.120S | Subluxation of C1/C2 cervical vertebrae, sequela |
| S13.121 | Dislocation of C1/C2 cervical vertebrae |
| S13.121A | Dislocation of C1/C2 cervical vertebrae, initial encounter |
| S13.121D | Dislocation of C1/C2 cervical vertebrae, subsequent encounter |
| S13.121S | Dislocation of C1/C2 cervical vertebrae, sequela |
| S13.13 | Subluxation and dislocation of C2/C3 cervical vertebrae |
| S13.130 | Subluxation of C2/C3 cervical vertebrae |
| S13.130A | Subluxation of C2/C3 cervical vertebrae, initial encounter |
| S13.130D | Subluxation of C2/C3 cervical vertebrae, subsequent encounter |
| S13.130S | Subluxation of C2/C3 cervical vertebrae, sequela |
| S13.131 | Dislocation of C2/C3 cervical vertebrae |
| S13.131A | Dislocation of C2/C3 cervical vertebrae, initial encounter |
| S13.131D | Dislocation of C2/C3 cervical vertebrae, subsequent encounter |
| S13.131S | Dislocation of C2/C3 cervical vertebrae, sequela |
| S13.14 | Subluxation and dislocation of C3/C4 cervical vertebrae |
| S13.140 | Subluxation of C3/C4 cervical vertebrae |
| S13.140A | Subluxation of C3/C4 cervical vertebrae, initial encounter |
| S13.140D | Subluxation of C3/C4 cervical vertebrae, subsequent encounter |
| S13.140S | Subluxation of C3/C4 cervical vertebrae, sequela |
| S13.141 | Dislocation of C3/C4 cervical vertebrae |
| S13.141A | Dislocation of C3/C4 cervical vertebrae, initial encounter |
| S13.141D | Dislocation of C3/C4 cervical vertebrae, subsequent encounter |
| S13.141S | Dislocation of C3/C4 cervical vertebrae, sequela |
| S13.15 | Subluxation and dislocation of C4/C5 cervical vertebrae |
| S13.150 | Subluxation of C4/C5 cervical vertebrae |
| S13.150A | Subluxation of C4/C5 cervical vertebrae, initial encounter |
| S13.150D | Subluxation of C4/C5 cervical vertebrae, subsequent encounter |
| S13.150S | Subluxation of C4/C5 cervical vertebrae, sequela |
| S13.151 | Dislocation of C4/C5 cervical vertebrae |
| S13.151A | Dislocation of C4/C5 cervical vertebrae, initial encounter |
| S13.151D | Dislocation of C4/C5 cervical vertebrae, subsequent encounter |
| S13.151S | Dislocation of C4/C5 cervical vertebrae, sequela |
| S13.16 | Subluxation and dislocation of C5/C6 cervical vertebrae |
| S13.160 | Subluxation of C5/C6 cervical vertebrae |
| S13.160A | Subluxation of C5/C6 cervical vertebrae, initial encounter |
| S13.160D | Subluxation of C5/C6 cervical vertebrae, subsequent encounter |
| S13.160S | Subluxation of C5/C6 cervical vertebrae, sequela |
| S13.161 | Dislocation of C5/C6 cervical vertebrae |
| S13.161A | Dislocation of C5/C6 cervical vertebrae, initial encounter |
| S13.161D | Dislocation of C5/C6 cervical vertebrae, subsequent encounter |
| S13.161S | Dislocation of C5/C6 cervical vertebrae, sequela |
| S13.17 | Subluxation and dislocation of C6/C7 cervical vertebrae |
| S13.170 | Subluxation of C6/C7 cervical vertebrae |
| S13.170A | Subluxation of C6/C7 cervical vertebrae, initial encounter |
| S13.170D | Subluxation of C6/C7 cervical vertebrae, subsequent encounter |
| S13.170S | Subluxation of C6/C7 cervical vertebrae, sequela |
| S13.171 | Dislocation of C6/C7 cervical vertebrae |
| S13.171A | Dislocation of C6/C7 cervical vertebrae, initial encounter |
| S13.171D | Dislocation of C6/C7 cervical vertebrae, subsequent encounter |
| S13.171S | Dislocation of C6/C7 cervical vertebrae, sequela |
| S13.18 | Subluxation and dislocation of C7/T1 cervical vertebrae |
| S13.180 | Subluxation of C7/T1 cervical vertebrae |
| S13.180A | Subluxation of C7/T1 cervical vertebrae, initial encounter |
| S13.180D | Subluxation of C7/T1 cervical vertebrae, subsequent encounter |
| S13.180S | Subluxation of C7/T1 cervical vertebrae, sequela |
| S13.181 | Dislocation of C7/T1 cervical vertebrae |
| S13.181A | Dislocation of C7/T1 cervical vertebrae, initial encounter |
| S13.181D | Dislocation of C7/T1 cervical vertebrae, subsequent encounter |
| S13.181S | Dislocation of C7/T1 cervical vertebrae, sequela |
| S13.2 | Dislocation of other and unspecified parts of neck |
| S13.20 | Dislocation of unspecified parts of neck |
| S13.20XA | Dislocation of unspecified parts of neck, initial encounter |
| S13.20XD | Dislocation of unspecified parts of neck, subsequent encounter |
| S13.20XS | Dislocation of unspecified parts of neck, sequela |
| S13.29 | Dislocation of other parts of neck |
| S13.29XA | Dislocation of other parts of neck, initial encounter |
| S13.29XD | Dislocation of other parts of neck, subsequent encounter |
| S13.29XS | Dislocation of other parts of neck, sequela |
| S13.4 | Sprain of ligaments of cervical spine |
| S13.4XXA | Sprain of ligaments of cervical spine, initial encounter |
| S13.4XXD | Sprain of ligaments of cervical spine, subsequent encounter |
| S13.4XXS | Sprain of ligaments of cervical spine, sequela |
| S13.5 | Sprain of thyroid region |
| S13.5XXA | Sprain of thyroid region, initial encounter |
| S13.5XXD | Sprain of thyroid region, subsequent encounter |
| S13.5XXS | Sprain of thyroid region, sequela |
| S13.8 | Sprain of joints and ligaments of other parts of neck |
| S13.8XXA | Sprain of joints and ligaments of other parts of neck, initial encounter |
| S13.8XXD | Sprain of joints and ligaments of other parts of neck, subsequent encounter |
| S13.8XXS | Sprain of joints and ligaments of other parts of neck, sequela |
| S13.9 | Sprain of joints and ligaments of unspecified parts of neck |
| S13.9XXA | Sprain of joints and ligaments of unspecified parts of neck, initial encounter |
| S13.9XXD | Sprain of joints and ligaments of unspecified parts of neck, subsequent encounter |
| S13.9XXS | Sprain of joints and ligaments of unspecified parts of neck, sequela |
| S14 | Injury of nerves and spinal cord at neck level |
| S14.0 | Concussion and edema of cervical spinal cord |
| S14.0XXA | Concussion and edema of cervical spinal cord, initial encounter |
| S14.0XXD | Concussion and edema of cervical spinal cord, subsequent encounter |
| S14.0XXS | Concussion and edema of cervical spinal cord, sequela |
| S14.1 | Other and unspecified injuries of cervical spinal cord |
| S14.10 | Unspecified injury of cervical spinal cord |
| S14.101 | Unspecified injury at C1 level of cervical spinal cord |
| S14.101A | Unspecified injury at C1 level of cervical spinal cord, initial encounter |
| S14.101D | Unspecified injury at C1 level of cervical spinal cord, subsequent encounter |
| S14.101S | Unspecified injury at C1 level of cervical spinal cord, sequela |
| S14.102 | Unspecified injury at C2 level of cervical spinal cord |
| S14.102A | Unspecified injury at C2 level of cervical spinal cord, initial encounter |
| S14.102D | Unspecified injury at C2 level of cervical spinal cord, subsequent encounter |
| S14.102S | Unspecified injury at C2 level of cervical spinal cord, sequela |
| S14.103 | Unspecified injury at C3 level of cervical spinal cord |
| S14.103A | Unspecified injury at C3 level of cervical spinal cord, initial encounter |
| S14.103D | Unspecified injury at C3 level of cervical spinal cord, subsequent encounter |
| S14.103S | Unspecified injury at C3 level of cervical spinal cord, sequela |
| S14.104 | Unspecified injury at C4 level of cervical spinal cord |
| S14.104A | Unspecified injury at C4 level of cervical spinal cord, initial encounter |
| S14.104D | Unspecified injury at C4 level of cervical spinal cord, subsequent encounter |
| S14.104S | Unspecified injury at C4 level of cervical spinal cord, sequela |
| S14.105 | Unspecified injury at C5 level of cervical spinal cord |
| S14.105A | Unspecified injury at C5 level of cervical spinal cord, initial encounter |
| S14.105D | Unspecified injury at C5 level of cervical spinal cord, subsequent encounter |
| S14.105S | Unspecified injury at C5 level of cervical spinal cord, sequela |
| S14.106 | Unspecified injury at C6 level of cervical spinal cord |
| S14.106A | Unspecified injury at C6 level of cervical spinal cord, initial encounter |
| S14.106D | Unspecified injury at C6 level of cervical spinal cord, subsequent encounter |
| S14.106S | Unspecified injury at C6 level of cervical spinal cord, sequela |
| S14.107 | Unspecified injury at C7 level of cervical spinal cord |
| S14.107A | Unspecified injury at C7 level of cervical spinal cord, initial encounter |
| S14.107D | Unspecified injury at C7 level of cervical spinal cord, subsequent encounter |
| S14.107S | Unspecified injury at C7 level of cervical spinal cord, sequela |
| S14.108 | Unspecified injury at C8 level of cervical spinal cord |
| S14.108A | Unspecified injury at C8 level of cervical spinal cord, initial encounter |
| S14.108D | Unspecified injury at C8 level of cervical spinal cord, subsequent encounter |
| S14.108S | Unspecified injury at C8 level of cervical spinal cord, sequela |
| S14.109 | Unspecified injury at unspecified level of cervical spinal cord |
| S14.109A | Unspecified injury at unspecified level of cervical spinal cord, initial encounter |
| S14.109D | Unspecified injury at unspecified level of cervical spinal cord, subsequent encounter |
| S14.109S | Unspecified injury at unspecified level of cervical spinal cord, sequela |
| S14.11 | Complete lesion of cervical spinal cord |
| S14.111 | Complete lesion at C1 level of cervical spinal cord |
| S14.111A | Complete lesion at C1 level of cervical spinal cord, initial encounter |
| S14.111D | Complete lesion at C1 level of cervical spinal cord, subsequent encounter |
| S14.111S | Complete lesion at C1 level of cervical spinal cord, sequela |
| S14.112 | Complete lesion at C2 level of cervical spinal cord |
| S14.112A | Complete lesion at C2 level of cervical spinal cord, initial encounter |
| S14.112D | Complete lesion at C2 level of cervical spinal cord, subsequent encounter |
| S14.112S | Complete lesion at C2 level of cervical spinal cord, sequela |
| S14.113 | Complete lesion at C3 level of cervical spinal cord |
| S14.113A | Complete lesion at C3 level of cervical spinal cord, initial encounter |
| S14.113D | Complete lesion at C3 level of cervical spinal cord, subsequent encounter |
| S14.113S | Complete lesion at C3 level of cervical spinal cord, sequela |
| S14.114 | Complete lesion at C4 level of cervical spinal cord |
| S14.114A | Complete lesion at C4 level of cervical spinal cord, initial encounter |
| S14.114D | Complete lesion at C4 level of cervical spinal cord, subsequent encounter |
| S14.114S | Complete lesion at C4 level of cervical spinal cord, sequela |
| S14.115 | Complete lesion at C5 level of cervical spinal cord |
| S14.115A | Complete lesion at C5 level of cervical spinal cord, initial encounter |
| S14.115D | Complete lesion at C5 level of cervical spinal cord, subsequent encounter |
| S14.115S | Complete lesion at C5 level of cervical spinal cord, sequela |
| S14.116 | Complete lesion at C6 level of cervical spinal cord |
| S14.116A | Complete lesion at C6 level of cervical spinal cord, initial encounter |
| S14.116D | Complete lesion at C6 level of cervical spinal cord, subsequent encounter |
| S14.116S | Complete lesion at C6 level of cervical spinal cord, sequela |
| S14.117 | Complete lesion at C7 level of cervical spinal cord |
| S14.117A | Complete lesion at C7 level of cervical spinal cord, initial encounter |
| S14.117D | Complete lesion at C7 level of cervical spinal cord, subsequent encounter |
| S14.117S | Complete lesion at C7 level of cervical spinal cord, sequela |
| S14.118 | Complete lesion at C8 level of cervical spinal cord |
| S14.118A | Complete lesion at C8 level of cervical spinal cord, initial encounter |
| S14.118D | Complete lesion at C8 level of cervical spinal cord, subsequent encounter |
| S14.118S | Complete lesion at C8 level of cervical spinal cord, sequela |
| S14.119 | Complete lesion at unspecified level of cervical spinal cord |
| S14.119A | Complete lesion at unspecified level of cervical spinal cord, initial encounter |
| S14.119D | Complete lesion at unspecified level of cervical spinal cord, subsequent encounter |
| S14.119S | Complete lesion at unspecified level of cervical spinal cord, sequela |
| S14.12 | Central cord syndrome of cervical spinal cord |
| S14.121 | Central cord syndrome at C1 level of cervical spinal cord |
| S14.121A | Central cord syndrome at C1 level of cervical spinal cord, initial encounter |
| S14.121D | Central cord syndrome at C1 level of cervical spinal cord, subsequent encounter |
| S14.121S | Central cord syndrome at C1 level of cervical spinal cord, sequela |
| S14.122 | Central cord syndrome at C2 level of cervical spinal cord |
| S14.122A | Central cord syndrome at C2 level of cervical spinal cord, initial encounter |
| S14.122D | Central cord syndrome at C2 level of cervical spinal cord, subsequent encounter |
| S14.122S | Central cord syndrome at C2 level of cervical spinal cord, sequela |
| S14.123 | Central cord syndrome at C3 level of cervical spinal cord |
| S14.123A | Central cord syndrome at C3 level of cervical spinal cord, initial encounter |
| S14.123D | Central cord syndrome at C3 level of cervical spinal cord, subsequent encounter |
| S14.123S | Central cord syndrome at C3 level of cervical spinal cord, sequela |
| S14.124 | Central cord syndrome at C4 level of cervical spinal cord |
| S14.124A | Central cord syndrome at C4 level of cervical spinal cord, initial encounter |
| S14.124D | Central cord syndrome at C4 level of cervical spinal cord, subsequent encounter |
| S14.124S | Central cord syndrome at C4 level of cervical spinal cord, sequela |
| S14.125 | Central cord syndrome at C5 level of cervical spinal cord |
| S14.125A | Central cord syndrome at C5 level of cervical spinal cord, initial encounter |
| S14.125D | Central cord syndrome at C5 level of cervical spinal cord, subsequent encounter |
| S14.125S | Central cord syndrome at C5 level of cervical spinal cord, sequela |
| S14.126 | Central cord syndrome at C6 level of cervical spinal cord |
| S14.126A | Central cord syndrome at C6 level of cervical spinal cord, initial encounter |
| S14.126D | Central cord syndrome at C6 level of cervical spinal cord, subsequent encounter |
| S14.126S | Central cord syndrome at C6 level of cervical spinal cord, sequela |
| S14.127 | Central cord syndrome at C7 level of cervical spinal cord |
| S14.127A | Central cord syndrome at C7 level of cervical spinal cord, initial encounter |
| S14.127D | Central cord syndrome at C7 level of cervical spinal cord, subsequent encounter |
| S14.127S | Central cord syndrome at C7 level of cervical spinal cord, sequela |
| S14.128 | Central cord syndrome at C8 level of cervical spinal cord |
| S14.128A | Central cord syndrome at C8 level of cervical spinal cord, initial encounter |
| S14.128D | Central cord syndrome at C8 level of cervical spinal cord, subsequent encounter |
| S14.128S | Central cord syndrome at C8 level of cervical spinal cord, sequela |
| S14.129 | Central cord syndrome at unspecified level of cervical spinal cord |
| S14.129A | Central cord syndrome at unspecified level of cervical spinal cord, initial encounter |
| S14.129D | Central cord syndrome at unspecified level of cervical spinal cord, subsequent encounter |
| S14.129S | Central cord syndrome at unspecified level of cervical spinal cord, sequela |
| S14.13 | Anterior cord syndrome of cervical spinal cord |
| S14.131 | Anterior cord syndrome at C1 level of cervical spinal cord |
| S14.131A | Anterior cord syndrome at C1 level of cervical spinal cord, initial encounter |
| S14.131D | Anterior cord syndrome at C1 level of cervical spinal cord, subsequent encounter |
| S14.131S | Anterior cord syndrome at C1 level of cervical spinal cord, sequela |
| S14.132 | Anterior cord syndrome at C2 level of cervical spinal cord |
| S14.132A | Anterior cord syndrome at C2 level of cervical spinal cord, initial encounter |
| S14.132D | Anterior cord syndrome at C2 level of cervical spinal cord, subsequent encounter |
| S14.132S | Anterior cord syndrome at C2 level of cervical spinal cord, sequela |
| S14.133 | Anterior cord syndrome at C3 level of cervical spinal cord |
| S14.133A | Anterior cord syndrome at C3 level of cervical spinal cord, initial encounter |
| S14.133D | Anterior cord syndrome at C3 level of cervical spinal cord, subsequent encounter |
| S14.133S | Anterior cord syndrome at C3 level of cervical spinal cord, sequela |
| S14.134 | Anterior cord syndrome at C4 level of cervical spinal cord |
| S14.134A | Anterior cord syndrome at C4 level of cervical spinal cord, initial encounter |
| S14.134D | Anterior cord syndrome at C4 level of cervical spinal cord, subsequent encounter |
| S14.134S | Anterior cord syndrome at C4 level of cervical spinal cord, sequela |
| S14.135 | Anterior cord syndrome at C5 level of cervical spinal cord |
| S14.135A | Anterior cord syndrome at C5 level of cervical spinal cord, initial encounter |
| S14.135D | Anterior cord syndrome at C5 level of cervical spinal cord, subsequent encounter |
| S14.135S | Anterior cord syndrome at C5 level of cervical spinal cord, sequela |
| S14.136 | Anterior cord syndrome at C6 level of cervical spinal cord |
| S14.136A | Anterior cord syndrome at C6 level of cervical spinal cord, initial encounter |
| S14.136D | Anterior cord syndrome at C6 level of cervical spinal cord, subsequent encounter |
| S14.136S | Anterior cord syndrome at C6 level of cervical spinal cord, sequela |
| S14.137 | Anterior cord syndrome at C7 level of cervical spinal cord |
| S14.137A | Anterior cord syndrome at C7 level of cervical spinal cord, initial encounter |
| S14.137D | Anterior cord syndrome at C7 level of cervical spinal cord, subsequent encounter |
| S14.137S | Anterior cord syndrome at C7 level of cervical spinal cord, sequela |
| S14.138 | Anterior cord syndrome at C8 level of cervical spinal cord |
| S14.138A | Anterior cord syndrome at C8 level of cervical spinal cord, initial encounter |
| S14.138D | Anterior cord syndrome at C8 level of cervical spinal cord, subsequent encounter |
| S14.138S | Anterior cord syndrome at C8 level of cervical spinal cord, sequela |
| S14.139 | Anterior cord syndrome at unspecified level of cervical spinal cord |
| S14.139A | Anterior cord syndrome at unspecified level of cervical spinal cord, initial encounter |
| S14.139D | Anterior cord syndrome at unspecified level of cervical spinal cord, subsequent encounter |
| S14.139S | Anterior cord syndrome at unspecified level of cervical spinal cord, sequela |
| S14.14 | Brown-Sequard syndrome of cervical spinal cord |
| S14.141 | Brown-Sequard syndrome at C1 level of cervical spinal cord |
| S14.141A | Brown-Sequard syndrome at C1 level of cervical spinal cord, initial encounter |
| S14.141D | Brown-Sequard syndrome at C1 level of cervical spinal cord, subsequent encounter |
| S14.141S | Brown-Sequard syndrome at C1 level of cervical spinal cord, sequela |
| S14.142 | Brown-Sequard syndrome at C2 level of cervical spinal cord |
| S14.142A | Brown-Sequard syndrome at C2 level of cervical spinal cord, initial encounter |
| S14.142D | Brown-Sequard syndrome at C2 level of cervical spinal cord, subsequent encounter |
| S14.142S | Brown-Sequard syndrome at C2 level of cervical spinal cord, sequela |
| S14.143 | Brown-Sequard syndrome at C3 level of cervical spinal cord |
| S14.143A | Brown-Sequard syndrome at C3 level of cervical spinal cord, initial encounter |
| S14.143D | Brown-Sequard syndrome at C3 level of cervical spinal cord, subsequent encounter |
| S14.143S | Brown-Sequard syndrome at C3 level of cervical spinal cord, sequela |
| S14.144 | Brown-Sequard syndrome at C4 level of cervical spinal cord |
| S14.144A | Brown-Sequard syndrome at C4 level of cervical spinal cord, initial encounter |
| S14.144D | Brown-Sequard syndrome at C4 level of cervical spinal cord, subsequent encounter |
| S14.144S | Brown-Sequard syndrome at C4 level of cervical spinal cord, sequela |
| S14.145 | Brown-Sequard syndrome at C5 level of cervical spinal cord |
| S14.145A | Brown-Sequard syndrome at C5 level of cervical spinal cord, initial encounter |
| S14.145D | Brown-Sequard syndrome at C5 level of cervical spinal cord, subsequent encounter |
| S14.145S | Brown-Sequard syndrome at C5 level of cervical spinal cord, sequela |
| S14.146 | Brown-Sequard syndrome at C6 level of cervical spinal cord |
| S14.146A | Brown-Sequard syndrome at C6 level of cervical spinal cord, initial encounter |
| S14.146D | Brown-Sequard syndrome at C6 level of cervical spinal cord, subsequent encounter |
| S14.146S | Brown-Sequard syndrome at C6 level of cervical spinal cord, sequela |
| S14.147 | Brown-Sequard syndrome at C7 level of cervical spinal cord |
| S14.147A | Brown-Sequard syndrome at C7 level of cervical spinal cord, initial encounter |
| S14.147D | Brown-Sequard syndrome at C7 level of cervical spinal cord, subsequent encounter |
| S14.147S | Brown-Sequard syndrome at C7 level of cervical spinal cord, sequela |
| S14.148 | Brown-Sequard syndrome at C8 level of cervical spinal cord |
| S14.148A | Brown-Sequard syndrome at C8 level of cervical spinal cord, initial encounter |
| S14.148D | Brown-Sequard syndrome at C8 level of cervical spinal cord, subsequent encounter |
| S14.148S | Brown-Sequard syndrome at C8 level of cervical spinal cord, sequela |
| S14.149 | Brown-Sequard syndrome at unspecified level of cervical spinal cord |
| S14.149A | Brown-Sequard syndrome at unspecified level of cervical spinal cord, initial encounter |
| S14.149D | Brown-Sequard syndrome at unspecified level of cervical spinal cord, subsequent encounter |
| S14.149S | Brown-Sequard syndrome at unspecified level of cervical spinal cord, sequela |
| S14.15 | Other incomplete lesions of cervical spinal cord |
| S14.151 | Other incomplete lesion at C1 level of cervical spinal cord |
| S14.151A | Other incomplete lesion at C1 level of cervical spinal cord, initial encounter |
| S14.151D | Other incomplete lesion at C1 level of cervical spinal cord, subsequent encounter |
| S14.151S | Other incomplete lesion at C1 level of cervical spinal cord, sequela |
| S14.152 | Other incomplete lesion at C2 level of cervical spinal cord |
| S14.152A | Other incomplete lesion at C2 level of cervical spinal cord, initial encounter |
| S14.152D | Other incomplete lesion at C2 level of cervical spinal cord, subsequent encounter |
| S14.152S | Other incomplete lesion at C2 level of cervical spinal cord, sequela |
| S14.153 | Other incomplete lesion at C3 level of cervical spinal cord |
| S14.153A | Other incomplete lesion at C3 level of cervical spinal cord, initial encounter |
| S14.153D | Other incomplete lesion at C3 level of cervical spinal cord, subsequent encounter |
| S14.153S | Other incomplete lesion at C3 level of cervical spinal cord, sequela |
| S14.154 | Other incomplete lesion at C4 level of cervical spinal cord |
| S14.154A | Other incomplete lesion at C4 level of cervical spinal cord, initial encounter |
| S14.154D | Other incomplete lesion at C4 level of cervical spinal cord, subsequent encounter |
| S14.154S | Other incomplete lesion at C4 level of cervical spinal cord, sequela |
| S14.155 | Other incomplete lesion at C5 level of cervical spinal cord |
| S14.155A | Other incomplete lesion at C5 level of cervical spinal cord, initial encounter |
| S14.155D | Other incomplete lesion at C5 level of cervical spinal cord, subsequent encounter |
| S14.155S | Other incomplete lesion at C5 level of cervical spinal cord, sequela |
| S14.156 | Other incomplete lesion at C6 level of cervical spinal cord |
| S14.156A | Other incomplete lesion at C6 level of cervical spinal cord, initial encounter |
| S14.156D | Other incomplete lesion at C6 level of cervical spinal cord, subsequent encounter |
| S14.156S | Other incomplete lesion at C6 level of cervical spinal cord, sequela |
| S14.157 | Other incomplete lesion at C7 level of cervical spinal cord |
| S14.157A | Other incomplete lesion at C7 level of cervical spinal cord, initial encounter |
| S14.157D | Other incomplete lesion at C7 level of cervical spinal cord, subsequent encounter |
| S14.157S | Other incomplete lesion at C7 level of cervical spinal cord, sequela |
| S14.158 | Other incomplete lesion at C8 level of cervical spinal cord |
| S14.158A | Other incomplete lesion at C8 level of cervical spinal cord, initial encounter |
| S14.158D | Other incomplete lesion at C8 level of cervical spinal cord, subsequent encounter |
| S14.158S | Other incomplete lesion at C8 level of cervical spinal cord, sequela |
| S14.159 | Other incomplete lesion at unspecified level of cervical spinal cord |
| S14.159A | Other incomplete lesion at unspecified level of cervical spinal cord, initial encounter |
| S14.159D | Other incomplete lesion at unspecified level of cervical spinal cord, subsequent encounter |
| S14.159S | Other incomplete lesion at unspecified level of cervical spinal cord, sequela |
| S14.2 | Injury of nerve root of cervical spine |
| S14.2XXA | Injury of nerve root of cervical spine, initial encounter |
| S14.2XXD | Injury of nerve root of cervical spine, subsequent encounter |
| S14.2XXS | Injury of nerve root of cervical spine, sequela |
| S14.3 | Injury of brachial plexus |
| S14.3XXA | Injury of brachial plexus, initial encounter |
| S14.3XXD | Injury of brachial plexus, subsequent encounter |
| S14.3XXS | Injury of brachial plexus, sequela |
| S14.4 | Injury of peripheral nerves of neck |
| S14.4XXA | Injury of peripheral nerves of neck, initial encounter |
| S14.4XXD | Injury of peripheral nerves of neck, subsequent encounter |
| S14.4XXS | Injury of peripheral nerves of neck, sequela |
| S14.5 | Injury of cervical sympathetic nerves |
| S14.5XXA | Injury of cervical sympathetic nerves, initial encounter |
| S14.5XXD | Injury of cervical sympathetic nerves, subsequent encounter |
| S14.5XXS | Injury of cervical sympathetic nerves, sequela |
| S14.8 | Injury of other specified nerves of neck |
| S14.8XXA | Injury of other specified nerves of neck, initial encounter |
| S14.8XXD | Injury of other specified nerves of neck, subsequent encounter |
| S14.8XXS | Injury of other specified nerves of neck, sequela |
| S14.9 | Injury of unspecified nerves of neck |
| S14.9XXA | Injury of unspecified nerves of neck, initial encounter |
| S14.9XXD | Injury of unspecified nerves of neck, subsequent encounter |
| S14.9XXS | Injury of unspecified nerves of neck, sequela |
| S21 | Open wound of thorax |
| S21.0 | Open wound of breast |
| S21.00 | Unspecified open wound of breast |
| S21.001 | Unspecified open wound of right breast |
| S21.001A | Unspecified open wound of right breast, initial encounter |
| S21.001D | Unspecified open wound of right breast, subsequent encounter |
| S21.001S | Unspecified open wound of right breast, sequela |
| S21.002 | Unspecified open wound of left breast |
| S21.002A | Unspecified open wound of left breast, initial encounter |
| S21.002D | Unspecified open wound of left breast, subsequent encounter |
| S21.002S | Unspecified open wound of left breast, sequela |
| S21.009 | Unspecified open wound of unspecified breast |
| S21.009A | Unspecified open wound of unspecified breast, initial encounter |
| S21.009D | Unspecified open wound of unspecified breast, subsequent encounter |
| S21.009S | Unspecified open wound of unspecified breast, sequela |
| S21.01 | Laceration without foreign body of breast |
| S21.011 | Laceration without foreign body of right breast |
| S21.011A | Laceration without foreign body of right breast, initial encounter |
| S21.011D | Laceration without foreign body of right breast, subsequent encounter |
| S21.011S | Laceration without foreign body of right breast, sequela |
| S21.012 | Laceration without foreign body of left breast |
| S21.012A | Laceration without foreign body of left breast, initial encounter |
| S21.012D | Laceration without foreign body of left breast, subsequent encounter |
| S21.012S | Laceration without foreign body of left breast, sequela |
| S21.019 | Laceration without foreign body of unspecified breast |
| S21.019A | Laceration without foreign body of unspecified breast, initial encounter |
| S21.019D | Laceration without foreign body of unspecified breast, subsequent encounter |
| S21.019S | Laceration without foreign body of unspecified breast, sequela |
| S21.02 | Laceration with foreign body of breast |
| S21.021 | Laceration with foreign body of right breast |
| S21.021A | Laceration with foreign body of right breast, initial encounter |
| S21.021D | Laceration with foreign body of right breast, subsequent encounter |
| S21.021S | Laceration with foreign body of right breast, sequela |
| S21.022 | Laceration with foreign body of left breast |
| S21.022A | Laceration with foreign body of left breast, initial encounter |
| S21.022D | Laceration with foreign body of left breast, subsequent encounter |
| S21.022S | Laceration with foreign body of left breast, sequela |
| S21.029 | Laceration with foreign body of unspecified breast |
| S21.029A | Laceration with foreign body of unspecified breast, initial encounter |
| S21.029D | Laceration with foreign body of unspecified breast, subsequent encounter |
| S21.029S | Laceration with foreign body of unspecified breast, sequela |
| S21.03 | Puncture wound without foreign body of breast |
| S21.031 | Puncture wound without foreign body of right breast |
| S21.031A | Puncture wound without foreign body of right breast, initial encounter |
| S21.031D | Puncture wound without foreign body of right breast, subsequent encounter |
| S21.031S | Puncture wound without foreign body of right breast, sequela |
| S21.032 | Puncture wound without foreign body of left breast |
| S21.032A | Puncture wound without foreign body of left breast, initial encounter |
| S21.032D | Puncture wound without foreign body of left breast, subsequent encounter |
| S21.032S | Puncture wound without foreign body of left breast, sequela |
| S21.039 | Puncture wound without foreign body of unspecified breast |
| S21.039A | Puncture wound without foreign body of unspecified breast, initial encounter |
| S21.039D | Puncture wound without foreign body of unspecified breast, subsequent encounter |
| S21.039S | Puncture wound without foreign body of unspecified breast, sequela |
| S21.04 | Puncture wound with foreign body of breast |
| S21.041 | Puncture wound with foreign body of right breast |
| S21.041A | Puncture wound with foreign body of right breast, initial encounter |
| S21.041D | Puncture wound with foreign body of right breast, subsequent encounter |
| S21.041S | Puncture wound with foreign body of right breast, sequela |
| S21.042 | Puncture wound with foreign body of left breast |
| S21.042A | Puncture wound with foreign body of left breast, initial encounter |
| S21.042D | Puncture wound with foreign body of left breast, subsequent encounter |
| S21.042S | Puncture wound with foreign body of left breast, sequela |
| S21.049 | Puncture wound with foreign body of unspecified breast |
| S21.049A | Puncture wound with foreign body of unspecified breast, initial encounter |
| S21.049D | Puncture wound with foreign body of unspecified breast, subsequent encounter |
| S21.049S | Puncture wound with foreign body of unspecified breast, sequela |
| S21.05 | Open bite of breast |
| S21.051 | Open bite of right breast |
| S21.051A | Open bite of right breast, initial encounter |
| S21.051D | Open bite of right breast, subsequent encounter |
| S21.051S | Open bite of right breast, sequela |
| S21.052 | Open bite of left breast |
| S21.052A | Open bite of left breast, initial encounter |
| S21.052D | Open bite of left breast, subsequent encounter |
| S21.052S | Open bite of left breast, sequela |
| S21.059 | Open bite of unspecified breast |
| S21.059A | Open bite of unspecified breast, initial encounter |
| S21.059D | Open bite of unspecified breast, subsequent encounter |
| S21.059S | Open bite of unspecified breast, sequela |
| S21.1 | Open wound of front wall of thorax without penetration into thoracic cavity |
| S21.10 | Unspecified open wound of front wall of thorax without penetration into thoracic cavity |
| S21.101 | Unspecified open wound of right front wall of thorax without penetration into thoracic cavity |
| S21.101A | Unspecified open wound of right front wall of thorax without penetration into thoracic cavity, initial encounter |
| S21.101D | Unspecified open wound of right front wall of thorax without penetration into thoracic cavity, subsequent encounter |
| S21.101S | Unspecified open wound of right front wall of thorax without penetration into thoracic cavity, sequela |
| S21.102 | Unspecified open wound of left front wall of thorax without penetration into thoracic cavity |
| S21.102A | Unspecified open wound of left front wall of thorax without penetration into thoracic cavity, initial encounter |
| S21.102D | Unspecified open wound of left front wall of thorax without penetration into thoracic cavity, subsequent encounter |
| S21.102S | Unspecified open wound of left front wall of thorax without penetration into thoracic cavity, sequela |
| S21.109 | Unspecified open wound of unspecified front wall of thorax without penetration into thoracic cavity |
| S21.109A | Unspecified open wound of unspecified front wall of thorax without penetration into thoracic cavity, initial encounter |
| S21.109D | Unspecified open wound of unspecified front wall of thorax without penetration into thoracic cavity, subsequent encounter |
| S21.109S | Unspecified open wound of unspecified front wall of thorax without penetration into thoracic cavity, sequela |
| S21.11 | Laceration without foreign body of front wall of thorax without penetration into thoracic cavity |
| S21.111 | Laceration without foreign body of right front wall of thorax without penetration into thoracic cavity |
| S21.111A | Laceration without foreign body of right front wall of thorax without penetration into thoracic cavity, initial encounter |
| S21.111D | Laceration without foreign body of right front wall of thorax without penetration into thoracic cavity, subsequent encounter |
| S21.111S | Laceration without foreign body of right front wall of thorax without penetration into thoracic cavity, sequela |
| S21.112 | Laceration without foreign body of left front wall of thorax without penetration into thoracic cavity |
| S21.112A | Laceration without foreign body of left front wall of thorax without penetration into thoracic cavity, initial encounter |
| S21.112D | Laceration without foreign body of left front wall of thorax without penetration into thoracic cavity, subsequent encounter |
| S21.112S | Laceration without foreign body of left front wall of thorax without penetration into thoracic cavity, sequela |
| S21.119 | Laceration without foreign body of unspecified front wall of thorax without penetration into thoracic cavity |
| S21.119A | Laceration without foreign body of unspecified front wall of thorax without penetration into thoracic cavity, initial encounter |
| S21.119D | Laceration without foreign body of unspecified front wall of thorax without penetration into thoracic cavity, subsequent encounter |
| S21.119S | Laceration without foreign body of unspecified front wall of thorax without penetration into thoracic cavity, sequela |
| S21.12 | Laceration with foreign body of front wall of thorax without penetration into thoracic cavity |
| S21.121 | Laceration with foreign body of right front wall of thorax without penetration into thoracic cavity |
| S21.121A | Laceration with foreign body of right front wall of thorax without penetration into thoracic cavity, initial encounter |
| S21.121D | Laceration with foreign body of right front wall of thorax without penetration into thoracic cavity, subsequent encounter |
| S21.121S | Laceration with foreign body of right front wall of thorax without penetration into thoracic cavity, sequela |
| S21.122 | Laceration with foreign body of left front wall of thorax without penetration into thoracic cavity |
| S21.122A | Laceration with foreign body of left front wall of thorax without penetration into thoracic cavity, initial encounter |
| S21.122D | Laceration with foreign body of left front wall of thorax without penetration into thoracic cavity, subsequent encounter |
| S21.122S | Laceration with foreign body of left front wall of thorax without penetration into thoracic cavity, sequela |
| S21.129 | Laceration with foreign body of unspecified front wall of thorax without penetration into thoracic cavity |
| S21.129A | Laceration with foreign body of unspecified front wall of thorax without penetration into thoracic cavity, initial encounter |
| S21.129D | Laceration with foreign body of unspecified front wall of thorax without penetration into thoracic cavity, subsequent encounter |
| S21.129S | Laceration with foreign body of unspecified front wall of thorax without penetration into thoracic cavity, sequela |
| S21.13 | Puncture wound without foreign body of front wall of thorax without penetration into thoracic cavity |
| S21.131 | Puncture wound without foreign body of right front wall of thorax without penetration into thoracic cavity |
| S21.131A | Puncture wound without foreign body of right front wall of thorax without penetration into thoracic cavity, initial encounter |
| S21.131D | Puncture wound without foreign body of right front wall of thorax without penetration into thoracic cavity, subsequent encounter |
| S21.131S | Puncture wound without foreign body of right front wall of thorax without penetration into thoracic cavity, sequela |
| S21.132 | Puncture wound without foreign body of left front wall of thorax without penetration into thoracic cavity |
| S21.132A | Puncture wound without foreign body of left front wall of thorax without penetration into thoracic cavity, initial encounter |
| S21.132D | Puncture wound without foreign body of left front wall of thorax without penetration into thoracic cavity, subsequent encounter |
| S21.132S | Puncture wound without foreign body of left front wall of thorax without penetration into thoracic cavity, sequela |
| S21.139 | Puncture wound without foreign body of unspecified front wall of thorax without penetration into thoracic cavity |
| S21.139A | Puncture wound without foreign body of unspecified front wall of thorax without penetration into thoracic cavity, initial encounter |
| S21.139D | Puncture wound without foreign body of unspecified front wall of thorax without penetration into thoracic cavity, subsequent encounter |
| S21.139S | Puncture wound without foreign body of unspecified front wall of thorax without penetration into thoracic cavity, sequela |
| S21.14 | Puncture wound with foreign body of front wall of thorax without penetration into thoracic cavity |
| S21.141 | Puncture wound with foreign body of right front wall of thorax without penetration into thoracic cavity |
| S21.141A | Puncture wound with foreign body of right front wall of thorax without penetration into thoracic cavity, initial encounter |
| S21.141D | Puncture wound with foreign body of right front wall of thorax without penetration into thoracic cavity, subsequent encounter |
| S21.141S | Puncture wound with foreign body of right front wall of thorax without penetration into thoracic cavity, sequela |
| S21.142 | Puncture wound with foreign body of left front wall of thorax without penetration into thoracic cavity |
| S21.142A | Puncture wound with foreign body of left front wall of thorax without penetration into thoracic cavity, initial encounter |
| S21.142D | Puncture wound with foreign body of left front wall of thorax without penetration into thoracic cavity, subsequent encounter |
| S21.142S | Puncture wound with foreign body of left front wall of thorax without penetration into thoracic cavity, sequela |
| S21.149 | Puncture wound with foreign body of unspecified front wall of thorax without penetration into thoracic cavity |
| S21.149A | Puncture wound with foreign body of unspecified front wall of thorax without penetration into thoracic cavity, initial encounter |
| S21.149D | Puncture wound with foreign body of unspecified front wall of thorax without penetration into thoracic cavity, subsequent encounter |
| S21.149S | Puncture wound with foreign body of unspecified front wall of thorax without penetration into thoracic cavity, sequela |
| S21.15 | Open bite of front wall of thorax without penetration into thoracic cavity |
| S21.151 | Open bite of right front wall of thorax without penetration into thoracic cavity |
| S21.151A | Open bite of right front wall of thorax without penetration into thoracic cavity, initial encounter |
| S21.151D | Open bite of right front wall of thorax without penetration into thoracic cavity, subsequent encounter |
| S21.151S | Open bite of right front wall of thorax without penetration into thoracic cavity, sequela |
| S21.152 | Open bite of left front wall of thorax without penetration into thoracic cavity |
| S21.152A | Open bite of left front wall of thorax without penetration into thoracic cavity, initial encounter |
| S21.152D | Open bite of left front wall of thorax without penetration into thoracic cavity, subsequent encounter |
| S21.152S | Open bite of left front wall of thorax without penetration into thoracic cavity, sequela |
| S21.159 | Open bite of unspecified front wall of thorax without penetration into thoracic cavity |
| S21.159A | Open bite of unspecified front wall of thorax without penetration into thoracic cavity, initial encounter |
| S21.159D | Open bite of unspecified front wall of thorax without penetration into thoracic cavity, subsequent encounter |
| S21.159S | Open bite of unspecified front wall of thorax without penetration into thoracic cavity, sequela |
| S21.2 | Open wound of back wall of thorax without penetration into thoracic cavity |
| S21.20 | Unspecified open wound of back wall of thorax without penetration into thoracic cavity |
| S21.201 | Unspecified open wound of right back wall of thorax without penetration into thoracic cavity |
| S21.201A | Unspecified open wound of right back wall of thorax without penetration into thoracic cavity, initial encounter |
| S21.201D | Unspecified open wound of right back wall of thorax without penetration into thoracic cavity, subsequent encounter |
| S21.201S | Unspecified open wound of right back wall of thorax without penetration into thoracic cavity, sequela |
| S21.202 | Unspecified open wound of left back wall of thorax without penetration into thoracic cavity |
| S21.202A | Unspecified open wound of left back wall of thorax without penetration into thoracic cavity, initial encounter |
| S21.202D | Unspecified open wound of left back wall of thorax without penetration into thoracic cavity, subsequent encounter |
| S21.202S | Unspecified open wound of left back wall of thorax without penetration into thoracic cavity, sequela |
| S21.209 | Unspecified open wound of unspecified back wall of thorax without penetration into thoracic cavity |
| S21.209A | Unspecified open wound of unspecified back wall of thorax without penetration into thoracic cavity, initial encounter |
| S21.209D | Unspecified open wound of unspecified back wall of thorax without penetration into thoracic cavity, subsequent encounter |
| S21.209S | Unspecified open wound of unspecified back wall of thorax without penetration into thoracic cavity, sequela |
| S21.21 | Laceration without foreign body of back wall of thorax without penetration into thoracic cavity |
| S21.211 | Laceration without foreign body of right back wall of thorax without penetration into thoracic cavity |
| S21.211A | Laceration without foreign body of right back wall of thorax without penetration into thoracic cavity, initial encounter |
| S21.211D | Laceration without foreign body of right back wall of thorax without penetration into thoracic cavity, subsequent encounter |
| S21.211S | Laceration without foreign body of right back wall of thorax without penetration into thoracic cavity, sequela |
| S21.212 | Laceration without foreign body of left back wall of thorax without penetration into thoracic cavity |
| S21.212A | Laceration without foreign body of left back wall of thorax without penetration into thoracic cavity, initial encounter |
| S21.212D | Laceration without foreign body of left back wall of thorax without penetration into thoracic cavity, subsequent encounter |
| S21.212S | Laceration without foreign body of left back wall of thorax without penetration into thoracic cavity, sequela |
| S21.219 | Laceration without foreign body of unspecified back wall of thorax without penetration into thoracic cavity |
| S21.219A | Laceration without foreign body of unspecified back wall of thorax without penetration into thoracic cavity, initial encounter |
| S21.219D | Laceration without foreign body of unspecified back wall of thorax without penetration into thoracic cavity, subsequent encounter |
| S21.219S | Laceration without foreign body of unspecified back wall of thorax without penetration into thoracic cavity, sequela |
| S21.22 | Laceration with foreign body of back wall of thorax without penetration into thoracic cavity |
| S21.221 | Laceration with foreign body of right back wall of thorax without penetration into thoracic cavity |
| S21.221A | Laceration with foreign body of right back wall of thorax without penetration into thoracic cavity, initial encounter |
| S21.221D | Laceration with foreign body of right back wall of thorax without penetration into thoracic cavity, subsequent encounter |
| S21.221S | Laceration with foreign body of right back wall of thorax without penetration into thoracic cavity, sequela |
| S21.222 | Laceration with foreign body of left back wall of thorax without penetration into thoracic cavity |
| S21.222A | Laceration with foreign body of left back wall of thorax without penetration into thoracic cavity, initial encounter |
| S21.222D | Laceration with foreign body of left back wall of thorax without penetration into thoracic cavity, subsequent encounter |
| S21.222S | Laceration with foreign body of left back wall of thorax without penetration into thoracic cavity, sequela |
| S21.229 | Laceration with foreign body of unspecified back wall of thorax without penetration into thoracic cavity |
| S21.229A | Laceration with foreign body of unspecified back wall of thorax without penetration into thoracic cavity, initial encounter |
| S21.229D | Laceration with foreign body of unspecified back wall of thorax without penetration into thoracic cavity, subsequent encounter |
| S21.229S | Laceration with foreign body of unspecified back wall of thorax without penetration into thoracic cavity, sequela |
| S21.23 | Puncture wound without foreign body of back wall of thorax without penetration into thoracic cavity |
| S21.231 | Puncture wound without foreign body of right back wall of thorax without penetration into thoracic cavity |
| S21.231A | Puncture wound without foreign body of right back wall of thorax without penetration into thoracic cavity, initial encounter |
| S21.231D | Puncture wound without foreign body of right back wall of thorax without penetration into thoracic cavity, subsequent encounter |
| S21.231S | Puncture wound without foreign body of right back wall of thorax without penetration into thoracic cavity, sequela |
| S21.232 | Puncture wound without foreign body of left back wall of thorax without penetration into thoracic cavity |
| S21.232A | Puncture wound without foreign body of left back wall of thorax without penetration into thoracic cavity, initial encounter |
| S21.232D | Puncture wound without foreign body of left back wall of thorax without penetration into thoracic cavity, subsequent encounter |
| S21.232S | Puncture wound without foreign body of left back wall of thorax without penetration into thoracic cavity, sequela |
| S21.239 | Puncture wound without foreign body of unspecified back wall of thorax without penetration into thoracic cavity |
| S21.239A | Puncture wound without foreign body of unspecified back wall of thorax without penetration into thoracic cavity, initial encounter |
| S21.239D | Puncture wound without foreign body of unspecified back wall of thorax without penetration into thoracic cavity, subsequent encounter |
| S21.239S | Puncture wound without foreign body of unspecified back wall of thorax without penetration into thoracic cavity, sequela |
| S21.24 | Puncture wound with foreign body of back wall of thorax without penetration into thoracic cavity |
| S21.241 | Puncture wound with foreign body of right back wall of thorax without penetration into thoracic cavity |
| S21.241A | Puncture wound with foreign body of right back wall of thorax without penetration into thoracic cavity, initial encounter |
| S21.241D | Puncture wound with foreign body of right back wall of thorax without penetration into thoracic cavity, subsequent encounter |
| S21.241S | Puncture wound with foreign body of right back wall of thorax without penetration into thoracic cavity, sequela |
| S21.242 | Puncture wound with foreign body of left back wall of thorax without penetration into thoracic cavity |
| S21.242A | Puncture wound with foreign body of left back wall of thorax without penetration into thoracic cavity, initial encounter |
| S21.242D | Puncture wound with foreign body of left back wall of thorax without penetration into thoracic cavity, subsequent encounter |
| S21.242S | Puncture wound with foreign body of left back wall of thorax without penetration into thoracic cavity, sequela |
| S21.249 | Puncture wound with foreign body of unspecified back wall of thorax without penetration into thoracic cavity |
| S21.249A | Puncture wound with foreign body of unspecified back wall of thorax without penetration into thoracic cavity, initial encounter |
| S21.249D | Puncture wound with foreign body of unspecified back wall of thorax without penetration into thoracic cavity, subsequent encounter |
| S21.249S | Puncture wound with foreign body of unspecified back wall of thorax without penetration into thoracic cavity, sequela |
| S21.25 | Open bite of back wall of thorax without penetration into thoracic cavity |
| S21.251 | Open bite of right back wall of thorax without penetration into thoracic cavity |
| S21.251A | Open bite of right back wall of thorax without penetration into thoracic cavity, initial encounter |
| S21.251D | Open bite of right back wall of thorax without penetration into thoracic cavity, subsequent encounter |
| S21.251S | Open bite of right back wall of thorax without penetration into thoracic cavity, sequela |
| S21.252 | Open bite of left back wall of thorax without penetration into thoracic cavity |
| S21.252A | Open bite of left back wall of thorax without penetration into thoracic cavity, initial encounter |
| S21.252D | Open bite of left back wall of thorax without penetration into thoracic cavity, subsequent encounter |
| S21.252S | Open bite of left back wall of thorax without penetration into thoracic cavity, sequela |
| S21.259 | Open bite of unspecified back wall of thorax without penetration into thoracic cavity |
| S21.259A | Open bite of unspecified back wall of thorax without penetration into thoracic cavity, initial encounter |
| S21.259D | Open bite of unspecified back wall of thorax without penetration into thoracic cavity, subsequent encounter |
| S21.259S | Open bite of unspecified back wall of thorax without penetration into thoracic cavity, sequela |
| S21.3 | Open wound of front wall of thorax with penetration into thoracic cavity |
| S21.30 | Unspecified open wound of front wall of thorax with penetration into thoracic cavity |
| S21.301 | Unspecified open wound of right front wall of thorax with penetration into thoracic cavity |
| S21.301A | Unspecified open wound of right front wall of thorax with penetration into thoracic cavity, initial encounter |
| S21.301D | Unspecified open wound of right front wall of thorax with penetration into thoracic cavity, subsequent encounter |
| S21.301S | Unspecified open wound of right front wall of thorax with penetration into thoracic cavity, sequela |
| S21.302 | Unspecified open wound of left front wall of thorax with penetration into thoracic cavity |
| S21.302A | Unspecified open wound of left front wall of thorax with penetration into thoracic cavity, initial encounter |
| S21.302D | Unspecified open wound of left front wall of thorax with penetration into thoracic cavity, subsequent encounter |
| S21.302S | Unspecified open wound of left front wall of thorax with penetration into thoracic cavity, sequela |
| S21.309 | Unspecified open wound of unspecified front wall of thorax with penetration into thoracic cavity |
| S21.309A | Unspecified open wound of unspecified front wall of thorax with penetration into thoracic cavity, initial encounter |
| S21.309D | Unspecified open wound of unspecified front wall of thorax with penetration into thoracic cavity, subsequent encounter |
| S21.309S | Unspecified open wound of unspecified front wall of thorax with penetration into thoracic cavity, sequela |
| S21.31 | Laceration without foreign body of front wall of thorax with penetration into thoracic cavity |
| S21.311 | Laceration without foreign body of right front wall of thorax with penetration into thoracic cavity |
| S21.311A | Laceration without foreign body of right front wall of thorax with penetration into thoracic cavity, initial encounter |
| S21.311D | Laceration without foreign body of right front wall of thorax with penetration into thoracic cavity, subsequent encounter |
| S21.311S | Laceration without foreign body of right front wall of thorax with penetration into thoracic cavity, sequela |
| S21.312 | Laceration without foreign body of left front wall of thorax with penetration into thoracic cavity |
| S21.312A | Laceration without foreign body of left front wall of thorax with penetration into thoracic cavity, initial encounter |
| S21.312D | Laceration without foreign body of left front wall of thorax with penetration into thoracic cavity, subsequent encounter |
| S21.312S | Laceration without foreign body of left front wall of thorax with penetration into thoracic cavity, sequela |
| S21.319 | Laceration without foreign body of unspecified front wall of thorax with penetration into thoracic cavity |
| S21.319A | Laceration without foreign body of unspecified front wall of thorax with penetration into thoracic cavity, initial encounter |
| S21.319D | Laceration without foreign body of unspecified front wall of thorax with penetration into thoracic cavity, subsequent encounter |
| S21.319S | Laceration without foreign body of unspecified front wall of thorax with penetration into thoracic cavity, sequela |
| S21.32 | Laceration with foreign body of front wall of thorax with penetration into thoracic cavity |
| S21.321 | Laceration with foreign body of right front wall of thorax with penetration into thoracic cavity |
| S21.321A | Laceration with foreign body of right front wall of thorax with penetration into thoracic cavity, initial encounter |
| S21.321D | Laceration with foreign body of right front wall of thorax with penetration into thoracic cavity, subsequent encounter |
| S21.321S | Laceration with foreign body of right front wall of thorax with penetration into thoracic cavity, sequela |
| S21.322 | Laceration with foreign body of left front wall of thorax with penetration into thoracic cavity |
| S21.322A | Laceration with foreign body of left front wall of thorax with penetration into thoracic cavity, initial encounter |
| S21.322D | Laceration with foreign body of left front wall of thorax with penetration into thoracic cavity, subsequent encounter |
| S21.322S | Laceration with foreign body of left front wall of thorax with penetration into thoracic cavity, sequela |
| S21.329 | Laceration with foreign body of unspecified front wall of thorax with penetration into thoracic cavity |
| S21.329A | Laceration with foreign body of unspecified front wall of thorax with penetration into thoracic cavity, initial encounter |
| S21.329D | Laceration with foreign body of unspecified front wall of thorax with penetration into thoracic cavity, subsequent encounter |
| S21.329S | Laceration with foreign body of unspecified front wall of thorax with penetration into thoracic cavity, sequela |
| S21.33 | Puncture wound without foreign body of front wall of thorax with penetration into thoracic cavity |
| S21.331 | Puncture wound without foreign body of right front wall of thorax with penetration into thoracic cavity |
| S21.331A | Puncture wound without foreign body of right front wall of thorax with penetration into thoracic cavity, initial encounter |
| S21.331D | Puncture wound without foreign body of right front wall of thorax with penetration into thoracic cavity, subsequent encounter |
| S21.331S | Puncture wound without foreign body of right front wall of thorax with penetration into thoracic cavity, sequela |
| S21.332 | Puncture wound without foreign body of left front wall of thorax with penetration into thoracic cavity |
| S21.332A | Puncture wound without foreign body of left front wall of thorax with penetration into thoracic cavity, initial encounter |
| S21.332D | Puncture wound without foreign body of left front wall of thorax with penetration into thoracic cavity, subsequent encounter |
| S21.332S | Puncture wound without foreign body of left front wall of thorax with penetration into thoracic cavity, sequela |
| S21.339 | Puncture wound without foreign body of unspecified front wall of thorax with penetration into thoracic cavity |
| S21.339A | Puncture wound without foreign body of unspecified front wall of thorax with penetration into thoracic cavity, initial encounter |
| S21.339D | Puncture wound without foreign body of unspecified front wall of thorax with penetration into thoracic cavity, subsequent encounter |
| S21.339S | Puncture wound without foreign body of unspecified front wall of thorax with penetration into thoracic cavity, sequela |
| S21.34 | Puncture wound with foreign body of front wall of thorax with penetration into thoracic cavity |
| S21.341 | Puncture wound with foreign body of right front wall of thorax with penetration into thoracic cavity |
| S21.341A | Puncture wound with foreign body of right front wall of thorax with penetration into thoracic cavity, initial encounter |
| S21.341D | Puncture wound with foreign body of right front wall of thorax with penetration into thoracic cavity, subsequent encounter |
| S21.341S | Puncture wound with foreign body of right front wall of thorax with penetration into thoracic cavity, sequela |
| S21.342 | Puncture wound with foreign body of left front wall of thorax with penetration into thoracic cavity |
| S21.342A | Puncture wound with foreign body of left front wall of thorax with penetration into thoracic cavity, initial encounter |
| S21.342D | Puncture wound with foreign body of left front wall of thorax with penetration into thoracic cavity, subsequent encounter |
| S21.342S | Puncture wound with foreign body of left front wall of thorax with penetration into thoracic cavity, sequela |
| S21.349 | Puncture wound with foreign body of unspecified front wall of thorax with penetration into thoracic cavity |
| S21.349A | Puncture wound with foreign body of unspecified front wall of thorax with penetration into thoracic cavity, initial encounter |
| S21.349D | Puncture wound with foreign body of unspecified front wall of thorax with penetration into thoracic cavity, subsequent encounter |
| S21.349S | Puncture wound with foreign body of unspecified front wall of thorax with penetration into thoracic cavity, sequela |
| S21.35 | Open bite of front wall of thorax with penetration into thoracic cavity |
| S21.351 | Open bite of right front wall of thorax with penetration into thoracic cavity |
| S21.351A | Open bite of right front wall of thorax with penetration into thoracic cavity, initial encounter |
| S21.351D | Open bite of right front wall of thorax with penetration into thoracic cavity, subsequent encounter |
| S21.351S | Open bite of right front wall of thorax with penetration into thoracic cavity, sequela |
| S21.352 | Open bite of left front wall of thorax with penetration into thoracic cavity |
| S21.352A | Open bite of left front wall of thorax with penetration into thoracic cavity, initial encounter |
| S21.352D | Open bite of left front wall of thorax with penetration into thoracic cavity, subsequent encounter |
| S21.352S | Open bite of left front wall of thorax with penetration into thoracic cavity, sequela |
| S21.359 | Open bite of unspecified front wall of thorax with penetration into thoracic cavity |
| S21.359A | Open bite of unspecified front wall of thorax with penetration into thoracic cavity, initial encounter |
| S21.359D | Open bite of unspecified front wall of thorax with penetration into thoracic cavity, subsequent encounter |
| S21.359S | Open bite of unspecified front wall of thorax with penetration into thoracic cavity, sequela |
| S21.4 | Open wound of back wall of thorax with penetration into thoracic cavity |
| S21.40 | Unspecified open wound of back wall of thorax with penetration into thoracic cavity |
| S21.401 | Unspecified open wound of right back wall of thorax with penetration into thoracic cavity |
| S21.401A | Unspecified open wound of right back wall of thorax with penetration into thoracic cavity, initial encounter |
| S21.401D | Unspecified open wound of right back wall of thorax with penetration into thoracic cavity, subsequent encounter |
| S21.401S | Unspecified open wound of right back wall of thorax with penetration into thoracic cavity, sequela |
| S21.402 | Unspecified open wound of left back wall of thorax with penetration into thoracic cavity |
| S21.402A | Unspecified open wound of left back wall of thorax with penetration into thoracic cavity, initial encounter |
| S21.402D | Unspecified open wound of left back wall of thorax with penetration into thoracic cavity, subsequent encounter |
| S21.402S | Unspecified open wound of left back wall of thorax with penetration into thoracic cavity, sequela |
| S21.409 | Unspecified open wound of unspecified back wall of thorax with penetration into thoracic cavity |
| S21.409A | Unspecified open wound of unspecified back wall of thorax with penetration into thoracic cavity, initial encounter |
| S21.409D | Unspecified open wound of unspecified back wall of thorax with penetration into thoracic cavity, subsequent encounter |
| S21.409S | Unspecified open wound of unspecified back wall of thorax with penetration into thoracic cavity, sequela |
| S21.41 | Laceration without foreign body of back wall of thorax with penetration into thoracic cavity |
| S21.411 | Laceration without foreign body of right back wall of thorax with penetration into thoracic cavity |
| S21.411A | Laceration without foreign body of right back wall of thorax with penetration into thoracic cavity, initial encounter |
| S21.411D | Laceration without foreign body of right back wall of thorax with penetration into thoracic cavity, subsequent encounter |
| S21.411S | Laceration without foreign body of right back wall of thorax with penetration into thoracic cavity, sequela |
| S21.412 | Laceration without foreign body of left back wall of thorax with penetration into thoracic cavity |
| S21.412A | Laceration without foreign body of left back wall of thorax with penetration into thoracic cavity, initial encounter |
| S21.412D | Laceration without foreign body of left back wall of thorax with penetration into thoracic cavity, subsequent encounter |
| S21.412S | Laceration without foreign body of left back wall of thorax with penetration into thoracic cavity, sequela |
| S21.419 | Laceration without foreign body of unspecified back wall of thorax with penetration into thoracic cavity |
| S21.419A | Laceration without foreign body of unspecified back wall of thorax with penetration into thoracic cavity, initial encounter |
| S21.419D | Laceration without foreign body of unspecified back wall of thorax with penetration into thoracic cavity, subsequent encounter |
| S21.419S | Laceration without foreign body of unspecified back wall of thorax with penetration into thoracic cavity, sequela |
| S21.42 | Laceration with foreign body of back wall of thorax with penetration into thoracic cavity |
| S21.421 | Laceration with foreign body of right back wall of thorax with penetration into thoracic cavity |
| S21.421A | Laceration with foreign body of right back wall of thorax with penetration into thoracic cavity, initial encounter |
| S21.421D | Laceration with foreign body of right back wall of thorax with penetration into thoracic cavity, subsequent encounter |
| S21.421S | Laceration with foreign body of right back wall of thorax with penetration into thoracic cavity, sequela |
| S21.422 | Laceration with foreign body of left back wall of thorax with penetration into thoracic cavity |
| S21.422A | Laceration with foreign body of left back wall of thorax with penetration into thoracic cavity, initial encounter |
| S21.422D | Laceration with foreign body of left back wall of thorax with penetration into thoracic cavity, subsequent encounter |
| S21.422S | Laceration with foreign body of left back wall of thorax with penetration into thoracic cavity, sequela |
| S21.429 | Laceration with foreign body of unspecified back wall of thorax with penetration into thoracic cavity |
| S21.429A | Laceration with foreign body of unspecified back wall of thorax with penetration into thoracic cavity, initial encounter |
| S21.429D | Laceration with foreign body of unspecified back wall of thorax with penetration into thoracic cavity, subsequent encounter |
| S21.429S | Laceration with foreign body of unspecified back wall of thorax with penetration into thoracic cavity, sequela |
| S21.43 | Puncture wound without foreign body of back wall of thorax with penetration into thoracic cavity |
| S21.431 | Puncture wound without foreign body of right back wall of thorax with penetration into thoracic cavity |
| S21.431A | Puncture wound without foreign body of right back wall of thorax with penetration into thoracic cavity, initial encounter |
| S21.431D | Puncture wound without foreign body of right back wall of thorax with penetration into thoracic cavity, subsequent encounter |
| S21.431S | Puncture wound without foreign body of right back wall of thorax with penetration into thoracic cavity, sequela |
| S21.432 | Puncture wound without foreign body of left back wall of thorax with penetration into thoracic cavity |
| S21.432A | Puncture wound without foreign body of left back wall of thorax with penetration into thoracic cavity, initial encounter |
| S21.432D | Puncture wound without foreign body of left back wall of thorax with penetration into thoracic cavity, subsequent encounter |
| S21.432S | Puncture wound without foreign body of left back wall of thorax with penetration into thoracic cavity, sequela |
| S21.439 | Puncture wound without foreign body of unspecified back wall of thorax with penetration into thoracic cavity |
| S21.439A | Puncture wound without foreign body of unspecified back wall of thorax with penetration into thoracic cavity, initial encounter |
| S21.439D | Puncture wound without foreign body of unspecified back wall of thorax with penetration into thoracic cavity, subsequent encounter |
| S21.439S | Puncture wound without foreign body of unspecified back wall of thorax with penetration into thoracic cavity, sequela |
| S21.44 | Puncture wound with foreign body of back wall of thorax with penetration into thoracic cavity |
| S21.441 | Puncture wound with foreign body of right back wall of thorax with penetration into thoracic cavity |
| S21.441A | Puncture wound with foreign body of right back wall of thorax with penetration into thoracic cavity, initial encounter |
| S21.441D | Puncture wound with foreign body of right back wall of thorax with penetration into thoracic cavity, subsequent encounter |
| S21.441S | Puncture wound with foreign body of right back wall of thorax with penetration into thoracic cavity, sequela |
| S21.442 | Puncture wound with foreign body of left back wall of thorax with penetration into thoracic cavity |
| S21.442A | Puncture wound with foreign body of left back wall of thorax with penetration into thoracic cavity, initial encounter |
| S21.442D | Puncture wound with foreign body of left back wall of thorax with penetration into thoracic cavity, subsequent encounter |
| S21.442S | Puncture wound with foreign body of left back wall of thorax with penetration into thoracic cavity, sequela |
| S21.449 | Puncture wound with foreign body of unspecified back wall of thorax with penetration into thoracic cavity |
| S21.449A | Puncture wound with foreign body of unspecified back wall of thorax with penetration into thoracic cavity, initial encounter |
| S21.449D | Puncture wound with foreign body of unspecified back wall of thorax with penetration into thoracic cavity, subsequent encounter |
| S21.449S | Puncture wound with foreign body of unspecified back wall of thorax with penetration into thoracic cavity, sequela |
| S21.45 | Open bite of back wall of thorax with penetration into thoracic cavity |
| S21.451 | Open bite of right back wall of thorax with penetration into thoracic cavity |
| S21.451A | Open bite of right back wall of thorax with penetration into thoracic cavity, initial encounter |
| S21.451D | Open bite of right back wall of thorax with penetration into thoracic cavity, subsequent encounter |
| S21.451S | Open bite of right back wall of thorax with penetration into thoracic cavity, sequela |
| S21.452 | Open bite of left back wall of thorax with penetration into thoracic cavity |
| S21.452A | Open bite of left back wall of thorax with penetration into thoracic cavity, initial encounter |
| S21.452D | Open bite of left back wall of thorax with penetration into thoracic cavity, subsequent encounter |
| S21.452S | Open bite of left back wall of thorax with penetration into thoracic cavity, sequela |
| S21.459 | Open bite of unspecified back wall of thorax with penetration into thoracic cavity |
| S21.459A | Open bite of unspecified back wall of thorax with penetration into thoracic cavity, initial encounter |
| S21.459D | Open bite of unspecified back wall of thorax with penetration into thoracic cavity, subsequent encounter |
| S21.459S | Open bite of unspecified back wall of thorax with penetration into thoracic cavity, sequela |
| S21.9 | Open wound of unspecified part of thorax |
| S21.90 | Unspecified open wound of unspecified part of thorax |
| S21.90XA | Unspecified open wound of unspecified part of thorax, initial encounter |
| S21.90XD | Unspecified open wound of unspecified part of thorax, subsequent encounter |
| S21.90XS | Unspecified open wound of unspecified part of thorax, sequela |
| S21.91 | Laceration without foreign body of unspecified part of thorax |
| S21.91XA | Laceration without foreign body of unspecified part of thorax, initial encounter |
| S21.91XD | Laceration without foreign body of unspecified part of thorax, subsequent encounter |
| S21.91XS | Laceration without foreign body of unspecified part of thorax, sequela |
| S21.92 | Laceration with foreign body of unspecified part of thorax |
| S21.92XA | Laceration with foreign body of unspecified part of thorax, initial encounter |
| S21.92XD | Laceration with foreign body of unspecified part of thorax, subsequent encounter |
| S21.92XS | Laceration with foreign body of unspecified part of thorax, sequela |
| S21.93 | Puncture wound without foreign body of unspecified part of thorax |
| S21.93XA | Puncture wound without foreign body of unspecified part of thorax, initial encounter |
| S21.93XD | Puncture wound without foreign body of unspecified part of thorax, subsequent encounter |
| S21.93XS | Puncture wound without foreign body of unspecified part of thorax, sequela |
| S21.94 | Puncture wound with foreign body of unspecified part of thorax |
| S21.94XA | Puncture wound with foreign body of unspecified part of thorax, initial encounter |
| S21.94XD | Puncture wound with foreign body of unspecified part of thorax, subsequent encounter |
| S21.94XS | Puncture wound with foreign body of unspecified part of thorax, sequela |
| S21.95 | Open bite of unspecified part of thorax |
| S21.95XA | Open bite of unspecified part of thorax, initial encounter |
| S21.95XD | Open bite of unspecified part of thorax, subsequent encounter |
| S21.95XS | Open bite of unspecified part of thorax, sequela |
| S22 | Fracture of rib(s), sternum and thoracic spine |
| S22.0 | Fracture of thoracic vertebra |
| S22.00 | Fracture of unspecified thoracic vertebra |
| S22.000 | Wedge compression fracture of unspecified thoracic vertebra |
| S22.000A | Wedge compression fracture of unspecified thoracic vertebra, initial encounter for closed fracture |
| S22.000B | Wedge compression fracture of unspecified thoracic vertebra, initial encounter for open fracture |
| S22.000D | Wedge compression fracture of unspecified thoracic vertebra, subsequent encounter for fracture with routine healing |
| S22.000G | Wedge compression fracture of unspecified thoracic vertebra, subsequent encounter for fracture with delayed healing |
| S22.000K | Wedge compression fracture of unspecified thoracic vertebra, subsequent encounter for fracture with nonunion |
| S22.000S | Wedge compression fracture of unspecified thoracic vertebra, sequela |
| S22.001 | Stable burst fracture of unspecified thoracic vertebra |
| S22.001A | Stable burst fracture of unspecified thoracic vertebra, initial encounter for closed fracture |
| S22.001B | Stable burst fracture of unspecified thoracic vertebra, initial encounter for open fracture |
| S22.001D | Stable burst fracture of unspecified thoracic vertebra, subsequent encounter for fracture with routine healing |
| S22.001G | Stable burst fracture of unspecified thoracic vertebra, subsequent encounter for fracture with delayed healing |
| S22.001K | Stable burst fracture of unspecified thoracic vertebra, subsequent encounter for fracture with nonunion |
| S22.001S | Stable burst fracture of unspecified thoracic vertebra, sequela |
| S22.002 | Unstable burst fracture of unspecified thoracic vertebra |
| S22.002A | Unstable burst fracture of unspecified thoracic vertebra, initial encounter for closed fracture |
| S22.002B | Unstable burst fracture of unspecified thoracic vertebra, initial encounter for open fracture |
| S22.002D | Unstable burst fracture of unspecified thoracic vertebra, subsequent encounter for fracture with routine healing |
| S22.002G | Unstable burst fracture of unspecified thoracic vertebra, subsequent encounter for fracture with delayed healing |
| S22.002K | Unstable burst fracture of unspecified thoracic vertebra, subsequent encounter for fracture with nonunion |
| S22.002S | Unstable burst fracture of unspecified thoracic vertebra, sequela |
| S22.008 | Other fracture of unspecified thoracic vertebra |
| S22.008A | Other fracture of unspecified thoracic vertebra, initial encounter for closed fracture |
| S22.008B | Other fracture of unspecified thoracic vertebra, initial encounter for open fracture |
| S22.008D | Other fracture of unspecified thoracic vertebra, subsequent encounter for fracture with routine healing |
| S22.008G | Other fracture of unspecified thoracic vertebra, subsequent encounter for fracture with delayed healing |
| S22.008K | Other fracture of unspecified thoracic vertebra, subsequent encounter for fracture with nonunion |
| S22.008S | Other fracture of unspecified thoracic vertebra, sequela |
| S22.009 | Unspecified fracture of unspecified thoracic vertebra |
| S22.009A | Unspecified fracture of unspecified thoracic vertebra, initial encounter for closed fracture |
| S22.009B | Unspecified fracture of unspecified thoracic vertebra, initial encounter for open fracture |
| S22.009D | Unspecified fracture of unspecified thoracic vertebra, subsequent encounter for fracture with routine healing |
| S22.009G | Unspecified fracture of unspecified thoracic vertebra, subsequent encounter for fracture with delayed healing |
| S22.009K | Unspecified fracture of unspecified thoracic vertebra, subsequent encounter for fracture with nonunion |
| S22.009S | Unspecified fracture of unspecified thoracic vertebra, sequela |
| S22.01 | Fracture of first thoracic vertebra |
| S22.010 | Wedge compression fracture of first thoracic vertebra |
| S22.010A | Wedge compression fracture of first thoracic vertebra, initial encounter for closed fracture |
| S22.010B | Wedge compression fracture of first thoracic vertebra, initial encounter for open fracture |
| S22.010D | Wedge compression fracture of first thoracic vertebra, subsequent encounter for fracture with routine healing |
| S22.010G | Wedge compression fracture of first thoracic vertebra, subsequent encounter for fracture with delayed healing |
| S22.010K | Wedge compression fracture of first thoracic vertebra, subsequent encounter for fracture with nonunion |
| S22.010S | Wedge compression fracture of first thoracic vertebra, sequela |
| S22.011 | Stable burst fracture of first thoracic vertebra |
| S22.011A | Stable burst fracture of first thoracic vertebra, initial encounter for closed fracture |
| S22.011B | Stable burst fracture of first thoracic vertebra, initial encounter for open fracture |
| S22.011D | Stable burst fracture of first thoracic vertebra, subsequent encounter for fracture with routine healing |
| S22.011G | Stable burst fracture of first thoracic vertebra, subsequent encounter for fracture with delayed healing |
| S22.011K | Stable burst fracture of first thoracic vertebra, subsequent encounter for fracture with nonunion |
| S22.011S | Stable burst fracture of first thoracic vertebra, sequela |
| S22.012 | Unstable burst fracture of first thoracic vertebra |
| S22.012A | Unstable burst fracture of first thoracic vertebra, initial encounter for closed fracture |
| S22.012B | Unstable burst fracture of first thoracic vertebra, initial encounter for open fracture |
| S22.012D | Unstable burst fracture of first thoracic vertebra, subsequent encounter for fracture with routine healing |
| S22.012G | Unstable burst fracture of first thoracic vertebra, subsequent encounter for fracture with delayed healing |
| S22.012K | Unstable burst fracture of first thoracic vertebra, subsequent encounter for fracture with nonunion |
| S22.012S | Unstable burst fracture of first thoracic vertebra, sequela |
| S22.018 | Other fracture of first thoracic vertebra |
| S22.018A | Other fracture of first thoracic vertebra, initial encounter for closed fracture |
| S22.018B | Other fracture of first thoracic vertebra, initial encounter for open fracture |
| S22.018D | Other fracture of first thoracic vertebra, subsequent encounter for fracture with routine healing |
| S22.018G | Other fracture of first thoracic vertebra, subsequent encounter for fracture with delayed healing |
| S22.018K | Other fracture of first thoracic vertebra, subsequent encounter for fracture with nonunion |
| S22.018S | Other fracture of first thoracic vertebra, sequela |
| S22.019 | Unspecified fracture of first thoracic vertebra |
| S22.019A | Unspecified fracture of first thoracic vertebra, initial encounter for closed fracture |
| S22.019B | Unspecified fracture of first thoracic vertebra, initial encounter for open fracture |
| S22.019D | Unspecified fracture of first thoracic vertebra, subsequent encounter for fracture with routine healing |
| S22.019G | Unspecified fracture of first thoracic vertebra, subsequent encounter for fracture with delayed healing |
| S22.019K | Unspecified fracture of first thoracic vertebra, subsequent encounter for fracture with nonunion |
| S22.019S | Unspecified fracture of first thoracic vertebra, sequela |
| S22.02 | Fracture of second thoracic vertebra |
| S22.020 | Wedge compression fracture of second thoracic vertebra |
| S22.020A | Wedge compression fracture of second thoracic vertebra, initial encounter for closed fracture |
| S22.020B | Wedge compression fracture of second thoracic vertebra, initial encounter for open fracture |
| S22.020D | Wedge compression fracture of second thoracic vertebra, subsequent encounter for fracture with routine healing |
| S22.020G | Wedge compression fracture of second thoracic vertebra, subsequent encounter for fracture with delayed healing |
| S22.020K | Wedge compression fracture of second thoracic vertebra, subsequent encounter for fracture with nonunion |
| S22.020S | Wedge compression fracture of second thoracic vertebra, sequela |
| S22.021 | Stable burst fracture of second thoracic vertebra |
| S22.021A | Stable burst fracture of second thoracic vertebra, initial encounter for closed fracture |
| S22.021B | Stable burst fracture of second thoracic vertebra, initial encounter for open fracture |
| S22.021D | Stable burst fracture of second thoracic vertebra, subsequent encounter for fracture with routine healing |
| S22.021G | Stable burst fracture of second thoracic vertebra, subsequent encounter for fracture with delayed healing |
| S22.021K | Stable burst fracture of second thoracic vertebra, subsequent encounter for fracture with nonunion |
| S22.021S | Stable burst fracture of second thoracic vertebra, sequela |
| S22.022 | Unstable burst fracture of second thoracic vertebra |
| S22.022A | Unstable burst fracture of second thoracic vertebra, initial encounter for closed fracture |
| S22.022B | Unstable burst fracture of second thoracic vertebra, initial encounter for open fracture |
| S22.022D | Unstable burst fracture of second thoracic vertebra, subsequent encounter for fracture with routine healing |
| S22.022G | Unstable burst fracture of second thoracic vertebra, subsequent encounter for fracture with delayed healing |
| S22.022K | Unstable burst fracture of second thoracic vertebra, subsequent encounter for fracture with nonunion |
| S22.022S | Unstable burst fracture of second thoracic vertebra, sequela |
| S22.028 | Other fracture of second thoracic vertebra |
| S22.028A | Other fracture of second thoracic vertebra, initial encounter for closed fracture |
| S22.028B | Other fracture of second thoracic vertebra, initial encounter for open fracture |
| S22.028D | Other fracture of second thoracic vertebra, subsequent encounter for fracture with routine healing |
| S22.028G | Other fracture of second thoracic vertebra, subsequent encounter for fracture with delayed healing |
| S22.028K | Other fracture of second thoracic vertebra, subsequent encounter for fracture with nonunion |
| S22.028S | Other fracture of second thoracic vertebra, sequela |
| S22.029 | Unspecified fracture of second thoracic vertebra |
| S22.029A | Unspecified fracture of second thoracic vertebra, initial encounter for closed fracture |
| S22.029B | Unspecified fracture of second thoracic vertebra, initial encounter for open fracture |
| S22.029D | Unspecified fracture of second thoracic vertebra, subsequent encounter for fracture with routine healing |
| S22.029G | Unspecified fracture of second thoracic vertebra, subsequent encounter for fracture with delayed healing |
| S22.029K | Unspecified fracture of second thoracic vertebra, subsequent encounter for fracture with nonunion |
| S22.029S | Unspecified fracture of second thoracic vertebra, sequela |
| S22.03 | Fracture of third thoracic vertebra |
| S22.030 | Wedge compression fracture of third thoracic vertebra |
| S22.030A | Wedge compression fracture of third thoracic vertebra, initial encounter for closed fracture |
| S22.030B | Wedge compression fracture of third thoracic vertebra, initial encounter for open fracture |
| S22.030D | Wedge compression fracture of third thoracic vertebra, subsequent encounter for fracture with routine healing |
| S22.030G | Wedge compression fracture of third thoracic vertebra, subsequent encounter for fracture with delayed healing |
| S22.030K | Wedge compression fracture of third thoracic vertebra, subsequent encounter for fracture with nonunion |
| S22.030S | Wedge compression fracture of third thoracic vertebra, sequela |
| S22.031 | Stable burst fracture of third thoracic vertebra |
| S22.031A | Stable burst fracture of third thoracic vertebra, initial encounter for closed fracture |
| S22.031B | Stable burst fracture of third thoracic vertebra, initial encounter for open fracture |
| S22.031D | Stable burst fracture of third thoracic vertebra, subsequent encounter for fracture with routine healing |
| S22.031G | Stable burst fracture of third thoracic vertebra, subsequent encounter for fracture with delayed healing |
| S22.031K | Stable burst fracture of third thoracic vertebra, subsequent encounter for fracture with nonunion |
| S22.031S | Stable burst fracture of third thoracic vertebra, sequela |
| S22.032 | Unstable burst fracture of third thoracic vertebra |
| S22.032A | Unstable burst fracture of third thoracic vertebra, initial encounter for closed fracture |
| S22.032B | Unstable burst fracture of third thoracic vertebra, initial encounter for open fracture |
| S22.032D | Unstable burst fracture of third thoracic vertebra, subsequent encounter for fracture with routine healing |
| S22.032G | Unstable burst fracture of third thoracic vertebra, subsequent encounter for fracture with delayed healing |
| S22.032K | Unstable burst fracture of third thoracic vertebra, subsequent encounter for fracture with nonunion |
| S22.032S | Unstable burst fracture of third thoracic vertebra, sequela |
| S22.038 | Other fracture of third thoracic vertebra |
| S22.038A | Other fracture of third thoracic vertebra, initial encounter for closed fracture |
| S22.038B | Other fracture of third thoracic vertebra, initial encounter for open fracture |
| S22.038D | Other fracture of third thoracic vertebra, subsequent encounter for fracture with routine healing |
| S22.038G | Other fracture of third thoracic vertebra, subsequent encounter for fracture with delayed healing |
| S22.038K | Other fracture of third thoracic vertebra, subsequent encounter for fracture with nonunion |
| S22.038S | Other fracture of third thoracic vertebra, sequela |
| S22.039 | Unspecified fracture of third thoracic vertebra |
| S22.039A | Unspecified fracture of third thoracic vertebra, initial encounter for closed fracture |
| S22.039B | Unspecified fracture of third thoracic vertebra, initial encounter for open fracture |
| S22.039D | Unspecified fracture of third thoracic vertebra, subsequent encounter for fracture with routine healing |
| S22.039G | Unspecified fracture of third thoracic vertebra, subsequent encounter for fracture with delayed healing |
| S22.039K | Unspecified fracture of third thoracic vertebra, subsequent encounter for fracture with nonunion |
| S22.039S | Unspecified fracture of third thoracic vertebra, sequela |
| S22.04 | Fracture of fourth thoracic vertebra |
| S22.040 | Wedge compression fracture of fourth thoracic vertebra |
| S22.040A | Wedge compression fracture of fourth thoracic vertebra, initial encounter for closed fracture |
| S22.040B | Wedge compression fracture of fourth thoracic vertebra, initial encounter for open fracture |
| S22.040D | Wedge compression fracture of fourth thoracic vertebra, subsequent encounter for fracture with routine healing |
| S22.040G | Wedge compression fracture of fourth thoracic vertebra, subsequent encounter for fracture with delayed healing |
| S22.040K | Wedge compression fracture of fourth thoracic vertebra, subsequent encounter for fracture with nonunion |
| S22.040S | Wedge compression fracture of fourth thoracic vertebra, sequela |
| S22.041 | Stable burst fracture of fourth thoracic vertebra |
| S22.041A | Stable burst fracture of fourth thoracic vertebra, initial encounter for closed fracture |
| S22.041B | Stable burst fracture of fourth thoracic vertebra, initial encounter for open fracture |
| S22.041D | Stable burst fracture of fourth thoracic vertebra, subsequent encounter for fracture with routine healing |
| S22.041G | Stable burst fracture of fourth thoracic vertebra, subsequent encounter for fracture with delayed healing |
| S22.041K | Stable burst fracture of fourth thoracic vertebra, subsequent encounter for fracture with nonunion |
| S22.041S | Stable burst fracture of fourth thoracic vertebra, sequela |
| S22.042 | Unstable burst fracture of fourth thoracic vertebra |
| S22.042A | Unstable burst fracture of fourth thoracic vertebra, initial encounter for closed fracture |
| S22.042B | Unstable burst fracture of fourth thoracic vertebra, initial encounter for open fracture |
| S22.042D | Unstable burst fracture of fourth thoracic vertebra, subsequent encounter for fracture with routine healing |
| S22.042G | Unstable burst fracture of fourth thoracic vertebra, subsequent encounter for fracture with delayed healing |
| S22.042K | Unstable burst fracture of fourth thoracic vertebra, subsequent encounter for fracture with nonunion |
| S22.042S | Unstable burst fracture of fourth thoracic vertebra, sequela |
| S22.048 | Other fracture of fourth thoracic vertebra |
| S22.048A | Other fracture of fourth thoracic vertebra, initial encounter for closed fracture |
| S22.048B | Other fracture of fourth thoracic vertebra, initial encounter for open fracture |
| S22.048D | Other fracture of fourth thoracic vertebra, subsequent encounter for fracture with routine healing |
| S22.048G | Other fracture of fourth thoracic vertebra, subsequent encounter for fracture with delayed healing |
| S22.048K | Other fracture of fourth thoracic vertebra, subsequent encounter for fracture with nonunion |
| S22.048S | Other fracture of fourth thoracic vertebra, sequela |
| S22.049 | Unspecified fracture of fourth thoracic vertebra |
| S22.049A | Unspecified fracture of fourth thoracic vertebra, initial encounter for closed fracture |
| S22.049B | Unspecified fracture of fourth thoracic vertebra, initial encounter for open fracture |
| S22.049D | Unspecified fracture of fourth thoracic vertebra, subsequent encounter for fracture with routine healing |
| S22.049G | Unspecified fracture of fourth thoracic vertebra, subsequent encounter for fracture with delayed healing |
| S22.049K | Unspecified fracture of fourth thoracic vertebra, subsequent encounter for fracture with nonunion |
| S22.049S | Unspecified fracture of fourth thoracic vertebra, sequela |
| S22.05 | Fracture of T5-T6 vertebra |
| S22.050 | Wedge compression fracture of T5-T6 vertebra |
| S22.050A | Wedge compression fracture of T5-T6 vertebra, initial encounter for closed fracture |
| S22.050B | Wedge compression fracture of T5-T6 vertebra, initial encounter for open fracture |
| S22.050D | Wedge compression fracture of T5-T6 vertebra, subsequent encounter for fracture with routine healing |
| S22.050G | Wedge compression fracture of T5-T6 vertebra, subsequent encounter for fracture with delayed healing |
| S22.050K | Wedge compression fracture of T5-T6 vertebra, subsequent encounter for fracture with nonunion |
| S22.050S | Wedge compression fracture of T5-T6 vertebra, sequela |
| S22.051 | Stable burst fracture of T5-T6 vertebra |
| S22.051A | Stable burst fracture of T5-T6 vertebra, initial encounter for closed fracture |
| S22.051B | Stable burst fracture of T5-T6 vertebra, initial encounter for open fracture |
| S22.051D | Stable burst fracture of T5-T6 vertebra, subsequent encounter for fracture with routine healing |
| S22.051G | Stable burst fracture of T5-T6 vertebra, subsequent encounter for fracture with delayed healing |
| S22.051K | Stable burst fracture of T5-T6 vertebra, subsequent encounter for fracture with nonunion |
| S22.051S | Stable burst fracture of T5-T6 vertebra, sequela |
| S22.052 | Unstable burst fracture of T5-T6 vertebra |
| S22.052A | Unstable burst fracture of T5-T6 vertebra, initial encounter for closed fracture |
| S22.052B | Unstable burst fracture of T5-T6 vertebra, initial encounter for open fracture |
| S22.052D | Unstable burst fracture of T5-T6 vertebra, subsequent encounter for fracture with routine healing |
| S22.052G | Unstable burst fracture of T5-T6 vertebra, subsequent encounter for fracture with delayed healing |
| S22.052K | Unstable burst fracture of T5-T6 vertebra, subsequent encounter for fracture with nonunion |
| S22.052S | Unstable burst fracture of T5-T6 vertebra, sequela |
| S22.058 | Other fracture of T5-T6 vertebra |
| S22.058A | Other fracture of T5-T6 vertebra, initial encounter for closed fracture |
| S22.058B | Other fracture of T5-T6 vertebra, initial encounter for open fracture |
| S22.058D | Other fracture of T5-T6 vertebra, subsequent encounter for fracture with routine healing |
| S22.058G | Other fracture of T5-T6 vertebra, subsequent encounter for fracture with delayed healing |
| S22.058K | Other fracture of T5-T6 vertebra, subsequent encounter for fracture with nonunion |
| S22.058S | Other fracture of T5-T6 vertebra, sequela |
| S22.059 | Unspecified fracture of T5-T6 vertebra |
| S22.059A | Unspecified fracture of T5-T6 vertebra, initial encounter for closed fracture |
| S22.059B | Unspecified fracture of T5-T6 vertebra, initial encounter for open fracture |
| S22.059D | Unspecified fracture of T5-T6 vertebra, subsequent encounter for fracture with routine healing |
| S22.059G | Unspecified fracture of T5-T6 vertebra, subsequent encounter for fracture with delayed healing |
| S22.059K | Unspecified fracture of T5-T6 vertebra, subsequent encounter for fracture with nonunion |
| S22.059S | Unspecified fracture of T5-T6 vertebra, sequela |
| S22.06 | Fracture of T7-T8 vertebra |
| S22.060 | Wedge compression fracture of T7-T8 vertebra |
| S22.060A | Wedge compression fracture of T7-T8 vertebra, initial encounter for closed fracture |
| S22.060B | Wedge compression fracture of T7-T8 vertebra, initial encounter for open fracture |
| S22.060D | Wedge compression fracture of T7-T8 vertebra, subsequent encounter for fracture with routine healing |
| S22.060G | Wedge compression fracture of T7-T8 vertebra, subsequent encounter for fracture with delayed healing |
| S22.060K | Wedge compression fracture of T7-T8 vertebra, subsequent encounter for fracture with nonunion |
| S22.060S | Wedge compression fracture of T7-T8 vertebra, sequela |
| S22.061 | Stable burst fracture of T7-T8 vertebra |
| S22.061A | Stable burst fracture of T7-T8 vertebra, initial encounter for closed fracture |
| S22.061B | Stable burst fracture of T7-T8 vertebra, initial encounter for open fracture |
| S22.061D | Stable burst fracture of T7-T8 vertebra, subsequent encounter for fracture with routine healing |
| S22.061G | Stable burst fracture of T7-T8 vertebra, subsequent encounter for fracture with delayed healing |
| S22.061K | Stable burst fracture of T7-T8 vertebra, subsequent encounter for fracture with nonunion |
| S22.061S | Stable burst fracture of T7-T8 vertebra, sequela |
| S22.062 | Unstable burst fracture of T7-T8 vertebra |
| S22.062A | Unstable burst fracture of T7-T8 vertebra, initial encounter for closed fracture |
| S22.062B | Unstable burst fracture of T7-T8 vertebra, initial encounter for open fracture |
| S22.062D | Unstable burst fracture of T7-T8 vertebra, subsequent encounter for fracture with routine healing |
| S22.062G | Unstable burst fracture of T7-T8 vertebra, subsequent encounter for fracture with delayed healing |
| S22.062K | Unstable burst fracture of T7-T8 vertebra, subsequent encounter for fracture with nonunion |
| S22.062S | Unstable burst fracture of T7-T8 vertebra, sequela |
| S22.068 | Other fracture of T7-T8 thoracic vertebra |
| S22.068A | Other fracture of T7-T8 thoracic vertebra, initial encounter for closed fracture |
| S22.068B | Other fracture of T7-T8 thoracic vertebra, initial encounter for open fracture |
| S22.068D | Other fracture of T7-T8 thoracic vertebra, subsequent encounter for fracture with routine healing |
| S22.068G | Other fracture of T7-T8 thoracic vertebra, subsequent encounter for fracture with delayed healing |
| S22.068K | Other fracture of T7-T8 thoracic vertebra, subsequent encounter for fracture with nonunion |
| S22.068S | Other fracture of T7-T8 thoracic vertebra, sequela |
| S22.069 | Unspecified fracture of T7-T8 vertebra |
| S22.069A | Unspecified fracture of T7-T8 vertebra, initial encounter for closed fracture |
| S22.069B | Unspecified fracture of T7-T8 vertebra, initial encounter for open fracture |
| S22.069D | Unspecified fracture of T7-T8 vertebra, subsequent encounter for fracture with routine healing |
| S22.069G | Unspecified fracture of T7-T8 vertebra, subsequent encounter for fracture with delayed healing |
| S22.069K | Unspecified fracture of T7-T8 vertebra, subsequent encounter for fracture with nonunion |
| S22.069S | Unspecified fracture of T7-T8 vertebra, sequela |
| S22.07 | Fracture of T9-T10 vertebra |
| S22.070 | Wedge compression fracture of T9-T10 vertebra |
| S22.070A | Wedge compression fracture of T9-T10 vertebra, initial encounter for closed fracture |
| S22.070B | Wedge compression fracture of T9-T10 vertebra, initial encounter for open fracture |
| S22.070D | Wedge compression fracture of T9-T10 vertebra, subsequent encounter for fracture with routine healing |
| S22.070G | Wedge compression fracture of T9-T10 vertebra, subsequent encounter for fracture with delayed healing |
| S22.070K | Wedge compression fracture of T9-T10 vertebra, subsequent encounter for fracture with nonunion |
| S22.070S | Wedge compression fracture of T9-T10 vertebra, sequela |
| S22.071 | Stable burst fracture of T9-T10 vertebra |
| S22.071A | Stable burst fracture of T9-T10 vertebra, initial encounter for closed fracture |
| S22.071B | Stable burst fracture of T9-T10 vertebra, initial encounter for open fracture |
| S22.071D | Stable burst fracture of T9-T10 vertebra, subsequent encounter for fracture with routine healing |
| S22.071G | Stable burst fracture of T9-T10 vertebra, subsequent encounter for fracture with delayed healing |
| S22.071K | Stable burst fracture of T9-T10 vertebra, subsequent encounter for fracture with nonunion |
| S22.071S | Stable burst fracture of T9-T10 vertebra, sequela |
| S22.072 | Unstable burst fracture of T9-T10 vertebra |
| S22.072A | Unstable burst fracture of T9-T10 vertebra, initial encounter for closed fracture |
| S22.072B | Unstable burst fracture of T9-T10 vertebra, initial encounter for open fracture |
| S22.072D | Unstable burst fracture of T9-T10 vertebra, subsequent encounter for fracture with routine healing |
| S22.072G | Unstable burst fracture of T9-T10 vertebra, subsequent encounter for fracture with delayed healing |
| S22.072K | Unstable burst fracture of T9-T10 vertebra, subsequent encounter for fracture with nonunion |
| S22.072S | Unstable burst fracture of T9-T10 vertebra, sequela |
| S22.078 | Other fracture of T9-T10 vertebra |
| S22.078A | Other fracture of T9-T10 vertebra, initial encounter for closed fracture |
| S22.078B | Other fracture of T9-T10 vertebra, initial encounter for open fracture |
| S22.078D | Other fracture of T9-T10 vertebra, subsequent encounter for fracture with routine healing |
| S22.078G | Other fracture of T9-T10 vertebra, subsequent encounter for fracture with delayed healing |
| S22.078K | Other fracture of T9-T10 vertebra, subsequent encounter for fracture with nonunion |
| S22.078S | Other fracture of T9-T10 vertebra, sequela |
| S22.079 | Unspecified fracture of T9-T10 vertebra |
| S22.079A | Unspecified fracture of T9-T10 vertebra, initial encounter for closed fracture |
| S22.079B | Unspecified fracture of T9-T10 vertebra, initial encounter for open fracture |
| S22.079D | Unspecified fracture of T9-T10 vertebra, subsequent encounter for fracture with routine healing |
| S22.079G | Unspecified fracture of T9-T10 vertebra, subsequent encounter for fracture with delayed healing |
| S22.079K | Unspecified fracture of T9-T10 vertebra, subsequent encounter for fracture with nonunion |
| S22.079S | Unspecified fracture of T9-T10 vertebra, sequela |
| S22.08 | Fracture of T11-T12 vertebra |
| S22.080 | Wedge compression fracture of T11-T12 vertebra |
| S22.080A | Wedge compression fracture of T11-T12 vertebra, initial encounter for closed fracture |
| S22.080B | Wedge compression fracture of T11-T12 vertebra, initial encounter for open fracture |
| S22.080D | Wedge compression fracture of T11-T12 vertebra, subsequent encounter for fracture with routine healing |
| S22.080G | Wedge compression fracture of T11-T12 vertebra, subsequent encounter for fracture with delayed healing |
| S22.080K | Wedge compression fracture of T11-T12 vertebra, subsequent encounter for fracture with nonunion |
| S22.080S | Wedge compression fracture of T11-T12 vertebra, sequela |
| S22.081 | Stable burst fracture of T11-T12 vertebra |
| S22.081A | Stable burst fracture of T11-T12 vertebra, initial encounter for closed fracture |
| S22.081B | Stable burst fracture of T11-T12 vertebra, initial encounter for open fracture |
| S22.081D | Stable burst fracture of T11-T12 vertebra, subsequent encounter for fracture with routine healing |
| S22.081G | Stable burst fracture of T11-T12 vertebra, subsequent encounter for fracture with delayed healing |
| S22.081K | Stable burst fracture of T11-T12 vertebra, subsequent encounter for fracture with nonunion |
| S22.081S | Stable burst fracture of T11-T12 vertebra, sequela |
| S22.082 | Unstable burst fracture of T11-T12 vertebra |
| S22.082A | Unstable burst fracture of T11-T12 vertebra, initial encounter for closed fracture |
| S22.082B | Unstable burst fracture of T11-T12 vertebra, initial encounter for open fracture |
| S22.082D | Unstable burst fracture of T11-T12 vertebra, subsequent encounter for fracture with routine healing |
| S22.082G | Unstable burst fracture of T11-T12 vertebra, subsequent encounter for fracture with delayed healing |
| S22.082K | Unstable burst fracture of T11-T12 vertebra, subsequent encounter for fracture with nonunion |
| S22.082S | Unstable burst fracture of T11-T12 vertebra, sequela |
| S22.088 | Other fracture of T11-T12 vertebra |
| S22.088A | Other fracture of T11-T12 vertebra, initial encounter for closed fracture |
| S22.088B | Other fracture of T11-T12 vertebra, initial encounter for open fracture |
| S22.088D | Other fracture of T11-T12 vertebra, subsequent encounter for fracture with routine healing |
| S22.088G | Other fracture of T11-T12 vertebra, subsequent encounter for fracture with delayed healing |
| S22.088K | Other fracture of T11-T12 vertebra, subsequent encounter for fracture with nonunion |
| S22.088S | Other fracture of T11-T12 vertebra, sequela |
| S22.089 | Unspecified fracture of T11-T12 vertebra |
| S22.089A | Unspecified fracture of T11-T12 vertebra, initial encounter for closed fracture |
| S22.089B | Unspecified fracture of T11-T12 vertebra, initial encounter for open fracture |
| S22.089D | Unspecified fracture of T11-T12 vertebra, subsequent encounter for fracture with routine healing |
| S22.089G | Unspecified fracture of T11-T12 vertebra, subsequent encounter for fracture with delayed healing |
| S22.089K | Unspecified fracture of T11-T12 vertebra, subsequent encounter for fracture with nonunion |
| S22.089S | Unspecified fracture of T11-T12 vertebra, sequela |
| S22.2 | Fracture of sternum |
| S22.20 | Unspecified fracture of sternum |
| S22.20XA | Unspecified fracture of sternum, initial encounter for closed fracture |
| S22.20XB | Unspecified fracture of sternum, initial encounter for open fracture |
| S22.20XD | Unspecified fracture of sternum, subsequent encounter for fracture with routine healing |
| S22.20XG | Unspecified fracture of sternum, subsequent encounter for fracture with delayed healing |
| S22.20XK | Unspecified fracture of sternum, subsequent encounter for fracture with nonunion |
| S22.20XS | Unspecified fracture of sternum, sequela |
| S22.21 | Fracture of manubrium |
| S22.21XA | Fracture of manubrium, initial encounter for closed fracture |
| S22.21XB | Fracture of manubrium, initial encounter for open fracture |
| S22.21XD | Fracture of manubrium, subsequent encounter for fracture with routine healing |
| S22.21XG | Fracture of manubrium, subsequent encounter for fracture with delayed healing |
| S22.21XK | Fracture of manubrium, subsequent encounter for fracture with nonunion |
| S22.21XS | Fracture of manubrium, sequela |
| S22.22 | Fracture of body of sternum |
| S22.22XA | Fracture of body of sternum, initial encounter for closed fracture |
| S22.22XB | Fracture of body of sternum, initial encounter for open fracture |
| S22.22XD | Fracture of body of sternum, subsequent encounter for fracture with routine healing |
| S22.22XG | Fracture of body of sternum, subsequent encounter for fracture with delayed healing |
| S22.22XK | Fracture of body of sternum, subsequent encounter for fracture with nonunion |
| S22.22XS | Fracture of body of sternum, sequela |
| S22.23 | Sternal manubrial dissociation |
| S22.23XA | Sternal manubrial dissociation, initial encounter for closed fracture |
| S22.23XB | Sternal manubrial dissociation, initial encounter for open fracture |
| S22.23XD | Sternal manubrial dissociation, subsequent encounter for fracture with routine healing |
| S22.23XG | Sternal manubrial dissociation, subsequent encounter for fracture with delayed healing |
| S22.23XK | Sternal manubrial dissociation, subsequent encounter for fracture with nonunion |
| S22.23XS | Sternal manubrial dissociation, sequela |
| S22.24 | Fracture of xiphoid process |
| S22.24XA | Fracture of xiphoid process, initial encounter for closed fracture |
| S22.24XB | Fracture of xiphoid process, initial encounter for open fracture |
| S22.24XD | Fracture of xiphoid process, subsequent encounter for fracture with routine healing |
| S22.24XG | Fracture of xiphoid process, subsequent encounter for fracture with delayed healing |
| S22.24XK | Fracture of xiphoid process, subsequent encounter for fracture with nonunion |
| S22.24XS | Fracture of xiphoid process, sequela |
| S22.3 | Fracture of one rib |
| S22.31 | Fracture of one rib, right side |
| S22.31XA | Fracture of one rib, right side, initial encounter for closed fracture |
| S22.31XB | Fracture of one rib, right side, initial encounter for open fracture |
| S22.31XD | Fracture of one rib, right side, subsequent encounter for fracture with routine healing |
| S22.31XG | Fracture of one rib, right side, subsequent encounter for fracture with delayed healing |
| S22.31XK | Fracture of one rib, right side, subsequent encounter for fracture with nonunion |
| S22.31XS | Fracture of one rib, right side, sequela |
| S22.32 | Fracture of one rib, left side |
| S22.32XA | Fracture of one rib, left side, initial encounter for closed fracture |
| S22.32XB | Fracture of one rib, left side, initial encounter for open fracture |
| S22.32XD | Fracture of one rib, left side, subsequent encounter for fracture with routine healing |
| S22.32XG | Fracture of one rib, left side, subsequent encounter for fracture with delayed healing |
| S22.32XK | Fracture of one rib, left side, subsequent encounter for fracture with nonunion |
| S22.32XS | Fracture of one rib, left side, sequela |
| S22.39 | Fracture of one rib, unspecified side |
| S22.39XA | Fracture of one rib, unspecified side, initial encounter for closed fracture |
| S22.39XB | Fracture of one rib, unspecified side, initial encounter for open fracture |
| S22.39XD | Fracture of one rib, unspecified side, subsequent encounter for fracture with routine healing |
| S22.39XG | Fracture of one rib, unspecified side, subsequent encounter for fracture with delayed healing |
| S22.39XK | Fracture of one rib, unspecified side, subsequent encounter for fracture with nonunion |
| S22.39XS | Fracture of one rib, unspecified side, sequela |
| S22.4 | Multiple fractures of ribs |
| S22.41 | Multiple fractures of ribs, right side |
| S22.41XA | Multiple fractures of ribs, right side, initial encounter for closed fracture |
| S22.41XB | Multiple fractures of ribs, right side, initial encounter for open fracture |
| S22.41XD | Multiple fractures of ribs, right side, subsequent encounter for fracture with routine healing |
| S22.41XG | Multiple fractures of ribs, right side, subsequent encounter for fracture with delayed healing |
| S22.41XK | Multiple fractures of ribs, right side, subsequent encounter for fracture with nonunion |
| S22.41XS | Multiple fractures of ribs, right side, sequela |
| S22.42 | Multiple fractures of ribs, left side |
| S22.42XA | Multiple fractures of ribs, left side, initial encounter for closed fracture |
| S22.42XB | Multiple fractures of ribs, left side, initial encounter for open fracture |
| S22.42XD | Multiple fractures of ribs, left side, subsequent encounter for fracture with routine healing |
| S22.42XG | Multiple fractures of ribs, left side, subsequent encounter for fracture with delayed healing |
| S22.42XK | Multiple fractures of ribs, left side, subsequent encounter for fracture with nonunion |
| S22.42XS | Multiple fractures of ribs, left side, sequela |
| S22.43 | Multiple fractures of ribs, bilateral |
| S22.43XA | Multiple fractures of ribs, bilateral, initial encounter for closed fracture |
| S22.43XB | Multiple fractures of ribs, bilateral, initial encounter for open fracture |
| S22.43XD | Multiple fractures of ribs, bilateral, subsequent encounter for fracture with routine healing |
| S22.43XG | Multiple fractures of ribs, bilateral, subsequent encounter for fracture with delayed healing |
| S22.43XK | Multiple fractures of ribs, bilateral, subsequent encounter for fracture with nonunion |
| S22.43XS | Multiple fractures of ribs, bilateral, sequela |
| S22.49 | Multiple fractures of ribs, unspecified side |
| S22.49XA | Multiple fractures of ribs, unspecified side, initial encounter for closed fracture |
| S22.49XB | Multiple fractures of ribs, unspecified side, initial encounter for open fracture |
| S22.49XD | Multiple fractures of ribs, unspecified side, subsequent encounter for fracture with routine healing |
| S22.49XG | Multiple fractures of ribs, unspecified side, subsequent encounter for fracture with delayed healing |
| S22.49XK | Multiple fractures of ribs, unspecified side, subsequent encounter for fracture with nonunion |
| S22.49XS | Multiple fractures of ribs, unspecified side, sequela |
| S22.5 | Flail chest |
| S22.5XXA | Flail chest, initial encounter for closed fracture |
| S22.5XXB | Flail chest, initial encounter for open fracture |
| S22.5XXD | Flail chest, subsequent encounter for fracture with routine healing |
| S22.5XXG | Flail chest, subsequent encounter for fracture with delayed healing |
| S22.5XXK | Flail chest, subsequent encounter for fracture with nonunion |
| S22.5XXS | Flail chest, sequela |
| S22.9 | Fracture of bony thorax, part unspecified |
| S22.9XXA | Fracture of bony thorax, part unspecified, initial encounter for closed fracture |
| S22.9XXB | Fracture of bony thorax, part unspecified, initial encounter for open fracture |
| S22.9XXD | Fracture of bony thorax, part unspecified, subsequent encounter for fracture with routine healing |
| S22.9XXG | Fracture of bony thorax, part unspecified, subsequent encounter for fracture with delayed healing |
| S22.9XXK | Fracture of bony thorax, part unspecified, subsequent encounter for fracture with nonunion |
| S22.9XXS | Fracture of bony thorax, part unspecified, sequela |
| S23 | Dislocation and sprain of joints and ligaments of thorax |
| S23.0 | Traumatic rupture of thoracic intervertebral disc |
| S23.0XXA | Traumatic rupture of thoracic intervertebral disc, initial encounter |
| S23.0XXD | Traumatic rupture of thoracic intervertebral disc, subsequent encounter |
| S23.0XXS | Traumatic rupture of thoracic intervertebral disc, sequela |
| S23.1 | Subluxation and dislocation of thoracic vertebra |
| S23.10 | Subluxation and dislocation of unspecified thoracic vertebra |
| S23.100 | Subluxation of unspecified thoracic vertebra |
| S23.100A | Subluxation of unspecified thoracic vertebra, initial encounter |
| S23.100D | Subluxation of unspecified thoracic vertebra, subsequent encounter |
| S23.100S | Subluxation of unspecified thoracic vertebra, sequela |
| S23.101 | Dislocation of unspecified thoracic vertebra |
| S23.101A | Dislocation of unspecified thoracic vertebra, initial encounter |
| S23.101D | Dislocation of unspecified thoracic vertebra, subsequent encounter |
| S23.101S | Dislocation of unspecified thoracic vertebra, sequela |
| S23.11 | Subluxation and dislocation of T1/T2 thoracic vertebra |
| S23.110 | Subluxation of T1/T2 thoracic vertebra |
| S23.110A | Subluxation of T1/T2 thoracic vertebra, initial encounter |
| S23.110D | Subluxation of T1/T2 thoracic vertebra, subsequent encounter |
| S23.110S | Subluxation of T1/T2 thoracic vertebra, sequela |
| S23.111 | Dislocation of T1/T2 thoracic vertebra |
| S23.111A | Dislocation of T1/T2 thoracic vertebra, initial encounter |
| S23.111D | Dislocation of T1/T2 thoracic vertebra, subsequent encounter |
| S23.111S | Dislocation of T1/T2 thoracic vertebra, sequela |
| S23.12 | Subluxation and dislocation of T2/T3-T3/T4 thoracic vertebra |
| S23.120 | Subluxation of T2/T3 thoracic vertebra |
| S23.120A | Subluxation of T2/T3 thoracic vertebra, initial encounter |
| S23.120D | Subluxation of T2/T3 thoracic vertebra, subsequent encounter |
| S23.120S | Subluxation of T2/T3 thoracic vertebra, sequela |
| S23.121 | Dislocation of T2/T3 thoracic vertebra |
| S23.121A | Dislocation of T2/T3 thoracic vertebra, initial encounter |
| S23.121D | Dislocation of T2/T3 thoracic vertebra, subsequent encounter |
| S23.121S | Dislocation of T2/T3 thoracic vertebra, sequela |
| S23.122 | Subluxation of T3/T4 thoracic vertebra |
| S23.122A | Subluxation of T3/T4 thoracic vertebra, initial encounter |
| S23.122D | Subluxation of T3/T4 thoracic vertebra, subsequent encounter |
| S23.122S | Subluxation of T3/T4 thoracic vertebra, sequela |
| S23.123 | Dislocation of T3/T4 thoracic vertebra |
| S23.123A | Dislocation of T3/T4 thoracic vertebra, initial encounter |
| S23.123D | Dislocation of T3/T4 thoracic vertebra, subsequent encounter |
| S23.123S | Dislocation of T3/T4 thoracic vertebra, sequela |
| S23.13 | Subluxation and dislocation of T4/T5-T5/T6 thoracic vertebra |
| S23.130 | Subluxation of T4/T5 thoracic vertebra |
| S23.130A | Subluxation of T4/T5 thoracic vertebra, initial encounter |
| S23.130D | Subluxation of T4/T5 thoracic vertebra, subsequent encounter |
| S23.130S | Subluxation of T4/T5 thoracic vertebra, sequela |
| S23.131 | Dislocation of T4/T5 thoracic vertebra |
| S23.131A | Dislocation of T4/T5 thoracic vertebra, initial encounter |
| S23.131D | Dislocation of T4/T5 thoracic vertebra, subsequent encounter |
| S23.131S | Dislocation of T4/T5 thoracic vertebra, sequela |
| S23.132 | Subluxation of T5/T6 thoracic vertebra |
| S23.132A | Subluxation of T5/T6 thoracic vertebra, initial encounter |
| S23.132D | Subluxation of T5/T6 thoracic vertebra, subsequent encounter |
| S23.132S | Subluxation of T5/T6 thoracic vertebra, sequela |
| S23.133 | Dislocation of T5/T6 thoracic vertebra |
| S23.133A | Dislocation of T5/T6 thoracic vertebra, initial encounter |
| S23.133D | Dislocation of T5/T6 thoracic vertebra, subsequent encounter |
| S23.133S | Dislocation of T5/T6 thoracic vertebra, sequela |
| S23.14 | Subluxation and dislocation of T6/T7-T7/T8 thoracic vertebra |
| S23.140 | Subluxation of T6/T7 thoracic vertebra |
| S23.140A | Subluxation of T6/T7 thoracic vertebra, initial encounter |
| S23.140D | Subluxation of T6/T7 thoracic vertebra, subsequent encounter |
| S23.140S | Subluxation of T6/T7 thoracic vertebra, sequela |
| S23.141 | Dislocation of T6/T7 thoracic vertebra |
| S23.141A | Dislocation of T6/T7 thoracic vertebra, initial encounter |
| S23.141D | Dislocation of T6/T7 thoracic vertebra, subsequent encounter |
| S23.141S | Dislocation of T6/T7 thoracic vertebra, sequela |
| S23.142 | Subluxation of T7/T8 thoracic vertebra |
| S23.142A | Subluxation of T7/T8 thoracic vertebra, initial encounter |
| S23.142D | Subluxation of T7/T8 thoracic vertebra, subsequent encounter |
| S23.142S | Subluxation of T7/T8 thoracic vertebra, sequela |
| S23.143 | Dislocation of T7/T8 thoracic vertebra |
| S23.143A | Dislocation of T7/T8 thoracic vertebra, initial encounter |
| S23.143D | Dislocation of T7/T8 thoracic vertebra, subsequent encounter |
| S23.143S | Dislocation of T7/T8 thoracic vertebra, sequela |
| S23.15 | Subluxation and dislocation of T8/T9-T9/T10 thoracic vertebra |
| S23.150 | Subluxation of T8/T9 thoracic vertebra |
| S23.150A | Subluxation of T8/T9 thoracic vertebra, initial encounter |
| S23.150D | Subluxation of T8/T9 thoracic vertebra, subsequent encounter |
| S23.150S | Subluxation of T8/T9 thoracic vertebra, sequela |
| S23.151 | Dislocation of T8/T9 thoracic vertebra |
| S23.151A | Dislocation of T8/T9 thoracic vertebra, initial encounter |
| S23.151D | Dislocation of T8/T9 thoracic vertebra, subsequent encounter |
| S23.151S | Dislocation of T8/T9 thoracic vertebra, sequela |
| S23.152 | Subluxation of T9/T10 thoracic vertebra |
| S23.152A | Subluxation of T9/T10 thoracic vertebra, initial encounter |
| S23.152D | Subluxation of T9/T10 thoracic vertebra, subsequent encounter |
| S23.152S | Subluxation of T9/T10 thoracic vertebra, sequela |
| S23.153 | Dislocation of T9/T10 thoracic vertebra |
| S23.153A | Dislocation of T9/T10 thoracic vertebra, initial encounter |
| S23.153D | Dislocation of T9/T10 thoracic vertebra, subsequent encounter |
| S23.153S | Dislocation of T9/T10 thoracic vertebra, sequela |
| S23.16 | Subluxation and dislocation of T10/T11-T11/T12 thoracic vertebra |
| S23.160 | Subluxation of T10/T11 thoracic vertebra |
| S23.160A | Subluxation of T10/T11 thoracic vertebra, initial encounter |
| S23.160D | Subluxation of T10/T11 thoracic vertebra, subsequent encounter |
| S23.160S | Subluxation of T10/T11 thoracic vertebra, sequela |
| S23.161 | Dislocation of T10/T11 thoracic vertebra |
| S23.161A | Dislocation of T10/T11 thoracic vertebra, initial encounter |
| S23.161D | Dislocation of T10/T11 thoracic vertebra, subsequent encounter |
| S23.161S | Dislocation of T10/T11 thoracic vertebra, sequela |
| S23.162 | Subluxation of T11/T12 thoracic vertebra |
| S23.162A | Subluxation of T11/T12 thoracic vertebra, initial encounter |
| S23.162D | Subluxation of T11/T12 thoracic vertebra, subsequent encounter |
| S23.162S | Subluxation of T11/T12 thoracic vertebra, sequela |
| S23.163 | Dislocation of T11/T12 thoracic vertebra |
| S23.163A | Dislocation of T11/T12 thoracic vertebra, initial encounter |
| S23.163D | Dislocation of T11/T12 thoracic vertebra, subsequent encounter |
| S23.163S | Dislocation of T11/T12 thoracic vertebra, sequela |
| S23.17 | Subluxation and dislocation of T12/L1 thoracic vertebra |
| S23.170 | Subluxation of T12/L1 thoracic vertebra |
| S23.170A | Subluxation of T12/L1 thoracic vertebra, initial encounter |
| S23.170D | Subluxation of T12/L1 thoracic vertebra, subsequent encounter |
| S23.170S | Subluxation of T12/L1 thoracic vertebra, sequela |
| S23.171 | Dislocation of T12/L1 thoracic vertebra |
| S23.171A | Dislocation of T12/L1 thoracic vertebra, initial encounter |
| S23.171D | Dislocation of T12/L1 thoracic vertebra, subsequent encounter |
| S23.171S | Dislocation of T12/L1 thoracic vertebra, sequela |
| S23.2 | Dislocation of other and unspecified parts of thorax |
| S23.20 | Dislocation of unspecified part of thorax |
| S23.20XA | Dislocation of unspecified part of thorax, initial encounter |
| S23.20XD | Dislocation of unspecified part of thorax, subsequent encounter |
| S23.20XS | Dislocation of unspecified part of thorax, sequela |
| S23.29 | Dislocation of other parts of thorax |
| S23.29XA | Dislocation of other parts of thorax, initial encounter |
| S23.29XD | Dislocation of other parts of thorax, subsequent encounter |
| S23.29XS | Dislocation of other parts of thorax, sequela |
| S23.3 | Sprain of ligaments of thoracic spine |
| S23.3XXA | Sprain of ligaments of thoracic spine, initial encounter |
| S23.3XXD | Sprain of ligaments of thoracic spine, subsequent encounter |
| S23.3XXS | Sprain of ligaments of thoracic spine, sequela |
| S23.4 | Sprain of ribs and sternum |
| S23.41 | Sprain of ribs |
| S23.41XA | Sprain of ribs, initial encounter |
| S23.41XD | Sprain of ribs, subsequent encounter |
| S23.41XS | Sprain of ribs, sequela |
| S23.42 | Sprain of sternum |
| S23.420 | Sprain of sternoclavicular (joint) (ligament) |
| S23.420A | Sprain of sternoclavicular (joint) (ligament), initial encounter |
| S23.420D | Sprain of sternoclavicular (joint) (ligament), subsequent encounter |
| S23.420S | Sprain of sternoclavicular (joint) (ligament), sequela |
| S23.421 | Sprain of chondrosternal joint |
| S23.421A | Sprain of chondrosternal joint, initial encounter |
| S23.421D | Sprain of chondrosternal joint, subsequent encounter |
| S23.421S | Sprain of chondrosternal joint, sequela |
| S23.428 | Other sprain of sternum |
| S23.428A | Other sprain of sternum, initial encounter |
| S23.428D | Other sprain of sternum, subsequent encounter |
| S23.428S | Other sprain of sternum, sequela |
| S23.429 | Unspecified sprain of sternum |
| S23.429A | Unspecified sprain of sternum, initial encounter |
| S23.429D | Unspecified sprain of sternum, subsequent encounter |
| S23.429S | Unspecified sprain of sternum, sequela |
| S23.8 | Sprain of other specified parts of thorax |
| S23.8XXA | Sprain of other specified parts of thorax, initial encounter |
| S23.8XXD | Sprain of other specified parts of thorax, subsequent encounter |
| S23.8XXS | Sprain of other specified parts of thorax, sequela |
| S23.9 | Sprain of unspecified parts of thorax |
| S23.9XXA | Sprain of unspecified parts of thorax, initial encounter |
| S23.9XXD | Sprain of unspecified parts of thorax, subsequent encounter |
| S23.9XXS | Sprain of unspecified parts of thorax, sequela |
| S24 | Injury of nerves and spinal cord at thorax level |
| S24.0 | Concussion and edema of thoracic spinal cord |
| S24.0XXA | Concussion and edema of thoracic spinal cord, initial encounter |
| S24.0XXD | Concussion and edema of thoracic spinal cord, subsequent encounter |
| S24.0XXS | Concussion and edema of thoracic spinal cord, sequela |
| S24.1 | Other and unspecified injuries of thoracic spinal cord |
| S24.10 | Unspecified injury of thoracic spinal cord |
| S24.101 | Unspecified injury at T1 level of thoracic spinal cord |
| S24.101A | Unspecified injury at T1 level of thoracic spinal cord, initial encounter |
| S24.101D | Unspecified injury at T1 level of thoracic spinal cord, subsequent encounter |
| S24.101S | Unspecified injury at T1 level of thoracic spinal cord, sequela |
| S24.102 | Unspecified injury at T2-T6 level of thoracic spinal cord |
| S24.102A | Unspecified injury at T2-T6 level of thoracic spinal cord, initial encounter |
| S24.102D | Unspecified injury at T2-T6 level of thoracic spinal cord, subsequent encounter |
| S24.102S | Unspecified injury at T2-T6 level of thoracic spinal cord, sequela |
| S24.103 | Unspecified injury at T7-T10 level of thoracic spinal cord |
| S24.103A | Unspecified injury at T7-T10 level of thoracic spinal cord, initial encounter |
| S24.103D | Unspecified injury at T7-T10 level of thoracic spinal cord, subsequent encounter |
| S24.103S | Unspecified injury at T7-T10 level of thoracic spinal cord, sequela |
| S24.104 | Unspecified injury at T11-T12 level of thoracic spinal cord |
| S24.104A | Unspecified injury at T11-T12 level of thoracic spinal cord, initial encounter |
| S24.104D | Unspecified injury at T11-T12 level of thoracic spinal cord, subsequent encounter |
| S24.104S | Unspecified injury at T11-T12 level of thoracic spinal cord, sequela |
| S24.109 | Unspecified injury at unspecified level of thoracic spinal cord |
| S24.109A | Unspecified injury at unspecified level of thoracic spinal cord, initial encounter |
| S24.109D | Unspecified injury at unspecified level of thoracic spinal cord, subsequent encounter |
| S24.109S | Unspecified injury at unspecified level of thoracic spinal cord, sequela |
| S24.11 | Complete lesion of thoracic spinal cord |
| S24.111 | Complete lesion at T1 level of thoracic spinal cord |
| S24.111A | Complete lesion at T1 level of thoracic spinal cord, initial encounter |
| S24.111D | Complete lesion at T1 level of thoracic spinal cord, subsequent encounter |
| S24.111S | Complete lesion at T1 level of thoracic spinal cord, sequela |
| S24.112 | Complete lesion at T2-T6 level of thoracic spinal cord |
| S24.112A | Complete lesion at T2-T6 level of thoracic spinal cord, initial encounter |
| S24.112D | Complete lesion at T2-T6 level of thoracic spinal cord, subsequent encounter |
| S24.112S | Complete lesion at T2-T6 level of thoracic spinal cord, sequela |
| S24.113 | Complete lesion at T7-T10 level of thoracic spinal cord |
| S24.113A | Complete lesion at T7-T10 level of thoracic spinal cord, initial encounter |
| S24.113D | Complete lesion at T7-T10 level of thoracic spinal cord, subsequent encounter |
| S24.113S | Complete lesion at T7-T10 level of thoracic spinal cord, sequela |
| S24.114 | Complete lesion at T11-T12 level of thoracic spinal cord |
| S24.114A | Complete lesion at T11-T12 level of thoracic spinal cord, initial encounter |
| S24.114D | Complete lesion at T11-T12 level of thoracic spinal cord, subsequent encounter |
| S24.114S | Complete lesion at T11-T12 level of thoracic spinal cord, sequela |
| S24.119 | Complete lesion at unspecified level of thoracic spinal cord |
| S24.119A | Complete lesion at unspecified level of thoracic spinal cord, initial encounter |
| S24.119D | Complete lesion at unspecified level of thoracic spinal cord, subsequent encounter |
| S24.119S | Complete lesion at unspecified level of thoracic spinal cord, sequela |
| S24.13 | Anterior cord syndrome of thoracic spinal cord |
| S24.131 | Anterior cord syndrome at T1 level of thoracic spinal cord |
| S24.131A | Anterior cord syndrome at T1 level of thoracic spinal cord, initial encounter |
| S24.131D | Anterior cord syndrome at T1 level of thoracic spinal cord, subsequent encounter |
| S24.131S | Anterior cord syndrome at T1 level of thoracic spinal cord, sequela |
| S24.132 | Anterior cord syndrome at T2-T6 level of thoracic spinal cord |
| S24.132A | Anterior cord syndrome at T2-T6 level of thoracic spinal cord, initial encounter |
| S24.132D | Anterior cord syndrome at T2-T6 level of thoracic spinal cord, subsequent encounter |
| S24.132S | Anterior cord syndrome at T2-T6 level of thoracic spinal cord, sequela |
| S24.133 | Anterior cord syndrome at T7-T10 level of thoracic spinal cord |
| S24.133A | Anterior cord syndrome at T7-T10 level of thoracic spinal cord, initial encounter |
| S24.133D | Anterior cord syndrome at T7-T10 level of thoracic spinal cord, subsequent encounter |
| S24.133S | Anterior cord syndrome at T7-T10 level of thoracic spinal cord, sequela |
| S24.134 | Anterior cord syndrome at T11-T12 level of thoracic spinal cord |
| S24.134A | Anterior cord syndrome at T11-T12 level of thoracic spinal cord, initial encounter |
| S24.134D | Anterior cord syndrome at T11-T12 level of thoracic spinal cord, subsequent encounter |
| S24.134S | Anterior cord syndrome at T11-T12 level of thoracic spinal cord, sequela |
| S24.139 | Anterior cord syndrome at unspecified level of thoracic spinal cord |
| S24.139A | Anterior cord syndrome at unspecified level of thoracic spinal cord, initial encounter |
| S24.139D | Anterior cord syndrome at unspecified level of thoracic spinal cord, subsequent encounter |
| S24.139S | Anterior cord syndrome at unspecified level of thoracic spinal cord, sequela |
| S24.14 | Brown-Sequard syndrome of thoracic spinal cord |
| S24.141 | Brown-Sequard syndrome at T1 level of thoracic spinal cord |
| S24.141A | Brown-Sequard syndrome at T1 level of thoracic spinal cord, initial encounter |
| S24.141D | Brown-Sequard syndrome at T1 level of thoracic spinal cord, subsequent encounter |
| S24.141S | Brown-Sequard syndrome at T1 level of thoracic spinal cord, sequela |
| S24.142 | Brown-Sequard syndrome at T2-T6 level of thoracic spinal cord |
| S24.142A | Brown-Sequard syndrome at T2-T6 level of thoracic spinal cord, initial encounter |
| S24.142D | Brown-Sequard syndrome at T2-T6 level of thoracic spinal cord, subsequent encounter |
| S24.142S | Brown-Sequard syndrome at T2-T6 level of thoracic spinal cord, sequela |
| S24.143 | Brown-Sequard syndrome at T7-T10 level of thoracic spinal cord |
| S24.143A | Brown-Sequard syndrome at T7-T10 level of thoracic spinal cord, initial encounter |
| S24.143D | Brown-Sequard syndrome at T7-T10 level of thoracic spinal cord, subsequent encounter |
| S24.143S | Brown-Sequard syndrome at T7-T10 level of thoracic spinal cord, sequela |
| S24.144 | Brown-Sequard syndrome at T11-T12 level of thoracic spinal cord |
| S24.144A | Brown-Sequard syndrome at T11-T12 level of thoracic spinal cord, initial encounter |
| S24.144D | Brown-Sequard syndrome at T11-T12 level of thoracic spinal cord, subsequent encounter |
| S24.144S | Brown-Sequard syndrome at T11-T12 level of thoracic spinal cord, sequela |
| S24.149 | Brown-Sequard syndrome at unspecified level of thoracic spinal cord |
| S24.149A | Brown-Sequard syndrome at unspecified level of thoracic spinal cord, initial encounter |
| S24.149D | Brown-Sequard syndrome at unspecified level of thoracic spinal cord, subsequent encounter |
| S24.149S | Brown-Sequard syndrome at unspecified level of thoracic spinal cord, sequela |
| S24.15 | Other incomplete lesions of thoracic spinal cord |
| S24.151 | Other incomplete lesion at T1 level of thoracic spinal cord |
| S24.151A | Other incomplete lesion at T1 level of thoracic spinal cord, initial encounter |
| S24.151D | Other incomplete lesion at T1 level of thoracic spinal cord, subsequent encounter |
| S24.151S | Other incomplete lesion at T1 level of thoracic spinal cord, sequela |
| S24.152 | Other incomplete lesion at T2-T6 level of thoracic spinal cord |
| S24.152A | Other incomplete lesion at T2-T6 level of thoracic spinal cord, initial encounter |
| S24.152D | Other incomplete lesion at T2-T6 level of thoracic spinal cord, subsequent encounter |
| S24.152S | Other incomplete lesion at T2-T6 level of thoracic spinal cord, sequela |
| S24.153 | Other incomplete lesion at T7-T10 level of thoracic spinal cord |
| S24.153A | Other incomplete lesion at T7-T10 level of thoracic spinal cord, initial encounter |
| S24.153D | Other incomplete lesion at T7-T10 level of thoracic spinal cord, subsequent encounter |
| S24.153S | Other incomplete lesion at T7-T10 level of thoracic spinal cord, sequela |
| S24.154 | Other incomplete lesion at T11-T12 level of thoracic spinal cord |
| S24.154A | Other incomplete lesion at T11-T12 level of thoracic spinal cord, initial encounter |
| S24.154D | Other incomplete lesion at T11-T12 level of thoracic spinal cord, subsequent encounter |
| S24.154S | Other incomplete lesion at T11-T12 level of thoracic spinal cord, sequela |
| S24.159 | Other incomplete lesion at unspecified level of thoracic spinal cord |
| S24.159A | Other incomplete lesion at unspecified level of thoracic spinal cord, initial encounter |
| S24.159D | Other incomplete lesion at unspecified level of thoracic spinal cord, subsequent encounter |
| S24.159S | Other incomplete lesion at unspecified level of thoracic spinal cord, sequela |
| S24.2 | Injury of nerve root of thoracic spine |
| S24.2XXA | Injury of nerve root of thoracic spine, initial encounter |
| S24.2XXD | Injury of nerve root of thoracic spine, subsequent encounter |
| S24.2XXS | Injury of nerve root of thoracic spine, sequela |
| S24.3 | Injury of peripheral nerves of thorax |
| S24.3XXA | Injury of peripheral nerves of thorax, initial encounter |
| S24.3XXD | Injury of peripheral nerves of thorax, subsequent encounter |
| S24.3XXS | Injury of peripheral nerves of thorax, sequela |
| S24.4 | Injury of thoracic sympathetic nervous system |
| S24.4XXA | Injury of thoracic sympathetic nervous system, initial encounter |
| S24.4XXD | Injury of thoracic sympathetic nervous system, subsequent encounter |
| S24.4XXS | Injury of thoracic sympathetic nervous system, sequela |
| S24.8 | Injury of other specified nerves of thorax |
| S24.8XXA | Injury of other specified nerves of thorax, initial encounter |
| S24.8XXD | Injury of other specified nerves of thorax, subsequent encounter |
| S24.8XXS | Injury of other specified nerves of thorax, sequela |
| S24.9 | Injury of unspecified nerve of thorax |
| S24.9XXA | Injury of unspecified nerve of thorax, initial encounter |
| S24.9XXD | Injury of unspecified nerve of thorax, subsequent encounter |
| S24.9XXS | Injury of unspecified nerve of thorax, sequela |
| S28 | Crushing injury of thorax, and traumatic amputation of part of thorax |
| S28.0 | Crushed chest |
| S28.0XXA | Crushed chest, initial encounter |
| S28.0XXD | Crushed chest, subsequent encounter |
| S28.0XXS | Crushed chest, sequela |
| S28.1 | Traumatic amputation (partial) of part of thorax, except breast |
| S28.1XXA | Traumatic amputation (partial) of part of thorax, except breast, initial encounter |
| S28.1XXD | Traumatic amputation (partial) of part of thorax, except breast, subsequent encounter |
| S28.1XXS | Traumatic amputation (partial) of part of thorax, except breast, sequela |
| S28.2 | Traumatic amputation of breast |
| S28.21 | Complete traumatic amputation of breast |
| S28.211 | Complete traumatic amputation of right breast |
| S28.211A | Complete traumatic amputation of right breast, initial encounter |
| S28.211D | Complete traumatic amputation of right breast, subsequent encounter |
| S28.211S | Complete traumatic amputation of right breast, sequela |
| S28.212 | Complete traumatic amputation of left breast |
| S28.212A | Complete traumatic amputation of left breast, initial encounter |
| S28.212D | Complete traumatic amputation of left breast, subsequent encounter |
| S28.212S | Complete traumatic amputation of left breast, sequela |
| S28.219 | Complete traumatic amputation of unspecified breast |
| S28.219A | Complete traumatic amputation of unspecified breast, initial encounter |
| S28.219D | Complete traumatic amputation of unspecified breast, subsequent encounter |
| S28.219S | Complete traumatic amputation of unspecified breast, sequela |
| S28.22 | Partial traumatic amputation of breast |
| S28.221 | Partial traumatic amputation of right breast |
| S28.221A | Partial traumatic amputation of right breast, initial encounter |
| S28.221D | Partial traumatic amputation of right breast, subsequent encounter |
| S28.221S | Partial traumatic amputation of right breast, sequela |
| S28.222 | Partial traumatic amputation of left breast |
| S28.222A | Partial traumatic amputation of left breast, initial encounter |
| S28.222D | Partial traumatic amputation of left breast, subsequent encounter |
| S28.222S | Partial traumatic amputation of left breast, sequela |
| S28.229 | Partial traumatic amputation of unspecified breast |
| S28.229A | Partial traumatic amputation of unspecified breast, initial encounter |
| S28.229D | Partial traumatic amputation of unspecified breast, subsequent encounter |
| S28.229S | Partial traumatic amputation of unspecified breast, sequela |
| S31 | Open wound of abdomen, lower back, pelvis and external genitals |
| S31.0 | Open wound of lower back and pelvis |
| S31.00 | Unspecified open wound of lower back and pelvis |
| S31.000 | Unspecified open wound of lower back and pelvis without penetration into retroperitoneum |
| S31.000A | Unspecified open wound of lower back and pelvis without penetration into retroperitoneum, initial encounter |
| S31.000D | Unspecified open wound of lower back and pelvis without penetration into retroperitoneum, subsequent encounter |
| S31.000S | Unspecified open wound of lower back and pelvis without penetration into retroperitoneum, sequela |
| S31.001 | Unspecified open wound of lower back and pelvis with penetration into retroperitoneum |
| S31.001A | Unspecified open wound of lower back and pelvis with penetration into retroperitoneum, initial encounter |
| S31.001D | Unspecified open wound of lower back and pelvis with penetration into retroperitoneum, subsequent encounter |
| S31.001S | Unspecified open wound of lower back and pelvis with penetration into retroperitoneum, sequela |
| S31.01 | Laceration without foreign body of lower back and pelvis |
| S31.010 | Laceration without foreign body of lower back and pelvis without penetration into retroperitoneum |
| S31.010A | Laceration without foreign body of lower back and pelvis without penetration into retroperitoneum, initial encounter |
| S31.010D | Laceration without foreign body of lower back and pelvis without penetration into retroperitoneum, subsequent encounter |
| S31.010S | Laceration without foreign body of lower back and pelvis without penetration into retroperitoneum, sequela |
| S31.011 | Laceration without foreign body of lower back and pelvis with penetration into retroperitoneum |
| S31.011A | Laceration without foreign body of lower back and pelvis with penetration into retroperitoneum, initial encounter |
| S31.011D | Laceration without foreign body of lower back and pelvis with penetration into retroperitoneum, subsequent encounter |
| S31.011S | Laceration without foreign body of lower back and pelvis with penetration into retroperitoneum, sequela |
| S31.02 | Laceration with foreign body of lower back and pelvis |
| S31.020 | Laceration with foreign body of lower back and pelvis without penetration into retroperitoneum |
| S31.020A | Laceration with foreign body of lower back and pelvis without penetration into retroperitoneum, initial encounter |
| S31.020D | Laceration with foreign body of lower back and pelvis without penetration into retroperitoneum, subsequent encounter |
| S31.020S | Laceration with foreign body of lower back and pelvis without penetration into retroperitoneum, sequela |
| S31.021 | Laceration with foreign body of lower back and pelvis with penetration into retroperitoneum |
| S31.021A | Laceration with foreign body of lower back and pelvis with penetration into retroperitoneum, initial encounter |
| S31.021D | Laceration with foreign body of lower back and pelvis with penetration into retroperitoneum, subsequent encounter |
| S31.021S | Laceration with foreign body of lower back and pelvis with penetration into retroperitoneum, sequela |
| S31.03 | Puncture wound without foreign body of lower back and pelvis |
| S31.030 | Puncture wound without foreign body of lower back and pelvis without penetration into retroperitoneum |
| S31.030A | Puncture wound without foreign body of lower back and pelvis without penetration into retroperitoneum, initial encounter |
| S31.030D | Puncture wound without foreign body of lower back and pelvis without penetration into retroperitoneum, subsequent encounter |
| S31.030S | Puncture wound without foreign body of lower back and pelvis without penetration into retroperitoneum, sequela |
| S31.031 | Puncture wound without foreign body of lower back and pelvis with penetration into retroperitoneum |
| S31.031A | Puncture wound without foreign body of lower back and pelvis with penetration into retroperitoneum, initial encounter |
| S31.031D | Puncture wound without foreign body of lower back and pelvis with penetration into retroperitoneum, subsequent encounter |
| S31.031S | Puncture wound without foreign body of lower back and pelvis with penetration into retroperitoneum, sequela |
| S31.04 | Puncture wound with foreign body of lower back and pelvis |
| S31.040 | Puncture wound with foreign body of lower back and pelvis without penetration into retroperitoneum |
| S31.040A | Puncture wound with foreign body of lower back and pelvis without penetration into retroperitoneum, initial encounter |
| S31.040D | Puncture wound with foreign body of lower back and pelvis without penetration into retroperitoneum, subsequent encounter |
| S31.040S | Puncture wound with foreign body of lower back and pelvis without penetration into retroperitoneum, sequela |
| S31.041 | Puncture wound with foreign body of lower back and pelvis with penetration into retroperitoneum |
| S31.041A | Puncture wound with foreign body of lower back and pelvis with penetration into retroperitoneum, initial encounter |
| S31.041D | Puncture wound with foreign body of lower back and pelvis with penetration into retroperitoneum, subsequent encounter |
| S31.041S | Puncture wound with foreign body of lower back and pelvis with penetration into retroperitoneum, sequela |
| S31.05 | Open bite of lower back and pelvis |
| S31.050 | Open bite of lower back and pelvis without penetration into retroperitoneum |
| S31.050A | Open bite of lower back and pelvis without penetration into retroperitoneum, initial encounter |
| S31.050D | Open bite of lower back and pelvis without penetration into retroperitoneum, subsequent encounter |
| S31.050S | Open bite of lower back and pelvis without penetration into retroperitoneum, sequela |
| S31.051 | Open bite of lower back and pelvis with penetration into retroperitoneum |
| S31.051A | Open bite of lower back and pelvis with penetration into retroperitoneum, initial encounter |
| S31.051D | Open bite of lower back and pelvis with penetration into retroperitoneum, subsequent encounter |
| S31.051S | Open bite of lower back and pelvis with penetration into retroperitoneum, sequela |
| S31.1 | Open wound of abdominal wall without penetration into peritoneal cavity |
| S31.10 | Unspecified open wound of abdominal wall without penetration into peritoneal cavity |
| S31.100 | Unspecified open wound of abdominal wall, right upper quadrant without penetration into peritoneal cavity |
| S31.100A | Unspecified open wound of abdominal wall, right upper quadrant without penetration into peritoneal cavity, initial encounter |
| S31.100D | Unspecified open wound of abdominal wall, right upper quadrant without penetration into peritoneal cavity, subsequent encounter |
| S31.100S | Unspecified open wound of abdominal wall, right upper quadrant without penetration into peritoneal cavity, sequela |
| S31.101 | Unspecified open wound of abdominal wall, left upper quadrant without penetration into peritoneal cavity |
| S31.101A | Unspecified open wound of abdominal wall, left upper quadrant without penetration into peritoneal cavity, initial encounter |
| S31.101D | Unspecified open wound of abdominal wall, left upper quadrant without penetration into peritoneal cavity, subsequent encounter |
| S31.101S | Unspecified open wound of abdominal wall, left upper quadrant without penetration into peritoneal cavity, sequela |
| S31.102 | Unspecified open wound of abdominal wall, epigastric region without penetration into peritoneal cavity |
| S31.102A | Unspecified open wound of abdominal wall, epigastric region without penetration into peritoneal cavity, initial encounter |
| S31.102D | Unspecified open wound of abdominal wall, epigastric region without penetration into peritoneal cavity, subsequent encounter |
| S31.102S | Unspecified open wound of abdominal wall, epigastric region without penetration into peritoneal cavity, sequela |
| S31.103 | Unspecified open wound of abdominal wall, right lower quadrant without penetration into peritoneal cavity |
| S31.103A | Unspecified open wound of abdominal wall, right lower quadrant without penetration into peritoneal cavity, initial encounter |
| S31.103D | Unspecified open wound of abdominal wall, right lower quadrant without penetration into peritoneal cavity, subsequent encounter |
| S31.103S | Unspecified open wound of abdominal wall, right lower quadrant without penetration into peritoneal cavity, sequela |
| S31.104 | Unspecified open wound of abdominal wall, left lower quadrant without penetration into peritoneal cavity |
| S31.104A | Unspecified open wound of abdominal wall, left lower quadrant without penetration into peritoneal cavity, initial encounter |
| S31.104D | Unspecified open wound of abdominal wall, left lower quadrant without penetration into peritoneal cavity, subsequent encounter |
| S31.104S | Unspecified open wound of abdominal wall, left lower quadrant without penetration into peritoneal cavity, sequela |
| S31.105 | Unspecified open wound of abdominal wall, periumbilic region without penetration into peritoneal cavity |
| S31.105A | Unspecified open wound of abdominal wall, periumbilic region without penetration into peritoneal cavity, initial encounter |
| S31.105D | Unspecified open wound of abdominal wall, periumbilic region without penetration into peritoneal cavity, subsequent encounter |
| S31.105S | Unspecified open wound of abdominal wall, periumbilic region without penetration into peritoneal cavity, sequela |
| S31.109 | Unspecified open wound of abdominal wall, unspecified quadrant without penetration into peritoneal cavity |
| S31.109A | Unspecified open wound of abdominal wall, unspecified quadrant without penetration into peritoneal cavity, initial encounter |
| S31.109D | Unspecified open wound of abdominal wall, unspecified quadrant without penetration into peritoneal cavity, subsequent encounter |
| S31.109S | Unspecified open wound of abdominal wall, unspecified quadrant without penetration into peritoneal cavity, sequela |
| S31.11 | Laceration without foreign body of abdominal wall without penetration into peritoneal cavity |
| S31.110 | Laceration without foreign body of abdominal wall, right upper quadrant without penetration into peritoneal cavity |
| S31.110A | Laceration without foreign body of abdominal wall, right upper quadrant without penetration into peritoneal cavity, initial encounter |
| S31.110D | Laceration without foreign body of abdominal wall, right upper quadrant without penetration into peritoneal cavity, subsequent encounter |
| S31.110S | Laceration without foreign body of abdominal wall, right upper quadrant without penetration into peritoneal cavity, sequela |
| S31.111 | Laceration without foreign body of abdominal wall, left upper quadrant without penetration into peritoneal cavity |
| S31.111A | Laceration without foreign body of abdominal wall, left upper quadrant without penetration into peritoneal cavity, initial encounter |
| S31.111D | Laceration without foreign body of abdominal wall, left upper quadrant without penetration into peritoneal cavity, subsequent encounter |
| S31.111S | Laceration without foreign body of abdominal wall, left upper quadrant without penetration into peritoneal cavity, sequela |
| S31.112 | Laceration without foreign body of abdominal wall, epigastric region without penetration into peritoneal cavity |
| S31.112A | Laceration without foreign body of abdominal wall, epigastric region without penetration into peritoneal cavity, initial encounter |
| S31.112D | Laceration without foreign body of abdominal wall, epigastric region without penetration into peritoneal cavity, subsequent encounter |
| S31.112S | Laceration without foreign body of abdominal wall, epigastric region without penetration into peritoneal cavity, sequela |
| S31.113 | Laceration without foreign body of abdominal wall, right lower quadrant without penetration into peritoneal cavity |
| S31.113A | Laceration without foreign body of abdominal wall, right lower quadrant without penetration into peritoneal cavity, initial encounter |
| S31.113D | Laceration without foreign body of abdominal wall, right lower quadrant without penetration into peritoneal cavity, subsequent encounter |
| S31.113S | Laceration without foreign body of abdominal wall, right lower quadrant without penetration into peritoneal cavity, sequela |
| S31.114 | Laceration without foreign body of abdominal wall, left lower quadrant without penetration into peritoneal cavity |
| S31.114A | Laceration without foreign body of abdominal wall, left lower quadrant without penetration into peritoneal cavity, initial encounter |
| S31.114D | Laceration without foreign body of abdominal wall, left lower quadrant without penetration into peritoneal cavity, subsequent encounter |
| S31.114S | Laceration without foreign body of abdominal wall, left lower quadrant without penetration into peritoneal cavity, sequela |
| S31.115 | Laceration without foreign body of abdominal wall, periumbilic region without penetration into peritoneal cavity |
| S31.115A | Laceration without foreign body of abdominal wall, periumbilic region without penetration into peritoneal cavity, initial encounter |
| S31.115D | Laceration without foreign body of abdominal wall, periumbilic region without penetration into peritoneal cavity, subsequent encounter |
| S31.115S | Laceration without foreign body of abdominal wall, periumbilic region without penetration into peritoneal cavity, sequela |
| S31.119 | Laceration without foreign body of abdominal wall, unspecified quadrant without penetration into peritoneal cavity |
| S31.119A | Laceration without foreign body of abdominal wall, unspecified quadrant without penetration into peritoneal cavity, initial encounter |
| S31.119D | Laceration without foreign body of abdominal wall, unspecified quadrant without penetration into peritoneal cavity, subsequent encounter |
| S31.119S | Laceration without foreign body of abdominal wall, unspecified quadrant without penetration into peritoneal cavity, sequela |
| S31.12 | Laceration with foreign body of abdominal wall without penetration into peritoneal cavity |
| S31.120 | Laceration of abdominal wall with foreign body, right upper quadrant without penetration into peritoneal cavity |
| S31.120A | Laceration of abdominal wall with foreign body, right upper quadrant without penetration into peritoneal cavity, initial encounter |
| S31.120D | Laceration of abdominal wall with foreign body, right upper quadrant without penetration into peritoneal cavity, subsequent encounter |
| S31.120S | Laceration of abdominal wall with foreign body, right upper quadrant without penetration into peritoneal cavity, sequela |
| S31.121 | Laceration of abdominal wall with foreign body, left upper quadrant without penetration into peritoneal cavity |
| S31.121A | Laceration of abdominal wall with foreign body, left upper quadrant without penetration into peritoneal cavity, initial encounter |
| S31.121D | Laceration of abdominal wall with foreign body, left upper quadrant without penetration into peritoneal cavity, subsequent encounter |
| S31.121S | Laceration of abdominal wall with foreign body, left upper quadrant without penetration into peritoneal cavity, sequela |
| S31.122 | Laceration of abdominal wall with foreign body, epigastric region without penetration into peritoneal cavity |
| S31.122A | Laceration of abdominal wall with foreign body, epigastric region without penetration into peritoneal cavity, initial encounter |
| S31.122D | Laceration of abdominal wall with foreign body, epigastric region without penetration into peritoneal cavity, subsequent encounter |
| S31.122S | Laceration of abdominal wall with foreign body, epigastric region without penetration into peritoneal cavity, sequela |
| S31.123 | Laceration of abdominal wall with foreign body, right lower quadrant without penetration into peritoneal cavity |
| S31.123A | Laceration of abdominal wall with foreign body, right lower quadrant without penetration into peritoneal cavity, initial encounter |
| S31.123D | Laceration of abdominal wall with foreign body, right lower quadrant without penetration into peritoneal cavity, subsequent encounter |
| S31.123S | Laceration of abdominal wall with foreign body, right lower quadrant without penetration into peritoneal cavity, sequela |
| S31.124 | Laceration of abdominal wall with foreign body, left lower quadrant without penetration into peritoneal cavity |
| S31.124A | Laceration of abdominal wall with foreign body, left lower quadrant without penetration into peritoneal cavity, initial encounter |
| S31.124D | Laceration of abdominal wall with foreign body, left lower quadrant without penetration into peritoneal cavity, subsequent encounter |
| S31.124S | Laceration of abdominal wall with foreign body, left lower quadrant without penetration into peritoneal cavity, sequela |
| S31.125 | Laceration of abdominal wall with foreign body, periumbilic region without penetration into peritoneal cavity |
| S31.125A | Laceration of abdominal wall with foreign body, periumbilic region without penetration into peritoneal cavity, initial encounter |
| S31.125D | Laceration of abdominal wall with foreign body, periumbilic region without penetration into peritoneal cavity, subsequent encounter |
| S31.125S | Laceration of abdominal wall with foreign body, periumbilic region without penetration into peritoneal cavity, sequela |
| S31.129 | Laceration of abdominal wall with foreign body, unspecified quadrant without penetration into peritoneal cavity |
| S31.129A | Laceration of abdominal wall with foreign body, unspecified quadrant without penetration into peritoneal cavity, initial encounter |
| S31.129D | Laceration of abdominal wall with foreign body, unspecified quadrant without penetration into peritoneal cavity, subsequent encounter |
| S31.129S | Laceration of abdominal wall with foreign body, unspecified quadrant without penetration into peritoneal cavity, sequela |
| S31.13 | Puncture wound of abdominal wall without foreign body without penetration into peritoneal cavity |
| S31.130 | Puncture wound of abdominal wall without foreign body, right upper quadrant without penetration into peritoneal cavity |
| S31.130A | Puncture wound of abdominal wall without foreign body, right upper quadrant without penetration into peritoneal cavity, initial encounter |
| S31.130D | Puncture wound of abdominal wall without foreign body, right upper quadrant without penetration into peritoneal cavity, subsequent encounter |
| S31.130S | Puncture wound of abdominal wall without foreign body, right upper quadrant without penetration into peritoneal cavity, sequela |
| S31.131 | Puncture wound of abdominal wall without foreign body, left upper quadrant without penetration into peritoneal cavity |
| S31.131A | Puncture wound of abdominal wall without foreign body, left upper quadrant without penetration into peritoneal cavity, initial encounter |
| S31.131D | Puncture wound of abdominal wall without foreign body, left upper quadrant without penetration into peritoneal cavity, subsequent encounter |
| S31.131S | Puncture wound of abdominal wall without foreign body, left upper quadrant without penetration into peritoneal cavity, sequela |
| S31.132 | Puncture wound of abdominal wall without foreign body, epigastric region without penetration into peritoneal cavity |
| S31.132A | Puncture wound of abdominal wall without foreign body, epigastric region without penetration into peritoneal cavity, initial encounter |
| S31.132D | Puncture wound of abdominal wall without foreign body, epigastric region without penetration into peritoneal cavity, subsequent encounter |
| S31.132S | Puncture wound of abdominal wall without foreign body, epigastric region without penetration into peritoneal cavity, sequela |
| S31.133 | Puncture wound of abdominal wall without foreign body, right lower quadrant without penetration into peritoneal cavity |
| S31.133A | Puncture wound of abdominal wall without foreign body, right lower quadrant without penetration into peritoneal cavity, initial encounter |
| S31.133D | Puncture wound of abdominal wall without foreign body, right lower quadrant without penetration into peritoneal cavity, subsequent encounter |
| S31.133S | Puncture wound of abdominal wall without foreign body, right lower quadrant without penetration into peritoneal cavity, sequela |
| S31.134 | Puncture wound of abdominal wall without foreign body, left lower quadrant without penetration into peritoneal cavity |
| S31.134A | Puncture wound of abdominal wall without foreign body, left lower quadrant without penetration into peritoneal cavity, initial encounter |
| S31.134D | Puncture wound of abdominal wall without foreign body, left lower quadrant without penetration into peritoneal cavity, subsequent encounter |
| S31.134S | Puncture wound of abdominal wall without foreign body, left lower quadrant without penetration into peritoneal cavity, sequela |
| S31.135 | Puncture wound of abdominal wall without foreign body, periumbilic region without penetration into peritoneal cavity |
| S31.135A | Puncture wound of abdominal wall without foreign body, periumbilic region without penetration into peritoneal cavity, initial encounter |
| S31.135D | Puncture wound of abdominal wall without foreign body, periumbilic region without penetration into peritoneal cavity, subsequent encounter |
| S31.135S | Puncture wound of abdominal wall without foreign body, periumbilic region without penetration into peritoneal cavity, sequela |
| S31.139 | Puncture wound of abdominal wall without foreign body, unspecified quadrant without penetration into peritoneal cavity |
| S31.139A | Puncture wound of abdominal wall without foreign body, unspecified quadrant without penetration into peritoneal cavity, initial encounter |
| S31.139D | Puncture wound of abdominal wall without foreign body, unspecified quadrant without penetration into peritoneal cavity, subsequent encounter |
| S31.139S | Puncture wound of abdominal wall without foreign body, unspecified quadrant without penetration into peritoneal cavity, sequela |
| S31.14 | Puncture wound of abdominal wall with foreign body without penetration into peritoneal cavity |
| S31.140 | Puncture wound of abdominal wall with foreign body, right upper quadrant without penetration into peritoneal cavity |
| S31.140A | Puncture wound of abdominal wall with foreign body, right upper quadrant without penetration into peritoneal cavity, initial encounter |
| S31.140D | Puncture wound of abdominal wall with foreign body, right upper quadrant without penetration into peritoneal cavity, subsequent encounter |
| S31.140S | Puncture wound of abdominal wall with foreign body, right upper quadrant without penetration into peritoneal cavity, sequela |
| S31.141 | Puncture wound of abdominal wall with foreign body, left upper quadrant without penetration into peritoneal cavity |
| S31.141A | Puncture wound of abdominal wall with foreign body, left upper quadrant without penetration into peritoneal cavity, initial encounter |
| S31.141D | Puncture wound of abdominal wall with foreign body, left upper quadrant without penetration into peritoneal cavity, subsequent encounter |
| S31.141S | Puncture wound of abdominal wall with foreign body, left upper quadrant without penetration into peritoneal cavity, sequela |
| S31.142 | Puncture wound of abdominal wall with foreign body, epigastric region without penetration into peritoneal cavity |
| S31.142A | Puncture wound of abdominal wall with foreign body, epigastric region without penetration into peritoneal cavity, initial encounter |
| S31.142D | Puncture wound of abdominal wall with foreign body, epigastric region without penetration into peritoneal cavity, subsequent encounter |
| S31.142S | Puncture wound of abdominal wall with foreign body, epigastric region without penetration into peritoneal cavity, sequela |
| S31.143 | Puncture wound of abdominal wall with foreign body, right lower quadrant without penetration into peritoneal cavity |
| S31.143A | Puncture wound of abdominal wall with foreign body, right lower quadrant without penetration into peritoneal cavity, initial encounter |
| S31.143D | Puncture wound of abdominal wall with foreign body, right lower quadrant without penetration into peritoneal cavity, subsequent encounter |
| S31.143S | Puncture wound of abdominal wall with foreign body, right lower quadrant without penetration into peritoneal cavity, sequela |
| S31.144 | Puncture wound of abdominal wall with foreign body, left lower quadrant without penetration into peritoneal cavity |
| S31.144A | Puncture wound of abdominal wall with foreign body, left lower quadrant without penetration into peritoneal cavity, initial encounter |
| S31.144D | Puncture wound of abdominal wall with foreign body, left lower quadrant without penetration into peritoneal cavity, subsequent encounter |
| S31.144S | Puncture wound of abdominal wall with foreign body, left lower quadrant without penetration into peritoneal cavity, sequela |
| S31.145 | Puncture wound of abdominal wall with foreign body, periumbilic region without penetration into peritoneal cavity |
| S31.145A | Puncture wound of abdominal wall with foreign body, periumbilic region without penetration into peritoneal cavity, initial encounter |
| S31.145D | Puncture wound of abdominal wall with foreign body, periumbilic region without penetration into peritoneal cavity, subsequent encounter |
| S31.145S | Puncture wound of abdominal wall with foreign body, periumbilic region without penetration into peritoneal cavity, sequela |
| S31.149 | Puncture wound of abdominal wall with foreign body, unspecified quadrant without penetration into peritoneal cavity |
| S31.149A | Puncture wound of abdominal wall with foreign body, unspecified quadrant without penetration into peritoneal cavity, initial encounter |
| S31.149D | Puncture wound of abdominal wall with foreign body, unspecified quadrant without penetration into peritoneal cavity, subsequent encounter |
| S31.149S | Puncture wound of abdominal wall with foreign body, unspecified quadrant without penetration into peritoneal cavity, sequela |
| S31.15 | Open bite of abdominal wall without penetration into peritoneal cavity |
| S31.150 | Open bite of abdominal wall, right upper quadrant without penetration into peritoneal cavity |
| S31.150A | Open bite of abdominal wall, right upper quadrant without penetration into peritoneal cavity, initial encounter |
| S31.150D | Open bite of abdominal wall, right upper quadrant without penetration into peritoneal cavity, subsequent encounter |
| S31.150S | Open bite of abdominal wall, right upper quadrant without penetration into peritoneal cavity, sequela |
| S31.151 | Open bite of abdominal wall, left upper quadrant without penetration into peritoneal cavity |
| S31.151A | Open bite of abdominal wall, left upper quadrant without penetration into peritoneal cavity, initial encounter |
| S31.151D | Open bite of abdominal wall, left upper quadrant without penetration into peritoneal cavity, subsequent encounter |
| S31.151S | Open bite of abdominal wall, left upper quadrant without penetration into peritoneal cavity, sequela |
| S31.152 | Open bite of abdominal wall, epigastric region without penetration into peritoneal cavity |
| S31.152A | Open bite of abdominal wall, epigastric region without penetration into peritoneal cavity, initial encounter |
| S31.152D | Open bite of abdominal wall, epigastric region without penetration into peritoneal cavity, subsequent encounter |
| S31.152S | Open bite of abdominal wall, epigastric region without penetration into peritoneal cavity, sequela |
| S31.153 | Open bite of abdominal wall, right lower quadrant without penetration into peritoneal cavity |
| S31.153A | Open bite of abdominal wall, right lower quadrant without penetration into peritoneal cavity, initial encounter |
| S31.153D | Open bite of abdominal wall, right lower quadrant without penetration into peritoneal cavity, subsequent encounter |
| S31.153S | Open bite of abdominal wall, right lower quadrant without penetration into peritoneal cavity, sequela |
| S31.154 | Open bite of abdominal wall, left lower quadrant without penetration into peritoneal cavity |
| S31.154A | Open bite of abdominal wall, left lower quadrant without penetration into peritoneal cavity, initial encounter |
| S31.154D | Open bite of abdominal wall, left lower quadrant without penetration into peritoneal cavity, subsequent encounter |
| S31.154S | Open bite of abdominal wall, left lower quadrant without penetration into peritoneal cavity, sequela |
| S31.155 | Open bite of abdominal wall, periumbilic region without penetration into peritoneal cavity |
| S31.155A | Open bite of abdominal wall, periumbilic region without penetration into peritoneal cavity, initial encounter |
| S31.155D | Open bite of abdominal wall, periumbilic region without penetration into peritoneal cavity, subsequent encounter |
| S31.155S | Open bite of abdominal wall, periumbilic region without penetration into peritoneal cavity, sequela |
| S31.159 | Open bite of abdominal wall, unspecified quadrant without penetration into peritoneal cavity |
| S31.159A | Open bite of abdominal wall, unspecified quadrant without penetration into peritoneal cavity, initial encounter |
| S31.159D | Open bite of abdominal wall, unspecified quadrant without penetration into peritoneal cavity, subsequent encounter |
| S31.159S | Open bite of abdominal wall, unspecified quadrant without penetration into peritoneal cavity, sequela |
| S31.2 | Open wound of penis |
| S31.20 | Unspecified open wound of penis |
| S31.20XA | Unspecified open wound of penis, initial encounter |
| S31.20XD | Unspecified open wound of penis, subsequent encounter |
| S31.20XS | Unspecified open wound of penis, sequela |
| S31.21 | Laceration without foreign body of penis |
| S31.21XA | Laceration without foreign body of penis, initial encounter |
| S31.21XD | Laceration without foreign body of penis, subsequent encounter |
| S31.21XS | Laceration without foreign body of penis, sequela |
| S31.22 | Laceration with foreign body of penis |
| S31.22XA | Laceration with foreign body of penis, initial encounter |
| S31.22XD | Laceration with foreign body of penis, subsequent encounter |
| S31.22XS | Laceration with foreign body of penis, sequela |
| S31.23 | Puncture wound without foreign body of penis |
| S31.23XA | Puncture wound without foreign body of penis, initial encounter |
| S31.23XD | Puncture wound without foreign body of penis, subsequent encounter |
| S31.23XS | Puncture wound without foreign body of penis, sequela |
| S31.24 | Puncture wound with foreign body of penis |
| S31.24XA | Puncture wound with foreign body of penis, initial encounter |
| S31.24XD | Puncture wound with foreign body of penis, subsequent encounter |
| S31.24XS | Puncture wound with foreign body of penis, sequela |
| S31.25 | Open bite of penis |
| S31.25XA | Open bite of penis, initial encounter |
| S31.25XD | Open bite of penis, subsequent encounter |
| S31.25XS | Open bite of penis, sequela |
| S31.3 | Open wound of scrotum and testes |
| S31.30 | Unspecified open wound of scrotum and testes |
| S31.30XA | Unspecified open wound of scrotum and testes, initial encounter |
| S31.30XD | Unspecified open wound of scrotum and testes, subsequent encounter |
| S31.30XS | Unspecified open wound of scrotum and testes, sequela |
| S31.31 | Laceration without foreign body of scrotum and testes |
| S31.31XA | Laceration without foreign body of scrotum and testes, initial encounter |
| S31.31XD | Laceration without foreign body of scrotum and testes, subsequent encounter |
| S31.31XS | Laceration without foreign body of scrotum and testes, sequela |
| S31.32 | Laceration with foreign body of scrotum and testes |
| S31.32XA | Laceration with foreign body of scrotum and testes, initial encounter |
| S31.32XD | Laceration with foreign body of scrotum and testes, subsequent encounter |
| S31.32XS | Laceration with foreign body of scrotum and testes, sequela |
| S31.33 | Puncture wound without foreign body of scrotum and testes |
| S31.33XA | Puncture wound without foreign body of scrotum and testes, initial encounter |
| S31.33XD | Puncture wound without foreign body of scrotum and testes, subsequent encounter |
| S31.33XS | Puncture wound without foreign body of scrotum and testes, sequela |
| S31.34 | Puncture wound with foreign body of scrotum and testes |
| S31.34XA | Puncture wound with foreign body of scrotum and testes, initial encounter |
| S31.34XD | Puncture wound with foreign body of scrotum and testes, subsequent encounter |
| S31.34XS | Puncture wound with foreign body of scrotum and testes, sequela |
| S31.35 | Open bite of scrotum and testes |
| S31.35XA | Open bite of scrotum and testes, initial encounter |
| S31.35XD | Open bite of scrotum and testes, subsequent encounter |
| S31.35XS | Open bite of scrotum and testes, sequela |
| S31.4 | Open wound of vagina and vulva |
| S31.40 | Unspecified open wound of vagina and vulva |
| S31.40XA | Unspecified open wound of vagina and vulva, initial encounter |
| S31.40XD | Unspecified open wound of vagina and vulva, subsequent encounter |
| S31.40XS | Unspecified open wound of vagina and vulva, sequela |
| S31.41 | Laceration without foreign body of vagina and vulva |
| S31.41XA | Laceration without foreign body of vagina and vulva, initial encounter |
| S31.41XD | Laceration without foreign body of vagina and vulva, subsequent encounter |
| S31.41XS | Laceration without foreign body of vagina and vulva, sequela |
| S31.42 | Laceration with foreign body of vagina and vulva |
| S31.42XA | Laceration with foreign body of vagina and vulva, initial encounter |
| S31.42XD | Laceration with foreign body of vagina and vulva, subsequent encounter |
| S31.42XS | Laceration with foreign body of vagina and vulva, sequela |
| S31.43 | Puncture wound without foreign body of vagina and vulva |
| S31.43XA | Puncture wound without foreign body of vagina and vulva, initial encounter |
| S31.43XD | Puncture wound without foreign body of vagina and vulva, subsequent encounter |
| S31.43XS | Puncture wound without foreign body of vagina and vulva, sequela |
| S31.44 | Puncture wound with foreign body of vagina and vulva |
| S31.44XA | Puncture wound with foreign body of vagina and vulva, initial encounter |
| S31.44XD | Puncture wound with foreign body of vagina and vulva, subsequent encounter |
| S31.44XS | Puncture wound with foreign body of vagina and vulva, sequela |
| S31.45 | Open bite of vagina and vulva |
| S31.45XA | Open bite of vagina and vulva, initial encounter |
| S31.45XD | Open bite of vagina and vulva, subsequent encounter |
| S31.45XS | Open bite of vagina and vulva, sequela |
| S31.5 | Open wound of unspecified external genital organs |
| S31.50 | Unspecified open wound of unspecified external genital organs |
| S31.501 | Unspecified open wound of unspecified external genital organs, male |
| S31.501A | Unspecified open wound of unspecified external genital organs, male, initial encounter |
| S31.501D | Unspecified open wound of unspecified external genital organs, male, subsequent encounter |
| S31.501S | Unspecified open wound of unspecified external genital organs, male, sequela |
| S31.502 | Unspecified open wound of unspecified external genital organs, female |
| S31.502A | Unspecified open wound of unspecified external genital organs, female, initial encounter |
| S31.502D | Unspecified open wound of unspecified external genital organs, female, subsequent encounter |
| S31.502S | Unspecified open wound of unspecified external genital organs, female, sequela |
| S31.51 | Laceration without foreign body of unspecified external genital organs |
| S31.511 | Laceration without foreign body of unspecified external genital organs, male |
| S31.511A | Laceration without foreign body of unspecified external genital organs, male, initial encounter |
| S31.511D | Laceration without foreign body of unspecified external genital organs, male, subsequent encounter |
| S31.511S | Laceration without foreign body of unspecified external genital organs, male, sequela |
| S31.512 | Laceration without foreign body of unspecified external genital organs, female |
| S31.512A | Laceration without foreign body of unspecified external genital organs, female, initial encounter |
| S31.512D | Laceration without foreign body of unspecified external genital organs, female, subsequent encounter |
| S31.512S | Laceration without foreign body of unspecified external genital organs, female, sequela |
| S31.52 | Laceration with foreign body of unspecified external genital organs |
| S31.521 | Laceration with foreign body of unspecified external genital organs, male |
| S31.521A | Laceration with foreign body of unspecified external genital organs, male, initial encounter |
| S31.521D | Laceration with foreign body of unspecified external genital organs, male, subsequent encounter |
| S31.521S | Laceration with foreign body of unspecified external genital organs, male, sequela |
| S31.522 | Laceration with foreign body of unspecified external genital organs, female |
| S31.522A | Laceration with foreign body of unspecified external genital organs, female, initial encounter |
| S31.522D | Laceration with foreign body of unspecified external genital organs, female, subsequent encounter |
| S31.522S | Laceration with foreign body of unspecified external genital organs, female, sequela |
| S31.53 | Puncture wound without foreign body of unspecified external genital organs |
| S31.531 | Puncture wound without foreign body of unspecified external genital organs, male |
| S31.531A | Puncture wound without foreign body of unspecified external genital organs, male, initial encounter |
| S31.531D | Puncture wound without foreign body of unspecified external genital organs, male, subsequent encounter |
| S31.531S | Puncture wound without foreign body of unspecified external genital organs, male, sequela |
| S31.532 | Puncture wound without foreign body of unspecified external genital organs, female |
| S31.532A | Puncture wound without foreign body of unspecified external genital organs, female, initial encounter |
| S31.532D | Puncture wound without foreign body of unspecified external genital organs, female, subsequent encounter |
| S31.532S | Puncture wound without foreign body of unspecified external genital organs, female, sequela |
| S31.54 | Puncture wound with foreign body of unspecified external genital organs |
| S31.541 | Puncture wound with foreign body of unspecified external genital organs, male |
| S31.541A | Puncture wound with foreign body of unspecified external genital organs, male, initial encounter |
| S31.541D | Puncture wound with foreign body of unspecified external genital organs, male, subsequent encounter |
| S31.541S | Puncture wound with foreign body of unspecified external genital organs, male, sequela |
| S31.542 | Puncture wound with foreign body of unspecified external genital organs, female |
| S31.542A | Puncture wound with foreign body of unspecified external genital organs, female, initial encounter |
| S31.542D | Puncture wound with foreign body of unspecified external genital organs, female, subsequent encounter |
| S31.542S | Puncture wound with foreign body of unspecified external genital organs, female, sequela |
| S31.55 | Open bite of unspecified external genital organs |
| S31.551 | Open bite of unspecified external genital organs, male |
| S31.551A | Open bite of unspecified external genital organs, male, initial encounter |
| S31.551D | Open bite of unspecified external genital organs, male, subsequent encounter |
| S31.551S | Open bite of unspecified external genital organs, male, sequela |
| S31.552 | Open bite of unspecified external genital organs, female |
| S31.552A | Open bite of unspecified external genital organs, female, initial encounter |
| S31.552D | Open bite of unspecified external genital organs, female, subsequent encounter |
| S31.552S | Open bite of unspecified external genital organs, female, sequela |
| S31.6 | Open wound of abdominal wall with penetration into peritoneal cavity |
| S31.60 | Unspecified open wound of abdominal wall with penetration into peritoneal cavity |
| S31.600 | Unspecified open wound of abdominal wall, right upper quadrant with penetration into peritoneal cavity |
| S31.600A | Unspecified open wound of abdominal wall, right upper quadrant with penetration into peritoneal cavity, initial encounter |
| S31.600D | Unspecified open wound of abdominal wall, right upper quadrant with penetration into peritoneal cavity, subsequent encounter |
| S31.600S | Unspecified open wound of abdominal wall, right upper quadrant with penetration into peritoneal cavity, sequela |
| S31.601 | Unspecified open wound of abdominal wall, left upper quadrant with penetration into peritoneal cavity |
| S31.601A | Unspecified open wound of abdominal wall, left upper quadrant with penetration into peritoneal cavity, initial encounter |
| S31.601D | Unspecified open wound of abdominal wall, left upper quadrant with penetration into peritoneal cavity, subsequent encounter |
| S31.601S | Unspecified open wound of abdominal wall, left upper quadrant with penetration into peritoneal cavity, sequela |
| S31.602 | Unspecified open wound of abdominal wall, epigastric region with penetration into peritoneal cavity |
| S31.602A | Unspecified open wound of abdominal wall, epigastric region with penetration into peritoneal cavity, initial encounter |
| S31.602D | Unspecified open wound of abdominal wall, epigastric region with penetration into peritoneal cavity, subsequent encounter |
| S31.602S | Unspecified open wound of abdominal wall, epigastric region with penetration into peritoneal cavity, sequela |
| S31.603 | Unspecified open wound of abdominal wall, right lower quadrant with penetration into peritoneal cavity |
| S31.603A | Unspecified open wound of abdominal wall, right lower quadrant with penetration into peritoneal cavity, initial encounter |
| S31.603D | Unspecified open wound of abdominal wall, right lower quadrant with penetration into peritoneal cavity, subsequent encounter |
| S31.603S | Unspecified open wound of abdominal wall, right lower quadrant with penetration into peritoneal cavity, sequela |
| S31.604 | Unspecified open wound of abdominal wall, left lower quadrant with penetration into peritoneal cavity |
| S31.604A | Unspecified open wound of abdominal wall, left lower quadrant with penetration into peritoneal cavity, initial encounter |
| S31.604D | Unspecified open wound of abdominal wall, left lower quadrant with penetration into peritoneal cavity, subsequent encounter |
| S31.604S | Unspecified open wound of abdominal wall, left lower quadrant with penetration into peritoneal cavity, sequela |
| S31.605 | Unspecified open wound of abdominal wall, periumbilic region with penetration into peritoneal cavity |
| S31.605A | Unspecified open wound of abdominal wall, periumbilic region with penetration into peritoneal cavity, initial encounter |
| S31.605D | Unspecified open wound of abdominal wall, periumbilic region with penetration into peritoneal cavity, subsequent encounter |
| S31.605S | Unspecified open wound of abdominal wall, periumbilic region with penetration into peritoneal cavity, sequela |
| S31.609 | Unspecified open wound of abdominal wall, unspecified quadrant with penetration into peritoneal cavity |
| S31.609A | Unspecified open wound of abdominal wall, unspecified quadrant with penetration into peritoneal cavity, initial encounter |
| S31.609D | Unspecified open wound of abdominal wall, unspecified quadrant with penetration into peritoneal cavity, subsequent encounter |
| S31.609S | Unspecified open wound of abdominal wall, unspecified quadrant with penetration into peritoneal cavity, sequela |
| S31.61 | Laceration without foreign body of abdominal wall with penetration into peritoneal cavity |
| S31.610 | Laceration without foreign body of abdominal wall, right upper quadrant with penetration into peritoneal cavity |
| S31.610A | Laceration without foreign body of abdominal wall, right upper quadrant with penetration into peritoneal cavity, initial encounter |
| S31.610D | Laceration without foreign body of abdominal wall, right upper quadrant with penetration into peritoneal cavity, subsequent encounter |
| S31.610S | Laceration without foreign body of abdominal wall, right upper quadrant with penetration into peritoneal cavity, sequela |
| S31.611 | Laceration without foreign body of abdominal wall, left upper quadrant with penetration into peritoneal cavity |
| S31.611A | Laceration without foreign body of abdominal wall, left upper quadrant with penetration into peritoneal cavity, initial encounter |
| S31.611D | Laceration without foreign body of abdominal wall, left upper quadrant with penetration into peritoneal cavity, subsequent encounter |
| S31.611S | Laceration without foreign body of abdominal wall, left upper quadrant with penetration into peritoneal cavity, sequela |
| S31.612 | Laceration without foreign body of abdominal wall, epigastric region with penetration into peritoneal cavity |
| S31.612A | Laceration without foreign body of abdominal wall, epigastric region with penetration into peritoneal cavity, initial encounter |
| S31.612D | Laceration without foreign body of abdominal wall, epigastric region with penetration into peritoneal cavity, subsequent encounter |
| S31.612S | Laceration without foreign body of abdominal wall, epigastric region with penetration into peritoneal cavity, sequela |
| S31.613 | Laceration without foreign body of abdominal wall, right lower quadrant with penetration into peritoneal cavity |
| S31.613A | Laceration without foreign body of abdominal wall, right lower quadrant with penetration into peritoneal cavity, initial encounter |
| S31.613D | Laceration without foreign body of abdominal wall, right lower quadrant with penetration into peritoneal cavity, subsequent encounter |
| S31.613S | Laceration without foreign body of abdominal wall, right lower quadrant with penetration into peritoneal cavity, sequela |
| S31.614 | Laceration without foreign body of abdominal wall, left lower quadrant with penetration into peritoneal cavity |
| S31.614A | Laceration without foreign body of abdominal wall, left lower quadrant with penetration into peritoneal cavity, initial encounter |
| S31.614D | Laceration without foreign body of abdominal wall, left lower quadrant with penetration into peritoneal cavity, subsequent encounter |
| S31.614S | Laceration without foreign body of abdominal wall, left lower quadrant with penetration into peritoneal cavity, sequela |
| S31.615 | Laceration without foreign body of abdominal wall, periumbilic region with penetration into peritoneal cavity |
| S31.615A | Laceration without foreign body of abdominal wall, periumbilic region with penetration into peritoneal cavity, initial encounter |
| S31.615D | Laceration without foreign body of abdominal wall, periumbilic region with penetration into peritoneal cavity, subsequent encounter |
| S31.615S | Laceration without foreign body of abdominal wall, periumbilic region with penetration into peritoneal cavity, sequela |
| S31.619 | Laceration without foreign body of abdominal wall, unspecified quadrant with penetration into peritoneal cavity |
| S31.619A | Laceration without foreign body of abdominal wall, unspecified quadrant with penetration into peritoneal cavity, initial encounter |
| S31.619D | Laceration without foreign body of abdominal wall, unspecified quadrant with penetration into peritoneal cavity, subsequent encounter |
| S31.619S | Laceration without foreign body of abdominal wall, unspecified quadrant with penetration into peritoneal cavity, sequela |
| S31.62 | Laceration with foreign body of abdominal wall with penetration into peritoneal cavity |
| S31.620 | Laceration with foreign body of abdominal wall, right upper quadrant with penetration into peritoneal cavity |
| S31.620A | Laceration with foreign body of abdominal wall, right upper quadrant with penetration into peritoneal cavity, initial encounter |
| S31.620D | Laceration with foreign body of abdominal wall, right upper quadrant with penetration into peritoneal cavity, subsequent encounter |
| S31.620S | Laceration with foreign body of abdominal wall, right upper quadrant with penetration into peritoneal cavity, sequela |
| S31.621 | Laceration with foreign body of abdominal wall, left upper quadrant with penetration into peritoneal cavity |
| S31.621A | Laceration with foreign body of abdominal wall, left upper quadrant with penetration into peritoneal cavity, initial encounter |
| S31.621D | Laceration with foreign body of abdominal wall, left upper quadrant with penetration into peritoneal cavity, subsequent encounter |
| S31.621S | Laceration with foreign body of abdominal wall, left upper quadrant with penetration into peritoneal cavity, sequela |
| S31.622 | Laceration with foreign body of abdominal wall, epigastric region with penetration into peritoneal cavity |
| S31.622A | Laceration with foreign body of abdominal wall, epigastric region with penetration into peritoneal cavity, initial encounter |
| S31.622D | Laceration with foreign body of abdominal wall, epigastric region with penetration into peritoneal cavity, subsequent encounter |
| S31.622S | Laceration with foreign body of abdominal wall, epigastric region with penetration into peritoneal cavity, sequela |
| S31.623 | Laceration with foreign body of abdominal wall, right lower quadrant with penetration into peritoneal cavity |
| S31.623A | Laceration with foreign body of abdominal wall, right lower quadrant with penetration into peritoneal cavity, initial encounter |
| S31.623D | Laceration with foreign body of abdominal wall, right lower quadrant with penetration into peritoneal cavity, subsequent encounter |
| S31.623S | Laceration with foreign body of abdominal wall, right lower quadrant with penetration into peritoneal cavity, sequela |
| S31.624 | Laceration with foreign body of abdominal wall, left lower quadrant with penetration into peritoneal cavity |
| S31.624A | Laceration with foreign body of abdominal wall, left lower quadrant with penetration into peritoneal cavity, initial encounter |
| S31.624D | Laceration with foreign body of abdominal wall, left lower quadrant with penetration into peritoneal cavity, subsequent encounter |
| S31.624S | Laceration with foreign body of abdominal wall, left lower quadrant with penetration into peritoneal cavity, sequela |
| S31.625 | Laceration with foreign body of abdominal wall, periumbilic region with penetration into peritoneal cavity |
| S31.625A | Laceration with foreign body of abdominal wall, periumbilic region with penetration into peritoneal cavity, initial encounter |
| S31.625D | Laceration with foreign body of abdominal wall, periumbilic region with penetration into peritoneal cavity, subsequent encounter |
| S31.625S | Laceration with foreign body of abdominal wall, periumbilic region with penetration into peritoneal cavity, sequela |
| S31.629 | Laceration with foreign body of abdominal wall, unspecified quadrant with penetration into peritoneal cavity |
| S31.629A | Laceration with foreign body of abdominal wall, unspecified quadrant with penetration into peritoneal cavity, initial encounter |
| S31.629D | Laceration with foreign body of abdominal wall, unspecified quadrant with penetration into peritoneal cavity, subsequent encounter |
| S31.629S | Laceration with foreign body of abdominal wall, unspecified quadrant with penetration into peritoneal cavity, sequela |
| S31.63 | Puncture wound without foreign body of abdominal wall with penetration into peritoneal cavity |
| S31.630 | Puncture wound without foreign body of abdominal wall, right upper quadrant with penetration into peritoneal cavity |
| S31.630A | Puncture wound without foreign body of abdominal wall, right upper quadrant with penetration into peritoneal cavity, initial encounter |
| S31.630D | Puncture wound without foreign body of abdominal wall, right upper quadrant with penetration into peritoneal cavity, subsequent encounter |
| S31.630S | Puncture wound without foreign body of abdominal wall, right upper quadrant with penetration into peritoneal cavity, sequela |
| S31.631 | Puncture wound without foreign body of abdominal wall, left upper quadrant with penetration into peritoneal cavity |
| S31.631A | Puncture wound without foreign body of abdominal wall, left upper quadrant with penetration into peritoneal cavity, initial encounter |
| S31.631D | Puncture wound without foreign body of abdominal wall, left upper quadrant with penetration into peritoneal cavity, subsequent encounter |
| S31.631S | Puncture wound without foreign body of abdominal wall, left upper quadrant with penetration into peritoneal cavity, sequela |
| S31.632 | Puncture wound without foreign body of abdominal wall, epigastric region with penetration into peritoneal cavity |
| S31.632A | Puncture wound without foreign body of abdominal wall, epigastric region with penetration into peritoneal cavity, initial encounter |
| S31.632D | Puncture wound without foreign body of abdominal wall, epigastric region with penetration into peritoneal cavity, subsequent encounter |
| S31.632S | Puncture wound without foreign body of abdominal wall, epigastric region with penetration into peritoneal cavity, sequela |
| S31.633 | Puncture wound without foreign body of abdominal wall, right lower quadrant with penetration into peritoneal cavity |
| S31.633A | Puncture wound without foreign body of abdominal wall, right lower quadrant with penetration into peritoneal cavity, initial encounter |
| S31.633D | Puncture wound without foreign body of abdominal wall, right lower quadrant with penetration into peritoneal cavity, subsequent encounter |
| S31.633S | Puncture wound without foreign body of abdominal wall, right lower quadrant with penetration into peritoneal cavity, sequela |
| S31.634 | Puncture wound without foreign body of abdominal wall, left lower quadrant with penetration into peritoneal cavity |
| S31.634A | Puncture wound without foreign body of abdominal wall, left lower quadrant with penetration into peritoneal cavity, initial encounter |
| S31.634D | Puncture wound without foreign body of abdominal wall, left lower quadrant with penetration into peritoneal cavity, subsequent encounter |
| S31.634S | Puncture wound without foreign body of abdominal wall, left lower quadrant with penetration into peritoneal cavity, sequela |
| S31.635 | Puncture wound without foreign body of abdominal wall, periumbilic region with penetration into peritoneal cavity |
| S31.635A | Puncture wound without foreign body of abdominal wall, periumbilic region with penetration into peritoneal cavity, initial encounter |
| S31.635D | Puncture wound without foreign body of abdominal wall, periumbilic region with penetration into peritoneal cavity, subsequent encounter |
| S31.635S | Puncture wound without foreign body of abdominal wall, periumbilic region with penetration into peritoneal cavity, sequela |
| S31.639 | Puncture wound without foreign body of abdominal wall, unspecified quadrant with penetration into peritoneal cavity |
| S31.639A | Puncture wound without foreign body of abdominal wall, unspecified quadrant with penetration into peritoneal cavity, initial encounter |
| S31.639D | Puncture wound without foreign body of abdominal wall, unspecified quadrant with penetration into peritoneal cavity, subsequent encounter |
| S31.639S | Puncture wound without foreign body of abdominal wall, unspecified quadrant with penetration into peritoneal cavity, sequela |
| S31.64 | Puncture wound with foreign body of abdominal wall with penetration into peritoneal cavity |
| S31.640 | Puncture wound with foreign body of abdominal wall, right upper quadrant with penetration into peritoneal cavity |
| S31.640A | Puncture wound with foreign body of abdominal wall, right upper quadrant with penetration into peritoneal cavity, initial encounter |
| S31.640D | Puncture wound with foreign body of abdominal wall, right upper quadrant with penetration into peritoneal cavity, subsequent encounter |
| S31.640S | Puncture wound with foreign body of abdominal wall, right upper quadrant with penetration into peritoneal cavity, sequela |
| S31.641 | Puncture wound with foreign body of abdominal wall, left upper quadrant with penetration into peritoneal cavity |
| S31.641A | Puncture wound with foreign body of abdominal wall, left upper quadrant with penetration into peritoneal cavity, initial encounter |
| S31.641D | Puncture wound with foreign body of abdominal wall, left upper quadrant with penetration into peritoneal cavity, subsequent encounter |
| S31.641S | Puncture wound with foreign body of abdominal wall, left upper quadrant with penetration into peritoneal cavity, sequela |
| S31.642 | Puncture wound with foreign body of abdominal wall, epigastric region with penetration into peritoneal cavity |
| S31.642A | Puncture wound with foreign body of abdominal wall, epigastric region with penetration into peritoneal cavity, initial encounter |
| S31.642D | Puncture wound with foreign body of abdominal wall, epigastric region with penetration into peritoneal cavity, subsequent encounter |
| S31.642S | Puncture wound with foreign body of abdominal wall, epigastric region with penetration into peritoneal cavity, sequela |
| S31.643 | Puncture wound with foreign body of abdominal wall, right lower quadrant with penetration into peritoneal cavity |
| S31.643A | Puncture wound with foreign body of abdominal wall, right lower quadrant with penetration into peritoneal cavity, initial encounter |
| S31.643D | Puncture wound with foreign body of abdominal wall, right lower quadrant with penetration into peritoneal cavity, subsequent encounter |
| S31.643S | Puncture wound with foreign body of abdominal wall, right lower quadrant with penetration into peritoneal cavity, sequela |
| S31.644 | Puncture wound with foreign body of abdominal wall, left lower quadrant with penetration into peritoneal cavity |
| S31.644A | Puncture wound with foreign body of abdominal wall, left lower quadrant with penetration into peritoneal cavity, initial encounter |
| S31.644D | Puncture wound with foreign body of abdominal wall, left lower quadrant with penetration into peritoneal cavity, subsequent encounter |
| S31.644S | Puncture wound with foreign body of abdominal wall, left lower quadrant with penetration into peritoneal cavity, sequela |
| S31.645 | Puncture wound with foreign body of abdominal wall, periumbilic region with penetration into peritoneal cavity |
| S31.645A | Puncture wound with foreign body of abdominal wall, periumbilic region with penetration into peritoneal cavity, initial encounter |
| S31.645D | Puncture wound with foreign body of abdominal wall, periumbilic region with penetration into peritoneal cavity, subsequent encounter |
| S31.645S | Puncture wound with foreign body of abdominal wall, periumbilic region with penetration into peritoneal cavity, sequela |
| S31.649 | Puncture wound with foreign body of abdominal wall, unspecified quadrant with penetration into peritoneal cavity |
| S31.649A | Puncture wound with foreign body of abdominal wall, unspecified quadrant with penetration into peritoneal cavity, initial encounter |
| S31.649D | Puncture wound with foreign body of abdominal wall, unspecified quadrant with penetration into peritoneal cavity, subsequent encounter |
| S31.649S | Puncture wound with foreign body of abdominal wall, unspecified quadrant with penetration into peritoneal cavity, sequela |
| S31.65 | Open bite of abdominal wall with penetration into peritoneal cavity |
| S31.650 | Open bite of abdominal wall, right upper quadrant with penetration into peritoneal cavity |
| S31.650A | Open bite of abdominal wall, right upper quadrant with penetration into peritoneal cavity, initial encounter |
| S31.650D | Open bite of abdominal wall, right upper quadrant with penetration into peritoneal cavity, subsequent encounter |
| S31.650S | Open bite of abdominal wall, right upper quadrant with penetration into peritoneal cavity, sequela |
| S31.651 | Open bite of abdominal wall, left upper quadrant with penetration into peritoneal cavity |
| S31.651A | Open bite of abdominal wall, left upper quadrant with penetration into peritoneal cavity, initial encounter |
| S31.651D | Open bite of abdominal wall, left upper quadrant with penetration into peritoneal cavity, subsequent encounter |
| S31.651S | Open bite of abdominal wall, left upper quadrant with penetration into peritoneal cavity, sequela |
| S31.652 | Open bite of abdominal wall, epigastric region with penetration into peritoneal cavity |
| S31.652A | Open bite of abdominal wall, epigastric region with penetration into peritoneal cavity, initial encounter |
| S31.652D | Open bite of abdominal wall, epigastric region with penetration into peritoneal cavity, subsequent encounter |
| S31.652S | Open bite of abdominal wall, epigastric region with penetration into peritoneal cavity, sequela |
| S31.653 | Open bite of abdominal wall, right lower quadrant with penetration into peritoneal cavity |
| S31.653A | Open bite of abdominal wall, right lower quadrant with penetration into peritoneal cavity, initial encounter |
| S31.653D | Open bite of abdominal wall, right lower quadrant with penetration into peritoneal cavity, subsequent encounter |
| S31.653S | Open bite of abdominal wall, right lower quadrant with penetration into peritoneal cavity, sequela |
| S31.654 | Open bite of abdominal wall, left lower quadrant with penetration into peritoneal cavity |
| S31.654A | Open bite of abdominal wall, left lower quadrant with penetration into peritoneal cavity, initial encounter |
| S31.654D | Open bite of abdominal wall, left lower quadrant with penetration into peritoneal cavity, subsequent encounter |
| S31.654S | Open bite of abdominal wall, left lower quadrant with penetration into peritoneal cavity, sequela |
| S31.655 | Open bite of abdominal wall, periumbilic region with penetration into peritoneal cavity |
| S31.655A | Open bite of abdominal wall, periumbilic region with penetration into peritoneal cavity, initial encounter |
| S31.655D | Open bite of abdominal wall, periumbilic region with penetration into peritoneal cavity, subsequent encounter |
| S31.655S | Open bite of abdominal wall, periumbilic region with penetration into peritoneal cavity, sequela |
| S31.659 | Open bite of abdominal wall, unspecified quadrant with penetration into peritoneal cavity |
| S31.659A | Open bite of abdominal wall, unspecified quadrant with penetration into peritoneal cavity, initial encounter |
| S31.659D | Open bite of abdominal wall, unspecified quadrant with penetration into peritoneal cavity, subsequent encounter |
| S31.659S | Open bite of abdominal wall, unspecified quadrant with penetration into peritoneal cavity, sequela |
| S31.8 | Open wound of other parts of abdomen, lower back and pelvis |
| S31.80 | Open wound of unspecified buttock |
| S31.801 | Laceration without foreign body of unspecified buttock |
| S31.801A | Laceration without foreign body of unspecified buttock, initial encounter |
| S31.801D | Laceration without foreign body of unspecified buttock, subsequent encounter |
| S31.801S | Laceration without foreign body of unspecified buttock, sequela |
| S31.802 | Laceration with foreign body of unspecified buttock |
| S31.802A | Laceration with foreign body of unspecified buttock, initial encounter |
| S31.802D | Laceration with foreign body of unspecified buttock, subsequent encounter |
| S31.802S | Laceration with foreign body of unspecified buttock, sequela |
| S31.803 | Puncture wound without foreign body of unspecified buttock |
| S31.803A | Puncture wound without foreign body of unspecified buttock, initial encounter |
| S31.803D | Puncture wound without foreign body of unspecified buttock, subsequent encounter |
| S31.803S | Puncture wound without foreign body of unspecified buttock, sequela |
| S31.804 | Puncture wound with foreign body of unspecified buttock |
| S31.804A | Puncture wound with foreign body of unspecified buttock, initial encounter |
| S31.804D | Puncture wound with foreign body of unspecified buttock, subsequent encounter |
| S31.804S | Puncture wound with foreign body of unspecified buttock, sequela |
| S31.805 | Open bite of unspecified buttock |
| S31.805A | Open bite of unspecified buttock, initial encounter |
| S31.805D | Open bite of unspecified buttock, subsequent encounter |
| S31.805S | Open bite of unspecified buttock, sequela |
| S31.809 | Unspecified open wound of unspecified buttock |
| S31.809A | Unspecified open wound of unspecified buttock, initial encounter |
| S31.809D | Unspecified open wound of unspecified buttock, subsequent encounter |
| S31.809S | Unspecified open wound of unspecified buttock, sequela |
| S31.81 | Open wound of right buttock |
| S31.811 | Laceration without foreign body of right buttock |
| S31.811A | Laceration without foreign body of right buttock, initial encounter |
| S31.811D | Laceration without foreign body of right buttock, subsequent encounter |
| S31.811S | Laceration without foreign body of right buttock, sequela |
| S31.812 | Laceration with foreign body of right buttock |
| S31.812A | Laceration with foreign body of right buttock, initial encounter |
| S31.812D | Laceration with foreign body of right buttock, subsequent encounter |
| S31.812S | Laceration with foreign body of right buttock, sequela |
| S31.813 | Puncture wound without foreign body of right buttock |
| S31.813A | Puncture wound without foreign body of right buttock, initial encounter |
| S31.813D | Puncture wound without foreign body of right buttock, subsequent encounter |
| S31.813S | Puncture wound without foreign body of right buttock, sequela |
| S31.814 | Puncture wound with foreign body of right buttock |
| S31.814A | Puncture wound with foreign body of right buttock, initial encounter |
| S31.814D | Puncture wound with foreign body of right buttock, subsequent encounter |
| S31.814S | Puncture wound with foreign body of right buttock, sequela |
| S31.815 | Open bite of right buttock |
| S31.815A | Open bite of right buttock, initial encounter |
| S31.815D | Open bite of right buttock, subsequent encounter |
| S31.815S | Open bite of right buttock, sequela |
| S31.819 | Unspecified open wound of right buttock |
| S31.819A | Unspecified open wound of right buttock, initial encounter |
| S31.819D | Unspecified open wound of right buttock, subsequent encounter |
| S31.819S | Unspecified open wound of right buttock, sequela |
| S31.82 | Open wound of left buttock |
| S31.821 | Laceration without foreign body of left buttock |
| S31.821A | Laceration without foreign body of left buttock, initial encounter |
| S31.821D | Laceration without foreign body of left buttock, subsequent encounter |
| S31.821S | Laceration without foreign body of left buttock, sequela |
| S31.822 | Laceration with foreign body of left buttock |
| S31.822A | Laceration with foreign body of left buttock, initial encounter |
| S31.822D | Laceration with foreign body of left buttock, subsequent encounter |
| S31.822S | Laceration with foreign body of left buttock, sequela |
| S31.823 | Puncture wound without foreign body of left buttock |
| S31.823A | Puncture wound without foreign body of left buttock, initial encounter |
| S31.823D | Puncture wound without foreign body of left buttock, subsequent encounter |
| S31.823S | Puncture wound without foreign body of left buttock, sequela |
| S31.824 | Puncture wound with foreign body of left buttock |
| S31.824A | Puncture wound with foreign body of left buttock, initial encounter |
| S31.824D | Puncture wound with foreign body of left buttock, subsequent encounter |
| S31.824S | Puncture wound with foreign body of left buttock, sequela |
| S31.825 | Open bite of left buttock |
| S31.825A | Open bite of left buttock, initial encounter |
| S31.825D | Open bite of left buttock, subsequent encounter |
| S31.825S | Open bite of left buttock, sequela |
| S31.829 | Unspecified open wound of left buttock |
| S31.829A | Unspecified open wound of left buttock, initial encounter |
| S31.829D | Unspecified open wound of left buttock, subsequent encounter |
| S31.829S | Unspecified open wound of left buttock, sequela |
| S31.83 | Open wound of anus |
| S31.831 | Laceration without foreign body of anus |
| S31.831A | Laceration without foreign body of anus, initial encounter |
| S31.831D | Laceration without foreign body of anus, subsequent encounter |
| S31.831S | Laceration without foreign body of anus, sequela |
| S31.832 | Laceration with foreign body of anus |
| S31.832A | Laceration with foreign body of anus, initial encounter |
| S31.832D | Laceration with foreign body of anus, subsequent encounter |
| S31.832S | Laceration with foreign body of anus, sequela |
| S31.833 | Puncture wound without foreign body of anus |
| S31.833A | Puncture wound without foreign body of anus, initial encounter |
| S31.833D | Puncture wound without foreign body of anus, subsequent encounter |
| S31.833S | Puncture wound without foreign body of anus, sequela |
| S31.834 | Puncture wound with foreign body of anus |
| S31.834A | Puncture wound with foreign body of anus, initial encounter |
| S31.834D | Puncture wound with foreign body of anus, subsequent encounter |
| S31.834S | Puncture wound with foreign body of anus, sequela |
| S31.835 | Open bite of anus |
| S31.835A | Open bite of anus, initial encounter |
| S31.835D | Open bite of anus, subsequent encounter |
| S31.835S | Open bite of anus, sequela |
| S31.839 | Unspecified open wound of anus |
| S31.839A | Unspecified open wound of anus, initial encounter |
| S31.839D | Unspecified open wound of anus, subsequent encounter |
| S31.839S | Unspecified open wound of anus, sequela |
| S32 | Fracture of lumbar spine and pelvis |
| S32.0 | Fracture of lumbar vertebra |
| S32.00 | Fracture of unspecified lumbar vertebra |
| S32.000 | Wedge compression fracture of unspecified lumbar vertebra |
| S32.000A | Wedge compression fracture of unspecified lumbar vertebra, initial encounter for closed fracture |
| S32.000B | Wedge compression fracture of unspecified lumbar vertebra, initial encounter for open fracture |
| S32.000D | Wedge compression fracture of unspecified lumbar vertebra, subsequent encounter for fracture with routine healing |
| S32.000G | Wedge compression fracture of unspecified lumbar vertebra, subsequent encounter for fracture with delayed healing |
| S32.000K | Wedge compression fracture of unspecified lumbar vertebra, subsequent encounter for fracture with nonunion |
| S32.000S | Wedge compression fracture of unspecified lumbar vertebra, sequela |
| S32.001 | Stable burst fracture of unspecified lumbar vertebra |
| S32.001A | Stable burst fracture of unspecified lumbar vertebra, initial encounter for closed fracture |
| S32.001B | Stable burst fracture of unspecified lumbar vertebra, initial encounter for open fracture |
| S32.001D | Stable burst fracture of unspecified lumbar vertebra, subsequent encounter for fracture with routine healing |
| S32.001G | Stable burst fracture of unspecified lumbar vertebra, subsequent encounter for fracture with delayed healing |
| S32.001K | Stable burst fracture of unspecified lumbar vertebra, subsequent encounter for fracture with nonunion |
| S32.001S | Stable burst fracture of unspecified lumbar vertebra, sequela |
| S32.002 | Unstable burst fracture of unspecified lumbar vertebra |
| S32.002A | Unstable burst fracture of unspecified lumbar vertebra, initial encounter for closed fracture |
| S32.002B | Unstable burst fracture of unspecified lumbar vertebra, initial encounter for open fracture |
| S32.002D | Unstable burst fracture of unspecified lumbar vertebra, subsequent encounter for fracture with routine healing |
| S32.002G | Unstable burst fracture of unspecified lumbar vertebra, subsequent encounter for fracture with delayed healing |
| S32.002K | Unstable burst fracture of unspecified lumbar vertebra, subsequent encounter for fracture with nonunion |
| S32.002S | Unstable burst fracture of unspecified lumbar vertebra, sequela |
| S32.008 | Other fracture of unspecified lumbar vertebra |
| S32.008A | Other fracture of unspecified lumbar vertebra, initial encounter for closed fracture |
| S32.008B | Other fracture of unspecified lumbar vertebra, initial encounter for open fracture |
| S32.008D | Other fracture of unspecified lumbar vertebra, subsequent encounter for fracture with routine healing |
| S32.008G | Other fracture of unspecified lumbar vertebra, subsequent encounter for fracture with delayed healing |
| S32.008K | Other fracture of unspecified lumbar vertebra, subsequent encounter for fracture with nonunion |
| S32.008S | Other fracture of unspecified lumbar vertebra, sequela |
| S32.009 | Unspecified fracture of unspecified lumbar vertebra |
| S32.009A | Unspecified fracture of unspecified lumbar vertebra, initial encounter for closed fracture |
| S32.009B | Unspecified fracture of unspecified lumbar vertebra, initial encounter for open fracture |
| S32.009D | Unspecified fracture of unspecified lumbar vertebra, subsequent encounter for fracture with routine healing |
| S32.009G | Unspecified fracture of unspecified lumbar vertebra, subsequent encounter for fracture with delayed healing |
| S32.009K | Unspecified fracture of unspecified lumbar vertebra, subsequent encounter for fracture with nonunion |
| S32.009S | Unspecified fracture of unspecified lumbar vertebra, sequela |
| S32.01 | Fracture of first lumbar vertebra |
| S32.010 | Wedge compression fracture of first lumbar vertebra |
| S32.010A | Wedge compression fracture of first lumbar vertebra, initial encounter for closed fracture |
| S32.010B | Wedge compression fracture of first lumbar vertebra, initial encounter for open fracture |
| S32.010D | Wedge compression fracture of first lumbar vertebra, subsequent encounter for fracture with routine healing |
| S32.010G | Wedge compression fracture of first lumbar vertebra, subsequent encounter for fracture with delayed healing |
| S32.010K | Wedge compression fracture of first lumbar vertebra, subsequent encounter for fracture with nonunion |
| S32.010S | Wedge compression fracture of first lumbar vertebra, sequela |
| S32.011 | Stable burst fracture of first lumbar vertebra |
| S32.011A | Stable burst fracture of first lumbar vertebra, initial encounter for closed fracture |
| S32.011B | Stable burst fracture of first lumbar vertebra, initial encounter for open fracture |
| S32.011D | Stable burst fracture of first lumbar vertebra, subsequent encounter for fracture with routine healing |
| S32.011G | Stable burst fracture of first lumbar vertebra, subsequent encounter for fracture with delayed healing |
| S32.011K | Stable burst fracture of first lumbar vertebra, subsequent encounter for fracture with nonunion |
| S32.011S | Stable burst fracture of first lumbar vertebra, sequela |
| S32.012 | Unstable burst fracture of first lumbar vertebra |
| S32.012A | Unstable burst fracture of first lumbar vertebra, initial encounter for closed fracture |
| S32.012B | Unstable burst fracture of first lumbar vertebra, initial encounter for open fracture |
| S32.012D | Unstable burst fracture of first lumbar vertebra, subsequent encounter for fracture with routine healing |
| S32.012G | Unstable burst fracture of first lumbar vertebra, subsequent encounter for fracture with delayed healing |
| S32.012K | Unstable burst fracture of first lumbar vertebra, subsequent encounter for fracture with nonunion |
| S32.012S | Unstable burst fracture of first lumbar vertebra, sequela |
| S32.018 | Other fracture of first lumbar vertebra |
| S32.018A | Other fracture of first lumbar vertebra, initial encounter for closed fracture |
| S32.018B | Other fracture of first lumbar vertebra, initial encounter for open fracture |
| S32.018D | Other fracture of first lumbar vertebra, subsequent encounter for fracture with routine healing |
| S32.018G | Other fracture of first lumbar vertebra, subsequent encounter for fracture with delayed healing |
| S32.018K | Other fracture of first lumbar vertebra, subsequent encounter for fracture with nonunion |
| S32.018S | Other fracture of first lumbar vertebra, sequela |
| S32.019 | Unspecified fracture of first lumbar vertebra |
| S32.019A | Unspecified fracture of first lumbar vertebra, initial encounter for closed fracture |
| S32.019B | Unspecified fracture of first lumbar vertebra, initial encounter for open fracture |
| S32.019D | Unspecified fracture of first lumbar vertebra, subsequent encounter for fracture with routine healing |
| S32.019G | Unspecified fracture of first lumbar vertebra, subsequent encounter for fracture with delayed healing |
| S32.019K | Unspecified fracture of first lumbar vertebra, subsequent encounter for fracture with nonunion |
| S32.019S | Unspecified fracture of first lumbar vertebra, sequela |
| S32.02 | Fracture of second lumbar vertebra |
| S32.020 | Wedge compression fracture of second lumbar vertebra |
| S32.020A | Wedge compression fracture of second lumbar vertebra, initial encounter for closed fracture |
| S32.020B | Wedge compression fracture of second lumbar vertebra, initial encounter for open fracture |
| S32.020D | Wedge compression fracture of second lumbar vertebra, subsequent encounter for fracture with routine healing |
| S32.020G | Wedge compression fracture of second lumbar vertebra, subsequent encounter for fracture with delayed healing |
| S32.020K | Wedge compression fracture of second lumbar vertebra, subsequent encounter for fracture with nonunion |
| S32.020S | Wedge compression fracture of second lumbar vertebra, sequela |
| S32.021 | Stable burst fracture of second lumbar vertebra |
| S32.021A | Stable burst fracture of second lumbar vertebra, initial encounter for closed fracture |
| S32.021B | Stable burst fracture of second lumbar vertebra, initial encounter for open fracture |
| S32.021D | Stable burst fracture of second lumbar vertebra, subsequent encounter for fracture with routine healing |
| S32.021G | Stable burst fracture of second lumbar vertebra, subsequent encounter for fracture with delayed healing |
| S32.021K | Stable burst fracture of second lumbar vertebra, subsequent encounter for fracture with nonunion |
| S32.021S | Stable burst fracture of second lumbar vertebra, sequela |
| S32.022 | Unstable burst fracture of second lumbar vertebra |
| S32.022A | Unstable burst fracture of second lumbar vertebra, initial encounter for closed fracture |
| S32.022B | Unstable burst fracture of second lumbar vertebra, initial encounter for open fracture |
| S32.022D | Unstable burst fracture of second lumbar vertebra, subsequent encounter for fracture with routine healing |
| S32.022G | Unstable burst fracture of second lumbar vertebra, subsequent encounter for fracture with delayed healing |
| S32.022K | Unstable burst fracture of second lumbar vertebra, subsequent encounter for fracture with nonunion |
| S32.022S | Unstable burst fracture of second lumbar vertebra, sequela |
| S32.028 | Other fracture of second lumbar vertebra |
| S32.028A | Other fracture of second lumbar vertebra, initial encounter for closed fracture |
| S32.028B | Other fracture of second lumbar vertebra, initial encounter for open fracture |
| S32.028D | Other fracture of second lumbar vertebra, subsequent encounter for fracture with routine healing |
| S32.028G | Other fracture of second lumbar vertebra, subsequent encounter for fracture with delayed healing |
| S32.028K | Other fracture of second lumbar vertebra, subsequent encounter for fracture with nonunion |
| S32.028S | Other fracture of second lumbar vertebra, sequela |
| S32.029 | Unspecified fracture of second lumbar vertebra |
| S32.029A | Unspecified fracture of second lumbar vertebra, initial encounter for closed fracture |
| S32.029B | Unspecified fracture of second lumbar vertebra, initial encounter for open fracture |
| S32.029D | Unspecified fracture of second lumbar vertebra, subsequent encounter for fracture with routine healing |
| S32.029G | Unspecified fracture of second lumbar vertebra, subsequent encounter for fracture with delayed healing |
| S32.029K | Unspecified fracture of second lumbar vertebra, subsequent encounter for fracture with nonunion |
| S32.029S | Unspecified fracture of second lumbar vertebra, sequela |
| S32.03 | Fracture of third lumbar vertebra |
| S32.030 | Wedge compression fracture of third lumbar vertebra |
| S32.030A | Wedge compression fracture of third lumbar vertebra, initial encounter for closed fracture |
| S32.030B | Wedge compression fracture of third lumbar vertebra, initial encounter for open fracture |
| S32.030D | Wedge compression fracture of third lumbar vertebra, subsequent encounter for fracture with routine healing |
| S32.030G | Wedge compression fracture of third lumbar vertebra, subsequent encounter for fracture with delayed healing |
| S32.030K | Wedge compression fracture of third lumbar vertebra, subsequent encounter for fracture with nonunion |
| S32.030S | Wedge compression fracture of third lumbar vertebra, sequela |
| S32.031 | Stable burst fracture of third lumbar vertebra |
| S32.031A | Stable burst fracture of third lumbar vertebra, initial encounter for closed fracture |
| S32.031B | Stable burst fracture of third lumbar vertebra, initial encounter for open fracture |
| S32.031D | Stable burst fracture of third lumbar vertebra, subsequent encounter for fracture with routine healing |
| S32.031G | Stable burst fracture of third lumbar vertebra, subsequent encounter for fracture with delayed healing |
| S32.031K | Stable burst fracture of third lumbar vertebra, subsequent encounter for fracture with nonunion |
| S32.031S | Stable burst fracture of third lumbar vertebra, sequela |
| S32.032 | Unstable burst fracture of third lumbar vertebra |
| S32.032A | Unstable burst fracture of third lumbar vertebra, initial encounter for closed fracture |
| S32.032B | Unstable burst fracture of third lumbar vertebra, initial encounter for open fracture |
| S32.032D | Unstable burst fracture of third lumbar vertebra, subsequent encounter for fracture with routine healing |
| S32.032G | Unstable burst fracture of third lumbar vertebra, subsequent encounter for fracture with delayed healing |
| S32.032K | Unstable burst fracture of third lumbar vertebra, subsequent encounter for fracture with nonunion |
| S32.032S | Unstable burst fracture of third lumbar vertebra, sequela |
| S32.038 | Other fracture of third lumbar vertebra |
| S32.038A | Other fracture of third lumbar vertebra, initial encounter for closed fracture |
| S32.038B | Other fracture of third lumbar vertebra, initial encounter for open fracture |
| S32.038D | Other fracture of third lumbar vertebra, subsequent encounter for fracture with routine healing |
| S32.038G | Other fracture of third lumbar vertebra, subsequent encounter for fracture with delayed healing |
| S32.038K | Other fracture of third lumbar vertebra, subsequent encounter for fracture with nonunion |
| S32.038S | Other fracture of third lumbar vertebra, sequela |
| S32.039 | Unspecified fracture of third lumbar vertebra |
| S32.039A | Unspecified fracture of third lumbar vertebra, initial encounter for closed fracture |
| S32.039B | Unspecified fracture of third lumbar vertebra, initial encounter for open fracture |
| S32.039D | Unspecified fracture of third lumbar vertebra, subsequent encounter for fracture with routine healing |
| S32.039G | Unspecified fracture of third lumbar vertebra, subsequent encounter for fracture with delayed healing |
| S32.039K | Unspecified fracture of third lumbar vertebra, subsequent encounter for fracture with nonunion |
| S32.039S | Unspecified fracture of third lumbar vertebra, sequela |
| S32.04 | Fracture of fourth lumbar vertebra |
| S32.040 | Wedge compression fracture of fourth lumbar vertebra |
| S32.040A | Wedge compression fracture of fourth lumbar vertebra, initial encounter for closed fracture |
| S32.040B | Wedge compression fracture of fourth lumbar vertebra, initial encounter for open fracture |
| S32.040D | Wedge compression fracture of fourth lumbar vertebra, subsequent encounter for fracture with routine healing |
| S32.040G | Wedge compression fracture of fourth lumbar vertebra, subsequent encounter for fracture with delayed healing |
| S32.040K | Wedge compression fracture of fourth lumbar vertebra, subsequent encounter for fracture with nonunion |
| S32.040S | Wedge compression fracture of fourth lumbar vertebra, sequela |
| S32.041 | Stable burst fracture of fourth lumbar vertebra |
| S32.041A | Stable burst fracture of fourth lumbar vertebra, initial encounter for closed fracture |
| S32.041B | Stable burst fracture of fourth lumbar vertebra, initial encounter for open fracture |
| S32.041D | Stable burst fracture of fourth lumbar vertebra, subsequent encounter for fracture with routine healing |
| S32.041G | Stable burst fracture of fourth lumbar vertebra, subsequent encounter for fracture with delayed healing |
| S32.041K | Stable burst fracture of fourth lumbar vertebra, subsequent encounter for fracture with nonunion |
| S32.041S | Stable burst fracture of fourth lumbar vertebra, sequela |
| S32.042 | Unstable burst fracture of fourth lumbar vertebra |
| S32.042A | Unstable burst fracture of fourth lumbar vertebra, initial encounter for closed fracture |
| S32.042B | Unstable burst fracture of fourth lumbar vertebra, initial encounter for open fracture |
| S32.042D | Unstable burst fracture of fourth lumbar vertebra, subsequent encounter for fracture with routine healing |
| S32.042G | Unstable burst fracture of fourth lumbar vertebra, subsequent encounter for fracture with delayed healing |
| S32.042K | Unstable burst fracture of fourth lumbar vertebra, subsequent encounter for fracture with nonunion |
| S32.042S | Unstable burst fracture of fourth lumbar vertebra, sequela |
| S32.048 | Other fracture of fourth lumbar vertebra |
| S32.048A | Other fracture of fourth lumbar vertebra, initial encounter for closed fracture |
| S32.048B | Other fracture of fourth lumbar vertebra, initial encounter for open fracture |
| S32.048D | Other fracture of fourth lumbar vertebra, subsequent encounter for fracture with routine healing |
| S32.048G | Other fracture of fourth lumbar vertebra, subsequent encounter for fracture with delayed healing |
| S32.048K | Other fracture of fourth lumbar vertebra, subsequent encounter for fracture with nonunion |
| S32.048S | Other fracture of fourth lumbar vertebra, sequela |
| S32.049 | Unspecified fracture of fourth lumbar vertebra |
| S32.049A | Unspecified fracture of fourth lumbar vertebra, initial encounter for closed fracture |
| S32.049B | Unspecified fracture of fourth lumbar vertebra, initial encounter for open fracture |
| S32.049D | Unspecified fracture of fourth lumbar vertebra, subsequent encounter for fracture with routine healing |
| S32.049G | Unspecified fracture of fourth lumbar vertebra, subsequent encounter for fracture with delayed healing |
| S32.049K | Unspecified fracture of fourth lumbar vertebra, subsequent encounter for fracture with nonunion |
| S32.049S | Unspecified fracture of fourth lumbar vertebra, sequela |
| S32.05 | Fracture of fifth lumbar vertebra |
| S32.050 | Wedge compression fracture of fifth lumbar vertebra |
| S32.050A | Wedge compression fracture of fifth lumbar vertebra, initial encounter for closed fracture |
| S32.050B | Wedge compression fracture of fifth lumbar vertebra, initial encounter for open fracture |
| S32.050D | Wedge compression fracture of fifth lumbar vertebra, subsequent encounter for fracture with routine healing |
| S32.050G | Wedge compression fracture of fifth lumbar vertebra, subsequent encounter for fracture with delayed healing |
| S32.050K | Wedge compression fracture of fifth lumbar vertebra, subsequent encounter for fracture with nonunion |
| S32.050S | Wedge compression fracture of fifth lumbar vertebra, sequela |
| S32.051 | Stable burst fracture of fifth lumbar vertebra |
| S32.051A | Stable burst fracture of fifth lumbar vertebra, initial encounter for closed fracture |
| S32.051B | Stable burst fracture of fifth lumbar vertebra, initial encounter for open fracture |
| S32.051D | Stable burst fracture of fifth lumbar vertebra, subsequent encounter for fracture with routine healing |
| S32.051G | Stable burst fracture of fifth lumbar vertebra, subsequent encounter for fracture with delayed healing |
| S32.051K | Stable burst fracture of fifth lumbar vertebra, subsequent encounter for fracture with nonunion |
| S32.051S | Stable burst fracture of fifth lumbar vertebra, sequela |
| S32.052 | Unstable burst fracture of fifth lumbar vertebra |
| S32.052A | Unstable burst fracture of fifth lumbar vertebra, initial encounter for closed fracture |
| S32.052B | Unstable burst fracture of fifth lumbar vertebra, initial encounter for open fracture |
| S32.052D | Unstable burst fracture of fifth lumbar vertebra, subsequent encounter for fracture with routine healing |
| S32.052G | Unstable burst fracture of fifth lumbar vertebra, subsequent encounter for fracture with delayed healing |
| S32.052K | Unstable burst fracture of fifth lumbar vertebra, subsequent encounter for fracture with nonunion |
| S32.052S | Unstable burst fracture of fifth lumbar vertebra, sequela |
| S32.058 | Other fracture of fifth lumbar vertebra |
| S32.058A | Other fracture of fifth lumbar vertebra, initial encounter for closed fracture |
| S32.058B | Other fracture of fifth lumbar vertebra, initial encounter for open fracture |
| S32.058D | Other fracture of fifth lumbar vertebra, subsequent encounter for fracture with routine healing |
| S32.058G | Other fracture of fifth lumbar vertebra, subsequent encounter for fracture with delayed healing |
| S32.058K | Other fracture of fifth lumbar vertebra, subsequent encounter for fracture with nonunion |
| S32.058S | Other fracture of fifth lumbar vertebra, sequela |
| S32.059 | Unspecified fracture of fifth lumbar vertebra |
| S32.059A | Unspecified fracture of fifth lumbar vertebra, initial encounter for closed fracture |
| S32.059B | Unspecified fracture of fifth lumbar vertebra, initial encounter for open fracture |
| S32.059D | Unspecified fracture of fifth lumbar vertebra, subsequent encounter for fracture with routine healing |
| S32.059G | Unspecified fracture of fifth lumbar vertebra, subsequent encounter for fracture with delayed healing |
| S32.059K | Unspecified fracture of fifth lumbar vertebra, subsequent encounter for fracture with nonunion |
| S32.059S | Unspecified fracture of fifth lumbar vertebra, sequela |
| S32.1 | Fracture of sacrum |
| S32.10 | Unspecified fracture of sacrum |
| S32.10XA | Unspecified fracture of sacrum, initial encounter for closed fracture |
| S32.10XB | Unspecified fracture of sacrum, initial encounter for open fracture |
| S32.10XD | Unspecified fracture of sacrum, subsequent encounter for fracture with routine healing |
| S32.10XG | Unspecified fracture of sacrum, subsequent encounter for fracture with delayed healing |
| S32.10XK | Unspecified fracture of sacrum, subsequent encounter for fracture with nonunion |
| S32.10XS | Unspecified fracture of sacrum, sequela |
| S32.11 | Zone I fracture of sacrum |
| S32.110 | Nondisplaced Zone I fracture of sacrum |
| S32.110A | Nondisplaced Zone I fracture of sacrum, initial encounter for closed fracture |
| S32.110B | Nondisplaced Zone I fracture of sacrum, initial encounter for open fracture |
| S32.110D | Nondisplaced Zone I fracture of sacrum, subsequent encounter for fracture with routine healing |
| S32.110G | Nondisplaced Zone I fracture of sacrum, subsequent encounter for fracture with delayed healing |
| S32.110K | Nondisplaced Zone I fracture of sacrum, subsequent encounter for fracture with nonunion |
| S32.110S | Nondisplaced Zone I fracture of sacrum, sequela |
| S32.111 | Minimally displaced Zone I fracture of sacrum |
| S32.111A | Minimally displaced Zone I fracture of sacrum, initial encounter for closed fracture |
| S32.111B | Minimally displaced Zone I fracture of sacrum, initial encounter for open fracture |
| S32.111D | Minimally displaced Zone I fracture of sacrum, subsequent encounter for fracture with routine healing |
| S32.111G | Minimally displaced Zone I fracture of sacrum, subsequent encounter for fracture with delayed healing |
| S32.111K | Minimally displaced Zone I fracture of sacrum, subsequent encounter for fracture with nonunion |
| S32.111S | Minimally displaced Zone I fracture of sacrum, sequela |
| S32.112 | Severely displaced Zone I fracture of sacrum |
| S32.112A | Severely displaced Zone I fracture of sacrum, initial encounter for closed fracture |
| S32.112B | Severely displaced Zone I fracture of sacrum, initial encounter for open fracture |
| S32.112D | Severely displaced Zone I fracture of sacrum, subsequent encounter for fracture with routine healing |
| S32.112G | Severely displaced Zone I fracture of sacrum, subsequent encounter for fracture with delayed healing |
| S32.112K | Severely displaced Zone I fracture of sacrum, subsequent encounter for fracture with nonunion |
| S32.112S | Severely displaced Zone I fracture of sacrum, sequela |
| S32.119 | Unspecified Zone I fracture of sacrum |
| S32.119A | Unspecified Zone I fracture of sacrum, initial encounter for closed fracture |
| S32.119B | Unspecified Zone I fracture of sacrum, initial encounter for open fracture |
| S32.119D | Unspecified Zone I fracture of sacrum, subsequent encounter for fracture with routine healing |
| S32.119G | Unspecified Zone I fracture of sacrum, subsequent encounter for fracture with delayed healing |
| S32.119K | Unspecified Zone I fracture of sacrum, subsequent encounter for fracture with nonunion |
| S32.119S | Unspecified Zone I fracture of sacrum, sequela |
| S32.12 | Zone II fracture of sacrum |
| S32.120 | Nondisplaced Zone II fracture of sacrum |
| S32.120A | Nondisplaced Zone II fracture of sacrum, initial encounter for closed fracture |
| S32.120B | Nondisplaced Zone II fracture of sacrum, initial encounter for open fracture |
| S32.120D | Nondisplaced Zone II fracture of sacrum, subsequent encounter for fracture with routine healing |
| S32.120G | Nondisplaced Zone II fracture of sacrum, subsequent encounter for fracture with delayed healing |
| S32.120K | Nondisplaced Zone II fracture of sacrum, subsequent encounter for fracture with nonunion |
| S32.120S | Nondisplaced Zone II fracture of sacrum, sequela |
| S32.121 | Minimally displaced Zone II fracture of sacrum |
| S32.121A | Minimally displaced Zone II fracture of sacrum, initial encounter for closed fracture |
| S32.121B | Minimally displaced Zone II fracture of sacrum, initial encounter for open fracture |
| S32.121D | Minimally displaced Zone II fracture of sacrum, subsequent encounter for fracture with routine healing |
| S32.121G | Minimally displaced Zone II fracture of sacrum, subsequent encounter for fracture with delayed healing |
| S32.121K | Minimally displaced Zone II fracture of sacrum, subsequent encounter for fracture with nonunion |
| S32.121S | Minimally displaced Zone II fracture of sacrum, sequela |
| S32.122 | Severely displaced Zone II fracture of sacrum |
| S32.122A | Severely displaced Zone II fracture of sacrum, initial encounter for closed fracture |
| S32.122B | Severely displaced Zone II fracture of sacrum, initial encounter for open fracture |
| S32.122D | Severely displaced Zone II fracture of sacrum, subsequent encounter for fracture with routine healing |
| S32.122G | Severely displaced Zone II fracture of sacrum, subsequent encounter for fracture with delayed healing |
| S32.122K | Severely displaced Zone II fracture of sacrum, subsequent encounter for fracture with nonunion |
| S32.122S | Severely displaced Zone II fracture of sacrum, sequela |
| S32.129 | Unspecified Zone II fracture of sacrum |
| S32.129A | Unspecified Zone II fracture of sacrum, initial encounter for closed fracture |
| S32.129B | Unspecified Zone II fracture of sacrum, initial encounter for open fracture |
| S32.129D | Unspecified Zone II fracture of sacrum, subsequent encounter for fracture with routine healing |
| S32.129G | Unspecified Zone II fracture of sacrum, subsequent encounter for fracture with delayed healing |
| S32.129K | Unspecified Zone II fracture of sacrum, subsequent encounter for fracture with nonunion |
| S32.129S | Unspecified Zone II fracture of sacrum, sequela |
| S32.13 | Zone III fracture of sacrum |
| S32.130 | Nondisplaced Zone III fracture of sacrum |
| S32.130A | Nondisplaced Zone III fracture of sacrum, initial encounter for closed fracture |
| S32.130B | Nondisplaced Zone III fracture of sacrum, initial encounter for open fracture |
| S32.130D | Nondisplaced Zone III fracture of sacrum, subsequent encounter for fracture with routine healing |
| S32.130G | Nondisplaced Zone III fracture of sacrum, subsequent encounter for fracture with delayed healing |
| S32.130K | Nondisplaced Zone III fracture of sacrum, subsequent encounter for fracture with nonunion |
| S32.130S | Nondisplaced Zone III fracture of sacrum, sequela |
| S32.131 | Minimally displaced Zone III fracture of sacrum |
| S32.131A | Minimally displaced Zone III fracture of sacrum, initial encounter for closed fracture |
| S32.131B | Minimally displaced Zone III fracture of sacrum, initial encounter for open fracture |
| S32.131D | Minimally displaced Zone III fracture of sacrum, subsequent encounter for fracture with routine healing |
| S32.131G | Minimally displaced Zone III fracture of sacrum, subsequent encounter for fracture with delayed healing |
| S32.131K | Minimally displaced Zone III fracture of sacrum, subsequent encounter for fracture with nonunion |
| S32.131S | Minimally displaced Zone III fracture of sacrum, sequela |
| S32.132 | Severely displaced Zone III fracture of sacrum |
| S32.132A | Severely displaced Zone III fracture of sacrum, initial encounter for closed fracture |
| S32.132B | Severely displaced Zone III fracture of sacrum, initial encounter for open fracture |
| S32.132D | Severely displaced Zone III fracture of sacrum, subsequent encounter for fracture with routine healing |
| S32.132G | Severely displaced Zone III fracture of sacrum, subsequent encounter for fracture with delayed healing |
| S32.132K | Severely displaced Zone III fracture of sacrum, subsequent encounter for fracture with nonunion |
| S32.132S | Severely displaced Zone III fracture of sacrum, sequela |
| S32.139 | Unspecified Zone III fracture of sacrum |
| S32.139A | Unspecified Zone III fracture of sacrum, initial encounter for closed fracture |
| S32.139B | Unspecified Zone III fracture of sacrum, initial encounter for open fracture |
| S32.139D | Unspecified Zone III fracture of sacrum, subsequent encounter for fracture with routine healing |
| S32.139G | Unspecified Zone III fracture of sacrum, subsequent encounter for fracture with delayed healing |
| S32.139K | Unspecified Zone III fracture of sacrum, subsequent encounter for fracture with nonunion |
| S32.139S | Unspecified Zone III fracture of sacrum, sequela |
| S32.14 | Type 1 fracture of sacrum |
| S32.14XA | Type 1 fracture of sacrum, initial encounter for closed fracture |
| S32.14XB | Type 1 fracture of sacrum, initial encounter for open fracture |
| S32.14XD | Type 1 fracture of sacrum, subsequent encounter for fracture with routine healing |
| S32.14XG | Type 1 fracture of sacrum, subsequent encounter for fracture with delayed healing |
| S32.14XK | Type 1 fracture of sacrum, subsequent encounter for fracture with nonunion |
| S32.14XS | Type 1 fracture of sacrum, sequela |
| S32.15 | Type 2 fracture of sacrum |
| S32.15XA | Type 2 fracture of sacrum, initial encounter for closed fracture |
| S32.15XB | Type 2 fracture of sacrum, initial encounter for open fracture |
| S32.15XD | Type 2 fracture of sacrum, subsequent encounter for fracture with routine healing |
| S32.15XG | Type 2 fracture of sacrum, subsequent encounter for fracture with delayed healing |
| S32.15XK | Type 2 fracture of sacrum, subsequent encounter for fracture with nonunion |
| S32.15XS | Type 2 fracture of sacrum, sequela |
| S32.16 | Type 3 fracture of sacrum |
| S32.16XA | Type 3 fracture of sacrum, initial encounter for closed fracture |
| S32.16XB | Type 3 fracture of sacrum, initial encounter for open fracture |
| S32.16XD | Type 3 fracture of sacrum, subsequent encounter for fracture with routine healing |
| S32.16XG | Type 3 fracture of sacrum, subsequent encounter for fracture with delayed healing |
| S32.16XK | Type 3 fracture of sacrum, subsequent encounter for fracture with nonunion |
| S32.16XS | Type 3 fracture of sacrum, sequela |
| S32.17 | Type 4 fracture of sacrum |
| S32.17XA | Type 4 fracture of sacrum, initial encounter for closed fracture |
| S32.17XB | Type 4 fracture of sacrum, initial encounter for open fracture |
| S32.17XD | Type 4 fracture of sacrum, subsequent encounter for fracture with routine healing |
| S32.17XG | Type 4 fracture of sacrum, subsequent encounter for fracture with delayed healing |
| S32.17XK | Type 4 fracture of sacrum, subsequent encounter for fracture with nonunion |
| S32.17XS | Type 4 fracture of sacrum, sequela |
| S32.19 | Other fracture of sacrum |
| S32.19XA | Other fracture of sacrum, initial encounter for closed fracture |
| S32.19XB | Other fracture of sacrum, initial encounter for open fracture |
| S32.19XD | Other fracture of sacrum, subsequent encounter for fracture with routine healing |
| S32.19XG | Other fracture of sacrum, subsequent encounter for fracture with delayed healing |
| S32.19XK | Other fracture of sacrum, subsequent encounter for fracture with nonunion |
| S32.19XS | Other fracture of sacrum, sequela |
| S32.2 | Fracture of coccyx |
| S32.2XXA | Fracture of coccyx, initial encounter for closed fracture |
| S32.2XXB | Fracture of coccyx, initial encounter for open fracture |
| S32.2XXD | Fracture of coccyx, subsequent encounter for fracture with routine healing |
| S32.2XXG | Fracture of coccyx, subsequent encounter for fracture with delayed healing |
| S32.2XXK | Fracture of coccyx, subsequent encounter for fracture with nonunion |
| S32.2XXS | Fracture of coccyx, sequela |
| S32.3 | Fracture of ilium |
| S32.30 | Unspecified fracture of ilium |
| S32.301 | Unspecified fracture of right ilium |
| S32.301A | Unspecified fracture of right ilium, initial encounter for closed fracture |
| S32.301B | Unspecified fracture of right ilium, initial encounter for open fracture |
| S32.301D | Unspecified fracture of right ilium, subsequent encounter for fracture with routine healing |
| S32.301G | Unspecified fracture of right ilium, subsequent encounter for fracture with delayed healing |
| S32.301K | Unspecified fracture of right ilium, subsequent encounter for fracture with nonunion |
| S32.301S | Unspecified fracture of right ilium, sequela |
| S32.302 | Unspecified fracture of left ilium |
| S32.302A | Unspecified fracture of left ilium, initial encounter for closed fracture |
| S32.302B | Unspecified fracture of left ilium, initial encounter for open fracture |
| S32.302D | Unspecified fracture of left ilium, subsequent encounter for fracture with routine healing |
| S32.302G | Unspecified fracture of left ilium, subsequent encounter for fracture with delayed healing |
| S32.302K | Unspecified fracture of left ilium, subsequent encounter for fracture with nonunion |
| S32.302S | Unspecified fracture of left ilium, sequela |
| S32.309 | Unspecified fracture of unspecified ilium |
| S32.309A | Unspecified fracture of unspecified ilium, initial encounter for closed fracture |
| S32.309B | Unspecified fracture of unspecified ilium, initial encounter for open fracture |
| S32.309D | Unspecified fracture of unspecified ilium, subsequent encounter for fracture with routine healing |
| S32.309G | Unspecified fracture of unspecified ilium, subsequent encounter for fracture with delayed healing |
| S32.309K | Unspecified fracture of unspecified ilium, subsequent encounter for fracture with nonunion |
| S32.309S | Unspecified fracture of unspecified ilium, sequela |
| S32.31 | Avulsion fracture of ilium |
| S32.311 | Displaced avulsion fracture of right ilium |
| S32.311A | Displaced avulsion fracture of right ilium, initial encounter for closed fracture |
| S32.311B | Displaced avulsion fracture of right ilium, initial encounter for open fracture |
| S32.311D | Displaced avulsion fracture of right ilium, subsequent encounter for fracture with routine healing |
| S32.311G | Displaced avulsion fracture of right ilium, subsequent encounter for fracture with delayed healing |
| S32.311K | Displaced avulsion fracture of right ilium, subsequent encounter for fracture with nonunion |
| S32.311S | Displaced avulsion fracture of right ilium, sequela |
| S32.312 | Displaced avulsion fracture of left ilium |
| S32.312A | Displaced avulsion fracture of left ilium, initial encounter for closed fracture |
| S32.312B | Displaced avulsion fracture of left ilium, initial encounter for open fracture |
| S32.312D | Displaced avulsion fracture of left ilium, subsequent encounter for fracture with routine healing |
| S32.312G | Displaced avulsion fracture of left ilium, subsequent encounter for fracture with delayed healing |
| S32.312K | Displaced avulsion fracture of left ilium, subsequent encounter for fracture with nonunion |
| S32.312S | Displaced avulsion fracture of left ilium, sequela |
| S32.313 | Displaced avulsion fracture of unspecified ilium |
| S32.313A | Displaced avulsion fracture of unspecified ilium, initial encounter for closed fracture |
| S32.313B | Displaced avulsion fracture of unspecified ilium, initial encounter for open fracture |
| S32.313D | Displaced avulsion fracture of unspecified ilium, subsequent encounter for fracture with routine healing |
| S32.313G | Displaced avulsion fracture of unspecified ilium, subsequent encounter for fracture with delayed healing |
| S32.313K | Displaced avulsion fracture of unspecified ilium, subsequent encounter for fracture with nonunion |
| S32.313S | Displaced avulsion fracture of unspecified ilium, sequela |
| S32.314 | Nondisplaced avulsion fracture of right ilium |
| S32.314A | Nondisplaced avulsion fracture of right ilium, initial encounter for closed fracture |
| S32.314B | Nondisplaced avulsion fracture of right ilium, initial encounter for open fracture |
| S32.314D | Nondisplaced avulsion fracture of right ilium, subsequent encounter for fracture with routine healing |
| S32.314G | Nondisplaced avulsion fracture of right ilium, subsequent encounter for fracture with delayed healing |
| S32.314K | Nondisplaced avulsion fracture of right ilium, subsequent encounter for fracture with nonunion |
| S32.314S | Nondisplaced avulsion fracture of right ilium, sequela |
| S32.315 | Nondisplaced avulsion fracture of left ilium |
| S32.315A | Nondisplaced avulsion fracture of left ilium, initial encounter for closed fracture |
| S32.315B | Nondisplaced avulsion fracture of left ilium, initial encounter for open fracture |
| S32.315D | Nondisplaced avulsion fracture of left ilium, subsequent encounter for fracture with routine healing |
| S32.315G | Nondisplaced avulsion fracture of left ilium, subsequent encounter for fracture with delayed healing |
| S32.315K | Nondisplaced avulsion fracture of left ilium, subsequent encounter for fracture with nonunion |
| S32.315S | Nondisplaced avulsion fracture of left ilium, sequela |
| S32.316 | Nondisplaced avulsion fracture of unspecified ilium |
| S32.316A | Nondisplaced avulsion fracture of unspecified ilium, initial encounter for closed fracture |
| S32.316B | Nondisplaced avulsion fracture of unspecified ilium, initial encounter for open fracture |
| S32.316D | Nondisplaced avulsion fracture of unspecified ilium, subsequent encounter for fracture with routine healing |
| S32.316G | Nondisplaced avulsion fracture of unspecified ilium, subsequent encounter for fracture with delayed healing |
| S32.316K | Nondisplaced avulsion fracture of unspecified ilium, subsequent encounter for fracture with nonunion |
| S32.316S | Nondisplaced avulsion fracture of unspecified ilium, sequela |
| S32.39 | Other fracture of ilium |
| S32.391 | Other fracture of right ilium |
| S32.391A | Other fracture of right ilium, initial encounter for closed fracture |
| S32.391B | Other fracture of right ilium, initial encounter for open fracture |
| S32.391D | Other fracture of right ilium, subsequent encounter for fracture with routine healing |
| S32.391G | Other fracture of right ilium, subsequent encounter for fracture with delayed healing |
| S32.391K | Other fracture of right ilium, subsequent encounter for fracture with nonunion |
| S32.391S | Other fracture of right ilium, sequela |
| S32.392 | Other fracture of left ilium |
| S32.392A | Other fracture of left ilium, initial encounter for closed fracture |
| S32.392B | Other fracture of left ilium, initial encounter for open fracture |
| S32.392D | Other fracture of left ilium, subsequent encounter for fracture with routine healing |
| S32.392G | Other fracture of left ilium, subsequent encounter for fracture with delayed healing |
| S32.392K | Other fracture of left ilium, subsequent encounter for fracture with nonunion |
| S32.392S | Other fracture of left ilium, sequela |
| S32.399 | Other fracture of unspecified ilium |
| S32.399A | Other fracture of unspecified ilium, initial encounter for closed fracture |
| S32.399B | Other fracture of unspecified ilium, initial encounter for open fracture |
| S32.399D | Other fracture of unspecified ilium, subsequent encounter for fracture with routine healing |
| S32.399G | Other fracture of unspecified ilium, subsequent encounter for fracture with delayed healing |
| S32.399K | Other fracture of unspecified ilium, subsequent encounter for fracture with nonunion |
| S32.399S | Other fracture of unspecified ilium, sequela |
| S32.4 | Fracture of acetabulum |
| S32.40 | Unspecified fracture of acetabulum |
| S32.401 | Unspecified fracture of right acetabulum |
| S32.401A | Unspecified fracture of right acetabulum, initial encounter for closed fracture |
| S32.401B | Unspecified fracture of right acetabulum, initial encounter for open fracture |
| S32.401D | Unspecified fracture of right acetabulum, subsequent encounter for fracture with routine healing |
| S32.401G | Unspecified fracture of right acetabulum, subsequent encounter for fracture with delayed healing |
| S32.401K | Unspecified fracture of right acetabulum, subsequent encounter for fracture with nonunion |
| S32.401S | Unspecified fracture of right acetabulum, sequela |
| S32.402 | Unspecified fracture of left acetabulum |
| S32.402A | Unspecified fracture of left acetabulum, initial encounter for closed fracture |
| S32.402B | Unspecified fracture of left acetabulum, initial encounter for open fracture |
| S32.402D | Unspecified fracture of left acetabulum, subsequent encounter for fracture with routine healing |
| S32.402G | Unspecified fracture of left acetabulum, subsequent encounter for fracture with delayed healing |
| S32.402K | Unspecified fracture of left acetabulum, subsequent encounter for fracture with nonunion |
| S32.402S | Unspecified fracture of left acetabulum, sequela |
| S32.409 | Unspecified fracture of unspecified acetabulum |
| S32.409A | Unspecified fracture of unspecified acetabulum, initial encounter for closed fracture |
| S32.409B | Unspecified fracture of unspecified acetabulum, initial encounter for open fracture |
| S32.409D | Unspecified fracture of unspecified acetabulum, subsequent encounter for fracture with routine healing |
| S32.409G | Unspecified fracture of unspecified acetabulum, subsequent encounter for fracture with delayed healing |
| S32.409K | Unspecified fracture of unspecified acetabulum, subsequent encounter for fracture with nonunion |
| S32.409S | Unspecified fracture of unspecified acetabulum, sequela |
| S32.41 | Fracture of anterior wall of acetabulum |
| S32.411 | Displaced fracture of anterior wall of right acetabulum |
| S32.411A | Displaced fracture of anterior wall of right acetabulum, initial encounter for closed fracture |
| S32.411B | Displaced fracture of anterior wall of right acetabulum, initial encounter for open fracture |
| S32.411D | Displaced fracture of anterior wall of right acetabulum, subsequent encounter for fracture with routine healing |
| S32.411G | Displaced fracture of anterior wall of right acetabulum, subsequent encounter for fracture with delayed healing |
| S32.411K | Displaced fracture of anterior wall of right acetabulum, subsequent encounter for fracture with nonunion |
| S32.411S | Displaced fracture of anterior wall of right acetabulum, sequela |
| S32.412 | Displaced fracture of anterior wall of left acetabulum |
| S32.412A | Displaced fracture of anterior wall of left acetabulum, initial encounter for closed fracture |
| S32.412B | Displaced fracture of anterior wall of left acetabulum, initial encounter for open fracture |
| S32.412D | Displaced fracture of anterior wall of left acetabulum, subsequent encounter for fracture with routine healing |
| S32.412G | Displaced fracture of anterior wall of left acetabulum, subsequent encounter for fracture with delayed healing |
| S32.412K | Displaced fracture of anterior wall of left acetabulum, subsequent encounter for fracture with nonunion |
| S32.412S | Displaced fracture of anterior wall of left acetabulum, sequela |
| S32.413 | Displaced fracture of anterior wall of unspecified acetabulum |
| S32.413A | Displaced fracture of anterior wall of unspecified acetabulum, initial encounter for closed fracture |
| S32.413B | Displaced fracture of anterior wall of unspecified acetabulum, initial encounter for open fracture |
| S32.413D | Displaced fracture of anterior wall of unspecified acetabulum, subsequent encounter for fracture with routine healing |
| S32.413G | Displaced fracture of anterior wall of unspecified acetabulum, subsequent encounter for fracture with delayed healing |
| S32.413K | Displaced fracture of anterior wall of unspecified acetabulum, subsequent encounter for fracture with nonunion |
| S32.413S | Displaced fracture of anterior wall of unspecified acetabulum, sequela |
| S32.414 | Nondisplaced fracture of anterior wall of right acetabulum |
| S32.414A | Nondisplaced fracture of anterior wall of right acetabulum, initial encounter for closed fracture |
| S32.414B | Nondisplaced fracture of anterior wall of right acetabulum, initial encounter for open fracture |
| S32.414D | Nondisplaced fracture of anterior wall of right acetabulum, subsequent encounter for fracture with routine healing |
| S32.414G | Nondisplaced fracture of anterior wall of right acetabulum, subsequent encounter for fracture with delayed healing |
| S32.414K | Nondisplaced fracture of anterior wall of right acetabulum, subsequent encounter for fracture with nonunion |
| S32.414S | Nondisplaced fracture of anterior wall of right acetabulum, sequela |
| S32.415 | Nondisplaced fracture of anterior wall of left acetabulum |
| S32.415A | Nondisplaced fracture of anterior wall of left acetabulum, initial encounter for closed fracture |
| S32.415B | Nondisplaced fracture of anterior wall of left acetabulum, initial encounter for open fracture |
| S32.415D | Nondisplaced fracture of anterior wall of left acetabulum, subsequent encounter for fracture with routine healing |
| S32.415G | Nondisplaced fracture of anterior wall of left acetabulum, subsequent encounter for fracture with delayed healing |
| S32.415K | Nondisplaced fracture of anterior wall of left acetabulum, subsequent encounter for fracture with nonunion |
| S32.415S | Nondisplaced fracture of anterior wall of left acetabulum, sequela |
| S32.416 | Nondisplaced fracture of anterior wall of unspecified acetabulum |
| S32.416A | Nondisplaced fracture of anterior wall of unspecified acetabulum, initial encounter for closed fracture |
| S32.416B | Nondisplaced fracture of anterior wall of unspecified acetabulum, initial encounter for open fracture |
| S32.416D | Nondisplaced fracture of anterior wall of unspecified acetabulum, subsequent encounter for fracture with routine healing |
| S32.416G | Nondisplaced fracture of anterior wall of unspecified acetabulum, subsequent encounter for fracture with delayed healing |
| S32.416K | Nondisplaced fracture of anterior wall of unspecified acetabulum, subsequent encounter for fracture with nonunion |
| S32.416S | Nondisplaced fracture of anterior wall of unspecified acetabulum, sequela |
| S32.42 | Fracture of posterior wall of acetabulum |
| S32.421 | Displaced fracture of posterior wall of right acetabulum |
| S32.421A | Displaced fracture of posterior wall of right acetabulum, initial encounter for closed fracture |
| S32.421B | Displaced fracture of posterior wall of right acetabulum, initial encounter for open fracture |
| S32.421D | Displaced fracture of posterior wall of right acetabulum, subsequent encounter for fracture with routine healing |
| S32.421G | Displaced fracture of posterior wall of right acetabulum, subsequent encounter for fracture with delayed healing |
| S32.421K | Displaced fracture of posterior wall of right acetabulum, subsequent encounter for fracture with nonunion |
| S32.421S | Displaced fracture of posterior wall of right acetabulum, sequela |
| S32.422 | Displaced fracture of posterior wall of left acetabulum |
| S32.422A | Displaced fracture of posterior wall of left acetabulum, initial encounter for closed fracture |
| S32.422B | Displaced fracture of posterior wall of left acetabulum, initial encounter for open fracture |
| S32.422D | Displaced fracture of posterior wall of left acetabulum, subsequent encounter for fracture with routine healing |
| S32.422G | Displaced fracture of posterior wall of left acetabulum, subsequent encounter for fracture with delayed healing |
| S32.422K | Displaced fracture of posterior wall of left acetabulum, subsequent encounter for fracture with nonunion |
| S32.422S | Displaced fracture of posterior wall of left acetabulum, sequela |
| S32.423 | Displaced fracture of posterior wall of unspecified acetabulum |
| S32.423A | Displaced fracture of posterior wall of unspecified acetabulum, initial encounter for closed fracture |
| S32.423B | Displaced fracture of posterior wall of unspecified acetabulum, initial encounter for open fracture |
| S32.423D | Displaced fracture of posterior wall of unspecified acetabulum, subsequent encounter for fracture with routine healing |
| S32.423G | Displaced fracture of posterior wall of unspecified acetabulum, subsequent encounter for fracture with delayed healing |
| S32.423K | Displaced fracture of posterior wall of unspecified acetabulum, subsequent encounter for fracture with nonunion |
| S32.423S | Displaced fracture of posterior wall of unspecified acetabulum, sequela |
| S32.424 | Nondisplaced fracture of posterior wall of right acetabulum |
| S32.424A | Nondisplaced fracture of posterior wall of right acetabulum, initial encounter for closed fracture |
| S32.424B | Nondisplaced fracture of posterior wall of right acetabulum, initial encounter for open fracture |
| S32.424D | Nondisplaced fracture of posterior wall of right acetabulum, subsequent encounter for fracture with routine healing |
| S32.424G | Nondisplaced fracture of posterior wall of right acetabulum, subsequent encounter for fracture with delayed healing |
| S32.424K | Nondisplaced fracture of posterior wall of right acetabulum, subsequent encounter for fracture with nonunion |
| S32.424S | Nondisplaced fracture of posterior wall of right acetabulum, sequela |
| S32.425 | Nondisplaced fracture of posterior wall of left acetabulum |
| S32.425A | Nondisplaced fracture of posterior wall of left acetabulum, initial encounter for closed fracture |
| S32.425B | Nondisplaced fracture of posterior wall of left acetabulum, initial encounter for open fracture |
| S32.425D | Nondisplaced fracture of posterior wall of left acetabulum, subsequent encounter for fracture with routine healing |
| S32.425G | Nondisplaced fracture of posterior wall of left acetabulum, subsequent encounter for fracture with delayed healing |
| S32.425K | Nondisplaced fracture of posterior wall of left acetabulum, subsequent encounter for fracture with nonunion |
| S32.425S | Nondisplaced fracture of posterior wall of left acetabulum, sequela |
| S32.426 | Nondisplaced fracture of posterior wall of unspecified acetabulum |
| S32.426A | Nondisplaced fracture of posterior wall of unspecified acetabulum, initial encounter for closed fracture |
| S32.426B | Nondisplaced fracture of posterior wall of unspecified acetabulum, initial encounter for open fracture |
| S32.426D | Nondisplaced fracture of posterior wall of unspecified acetabulum, subsequent encounter for fracture with routine healing |
| S32.426G | Nondisplaced fracture of posterior wall of unspecified acetabulum, subsequent encounter for fracture with delayed healing |
| S32.426K | Nondisplaced fracture of posterior wall of unspecified acetabulum, subsequent encounter for fracture with nonunion |
| S32.426S | Nondisplaced fracture of posterior wall of unspecified acetabulum, sequela |
| S32.43 | Fracture of anterior column [iliopubic] of acetabulum |
| S32.431 | Displaced fracture of anterior column [iliopubic] of right acetabulum |
| S32.431A | Displaced fracture of anterior column [iliopubic] of right acetabulum, initial encounter for closed fracture |
| S32.431B | Displaced fracture of anterior column [iliopubic] of right acetabulum, initial encounter for open fracture |
| S32.431D | Displaced fracture of anterior column [iliopubic] of right acetabulum, subsequent encounter for fracture with routine healing |
| S32.431G | Displaced fracture of anterior column [iliopubic] of right acetabulum, subsequent encounter for fracture with delayed healing |
| S32.431K | Displaced fracture of anterior column [iliopubic] of right acetabulum, subsequent encounter for fracture with nonunion |
| S32.431S | Displaced fracture of anterior column [iliopubic] of right acetabulum, sequela |
| S32.432 | Displaced fracture of anterior column [iliopubic] of left acetabulum |
| S32.432A | Displaced fracture of anterior column [iliopubic] of left acetabulum, initial encounter for closed fracture |
| S32.432B | Displaced fracture of anterior column [iliopubic] of left acetabulum, initial encounter for open fracture |
| S32.432D | Displaced fracture of anterior column [iliopubic] of left acetabulum, subsequent encounter for fracture with routine healing |
| S32.432G | Displaced fracture of anterior column [iliopubic] of left acetabulum, subsequent encounter for fracture with delayed healing |
| S32.432K | Displaced fracture of anterior column [iliopubic] of left acetabulum, subsequent encounter for fracture with nonunion |
| S32.432S | Displaced fracture of anterior column [iliopubic] of left acetabulum, sequela |
| S32.433 | Displaced fracture of anterior column [iliopubic] of unspecified acetabulum |
| S32.433A | Displaced fracture of anterior column [iliopubic] of unspecified acetabulum, initial encounter for closed fracture |
| S32.433B | Displaced fracture of anterior column [iliopubic] of unspecified acetabulum, initial encounter for open fracture |
| S32.433D | Displaced fracture of anterior column [iliopubic] of unspecified acetabulum, subsequent encounter for fracture with routine healing |
| S32.433G | Displaced fracture of anterior column [iliopubic] of unspecified acetabulum, subsequent encounter for fracture with delayed healing |
| S32.433K | Displaced fracture of anterior column [iliopubic] of unspecified acetabulum, subsequent encounter for fracture with nonunion |
| S32.433S | Displaced fracture of anterior column [iliopubic] of unspecified acetabulum, sequela |
| S32.434 | Nondisplaced fracture of anterior column [iliopubic] of right acetabulum |
| S32.434A | Nondisplaced fracture of anterior column [iliopubic] of right acetabulum, initial encounter for closed fracture |
| S32.434B | Nondisplaced fracture of anterior column [iliopubic] of right acetabulum, initial encounter for open fracture |
| S32.434D | Nondisplaced fracture of anterior column [iliopubic] of right acetabulum, subsequent encounter for fracture with routine healing |
| S32.434G | Nondisplaced fracture of anterior column [iliopubic] of right acetabulum, subsequent encounter for fracture with delayed healing |
| S32.434K | Nondisplaced fracture of anterior column [iliopubic] of right acetabulum, subsequent encounter for fracture with nonunion |
| S32.434S | Nondisplaced fracture of anterior column [iliopubic] of right acetabulum, sequela |
| S32.435 | Nondisplaced fracture of anterior column [iliopubic] of left acetabulum |
| S32.435A | Nondisplaced fracture of anterior column [iliopubic] of left acetabulum, initial encounter for closed fracture |
| S32.435B | Nondisplaced fracture of anterior column [iliopubic] of left acetabulum, initial encounter for open fracture |
| S32.435D | Nondisplaced fracture of anterior column [iliopubic] of left acetabulum, subsequent encounter for fracture with routine healing |
| S32.435G | Nondisplaced fracture of anterior column [iliopubic] of left acetabulum, subsequent encounter for fracture with delayed healing |
| S32.435K | Nondisplaced fracture of anterior column [iliopubic] of left acetabulum, subsequent encounter for fracture with nonunion |
| S32.435S | Nondisplaced fracture of anterior column [iliopubic] of left acetabulum, sequela |
| S32.436 | Nondisplaced fracture of anterior column [iliopubic] of unspecified acetabulum |
| S32.436A | Nondisplaced fracture of anterior column [iliopubic] of unspecified acetabulum, initial encounter for closed fracture |
| S32.436B | Nondisplaced fracture of anterior column [iliopubic] of unspecified acetabulum, initial encounter for open fracture |
| S32.436D | Nondisplaced fracture of anterior column [iliopubic] of unspecified acetabulum, subsequent encounter for fracture with routine healing |
| S32.436G | Nondisplaced fracture of anterior column [iliopubic] of unspecified acetabulum, subsequent encounter for fracture with delayed healing |
| S32.436K | Nondisplaced fracture of anterior column [iliopubic] of unspecified acetabulum, subsequent encounter for fracture with nonunion |
| S32.436S | Nondisplaced fracture of anterior column [iliopubic] of unspecified acetabulum, sequela |
| S32.44 | Fracture of posterior column [ilioischial] of acetabulum |
| S32.441 | Displaced fracture of posterior column [ilioischial] of right acetabulum |
| S32.441A | Displaced fracture of posterior column [ilioischial] of right acetabulum, initial encounter for closed fracture |
| S32.441B | Displaced fracture of posterior column [ilioischial] of right acetabulum, initial encounter for open fracture |
| S32.441D | Displaced fracture of posterior column [ilioischial] of right acetabulum, subsequent encounter for fracture with routine healing |
| S32.441G | Displaced fracture of posterior column [ilioischial] of right acetabulum, subsequent encounter for fracture with delayed healing |
| S32.441K | Displaced fracture of posterior column [ilioischial] of right acetabulum, subsequent encounter for fracture with nonunion |
| S32.441S | Displaced fracture of posterior column [ilioischial] of right acetabulum, sequela |
| S32.442 | Displaced fracture of posterior column [ilioischial] of left acetabulum |
| S32.442A | Displaced fracture of posterior column [ilioischial] of left acetabulum, initial encounter for closed fracture |
| S32.442B | Displaced fracture of posterior column [ilioischial] of left acetabulum, initial encounter for open fracture |
| S32.442D | Displaced fracture of posterior column [ilioischial] of left acetabulum, subsequent encounter for fracture with routine healing |
| S32.442G | Displaced fracture of posterior column [ilioischial] of left acetabulum, subsequent encounter for fracture with delayed healing |
| S32.442K | Displaced fracture of posterior column [ilioischial] of left acetabulum, subsequent encounter for fracture with nonunion |
| S32.442S | Displaced fracture of posterior column [ilioischial] of left acetabulum, sequela |
| S32.443 | Displaced fracture of posterior column [ilioischial] of unspecified acetabulum |
| S32.443A | Displaced fracture of posterior column [ilioischial] of unspecified acetabulum, initial encounter for closed fracture |
| S32.443B | Displaced fracture of posterior column [ilioischial] of unspecified acetabulum, initial encounter for open fracture |
| S32.443D | Displaced fracture of posterior column [ilioischial] of unspecified acetabulum, subsequent encounter for fracture with routine healing |
| S32.443G | Displaced fracture of posterior column [ilioischial] of unspecified acetabulum, subsequent encounter for fracture with delayed healing |
| S32.443K | Displaced fracture of posterior column [ilioischial] of unspecified acetabulum, subsequent encounter for fracture with nonunion |
| S32.443S | Displaced fracture of posterior column [ilioischial] of unspecified acetabulum, sequela |
| S32.444 | Nondisplaced fracture of posterior column [ilioischial] of right acetabulum |
| S32.444A | Nondisplaced fracture of posterior column [ilioischial] of right acetabulum, initial encounter for closed fracture |
| S32.444B | Nondisplaced fracture of posterior column [ilioischial] of right acetabulum, initial encounter for open fracture |
| S32.444D | Nondisplaced fracture of posterior column [ilioischial] of right acetabulum, subsequent encounter for fracture with routine healing |
| S32.444G | Nondisplaced fracture of posterior column [ilioischial] of right acetabulum, subsequent encounter for fracture with delayed healing |
| S32.444K | Nondisplaced fracture of posterior column [ilioischial] of right acetabulum, subsequent encounter for fracture with nonunion |
| S32.444S | Nondisplaced fracture of posterior column [ilioischial] of right acetabulum, sequela |
| S32.445 | Nondisplaced fracture of posterior column [ilioischial] of left acetabulum |
| S32.445A | Nondisplaced fracture of posterior column [ilioischial] of left acetabulum, initial encounter for closed fracture |
| S32.445B | Nondisplaced fracture of posterior column [ilioischial] of left acetabulum, initial encounter for open fracture |
| S32.445D | Nondisplaced fracture of posterior column [ilioischial] of left acetabulum, subsequent encounter for fracture with routine healing |
| S32.445G | Nondisplaced fracture of posterior column [ilioischial] of left acetabulum, subsequent encounter for fracture with delayed healing |
| S32.445K | Nondisplaced fracture of posterior column [ilioischial] of left acetabulum, subsequent encounter for fracture with nonunion |
| S32.445S | Nondisplaced fracture of posterior column [ilioischial] of left acetabulum, sequela |
| S32.446 | Nondisplaced fracture of posterior column [ilioischial] of unspecified acetabulum |
| S32.446A | Nondisplaced fracture of posterior column [ilioischial] of unspecified acetabulum, initial encounter for closed fracture |
| S32.446B | Nondisplaced fracture of posterior column [ilioischial] of unspecified acetabulum, initial encounter for open fracture |
| S32.446D | Nondisplaced fracture of posterior column [ilioischial] of unspecified acetabulum, subsequent encounter for fracture with routine healing |
| S32.446G | Nondisplaced fracture of posterior column [ilioischial] of unspecified acetabulum, subsequent encounter for fracture with delayed healing |
| S32.446K | Nondisplaced fracture of posterior column [ilioischial] of unspecified acetabulum, subsequent encounter for fracture with nonunion |
| S32.446S | Nondisplaced fracture of posterior column [ilioischial] of unspecified acetabulum, sequela |
| S32.45 | Transverse fracture of acetabulum |
| S32.451 | Displaced transverse fracture of right acetabulum |
| S32.451A | Displaced transverse fracture of right acetabulum, initial encounter for closed fracture |
| S32.451B | Displaced transverse fracture of right acetabulum, initial encounter for open fracture |
| S32.451D | Displaced transverse fracture of right acetabulum, subsequent encounter for fracture with routine healing |
| S32.451G | Displaced transverse fracture of right acetabulum, subsequent encounter for fracture with delayed healing |
| S32.451K | Displaced transverse fracture of right acetabulum, subsequent encounter for fracture with nonunion |
| S32.451S | Displaced transverse fracture of right acetabulum, sequela |
| S32.452 | Displaced transverse fracture of left acetabulum |
| S32.452A | Displaced transverse fracture of left acetabulum, initial encounter for closed fracture |
| S32.452B | Displaced transverse fracture of left acetabulum, initial encounter for open fracture |
| S32.452D | Displaced transverse fracture of left acetabulum, subsequent encounter for fracture with routine healing |
| S32.452G | Displaced transverse fracture of left acetabulum, subsequent encounter for fracture with delayed healing |
| S32.452K | Displaced transverse fracture of left acetabulum, subsequent encounter for fracture with nonunion |
| S32.452S | Displaced transverse fracture of left acetabulum, sequela |
| S32.453 | Displaced transverse fracture of unspecified acetabulum |
| S32.453A | Displaced transverse fracture of unspecified acetabulum, initial encounter for closed fracture |
| S32.453B | Displaced transverse fracture of unspecified acetabulum, initial encounter for open fracture |
| S32.453D | Displaced transverse fracture of unspecified acetabulum, subsequent encounter for fracture with routine healing |
| S32.453G | Displaced transverse fracture of unspecified acetabulum, subsequent encounter for fracture with delayed healing |
| S32.453K | Displaced transverse fracture of unspecified acetabulum, subsequent encounter for fracture with nonunion |
| S32.453S | Displaced transverse fracture of unspecified acetabulum, sequela |
| S32.454 | Nondisplaced transverse fracture of right acetabulum |
| S32.454A | Nondisplaced transverse fracture of right acetabulum, initial encounter for closed fracture |
| S32.454B | Nondisplaced transverse fracture of right acetabulum, initial encounter for open fracture |
| S32.454D | Nondisplaced transverse fracture of right acetabulum, subsequent encounter for fracture with routine healing |
| S32.454G | Nondisplaced transverse fracture of right acetabulum, subsequent encounter for fracture with delayed healing |
| S32.454K | Nondisplaced transverse fracture of right acetabulum, subsequent encounter for fracture with nonunion |
| S32.454S | Nondisplaced transverse fracture of right acetabulum, sequela |
| S32.455 | Nondisplaced transverse fracture of left acetabulum |
| S32.455A | Nondisplaced transverse fracture of left acetabulum, initial encounter for closed fracture |
| S32.455B | Nondisplaced transverse fracture of left acetabulum, initial encounter for open fracture |
| S32.455D | Nondisplaced transverse fracture of left acetabulum, subsequent encounter for fracture with routine healing |
| S32.455G | Nondisplaced transverse fracture of left acetabulum, subsequent encounter for fracture with delayed healing |
| S32.455K | Nondisplaced transverse fracture of left acetabulum, subsequent encounter for fracture with nonunion |
| S32.455S | Nondisplaced transverse fracture of left acetabulum, sequela |
| S32.456 | Nondisplaced transverse fracture of unspecified acetabulum |
| S32.456A | Nondisplaced transverse fracture of unspecified acetabulum, initial encounter for closed fracture |
| S32.456B | Nondisplaced transverse fracture of unspecified acetabulum, initial encounter for open fracture |
| S32.456D | Nondisplaced transverse fracture of unspecified acetabulum, subsequent encounter for fracture with routine healing |
| S32.456G | Nondisplaced transverse fracture of unspecified acetabulum, subsequent encounter for fracture with delayed healing |
| S32.456K | Nondisplaced transverse fracture of unspecified acetabulum, subsequent encounter for fracture with nonunion |
| S32.456S | Nondisplaced transverse fracture of unspecified acetabulum, sequela |
| S32.46 | Associated transverse-posterior fracture of acetabulum |
| S32.461 | Displaced associated transverse-posterior fracture of right acetabulum |
| S32.461A | Displaced associated transverse-posterior fracture of right acetabulum, initial encounter for closed fracture |
| S32.461B | Displaced associated transverse-posterior fracture of right acetabulum, initial encounter for open fracture |
| S32.461D | Displaced associated transverse-posterior fracture of right acetabulum, subsequent encounter for fracture with routine healing |
| S32.461G | Displaced associated transverse-posterior fracture of right acetabulum, subsequent encounter for fracture with delayed healing |
| S32.461K | Displaced associated transverse-posterior fracture of right acetabulum, subsequent encounter for fracture with nonunion |
| S32.461S | Displaced associated transverse-posterior fracture of right acetabulum, sequela |
| S32.462 | Displaced associated transverse-posterior fracture of left acetabulum |
| S32.462A | Displaced associated transverse-posterior fracture of left acetabulum, initial encounter for closed fracture |
| S32.462B | Displaced associated transverse-posterior fracture of left acetabulum, initial encounter for open fracture |
| S32.462D | Displaced associated transverse-posterior fracture of left acetabulum, subsequent encounter for fracture with routine healing |
| S32.462G | Displaced associated transverse-posterior fracture of left acetabulum, subsequent encounter for fracture with delayed healing |
| S32.462K | Displaced associated transverse-posterior fracture of left acetabulum, subsequent encounter for fracture with nonunion |
| S32.462S | Displaced associated transverse-posterior fracture of left acetabulum, sequela |
| S32.463 | Displaced associated transverse-posterior fracture of unspecified acetabulum |
| S32.463A | Displaced associated transverse-posterior fracture of unspecified acetabulum, initial encounter for closed fracture |
| S32.463B | Displaced associated transverse-posterior fracture of unspecified acetabulum, initial encounter for open fracture |
| S32.463D | Displaced associated transverse-posterior fracture of unspecified acetabulum, subsequent encounter for fracture with routine healing |
| S32.463G | Displaced associated transverse-posterior fracture of unspecified acetabulum, subsequent encounter for fracture with delayed healing |
| S32.463K | Displaced associated transverse-posterior fracture of unspecified acetabulum, subsequent encounter for fracture with nonunion |
| S32.463S | Displaced associated transverse-posterior fracture of unspecified acetabulum, sequela |
| S32.464 | Nondisplaced associated transverse-posterior fracture of right acetabulum |
| S32.464A | Nondisplaced associated transverse-posterior fracture of right acetabulum, initial encounter for closed fracture |
| S32.464B | Nondisplaced associated transverse-posterior fracture of right acetabulum, initial encounter for open fracture |
| S32.464D | Nondisplaced associated transverse-posterior fracture of right acetabulum, subsequent encounter for fracture with routine healing |
| S32.464G | Nondisplaced associated transverse-posterior fracture of right acetabulum, subsequent encounter for fracture with delayed healing |
| S32.464K | Nondisplaced associated transverse-posterior fracture of right acetabulum, subsequent encounter for fracture with nonunion |
| S32.464S | Nondisplaced associated transverse-posterior fracture of right acetabulum, sequela |
| S32.465 | Nondisplaced associated transverse-posterior fracture of left acetabulum |
| S32.465A | Nondisplaced associated transverse-posterior fracture of left acetabulum, initial encounter for closed fracture |
| S32.465B | Nondisplaced associated transverse-posterior fracture of left acetabulum, initial encounter for open fracture |
| S32.465D | Nondisplaced associated transverse-posterior fracture of left acetabulum, subsequent encounter for fracture with routine healing |
| S32.465G | Nondisplaced associated transverse-posterior fracture of left acetabulum, subsequent encounter for fracture with delayed healing |
| S32.465K | Nondisplaced associated transverse-posterior fracture of left acetabulum, subsequent encounter for fracture with nonunion |
| S32.465S | Nondisplaced associated transverse-posterior fracture of left acetabulum, sequela |
| S32.466 | Nondisplaced associated transverse-posterior fracture of unspecified acetabulum |
| S32.466A | Nondisplaced associated transverse-posterior fracture of unspecified acetabulum, initial encounter for closed fracture |
| S32.466B | Nondisplaced associated transverse-posterior fracture of unspecified acetabulum, initial encounter for open fracture |
| S32.466D | Nondisplaced associated transverse-posterior fracture of unspecified acetabulum, subsequent encounter for fracture with routine healing |
| S32.466G | Nondisplaced associated transverse-posterior fracture of unspecified acetabulum, subsequent encounter for fracture with delayed healing |
| S32.466K | Nondisplaced associated transverse-posterior fracture of unspecified acetabulum, subsequent encounter for fracture with nonunion |
| S32.466S | Nondisplaced associated transverse-posterior fracture of unspecified acetabulum, sequela |
| S32.47 | Fracture of medial wall of acetabulum |
| S32.471 | Displaced fracture of medial wall of right acetabulum |
| S32.471A | Displaced fracture of medial wall of right acetabulum, initial encounter for closed fracture |
| S32.471B | Displaced fracture of medial wall of right acetabulum, initial encounter for open fracture |
| S32.471D | Displaced fracture of medial wall of right acetabulum, subsequent encounter for fracture with routine healing |
| S32.471G | Displaced fracture of medial wall of right acetabulum, subsequent encounter for fracture with delayed healing |
| S32.471K | Displaced fracture of medial wall of right acetabulum, subsequent encounter for fracture with nonunion |
| S32.471S | Displaced fracture of medial wall of right acetabulum, sequela |
| S32.472 | Displaced fracture of medial wall of left acetabulum |
| S32.472A | Displaced fracture of medial wall of left acetabulum, initial encounter for closed fracture |
| S32.472B | Displaced fracture of medial wall of left acetabulum, initial encounter for open fracture |
| S32.472D | Displaced fracture of medial wall of left acetabulum, subsequent encounter for fracture with routine healing |
| S32.472G | Displaced fracture of medial wall of left acetabulum, subsequent encounter for fracture with delayed healing |
| S32.472K | Displaced fracture of medial wall of left acetabulum, subsequent encounter for fracture with nonunion |
| S32.472S | Displaced fracture of medial wall of left acetabulum, sequela |
| S32.473 | Displaced fracture of medial wall of unspecified acetabulum |
| S32.473A | Displaced fracture of medial wall of unspecified acetabulum, initial encounter for closed fracture |
| S32.473B | Displaced fracture of medial wall of unspecified acetabulum, initial encounter for open fracture |
| S32.473D | Displaced fracture of medial wall of unspecified acetabulum, subsequent encounter for fracture with routine healing |
| S32.473G | Displaced fracture of medial wall of unspecified acetabulum, subsequent encounter for fracture with delayed healing |
| S32.473K | Displaced fracture of medial wall of unspecified acetabulum, subsequent encounter for fracture with nonunion |
| S32.473S | Displaced fracture of medial wall of unspecified acetabulum, sequela |
| S32.474 | Nondisplaced fracture of medial wall of right acetabulum |
| S32.474A | Nondisplaced fracture of medial wall of right acetabulum, initial encounter for closed fracture |
| S32.474B | Nondisplaced fracture of medial wall of right acetabulum, initial encounter for open fracture |
| S32.474D | Nondisplaced fracture of medial wall of right acetabulum, subsequent encounter for fracture with routine healing |
| S32.474G | Nondisplaced fracture of medial wall of right acetabulum, subsequent encounter for fracture with delayed healing |
| S32.474K | Nondisplaced fracture of medial wall of right acetabulum, subsequent encounter for fracture with nonunion |
| S32.474S | Nondisplaced fracture of medial wall of right acetabulum, sequela |
| S32.475 | Nondisplaced fracture of medial wall of left acetabulum |
| S32.475A | Nondisplaced fracture of medial wall of left acetabulum, initial encounter for closed fracture |
| S32.475B | Nondisplaced fracture of medial wall of left acetabulum, initial encounter for open fracture |
| S32.475D | Nondisplaced fracture of medial wall of left acetabulum, subsequent encounter for fracture with routine healing |
| S32.475G | Nondisplaced fracture of medial wall of left acetabulum, subsequent encounter for fracture with delayed healing |
| S32.475K | Nondisplaced fracture of medial wall of left acetabulum, subsequent encounter for fracture with nonunion |
| S32.475S | Nondisplaced fracture of medial wall of left acetabulum, sequela |
| S32.476 | Nondisplaced fracture of medial wall of unspecified acetabulum |
| S32.476A | Nondisplaced fracture of medial wall of unspecified acetabulum, initial encounter for closed fracture |
| S32.476B | Nondisplaced fracture of medial wall of unspecified acetabulum, initial encounter for open fracture |
| S32.476D | Nondisplaced fracture of medial wall of unspecified acetabulum, subsequent encounter for fracture with routine healing |
| S32.476G | Nondisplaced fracture of medial wall of unspecified acetabulum, subsequent encounter for fracture with delayed healing |
| S32.476K | Nondisplaced fracture of medial wall of unspecified acetabulum, subsequent encounter for fracture with nonunion |
| S32.476S | Nondisplaced fracture of medial wall of unspecified acetabulum, sequela |
| S32.48 | Dome fracture of acetabulum |
| S32.481 | Displaced dome fracture of right acetabulum |
| S32.481A | Displaced dome fracture of right acetabulum, initial encounter for closed fracture |
| S32.481B | Displaced dome fracture of right acetabulum, initial encounter for open fracture |
| S32.481D | Displaced dome fracture of right acetabulum, subsequent encounter for fracture with routine healing |
| S32.481G | Displaced dome fracture of right acetabulum, subsequent encounter for fracture with delayed healing |
| S32.481K | Displaced dome fracture of right acetabulum, subsequent encounter for fracture with nonunion |
| S32.481S | Displaced dome fracture of right acetabulum, sequela |
| S32.482 | Displaced dome fracture of left acetabulum |
| S32.482A | Displaced dome fracture of left acetabulum, initial encounter for closed fracture |
| S32.482B | Displaced dome fracture of left acetabulum, initial encounter for open fracture |
| S32.482D | Displaced dome fracture of left acetabulum, subsequent encounter for fracture with routine healing |
| S32.482G | Displaced dome fracture of left acetabulum, subsequent encounter for fracture with delayed healing |
| S32.482K | Displaced dome fracture of left acetabulum, subsequent encounter for fracture with nonunion |
| S32.482S | Displaced dome fracture of left acetabulum, sequela |
| S32.483 | Displaced dome fracture of unspecified acetabulum |
| S32.483A | Displaced dome fracture of unspecified acetabulum, initial encounter for closed fracture |
| S32.483B | Displaced dome fracture of unspecified acetabulum, initial encounter for open fracture |
| S32.483D | Displaced dome fracture of unspecified acetabulum, subsequent encounter for fracture with routine healing |
| S32.483G | Displaced dome fracture of unspecified acetabulum, subsequent encounter for fracture with delayed healing |
| S32.483K | Displaced dome fracture of unspecified acetabulum, subsequent encounter for fracture with nonunion |
| S32.483S | Displaced dome fracture of unspecified acetabulum, sequela |
| S32.484 | Nondisplaced dome fracture of right acetabulum |
| S32.484A | Nondisplaced dome fracture of right acetabulum, initial encounter for closed fracture |
| S32.484B | Nondisplaced dome fracture of right acetabulum, initial encounter for open fracture |
| S32.484D | Nondisplaced dome fracture of right acetabulum, subsequent encounter for fracture with routine healing |
| S32.484G | Nondisplaced dome fracture of right acetabulum, subsequent encounter for fracture with delayed healing |
| S32.484K | Nondisplaced dome fracture of right acetabulum, subsequent encounter for fracture with nonunion |
| S32.484S | Nondisplaced dome fracture of right acetabulum, sequela |
| S32.485 | Nondisplaced dome fracture of left acetabulum |
| S32.485A | Nondisplaced dome fracture of left acetabulum, initial encounter for closed fracture |
| S32.485B | Nondisplaced dome fracture of left acetabulum, initial encounter for open fracture |
| S32.485D | Nondisplaced dome fracture of left acetabulum, subsequent encounter for fracture with routine healing |
| S32.485G | Nondisplaced dome fracture of left acetabulum, subsequent encounter for fracture with delayed healing |
| S32.485K | Nondisplaced dome fracture of left acetabulum, subsequent encounter for fracture with nonunion |
| S32.485S | Nondisplaced dome fracture of left acetabulum, sequela |
| S32.486 | Nondisplaced dome fracture of unspecified acetabulum |
| S32.486A | Nondisplaced dome fracture of unspecified acetabulum, initial encounter for closed fracture |
| S32.486B | Nondisplaced dome fracture of unspecified acetabulum, initial encounter for open fracture |
| S32.486D | Nondisplaced dome fracture of unspecified acetabulum, subsequent encounter for fracture with routine healing |
| S32.486G | Nondisplaced dome fracture of unspecified acetabulum, subsequent encounter for fracture with delayed healing |
| S32.486K | Nondisplaced dome fracture of unspecified acetabulum, subsequent encounter for fracture with nonunion |
| S32.486S | Nondisplaced dome fracture of unspecified acetabulum, sequela |
| S32.49 | Other specified fracture of acetabulum |
| S32.491 | Other specified fracture of right acetabulum |
| S32.491A | Other specified fracture of right acetabulum, initial encounter for closed fracture |
| S32.491B | Other specified fracture of right acetabulum, initial encounter for open fracture |
| S32.491D | Other specified fracture of right acetabulum, subsequent encounter for fracture with routine healing |
| S32.491G | Other specified fracture of right acetabulum, subsequent encounter for fracture with delayed healing |
| S32.491K | Other specified fracture of right acetabulum, subsequent encounter for fracture with nonunion |
| S32.491S | Other specified fracture of right acetabulum, sequela |
| S32.492 | Other specified fracture of left acetabulum |
| S32.492A | Other specified fracture of left acetabulum, initial encounter for closed fracture |
| S32.492B | Other specified fracture of left acetabulum, initial encounter for open fracture |
| S32.492D | Other specified fracture of left acetabulum, subsequent encounter for fracture with routine healing |
| S32.492G | Other specified fracture of left acetabulum, subsequent encounter for fracture with delayed healing |
| S32.492K | Other specified fracture of left acetabulum, subsequent encounter for fracture with nonunion |
| S32.492S | Other specified fracture of left acetabulum, sequela |
| S32.499 | Other specified fracture of unspecified acetabulum |
| S32.499A | Other specified fracture of unspecified acetabulum, initial encounter for closed fracture |
| S32.499B | Other specified fracture of unspecified acetabulum, initial encounter for open fracture |
| S32.499D | Other specified fracture of unspecified acetabulum, subsequent encounter for fracture with routine healing |
| S32.499G | Other specified fracture of unspecified acetabulum, subsequent encounter for fracture with delayed healing |
| S32.499K | Other specified fracture of unspecified acetabulum, subsequent encounter for fracture with nonunion |
| S32.499S | Other specified fracture of unspecified acetabulum, sequela |
| S32.5 | Fracture of pubis |
| S32.50 | Unspecified fracture of pubis |
| S32.501 | Unspecified fracture of right pubis |
| S32.501A | Unspecified fracture of right pubis, initial encounter for closed fracture |
| S32.501B | Unspecified fracture of right pubis, initial encounter for open fracture |
| S32.501D | Unspecified fracture of right pubis, subsequent encounter for fracture with routine healing |
| S32.501G | Unspecified fracture of right pubis, subsequent encounter for fracture with delayed healing |
| S32.501K | Unspecified fracture of right pubis, subsequent encounter for fracture with nonunion |
| S32.501S | Unspecified fracture of right pubis, sequela |
| S32.502 | Unspecified fracture of left pubis |
| S32.502A | Unspecified fracture of left pubis, initial encounter for closed fracture |
| S32.502B | Unspecified fracture of left pubis, initial encounter for open fracture |
| S32.502D | Unspecified fracture of left pubis, subsequent encounter for fracture with routine healing |
| S32.502G | Unspecified fracture of left pubis, subsequent encounter for fracture with delayed healing |
| S32.502K | Unspecified fracture of left pubis, subsequent encounter for fracture with nonunion |
| S32.502S | Unspecified fracture of left pubis, sequela |
| S32.509 | Unspecified fracture of unspecified pubis |
| S32.509A | Unspecified fracture of unspecified pubis, initial encounter for closed fracture |
| S32.509B | Unspecified fracture of unspecified pubis, initial encounter for open fracture |
| S32.509D | Unspecified fracture of unspecified pubis, subsequent encounter for fracture with routine healing |
| S32.509G | Unspecified fracture of unspecified pubis, subsequent encounter for fracture with delayed healing |
| S32.509K | Unspecified fracture of unspecified pubis, subsequent encounter for fracture with nonunion |
| S32.509S | Unspecified fracture of unspecified pubis, sequela |
| S32.51 | Fracture of superior rim of pubis |
| S32.511 | Fracture of superior rim of right pubis |
| S32.511A | Fracture of superior rim of right pubis, initial encounter for closed fracture |
| S32.511B | Fracture of superior rim of right pubis, initial encounter for open fracture |
| S32.511D | Fracture of superior rim of right pubis, subsequent encounter for fracture with routine healing |
| S32.511G | Fracture of superior rim of right pubis, subsequent encounter for fracture with delayed healing |
| S32.511K | Fracture of superior rim of right pubis, subsequent encounter for fracture with nonunion |
| S32.511S | Fracture of superior rim of right pubis, sequela |
| S32.512 | Fracture of superior rim of left pubis |
| S32.512A | Fracture of superior rim of left pubis, initial encounter for closed fracture |
| S32.512B | Fracture of superior rim of left pubis, initial encounter for open fracture |
| S32.512D | Fracture of superior rim of left pubis, subsequent encounter for fracture with routine healing |
| S32.512G | Fracture of superior rim of left pubis, subsequent encounter for fracture with delayed healing |
| S32.512K | Fracture of superior rim of left pubis, subsequent encounter for fracture with nonunion |
| S32.512S | Fracture of superior rim of left pubis, sequela |
| S32.519 | Fracture of superior rim of unspecified pubis |
| S32.519A | Fracture of superior rim of unspecified pubis, initial encounter for closed fracture |
| S32.519B | Fracture of superior rim of unspecified pubis, initial encounter for open fracture |
| S32.519D | Fracture of superior rim of unspecified pubis, subsequent encounter for fracture with routine healing |
| S32.519G | Fracture of superior rim of unspecified pubis, subsequent encounter for fracture with delayed healing |
| S32.519K | Fracture of superior rim of unspecified pubis, subsequent encounter for fracture with nonunion |
| S32.519S | Fracture of superior rim of unspecified pubis, sequela |
| S32.59 | Other specified fracture of pubis |
| S32.591 | Other specified fracture of right pubis |
| S32.591A | Other specified fracture of right pubis, initial encounter for closed fracture |
| S32.591B | Other specified fracture of right pubis, initial encounter for open fracture |
| S32.591D | Other specified fracture of right pubis, subsequent encounter for fracture with routine healing |
| S32.591G | Other specified fracture of right pubis, subsequent encounter for fracture with delayed healing |
| S32.591K | Other specified fracture of right pubis, subsequent encounter for fracture with nonunion |
| S32.591S | Other specified fracture of right pubis, sequela |
| S32.592 | Other specified fracture of left pubis |
| S32.592A | Other specified fracture of left pubis, initial encounter for closed fracture |
| S32.592B | Other specified fracture of left pubis, initial encounter for open fracture |
| S32.592D | Other specified fracture of left pubis, subsequent encounter for fracture with routine healing |
| S32.592G | Other specified fracture of left pubis, subsequent encounter for fracture with delayed healing |
| S32.592K | Other specified fracture of left pubis, subsequent encounter for fracture with nonunion |
| S32.592S | Other specified fracture of left pubis, sequela |
| S32.599 | Other specified fracture of unspecified pubis |
| S32.599A | Other specified fracture of unspecified pubis, initial encounter for closed fracture |
| S32.599B | Other specified fracture of unspecified pubis, initial encounter for open fracture |
| S32.599D | Other specified fracture of unspecified pubis, subsequent encounter for fracture with routine healing |
| S32.599G | Other specified fracture of unspecified pubis, subsequent encounter for fracture with delayed healing |
| S32.599K | Other specified fracture of unspecified pubis, subsequent encounter for fracture with nonunion |
| S32.599S | Other specified fracture of unspecified pubis, sequela |
| S32.6 | Fracture of ischium |
| S32.60 | Unspecified fracture of ischium |
| S32.601 | Unspecified fracture of right ischium |
| S32.601A | Unspecified fracture of right ischium, initial encounter for closed fracture |
| S32.601B | Unspecified fracture of right ischium, initial encounter for open fracture |
| S32.601D | Unspecified fracture of right ischium, subsequent encounter for fracture with routine healing |
| S32.601G | Unspecified fracture of right ischium, subsequent encounter for fracture with delayed healing |
| S32.601K | Unspecified fracture of right ischium, subsequent encounter for fracture with nonunion |
| S32.601S | Unspecified fracture of right ischium, sequela |
| S32.602 | Unspecified fracture of left ischium |
| S32.602A | Unspecified fracture of left ischium, initial encounter for closed fracture |
| S32.602B | Unspecified fracture of left ischium, initial encounter for open fracture |
| S32.602D | Unspecified fracture of left ischium, subsequent encounter for fracture with routine healing |
| S32.602G | Unspecified fracture of left ischium, subsequent encounter for fracture with delayed healing |
| S32.602K | Unspecified fracture of left ischium, subsequent encounter for fracture with nonunion |
| S32.602S | Unspecified fracture of left ischium, sequela |
| S32.609 | Unspecified fracture of unspecified ischium |
| S32.609A | Unspecified fracture of unspecified ischium, initial encounter for closed fracture |
| S32.609B | Unspecified fracture of unspecified ischium, initial encounter for open fracture |
| S32.609D | Unspecified fracture of unspecified ischium, subsequent encounter for fracture with routine healing |
| S32.609G | Unspecified fracture of unspecified ischium, subsequent encounter for fracture with delayed healing |
| S32.609K | Unspecified fracture of unspecified ischium, subsequent encounter for fracture with nonunion |
| S32.609S | Unspecified fracture of unspecified ischium, sequela |
| S32.61 | Avulsion fracture of ischium |
| S32.611 | Displaced avulsion fracture of right ischium |
| S32.611A | Displaced avulsion fracture of right ischium, initial encounter for closed fracture |
| S32.611B | Displaced avulsion fracture of right ischium, initial encounter for open fracture |
| S32.611D | Displaced avulsion fracture of right ischium, subsequent encounter for fracture with routine healing |
| S32.611G | Displaced avulsion fracture of right ischium, subsequent encounter for fracture with delayed healing |
| S32.611K | Displaced avulsion fracture of right ischium, subsequent encounter for fracture with nonunion |
| S32.611S | Displaced avulsion fracture of right ischium, sequela |
| S32.612 | Displaced avulsion fracture of left ischium |
| S32.612A | Displaced avulsion fracture of left ischium, initial encounter for closed fracture |
| S32.612B | Displaced avulsion fracture of left ischium, initial encounter for open fracture |
| S32.612D | Displaced avulsion fracture of left ischium, subsequent encounter for fracture with routine healing |
| S32.612G | Displaced avulsion fracture of left ischium, subsequent encounter for fracture with delayed healing |
| S32.612K | Displaced avulsion fracture of left ischium, subsequent encounter for fracture with nonunion |
| S32.612S | Displaced avulsion fracture of left ischium, sequela |
| S32.613 | Displaced avulsion fracture of unspecified ischium |
| S32.613A | Displaced avulsion fracture of unspecified ischium, initial encounter for closed fracture |
| S32.613B | Displaced avulsion fracture of unspecified ischium, initial encounter for open fracture |
| S32.613D | Displaced avulsion fracture of unspecified ischium, subsequent encounter for fracture with routine healing |
| S32.613G | Displaced avulsion fracture of unspecified ischium, subsequent encounter for fracture with delayed healing |
| S32.613K | Displaced avulsion fracture of unspecified ischium, subsequent encounter for fracture with nonunion |
| S32.613S | Displaced avulsion fracture of unspecified ischium, sequela |
| S32.614 | Nondisplaced avulsion fracture of right ischium |
| S32.614A | Nondisplaced avulsion fracture of right ischium, initial encounter for closed fracture |
| S32.614B | Nondisplaced avulsion fracture of right ischium, initial encounter for open fracture |
| S32.614D | Nondisplaced avulsion fracture of right ischium, subsequent encounter for fracture with routine healing |
| S32.614G | Nondisplaced avulsion fracture of right ischium, subsequent encounter for fracture with delayed healing |
| S32.614K | Nondisplaced avulsion fracture of right ischium, subsequent encounter for fracture with nonunion |
| S32.614S | Nondisplaced avulsion fracture of right ischium, sequela |
| S32.615 | Nondisplaced avulsion fracture of left ischium |
| S32.615A | Nondisplaced avulsion fracture of left ischium, initial encounter for closed fracture |
| S32.615B | Nondisplaced avulsion fracture of left ischium, initial encounter for open fracture |
| S32.615D | Nondisplaced avulsion fracture of left ischium, subsequent encounter for fracture with routine healing |
| S32.615G | Nondisplaced avulsion fracture of left ischium, subsequent encounter for fracture with delayed healing |
| S32.615K | Nondisplaced avulsion fracture of left ischium, subsequent encounter for fracture with nonunion |
| S32.615S | Nondisplaced avulsion fracture of left ischium, sequela |
| S32.616 | Nondisplaced avulsion fracture of unspecified ischium |
| S32.616A | Nondisplaced avulsion fracture of unspecified ischium, initial encounter for closed fracture |
| S32.616B | Nondisplaced avulsion fracture of unspecified ischium, initial encounter for open fracture |
| S32.616D | Nondisplaced avulsion fracture of unspecified ischium, subsequent encounter for fracture with routine healing |
| S32.616G | Nondisplaced avulsion fracture of unspecified ischium, subsequent encounter for fracture with delayed healing |
| S32.616K | Nondisplaced avulsion fracture of unspecified ischium, subsequent encounter for fracture with nonunion |
| S32.616S | Nondisplaced avulsion fracture of unspecified ischium, sequela |
| S32.69 | Other specified fracture of ischium |
| S32.691 | Other specified fracture of right ischium |
| S32.691A | Other specified fracture of right ischium, initial encounter for closed fracture |
| S32.691B | Other specified fracture of right ischium, initial encounter for open fracture |
| S32.691D | Other specified fracture of right ischium, subsequent encounter for fracture with routine healing |
| S32.691G | Other specified fracture of right ischium, subsequent encounter for fracture with delayed healing |
| S32.691K | Other specified fracture of right ischium, subsequent encounter for fracture with nonunion |
| S32.691S | Other specified fracture of right ischium, sequela |
| S32.692 | Other specified fracture of left ischium |
| S32.692A | Other specified fracture of left ischium, initial encounter for closed fracture |
| S32.692B | Other specified fracture of left ischium, initial encounter for open fracture |
| S32.692D | Other specified fracture of left ischium, subsequent encounter for fracture with routine healing |
| S32.692G | Other specified fracture of left ischium, subsequent encounter for fracture with delayed healing |
| S32.692K | Other specified fracture of left ischium, subsequent encounter for fracture with nonunion |
| S32.692S | Other specified fracture of left ischium, sequela |
| S32.699 | Other specified fracture of unspecified ischium |
| S32.699A | Other specified fracture of unspecified ischium, initial encounter for closed fracture |
| S32.699B | Other specified fracture of unspecified ischium, initial encounter for open fracture |
| S32.699D | Other specified fracture of unspecified ischium, subsequent encounter for fracture with routine healing |
| S32.699G | Other specified fracture of unspecified ischium, subsequent encounter for fracture with delayed healing |
| S32.699K | Other specified fracture of unspecified ischium, subsequent encounter for fracture with nonunion |
| S32.699S | Other specified fracture of unspecified ischium, sequela |
| S32.8 | Fracture of other parts of pelvis |
| S32.81 | Multiple fractures of pelvis with disruption of pelvic ring |
| S32.810 | Multiple fractures of pelvis with stable disruption of pelvic ring |
| S32.810A | Multiple fractures of pelvis with stable disruption of pelvic ring, initial encounter for closed fracture |
| S32.810B | Multiple fractures of pelvis with stable disruption of pelvic ring, initial encounter for open fracture |
| S32.810D | Multiple fractures of pelvis with stable disruption of pelvic ring, subsequent encounter for fracture with routine healing |
| S32.810G | Multiple fractures of pelvis with stable disruption of pelvic ring, subsequent encounter for fracture with delayed healing |
| S32.810K | Multiple fractures of pelvis with stable disruption of pelvic ring, subsequent encounter for fracture with nonunion |
| S32.810S | Multiple fractures of pelvis with stable disruption of pelvic ring, sequela |
| S32.811 | Multiple fractures of pelvis with unstable disruption of pelvic ring |
| S32.811A | Multiple fractures of pelvis with unstable disruption of pelvic ring, initial encounter for closed fracture |
| S32.811B | Multiple fractures of pelvis with unstable disruption of pelvic ring, initial encounter for open fracture |
| S32.811D | Multiple fractures of pelvis with unstable disruption of pelvic ring, subsequent encounter for fracture with routine healing |
| S32.811G | Multiple fractures of pelvis with unstable disruption of pelvic ring, subsequent encounter for fracture with delayed healing |
| S32.811K | Multiple fractures of pelvis with unstable disruption of pelvic ring, subsequent encounter for fracture with nonunion |
| S32.811S | Multiple fractures of pelvis with unstable disruption of pelvic ring, sequela |
| S32.82 | Multiple fractures of pelvis without disruption of pelvic ring |
| S32.82XA | Multiple fractures of pelvis without disruption of pelvic ring, initial encounter for closed fracture |
| S32.82XB | Multiple fractures of pelvis without disruption of pelvic ring, initial encounter for open fracture |
| S32.82XD | Multiple fractures of pelvis without disruption of pelvic ring, subsequent encounter for fracture with routine healing |
| S32.82XG | Multiple fractures of pelvis without disruption of pelvic ring, subsequent encounter for fracture with delayed healing |
| S32.82XK | Multiple fractures of pelvis without disruption of pelvic ring, subsequent encounter for fracture with nonunion |
| S32.82XS | Multiple fractures of pelvis without disruption of pelvic ring, sequela |
| S32.89 | Fracture of other parts of pelvis |
| S32.89XA | Fracture of other parts of pelvis, initial encounter for closed fracture |
| S32.89XB | Fracture of other parts of pelvis, initial encounter for open fracture |
| S32.89XD | Fracture of other parts of pelvis, subsequent encounter for fracture with routine healing |
| S32.89XG | Fracture of other parts of pelvis, subsequent encounter for fracture with delayed healing |
| S32.89XK | Fracture of other parts of pelvis, subsequent encounter for fracture with nonunion |
| S32.89XS | Fracture of other parts of pelvis, sequela |
| S32.9 | Fracture of unspecified parts of lumbosacral spine and pelvis |
| S32.9XXA | Fracture of unspecified parts of lumbosacral spine and pelvis, initial encounter for closed fracture |
| S32.9XXB | Fracture of unspecified parts of lumbosacral spine and pelvis, initial encounter for open fracture |
| S32.9XXD | Fracture of unspecified parts of lumbosacral spine and pelvis, subsequent encounter for fracture with routine healing |
| S32.9XXG | Fracture of unspecified parts of lumbosacral spine and pelvis, subsequent encounter for fracture with delayed healing |
| S32.9XXK | Fracture of unspecified parts of lumbosacral spine and pelvis, subsequent encounter for fracture with nonunion |
| S32.9XXS | Fracture of unspecified parts of lumbosacral spine and pelvis, sequela |
| S33 | Dislocation and sprain of joints and ligaments of lumbar spine and pelvis |
| S33.0 | Traumatic rupture of lumbar intervertebral disc |
| S33.0XXA | Traumatic rupture of lumbar intervertebral disc, initial encounter |
| S33.0XXD | Traumatic rupture of lumbar intervertebral disc, subsequent encounter |
| S33.0XXS | Traumatic rupture of lumbar intervertebral disc, sequela |
| S33.1 | Subluxation and dislocation of lumbar vertebra |
| S33.10 | Subluxation and dislocation of unspecified lumbar vertebra |
| S33.100 | Subluxation of unspecified lumbar vertebra |
| S33.100A | Subluxation of unspecified lumbar vertebra, initial encounter |
| S33.100D | Subluxation of unspecified lumbar vertebra, subsequent encounter |
| S33.100S | Subluxation of unspecified lumbar vertebra, sequela |
| S33.101 | Dislocation of unspecified lumbar vertebra |
| S33.101A | Dislocation of unspecified lumbar vertebra, initial encounter |
| S33.101D | Dislocation of unspecified lumbar vertebra, subsequent encounter |
| S33.101S | Dislocation of unspecified lumbar vertebra, sequela |
| S33.11 | Subluxation and dislocation of L1/L2 lumbar vertebra |
| S33.110 | Subluxation of L1/L2 lumbar vertebra |
| S33.110A | Subluxation of L1/L2 lumbar vertebra, initial encounter |
| S33.110D | Subluxation of L1/L2 lumbar vertebra, subsequent encounter |
| S33.110S | Subluxation of L1/L2 lumbar vertebra, sequela |
| S33.111 | Dislocation of L1/L2 lumbar vertebra |
| S33.111A | Dislocation of L1/L2 lumbar vertebra, initial encounter |
| S33.111D | Dislocation of L1/L2 lumbar vertebra, subsequent encounter |
| S33.111S | Dislocation of L1/L2 lumbar vertebra, sequela |
| S33.12 | Subluxation and dislocation of L2/L3 lumbar vertebra |
| S33.120 | Subluxation of L2/L3 lumbar vertebra |
| S33.120A | Subluxation of L2/L3 lumbar vertebra, initial encounter |
| S33.120D | Subluxation of L2/L3 lumbar vertebra, subsequent encounter |
| S33.120S | Subluxation of L2/L3 lumbar vertebra, sequela |
| S33.121 | Dislocation of L2/L3 lumbar vertebra |
| S33.121A | Dislocation of L2/L3 lumbar vertebra, initial encounter |
| S33.121D | Dislocation of L2/L3 lumbar vertebra, subsequent encounter |
| S33.121S | Dislocation of L2/L3 lumbar vertebra, sequela |
| S33.13 | Subluxation and dislocation of L3/L4 lumbar vertebra |
| S33.130 | Subluxation of L3/L4 lumbar vertebra |
| S33.130A | Subluxation of L3/L4 lumbar vertebra, initial encounter |
| S33.130D | Subluxation of L3/L4 lumbar vertebra, subsequent encounter |
| S33.130S | Subluxation of L3/L4 lumbar vertebra, sequela |
| S33.131 | Dislocation of L3/L4 lumbar vertebra |
| S33.131A | Dislocation of L3/L4 lumbar vertebra, initial encounter |
| S33.131D | Dislocation of L3/L4 lumbar vertebra, subsequent encounter |
| S33.131S | Dislocation of L3/L4 lumbar vertebra, sequela |
| S33.14 | Subluxation and dislocation of L4/L5 lumbar vertebra |
| S33.140 | Subluxation of L4/L5 lumbar vertebra |
| S33.140A | Subluxation of L4/L5 lumbar vertebra, initial encounter |
| S33.140D | Subluxation of L4/L5 lumbar vertebra, subsequent encounter |
| S33.140S | Subluxation of L4/L5 lumbar vertebra, sequela |
| S33.141 | Dislocation of L4/L5 lumbar vertebra |
| S33.141A | Dislocation of L4/L5 lumbar vertebra, initial encounter |
| S33.141D | Dislocation of L4/L5 lumbar vertebra, subsequent encounter |
| S33.141S | Dislocation of L4/L5 lumbar vertebra, sequela |
| S33.2 | Dislocation of sacroiliac and sacrococcygeal joint |
| S33.2XXA | Dislocation of sacroiliac and sacrococcygeal joint, initial encounter |
| S33.2XXD | Dislocation of sacroiliac and sacrococcygeal joint, subsequent encounter |
| S33.2XXS | Dislocation of sacroiliac and sacrococcygeal joint, sequela |
| S33.3 | Dislocation of other and unspecified parts of lumbar spine and pelvis |
| S33.30 | Dislocation of unspecified parts of lumbar spine and pelvis |
| S33.30XA | Dislocation of unspecified parts of lumbar spine and pelvis, initial encounter |
| S33.30XD | Dislocation of unspecified parts of lumbar spine and pelvis, subsequent encounter |
| S33.30XS | Dislocation of unspecified parts of lumbar spine and pelvis, sequela |
| S33.39 | Dislocation of other parts of lumbar spine and pelvis |
| S33.39XA | Dislocation of other parts of lumbar spine and pelvis, initial encounter |
| S33.39XD | Dislocation of other parts of lumbar spine and pelvis, subsequent encounter |
| S33.39XS | Dislocation of other parts of lumbar spine and pelvis, sequela |
| S33.4 | Traumatic rupture of symphysis pubis |
| S33.4XXA | Traumatic rupture of symphysis pubis, initial encounter |
| S33.4XXD | Traumatic rupture of symphysis pubis, subsequent encounter |
| S33.4XXS | Traumatic rupture of symphysis pubis, sequela |
| S33.5 | Sprain of ligaments of lumbar spine |
| S33.5XXA | Sprain of ligaments of lumbar spine, initial encounter |
| S33.5XXD | Sprain of ligaments of lumbar spine, subsequent encounter |
| S33.5XXS | Sprain of ligaments of lumbar spine, sequela |
| S33.6 | Sprain of sacroiliac joint |
| S33.6XXA | Sprain of sacroiliac joint, initial encounter |
| S33.6XXD | Sprain of sacroiliac joint, subsequent encounter |
| S33.6XXS | Sprain of sacroiliac joint, sequela |
| S33.8 | Sprain of other parts of lumbar spine and pelvis |
| S33.8XXA | Sprain of other parts of lumbar spine and pelvis, initial encounter |
| S33.8XXD | Sprain of other parts of lumbar spine and pelvis, subsequent encounter |
| S33.8XXS | Sprain of other parts of lumbar spine and pelvis, sequela |
| S33.9 | Sprain of unspecified parts of lumbar spine and pelvis |
| S33.9XXA | Sprain of unspecified parts of lumbar spine and pelvis, initial encounter |
| S33.9XXD | Sprain of unspecified parts of lumbar spine and pelvis, subsequent encounter |
| S33.9XXS | Sprain of unspecified parts of lumbar spine and pelvis, sequela |
| S34 | Injury of lumbar and sacral spinal cord and nerves at abdomen, lower back and pelvis level |
| S34.0 | Concussion and edema of lumbar and sacral spinal cord |
| S34.01 | Concussion and edema of lumbar spinal cord |
| S34.01XA | Concussion and edema of lumbar spinal cord, initial encounter |
| S34.01XD | Concussion and edema of lumbar spinal cord, subsequent encounter |
| S34.01XS | Concussion and edema of lumbar spinal cord, sequela |
| S34.02 | Concussion and edema of sacral spinal cord |
| S34.02XA | Concussion and edema of sacral spinal cord, initial encounter |
| S34.02XD | Concussion and edema of sacral spinal cord, subsequent encounter |
| S34.02XS | Concussion and edema of sacral spinal cord, sequela |
| S34.1 | Other and unspecified injury of lumbar and sacral spinal cord |
| S34.10 | Unspecified injury to lumbar spinal cord |
| S34.101 | Unspecified injury to L1 level of lumbar spinal cord |
| S34.101A | Unspecified injury to L1 level of lumbar spinal cord, initial encounter |
| S34.101D | Unspecified injury to L1 level of lumbar spinal cord, subsequent encounter |
| S34.101S | Unspecified injury to L1 level of lumbar spinal cord, sequela |
| S34.102 | Unspecified injury to L2 level of lumbar spinal cord |
| S34.102A | Unspecified injury to L2 level of lumbar spinal cord, initial encounter |
| S34.102D | Unspecified injury to L2 level of lumbar spinal cord, subsequent encounter |
| S34.102S | Unspecified injury to L2 level of lumbar spinal cord, sequela |
| S34.103 | Unspecified injury to L3 level of lumbar spinal cord |
| S34.103A | Unspecified injury to L3 level of lumbar spinal cord, initial encounter |
| S34.103D | Unspecified injury to L3 level of lumbar spinal cord, subsequent encounter |
| S34.103S | Unspecified injury to L3 level of lumbar spinal cord, sequela |
| S34.104 | Unspecified injury to L4 level of lumbar spinal cord |
| S34.104A | Unspecified injury to L4 level of lumbar spinal cord, initial encounter |
| S34.104D | Unspecified injury to L4 level of lumbar spinal cord, subsequent encounter |
| S34.104S | Unspecified injury to L4 level of lumbar spinal cord, sequela |
| S34.105 | Unspecified injury to L5 level of lumbar spinal cord |
| S34.105A | Unspecified injury to L5 level of lumbar spinal cord, initial encounter |
| S34.105D | Unspecified injury to L5 level of lumbar spinal cord, subsequent encounter |
| S34.105S | Unspecified injury to L5 level of lumbar spinal cord, sequela |
| S34.109 | Unspecified injury to unspecified level of lumbar spinal cord |
| S34.109A | Unspecified injury to unspecified level of lumbar spinal cord, initial encounter |
| S34.109D | Unspecified injury to unspecified level of lumbar spinal cord, subsequent encounter |
| S34.109S | Unspecified injury to unspecified level of lumbar spinal cord, sequela |
| S34.11 | Complete lesion of lumbar spinal cord |
| S34.111 | Complete lesion of L1 level of lumbar spinal cord |
| S34.111A | Complete lesion of L1 level of lumbar spinal cord, initial encounter |
| S34.111D | Complete lesion of L1 level of lumbar spinal cord, subsequent encounter |
| S34.111S | Complete lesion of L1 level of lumbar spinal cord, sequela |
| S34.112 | Complete lesion of L2 level of lumbar spinal cord |
| S34.112A | Complete lesion of L2 level of lumbar spinal cord, initial encounter |
| S34.112D | Complete lesion of L2 level of lumbar spinal cord, subsequent encounter |
| S34.112S | Complete lesion of L2 level of lumbar spinal cord, sequela |
| S34.113 | Complete lesion of L3 level of lumbar spinal cord |
| S34.113A | Complete lesion of L3 level of lumbar spinal cord, initial encounter |
| S34.113D | Complete lesion of L3 level of lumbar spinal cord, subsequent encounter |
| S34.113S | Complete lesion of L3 level of lumbar spinal cord, sequela |
| S34.114 | Complete lesion of L4 level of lumbar spinal cord |
| S34.114A | Complete lesion of L4 level of lumbar spinal cord, initial encounter |
| S34.114D | Complete lesion of L4 level of lumbar spinal cord, subsequent encounter |
| S34.114S | Complete lesion of L4 level of lumbar spinal cord, sequela |
| S34.115 | Complete lesion of L5 level of lumbar spinal cord |
| S34.115A | Complete lesion of L5 level of lumbar spinal cord, initial encounter |
| S34.115D | Complete lesion of L5 level of lumbar spinal cord, subsequent encounter |
| S34.115S | Complete lesion of L5 level of lumbar spinal cord, sequela |
| S34.119 | Complete lesion of unspecified level of lumbar spinal cord |
| S34.119A | Complete lesion of unspecified level of lumbar spinal cord, initial encounter |
| S34.119D | Complete lesion of unspecified level of lumbar spinal cord, subsequent encounter |
| S34.119S | Complete lesion of unspecified level of lumbar spinal cord, sequela |
| S34.12 | Incomplete lesion of lumbar spinal cord |
| S34.121 | Incomplete lesion of L1 level of lumbar spinal cord |
| S34.121A | Incomplete lesion of L1 level of lumbar spinal cord, initial encounter |
| S34.121D | Incomplete lesion of L1 level of lumbar spinal cord, subsequent encounter |
| S34.121S | Incomplete lesion of L1 level of lumbar spinal cord, sequela |
| S34.122 | Incomplete lesion of L2 level of lumbar spinal cord |
| S34.122A | Incomplete lesion of L2 level of lumbar spinal cord, initial encounter |
| S34.122D | Incomplete lesion of L2 level of lumbar spinal cord, subsequent encounter |
| S34.122S | Incomplete lesion of L2 level of lumbar spinal cord, sequela |
| S34.123 | Incomplete lesion of L3 level of lumbar spinal cord |
| S34.123A | Incomplete lesion of L3 level of lumbar spinal cord, initial encounter |
| S34.123D | Incomplete lesion of L3 level of lumbar spinal cord, subsequent encounter |
| S34.123S | Incomplete lesion of L3 level of lumbar spinal cord, sequela |
| S34.124 | Incomplete lesion of L4 level of lumbar spinal cord |
| S34.124A | Incomplete lesion of L4 level of lumbar spinal cord, initial encounter |
| S34.124D | Incomplete lesion of L4 level of lumbar spinal cord, subsequent encounter |
| S34.124S | Incomplete lesion of L4 level of lumbar spinal cord, sequela |
| S34.125 | Incomplete lesion of L5 level of lumbar spinal cord |
| S34.125A | Incomplete lesion of L5 level of lumbar spinal cord, initial encounter |
| S34.125D | Incomplete lesion of L5 level of lumbar spinal cord, subsequent encounter |
| S34.125S | Incomplete lesion of L5 level of lumbar spinal cord, sequela |
| S34.129 | Incomplete lesion of unspecified level of lumbar spinal cord |
| S34.129A | Incomplete lesion of unspecified level of lumbar spinal cord, initial encounter |
| S34.129D | Incomplete lesion of unspecified level of lumbar spinal cord, subsequent encounter |
| S34.129S | Incomplete lesion of unspecified level of lumbar spinal cord, sequela |
| S34.13 | Other and unspecified injury to sacral spinal cord |
| S34.131 | Complete lesion of sacral spinal cord |
| S34.131A | Complete lesion of sacral spinal cord, initial encounter |
| S34.131D | Complete lesion of sacral spinal cord, subsequent encounter |
| S34.131S | Complete lesion of sacral spinal cord, sequela |
| S34.132 | Incomplete lesion of sacral spinal cord |
| S34.132A | Incomplete lesion of sacral spinal cord, initial encounter |
| S34.132D | Incomplete lesion of sacral spinal cord, subsequent encounter |
| S34.132S | Incomplete lesion of sacral spinal cord, sequela |
| S34.139 | Unspecified injury to sacral spinal cord |
| S34.139A | Unspecified injury to sacral spinal cord, initial encounter |
| S34.139D | Unspecified injury to sacral spinal cord, subsequent encounter |
| S34.139S | Unspecified injury to sacral spinal cord, sequela |
| S34.2 | Injury of nerve root of lumbar and sacral spine |
| S34.21 | Injury of nerve root of lumbar spine |
| S34.21XA | Injury of nerve root of lumbar spine, initial encounter |
| S34.21XD | Injury of nerve root of lumbar spine, subsequent encounter |
| S34.21XS | Injury of nerve root of lumbar spine, sequela |
| S34.22 | Injury of nerve root of sacral spine |
| S34.22XA | Injury of nerve root of sacral spine, initial encounter |
| S34.22XD | Injury of nerve root of sacral spine, subsequent encounter |
| S34.22XS | Injury of nerve root of sacral spine, sequela |
| S34.3 | Injury of cauda equina |
| S34.3XXA | Injury of cauda equina, initial encounter |
| S34.3XXD | Injury of cauda equina, subsequent encounter |
| S34.3XXS | Injury of cauda equina, sequela |
| S34.4 | Injury of lumbosacral plexus |
| S34.4XXA | Injury of lumbosacral plexus, initial encounter |
| S34.4XXD | Injury of lumbosacral plexus, subsequent encounter |
| S34.4XXS | Injury of lumbosacral plexus, sequela |
| S34.5 | Injury of lumbar, sacral and pelvic sympathetic nerves |
| S34.5XXA | Injury of lumbar, sacral and pelvic sympathetic nerves, initial encounter |
| S34.5XXD | Injury of lumbar, sacral and pelvic sympathetic nerves, subsequent encounter |
| S34.5XXS | Injury of lumbar, sacral and pelvic sympathetic nerves, sequela |
| S34.6 | Injury of peripheral nerve(s) at abdomen, lower back and pelvis level |
| S34.6XXA | Injury of peripheral nerve(s) at abdomen, lower back and pelvis level, initial encounter |
| S34.6XXD | Injury of peripheral nerve(s) at abdomen, lower back and pelvis level, subsequent encounter |
| S34.6XXS | Injury of peripheral nerve(s) at abdomen, lower back and pelvis level, sequela |
| S34.8 | Injury of other nerves at abdomen, lower back and pelvis level |
| S34.8XXA | Injury of other nerves at abdomen, lower back and pelvis level, initial encounter |
| S34.8XXD | Injury of other nerves at abdomen, lower back and pelvis level, subsequent encounter |
| S34.8XXS | Injury of other nerves at abdomen, lower back and pelvis level, sequela |
| S34.9 | Injury of unspecified nerves at abdomen, lower back and pelvis level |
| S34.9XXA | Injury of unspecified nerves at abdomen, lower back and pelvis level, initial encounter |
| S34.9XXD | Injury of unspecified nerves at abdomen, lower back and pelvis level, subsequent encounter |
| S34.9XXS | Injury of unspecified nerves at abdomen, lower back and pelvis level, sequela |
| S38 | Crushing injury and traumatic amputation of abdomen, lower back, pelvis and external genitals |
| S38.0 | Crushing injury of external genital organs |
| S38.00 | Crushing injury of unspecified external genital organs |
| S38.001 | Crushing injury of unspecified external genital organs, male |
| S38.001A | Crushing injury of unspecified external genital organs, male, initial encounter |
| S38.001D | Crushing injury of unspecified external genital organs, male, subsequent encounter |
| S38.001S | Crushing injury of unspecified external genital organs, male, sequela |
| S38.002 | Crushing injury of unspecified external genital organs, female |
| S38.002A | Crushing injury of unspecified external genital organs, female, initial encounter |
| S38.002D | Crushing injury of unspecified external genital organs, female, subsequent encounter |
| S38.002S | Crushing injury of unspecified external genital organs, female, sequela |
| S38.01 | Crushing injury of penis |
| S38.01XA | Crushing injury of penis, initial encounter |
| S38.01XD | Crushing injury of penis, subsequent encounter |
| S38.01XS | Crushing injury of penis, sequela |
| S38.02 | Crushing injury of scrotum and testis |
| S38.02XA | Crushing injury of scrotum and testis, initial encounter |
| S38.02XD | Crushing injury of scrotum and testis, subsequent encounter |
| S38.02XS | Crushing injury of scrotum and testis, sequela |
| S38.03 | Crushing injury of vulva |
| S38.03XA | Crushing injury of vulva, initial encounter |
| S38.03XD | Crushing injury of vulva, subsequent encounter |
| S38.03XS | Crushing injury of vulva, sequela |
| S38.1 | Crushing injury of abdomen, lower back, and pelvis |
| S38.1XXA | Crushing injury of abdomen, lower back, and pelvis, initial encounter |
| S38.1XXD | Crushing injury of abdomen, lower back, and pelvis, subsequent encounter |
| S38.1XXS | Crushing injury of abdomen, lower back, and pelvis, sequela |
| S38.2 | Traumatic amputation of external genital organs |
| S38.21 | Traumatic amputation of female external genital organs |
| S38.211 | Complete traumatic amputation of female external genital organs |
| S38.211A | Complete traumatic amputation of female external genital organs, initial encounter |
| S38.211D | Complete traumatic amputation of female external genital organs, subsequent encounter |
| S38.211S | Complete traumatic amputation of female external genital organs, sequela |
| S38.212 | Partial traumatic amputation of female external genital organs |
| S38.212A | Partial traumatic amputation of female external genital organs, initial encounter |
| S38.212D | Partial traumatic amputation of female external genital organs, subsequent encounter |
| S38.212S | Partial traumatic amputation of female external genital organs, sequela |
| S38.22 | Traumatic amputation of penis |
| S38.221 | Complete traumatic amputation of penis |
| S38.221A | Complete traumatic amputation of penis, initial encounter |
| S38.221D | Complete traumatic amputation of penis, subsequent encounter |
| S38.221S | Complete traumatic amputation of penis, sequela |
| S38.222 | Partial traumatic amputation of penis |
| S38.222A | Partial traumatic amputation of penis, initial encounter |
| S38.222D | Partial traumatic amputation of penis, subsequent encounter |
| S38.222S | Partial traumatic amputation of penis, sequela |
| S38.23 | Traumatic amputation of scrotum and testis |
| S38.231 | Complete traumatic amputation of scrotum and testis |
| S38.231A | Complete traumatic amputation of scrotum and testis, initial encounter |
| S38.231D | Complete traumatic amputation of scrotum and testis, subsequent encounter |
| S38.231S | Complete traumatic amputation of scrotum and testis, sequela |
| S38.232 | Partial traumatic amputation of scrotum and testis |
| S38.232A | Partial traumatic amputation of scrotum and testis, initial encounter |
| S38.232D | Partial traumatic amputation of scrotum and testis, subsequent encounter |
| S38.232S | Partial traumatic amputation of scrotum and testis, sequela |
| S38.3 | Transection (partial) of abdomen |
| S38.3XXA | Transection (partial) of abdomen, initial encounter |
| S38.3XXD | Transection (partial) of abdomen, subsequent encounter |
| S38.3XXS | Transection (partial) of abdomen, sequela |
| S42.15 | Fracture of neck of scapula |
| S42.151 | Displaced fracture of neck of scapula, right shoulder |
| S42.151A | Displaced fracture of neck of scapula, right shoulder, initial encounter for closed fracture |
| S42.151B | Displaced fracture of neck of scapula, right shoulder, initial encounter for open fracture |
| S42.151D | Displaced fracture of neck of scapula, right shoulder, subsequent encounter for fracture with routine healing |
| S42.151G | Displaced fracture of neck of scapula, right shoulder, subsequent encounter for fracture with delayed healing |
| S42.151K | Displaced fracture of neck of scapula, right shoulder, subsequent encounter for fracture with nonunion |
| S42.151P | Displaced fracture of neck of scapula, right shoulder, subsequent encounter for fracture with malunion |
| S42.151S | Displaced fracture of neck of scapula, right shoulder, sequela |
| S42.152 | Displaced fracture of neck of scapula, left shoulder |
| S42.152A | Displaced fracture of neck of scapula, left shoulder, initial encounter for closed fracture |
| S42.152B | Displaced fracture of neck of scapula, left shoulder, initial encounter for open fracture |
| S42.152D | Displaced fracture of neck of scapula, left shoulder, subsequent encounter for fracture with routine healing |
| S42.152G | Displaced fracture of neck of scapula, left shoulder, subsequent encounter for fracture with delayed healing |
| S42.152K | Displaced fracture of neck of scapula, left shoulder, subsequent encounter for fracture with nonunion |
| S42.152P | Displaced fracture of neck of scapula, left shoulder, subsequent encounter for fracture with malunion |
| S42.152S | Displaced fracture of neck of scapula, left shoulder, sequela |
| S42.153 | Displaced fracture of neck of scapula, unspecified shoulder |
| S42.153A | Displaced fracture of neck of scapula, unspecified shoulder, initial encounter for closed fracture |
| S42.153B | Displaced fracture of neck of scapula, unspecified shoulder, initial encounter for open fracture |
| S42.153D | Displaced fracture of neck of scapula, unspecified shoulder, subsequent encounter for fracture with routine healing |
| S42.153G | Displaced fracture of neck of scapula, unspecified shoulder, subsequent encounter for fracture with delayed healing |
| S42.153K | Displaced fracture of neck of scapula, unspecified shoulder, subsequent encounter for fracture with nonunion |
| S42.153P | Displaced fracture of neck of scapula, unspecified shoulder, subsequent encounter for fracture with malunion |
| S42.153S | Displaced fracture of neck of scapula, unspecified shoulder, sequela |
| S42.154 | Nondisplaced fracture of neck of scapula, right shoulder |
| S42.154A | Nondisplaced fracture of neck of scapula, right shoulder, initial encounter for closed fracture |
| S42.154B | Nondisplaced fracture of neck of scapula, right shoulder, initial encounter for open fracture |
| S42.154D | Nondisplaced fracture of neck of scapula, right shoulder, subsequent encounter for fracture with routine healing |
| S42.154G | Nondisplaced fracture of neck of scapula, right shoulder, subsequent encounter for fracture with delayed healing |
| S42.154K | Nondisplaced fracture of neck of scapula, right shoulder, subsequent encounter for fracture with nonunion |
| S42.154P | Nondisplaced fracture of neck of scapula, right shoulder, subsequent encounter for fracture with malunion |
| S42.154S | Nondisplaced fracture of neck of scapula, right shoulder, sequela |
| S42.155 | Nondisplaced fracture of neck of scapula, left shoulder |
| S42.155A | Nondisplaced fracture of neck of scapula, left shoulder, initial encounter for closed fracture |
| S42.155B | Nondisplaced fracture of neck of scapula, left shoulder, initial encounter for open fracture |
| S42.155D | Nondisplaced fracture of neck of scapula, left shoulder, subsequent encounter for fracture with routine healing |
| S42.155G | Nondisplaced fracture of neck of scapula, left shoulder, subsequent encounter for fracture with delayed healing |
| S42.155K | Nondisplaced fracture of neck of scapula, left shoulder, subsequent encounter for fracture with nonunion |
| S42.155P | Nondisplaced fracture of neck of scapula, left shoulder, subsequent encounter for fracture with malunion |
| S42.155S | Nondisplaced fracture of neck of scapula, left shoulder, sequela |
| S42.156 | Nondisplaced fracture of neck of scapula, unspecified shoulder |
| S42.156A | Nondisplaced fracture of neck of scapula, unspecified shoulder, initial encounter for closed fracture |
| S42.156B | Nondisplaced fracture of neck of scapula, unspecified shoulder, initial encounter for open fracture |
| S42.156D | Nondisplaced fracture of neck of scapula, unspecified shoulder, subsequent encounter for fracture with routine healing |
| S42.156G | Nondisplaced fracture of neck of scapula, unspecified shoulder, subsequent encounter for fracture with delayed healing |
| S42.156K | Nondisplaced fracture of neck of scapula, unspecified shoulder, subsequent encounter for fracture with nonunion |
| S42.156P | Nondisplaced fracture of neck of scapula, unspecified shoulder, subsequent encounter for fracture with malunion |
| S42.156S | Nondisplaced fracture of neck of scapula, unspecified shoulder, sequela |
| S42.21 | Unspecified fracture of surgical neck of humerus |
| S42.211 | Unspecified displaced fracture of surgical neck of right humerus |
| S42.211A | Unspecified displaced fracture of surgical neck of right humerus, initial encounter for closed fracture |
| S42.211B | Unspecified displaced fracture of surgical neck of right humerus, initial encounter for open fracture |
| S42.211D | Unspecified displaced fracture of surgical neck of right humerus, subsequent encounter for fracture with routine healing |
| S42.211G | Unspecified displaced fracture of surgical neck of right humerus, subsequent encounter for fracture with delayed healing |
| S42.211K | Unspecified displaced fracture of surgical neck of right humerus, subsequent encounter for fracture with nonunion |
| S42.211P | Unspecified displaced fracture of surgical neck of right humerus, subsequent encounter for fracture with malunion |
| S42.211S | Unspecified displaced fracture of surgical neck of right humerus, sequela |
| S42.212 | Unspecified displaced fracture of surgical neck of left humerus |
| S42.212A | Unspecified displaced fracture of surgical neck of left humerus, initial encounter for closed fracture |
| S42.212B | Unspecified displaced fracture of surgical neck of left humerus, initial encounter for open fracture |
| S42.212D | Unspecified displaced fracture of surgical neck of left humerus, subsequent encounter for fracture with routine healing |
| S42.212G | Unspecified displaced fracture of surgical neck of left humerus, subsequent encounter for fracture with delayed healing |
| S42.212K | Unspecified displaced fracture of surgical neck of left humerus, subsequent encounter for fracture with nonunion |
| S42.212P | Unspecified displaced fracture of surgical neck of left humerus, subsequent encounter for fracture with malunion |
| S42.212S | Unspecified displaced fracture of surgical neck of left humerus, sequela |
| S42.213 | Unspecified displaced fracture of surgical neck of unspecified humerus |
| S42.213A | Unspecified displaced fracture of surgical neck of unspecified humerus, initial encounter for closed fracture |
| S42.213B | Unspecified displaced fracture of surgical neck of unspecified humerus, initial encounter for open fracture |
| S42.213D | Unspecified displaced fracture of surgical neck of unspecified humerus, subsequent encounter for fracture with routine healing |
| S42.213G | Unspecified displaced fracture of surgical neck of unspecified humerus, subsequent encounter for fracture with delayed healing |
| S42.213K | Unspecified displaced fracture of surgical neck of unspecified humerus, subsequent encounter for fracture with nonunion |
| S42.213P | Unspecified displaced fracture of surgical neck of unspecified humerus, subsequent encounter for fracture with malunion |
| S42.213S | Unspecified displaced fracture of surgical neck of unspecified humerus, sequela |
| S42.214 | Unspecified nondisplaced fracture of surgical neck of right humerus |
| S42.214A | Unspecified nondisplaced fracture of surgical neck of right humerus, initial encounter for closed fracture |
| S42.214B | Unspecified nondisplaced fracture of surgical neck of right humerus, initial encounter for open fracture |
| S42.214D | Unspecified nondisplaced fracture of surgical neck of right humerus, subsequent encounter for fracture with routine healing |
| S42.214G | Unspecified nondisplaced fracture of surgical neck of right humerus, subsequent encounter for fracture with delayed healing |
| S42.214K | Unspecified nondisplaced fracture of surgical neck of right humerus, subsequent encounter for fracture with nonunion |
| S42.214P | Unspecified nondisplaced fracture of surgical neck of right humerus, subsequent encounter for fracture with malunion |
| S42.214S | Unspecified nondisplaced fracture of surgical neck of right humerus, sequela |
| S42.215 | Unspecified nondisplaced fracture of surgical neck of left humerus |
| S42.215A | Unspecified nondisplaced fracture of surgical neck of left humerus, initial encounter for closed fracture |
| S42.215B | Unspecified nondisplaced fracture of surgical neck of left humerus, initial encounter for open fracture |
| S42.215D | Unspecified nondisplaced fracture of surgical neck of left humerus, subsequent encounter for fracture with routine healing |
| S42.215G | Unspecified nondisplaced fracture of surgical neck of left humerus, subsequent encounter for fracture with delayed healing |
| S42.215K | Unspecified nondisplaced fracture of surgical neck of left humerus, subsequent encounter for fracture with nonunion |
| S42.215P | Unspecified nondisplaced fracture of surgical neck of left humerus, subsequent encounter for fracture with malunion |
| S42.215S | Unspecified nondisplaced fracture of surgical neck of left humerus, sequela |
| S42.216 | Unspecified nondisplaced fracture of surgical neck of unspecified humerus |
| S42.216A | Unspecified nondisplaced fracture of surgical neck of unspecified humerus, initial encounter for closed fracture |
| S42.216B | Unspecified nondisplaced fracture of surgical neck of unspecified humerus, initial encounter for open fracture |
| S42.216D | Unspecified nondisplaced fracture of surgical neck of unspecified humerus, subsequent encounter for fracture with routine healing |
| S42.216G | Unspecified nondisplaced fracture of surgical neck of unspecified humerus, subsequent encounter for fracture with delayed healing |
| S42.216K | Unspecified nondisplaced fracture of surgical neck of unspecified humerus, subsequent encounter for fracture with nonunion |
| S42.216P | Unspecified nondisplaced fracture of surgical neck of unspecified humerus, subsequent encounter for fracture with malunion |
| S42.216S | Unspecified nondisplaced fracture of surgical neck of unspecified humerus, sequela |
| S42.22 | 2-part fracture of surgical neck of humerus |
| S42.221 | 2-part displaced fracture of surgical neck of right humerus |
| S42.221A | 2-part displaced fracture of surgical neck of right humerus, initial encounter for closed fracture |
| S42.221B | 2-part displaced fracture of surgical neck of right humerus, initial encounter for open fracture |
| S42.221D | 2-part displaced fracture of surgical neck of right humerus, subsequent encounter for fracture with routine healing |
| S42.221G | 2-part displaced fracture of surgical neck of right humerus, subsequent encounter for fracture with delayed healing |
| S42.221K | 2-part displaced fracture of surgical neck of right humerus, subsequent encounter for fracture with nonunion |
| S42.221P | 2-part displaced fracture of surgical neck of right humerus, subsequent encounter for fracture with malunion |
| S42.221S | 2-part displaced fracture of surgical neck of right humerus, sequela |
| S42.222 | 2-part displaced fracture of surgical neck of left humerus |
| S42.222A | 2-part displaced fracture of surgical neck of left humerus, initial encounter for closed fracture |
| S42.222B | 2-part displaced fracture of surgical neck of left humerus, initial encounter for open fracture |
| S42.222D | 2-part displaced fracture of surgical neck of left humerus, subsequent encounter for fracture with routine healing |
| S42.222G | 2-part displaced fracture of surgical neck of left humerus, subsequent encounter for fracture with delayed healing |
| S42.222K | 2-part displaced fracture of surgical neck of left humerus, subsequent encounter for fracture with nonunion |
| S42.222P | 2-part displaced fracture of surgical neck of left humerus, subsequent encounter for fracture with malunion |
| S42.222S | 2-part displaced fracture of surgical neck of left humerus, sequela |
| S42.223 | 2-part displaced fracture of surgical neck of unspecified humerus |
| S42.223A | 2-part displaced fracture of surgical neck of unspecified humerus, initial encounter for closed fracture |
| S42.223B | 2-part displaced fracture of surgical neck of unspecified humerus, initial encounter for open fracture |
| S42.223D | 2-part displaced fracture of surgical neck of unspecified humerus, subsequent encounter for fracture with routine healing |
| S42.223G | 2-part displaced fracture of surgical neck of unspecified humerus, subsequent encounter for fracture with delayed healing |
| S42.223K | 2-part displaced fracture of surgical neck of unspecified humerus, subsequent encounter for fracture with nonunion |
| S42.223P | 2-part displaced fracture of surgical neck of unspecified humerus, subsequent encounter for fracture with malunion |
| S42.223S | 2-part displaced fracture of surgical neck of unspecified humerus, sequela |
| S42.224 | 2-part nondisplaced fracture of surgical neck of right humerus |
| S42.224A | 2-part nondisplaced fracture of surgical neck of right humerus, initial encounter for closed fracture |
| S42.224B | 2-part nondisplaced fracture of surgical neck of right humerus, initial encounter for open fracture |
| S42.224D | 2-part nondisplaced fracture of surgical neck of right humerus, subsequent encounter for fracture with routine healing |
| S42.224G | 2-part nondisplaced fracture of surgical neck of right humerus, subsequent encounter for fracture with delayed healing |
| S42.224K | 2-part nondisplaced fracture of surgical neck of right humerus, subsequent encounter for fracture with nonunion |
| S42.224P | 2-part nondisplaced fracture of surgical neck of right humerus, subsequent encounter for fracture with malunion |
| S42.224S | 2-part nondisplaced fracture of surgical neck of right humerus, sequela |
| S42.225 | 2-part nondisplaced fracture of surgical neck of left humerus |
| S42.225A | 2-part nondisplaced fracture of surgical neck of left humerus, initial encounter for closed fracture |
| S42.225B | 2-part nondisplaced fracture of surgical neck of left humerus, initial encounter for open fracture |
| S42.225D | 2-part nondisplaced fracture of surgical neck of left humerus, subsequent encounter for fracture with routine healing |
| S42.225G | 2-part nondisplaced fracture of surgical neck of left humerus, subsequent encounter for fracture with delayed healing |
| S42.225K | 2-part nondisplaced fracture of surgical neck of left humerus, subsequent encounter for fracture with nonunion |
| S42.225P | 2-part nondisplaced fracture of surgical neck of left humerus, subsequent encounter for fracture with malunion |
| S42.225S | 2-part nondisplaced fracture of surgical neck of left humerus, sequela |
| S42.226 | 2-part nondisplaced fracture of surgical neck of unspecified humerus |
| S42.226A | 2-part nondisplaced fracture of surgical neck of unspecified humerus, initial encounter for closed fracture |
| S42.226B | 2-part nondisplaced fracture of surgical neck of unspecified humerus, initial encounter for open fracture |
| S42.226D | 2-part nondisplaced fracture of surgical neck of unspecified humerus, subsequent encounter for fracture with routine healing |
| S42.226G | 2-part nondisplaced fracture of surgical neck of unspecified humerus, subsequent encounter for fracture with delayed healing |
| S42.226K | 2-part nondisplaced fracture of surgical neck of unspecified humerus, subsequent encounter for fracture with nonunion |
| S42.226P | 2-part nondisplaced fracture of surgical neck of unspecified humerus, subsequent encounter for fracture with malunion |
| S42.226S | 2-part nondisplaced fracture of surgical neck of unspecified humerus, sequela |
| S42.23 | 3-part fracture of surgical neck of humerus |
| S42.231 | 3-part fracture of surgical neck of right humerus |
| S42.231A | 3-part fracture of surgical neck of right humerus, initial encounter for closed fracture |
| S42.231B | 3-part fracture of surgical neck of right humerus, initial encounter for open fracture |
| S42.231D | 3-part fracture of surgical neck of right humerus, subsequent encounter for fracture with routine healing |
| S42.231G | 3-part fracture of surgical neck of right humerus, subsequent encounter for fracture with delayed healing |
| S42.231K | 3-part fracture of surgical neck of right humerus, subsequent encounter for fracture with nonunion |
| S42.231P | 3-part fracture of surgical neck of right humerus, subsequent encounter for fracture with malunion |
| S42.231S | 3-part fracture of surgical neck of right humerus, sequela |
| S42.232 | 3-part fracture of surgical neck of left humerus |
| S42.232A | 3-part fracture of surgical neck of left humerus, initial encounter for closed fracture |
| S42.232B | 3-part fracture of surgical neck of left humerus, initial encounter for open fracture |
| S42.232D | 3-part fracture of surgical neck of left humerus, subsequent encounter for fracture with routine healing |
| S42.232G | 3-part fracture of surgical neck of left humerus, subsequent encounter for fracture with delayed healing |
| S42.232K | 3-part fracture of surgical neck of left humerus, subsequent encounter for fracture with nonunion |
| S42.232P | 3-part fracture of surgical neck of left humerus, subsequent encounter for fracture with malunion |
| S42.232S | 3-part fracture of surgical neck of left humerus, sequela |
| S42.239 | 3-part fracture of surgical neck of unspecified humerus |
| S42.239A | 3-part fracture of surgical neck of unspecified humerus, initial encounter for closed fracture |
| S42.239B | 3-part fracture of surgical neck of unspecified humerus, initial encounter for open fracture |
| S42.239D | 3-part fracture of surgical neck of unspecified humerus, subsequent encounter for fracture with routine healing |
| S42.239G | 3-part fracture of surgical neck of unspecified humerus, subsequent encounter for fracture with delayed healing |
| S42.239K | 3-part fracture of surgical neck of unspecified humerus, subsequent encounter for fracture with nonunion |
| S42.239P | 3-part fracture of surgical neck of unspecified humerus, subsequent encounter for fracture with malunion |
| S42.239S | 3-part fracture of surgical neck of unspecified humerus, sequela |
| S42.24 | 4-part fracture of surgical neck of humerus |
| S42.241 | 4-part fracture of surgical neck of right humerus |
| S42.241A | 4-part fracture of surgical neck of right humerus, initial encounter for closed fracture |
| S42.241B | 4-part fracture of surgical neck of right humerus, initial encounter for open fracture |
| S42.241D | 4-part fracture of surgical neck of right humerus, subsequent encounter for fracture with routine healing |
| S42.241G | 4-part fracture of surgical neck of right humerus, subsequent encounter for fracture with delayed healing |
| S42.241K | 4-part fracture of surgical neck of right humerus, subsequent encounter for fracture with nonunion |
| S42.241P | 4-part fracture of surgical neck of right humerus, subsequent encounter for fracture with malunion |
| S42.241S | 4-part fracture of surgical neck of right humerus, sequela |
| S42.242 | 4-part fracture of surgical neck of left humerus |
| S42.242A | 4-part fracture of surgical neck of left humerus, initial encounter for closed fracture |
| S42.242B | 4-part fracture of surgical neck of left humerus, initial encounter for open fracture |
| S42.242D | 4-part fracture of surgical neck of left humerus, subsequent encounter for fracture with routine healing |
| S42.242G | 4-part fracture of surgical neck of left humerus, subsequent encounter for fracture with delayed healing |
| S42.242K | 4-part fracture of surgical neck of left humerus, subsequent encounter for fracture with nonunion |
| S42.242P | 4-part fracture of surgical neck of left humerus, subsequent encounter for fracture with malunion |
| S42.242S | 4-part fracture of surgical neck of left humerus, sequela |
| S42.249 | 4-part fracture of surgical neck of unspecified humerus |
| S42.249A | 4-part fracture of surgical neck of unspecified humerus, initial encounter for closed fracture |
| S42.249B | 4-part fracture of surgical neck of unspecified humerus, initial encounter for open fracture |
| S42.249D | 4-part fracture of surgical neck of unspecified humerus, subsequent encounter for fracture with routine healing |
| S42.249G | 4-part fracture of surgical neck of unspecified humerus, subsequent encounter for fracture with delayed healing |
| S42.249K | 4-part fracture of surgical neck of unspecified humerus, subsequent encounter for fracture with nonunion |
| S42.249P | 4-part fracture of surgical neck of unspecified humerus, subsequent encounter for fracture with malunion |
| S42.249S | 4-part fracture of surgical neck of unspecified humerus, sequela |
| S43 | Dislocation and sprain of joints and ligaments of shoulder girdle |
| S43.0 | Subluxation and dislocation of shoulder joint |
| S43.00 | Unspecified subluxation and dislocation of shoulder joint |
| S43.001 | Unspecified subluxation of right shoulder joint |
| S43.001A | Unspecified subluxation of right shoulder joint, initial encounter |
| S43.001D | Unspecified subluxation of right shoulder joint, subsequent encounter |
| S43.001S | Unspecified subluxation of right shoulder joint, sequela |
| S43.002 | Unspecified subluxation of left shoulder joint |
| S43.002A | Unspecified subluxation of left shoulder joint, initial encounter |
| S43.002D | Unspecified subluxation of left shoulder joint, subsequent encounter |
| S43.002S | Unspecified subluxation of left shoulder joint, sequela |
| S43.003 | Unspecified subluxation of unspecified shoulder joint |
| S43.003A | Unspecified subluxation of unspecified shoulder joint, initial encounter |
| S43.003D | Unspecified subluxation of unspecified shoulder joint, subsequent encounter |
| S43.003S | Unspecified subluxation of unspecified shoulder joint, sequela |
| S43.004 | Unspecified dislocation of right shoulder joint |
| S43.004A | Unspecified dislocation of right shoulder joint, initial encounter |
| S43.004D | Unspecified dislocation of right shoulder joint, subsequent encounter |
| S43.004S | Unspecified dislocation of right shoulder joint, sequela |
| S43.005 | Unspecified dislocation of left shoulder joint |
| S43.005A | Unspecified dislocation of left shoulder joint, initial encounter |
| S43.005D | Unspecified dislocation of left shoulder joint, subsequent encounter |
| S43.005S | Unspecified dislocation of left shoulder joint, sequela |
| S43.006 | Unspecified dislocation of unspecified shoulder joint |
| S43.006A | Unspecified dislocation of unspecified shoulder joint, initial encounter |
| S43.006D | Unspecified dislocation of unspecified shoulder joint, subsequent encounter |
| S43.006S | Unspecified dislocation of unspecified shoulder joint, sequela |
| S43.01 | Anterior subluxation and dislocation of humerus |
| S43.011 | Anterior subluxation of right humerus |
| S43.011A | Anterior subluxation of right humerus, initial encounter |
| S43.011D | Anterior subluxation of right humerus, subsequent encounter |
| S43.011S | Anterior subluxation of right humerus, sequela |
| S43.012 | Anterior subluxation of left humerus |
| S43.012A | Anterior subluxation of left humerus, initial encounter |
| S43.012D | Anterior subluxation of left humerus, subsequent encounter |
| S43.012S | Anterior subluxation of left humerus, sequela |
| S43.013 | Anterior subluxation of unspecified humerus |
| S43.013A | Anterior subluxation of unspecified humerus, initial encounter |
| S43.013D | Anterior subluxation of unspecified humerus, subsequent encounter |
| S43.013S | Anterior subluxation of unspecified humerus, sequela |
| S43.014 | Anterior dislocation of right humerus |
| S43.014A | Anterior dislocation of right humerus, initial encounter |
| S43.014D | Anterior dislocation of right humerus, subsequent encounter |
| S43.014S | Anterior dislocation of right humerus, sequela |
| S43.015 | Anterior dislocation of left humerus |
| S43.015A | Anterior dislocation of left humerus, initial encounter |
| S43.015D | Anterior dislocation of left humerus, subsequent encounter |
| S43.015S | Anterior dislocation of left humerus, sequela |
| S43.016 | Anterior dislocation of unspecified humerus |
| S43.016A | Anterior dislocation of unspecified humerus, initial encounter |
| S43.016D | Anterior dislocation of unspecified humerus, subsequent encounter |
| S43.016S | Anterior dislocation of unspecified humerus, sequela |
| S43.02 | Posterior subluxation and dislocation of humerus |
| S43.021 | Posterior subluxation of right humerus |
| S43.021A | Posterior subluxation of right humerus, initial encounter |
| S43.021D | Posterior subluxation of right humerus, subsequent encounter |
| S43.021S | Posterior subluxation of right humerus, sequela |
| S43.022 | Posterior subluxation of left humerus |
| S43.022A | Posterior subluxation of left humerus, initial encounter |
| S43.022D | Posterior subluxation of left humerus, subsequent encounter |
| S43.022S | Posterior subluxation of left humerus, sequela |
| S43.023 | Posterior subluxation of unspecified humerus |
| S43.023A | Posterior subluxation of unspecified humerus, initial encounter |
| S43.023D | Posterior subluxation of unspecified humerus, subsequent encounter |
| S43.023S | Posterior subluxation of unspecified humerus, sequela |
| S43.024 | Posterior dislocation of right humerus |
| S43.024A | Posterior dislocation of right humerus, initial encounter |
| S43.024D | Posterior dislocation of right humerus, subsequent encounter |
| S43.024S | Posterior dislocation of right humerus, sequela |
| S43.025 | Posterior dislocation of left humerus |
| S43.025A | Posterior dislocation of left humerus, initial encounter |
| S43.025D | Posterior dislocation of left humerus, subsequent encounter |
| S43.025S | Posterior dislocation of left humerus, sequela |
| S43.026 | Posterior dislocation of unspecified humerus |
| S43.026A | Posterior dislocation of unspecified humerus, initial encounter |
| S43.026D | Posterior dislocation of unspecified humerus, subsequent encounter |
| S43.026S | Posterior dislocation of unspecified humerus, sequela |
| S43.03 | Inferior subluxation and dislocation of humerus |
| S43.031 | Inferior subluxation of right humerus |
| S43.031A | Inferior subluxation of right humerus, initial encounter |
| S43.031D | Inferior subluxation of right humerus, subsequent encounter |
| S43.031S | Inferior subluxation of right humerus, sequela |
| S43.032 | Inferior subluxation of left humerus |
| S43.032A | Inferior subluxation of left humerus, initial encounter |
| S43.032D | Inferior subluxation of left humerus, subsequent encounter |
| S43.032S | Inferior subluxation of left humerus, sequela |
| S43.033 | Inferior subluxation of unspecified humerus |
| S43.033A | Inferior subluxation of unspecified humerus, initial encounter |
| S43.033D | Inferior subluxation of unspecified humerus, subsequent encounter |
| S43.033S | Inferior subluxation of unspecified humerus, sequela |
| S43.034 | Inferior dislocation of right humerus |
| S43.034A | Inferior dislocation of right humerus, initial encounter |
| S43.034D | Inferior dislocation of right humerus, subsequent encounter |
| S43.034S | Inferior dislocation of right humerus, sequela |
| S43.035 | Inferior dislocation of left humerus |
| S43.035A | Inferior dislocation of left humerus, initial encounter |
| S43.035D | Inferior dislocation of left humerus, subsequent encounter |
| S43.035S | Inferior dislocation of left humerus, sequela |
| S43.036 | Inferior dislocation of unspecified humerus |
| S43.036A | Inferior dislocation of unspecified humerus, initial encounter |
| S43.036D | Inferior dislocation of unspecified humerus, subsequent encounter |
| S43.036S | Inferior dislocation of unspecified humerus, sequela |
| S43.08 | Other subluxation and dislocation of shoulder joint |
| S43.081 | Other subluxation of right shoulder joint |
| S43.081A | Other subluxation of right shoulder joint, initial encounter |
| S43.081D | Other subluxation of right shoulder joint, subsequent encounter |
| S43.081S | Other subluxation of right shoulder joint, sequela |
| S43.082 | Other subluxation of left shoulder joint |
| S43.082A | Other subluxation of left shoulder joint, initial encounter |
| S43.082D | Other subluxation of left shoulder joint, subsequent encounter |
| S43.082S | Other subluxation of left shoulder joint, sequela |
| S43.083 | Other subluxation of unspecified shoulder joint |
| S43.083A | Other subluxation of unspecified shoulder joint, initial encounter |
| S43.083D | Other subluxation of unspecified shoulder joint, subsequent encounter |
| S43.083S | Other subluxation of unspecified shoulder joint, sequela |
| S43.084 | Other dislocation of right shoulder joint |
| S43.084A | Other dislocation of right shoulder joint, initial encounter |
| S43.084D | Other dislocation of right shoulder joint, subsequent encounter |
| S43.084S | Other dislocation of right shoulder joint, sequela |
| S43.085 | Other dislocation of left shoulder joint |
| S43.085A | Other dislocation of left shoulder joint, initial encounter |
| S43.085D | Other dislocation of left shoulder joint, subsequent encounter |
| S43.085S | Other dislocation of left shoulder joint, sequela |
| S43.086 | Other dislocation of unspecified shoulder joint |
| S43.086A | Other dislocation of unspecified shoulder joint, initial encounter |
| S43.086D | Other dislocation of unspecified shoulder joint, subsequent encounter |
| S43.086S | Other dislocation of unspecified shoulder joint, sequela |
| S43.1 | Subluxation and dislocation of acromioclavicular joint |
| S43.10 | Unspecified dislocation of acromioclavicular joint |
| S43.101 | Unspecified dislocation of right acromioclavicular joint |
| S43.101A | Unspecified dislocation of right acromioclavicular joint, initial encounter |
| S43.101D | Unspecified dislocation of right acromioclavicular joint, subsequent encounter |
| S43.101S | Unspecified dislocation of right acromioclavicular joint, sequela |
| S43.102 | Unspecified dislocation of left acromioclavicular joint |
| S43.102A | Unspecified dislocation of left acromioclavicular joint, initial encounter |
| S43.102D | Unspecified dislocation of left acromioclavicular joint, subsequent encounter |
| S43.102S | Unspecified dislocation of left acromioclavicular joint, sequela |
| S43.109 | Unspecified dislocation of unspecified acromioclavicular joint |
| S43.109A | Unspecified dislocation of unspecified acromioclavicular joint, initial encounter |
| S43.109D | Unspecified dislocation of unspecified acromioclavicular joint, subsequent encounter |
| S43.109S | Unspecified dislocation of unspecified acromioclavicular joint, sequela |
| S43.11 | Subluxation of acromioclavicular joint |
| S43.111 | Subluxation of right acromioclavicular joint |
| S43.111A | Subluxation of right acromioclavicular joint, initial encounter |
| S43.111D | Subluxation of right acromioclavicular joint, subsequent encounter |
| S43.111S | Subluxation of right acromioclavicular joint, sequela |
| S43.112 | Subluxation of left acromioclavicular joint |
| S43.112A | Subluxation of left acromioclavicular joint, initial encounter |
| S43.112D | Subluxation of left acromioclavicular joint, subsequent encounter |
| S43.112S | Subluxation of left acromioclavicular joint, sequela |
| S43.119 | Subluxation of unspecified acromioclavicular joint |
| S43.119A | Subluxation of unspecified acromioclavicular joint, initial encounter |
| S43.119D | Subluxation of unspecified acromioclavicular joint, subsequent encounter |
| S43.119S | Subluxation of unspecified acromioclavicular joint, sequela |
| S43.12 | Dislocation of acromioclavicular joint, 100%-200% displacement |
| S43.121 | Dislocation of right acromioclavicular joint, 100%-200% displacement |
| S43.121A | Dislocation of right acromioclavicular joint, 100%-200% displacement, initial encounter |
| S43.121D | Dislocation of right acromioclavicular joint, 100%-200% displacement, subsequent encounter |
| S43.121S | Dislocation of right acromioclavicular joint, 100%-200% displacement, sequela |
| S43.122 | Dislocation of left acromioclavicular joint, 100%-200% displacement |
| S43.122A | Dislocation of left acromioclavicular joint, 100%-200% displacement, initial encounter |
| S43.122D | Dislocation of left acromioclavicular joint, 100%-200% displacement, subsequent encounter |
| S43.122S | Dislocation of left acromioclavicular joint, 100%-200% displacement, sequela |
| S43.129 | Dislocation of unspecified acromioclavicular joint, 100%-200% displacement |
| S43.129A | Dislocation of unspecified acromioclavicular joint, 100%-200% displacement, initial encounter |
| S43.129D | Dislocation of unspecified acromioclavicular joint, 100%-200% displacement, subsequent encounter |
| S43.129S | Dislocation of unspecified acromioclavicular joint, 100%-200% displacement, sequela |
| S43.13 | Dislocation of acromioclavicular joint, greater than 200% displacement |
| S43.131 | Dislocation of right acromioclavicular joint, greater than 200% displacement |
| S43.131A | Dislocation of right acromioclavicular joint, greater than 200% displacement, initial encounter |
| S43.131D | Dislocation of right acromioclavicular joint, greater than 200% displacement, subsequent encounter |
| S43.131S | Dislocation of right acromioclavicular joint, greater than 200% displacement, sequela |
| S43.132 | Dislocation of left acromioclavicular joint, greater than 200% displacement |
| S43.132A | Dislocation of left acromioclavicular joint, greater than 200% displacement, initial encounter |
| S43.132D | Dislocation of left acromioclavicular joint, greater than 200% displacement, subsequent encounter |
| S43.132S | Dislocation of left acromioclavicular joint, greater than 200% displacement, sequela |
| S43.139 | Dislocation of unspecified acromioclavicular joint, greater than 200% displacement |
| S43.139A | Dislocation of unspecified acromioclavicular joint, greater than 200% displacement, initial encounter |
| S43.139D | Dislocation of unspecified acromioclavicular joint, greater than 200% displacement, subsequent encounter |
| S43.139S | Dislocation of unspecified acromioclavicular joint, greater than 200% displacement, sequela |
| S43.14 | Inferior dislocation of acromioclavicular joint |
| S43.141 | Inferior dislocation of right acromioclavicular joint |
| S43.141A | Inferior dislocation of right acromioclavicular joint, initial encounter |
| S43.141D | Inferior dislocation of right acromioclavicular joint, subsequent encounter |
| S43.141S | Inferior dislocation of right acromioclavicular joint, sequela |
| S43.142 | Inferior dislocation of left acromioclavicular joint |
| S43.142A | Inferior dislocation of left acromioclavicular joint, initial encounter |
| S43.142D | Inferior dislocation of left acromioclavicular joint, subsequent encounter |
| S43.142S | Inferior dislocation of left acromioclavicular joint, sequela |
| S43.149 | Inferior dislocation of unspecified acromioclavicular joint |
| S43.149A | Inferior dislocation of unspecified acromioclavicular joint, initial encounter |
| S43.149D | Inferior dislocation of unspecified acromioclavicular joint, subsequent encounter |
| S43.149S | Inferior dislocation of unspecified acromioclavicular joint, sequela |
| S43.15 | Posterior dislocation of acromioclavicular joint |
| S43.151 | Posterior dislocation of right acromioclavicular joint |
| S43.151A | Posterior dislocation of right acromioclavicular joint, initial encounter |
| S43.151D | Posterior dislocation of right acromioclavicular joint, subsequent encounter |
| S43.151S | Posterior dislocation of right acromioclavicular joint, sequela |
| S43.152 | Posterior dislocation of left acromioclavicular joint |
| S43.152A | Posterior dislocation of left acromioclavicular joint, initial encounter |
| S43.152D | Posterior dislocation of left acromioclavicular joint, subsequent encounter |
| S43.152S | Posterior dislocation of left acromioclavicular joint, sequela |
| S43.159 | Posterior dislocation of unspecified acromioclavicular joint |
| S43.159A | Posterior dislocation of unspecified acromioclavicular joint, initial encounter |
| S43.159D | Posterior dislocation of unspecified acromioclavicular joint, subsequent encounter |
| S43.159S | Posterior dislocation of unspecified acromioclavicular joint, sequela |
| S43.2 | Subluxation and dislocation of sternoclavicular joint |
| S43.20 | Unspecified subluxation and dislocation of sternoclavicular joint |
| S43.201 | Unspecified subluxation of right sternoclavicular joint |
| S43.201A | Unspecified subluxation of right sternoclavicular joint, initial encounter |
| S43.201D | Unspecified subluxation of right sternoclavicular joint, subsequent encounter |
| S43.201S | Unspecified subluxation of right sternoclavicular joint, sequela |
| S43.202 | Unspecified subluxation of left sternoclavicular joint |
| S43.202A | Unspecified subluxation of left sternoclavicular joint, initial encounter |
| S43.202D | Unspecified subluxation of left sternoclavicular joint, subsequent encounter |
| S43.202S | Unspecified subluxation of left sternoclavicular joint, sequela |
| S43.203 | Unspecified subluxation of unspecified sternoclavicular joint |
| S43.203A | Unspecified subluxation of unspecified sternoclavicular joint, initial encounter |
| S43.203D | Unspecified subluxation of unspecified sternoclavicular joint, subsequent encounter |
| S43.203S | Unspecified subluxation of unspecified sternoclavicular joint, sequela |
| S43.204 | Unspecified dislocation of right sternoclavicular joint |
| S43.204A | Unspecified dislocation of right sternoclavicular joint, initial encounter |
| S43.204D | Unspecified dislocation of right sternoclavicular joint, subsequent encounter |
| S43.204S | Unspecified dislocation of right sternoclavicular joint, sequela |
| S43.205 | Unspecified dislocation of left sternoclavicular joint |
| S43.205A | Unspecified dislocation of left sternoclavicular joint, initial encounter |
| S43.205D | Unspecified dislocation of left sternoclavicular joint, subsequent encounter |
| S43.205S | Unspecified dislocation of left sternoclavicular joint, sequela |
| S43.206 | Unspecified dislocation of unspecified sternoclavicular joint |
| S43.206A | Unspecified dislocation of unspecified sternoclavicular joint, initial encounter |
| S43.206D | Unspecified dislocation of unspecified sternoclavicular joint, subsequent encounter |
| S43.206S | Unspecified dislocation of unspecified sternoclavicular joint, sequela |
| S43.21 | Anterior subluxation and dislocation of sternoclavicular joint |
| S43.211 | Anterior subluxation of right sternoclavicular joint |
| S43.211A | Anterior subluxation of right sternoclavicular joint, initial encounter |
| S43.211D | Anterior subluxation of right sternoclavicular joint, subsequent encounter |
| S43.211S | Anterior subluxation of right sternoclavicular joint, sequela |
| S43.212 | Anterior subluxation of left sternoclavicular joint |
| S43.212A | Anterior subluxation of left sternoclavicular joint, initial encounter |
| S43.212D | Anterior subluxation of left sternoclavicular joint, subsequent encounter |
| S43.212S | Anterior subluxation of left sternoclavicular joint, sequela |
| S43.213 | Anterior subluxation of unspecified sternoclavicular joint |
| S43.213A | Anterior subluxation of unspecified sternoclavicular joint, initial encounter |
| S43.213D | Anterior subluxation of unspecified sternoclavicular joint, subsequent encounter |
| S43.213S | Anterior subluxation of unspecified sternoclavicular joint, sequela |
| S43.214 | Anterior dislocation of right sternoclavicular joint |
| S43.214A | Anterior dislocation of right sternoclavicular joint, initial encounter |
| S43.214D | Anterior dislocation of right sternoclavicular joint, subsequent encounter |
| S43.214S | Anterior dislocation of right sternoclavicular joint, sequela |
| S43.215 | Anterior dislocation of left sternoclavicular joint |
| S43.215A | Anterior dislocation of left sternoclavicular joint, initial encounter |
| S43.215D | Anterior dislocation of left sternoclavicular joint, subsequent encounter |
| S43.215S | Anterior dislocation of left sternoclavicular joint, sequela |
| S43.216 | Anterior dislocation of unspecified sternoclavicular joint |
| S43.216A | Anterior dislocation of unspecified sternoclavicular joint, initial encounter |
| S43.216D | Anterior dislocation of unspecified sternoclavicular joint, subsequent encounter |
| S43.216S | Anterior dislocation of unspecified sternoclavicular joint, sequela |
| S43.22 | Posterior subluxation and dislocation of sternoclavicular joint |
| S43.221 | Posterior subluxation of right sternoclavicular joint |
| S43.221A | Posterior subluxation of right sternoclavicular joint, initial encounter |
| S43.221D | Posterior subluxation of right sternoclavicular joint, subsequent encounter |
| S43.221S | Posterior subluxation of right sternoclavicular joint, sequela |
| S43.222 | Posterior subluxation of left sternoclavicular joint |
| S43.222A | Posterior subluxation of left sternoclavicular joint, initial encounter |
| S43.222D | Posterior subluxation of left sternoclavicular joint, subsequent encounter |
| S43.222S | Posterior subluxation of left sternoclavicular joint, sequela |
| S43.223 | Posterior subluxation of unspecified sternoclavicular joint |
| S43.223A | Posterior subluxation of unspecified sternoclavicular joint, initial encounter |
| S43.223D | Posterior subluxation of unspecified sternoclavicular joint, subsequent encounter |
| S43.223S | Posterior subluxation of unspecified sternoclavicular joint, sequela |
| S43.224 | Posterior dislocation of right sternoclavicular joint |
| S43.224A | Posterior dislocation of right sternoclavicular joint, initial encounter |
| S43.224D | Posterior dislocation of right sternoclavicular joint, subsequent encounter |
| S43.224S | Posterior dislocation of right sternoclavicular joint, sequela |
| S43.225 | Posterior dislocation of left sternoclavicular joint |
| S43.225A | Posterior dislocation of left sternoclavicular joint, initial encounter |
| S43.225D | Posterior dislocation of left sternoclavicular joint, subsequent encounter |
| S43.225S | Posterior dislocation of left sternoclavicular joint, sequela |
| S43.226 | Posterior dislocation of unspecified sternoclavicular joint |
| S43.226A | Posterior dislocation of unspecified sternoclavicular joint, initial encounter |
| S43.226D | Posterior dislocation of unspecified sternoclavicular joint, subsequent encounter |
| S43.226S | Posterior dislocation of unspecified sternoclavicular joint, sequela |
| S43.3 | Subluxation and dislocation of other and unspecified parts of shoulder girdle |
| S43.30 | Subluxation and dislocation of unspecified parts of shoulder girdle |
| S43.301 | Subluxation of unspecified parts of right shoulder girdle |
| S43.301A | Subluxation of unspecified parts of right shoulder girdle, initial encounter |
| S43.301D | Subluxation of unspecified parts of right shoulder girdle, subsequent encounter |
| S43.301S | Subluxation of unspecified parts of right shoulder girdle, sequela |
| S43.302 | Subluxation of unspecified parts of left shoulder girdle |
| S43.302A | Subluxation of unspecified parts of left shoulder girdle, initial encounter |
| S43.302D | Subluxation of unspecified parts of left shoulder girdle, subsequent encounter |
| S43.302S | Subluxation of unspecified parts of left shoulder girdle, sequela |
| S43.303 | Subluxation of unspecified parts of unspecified shoulder girdle |
| S43.303A | Subluxation of unspecified parts of unspecified shoulder girdle, initial encounter |
| S43.303D | Subluxation of unspecified parts of unspecified shoulder girdle, subsequent encounter |
| S43.303S | Subluxation of unspecified parts of unspecified shoulder girdle, sequela |
| S43.304 | Dislocation of unspecified parts of right shoulder girdle |
| S43.304A | Dislocation of unspecified parts of right shoulder girdle, initial encounter |
| S43.304D | Dislocation of unspecified parts of right shoulder girdle, subsequent encounter |
| S43.304S | Dislocation of unspecified parts of right shoulder girdle, sequela |
| S43.305 | Dislocation of unspecified parts of left shoulder girdle |
| S43.305A | Dislocation of unspecified parts of left shoulder girdle, initial encounter |
| S43.305D | Dislocation of unspecified parts of left shoulder girdle, subsequent encounter |
| S43.305S | Dislocation of unspecified parts of left shoulder girdle, sequela |
| S43.306 | Dislocation of unspecified parts of unspecified shoulder girdle |
| S43.306A | Dislocation of unspecified parts of unspecified shoulder girdle, initial encounter |
| S43.306D | Dislocation of unspecified parts of unspecified shoulder girdle, subsequent encounter |
| S43.306S | Dislocation of unspecified parts of unspecified shoulder girdle, sequela |
| S43.31 | Subluxation and dislocation of scapula |
| S43.311 | Subluxation of right scapula |
| S43.311A | Subluxation of right scapula, initial encounter |
| S43.311D | Subluxation of right scapula, subsequent encounter |
| S43.311S | Subluxation of right scapula, sequela |
| S43.312 | Subluxation of left scapula |
| S43.312A | Subluxation of left scapula, initial encounter |
| S43.312D | Subluxation of left scapula, subsequent encounter |
| S43.312S | Subluxation of left scapula, sequela |
| S43.313 | Subluxation of unspecified scapula |
| S43.313A | Subluxation of unspecified scapula, initial encounter |
| S43.313D | Subluxation of unspecified scapula, subsequent encounter |
| S43.313S | Subluxation of unspecified scapula, sequela |
| S43.314 | Dislocation of right scapula |
| S43.314A | Dislocation of right scapula, initial encounter |
| S43.314D | Dislocation of right scapula, subsequent encounter |
| S43.314S | Dislocation of right scapula, sequela |
| S43.315 | Dislocation of left scapula |
| S43.315A | Dislocation of left scapula, initial encounter |
| S43.315D | Dislocation of left scapula, subsequent encounter |
| S43.315S | Dislocation of left scapula, sequela |
| S43.316 | Dislocation of unspecified scapula |
| S43.316A | Dislocation of unspecified scapula, initial encounter |
| S43.316D | Dislocation of unspecified scapula, subsequent encounter |
| S43.316S | Dislocation of unspecified scapula, sequela |
| S43.39 | Subluxation and dislocation of other parts of shoulder girdle |
| S43.391 | Subluxation of other parts of right shoulder girdle |
| S43.391A | Subluxation of other parts of right shoulder girdle, initial encounter |
| S43.391D | Subluxation of other parts of right shoulder girdle, subsequent encounter |
| S43.391S | Subluxation of other parts of right shoulder girdle, sequela |
| S43.392 | Subluxation of other parts of left shoulder girdle |
| S43.392A | Subluxation of other parts of left shoulder girdle, initial encounter |
| S43.392D | Subluxation of other parts of left shoulder girdle, subsequent encounter |
| S43.392S | Subluxation of other parts of left shoulder girdle, sequela |
| S43.393 | Subluxation of other parts of unspecified shoulder girdle |
| S43.393A | Subluxation of other parts of unspecified shoulder girdle, initial encounter |
| S43.393D | Subluxation of other parts of unspecified shoulder girdle, subsequent encounter |
| S43.393S | Subluxation of other parts of unspecified shoulder girdle, sequela |
| S43.394 | Dislocation of other parts of right shoulder girdle |
| S43.394A | Dislocation of other parts of right shoulder girdle, initial encounter |
| S43.394D | Dislocation of other parts of right shoulder girdle, subsequent encounter |
| S43.394S | Dislocation of other parts of right shoulder girdle, sequela |
| S43.395 | Dislocation of other parts of left shoulder girdle |
| S43.395A | Dislocation of other parts of left shoulder girdle, initial encounter |
| S43.395D | Dislocation of other parts of left shoulder girdle, subsequent encounter |
| S43.395S | Dislocation of other parts of left shoulder girdle, sequela |
| S43.396 | Dislocation of other parts of unspecified shoulder girdle |
| S43.396A | Dislocation of other parts of unspecified shoulder girdle, initial encounter |
| S43.396D | Dislocation of other parts of unspecified shoulder girdle, subsequent encounter |
| S43.396S | Dislocation of other parts of unspecified shoulder girdle, sequela |
| S43.4 | Sprain of shoulder joint |
| S43.40 | Unspecified sprain of shoulder joint |
| S43.401 | Unspecified sprain of right shoulder joint |
| S43.401A | Unspecified sprain of right shoulder joint, initial encounter |
| S43.401D | Unspecified sprain of right shoulder joint, subsequent encounter |
| S43.401S | Unspecified sprain of right shoulder joint, sequela |
| S43.402 | Unspecified sprain of left shoulder joint |
| S43.402A | Unspecified sprain of left shoulder joint, initial encounter |
| S43.402D | Unspecified sprain of left shoulder joint, subsequent encounter |
| S43.402S | Unspecified sprain of left shoulder joint, sequela |
| S43.409 | Unspecified sprain of unspecified shoulder joint |
| S43.409A | Unspecified sprain of unspecified shoulder joint, initial encounter |
| S43.409D | Unspecified sprain of unspecified shoulder joint, subsequent encounter |
| S43.409S | Unspecified sprain of unspecified shoulder joint, sequela |
| S43.41 | Sprain of coracohumeral (ligament) |
| S43.411 | Sprain of right coracohumeral (ligament) |
| S43.411A | Sprain of right coracohumeral (ligament), initial encounter |
| S43.411D | Sprain of right coracohumeral (ligament), subsequent encounter |
| S43.411S | Sprain of right coracohumeral (ligament), sequela |
| S43.412 | Sprain of left coracohumeral (ligament) |
| S43.412A | Sprain of left coracohumeral (ligament), initial encounter |
| S43.412D | Sprain of left coracohumeral (ligament), subsequent encounter |
| S43.412S | Sprain of left coracohumeral (ligament), sequela |
| S43.419 | Sprain of unspecified coracohumeral (ligament) |
| S43.419A | Sprain of unspecified coracohumeral (ligament), initial encounter |
| S43.419D | Sprain of unspecified coracohumeral (ligament), subsequent encounter |
| S43.419S | Sprain of unspecified coracohumeral (ligament), sequela |
| S43.42 | Sprain of rotator cuff capsule |
| S43.421 | Sprain of right rotator cuff capsule |
| S43.421A | Sprain of right rotator cuff capsule, initial encounter |
| S43.421D | Sprain of right rotator cuff capsule, subsequent encounter |
| S43.421S | Sprain of right rotator cuff capsule, sequela |
| S43.422 | Sprain of left rotator cuff capsule |
| S43.422A | Sprain of left rotator cuff capsule, initial encounter |
| S43.422D | Sprain of left rotator cuff capsule, subsequent encounter |
| S43.422S | Sprain of left rotator cuff capsule, sequela |
| S43.429 | Sprain of unspecified rotator cuff capsule |
| S43.429A | Sprain of unspecified rotator cuff capsule, initial encounter |
| S43.429D | Sprain of unspecified rotator cuff capsule, subsequent encounter |
| S43.429S | Sprain of unspecified rotator cuff capsule, sequela |
| S43.43 | Superior glenoid labrum lesion |
| S43.431 | Superior glenoid labrum lesion of right shoulder |
| S43.431A | Superior glenoid labrum lesion of right shoulder, initial encounter |
| S43.431D | Superior glenoid labrum lesion of right shoulder, subsequent encounter |
| S43.431S | Superior glenoid labrum lesion of right shoulder, sequela |
| S43.432 | Superior glenoid labrum lesion of left shoulder |
| S43.432A | Superior glenoid labrum lesion of left shoulder, initial encounter |
| S43.432D | Superior glenoid labrum lesion of left shoulder, subsequent encounter |
| S43.432S | Superior glenoid labrum lesion of left shoulder, sequela |
| S43.439 | Superior glenoid labrum lesion of unspecified shoulder |
| S43.439A | Superior glenoid labrum lesion of unspecified shoulder, initial encounter |
| S43.439D | Superior glenoid labrum lesion of unspecified shoulder, subsequent encounter |
| S43.439S | Superior glenoid labrum lesion of unspecified shoulder, sequela |
| S43.49 | Other sprain of shoulder joint |
| S43.491 | Other sprain of right shoulder joint |
| S43.491A | Other sprain of right shoulder joint, initial encounter |
| S43.491D | Other sprain of right shoulder joint, subsequent encounter |
| S43.491S | Other sprain of right shoulder joint, sequela |
| S43.492 | Other sprain of left shoulder joint |
| S43.492A | Other sprain of left shoulder joint, initial encounter |
| S43.492D | Other sprain of left shoulder joint, subsequent encounter |
| S43.492S | Other sprain of left shoulder joint, sequela |
| S43.499 | Other sprain of unspecified shoulder joint |
| S43.499A | Other sprain of unspecified shoulder joint, initial encounter |
| S43.499D | Other sprain of unspecified shoulder joint, subsequent encounter |
| S43.499S | Other sprain of unspecified shoulder joint, sequela |
| S43.5 | Sprain of acromioclavicular joint |
| S43.50 | Sprain of unspecified acromioclavicular joint |
| S43.50XA | Sprain of unspecified acromioclavicular joint, initial encounter |
| S43.50XD | Sprain of unspecified acromioclavicular joint, subsequent encounter |
| S43.50XS | Sprain of unspecified acromioclavicular joint, sequela |
| S43.51 | Sprain of right acromioclavicular joint |
| S43.51XA | Sprain of right acromioclavicular joint, initial encounter |
| S43.51XD | Sprain of right acromioclavicular joint, subsequent encounter |
| S43.51XS | Sprain of right acromioclavicular joint, sequela |
| S43.52 | Sprain of left acromioclavicular joint |
| S43.52XA | Sprain of left acromioclavicular joint, initial encounter |
| S43.52XD | Sprain of left acromioclavicular joint, subsequent encounter |
| S43.52XS | Sprain of left acromioclavicular joint, sequela |
| S43.6 | Sprain of sternoclavicular joint |
| S43.60 | Sprain of unspecified sternoclavicular joint |
| S43.60XA | Sprain of unspecified sternoclavicular joint, initial encounter |
| S43.60XD | Sprain of unspecified sternoclavicular joint, subsequent encounter |
| S43.60XS | Sprain of unspecified sternoclavicular joint, sequela |
| S43.61 | Sprain of right sternoclavicular joint |
| S43.61XA | Sprain of right sternoclavicular joint, initial encounter |
| S43.61XD | Sprain of right sternoclavicular joint, subsequent encounter |
| S43.61XS | Sprain of right sternoclavicular joint, sequela |
| S43.62 | Sprain of left sternoclavicular joint |
| S43.62XA | Sprain of left sternoclavicular joint, initial encounter |
| S43.62XD | Sprain of left sternoclavicular joint, subsequent encounter |
| S43.62XS | Sprain of left sternoclavicular joint, sequela |
| S43.8 | Sprain of other specified parts of shoulder girdle |
| S43.80 | Sprain of other specified parts of unspecified shoulder girdle |
| S43.80XA | Sprain of other specified parts of unspecified shoulder girdle, initial encounter |
| S43.80XD | Sprain of other specified parts of unspecified shoulder girdle, subsequent encounter |
| S43.80XS | Sprain of other specified parts of unspecified shoulder girdle, sequela |
| S43.81 | Sprain of other specified parts of right shoulder girdle |
| S43.81XA | Sprain of other specified parts of right shoulder girdle, initial encounter |
| S43.81XD | Sprain of other specified parts of right shoulder girdle, subsequent encounter |
| S43.81XS | Sprain of other specified parts of right shoulder girdle, sequela |
| S43.82 | Sprain of other specified parts of left shoulder girdle |
| S43.82XA | Sprain of other specified parts of left shoulder girdle, initial encounter |
| S43.82XD | Sprain of other specified parts of left shoulder girdle, subsequent encounter |
| S43.82XS | Sprain of other specified parts of left shoulder girdle, sequela |
| S43.9 | Sprain of unspecified parts of shoulder girdle |
| S43.90 | Sprain of unspecified parts of unspecified shoulder girdle |
| S43.90XA | Sprain of unspecified parts of unspecified shoulder girdle, initial encounter |
| S43.90XD | Sprain of unspecified parts of unspecified shoulder girdle, subsequent encounter |
| S43.90XS | Sprain of unspecified parts of unspecified shoulder girdle, sequela |
| S43.91 | Sprain of unspecified parts of right shoulder girdle |
| S43.91XA | Sprain of unspecified parts of right shoulder girdle, initial encounter |
| S43.91XD | Sprain of unspecified parts of right shoulder girdle, subsequent encounter |
| S43.91XS | Sprain of unspecified parts of right shoulder girdle, sequela |
| S43.92 | Sprain of unspecified parts of left shoulder girdle |
| S43.92XA | Sprain of unspecified parts of left shoulder girdle, initial encounter |
| S43.92XD | Sprain of unspecified parts of left shoulder girdle, subsequent encounter |
| S43.92XS | Sprain of unspecified parts of left shoulder girdle, sequela |
| S52.13 | Fracture of neck of radius |
| S52.131 | Displaced fracture of neck of right radius |
| S52.131A | Displaced fracture of neck of right radius, initial encounter for closed fracture |
| S52.131B | Displaced fracture of neck of right radius, initial encounter for open fracture type I or II |
| S52.131C | Displaced fracture of neck of right radius, initial encounter for open fracture type IIIA, IIIB, or IIIC |
| S52.131D | Displaced fracture of neck of right radius, subsequent encounter for closed fracture with routine healing |
| S52.131E | Displaced fracture of neck of right radius, subsequent encounter for open fracture type I or II with routine healing |
| S52.131F | Displaced fracture of neck of right radius, subsequent encounter for open fracture type IIIA, IIIB, or IIIC with routine healing |
| S52.131G | Displaced fracture of neck of right radius, subsequent encounter for closed fracture with delayed healing |
| S52.131H | Displaced fracture of neck of right radius, subsequent encounter for open fracture type I or II with delayed healing |
| S52.131J | Displaced fracture of neck of right radius, subsequent encounter for open fracture type IIIA, IIIB, or IIIC with delayed healing |
| S52.131K | Displaced fracture of neck of right radius, subsequent encounter for closed fracture with nonunion |
| S52.131M | Displaced fracture of neck of right radius, subsequent encounter for open fracture type I or II with nonunion |
| S52.131N | Displaced fracture of neck of right radius, subsequent encounter for open fracture type IIIA, IIIB, or IIIC with nonunion |
| S52.131P | Displaced fracture of neck of right radius, subsequent encounter for closed fracture with malunion |
| S52.131Q | Displaced fracture of neck of right radius, subsequent encounter for open fracture type I or II with malunion |
| S52.131R | Displaced fracture of neck of right radius, subsequent encounter for open fracture type IIIA, IIIB, or IIIC with malunion |
| S52.131S | Displaced fracture of neck of right radius, sequela |
| S52.132 | Displaced fracture of neck of left radius |
| S52.132A | Displaced fracture of neck of left radius, initial encounter for closed fracture |
| S52.132B | Displaced fracture of neck of left radius, initial encounter for open fracture type I or II |
| S52.132C | Displaced fracture of neck of left radius, initial encounter for open fracture type IIIA, IIIB, or IIIC |
| S52.132D | Displaced fracture of neck of left radius, subsequent encounter for closed fracture with routine healing |
| S52.132E | Displaced fracture of neck of left radius, subsequent encounter for open fracture type I or II with routine healing |
| S52.132F | Displaced fracture of neck of left radius, subsequent encounter for open fracture type IIIA, IIIB, or IIIC with routine healing |
| S52.132G | Displaced fracture of neck of left radius, subsequent encounter for closed fracture with delayed healing |
| S52.132H | Displaced fracture of neck of left radius, subsequent encounter for open fracture type I or II with delayed healing |
| S52.132J | Displaced fracture of neck of left radius, subsequent encounter for open fracture type IIIA, IIIB, or IIIC with delayed healing |
| S52.132K | Displaced fracture of neck of left radius, subsequent encounter for closed fracture with nonunion |
| S52.132M | Displaced fracture of neck of left radius, subsequent encounter for open fracture type I or II with nonunion |
| S52.132N | Displaced fracture of neck of left radius, subsequent encounter for open fracture type IIIA, IIIB, or IIIC with nonunion |
| S52.132P | Displaced fracture of neck of left radius, subsequent encounter for closed fracture with malunion |
| S52.132Q | Displaced fracture of neck of left radius, subsequent encounter for open fracture type I or II with malunion |
| S52.132R | Displaced fracture of neck of left radius, subsequent encounter for open fracture type IIIA, IIIB, or IIIC with malunion |
| S52.132S | Displaced fracture of neck of left radius, sequela |
| S52.133 | Displaced fracture of neck of unspecified radius |
| S52.133A | Displaced fracture of neck of unspecified radius, initial encounter for closed fracture |
| S52.133B | Displaced fracture of neck of unspecified radius, initial encounter for open fracture type I or II |
| S52.133C | Displaced fracture of neck of unspecified radius, initial encounter for open fracture type IIIA, IIIB, or IIIC |
| S52.133D | Displaced fracture of neck of unspecified radius, subsequent encounter for closed fracture with routine healing |
| S52.133E | Displaced fracture of neck of unspecified radius, subsequent encounter for open fracture type I or II with routine healing |
| S52.133F | Displaced fracture of neck of unspecified radius, subsequent encounter for open fracture type IIIA, IIIB, or IIIC with routine healing |
| S52.133G | Displaced fracture of neck of unspecified radius, subsequent encounter for closed fracture with delayed healing |
| S52.133H | Displaced fracture of neck of unspecified radius, subsequent encounter for open fracture type I or II with delayed healing |
| S52.133J | Displaced fracture of neck of unspecified radius, subsequent encounter for open fracture type IIIA, IIIB, or IIIC with delayed healing |
| S52.133K | Displaced fracture of neck of unspecified radius, subsequent encounter for closed fracture with nonunion |
| S52.133M | Displaced fracture of neck of unspecified radius, subsequent encounter for open fracture type I or II with nonunion |
| S52.133N | Displaced fracture of neck of unspecified radius, subsequent encounter for open fracture type IIIA, IIIB, or IIIC with nonunion |
| S52.133P | Displaced fracture of neck of unspecified radius, subsequent encounter for closed fracture with malunion |
| S52.133Q | Displaced fracture of neck of unspecified radius, subsequent encounter for open fracture type I or II with malunion |
| S52.133R | Displaced fracture of neck of unspecified radius, subsequent encounter for open fracture type IIIA, IIIB, or IIIC with malunion |
| S52.133S | Displaced fracture of neck of unspecified radius, sequela |
| S52.134 | Nondisplaced fracture of neck of right radius |
| S52.134A | Nondisplaced fracture of neck of right radius, initial encounter for closed fracture |
| S52.134B | Nondisplaced fracture of neck of right radius, initial encounter for open fracture type I or II |
| S52.134C | Nondisplaced fracture of neck of right radius, initial encounter for open fracture type IIIA, IIIB, or IIIC |
| S52.134D | Nondisplaced fracture of neck of right radius, subsequent encounter for closed fracture with routine healing |
| S52.134E | Nondisplaced fracture of neck of right radius, subsequent encounter for open fracture type I or II with routine healing |
| S52.134F | Nondisplaced fracture of neck of right radius, subsequent encounter for open fracture type IIIA, IIIB, or IIIC with routine healing |
| S52.134G | Nondisplaced fracture of neck of right radius, subsequent encounter for closed fracture with delayed healing |
| S52.134H | Nondisplaced fracture of neck of right radius, subsequent encounter for open fracture type I or II with delayed healing |
| S52.134J | Nondisplaced fracture of neck of right radius, subsequent encounter for open fracture type IIIA, IIIB, or IIIC with delayed healing |
| S52.134K | Nondisplaced fracture of neck of right radius, subsequent encounter for closed fracture with nonunion |
| S52.134M | Nondisplaced fracture of neck of right radius, subsequent encounter for open fracture type I or II with nonunion |
| S52.134N | Nondisplaced fracture of neck of right radius, subsequent encounter for open fracture type IIIA, IIIB, or IIIC with nonunion |
| S52.134P | Nondisplaced fracture of neck of right radius, subsequent encounter for closed fracture with malunion |
| S52.134Q | Nondisplaced fracture of neck of right radius, subsequent encounter for open fracture type I or II with malunion |
| S52.134R | Nondisplaced fracture of neck of right radius, subsequent encounter for open fracture type IIIA, IIIB, or IIIC with malunion |
| S52.134S | Nondisplaced fracture of neck of right radius, sequela |
| S52.135 | Nondisplaced fracture of neck of left radius |
| S52.135A | Nondisplaced fracture of neck of left radius, initial encounter for closed fracture |
| S52.135B | Nondisplaced fracture of neck of left radius, initial encounter for open fracture type I or II |
| S52.135C | Nondisplaced fracture of neck of left radius, initial encounter for open fracture type IIIA, IIIB, or IIIC |
| S52.135D | Nondisplaced fracture of neck of left radius, subsequent encounter for closed fracture with routine healing |
| S52.135E | Nondisplaced fracture of neck of left radius, subsequent encounter for open fracture type I or II with routine healing |
| S52.135F | Nondisplaced fracture of neck of left radius, subsequent encounter for open fracture type IIIA, IIIB, or IIIC with routine healing |
| S52.135G | Nondisplaced fracture of neck of left radius, subsequent encounter for closed fracture with delayed healing |
| S52.135H | Nondisplaced fracture of neck of left radius, subsequent encounter for open fracture type I or II with delayed healing |
| S52.135J | Nondisplaced fracture of neck of left radius, subsequent encounter for open fracture type IIIA, IIIB, or IIIC with delayed healing |
| S52.135K | Nondisplaced fracture of neck of left radius, subsequent encounter for closed fracture with nonunion |
| S52.135M | Nondisplaced fracture of neck of left radius, subsequent encounter for open fracture type I or II with nonunion |
| S52.135N | Nondisplaced fracture of neck of left radius, subsequent encounter for open fracture type IIIA, IIIB, or IIIC with nonunion |
| S52.135P | Nondisplaced fracture of neck of left radius, subsequent encounter for closed fracture with malunion |
| S52.135Q | Nondisplaced fracture of neck of left radius, subsequent encounter for open fracture type I or II with malunion |
| S52.135R | Nondisplaced fracture of neck of left radius, subsequent encounter for open fracture type IIIA, IIIB, or IIIC with malunion |
| S52.135S | Nondisplaced fracture of neck of left radius, sequela |
| S52.136 | Nondisplaced fracture of neck of unspecified radius |
| S52.136A | Nondisplaced fracture of neck of unspecified radius, initial encounter for closed fracture |
| S52.136B | Nondisplaced fracture of neck of unspecified radius, initial encounter for open fracture type I or II |
| S52.136C | Nondisplaced fracture of neck of unspecified radius, initial encounter for open fracture type IIIA, IIIB, or IIIC |
| S52.136D | Nondisplaced fracture of neck of unspecified radius, subsequent encounter for closed fracture with routine healing |
| S52.136E | Nondisplaced fracture of neck of unspecified radius, subsequent encounter for open fracture type I or II with routine healing |
| S52.136F | Nondisplaced fracture of neck of unspecified radius, subsequent encounter for open fracture type IIIA, IIIB, or IIIC with routine healing |
| S52.136G | Nondisplaced fracture of neck of unspecified radius, subsequent encounter for closed fracture with delayed healing |
| S52.136H | Nondisplaced fracture of neck of unspecified radius, subsequent encounter for open fracture type I or II with delayed healing |
| S52.136J | Nondisplaced fracture of neck of unspecified radius, subsequent encounter for open fracture type IIIA, IIIB, or IIIC with delayed healing |
| S52.136K | Nondisplaced fracture of neck of unspecified radius, subsequent encounter for closed fracture with nonunion |
| S52.136M | Nondisplaced fracture of neck of unspecified radius, subsequent encounter for open fracture type I or II with nonunion |
| S52.136N | Nondisplaced fracture of neck of unspecified radius, subsequent encounter for open fracture type IIIA, IIIB, or IIIC with nonunion |
| S52.136P | Nondisplaced fracture of neck of unspecified radius, subsequent encounter for closed fracture with malunion |
| S52.136Q | Nondisplaced fracture of neck of unspecified radius, subsequent encounter for open fracture type I or II with malunion |
| S52.136R | Nondisplaced fracture of neck of unspecified radius, subsequent encounter for open fracture type IIIA, IIIB, or IIIC with malunion |
| S52.136S | Nondisplaced fracture of neck of unspecified radius, sequela |
| S62.25 | Fracture of neck of first metacarpal bone |
| S62.251 | Displaced fracture of neck of first metacarpal bone, right hand |
| S62.251A | Displaced fracture of neck of first metacarpal bone, right hand, initial encounter for closed fracture |
| S62.251B | Displaced fracture of neck of first metacarpal bone, right hand, initial encounter for open fracture |
| S62.251D | Displaced fracture of neck of first metacarpal bone, right hand, subsequent encounter for fracture with routine healing |
| S62.251G | Displaced fracture of neck of first metacarpal bone, right hand, subsequent encounter for fracture with delayed healing |
| S62.251K | Displaced fracture of neck of first metacarpal bone, right hand, subsequent encounter for fracture with nonunion |
| S62.251P | Displaced fracture of neck of first metacarpal bone, right hand, subsequent encounter for fracture with malunion |
| S62.251S | Displaced fracture of neck of first metacarpal bone, right hand, sequela |
| S62.252 | Displaced fracture of neck of first metacarpal bone, left hand |
| S62.252A | Displaced fracture of neck of first metacarpal bone, left hand, initial encounter for closed fracture |
| S62.252B | Displaced fracture of neck of first metacarpal bone, left hand, initial encounter for open fracture |
| S62.252D | Displaced fracture of neck of first metacarpal bone, left hand, subsequent encounter for fracture with routine healing |
| S62.252G | Displaced fracture of neck of first metacarpal bone, left hand, subsequent encounter for fracture with delayed healing |
| S62.252K | Displaced fracture of neck of first metacarpal bone, left hand, subsequent encounter for fracture with nonunion |
| S62.252P | Displaced fracture of neck of first metacarpal bone, left hand, subsequent encounter for fracture with malunion |
| S62.252S | Displaced fracture of neck of first metacarpal bone, left hand, sequela |
| S62.253 | Displaced fracture of neck of first metacarpal bone, unspecified hand |
| S62.253A | Displaced fracture of neck of first metacarpal bone, unspecified hand, initial encounter for closed fracture |
| S62.253B | Displaced fracture of neck of first metacarpal bone, unspecified hand, initial encounter for open fracture |
| S62.253D | Displaced fracture of neck of first metacarpal bone, unspecified hand, subsequent encounter for fracture with routine healing |
| S62.253G | Displaced fracture of neck of first metacarpal bone, unspecified hand, subsequent encounter for fracture with delayed healing |
| S62.253K | Displaced fracture of neck of first metacarpal bone, unspecified hand, subsequent encounter for fracture with nonunion |
| S62.253P | Displaced fracture of neck of first metacarpal bone, unspecified hand, subsequent encounter for fracture with malunion |
| S62.253S | Displaced fracture of neck of first metacarpal bone, unspecified hand, sequela |
| S62.254 | Nondisplaced fracture of neck of first metacarpal bone, right hand |
| S62.254A | Nondisplaced fracture of neck of first metacarpal bone, right hand, initial encounter for closed fracture |
| S62.254B | Nondisplaced fracture of neck of first metacarpal bone, right hand, initial encounter for open fracture |
| S62.254D | Nondisplaced fracture of neck of first metacarpal bone, right hand, subsequent encounter for fracture with routine healing |
| S62.254G | Nondisplaced fracture of neck of first metacarpal bone, right hand, subsequent encounter for fracture with delayed healing |
| S62.254K | Nondisplaced fracture of neck of first metacarpal bone, right hand, subsequent encounter for fracture with nonunion |
| S62.254P | Nondisplaced fracture of neck of first metacarpal bone, right hand, subsequent encounter for fracture with malunion |
| S62.254S | Nondisplaced fracture of neck of first metacarpal bone, right hand, sequela |
| S62.255 | Nondisplaced fracture of neck of first metacarpal bone, left hand |
| S62.255A | Nondisplaced fracture of neck of first metacarpal bone, left hand, initial encounter for closed fracture |
| S62.255B | Nondisplaced fracture of neck of first metacarpal bone, left hand, initial encounter for open fracture |
| S62.255D | Nondisplaced fracture of neck of first metacarpal bone, left hand, subsequent encounter for fracture with routine healing |
| S62.255G | Nondisplaced fracture of neck of first metacarpal bone, left hand, subsequent encounter for fracture with delayed healing |
| S62.255K | Nondisplaced fracture of neck of first metacarpal bone, left hand, subsequent encounter for fracture with nonunion |
| S62.255P | Nondisplaced fracture of neck of first metacarpal bone, left hand, subsequent encounter for fracture with malunion |
| S62.255S | Nondisplaced fracture of neck of first metacarpal bone, left hand, sequela |
| S62.256 | Nondisplaced fracture of neck of first metacarpal bone, unspecified hand |
| S62.256A | Nondisplaced fracture of neck of first metacarpal bone, unspecified hand, initial encounter for closed fracture |
| S62.256B | Nondisplaced fracture of neck of first metacarpal bone, unspecified hand, initial encounter for open fracture |
| S62.256D | Nondisplaced fracture of neck of first metacarpal bone, unspecified hand, subsequent encounter for fracture with routine healing |
| S62.256G | Nondisplaced fracture of neck of first metacarpal bone, unspecified hand, subsequent encounter for fracture with delayed healing |
| S62.256K | Nondisplaced fracture of neck of first metacarpal bone, unspecified hand, subsequent encounter for fracture with nonunion |
| S62.256P | Nondisplaced fracture of neck of first metacarpal bone, unspecified hand, subsequent encounter for fracture with malunion |
| S62.256S | Nondisplaced fracture of neck of first metacarpal bone, unspecified hand, sequela |
| S62.33 | Displaced fracture of neck of other metacarpal bone |
| S62.330 | Displaced fracture of neck of second metacarpal bone, right hand |
| S62.330A | Displaced fracture of neck of second metacarpal bone, right hand, initial encounter for closed fracture |
| S62.330B | Displaced fracture of neck of second metacarpal bone, right hand, initial encounter for open fracture |
| S62.330D | Displaced fracture of neck of second metacarpal bone, right hand, subsequent encounter for fracture with routine healing |
| S62.330G | Displaced fracture of neck of second metacarpal bone, right hand, subsequent encounter for fracture with delayed healing |
| S62.330K | Displaced fracture of neck of second metacarpal bone, right hand, subsequent encounter for fracture with nonunion |
| S62.330P | Displaced fracture of neck of second metacarpal bone, right hand, subsequent encounter for fracture with malunion |
| S62.330S | Displaced fracture of neck of second metacarpal bone, right hand, sequela |
| S62.331 | Displaced fracture of neck of second metacarpal bone, left hand |
| S62.331A | Displaced fracture of neck of second metacarpal bone, left hand, initial encounter for closed fracture |
| S62.331B | Displaced fracture of neck of second metacarpal bone, left hand, initial encounter for open fracture |
| S62.331D | Displaced fracture of neck of second metacarpal bone, left hand, subsequent encounter for fracture with routine healing |
| S62.331G | Displaced fracture of neck of second metacarpal bone, left hand, subsequent encounter for fracture with delayed healing |
| S62.331K | Displaced fracture of neck of second metacarpal bone, left hand, subsequent encounter for fracture with nonunion |
| S62.331P | Displaced fracture of neck of second metacarpal bone, left hand, subsequent encounter for fracture with malunion |
| S62.331S | Displaced fracture of neck of second metacarpal bone, left hand, sequela |
| S62.332 | Displaced fracture of neck of third metacarpal bone, right hand |
| S62.332A | Displaced fracture of neck of third metacarpal bone, right hand, initial encounter for closed fracture |
| S62.332B | Displaced fracture of neck of third metacarpal bone, right hand, initial encounter for open fracture |
| S62.332D | Displaced fracture of neck of third metacarpal bone, right hand, subsequent encounter for fracture with routine healing |
| S62.332G | Displaced fracture of neck of third metacarpal bone, right hand, subsequent encounter for fracture with delayed healing |
| S62.332K | Displaced fracture of neck of third metacarpal bone, right hand, subsequent encounter for fracture with nonunion |
| S62.332P | Displaced fracture of neck of third metacarpal bone, right hand, subsequent encounter for fracture with malunion |
| S62.332S | Displaced fracture of neck of third metacarpal bone, right hand, sequela |
| S62.333 | Displaced fracture of neck of third metacarpal bone, left hand |
| S62.333A | Displaced fracture of neck of third metacarpal bone, left hand, initial encounter for closed fracture |
| S62.333B | Displaced fracture of neck of third metacarpal bone, left hand, initial encounter for open fracture |
| S62.333D | Displaced fracture of neck of third metacarpal bone, left hand, subsequent encounter for fracture with routine healing |
| S62.333G | Displaced fracture of neck of third metacarpal bone, left hand, subsequent encounter for fracture with delayed healing |
| S62.333K | Displaced fracture of neck of third metacarpal bone, left hand, subsequent encounter for fracture with nonunion |
| S62.333P | Displaced fracture of neck of third metacarpal bone, left hand, subsequent encounter for fracture with malunion |
| S62.333S | Displaced fracture of neck of third metacarpal bone, left hand, sequela |
| S62.334 | Displaced fracture of neck of fourth metacarpal bone, right hand |
| S62.334A | Displaced fracture of neck of fourth metacarpal bone, right hand, initial encounter for closed fracture |
| S62.334B | Displaced fracture of neck of fourth metacarpal bone, right hand, initial encounter for open fracture |
| S62.334D | Displaced fracture of neck of fourth metacarpal bone, right hand, subsequent encounter for fracture with routine healing |
| S62.334G | Displaced fracture of neck of fourth metacarpal bone, right hand, subsequent encounter for fracture with delayed healing |
| S62.334K | Displaced fracture of neck of fourth metacarpal bone, right hand, subsequent encounter for fracture with nonunion |
| S62.334P | Displaced fracture of neck of fourth metacarpal bone, right hand, subsequent encounter for fracture with malunion |
| S62.334S | Displaced fracture of neck of fourth metacarpal bone, right hand, sequela |
| S62.335 | Displaced fracture of neck of fourth metacarpal bone, left hand |
| S62.335A | Displaced fracture of neck of fourth metacarpal bone, left hand, initial encounter for closed fracture |
| S62.335B | Displaced fracture of neck of fourth metacarpal bone, left hand, initial encounter for open fracture |
| S62.335D | Displaced fracture of neck of fourth metacarpal bone, left hand, subsequent encounter for fracture with routine healing |
| S62.335G | Displaced fracture of neck of fourth metacarpal bone, left hand, subsequent encounter for fracture with delayed healing |
| S62.335K | Displaced fracture of neck of fourth metacarpal bone, left hand, subsequent encounter for fracture with nonunion |
| S62.335P | Displaced fracture of neck of fourth metacarpal bone, left hand, subsequent encounter for fracture with malunion |
| S62.335S | Displaced fracture of neck of fourth metacarpal bone, left hand, sequela |
| S62.336 | Displaced fracture of neck of fifth metacarpal bone, right hand |
| S62.336A | Displaced fracture of neck of fifth metacarpal bone, right hand, initial encounter for closed fracture |
| S62.336B | Displaced fracture of neck of fifth metacarpal bone, right hand, initial encounter for open fracture |
| S62.336D | Displaced fracture of neck of fifth metacarpal bone, right hand, subsequent encounter for fracture with routine healing |
| S62.336G | Displaced fracture of neck of fifth metacarpal bone, right hand, subsequent encounter for fracture with delayed healing |
| S62.336K | Displaced fracture of neck of fifth metacarpal bone, right hand, subsequent encounter for fracture with nonunion |
| S62.336P | Displaced fracture of neck of fifth metacarpal bone, right hand, subsequent encounter for fracture with malunion |
| S62.336S | Displaced fracture of neck of fifth metacarpal bone, right hand, sequela |
| S62.337 | Displaced fracture of neck of fifth metacarpal bone, left hand |
| S62.337A | Displaced fracture of neck of fifth metacarpal bone, left hand, initial encounter for closed fracture |
| S62.337B | Displaced fracture of neck of fifth metacarpal bone, left hand, initial encounter for open fracture |
| S62.337D | Displaced fracture of neck of fifth metacarpal bone, left hand, subsequent encounter for fracture with routine healing |
| S62.337G | Displaced fracture of neck of fifth metacarpal bone, left hand, subsequent encounter for fracture with delayed healing |
| S62.337K | Displaced fracture of neck of fifth metacarpal bone, left hand, subsequent encounter for fracture with nonunion |
| S62.337P | Displaced fracture of neck of fifth metacarpal bone, left hand, subsequent encounter for fracture with malunion |
| S62.337S | Displaced fracture of neck of fifth metacarpal bone, left hand, sequela |
| S62.338 | Displaced fracture of neck of other metacarpal bone |
| S62.338A | Displaced fracture of neck of other metacarpal bone, initial encounter for closed fracture |
| S62.338B | Displaced fracture of neck of other metacarpal bone, initial encounter for open fracture |
| S62.338D | Displaced fracture of neck of other metacarpal bone, subsequent encounter for fracture with routine healing |
| S62.338G | Displaced fracture of neck of other metacarpal bone, subsequent encounter for fracture with delayed healing |
| S62.338K | Displaced fracture of neck of other metacarpal bone, subsequent encounter for fracture with nonunion |
| S62.338P | Displaced fracture of neck of other metacarpal bone, subsequent encounter for fracture with malunion |
| S62.338S | Displaced fracture of neck of other metacarpal bone, sequela |
| S62.339 | Displaced fracture of neck of unspecified metacarpal bone |
| S62.339A | Displaced fracture of neck of unspecified metacarpal bone, initial encounter for closed fracture |
| S62.339B | Displaced fracture of neck of unspecified metacarpal bone, initial encounter for open fracture |
| S62.339D | Displaced fracture of neck of unspecified metacarpal bone, subsequent encounter for fracture with routine healing |
| S62.339G | Displaced fracture of neck of unspecified metacarpal bone, subsequent encounter for fracture with delayed healing |
| S62.339K | Displaced fracture of neck of unspecified metacarpal bone, subsequent encounter for fracture with nonunion |
| S62.339P | Displaced fracture of neck of unspecified metacarpal bone, subsequent encounter for fracture with malunion |
| S62.339S | Displaced fracture of neck of unspecified metacarpal bone, sequela |
| S62.36 | Nondisplaced fracture of neck of other metacarpal bone |
| S62.360 | Nondisplaced fracture of neck of second metacarpal bone, right hand |
| S62.360A | Nondisplaced fracture of neck of second metacarpal bone, right hand, initial encounter for closed fracture |
| S62.360B | Nondisplaced fracture of neck of second metacarpal bone, right hand, initial encounter for open fracture |
| S62.360D | Nondisplaced fracture of neck of second metacarpal bone, right hand, subsequent encounter for fracture with routine healing |
| S62.360G | Nondisplaced fracture of neck of second metacarpal bone, right hand, subsequent encounter for fracture with delayed healing |
| S62.360K | Nondisplaced fracture of neck of second metacarpal bone, right hand, subsequent encounter for fracture with nonunion |
| S62.360P | Nondisplaced fracture of neck of second metacarpal bone, right hand, subsequent encounter for fracture with malunion |
| S62.360S | Nondisplaced fracture of neck of second metacarpal bone, right hand, sequela |
| S62.361 | Nondisplaced fracture of neck of second metacarpal bone, left hand |
| S62.361A | Nondisplaced fracture of neck of second metacarpal bone, left hand, initial encounter for closed fracture |
| S62.361B | Nondisplaced fracture of neck of second metacarpal bone, left hand, initial encounter for open fracture |
| S62.361D | Nondisplaced fracture of neck of second metacarpal bone, left hand, subsequent encounter for fracture with routine healing |
| S62.361G | Nondisplaced fracture of neck of second metacarpal bone, left hand, subsequent encounter for fracture with delayed healing |
| S62.361K | Nondisplaced fracture of neck of second metacarpal bone, left hand, subsequent encounter for fracture with nonunion |
| S62.361P | Nondisplaced fracture of neck of second metacarpal bone, left hand, subsequent encounter for fracture with malunion |
| S62.361S | Nondisplaced fracture of neck of second metacarpal bone, left hand, sequela |
| S62.362 | Nondisplaced fracture of neck of third metacarpal bone, right hand |
| S62.362A | Nondisplaced fracture of neck of third metacarpal bone, right hand, initial encounter for closed fracture |
| S62.362B | Nondisplaced fracture of neck of third metacarpal bone, right hand, initial encounter for open fracture |
| S62.362D | Nondisplaced fracture of neck of third metacarpal bone, right hand, subsequent encounter for fracture with routine healing |
| S62.362G | Nondisplaced fracture of neck of third metacarpal bone, right hand, subsequent encounter for fracture with delayed healing |
| S62.362K | Nondisplaced fracture of neck of third metacarpal bone, right hand, subsequent encounter for fracture with nonunion |
| S62.362P | Nondisplaced fracture of neck of third metacarpal bone, right hand, subsequent encounter for fracture with malunion |
| S62.362S | Nondisplaced fracture of neck of third metacarpal bone, right hand, sequela |
| S62.363 | Nondisplaced fracture of neck of third metacarpal bone, left hand |
| S62.363A | Nondisplaced fracture of neck of third metacarpal bone, left hand, initial encounter for closed fracture |
| S62.363B | Nondisplaced fracture of neck of third metacarpal bone, left hand, initial encounter for open fracture |
| S62.363D | Nondisplaced fracture of neck of third metacarpal bone, left hand, subsequent encounter for fracture with routine healing |
| S62.363G | Nondisplaced fracture of neck of third metacarpal bone, left hand, subsequent encounter for fracture with delayed healing |
| S62.363K | Nondisplaced fracture of neck of third metacarpal bone, left hand, subsequent encounter for fracture with nonunion |
| S62.363P | Nondisplaced fracture of neck of third metacarpal bone, left hand, subsequent encounter for fracture with malunion |
| S62.363S | Nondisplaced fracture of neck of third metacarpal bone, left hand, sequela |
| S62.364 | Nondisplaced fracture of neck of fourth metacarpal bone, right hand |
| S62.364A | Nondisplaced fracture of neck of fourth metacarpal bone, right hand, initial encounter for closed fracture |
| S62.364B | Nondisplaced fracture of neck of fourth metacarpal bone, right hand, initial encounter for open fracture |
| S62.364D | Nondisplaced fracture of neck of fourth metacarpal bone, right hand, subsequent encounter for fracture with routine healing |
| S62.364G | Nondisplaced fracture of neck of fourth metacarpal bone, right hand, subsequent encounter for fracture with delayed healing |
| S62.364K | Nondisplaced fracture of neck of fourth metacarpal bone, right hand, subsequent encounter for fracture with nonunion |
| S62.364P | Nondisplaced fracture of neck of fourth metacarpal bone, right hand, subsequent encounter for fracture with malunion |
| S62.364S | Nondisplaced fracture of neck of fourth metacarpal bone, right hand, sequela |
| S62.365 | Nondisplaced fracture of neck of fourth metacarpal bone, left hand |
| S62.365A | Nondisplaced fracture of neck of fourth metacarpal bone, left hand, initial encounter for closed fracture |
| S62.365B | Nondisplaced fracture of neck of fourth metacarpal bone, left hand, initial encounter for open fracture |
| S62.365D | Nondisplaced fracture of neck of fourth metacarpal bone, left hand, subsequent encounter for fracture with routine healing |
| S62.365G | Nondisplaced fracture of neck of fourth metacarpal bone, left hand, subsequent encounter for fracture with delayed healing |
| S62.365K | Nondisplaced fracture of neck of fourth metacarpal bone, left hand, subsequent encounter for fracture with nonunion |
| S62.365P | Nondisplaced fracture of neck of fourth metacarpal bone, left hand, subsequent encounter for fracture with malunion |
| S62.365S | Nondisplaced fracture of neck of fourth metacarpal bone, left hand, sequela |
| S62.366 | Nondisplaced fracture of neck of fifth metacarpal bone, right hand |
| S62.366A | Nondisplaced fracture of neck of fifth metacarpal bone, right hand, initial encounter for closed fracture |
| S62.366B | Nondisplaced fracture of neck of fifth metacarpal bone, right hand, initial encounter for open fracture |
| S62.366D | Nondisplaced fracture of neck of fifth metacarpal bone, right hand, subsequent encounter for fracture with routine healing |
| S62.366G | Nondisplaced fracture of neck of fifth metacarpal bone, right hand, subsequent encounter for fracture with delayed healing |
| S62.366K | Nondisplaced fracture of neck of fifth metacarpal bone, right hand, subsequent encounter for fracture with nonunion |
| S62.366P | Nondisplaced fracture of neck of fifth metacarpal bone, right hand, subsequent encounter for fracture with malunion |
| S62.366S | Nondisplaced fracture of neck of fifth metacarpal bone, right hand, sequela |
| S62.367 | Nondisplaced fracture of neck of fifth metacarpal bone, left hand |
| S62.367A | Nondisplaced fracture of neck of fifth metacarpal bone, left hand, initial encounter for closed fracture |
| S62.367B | Nondisplaced fracture of neck of fifth metacarpal bone, left hand, initial encounter for open fracture |
| S62.367D | Nondisplaced fracture of neck of fifth metacarpal bone, left hand, subsequent encounter for fracture with routine healing |
| S62.367G | Nondisplaced fracture of neck of fifth metacarpal bone, left hand, subsequent encounter for fracture with delayed healing |
| S62.367K | Nondisplaced fracture of neck of fifth metacarpal bone, left hand, subsequent encounter for fracture with nonunion |
| S62.367P | Nondisplaced fracture of neck of fifth metacarpal bone, left hand, subsequent encounter for fracture with malunion |
| S62.367S | Nondisplaced fracture of neck of fifth metacarpal bone, left hand, sequela |
| S62.368 | Nondisplaced fracture of neck of other metacarpal bone |
| S62.368A | Nondisplaced fracture of neck of other metacarpal bone, initial encounter for closed fracture |
| S62.368B | Nondisplaced fracture of neck of other metacarpal bone, initial encounter for open fracture |
| S62.368D | Nondisplaced fracture of neck of other metacarpal bone, subsequent encounter for fracture with routine healing |
| S62.368G | Nondisplaced fracture of neck of other metacarpal bone, subsequent encounter for fracture with delayed healing |
| S62.368K | Nondisplaced fracture of neck of other metacarpal bone, subsequent encounter for fracture with nonunion |
| S62.368P | Nondisplaced fracture of neck of other metacarpal bone, subsequent encounter for fracture with malunion |
| S62.368S | Nondisplaced fracture of neck of other metacarpal bone, sequela |
| S62.369 | Nondisplaced fracture of neck of unspecified metacarpal bone |
| S62.369A | Nondisplaced fracture of neck of unspecified metacarpal bone, initial encounter for closed fracture |
| S62.369B | Nondisplaced fracture of neck of unspecified metacarpal bone, initial encounter for open fracture |
| S62.369D | Nondisplaced fracture of neck of unspecified metacarpal bone, subsequent encounter for fracture with routine healing |
| S62.369G | Nondisplaced fracture of neck of unspecified metacarpal bone, subsequent encounter for fracture with delayed healing |
| S62.369K | Nondisplaced fracture of neck of unspecified metacarpal bone, subsequent encounter for fracture with nonunion |
| S62.369P | Nondisplaced fracture of neck of unspecified metacarpal bone, subsequent encounter for fracture with malunion |
| S62.369S | Nondisplaced fracture of neck of unspecified metacarpal bone, sequela |
| S72.0 | Fracture of head and neck of femur |
| S72.00 | Fracture of unspecified part of neck of femur |
| S72.001 | Fracture of unspecified part of neck of right femur |
| S72.001A | Fracture of unspecified part of neck of right femur, initial encounter for closed fracture |
| S72.001B | Fracture of unspecified part of neck of right femur, initial encounter for open fracture type I or II |
| S72.001C | Fracture of unspecified part of neck of right femur, initial encounter for open fracture type IIIA, IIIB, or IIIC |
| S72.001D | Fracture of unspecified part of neck of right femur, subsequent encounter for closed fracture with routine healing |
| S72.001E | Fracture of unspecified part of neck of right femur, subsequent encounter for open fracture type I or II with routine healing |
| S72.001F | Fracture of unspecified part of neck of right femur, subsequent encounter for open fracture type IIIA, IIIB, or IIIC with routine healing |
| S72.001G | Fracture of unspecified part of neck of right femur, subsequent encounter for closed fracture with delayed healing |
| S72.001H | Fracture of unspecified part of neck of right femur, subsequent encounter for open fracture type I or II with delayed healing |
| S72.001J | Fracture of unspecified part of neck of right femur, subsequent encounter for open fracture type IIIA, IIIB, or IIIC with delayed healing |
| S72.001K | Fracture of unspecified part of neck of right femur, subsequent encounter for closed fracture with nonunion |
| S72.001M | Fracture of unspecified part of neck of right femur, subsequent encounter for open fracture type I or II with nonunion |
| S72.001N | Fracture of unspecified part of neck of right femur, subsequent encounter for open fracture type IIIA, IIIB, or IIIC with nonunion |
| S72.001P | Fracture of unspecified part of neck of right femur, subsequent encounter for closed fracture with malunion |
| S72.001Q | Fracture of unspecified part of neck of right femur, subsequent encounter for open fracture type I or II with malunion |
| S72.001R | Fracture of unspecified part of neck of right femur, subsequent encounter for open fracture type IIIA, IIIB, or IIIC with malunion |
| S72.001S | Fracture of unspecified part of neck of right femur, sequela |
| S72.002 | Fracture of unspecified part of neck of left femur |
| S72.002A | Fracture of unspecified part of neck of left femur, initial encounter for closed fracture |
| S72.002B | Fracture of unspecified part of neck of left femur, initial encounter for open fracture type I or II |
| S72.002C | Fracture of unspecified part of neck of left femur, initial encounter for open fracture type IIIA, IIIB, or IIIC |
| S72.002D | Fracture of unspecified part of neck of left femur, subsequent encounter for closed fracture with routine healing |
| S72.002E | Fracture of unspecified part of neck of left femur, subsequent encounter for open fracture type I or II with routine healing |
| S72.002F | Fracture of unspecified part of neck of left femur, subsequent encounter for open fracture type IIIA, IIIB, or IIIC with routine healing |
| S72.002G | Fracture of unspecified part of neck of left femur, subsequent encounter for closed fracture with delayed healing |
| S72.002H | Fracture of unspecified part of neck of left femur, subsequent encounter for open fracture type I or II with delayed healing |
| S72.002J | Fracture of unspecified part of neck of left femur, subsequent encounter for open fracture type IIIA, IIIB, or IIIC with delayed healing |
| S72.002K | Fracture of unspecified part of neck of left femur, subsequent encounter for closed fracture with nonunion |
| S72.002M | Fracture of unspecified part of neck of left femur, subsequent encounter for open fracture type I or II with nonunion |
| S72.002N | Fracture of unspecified part of neck of left femur, subsequent encounter for open fracture type IIIA, IIIB, or IIIC with nonunion |
| S72.002P | Fracture of unspecified part of neck of left femur, subsequent encounter for closed fracture with malunion |
| S72.002Q | Fracture of unspecified part of neck of left femur, subsequent encounter for open fracture type I or II with malunion |
| S72.002R | Fracture of unspecified part of neck of left femur, subsequent encounter for open fracture type IIIA, IIIB, or IIIC with malunion |
| S72.002S | Fracture of unspecified part of neck of left femur, sequela |
| S72.009 | Fracture of unspecified part of neck of unspecified femur |
| S72.009A | Fracture of unspecified part of neck of unspecified femur, initial encounter for closed fracture |
| S72.009B | Fracture of unspecified part of neck of unspecified femur, initial encounter for open fracture type I or II |
| S72.009C | Fracture of unspecified part of neck of unspecified femur, initial encounter for open fracture type IIIA, IIIB, or IIIC |
| S72.009D | Fracture of unspecified part of neck of unspecified femur, subsequent encounter for closed fracture with routine healing |
| S72.009E | Fracture of unspecified part of neck of unspecified femur, subsequent encounter for open fracture type I or II with routine healing |
| S72.009F | Fracture of unspecified part of neck of unspecified femur, subsequent encounter for open fracture type IIIA, IIIB, or IIIC with routine healing |
| S72.009G | Fracture of unspecified part of neck of unspecified femur, subsequent encounter for closed fracture with delayed healing |
| S72.009H | Fracture of unspecified part of neck of unspecified femur, subsequent encounter for open fracture type I or II with delayed healing |
| S72.009J | Fracture of unspecified part of neck of unspecified femur, subsequent encounter for open fracture type IIIA, IIIB, or IIIC with delayed healing |
| S72.009K | Fracture of unspecified part of neck of unspecified femur, subsequent encounter for closed fracture with nonunion |
| S72.009M | Fracture of unspecified part of neck of unspecified femur, subsequent encounter for open fracture type I or II with nonunion |
| S72.009N | Fracture of unspecified part of neck of unspecified femur, subsequent encounter for open fracture type IIIA, IIIB, or IIIC with nonunion |
| S72.009P | Fracture of unspecified part of neck of unspecified femur, subsequent encounter for closed fracture with malunion |
| S72.009Q | Fracture of unspecified part of neck of unspecified femur, subsequent encounter for open fracture type I or II with malunion |
| S72.009R | Fracture of unspecified part of neck of unspecified femur, subsequent encounter for open fracture type IIIA, IIIB, or IIIC with malunion |
| S72.009S | Fracture of unspecified part of neck of unspecified femur, sequela |
| S72.03 | Midcervical fracture of femur |
| S72.031 | Displaced midcervical fracture of right femur |
| S72.031A | Displaced midcervical fracture of right femur, initial encounter for closed fracture |
| S72.031B | Displaced midcervical fracture of right femur, initial encounter for open fracture type I or II |
| S72.031C | Displaced midcervical fracture of right femur, initial encounter for open fracture type IIIA, IIIB, or IIIC |
| S72.031D | Displaced midcervical fracture of right femur, subsequent encounter for closed fracture with routine healing |
| S72.031E | Displaced midcervical fracture of right femur, subsequent encounter for open fracture type I or II with routine healing |
| S72.031F | Displaced midcervical fracture of right femur, subsequent encounter for open fracture type IIIA, IIIB, or IIIC with routine healing |
| S72.031G | Displaced midcervical fracture of right femur, subsequent encounter for closed fracture with delayed healing |
| S72.031H | Displaced midcervical fracture of right femur, subsequent encounter for open fracture type I or II with delayed healing |
| S72.031J | Displaced midcervical fracture of right femur, subsequent encounter for open fracture type IIIA, IIIB, or IIIC with delayed healing |
| S72.031K | Displaced midcervical fracture of right femur, subsequent encounter for closed fracture with nonunion |
| S72.031M | Displaced midcervical fracture of right femur, subsequent encounter for open fracture type I or II with nonunion |
| S72.031N | Displaced midcervical fracture of right femur, subsequent encounter for open fracture type IIIA, IIIB, or IIIC with nonunion |
| S72.031P | Displaced midcervical fracture of right femur, subsequent encounter for closed fracture with malunion |
| S72.031Q | Displaced midcervical fracture of right femur, subsequent encounter for open fracture type I or II with malunion |
| S72.031R | Displaced midcervical fracture of right femur, subsequent encounter for open fracture type IIIA, IIIB, or IIIC with malunion |
| S72.031S | Displaced midcervical fracture of right femur, sequela |
| S72.032 | Displaced midcervical fracture of left femur |
| S72.032A | Displaced midcervical fracture of left femur, initial encounter for closed fracture |
| S72.032B | Displaced midcervical fracture of left femur, initial encounter for open fracture type I or II |
| S72.032C | Displaced midcervical fracture of left femur, initial encounter for open fracture type IIIA, IIIB, or IIIC |
| S72.032D | Displaced midcervical fracture of left femur, subsequent encounter for closed fracture with routine healing |
| S72.032E | Displaced midcervical fracture of left femur, subsequent encounter for open fracture type I or II with routine healing |
| S72.032F | Displaced midcervical fracture of left femur, subsequent encounter for open fracture type IIIA, IIIB, or IIIC with routine healing |
| S72.032G | Displaced midcervical fracture of left femur, subsequent encounter for closed fracture with delayed healing |
| S72.032H | Displaced midcervical fracture of left femur, subsequent encounter for open fracture type I or II with delayed healing |
| S72.032J | Displaced midcervical fracture of left femur, subsequent encounter for open fracture type IIIA, IIIB, or IIIC with delayed healing |
| S72.032K | Displaced midcervical fracture of left femur, subsequent encounter for closed fracture with nonunion |
| S72.032M | Displaced midcervical fracture of left femur, subsequent encounter for open fracture type I or II with nonunion |
| S72.032N | Displaced midcervical fracture of left femur, subsequent encounter for open fracture type IIIA, IIIB, or IIIC with nonunion |
| S72.032P | Displaced midcervical fracture of left femur, subsequent encounter for closed fracture with malunion |
| S72.032Q | Displaced midcervical fracture of left femur, subsequent encounter for open fracture type I or II with malunion |
| S72.032R | Displaced midcervical fracture of left femur, subsequent encounter for open fracture type IIIA, IIIB, or IIIC with malunion |
| S72.032S | Displaced midcervical fracture of left femur, sequela |
| S72.033 | Displaced midcervical fracture of unspecified femur |
| S72.033A | Displaced midcervical fracture of unspecified femur, initial encounter for closed fracture |
| S72.033B | Displaced midcervical fracture of unspecified femur, initial encounter for open fracture type I or II |
| S72.033C | Displaced midcervical fracture of unspecified femur, initial encounter for open fracture type IIIA, IIIB, or IIIC |
| S72.033D | Displaced midcervical fracture of unspecified femur, subsequent encounter for closed fracture with routine healing |
| S72.033E | Displaced midcervical fracture of unspecified femur, subsequent encounter for open fracture type I or II with routine healing |
| S72.033F | Displaced midcervical fracture of unspecified femur, subsequent encounter for open fracture type IIIA, IIIB, or IIIC with routine healing |
| S72.033G | Displaced midcervical fracture of unspecified femur, subsequent encounter for closed fracture with delayed healing |
| S72.033H | Displaced midcervical fracture of unspecified femur, subsequent encounter for open fracture type I or II with delayed healing |
| S72.033J | Displaced midcervical fracture of unspecified femur, subsequent encounter for open fracture type IIIA, IIIB, or IIIC with delayed healing |
| S72.033K | Displaced midcervical fracture of unspecified femur, subsequent encounter for closed fracture with nonunion |
| S72.033M | Displaced midcervical fracture of unspecified femur, subsequent encounter for open fracture type I or II with nonunion |
| S72.033N | Displaced midcervical fracture of unspecified femur, subsequent encounter for open fracture type IIIA, IIIB, or IIIC with nonunion |
| S72.033P | Displaced midcervical fracture of unspecified femur, subsequent encounter for closed fracture with malunion |
| S72.033Q | Displaced midcervical fracture of unspecified femur, subsequent encounter for open fracture type I or II with malunion |
| S72.033R | Displaced midcervical fracture of unspecified femur, subsequent encounter for open fracture type IIIA, IIIB, or IIIC with malunion |
| S72.033S | Displaced midcervical fracture of unspecified femur, sequela |
| S72.034 | Nondisplaced midcervical fracture of right femur |
| S72.034A | Nondisplaced midcervical fracture of right femur, initial encounter for closed fracture |
| S72.034B | Nondisplaced midcervical fracture of right femur, initial encounter for open fracture type I or II |
| S72.034C | Nondisplaced midcervical fracture of right femur, initial encounter for open fracture type IIIA, IIIB, or IIIC |
| S72.034D | Nondisplaced midcervical fracture of right femur, subsequent encounter for closed fracture with routine healing |
| S72.034E | Nondisplaced midcervical fracture of right femur, subsequent encounter for open fracture type I or II with routine healing |
| S72.034F | Nondisplaced midcervical fracture of right femur, subsequent encounter for open fracture type IIIA, IIIB, or IIIC with routine healing |
| S72.034G | Nondisplaced midcervical fracture of right femur, subsequent encounter for closed fracture with delayed healing |
| S72.034H | Nondisplaced midcervical fracture of right femur, subsequent encounter for open fracture type I or II with delayed healing |
| S72.034J | Nondisplaced midcervical fracture of right femur, subsequent encounter for open fracture type IIIA, IIIB, or IIIC with delayed healing |
| S72.034K | Nondisplaced midcervical fracture of right femur, subsequent encounter for closed fracture with nonunion |
| S72.034M | Nondisplaced midcervical fracture of right femur, subsequent encounter for open fracture type I or II with nonunion |
| S72.034N | Nondisplaced midcervical fracture of right femur, subsequent encounter for open fracture type IIIA, IIIB, or IIIC with nonunion |
| S72.034P | Nondisplaced midcervical fracture of right femur, subsequent encounter for closed fracture with malunion |
| S72.034Q | Nondisplaced midcervical fracture of right femur, subsequent encounter for open fracture type I or II with malunion |
| S72.034R | Nondisplaced midcervical fracture of right femur, subsequent encounter for open fracture type IIIA, IIIB, or IIIC with malunion |
| S72.034S | Nondisplaced midcervical fracture of right femur, sequela |
| S72.035 | Nondisplaced midcervical fracture of left femur |
| S72.035A | Nondisplaced midcervical fracture of left femur, initial encounter for closed fracture |
| S72.035B | Nondisplaced midcervical fracture of left femur, initial encounter for open fracture type I or II |
| S72.035C | Nondisplaced midcervical fracture of left femur, initial encounter for open fracture type IIIA, IIIB, or IIIC |
| S72.035D | Nondisplaced midcervical fracture of left femur, subsequent encounter for closed fracture with routine healing |
| S72.035E | Nondisplaced midcervical fracture of left femur, subsequent encounter for open fracture type I or II with routine healing |
| S72.035F | Nondisplaced midcervical fracture of left femur, subsequent encounter for open fracture type IIIA, IIIB, or IIIC with routine healing |
| S72.035G | Nondisplaced midcervical fracture of left femur, subsequent encounter for closed fracture with delayed healing |
| S72.035H | Nondisplaced midcervical fracture of left femur, subsequent encounter for open fracture type I or II with delayed healing |
| S72.035J | Nondisplaced midcervical fracture of left femur, subsequent encounter for open fracture type IIIA, IIIB, or IIIC with delayed healing |
| S72.035K | Nondisplaced midcervical fracture of left femur, subsequent encounter for closed fracture with nonunion |
| S72.035M | Nondisplaced midcervical fracture of left femur, subsequent encounter for open fracture type I or II with nonunion |
| S72.035N | Nondisplaced midcervical fracture of left femur, subsequent encounter for open fracture type IIIA, IIIB, or IIIC with nonunion |
| S72.035P | Nondisplaced midcervical fracture of left femur, subsequent encounter for closed fracture with malunion |
| S72.035Q | Nondisplaced midcervical fracture of left femur, subsequent encounter for open fracture type I or II with malunion |
| S72.035R | Nondisplaced midcervical fracture of left femur, subsequent encounter for open fracture type IIIA, IIIB, or IIIC with malunion |
| S72.035S | Nondisplaced midcervical fracture of left femur, sequela |
| S72.036 | Nondisplaced midcervical fracture of unspecified femur |
| S72.036A | Nondisplaced midcervical fracture of unspecified femur, initial encounter for closed fracture |
| S72.036B | Nondisplaced midcervical fracture of unspecified femur, initial encounter for open fracture type I or II |
| S72.036C | Nondisplaced midcervical fracture of unspecified femur, initial encounter for open fracture type IIIA, IIIB, or IIIC |
| S72.036D | Nondisplaced midcervical fracture of unspecified femur, subsequent encounter for closed fracture with routine healing |
| S72.036E | Nondisplaced midcervical fracture of unspecified femur, subsequent encounter for open fracture type I or II with routine healing |
| S72.036F | Nondisplaced midcervical fracture of unspecified femur, subsequent encounter for open fracture type IIIA, IIIB, or IIIC with routine healing |
| S72.036G | Nondisplaced midcervical fracture of unspecified femur, subsequent encounter for closed fracture with delayed healing |
| S72.036H | Nondisplaced midcervical fracture of unspecified femur, subsequent encounter for open fracture type I or II with delayed healing |
| S72.036J | Nondisplaced midcervical fracture of unspecified femur, subsequent encounter for open fracture type IIIA, IIIB, or IIIC with delayed healing |
| S72.036K | Nondisplaced midcervical fracture of unspecified femur, subsequent encounter for closed fracture with nonunion |
| S72.036M | Nondisplaced midcervical fracture of unspecified femur, subsequent encounter for open fracture type I or II with nonunion |
| S72.036N | Nondisplaced midcervical fracture of unspecified femur, subsequent encounter for open fracture type IIIA, IIIB, or IIIC with nonunion |
| S72.036P | Nondisplaced midcervical fracture of unspecified femur, subsequent encounter for closed fracture with malunion |
| S72.036Q | Nondisplaced midcervical fracture of unspecified femur, subsequent encounter for open fracture type I or II with malunion |
| S72.036R | Nondisplaced midcervical fracture of unspecified femur, subsequent encounter for open fracture type IIIA, IIIB, or IIIC with malunion |
| S72.036S | Nondisplaced midcervical fracture of unspecified femur, sequela |
| S72.04 | Fracture of base of neck of femur |
| S72.041 | Displaced fracture of base of neck of right femur |
| S72.041A | Displaced fracture of base of neck of right femur, initial encounter for closed fracture |
| S72.041B | Displaced fracture of base of neck of right femur, initial encounter for open fracture type I or II |
| S72.041C | Displaced fracture of base of neck of right femur, initial encounter for open fracture type IIIA, IIIB, or IIIC |
| S72.041D | Displaced fracture of base of neck of right femur, subsequent encounter for closed fracture with routine healing |
| S72.041E | Displaced fracture of base of neck of right femur, subsequent encounter for open fracture type I or II with routine healing |
| S72.041F | Displaced fracture of base of neck of right femur, subsequent encounter for open fracture type IIIA, IIIB, or IIIC with routine healing |
| S72.041G | Displaced fracture of base of neck of right femur, subsequent encounter for closed fracture with delayed healing |
| S72.041H | Displaced fracture of base of neck of right femur, subsequent encounter for open fracture type I or II with delayed healing |
| S72.041J | Displaced fracture of base of neck of right femur, subsequent encounter for open fracture type IIIA, IIIB, or IIIC with delayed healing |
| S72.041K | Displaced fracture of base of neck of right femur, subsequent encounter for closed fracture with nonunion |
| S72.041M | Displaced fracture of base of neck of right femur, subsequent encounter for open fracture type I or II with nonunion |
| S72.041N | Displaced fracture of base of neck of right femur, subsequent encounter for open fracture type IIIA, IIIB, or IIIC with nonunion |
| S72.041P | Displaced fracture of base of neck of right femur, subsequent encounter for closed fracture with malunion |
| S72.041Q | Displaced fracture of base of neck of right femur, subsequent encounter for open fracture type I or II with malunion |
| S72.041R | Displaced fracture of base of neck of right femur, subsequent encounter for open fracture type IIIA, IIIB, or IIIC with malunion |
| S72.041S | Displaced fracture of base of neck of right femur, sequela |
| S72.042 | Displaced fracture of base of neck of left femur |
| S72.042A | Displaced fracture of base of neck of left femur, initial encounter for closed fracture |
| S72.042B | Displaced fracture of base of neck of left femur, initial encounter for open fracture type I or II |
| S72.042C | Displaced fracture of base of neck of left femur, initial encounter for open fracture type IIIA, IIIB, or IIIC |
| S72.042D | Displaced fracture of base of neck of left femur, subsequent encounter for closed fracture with routine healing |
| S72.042E | Displaced fracture of base of neck of left femur, subsequent encounter for open fracture type I or II with routine healing |
| S72.042F | Displaced fracture of base of neck of left femur, subsequent encounter for open fracture type IIIA, IIIB, or IIIC with routine healing |
| S72.042G | Displaced fracture of base of neck of left femur, subsequent encounter for closed fracture with delayed healing |
| S72.042H | Displaced fracture of base of neck of left femur, subsequent encounter for open fracture type I or II with delayed healing |
| S72.042J | Displaced fracture of base of neck of left femur, subsequent encounter for open fracture type IIIA, IIIB, or IIIC with delayed healing |
| S72.042K | Displaced fracture of base of neck of left femur, subsequent encounter for closed fracture with nonunion |
| S72.042M | Displaced fracture of base of neck of left femur, subsequent encounter for open fracture type I or II with nonunion |
| S72.042N | Displaced fracture of base of neck of left femur, subsequent encounter for open fracture type IIIA, IIIB, or IIIC with nonunion |
| S72.042P | Displaced fracture of base of neck of left femur, subsequent encounter for closed fracture with malunion |
| S72.042Q | Displaced fracture of base of neck of left femur, subsequent encounter for open fracture type I or II with malunion |
| S72.042R | Displaced fracture of base of neck of left femur, subsequent encounter for open fracture type IIIA, IIIB, or IIIC with malunion |
| S72.042S | Displaced fracture of base of neck of left femur, sequela |
| S72.043 | Displaced fracture of base of neck of unspecified femur |
| S72.043A | Displaced fracture of base of neck of unspecified femur, initial encounter for closed fracture |
| S72.043B | Displaced fracture of base of neck of unspecified femur, initial encounter for open fracture type I or II |
| S72.043C | Displaced fracture of base of neck of unspecified femur, initial encounter for open fracture type IIIA, IIIB, or IIIC |
| S72.043D | Displaced fracture of base of neck of unspecified femur, subsequent encounter for closed fracture with routine healing |
| S72.043E | Displaced fracture of base of neck of unspecified femur, subsequent encounter for open fracture type I or II with routine healing |
| S72.043F | Displaced fracture of base of neck of unspecified femur, subsequent encounter for open fracture type IIIA, IIIB, or IIIC with routine healing |
| S72.043G | Displaced fracture of base of neck of unspecified femur, subsequent encounter for closed fracture with delayed healing |
| S72.043H | Displaced fracture of base of neck of unspecified femur, subsequent encounter for open fracture type I or II with delayed healing |
| S72.043J | Displaced fracture of base of neck of unspecified femur, subsequent encounter for open fracture type IIIA, IIIB, or IIIC with delayed healing |
| S72.043K | Displaced fracture of base of neck of unspecified femur, subsequent encounter for closed fracture with nonunion |
| S72.043M | Displaced fracture of base of neck of unspecified femur, subsequent encounter for open fracture type I or II with nonunion |
| S72.043N | Displaced fracture of base of neck of unspecified femur, subsequent encounter for open fracture type IIIA, IIIB, or IIIC with nonunion |
| S72.043P | Displaced fracture of base of neck of unspecified femur, subsequent encounter for closed fracture with malunion |
| S72.043Q | Displaced fracture of base of neck of unspecified femur, subsequent encounter for open fracture type I or II with malunion |
| S72.043R | Displaced fracture of base of neck of unspecified femur, subsequent encounter for open fracture type IIIA, IIIB, or IIIC with malunion |
| S72.043S | Displaced fracture of base of neck of unspecified femur, sequela |
| S72.044 | Nondisplaced fracture of base of neck of right femur |
| S72.044A | Nondisplaced fracture of base of neck of right femur, initial encounter for closed fracture |
| S72.044B | Nondisplaced fracture of base of neck of right femur, initial encounter for open fracture type I or II |
| S72.044C | Nondisplaced fracture of base of neck of right femur, initial encounter for open fracture type IIIA, IIIB, or IIIC |
| S72.044D | Nondisplaced fracture of base of neck of right femur, subsequent encounter for closed fracture with routine healing |
| S72.044E | Nondisplaced fracture of base of neck of right femur, subsequent encounter for open fracture type I or II with routine healing |
| S72.044F | Nondisplaced fracture of base of neck of right femur, subsequent encounter for open fracture type IIIA, IIIB, or IIIC with routine healing |
| S72.044G | Nondisplaced fracture of base of neck of right femur, subsequent encounter for closed fracture with delayed healing |
| S72.044H | Nondisplaced fracture of base of neck of right femur, subsequent encounter for open fracture type I or II with delayed healing |
| S72.044J | Nondisplaced fracture of base of neck of right femur, subsequent encounter for open fracture type IIIA, IIIB, or IIIC with delayed healing |
| S72.044K | Nondisplaced fracture of base of neck of right femur, subsequent encounter for closed fracture with nonunion |
| S72.044M | Nondisplaced fracture of base of neck of right femur, subsequent encounter for open fracture type I or II with nonunion |
| S72.044N | Nondisplaced fracture of base of neck of right femur, subsequent encounter for open fracture type IIIA, IIIB, or IIIC with nonunion |
| S72.044P | Nondisplaced fracture of base of neck of right femur, subsequent encounter for closed fracture with malunion |
| S72.044Q | Nondisplaced fracture of base of neck of right femur, subsequent encounter for open fracture type I or II with malunion |
| S72.044R | Nondisplaced fracture of base of neck of right femur, subsequent encounter for open fracture type IIIA, IIIB, or IIIC with malunion |
| S72.044S | Nondisplaced fracture of base of neck of right femur, sequela |
| S72.045 | Nondisplaced fracture of base of neck of left femur |
| S72.045A | Nondisplaced fracture of base of neck of left femur, initial encounter for closed fracture |
| S72.045B | Nondisplaced fracture of base of neck of left femur, initial encounter for open fracture type I or II |
| S72.045C | Nondisplaced fracture of base of neck of left femur, initial encounter for open fracture type IIIA, IIIB, or IIIC |
| S72.045D | Nondisplaced fracture of base of neck of left femur, subsequent encounter for closed fracture with routine healing |
| S72.045E | Nondisplaced fracture of base of neck of left femur, subsequent encounter for open fracture type I or II with routine healing |
| S72.045F | Nondisplaced fracture of base of neck of left femur, subsequent encounter for open fracture type IIIA, IIIB, or IIIC with routine healing |
| S72.045G | Nondisplaced fracture of base of neck of left femur, subsequent encounter for closed fracture with delayed healing |
| S72.045H | Nondisplaced fracture of base of neck of left femur, subsequent encounter for open fracture type I or II with delayed healing |
| S72.045J | Nondisplaced fracture of base of neck of left femur, subsequent encounter for open fracture type IIIA, IIIB, or IIIC with delayed healing |
| S72.045K | Nondisplaced fracture of base of neck of left femur, subsequent encounter for closed fracture with nonunion |
| S72.045M | Nondisplaced fracture of base of neck of left femur, subsequent encounter for open fracture type I or II with nonunion |
| S72.045N | Nondisplaced fracture of base of neck of left femur, subsequent encounter for open fracture type IIIA, IIIB, or IIIC with nonunion |
| S72.045P | Nondisplaced fracture of base of neck of left femur, subsequent encounter for closed fracture with malunion |
| S72.045Q | Nondisplaced fracture of base of neck of left femur, subsequent encounter for open fracture type I or II with malunion |
| S72.045R | Nondisplaced fracture of base of neck of left femur, subsequent encounter for open fracture type IIIA, IIIB, or IIIC with malunion |
| S72.045S | Nondisplaced fracture of base of neck of left femur, sequela |
| S72.046 | Nondisplaced fracture of base of neck of unspecified femur |
| S72.046A | Nondisplaced fracture of base of neck of unspecified femur, initial encounter for closed fracture |
| S72.046B | Nondisplaced fracture of base of neck of unspecified femur, initial encounter for open fracture type I or II |
| S72.046C | Nondisplaced fracture of base of neck of unspecified femur, initial encounter for open fracture type IIIA, IIIB, or IIIC |
| S72.046D | Nondisplaced fracture of base of neck of unspecified femur, subsequent encounter for closed fracture with routine healing |
| S72.046E | Nondisplaced fracture of base of neck of unspecified femur, subsequent encounter for open fracture type I or II with routine healing |
| S72.046F | Nondisplaced fracture of base of neck of unspecified femur, subsequent encounter for open fracture type IIIA, IIIB, or IIIC with routine healing |
| S72.046G | Nondisplaced fracture of base of neck of unspecified femur, subsequent encounter for closed fracture with delayed healing |
| S72.046H | Nondisplaced fracture of base of neck of unspecified femur, subsequent encounter for open fracture type I or II with delayed healing |
| S72.046J | Nondisplaced fracture of base of neck of unspecified femur, subsequent encounter for open fracture type IIIA, IIIB, or IIIC with delayed healing |
| S72.046K | Nondisplaced fracture of base of neck of unspecified femur, subsequent encounter for closed fracture with nonunion |
| S72.046M | Nondisplaced fracture of base of neck of unspecified femur, subsequent encounter for open fracture type I or II with nonunion |
| S72.046N | Nondisplaced fracture of base of neck of unspecified femur, subsequent encounter for open fracture type IIIA, IIIB, or IIIC with nonunion |
| S72.046P | Nondisplaced fracture of base of neck of unspecified femur, subsequent encounter for closed fracture with malunion |
| S72.046Q | Nondisplaced fracture of base of neck of unspecified femur, subsequent encounter for open fracture type I or II with malunion |
| S72.046R | Nondisplaced fracture of base of neck of unspecified femur, subsequent encounter for open fracture type IIIA, IIIB, or IIIC with malunion |
| S72.046S | Nondisplaced fracture of base of neck of unspecified femur, sequela |
| S72.09 | Other fracture of head and neck of femur |
| S72.091 | Other fracture of head and neck of right femur |
| S72.091A | Other fracture of head and neck of right femur, initial encounter for closed fracture |
| S72.091B | Other fracture of head and neck of right femur, initial encounter for open fracture type I or II |
| S72.091C | Other fracture of head and neck of right femur, initial encounter for open fracture type IIIA, IIIB, or IIIC |
| S72.091D | Other fracture of head and neck of right femur, subsequent encounter for closed fracture with routine healing |
| S72.091E | Other fracture of head and neck of right femur, subsequent encounter for open fracture type I or II with routine healing |
| S72.091F | Other fracture of head and neck of right femur, subsequent encounter for open fracture type IIIA, IIIB, or IIIC with routine healing |
| S72.091G | Other fracture of head and neck of right femur, subsequent encounter for closed fracture with delayed healing |
| S72.091H | Other fracture of head and neck of right femur, subsequent encounter for open fracture type I or II with delayed healing |
| S72.091J | Other fracture of head and neck of right femur, subsequent encounter for open fracture type IIIA, IIIB, or IIIC with delayed healing |
| S72.091K | Other fracture of head and neck of right femur, subsequent encounter for closed fracture with nonunion |
| S72.091M | Other fracture of head and neck of right femur, subsequent encounter for open fracture type I or II with nonunion |
| S72.091N | Other fracture of head and neck of right femur, subsequent encounter for open fracture type IIIA, IIIB, or IIIC with nonunion |
| S72.091P | Other fracture of head and neck of right femur, subsequent encounter for closed fracture with malunion |
| S72.091Q | Other fracture of head and neck of right femur, subsequent encounter for open fracture type I or II with malunion |
| S72.091R | Other fracture of head and neck of right femur, subsequent encounter for open fracture type IIIA, IIIB, or IIIC with malunion |
| S72.091S | Other fracture of head and neck of right femur, sequela |
| S72.092 | Other fracture of head and neck of left femur |
| S72.092A | Other fracture of head and neck of left femur, initial encounter for closed fracture |
| S72.092B | Other fracture of head and neck of left femur, initial encounter for open fracture type I or II |
| S72.092C | Other fracture of head and neck of left femur, initial encounter for open fracture type IIIA, IIIB, or IIIC |
| S72.092D | Other fracture of head and neck of left femur, subsequent encounter for closed fracture with routine healing |
| S72.092E | Other fracture of head and neck of left femur, subsequent encounter for open fracture type I or II with routine healing |
| S72.092F | Other fracture of head and neck of left femur, subsequent encounter for open fracture type IIIA, IIIB, or IIIC with routine healing |
| S72.092G | Other fracture of head and neck of left femur, subsequent encounter for closed fracture with delayed healing |
| S72.092H | Other fracture of head and neck of left femur, subsequent encounter for open fracture type I or II with delayed healing |
| S72.092J | Other fracture of head and neck of left femur, subsequent encounter for open fracture type IIIA, IIIB, or IIIC with delayed healing |
| S72.092K | Other fracture of head and neck of left femur, subsequent encounter for closed fracture with nonunion |
| S72.092M | Other fracture of head and neck of left femur, subsequent encounter for open fracture type I or II with nonunion |
| S72.092N | Other fracture of head and neck of left femur, subsequent encounter for open fracture type IIIA, IIIB, or IIIC with nonunion |
| S72.092P | Other fracture of head and neck of left femur, subsequent encounter for closed fracture with malunion |
| S72.092Q | Other fracture of head and neck of left femur, subsequent encounter for open fracture type I or II with malunion |
| S72.092R | Other fracture of head and neck of left femur, subsequent encounter for open fracture type IIIA, IIIB, or IIIC with malunion |
| S72.092S | Other fracture of head and neck of left femur, sequela |
| S72.099 | Other fracture of head and neck of unspecified femur |
| S72.099A | Other fracture of head and neck of unspecified femur, initial encounter for closed fracture |
| S72.099B | Other fracture of head and neck of unspecified femur, initial encounter for open fracture type I or II |
| S72.099C | Other fracture of head and neck of unspecified femur, initial encounter for open fracture type IIIA, IIIB, or IIIC |
| S72.099D | Other fracture of head and neck of unspecified femur, subsequent encounter for closed fracture with routine healing |
| S72.099E | Other fracture of head and neck of unspecified femur, subsequent encounter for open fracture type I or II with routine healing |
| S72.099F | Other fracture of head and neck of unspecified femur, subsequent encounter for open fracture type IIIA, IIIB, or IIIC with routine healing |
| S72.099G | Other fracture of head and neck of unspecified femur, subsequent encounter for closed fracture with delayed healing |
| S72.099H | Other fracture of head and neck of unspecified femur, subsequent encounter for open fracture type I or II with delayed healing |
| S72.099J | Other fracture of head and neck of unspecified femur, subsequent encounter for open fracture type IIIA, IIIB, or IIIC with delayed healing |
| S72.099K | Other fracture of head and neck of unspecified femur, subsequent encounter for closed fracture with nonunion |
| S72.099M | Other fracture of head and neck of unspecified femur, subsequent encounter for open fracture type I or II with nonunion |
| S72.099N | Other fracture of head and neck of unspecified femur, subsequent encounter for open fracture type IIIA, IIIB, or IIIC with nonunion |
| S72.099P | Other fracture of head and neck of unspecified femur, subsequent encounter for closed fracture with malunion |
| S72.099Q | Other fracture of head and neck of unspecified femur, subsequent encounter for open fracture type I or II with malunion |
| S72.099R | Other fracture of head and neck of unspecified femur, subsequent encounter for open fracture type IIIA, IIIB, or IIIC with malunion |
| S72.099S | Other fracture of head and neck of unspecified femur, sequela |
| S82.11 | Fracture of tibial spine |
| S82.111 | Displaced fracture of right tibial spine |
| S82.111A | Displaced fracture of right tibial spine, initial encounter for closed fracture |
| S82.111B | Displaced fracture of right tibial spine, initial encounter for open fracture type I or II |
| S82.111C | Displaced fracture of right tibial spine, initial encounter for open fracture type IIIA, IIIB, or IIIC |
| S82.111D | Displaced fracture of right tibial spine, subsequent encounter for closed fracture with routine healing |
| S82.111E | Displaced fracture of right tibial spine, subsequent encounter for open fracture type I or II with routine healing |
| S82.111F | Displaced fracture of right tibial spine, subsequent encounter for open fracture type IIIA, IIIB, or IIIC with routine healing |
| S82.111G | Displaced fracture of right tibial spine, subsequent encounter for closed fracture with delayed healing |
| S82.111H | Displaced fracture of right tibial spine, subsequent encounter for open fracture type I or II with delayed healing |
| S82.111J | Displaced fracture of right tibial spine, subsequent encounter for open fracture type IIIA, IIIB, or IIIC with delayed healing |
| S82.111K | Displaced fracture of right tibial spine, subsequent encounter for closed fracture with nonunion |
| S82.111M | Displaced fracture of right tibial spine, subsequent encounter for open fracture type I or II with nonunion |
| S82.111N | Displaced fracture of right tibial spine, subsequent encounter for open fracture type IIIA, IIIB, or IIIC with nonunion |
| S82.111P | Displaced fracture of right tibial spine, subsequent encounter for closed fracture with malunion |
| S82.111Q | Displaced fracture of right tibial spine, subsequent encounter for open fracture type I or II with malunion |
| S82.111R | Displaced fracture of right tibial spine, subsequent encounter for open fracture type IIIA, IIIB, or IIIC with malunion |
| S82.111S | Displaced fracture of right tibial spine, sequela |
| S82.112 | Displaced fracture of left tibial spine |
| S82.112A | Displaced fracture of left tibial spine, initial encounter for closed fracture |
| S82.112B | Displaced fracture of left tibial spine, initial encounter for open fracture type I or II |
| S82.112C | Displaced fracture of left tibial spine, initial encounter for open fracture type IIIA, IIIB, or IIIC |
| S82.112D | Displaced fracture of left tibial spine, subsequent encounter for closed fracture with routine healing |
| S82.112E | Displaced fracture of left tibial spine, subsequent encounter for open fracture type I or II with routine healing |
| S82.112F | Displaced fracture of left tibial spine, subsequent encounter for open fracture type IIIA, IIIB, or IIIC with routine healing |
| S82.112G | Displaced fracture of left tibial spine, subsequent encounter for closed fracture with delayed healing |
| S82.112H | Displaced fracture of left tibial spine, subsequent encounter for open fracture type I or II with delayed healing |
| S82.112J | Displaced fracture of left tibial spine, subsequent encounter for open fracture type IIIA, IIIB, or IIIC with delayed healing |
| S82.112K | Displaced fracture of left tibial spine, subsequent encounter for closed fracture with nonunion |
| S82.112M | Displaced fracture of left tibial spine, subsequent encounter for open fracture type I or II with nonunion |
| S82.112N | Displaced fracture of left tibial spine, subsequent encounter for open fracture type IIIA, IIIB, or IIIC with nonunion |
| S82.112P | Displaced fracture of left tibial spine, subsequent encounter for closed fracture with malunion |
| S82.112Q | Displaced fracture of left tibial spine, subsequent encounter for open fracture type I or II with malunion |
| S82.112R | Displaced fracture of left tibial spine, subsequent encounter for open fracture type IIIA, IIIB, or IIIC with malunion |
| S82.112S | Displaced fracture of left tibial spine, sequela |
| S82.113 | Displaced fracture of unspecified tibial spine |
| S82.113A | Displaced fracture of unspecified tibial spine, initial encounter for closed fracture |
| S82.113B | Displaced fracture of unspecified tibial spine, initial encounter for open fracture type I or II |
| S82.113C | Displaced fracture of unspecified tibial spine, initial encounter for open fracture type IIIA, IIIB, or IIIC |
| S82.113D | Displaced fracture of unspecified tibial spine, subsequent encounter for closed fracture with routine healing |
| S82.113E | Displaced fracture of unspecified tibial spine, subsequent encounter for open fracture type I or II with routine healing |
| S82.113F | Displaced fracture of unspecified tibial spine, subsequent encounter for open fracture type IIIA, IIIB, or IIIC with routine healing |
| S82.113G | Displaced fracture of unspecified tibial spine, subsequent encounter for closed fracture with delayed healing |
| S82.113H | Displaced fracture of unspecified tibial spine, subsequent encounter for open fracture type I or II with delayed healing |
| S82.113J | Displaced fracture of unspecified tibial spine, subsequent encounter for open fracture type IIIA, IIIB, or IIIC with delayed healing |
| S82.113K | Displaced fracture of unspecified tibial spine, subsequent encounter for closed fracture with nonunion |
| S82.113M | Displaced fracture of unspecified tibial spine, subsequent encounter for open fracture type I or II with nonunion |
| S82.113N | Displaced fracture of unspecified tibial spine, subsequent encounter for open fracture type IIIA, IIIB, or IIIC with nonunion |
| S82.113P | Displaced fracture of unspecified tibial spine, subsequent encounter for closed fracture with malunion |
| S82.113Q | Displaced fracture of unspecified tibial spine, subsequent encounter for open fracture type I or II with malunion |
| S82.113R | Displaced fracture of unspecified tibial spine, subsequent encounter for open fracture type IIIA, IIIB, or IIIC with malunion |
| S82.113S | Displaced fracture of unspecified tibial spine, sequela |
| S82.114 | Nondisplaced fracture of right tibial spine |
| S82.114A | Nondisplaced fracture of right tibial spine, initial encounter for closed fracture |
| S82.114B | Nondisplaced fracture of right tibial spine, initial encounter for open fracture type I or II |
| S82.114C | Nondisplaced fracture of right tibial spine, initial encounter for open fracture type IIIA, IIIB, or IIIC |
| S82.114D | Nondisplaced fracture of right tibial spine, subsequent encounter for closed fracture with routine healing |
| S82.114E | Nondisplaced fracture of right tibial spine, subsequent encounter for open fracture type I or II with routine healing |
| S82.114F | Nondisplaced fracture of right tibial spine, subsequent encounter for open fracture type IIIA, IIIB, or IIIC with routine healing |
| S82.114G | Nondisplaced fracture of right tibial spine, subsequent encounter for closed fracture with delayed healing |
| S82.114H | Nondisplaced fracture of right tibial spine, subsequent encounter for open fracture type I or II with delayed healing |
| S82.114J | Nondisplaced fracture of right tibial spine, subsequent encounter for open fracture type IIIA, IIIB, or IIIC with delayed healing |
| S82.114K | Nondisplaced fracture of right tibial spine, subsequent encounter for closed fracture with nonunion |
| S82.114M | Nondisplaced fracture of right tibial spine, subsequent encounter for open fracture type I or II with nonunion |
| S82.114N | Nondisplaced fracture of right tibial spine, subsequent encounter for open fracture type IIIA, IIIB, or IIIC with nonunion |
| S82.114P | Nondisplaced fracture of right tibial spine, subsequent encounter for closed fracture with malunion |
| S82.114Q | Nondisplaced fracture of right tibial spine, subsequent encounter for open fracture type I or II with malunion |
| S82.114R | Nondisplaced fracture of right tibial spine, subsequent encounter for open fracture type IIIA, IIIB, or IIIC with malunion |
| S82.114S | Nondisplaced fracture of right tibial spine, sequela |
| S82.115 | Nondisplaced fracture of left tibial spine |
| S82.115A | Nondisplaced fracture of left tibial spine, initial encounter for closed fracture |
| S82.115B | Nondisplaced fracture of left tibial spine, initial encounter for open fracture type I or II |
| S82.115C | Nondisplaced fracture of left tibial spine, initial encounter for open fracture type IIIA, IIIB, or IIIC |
| S82.115D | Nondisplaced fracture of left tibial spine, subsequent encounter for closed fracture with routine healing |
| S82.115E | Nondisplaced fracture of left tibial spine, subsequent encounter for open fracture type I or II with routine healing |
| S82.115F | Nondisplaced fracture of left tibial spine, subsequent encounter for open fracture type IIIA, IIIB, or IIIC with routine healing |
| S82.115G | Nondisplaced fracture of left tibial spine, subsequent encounter for closed fracture with delayed healing |
| S82.115H | Nondisplaced fracture of left tibial spine, subsequent encounter for open fracture type I or II with delayed healing |
| S82.115J | Nondisplaced fracture of left tibial spine, subsequent encounter for open fracture type IIIA, IIIB, or IIIC with delayed healing |
| S82.115K | Nondisplaced fracture of left tibial spine, subsequent encounter for closed fracture with nonunion |
| S82.115M | Nondisplaced fracture of left tibial spine, subsequent encounter for open fracture type I or II with nonunion |
| S82.115N | Nondisplaced fracture of left tibial spine, subsequent encounter for open fracture type IIIA, IIIB, or IIIC with nonunion |
| S82.115P | Nondisplaced fracture of left tibial spine, subsequent encounter for closed fracture with malunion |
| S82.115Q | Nondisplaced fracture of left tibial spine, subsequent encounter for open fracture type I or II with malunion |
| S82.115R | Nondisplaced fracture of left tibial spine, subsequent encounter for open fracture type IIIA, IIIB, or IIIC with malunion |
| S82.115S | Nondisplaced fracture of left tibial spine, sequela |
| S82.116 | Nondisplaced fracture of unspecified tibial spine |
| S82.116A | Nondisplaced fracture of unspecified tibial spine, initial encounter for closed fracture |
| S82.116B | Nondisplaced fracture of unspecified tibial spine, initial encounter for open fracture type I or II |
| S82.116C | Nondisplaced fracture of unspecified tibial spine, initial encounter for open fracture type IIIA, IIIB, or IIIC |
| S82.116D | Nondisplaced fracture of unspecified tibial spine, subsequent encounter for closed fracture with routine healing |
| S82.116E | Nondisplaced fracture of unspecified tibial spine, subsequent encounter for open fracture type I or II with routine healing |
| S82.116F | Nondisplaced fracture of unspecified tibial spine, subsequent encounter for open fracture type IIIA, IIIB, or IIIC with routine healing |
| S82.116G | Nondisplaced fracture of unspecified tibial spine, subsequent encounter for closed fracture with delayed healing |
| S82.116H | Nondisplaced fracture of unspecified tibial spine, subsequent encounter for open fracture type I or II with delayed healing |
| S82.116J | Nondisplaced fracture of unspecified tibial spine, subsequent encounter for open fracture type IIIA, IIIB, or IIIC with delayed healing |
| S82.116K | Nondisplaced fracture of unspecified tibial spine, subsequent encounter for closed fracture with nonunion |
| S82.116M | Nondisplaced fracture of unspecified tibial spine, subsequent encounter for open fracture type I or II with nonunion |
| S82.116N | Nondisplaced fracture of unspecified tibial spine, subsequent encounter for open fracture type IIIA, IIIB, or IIIC with nonunion |
| S82.116P | Nondisplaced fracture of unspecified tibial spine, subsequent encounter for closed fracture with malunion |
| S82.116Q | Nondisplaced fracture of unspecified tibial spine, subsequent encounter for open fracture type I or II with malunion |
| S82.116R | Nondisplaced fracture of unspecified tibial spine, subsequent encounter for open fracture type IIIA, IIIB, or IIIC with malunion |
| S82.116S | Nondisplaced fracture of unspecified tibial spine, sequela |
| S92.11 | Fracture of neck of talus |
| S92.111 | Displaced fracture of neck of right talus |
| S92.111A | Displaced fracture of neck of right talus, initial encounter for closed fracture |
| S92.111B | Displaced fracture of neck of right talus, initial encounter for open fracture |
| S92.111D | Displaced fracture of neck of right talus, subsequent encounter for fracture with routine healing |
| S92.111G | Displaced fracture of neck of right talus, subsequent encounter for fracture with delayed healing |
| S92.111K | Displaced fracture of neck of right talus, subsequent encounter for fracture with nonunion |
| S92.111P | Displaced fracture of neck of right talus, subsequent encounter for fracture with malunion |
| S92.111S | Displaced fracture of neck of right talus, sequela |
| S92.112 | Displaced fracture of neck of left talus |
| S92.112A | Displaced fracture of neck of left talus, initial encounter for closed fracture |
| S92.112B | Displaced fracture of neck of left talus, initial encounter for open fracture |
| S92.112D | Displaced fracture of neck of left talus, subsequent encounter for fracture with routine healing |
| S92.112G | Displaced fracture of neck of left talus, subsequent encounter for fracture with delayed healing |
| S92.112K | Displaced fracture of neck of left talus, subsequent encounter for fracture with nonunion |
| S92.112P | Displaced fracture of neck of left talus, subsequent encounter for fracture with malunion |
| S92.112S | Displaced fracture of neck of left talus, sequela |
| S92.113 | Displaced fracture of neck of unspecified talus |
| S92.113A | Displaced fracture of neck of unspecified talus, initial encounter for closed fracture |
| S92.113B | Displaced fracture of neck of unspecified talus, initial encounter for open fracture |
| S92.113D | Displaced fracture of neck of unspecified talus, subsequent encounter for fracture with routine healing |
| S92.113G | Displaced fracture of neck of unspecified talus, subsequent encounter for fracture with delayed healing |
| S92.113K | Displaced fracture of neck of unspecified talus, subsequent encounter for fracture with nonunion |
| S92.113P | Displaced fracture of neck of unspecified talus, subsequent encounter for fracture with malunion |
| S92.113S | Displaced fracture of neck of unspecified talus, sequela |
| S92.114 | Nondisplaced fracture of neck of right talus |
| S92.114A | Nondisplaced fracture of neck of right talus, initial encounter for closed fracture |
| S92.114B | Nondisplaced fracture of neck of right talus, initial encounter for open fracture |
| S92.114D | Nondisplaced fracture of neck of right talus, subsequent encounter for fracture with routine healing |
| S92.114G | Nondisplaced fracture of neck of right talus, subsequent encounter for fracture with delayed healing |
| S92.114K | Nondisplaced fracture of neck of right talus, subsequent encounter for fracture with nonunion |
| S92.114P | Nondisplaced fracture of neck of right talus, subsequent encounter for fracture with malunion |
| S92.114S | Nondisplaced fracture of neck of right talus, sequela |
| S92.115 | Nondisplaced fracture of neck of left talus |
| S92.115A | Nondisplaced fracture of neck of left talus, initial encounter for closed fracture |
| S92.115B | Nondisplaced fracture of neck of left talus, initial encounter for open fracture |
| S92.115D | Nondisplaced fracture of neck of left talus, subsequent encounter for fracture with routine healing |
| S92.115G | Nondisplaced fracture of neck of left talus, subsequent encounter for fracture with delayed healing |
| S92.115K | Nondisplaced fracture of neck of left talus, subsequent encounter for fracture with nonunion |
| S92.115P | Nondisplaced fracture of neck of left talus, subsequent encounter for fracture with malunion |
| S92.115S | Nondisplaced fracture of neck of left talus, sequela |
| S92.116 | Nondisplaced fracture of neck of unspecified talus |
| S92.116A | Nondisplaced fracture of neck of unspecified talus, initial encounter for closed fracture |
| S92.116B | Nondisplaced fracture of neck of unspecified talus, initial encounter for open fracture |
| S92.116D | Nondisplaced fracture of neck of unspecified talus, subsequent encounter for fracture with routine healing |
| S92.116G | Nondisplaced fracture of neck of unspecified talus, subsequent encounter for fracture with delayed healing |
| S92.116K | Nondisplaced fracture of neck of unspecified talus, subsequent encounter for fracture with nonunion |
| S92.116P | Nondisplaced fracture of neck of unspecified talus, subsequent encounter for fracture with malunion |
| S92.116S | Nondisplaced fracture of neck of unspecified talus, sequela |
| T21 | Burn and corrosion of trunk |
| T21.0 | Burn of unspecified degree of trunk |
| T21.00 | Burn of unspecified degree of trunk, unspecified site |
| T21.00XA | Burn of unspecified degree of trunk, unspecified site, initial encounter |
| T21.00XD | Burn of unspecified degree of trunk, unspecified site, subsequent encounter |
| T21.00XS | Burn of unspecified degree of trunk, unspecified site, sequela |
| T21.01 | Burn of unspecified degree of chest wall |
| T21.01XA | Burn of unspecified degree of chest wall, initial encounter |
| T21.01XD | Burn of unspecified degree of chest wall, subsequent encounter |
| T21.01XS | Burn of unspecified degree of chest wall, sequela |
| T21.02 | Burn of unspecified degree of abdominal wall |
| T21.02XA | Burn of unspecified degree of abdominal wall, initial encounter |
| T21.02XD | Burn of unspecified degree of abdominal wall, subsequent encounter |
| T21.02XS | Burn of unspecified degree of abdominal wall, sequela |
| T21.03 | Burn of unspecified degree of upper back |
| T21.03XA | Burn of unspecified degree of upper back, initial encounter |
| T21.03XD | Burn of unspecified degree of upper back, subsequent encounter |
| T21.03XS | Burn of unspecified degree of upper back, sequela |
| T21.04 | Burn of unspecified degree of lower back |
| T21.04XA | Burn of unspecified degree of lower back, initial encounter |
| T21.04XD | Burn of unspecified degree of lower back, subsequent encounter |
| T21.04XS | Burn of unspecified degree of lower back, sequela |
| T21.05 | Burn of unspecified degree of buttock |
| T21.05XA | Burn of unspecified degree of buttock, initial encounter |
| T21.05XD | Burn of unspecified degree of buttock, subsequent encounter |
| T21.05XS | Burn of unspecified degree of buttock, sequela |
| T21.06 | Burn of unspecified degree of male genital region |
| T21.06XA | Burn of unspecified degree of male genital region, initial encounter |
| T21.06XD | Burn of unspecified degree of male genital region, subsequent encounter |
| T21.06XS | Burn of unspecified degree of male genital region, sequela |
| T21.07 | Burn of unspecified degree of female genital region |
| T21.07XA | Burn of unspecified degree of female genital region, initial encounter |
| T21.07XD | Burn of unspecified degree of female genital region, subsequent encounter |
| T21.07XS | Burn of unspecified degree of female genital region, sequela |
| T21.09 | Burn of unspecified degree of other site of trunk |
| T21.09XA | Burn of unspecified degree of other site of trunk, initial encounter |
| T21.09XD | Burn of unspecified degree of other site of trunk, subsequent encounter |
| T21.09XS | Burn of unspecified degree of other site of trunk, sequela |
| T21.1 | Burn of first degree of trunk |
| T21.10 | Burn of first degree of trunk, unspecified site |
| T21.10XA | Burn of first degree of trunk, unspecified site, initial encounter |
| T21.10XD | Burn of first degree of trunk, unspecified site, subsequent encounter |
| T21.10XS | Burn of first degree of trunk, unspecified site, sequela |
| T21.11 | Burn of first degree of chest wall |
| T21.11XA | Burn of first degree of chest wall, initial encounter |
| T21.11XD | Burn of first degree of chest wall, subsequent encounter |
| T21.11XS | Burn of first degree of chest wall, sequela |
| T21.12 | Burn of first degree of abdominal wall |
| T21.12XA | Burn of first degree of abdominal wall, initial encounter |
| T21.12XD | Burn of first degree of abdominal wall, subsequent encounter |
| T21.12XS | Burn of first degree of abdominal wall, sequela |
| T21.13 | Burn of first degree of upper back |
| T21.13XA | Burn of first degree of upper back, initial encounter |
| T21.13XD | Burn of first degree of upper back, subsequent encounter |
| T21.13XS | Burn of first degree of upper back, sequela |
| T21.14 | Burn of first degree of lower back |
| T21.14XA | Burn of first degree of lower back, initial encounter |
| T21.14XD | Burn of first degree of lower back, subsequent encounter |
| T21.14XS | Burn of first degree of lower back, sequela |
| T21.15 | Burn of first degree of buttock |
| T21.15XA | Burn of first degree of buttock, initial encounter |
| T21.15XD | Burn of first degree of buttock, subsequent encounter |
| T21.15XS | Burn of first degree of buttock, sequela |
| T21.16 | Burn of first degree of male genital region |
| T21.16XA | Burn of first degree of male genital region, initial encounter |
| T21.16XD | Burn of first degree of male genital region, subsequent encounter |
| T21.16XS | Burn of first degree of male genital region, sequela |
| T21.17 | Burn of first degree of female genital region |
| T21.17XA | Burn of first degree of female genital region, initial encounter |
| T21.17XD | Burn of first degree of female genital region, subsequent encounter |
| T21.17XS | Burn of first degree of female genital region, sequela |
| T21.19 | Burn of first degree of other site of trunk |
| T21.19XA | Burn of first degree of other site of trunk, initial encounter |
| T21.19XD | Burn of first degree of other site of trunk, subsequent encounter |
| T21.19XS | Burn of first degree of other site of trunk, sequela |
| T21.2 | Burn of second degree of trunk |
| T21.20 | Burn of second degree of trunk, unspecified site |
| T21.20XA | Burn of second degree of trunk, unspecified site, initial encounter |
| T21.20XD | Burn of second degree of trunk, unspecified site, subsequent encounter |
| T21.20XS | Burn of second degree of trunk, unspecified site, sequela |
| T21.21 | Burn of second degree of chest wall |
| T21.21XA | Burn of second degree of chest wall, initial encounter |
| T21.21XD | Burn of second degree of chest wall, subsequent encounter |
| T21.21XS | Burn of second degree of chest wall, sequela |
| T21.22 | Burn of second degree of abdominal wall |
| T21.22XA | Burn of second degree of abdominal wall, initial encounter |
| T21.22XD | Burn of second degree of abdominal wall, subsequent encounter |
| T21.22XS | Burn of second degree of abdominal wall, sequela |
| T21.23 | Burn of second degree of upper back |
| T21.23XA | Burn of second degree of upper back, initial encounter |
| T21.23XD | Burn of second degree of upper back, subsequent encounter |
| T21.23XS | Burn of second degree of upper back, sequela |
| T21.24 | Burn of second degree of lower back |
| T21.24XA | Burn of second degree of lower back, initial encounter |
| T21.24XD | Burn of second degree of lower back, subsequent encounter |
| T21.24XS | Burn of second degree of lower back, sequela |
| T21.25 | Burn of second degree of buttock |
| T21.25XA | Burn of second degree of buttock, initial encounter |
| T21.25XD | Burn of second degree of buttock, subsequent encounter |
| T21.25XS | Burn of second degree of buttock, sequela |
| T21.26 | Burn of second degree of male genital region |
| T21.26XA | Burn of second degree of male genital region, initial encounter |
| T21.26XD | Burn of second degree of male genital region, subsequent encounter |
| T21.26XS | Burn of second degree of male genital region, sequela |
| T21.27 | Burn of second degree of female genital region |
| T21.27XA | Burn of second degree of female genital region, initial encounter |
| T21.27XD | Burn of second degree of female genital region, subsequent encounter |
| T21.27XS | Burn of second degree of female genital region, sequela |
| T21.29 | Burn of second degree of other site of trunk |
| T21.29XA | Burn of second degree of other site of trunk, initial encounter |
| T21.29XD | Burn of second degree of other site of trunk, subsequent encounter |
| T21.29XS | Burn of second degree of other site of trunk, sequela |
| T21.3 | Burn of third degree of trunk |
| T21.30 | Burn of third degree of trunk, unspecified site |
| T21.30XA | Burn of third degree of trunk, unspecified site, initial encounter |
| T21.30XD | Burn of third degree of trunk, unspecified site, subsequent encounter |
| T21.30XS | Burn of third degree of trunk, unspecified site, sequela |
| T21.31 | Burn of third degree of chest wall |
| T21.31XA | Burn of third degree of chest wall, initial encounter |
| T21.31XD | Burn of third degree of chest wall, subsequent encounter |
| T21.31XS | Burn of third degree of chest wall, sequela |
| T21.32 | Burn of third degree of abdominal wall |
| T21.32XA | Burn of third degree of abdominal wall, initial encounter |
| T21.32XD | Burn of third degree of abdominal wall, subsequent encounter |
| T21.32XS | Burn of third degree of abdominal wall, sequela |
| T21.33 | Burn of third degree of upper back |
| T21.33XA | Burn of third degree of upper back, initial encounter |
| T21.33XD | Burn of third degree of upper back, subsequent encounter |
| T21.33XS | Burn of third degree of upper back, sequela |
| T21.34 | Burn of third degree of lower back |
| T21.34XA | Burn of third degree of lower back, initial encounter |
| T21.34XD | Burn of third degree of lower back, subsequent encounter |
| T21.34XS | Burn of third degree of lower back, sequela |
| T21.35 | Burn of third degree of buttock |
| T21.35XA | Burn of third degree of buttock, initial encounter |
| T21.35XD | Burn of third degree of buttock, subsequent encounter |
| T21.35XS | Burn of third degree of buttock, sequela |
| T21.36 | Burn of third degree of male genital region |
| T21.36XA | Burn of third degree of male genital region, initial encounter |
| T21.36XD | Burn of third degree of male genital region, subsequent encounter |
| T21.36XS | Burn of third degree of male genital region, sequela |
| T21.37 | Burn of third degree of female genital region |
| T21.37XA | Burn of third degree of female genital region, initial encounter |
| T21.37XD | Burn of third degree of female genital region, subsequent encounter |
| T21.37XS | Burn of third degree of female genital region, sequela |
| T21.39 | Burn of third degree of other site of trunk |
| T21.39XA | Burn of third degree of other site of trunk, initial encounter |
| T21.39XD | Burn of third degree of other site of trunk, subsequent encounter |
| T21.39XS | Burn of third degree of other site of trunk, sequela |
| T21.4 | Corrosion of unspecified degree of trunk |
| T21.40 | Corrosion of unspecified degree of trunk, unspecified site |
| T21.40XA | Corrosion of unspecified degree of trunk, unspecified site, initial encounter |
| T21.40XD | Corrosion of unspecified degree of trunk, unspecified site, subsequent encounter |
| T21.40XS | Corrosion of unspecified degree of trunk, unspecified site, sequela |
| T21.41 | Corrosion of unspecified degree of chest wall |
| T21.41XA | Corrosion of unspecified degree of chest wall, initial encounter |
| T21.41XD | Corrosion of unspecified degree of chest wall, subsequent encounter |
| T21.41XS | Corrosion of unspecified degree of chest wall, sequela |
| T21.42 | Corrosion of unspecified degree of abdominal wall |
| T21.42XA | Corrosion of unspecified degree of abdominal wall, initial encounter |
| T21.42XD | Corrosion of unspecified degree of abdominal wall, subsequent encounter |
| T21.42XS | Corrosion of unspecified degree of abdominal wall, sequela |
| T21.43 | Corrosion of unspecified degree of upper back |
| T21.43XA | Corrosion of unspecified degree of upper back, initial encounter |
| T21.43XD | Corrosion of unspecified degree of upper back, subsequent encounter |
| T21.43XS | Corrosion of unspecified degree of upper back, sequela |
| T21.44 | Corrosion of unspecified degree of lower back |
| T21.44XA | Corrosion of unspecified degree of lower back, initial encounter |
| T21.44XD | Corrosion of unspecified degree of lower back, subsequent encounter |
| T21.44XS | Corrosion of unspecified degree of lower back, sequela |
| T21.45 | Corrosion of unspecified degree of buttock |
| T21.45XA | Corrosion of unspecified degree of buttock, initial encounter |
| T21.45XD | Corrosion of unspecified degree of buttock, subsequent encounter |
| T21.45XS | Corrosion of unspecified degree of buttock, sequela |
| T21.46 | Corrosion of unspecified degree of male genital region |
| T21.46XA | Corrosion of unspecified degree of male genital region, initial encounter |
| T21.46XD | Corrosion of unspecified degree of male genital region, subsequent encounter |
| T21.46XS | Corrosion of unspecified degree of male genital region, sequela |
| T21.47 | Corrosion of unspecified degree of female genital region |
| T21.47XA | Corrosion of unspecified degree of female genital region, initial encounter |
| T21.47XD | Corrosion of unspecified degree of female genital region, subsequent encounter |
| T21.47XS | Corrosion of unspecified degree of female genital region, sequela |
| T21.49 | Corrosion of unspecified degree of other site of trunk |
| T21.49XA | Corrosion of unspecified degree of other site of trunk, initial encounter |
| T21.49XD | Corrosion of unspecified degree of other site of trunk, subsequent encounter |
| T21.49XS | Corrosion of unspecified degree of other site of trunk, sequela |
| T21.5 | Corrosion of first degree of trunk |
| T21.50 | Corrosion of first degree of trunk, unspecified site |
| T21.50XA | Corrosion of first degree of trunk, unspecified site, initial encounter |
| T21.50XD | Corrosion of first degree of trunk, unspecified site, subsequent encounter |
| T21.50XS | Corrosion of first degree of trunk, unspecified site, sequela |
| T21.51 | Corrosion of first degree of chest wall |
| T21.51XA | Corrosion of first degree of chest wall, initial encounter |
| T21.51XD | Corrosion of first degree of chest wall, subsequent encounter |
| T21.51XS | Corrosion of first degree of chest wall, sequela |
| T21.52 | Corrosion of first degree of abdominal wall |
| T21.52XA | Corrosion of first degree of abdominal wall, initial encounter |
| T21.52XD | Corrosion of first degree of abdominal wall, subsequent encounter |
| T21.52XS | Corrosion of first degree of abdominal wall, sequela |
| T21.53 | Corrosion of first degree of upper back |
| T21.53XA | Corrosion of first degree of upper back, initial encounter |
| T21.53XD | Corrosion of first degree of upper back, subsequent encounter |
| T21.53XS | Corrosion of first degree of upper back, sequela |
| T21.54 | Corrosion of first degree of lower back |
| T21.54XA | Corrosion of first degree of lower back, initial encounter |
| T21.54XD | Corrosion of first degree of lower back, subsequent encounter |
| T21.54XS | Corrosion of first degree of lower back, sequela |
| T21.55 | Corrosion of first degree of buttock |
| T21.55XA | Corrosion of first degree of buttock, initial encounter |
| T21.55XD | Corrosion of first degree of buttock, subsequent encounter |
| T21.55XS | Corrosion of first degree of buttock, sequela |
| T21.56 | Corrosion of first degree of male genital region |
| T21.56XA | Corrosion of first degree of male genital region, initial encounter |
| T21.56XD | Corrosion of first degree of male genital region, subsequent encounter |
| T21.56XS | Corrosion of first degree of male genital region, sequela |
| T21.57 | Corrosion of first degree of female genital region |
| T21.57XA | Corrosion of first degree of female genital region, initial encounter |
| T21.57XD | Corrosion of first degree of female genital region, subsequent encounter |
| T21.57XS | Corrosion of first degree of female genital region, sequela |
| T21.59 | Corrosion of first degree of other site of trunk |
| T21.59XA | Corrosion of first degree of other site of trunk, initial encounter |
| T21.59XD | Corrosion of first degree of other site of trunk, subsequent encounter |
| T21.59XS | Corrosion of first degree of other site of trunk, sequela |
| T21.6 | Corrosion of second degree of trunk |
| T21.60 | Corrosion of second degree of trunk, unspecified site |
| T21.60XA | Corrosion of second degree of trunk, unspecified site, initial encounter |
| T21.60XD | Corrosion of second degree of trunk, unspecified site, subsequent encounter |
| T21.60XS | Corrosion of second degree of trunk, unspecified site, sequela |
| T21.61 | Corrosion of second degree of chest wall |
| T21.61XA | Corrosion of second degree of chest wall, initial encounter |
| T21.61XD | Corrosion of second degree of chest wall, subsequent encounter |
| T21.61XS | Corrosion of second degree of chest wall, sequela |
| T21.62 | Corrosion of second degree of abdominal wall |
| T21.62XA | Corrosion of second degree of abdominal wall, initial encounter |
| T21.62XD | Corrosion of second degree of abdominal wall, subsequent encounter |
| T21.62XS | Corrosion of second degree of abdominal wall, sequela |
| T21.63 | Corrosion of second degree of upper back |
| T21.63XA | Corrosion of second degree of upper back, initial encounter |
| T21.63XD | Corrosion of second degree of upper back, subsequent encounter |
| T21.63XS | Corrosion of second degree of upper back, sequela |
| T21.64 | Corrosion of second degree of lower back |
| T21.64XA | Corrosion of second degree of lower back, initial encounter |
| T21.64XD | Corrosion of second degree of lower back, subsequent encounter |
| T21.64XS | Corrosion of second degree of lower back, sequela |
| T21.65 | Corrosion of second degree of buttock |
| T21.65XA | Corrosion of second degree of buttock, initial encounter |
| T21.65XD | Corrosion of second degree of buttock, subsequent encounter |
| T21.65XS | Corrosion of second degree of buttock, sequela |
| T21.66 | Corrosion of second degree of male genital region |
| T21.66XA | Corrosion of second degree of male genital region, initial encounter |
| T21.66XD | Corrosion of second degree of male genital region, subsequent encounter |
| T21.66XS | Corrosion of second degree of male genital region, sequela |
| T21.67 | Corrosion of second degree of female genital region |
| T21.67XA | Corrosion of second degree of female genital region, initial encounter |
| T21.67XD | Corrosion of second degree of female genital region, subsequent encounter |
| T21.67XS | Corrosion of second degree of female genital region, sequela |
| T21.69 | Corrosion of second degree of other site of trunk |
| T21.69XA | Corrosion of second degree of other site of trunk, initial encounter |
| T21.69XD | Corrosion of second degree of other site of trunk, subsequent encounter |
| T21.69XS | Corrosion of second degree of other site of trunk, sequela |
| T21.7 | Corrosion of third degree of trunk |
| T21.70 | Corrosion of third degree of trunk, unspecified site |
| T21.70XA | Corrosion of third degree of trunk, unspecified site, initial encounter |
| T21.70XD | Corrosion of third degree of trunk, unspecified site, subsequent encounter |
| T21.70XS | Corrosion of third degree of trunk, unspecified site, sequela |
| T21.71 | Corrosion of third degree of chest wall |
| T21.71XA | Corrosion of third degree of chest wall, initial encounter |
| T21.71XD | Corrosion of third degree of chest wall, subsequent encounter |
| T21.71XS | Corrosion of third degree of chest wall, sequela |
| T21.72 | Corrosion of third degree of abdominal wall |
| T21.72XA | Corrosion of third degree of abdominal wall, initial encounter |
| T21.72XD | Corrosion of third degree of abdominal wall, subsequent encounter |
| T21.72XS | Corrosion of third degree of abdominal wall, sequela |
| T21.73 | Corrosion of third degree of upper back |
| T21.73XA | Corrosion of third degree of upper back, initial encounter |
| T21.73XD | Corrosion of third degree of upper back, subsequent encounter |
| T21.73XS | Corrosion of third degree of upper back, sequela |
| T21.74 | Corrosion of third degree of lower back |
| T21.74XA | Corrosion of third degree of lower back, initial encounter |
| T21.74XD | Corrosion of third degree of lower back, subsequent encounter |
| T21.74XS | Corrosion of third degree of lower back, sequela |
| T21.75 | Corrosion of third degree of buttock |
| T21.75XA | Corrosion of third degree of buttock, initial encounter |
| T21.75XD | Corrosion of third degree of buttock, subsequent encounter |
| T21.75XS | Corrosion of third degree of buttock, sequela |
| T21.76 | Corrosion of third degree of male genital region |
| T21.76XA | Corrosion of third degree of male genital region, initial encounter |
| T21.76XD | Corrosion of third degree of male genital region, subsequent encounter |
| T21.76XS | Corrosion of third degree of male genital region, sequela |
| T21.77 | Corrosion of third degree of female genital region |
| T21.77XA | Corrosion of third degree of female genital region, initial encounter |
| T21.77XD | Corrosion of third degree of female genital region, subsequent encounter |
| T21.77XS | Corrosion of third degree of female genital region, sequela |
| T21.79 | Corrosion of third degree of other site of trunk |
| T21.79XA | Corrosion of third degree of other site of trunk, initial encounter |
| T21.79XD | Corrosion of third degree of other site of trunk, subsequent encounter |
| T21.79XS | Corrosion of third degree of other site of trunk, sequela |
