# Supplementary material for: Guideline adherence and lost workdays for acute low back pain in the California workers’ compensation system
Source: PLoS One. 2021 Jun 17;16(6):e0253268. doi: 10.1371/journal.pone.0253268 (PMC8211224; doi:10.1371/journal.pone.0253268)
Supplement: S2 Table — Primary LBP codes are medical codes used to define the study population, whereas the unspecific LBP codes were used in the sensitivity analysis to explore the influence of the LBP definition. (DOCX) [file pone.0253268.s002.docx]

**Table S2. Medical codes used to define acute low back pain (LBP) cases. Primary LBP codes are medical codes used to define the study population, whereas the unspecific LBP codes were used in the sensitivity analysis to explore the influence of the LBP definition**

| **Medical Code** | **Medical Code Description** | **Primary LBP Code** | **Unspecific LBP Code** |
| --- | --- | --- | --- |
| 721.3 | Lumbosacral Spondylosis without Myelopathy; Arthritis; Osteoarthritis; Spondylarthritis | Yes | Yes |
| 721.8 | Other Allied Disorders of Spine | No | Yes |
| 721.9 | Spondylosis of Unspecified Site | No | Yes |
| 721.90 | Spondylosis of Unspecified Site without Mention of Myelopathy; Arthritis (Deformans) (Degenerative) (Hypertrophic); Osteoarthritis NOS; Spondylarthrosis NOS | No | Yes |
| 722.3 | Schmorl's Nodes | No | Yes |
| 722.30 | Schmorl's Nodes, Unspecified Region | No | Yes |
| 722.32 | Schmorl's Nodes, Lumbar Region | Yes | Yes |
| 722.39 | Schmorl's Nodes, Other | No | Yes |
| 722.5 | Degeneration of Thoracic or Lumbar Intervertebral Disc | No | Yes |
| 722.51 | Thoracic or Thoracolumbar Disc Degeneration | Yes | Yes |
| 722.52 | Lumbar or Lumbosacral Disc Degeneration | Yes | Yes |
| 722.6 | Degeneration of Intervertebral Disc, Site Unspecified | No | Yes |
| 722.9 | Disc Disorder, Other and Unspecified; Disc Calcification | No | Yes |
| 722.90 | Disc Disorder, Other and Unspecified, Unspecified Region | No | Yes |
| 722.93 | Disc Disorder, Other and Unspecified, Lumbar Region | Yes | Yes |
| 724.2 | Lumbago; Low Back Pain; Low Back Syndrome; Lumbalgia | Yes | Yes |
| 724.5 | Backache, Unspecified; Vertebrogenic (Pain) Syndrome NOS | No | Yes |
| 724.8 | Other Symptoms Referable to Back; Ossification of Posterior Longitudinal Ligament NOS; Panniculitis Specified as Sacral or Affecting Back | No | Yes |
| 846 | Sprains and Strains of Sacroiliac Region | Yes | Yes |
| 846.0 | Sprains and Strains of Sacroiliac Region, Lumbosacral Joint | Yes | Yes |
| 846.1 | Sprains and Strains of Sacroiliac Region, Sacroiliac Ligament | Yes | Yes |
| 846.2 | Sprains and Strains of Sacroiliac Region, Sacrospinatus (Ligament) | Yes | Yes |
| 846.3 | Sprains and Strains of Sacroiliac Region, Sacrotuberous (Ligament) | Yes | Yes |
| 846.8 | Sprains and Strains of Sacroiliac Region, Other Specified Sites | Yes | Yes |
| 846.9 | Sprains and Strains of Sacroiliac Region, Unspecified Site | Yes | Yes |
| 847 | Sprains and Strains of Other and Unspecified Parts of Back | No | Yes |
| 847.2 | Sprains and Strains of Other and Unspecified Parts of Back, Lumbar Spine | Yes | Yes |
| 847.3 | Sprains and Strains of Other and Unspecified Parts of Back, Sacrum; Sacrococcygeal (Ligament) | No | Yes |
| 847.9 | Sprains and Strains of Other and Unspecified Parts of Back, Unspecified Site; Back NOS | No | Yes |
| M46.1 | Sacroiliitis, not elsewhere classified | No | Yes |
| M47.81 | Spondylosis without myelopathy or radiculopathy | No | Yes |
| M47.815 | Spondylosis without myelopathy or radiculopathy, thoracolumbar region | Yes | Yes |
| M47.816 | Spondylosis without myelopathy or radiculopathy, lumbar region | Yes | Yes |
| M47.817 | Spondylosis without myelopathy or radiculopathy, lumbosacral region | Yes | Yes |
| M47.818 | Spondylosis without myelopathy or radiculopathy, sacral and sacrococcygeal region | No | Yes |
| M47.819 | Spondylosis without myelopathy or radiculopathy, site unspecified | No | Yes |
| M47.89 | Other spondylosis | No | Yes |
| M47.895 | Other spondylosis, thoracolumbar region | Yes | Yes |
| M47.896 | Other spondylosis, lumbar region | Yes | Yes |
| M47.897 | Other spondylosis, lumbosacral region | Yes | Yes |
| M47.898 | Other spondylosis, sacral and sacrococcygeal region | No | Yes |
| M47.899 | Other spondylosis, site unspecified | No | Yes |
| M47.9 | Spondylosis, unspecified | No | Yes |
| M51.3 | Other thoracic, thoracolumbar and lumbosacral intervertebral disc degeneration | No | Yes |
| M51.35 | Other intervertebral disc degeneration, thoracolumbar region | Yes | Yes |
| M51.36 | Other intervertebral disc degeneration, lumbar region | Yes | Yes |
| M51.37 | Other intervertebral disc degeneration, lumbosacral region | Yes | Yes |
| M51.4 | Schmorl's nodes | No | Yes |
| M51.45 | Schmorl's nodes, thoracolumbar region | Yes | Yes |
| M51.46 | Schmorl's nodes, lumbar region | Yes | Yes |
| M51.47 | Schmorl's nodes, lumbosacral region | Yes | Yes |
| M51.8 | Other thoracic, thoracolumbar and lumbosacral intervertebral disc disorders | No | Yes |
| M51.85 | Other intervertebral disc disorders, thoracolumbar region | Yes | Yes |
| M51.86 | Other intervertebral disc disorders, lumbar region | Yes | Yes |
| M51.87 | Other intervertebral disc disorders, lumbosacral region | Yes | Yes |
| M51.9 | Unspecified thoracic, thoracolumbar and lumbosacral intervertebral disc disorder | No | Yes |
| M54.5 | Low back pain | Yes | Yes |
| M62.830 | Muscle spasm of back | No | Yes |
| M99.0 | Segmental and somatic dysfunction | No | Yes |
| M99.03 | Segmental and somatic dysfunction of lumbar region | Yes | Yes |
| M99.04 | Segmental and somatic dysfunction of sacral region | No | Yes |
| S33.5 | Sprain of ligaments of lumbar spine | Yes | Yes |
| S33.5XXA | Sprain of ligaments of lumbar spine, initial encounter | Yes | Yes |
| S33.5XXD | Sprain of ligaments of lumbar spine, subsequent encounter | Yes | Yes |
| S33.5XXS | Sprain of ligaments of lumbar spine, sequela | Yes | Yes |
| S33.6 | Sprain of sacroiliac joint | Yes | Yes |
| S33.6XXA | Sprain of sacroiliac joint, initial encounter | Yes | Yes |
| S33.6XXD | Sprain of sacroiliac joint, subsequent encounter | Yes | Yes |
| S33.6XXS | Sprain of sacroiliac joint, sequela | Yes | Yes |
| S33.8 | Sprain of other parts of lumbar spine and pelvis | Yes | Yes |
| S33.8XXA | Sprain of other parts of lumbar spine and pelvis, initial encounter | Yes | Yes |
| S33.8XXD | Sprain of other parts of lumbar spine and pelvis, subsequent encounter | Yes | Yes |
| S33.8XXS | Sprain of other parts of lumbar spine and pelvis, sequela | Yes | Yes |
| S33.9 | Sprain of unspecified parts of lumbar spine and pelvis | Yes | Yes |
| S33.9XXA | Sprain of unspecified parts of lumbar spine and pelvis, initial encounter | Yes | Yes |
| S33.9XXD | Sprain of unspecified parts of lumbar spine and pelvis, subsequent encounter | Yes | Yes |
| S33.9XXS | Sprain of unspecified parts of lumbar spine and pelvis, sequela | Yes | Yes |
| S39.002 | Unspecified injury of muscle, fascia and tendon of lower back | Yes | Yes |
| S39.002A | Unspecified injury of muscle, fascia and tendon of lower back, initial encounter | Yes | Yes |
| S39.002D | Unspecified injury of muscle, fascia and tendon of lower back, subsequent encounter | Yes | Yes |
| S39.002S | Unspecified injury of muscle, fascia and tendon of lower back, sequela | Yes | Yes |
| S39.012 | Strain of muscle, fascia and tendon of lower back | Yes | Yes |
| S39.012A | Strain of muscle, fascia and tendon of lower back, initial encounter | Yes | Yes |
| S39.012D | Strain of muscle, fascia and tendon of lower back, subsequent encounter | Yes | Yes |
| S39.012S | Strain of muscle, fascia and tendon of lower back, sequela | Yes | Yes |
| S39.092 | Other injury of muscle, fascia and tendon of lower back | Yes | Yes |
| S39.092A | Other injury of muscle, fascia and tendon of lower back, initial encounter | Yes | Yes |
| S39.092D | Other injury of muscle, fascia and tendon of lower back, subsequent encounter | Yes | Yes |
| S39.092S | Other injury of muscle, fascia and tendon of lower back, sequela | Yes | Yes |
| S39.82 | Other specified injuries of lower back | No | Yes |
| S39.82XA | Other specified injuries of lower back, initial encounter | No | Yes |
| S39.82XD | Other specified injuries of lower back, subsequent encounter | No | Yes |
| S39.82XS | Other specified injuries of lower back, sequela | No | Yes |
| S39.92 | Unspecified injury of lower back | No | Yes |
| S39.92XA | Unspecified injury of lower back, initial encounter | No | Yes |
| S39.92XD | Unspecified injury of lower back, subsequent encounter | No | Yes |
| S39.92XS | Unspecified injury of lower back, sequela | No | Yes |
