# Supplementary material for: Guideline adherence and lost workdays for acute low back pain in the California workers’ compensation system
Source: PLoS One. 2021 Jun 17;16(6):e0253268. doi: 10.1371/journal.pone.0253268 (PMC8211224; doi:10.1371/journal.pone.0253268)
Supplement: S3 Table — (DOCX) [file pone.0253268.s003.docx]

**Table S3. Full results of the quantile regression model testing the influence of receiving only recommended, only non-recommended, and both recommended and non-recommended treatments on lost workdays.**

| **Variable (field type)** | **Days (95% CI)** | **p-value** |
| --- | --- | --- |
| **Received only recommended interventions**  **(no = 0, yes = 1)** | -11.5 (-13.9, -9.1) | <0.0001 |
| **Received recommended and non-recommended interventions (no = 0, yes = 1)** | -7.9 (-10.3, -5.5) | <0.0001 |
| **Received only no/other interventions (no = 0, yes = 1)** | -7.7 (-10.3, -5.1) | <0.0001 |
| **Medical claims from 4010 billing system (no = 0, yes = 1)** | 4.8 (2.2, 7.3) | <0.0001 |
| **Age (numeric)** | 0.4 (0.3, 0.4) | <0.0001 |
| **Workers’ industry (retail trade as baseline, no = 0, yes = 1)** |  |  |
| Information | 2.0 (-1.7, 5.6) | 0.288 |
| Health care and social assistance | -6.3 (-8.0, -4.6) | <0.0001 |
| Manufacturing | 6.0 (3.0, 9.0) | <0.0001 |
| Wholesale trade | 1.4 (-1.1, 3.9) | 0.264 |
| Administrative and support and waste management and remediation services | 9.2 (6.3, 12.2) | <0.0001 |
| Accommodation and food services | -3.7 (-5.8, -1.6) | 0.001 |
| Transportation and warehousing | 1.5 (-0.5, 3.5) | 0.148 |
| Real Estate and rental and leasing | -1.2 (-5.3, 3.0) | 0.582 |
| Construction | 8.0 (4.2, 11.9) | <0.0001 |
| Educational services | -8.6 (-10.4, -6.9) | <0.0001 |
| Professional, scientific, and technical services | -1.2 (-4.4, 2.0) | 0.466 |
| Other (industries <1%) | 8.4 (-3.3, 20.0) | 0.158 |
| Agriculture, forestry, fishing and hunting | -0.8 (-3.9, 2.4) | 0.642 |
| Other services (except public administration) | -4.5 (-7.9, -1.2) | 0.008 |
| Arts, entertainment, and recreation | -4.3 (-6.4, -2.1) | <0.0001 |
| Public administration | -5.3 (-7.5, -3.1) | <0.0001 |
| Finance and insurance | -0.2 (-2.9, 2.4) | 0.855 |
| **Year of injury (2009 as baseline, no = 0, yes = 1)** |  |  |
| 2010 | 2.9 (1.1, 4.6) | 0.001 |
| 2011 | 7.8 (5.2, 10.3) | <0.0001 |
| 2012 | 9.6 (7.1, 12.1) | <0.0001 |
| 2013 | 16.8 (13.1, 20.5) | <0.0001 |
| 2014 | 13.2 (10.6, 15.8) | <0.0001 |
| 2015 | 6.5 (4.7, 8.3) | <0.0001 |
| 2016 | -0.5 (-3.2, 2.2) | 0.716 |
| 2017 | 5.7 (2.8, 8.6) | <0.0001 |
| 2018 | 3.7 (0.6, 6.9) | 0.019 |
| **Lives in rural location (no = 0, yes = 1)** | -1.2 (-2.3, -0.1) | 0.029 |
| **Workers income (<$25,000 as baseline, no = 0, yes = 1)** | -2.5 (-4.0, -1.0) | 0.001 |
| $25,000 to <$35,000 | -3.2 (-4.9, -1.5) | <0.0001 |
| $35,000 to <$45,000 | -2.1 (-4.3, 0.2) | 0.075 |
| $45,000 to <$55,000 | -0.2 (-3.1, 2.8) | 0.916 |
| $55,000 to <$65,000 | -2.0 (-3.8, -0.3) | 0.022 |
| $65,000 to <$75,000 | -1.2 (-3.0, 0.6) | 0.203 |
| ≥$75,000 |  |  |
| **Number of medical visits in first week of treatment (numeric)** | -0.5 (-0.7, -0.3) | <0.0001 |
| **Number of distinct diagnoses in first week of treatment (numeric)** | 3.4 (2.9, 4.0) | <0.0001 |
| **Male (female as baseline, no = 0, yes = 1)** | -2.6 (-3.7, -1.6) | <0.0001 |
| **Any comorbidities (no = 0, yes = 1)** | -10.7 (-13.6, -7.8) | <0.0001 |
| **Worker has regular employment (no = 0, yes = 1)** | -1.4 (-2.7, -0.2) | 0.028 |
| **Time from injury to first medical visit (numeric)** | 0.1 (0.1, 0.2) | <0.0001 |
| **Any previous workers’ compensation claims (no = 0, yes = 1)** | 3.9 (2.7, 5.0) | <0.0001 |
