# Supplementary material for: Guideline adherence and lost workdays for acute low back pain in the California workers’ compensation system
Source: PLoS One. 2021 Jun 17;16(6):e0253268. doi: 10.1371/journal.pone.0253268 (PMC8211224; doi:10.1371/journal.pone.0253268)
Supplement: S4 Table — (DOCX) [file pone.0253268.s004.docx]

**Table S4. Sensitivity analysis including workers whose primary diagnosis was unspecific to the acute LPP diagnoses used in the main study population (n = 65,296) testing the influence of receiving only recommended, only non-recommended, and both recommended and non-recommended treatments on lost workdays.**

| **Variable (field type)** | **Days (95% CI)** | **p-value** |
| --- | --- | --- |
| **Received only recommended interventions**  **(no = 0, yes = 1)** | -11.7 (-14.0, -9.3) | <0.0001 |
| **Received recommended and non-recommended interventions (no = 0, yes = 1)** | -7.9 (-10.3, -5.5) | <0.0001 |
| **Received only no/other interventions (no = 0, yes = 1)** | -7.8 (-10.3, -5.3) | <0.0001 |
| **Medical claims from 4010 billing system (no = 0, yes = 1)** | 4.3 (2.3, 6.3) | <0.0001 |
| **Age (numeric)** | 0.4 (0.4, 0.5) | <0.0001 |
| **Workers’ industry (retail trade as baseline, no = 0, yes = 1)** |  |  |
| Information | 2.2 (-0.9, 5.4) | 0.168 |
| Health care and social assistance | -6.6 (-8.2, -4.9) | <0.0001 |
| Manufacturing | 6.2 (3.4, 9.0) | <0.0001 |
| Wholesale trade | 1.1 (-1.1, 3.2) | 0.323 |
| Administrative and support and waste management and remediation services | 9.5 (6.6, 12.4) | <0.0001 |
| Accommodation and food services | -3.6 (-5.3, -1.9) | <0.0001 |
| Transportation and warehousing | 1.5 (-0.6, 3.5) | 0.168 |
| Real Estate and rental and leasing | -1.2 (-5.9, 3.6) | 0.63 |
| Construction | 8.7 (5.0, 12.4) | <0.0001 |
| Educational services | -8.5 (-10.0, -7.0) | <0.0001 |
| Professional, scientific, and technical services | -1.7 (-4.9, 1.5) | 0.297 |
| Other (industries <1%) | 7.4 (-3.8, 18.6) | 0.197 |
| Agriculture, forestry, fishing and hunting | -0.1 (-3.0, 2.8) | 0.954 |
| Other services (except public administration) | -4.7 (-7.1, -2.2) | <0.0001 |
| Arts, entertainment, and recreation | -4.5 (-6.6, -2.3) | <0.0001 |
| Public administration | -4.9 (-6.9, -2.8) | <0.0001 |
| Finance and insurance | -1.4 (-4.8, 2.0) | 0.422 |
| **Year of injury (2009 as baseline, no = 0, yes = 1)** |  |  |
| 2010 | 2.9 (1.1, 4.6) | 0.001 |
| 2011 | 6.7 (4.4, 9.1) | <0.0001 |
| 2012 | 9.4 (7.0, 11.8) | <0.0001 |
| 2013 | 16.6 (13.1, 20.0) | <0.0001 |
| 2014 | 12.3 (9.8, 14.7) | <0.0001 |
| 2015 | 5.8 (4.1, 7.6) | <0.0001 |
| 2016 | -1.3 (-3.4, 0.9) | 0.245 |
| 2017 | 4.9 (2.4, 7.5) | <0.0001 |
| 2018 | 2.9 (0.5, 5.3) | 0.017 |
| **Lives in rural location (no = 0, yes = 1)** | -1.5 (-2.7, -0.4) | 0.011 |
| **Workers income (<$25,000 as baseline, no = 0, yes = 1)** | -3.1 (-4.5, -1.7) | <0.0001 |
| $25,000 to <$35,000 | -3.9 (-5.6, -2.2) | <0.0001 |
| $35,000 to <$45,000 | -2.9 (-5.1, -0.7) | 0.01 |
| $45,000 to <$55,000 | 0.5 (-2.3, 3.3) | 0.736 |
| $55,000 to <$65,000 | -2.2 (-3.7, -0.7) | 0.003 |
| $65,000 to <$75,000 | -1.9 (-3.3, -0.5) | 0.009 |
| ≥$75,000 |  |  |
| **Number of medical visits in first week of treatment (numeric)** | -0.4 (-0.6, -0.2) | <0.0001 |
| **Number of distinct diagnoses in first week of treatment (numeric)** | 3.2 (2.7, 3.8) | <0.0001 |
| **Male (female as baseline, no = 0, yes = 1)** | -3.1 (-4.1, -2.1) | <0.0001 |
| **Any comorbidities (no = 0, yes = 1)** | -9.9 (-12.9, -6.9) | <0.0001 |
| **Worker has regular employment (no = 0, yes = 1)** | -1.0 (-2.1, 0.0) | 0.061 |
| **Time from injury to first medical visit (numeric)** | 0.1 (0.1, 0.2) | <0.0001 |
| **Any previous workers’ compensation claims (no = 0, yes = 1)** | 3.8 (2.8, 4.8) | <0.0001 |
