# Supplementary material for: Guideline adherence and lost workdays for acute low back pain in the California workers’ compensation system
Source: PLoS One. 2021 Jun 17;16(6):e0253268. doi: 10.1371/journal.pone.0253268 (PMC8211224; doi:10.1371/journal.pone.0253268)
Supplement: S5 Table — (DOCX) [file pone.0253268.s005.docx]

**Table S5. Sensitivity analysis including propensity score weights within the quantile regression testing the influence of receiving only recommended, only non-recommended, and both recommended and non-recommended treatments on lost workdays.**

| **Variable (field type)** | **Days (95% CI)** | **p-value** |
| --- | --- | --- |
| **Received only recommended interventions**  **(no = 0, yes = 1)** | -11.8 (-14.5, -9.2) | <0.0001 |
| **Received recommended and non-recommended interventions (no = 0, yes = 1)** | -7.7 (-10.3, -5.1) | <0.0001 |
| **Received only no/other interventions (no = 0, yes = 1)** | -7.9 (-10.7, -5.1) | <0.0001 |
| **Medical claims from 4010 billing system (no = 0, yes = 1)** | 6.1 (3.0, 9.3) | <0.0001 |
| **Age (numeric)** | 0.4 (0.3, 0.5) | <0.0001 |
| **Workers’ industry (retail trade as baseline, no = 0, yes = 1)** |  |  |
| Information | 0.8 (-4.0, 5.6) | 0.748 |
| Health care and social assistance | -7.8 (-10.1, -5.6) | <0.0001 |
| Manufacturing | 5.1 (1.4, 8.7) | 0.006 |
| Wholesale trade | -0.3 (-3.2, 2.7) | 0.851 |
| Administrative and support and waste management and remediation services | 8.8 (5.0, 12.5) | <0.0001 |
| Accommodation and food services | -5.4 (-8.0, -2.7) | <0.0001 |
| Transportation and warehousing | 1.4 (-1.2, 4.1) | 0.289 |
| Real Estate and rental and leasing | -0.7 (-5.5, 4.0) | 0.767 |
| Construction | 7.6 (2.8, 12.5) | 0.002 |
| Educational services | -9.0 (-11.2, -6.7) | <0.0001 |
| Professional, scientific, and technical services | -0.7 (-3.6, 2.1) | 0.609 |
| Other (industries <1%) | 7.5 (-5.7, 20.7) | 0.266 |
| Agriculture, forestry, fishing and hunting | -2.3 (-5.4, 0.8) | 0.142 |
| Other services (except public administration) | -5.2 (-8.4, -1.9) | 0.002 |
| Arts, entertainment, and recreation | -5.3 (-8.5, -2.0) | 0.001 |
| Public administration | -6.0 (-8.6, -3.3) | <0.0001 |
| Finance and insurance | -2.2 (-7.6, 3.2) | 0.42 |
| **Year of injury (2009 as baseline, no = 0, yes = 1)** |  |  |
| 2010 | 3.3 (1.0, 5.6) | 0.004 |
| 2011 | 9.1 (5.8, 12.4) | <0.0001 |
| 2012 | 12.1 (9.1, 15.0) | <0.0001 |
| 2013 | 17.6 (13.3, 21.9) | <0.0001 |
| 2014 | 14.0 (10.9, 17.1) | <0.0001 |
| 2015 | 7.8 (5.8, 9.9) | <0.0001 |
| 2016 | 1.6 (-1.6, 4.9) | 0.328 |
| 2017 | 8.0 (4.2, 11.7) | <0.0001 |
| 2018 | 6.5 (2.8, 10.2) | 0.001 |
| **Lives in rural location (no = 0, yes = 1)** | -1.2 (-3.1, 0.7) | 0.227 |
| **Workers income (<$25,000 as baseline, no = 0, yes = 1)** |  |  |
| $25,000 to <$35,000 | -1.5 (-3.0, 0.0) | 0.052 |
| $35,000 to <$45,000 | -1.6 (-3.7, 0.5) | 0.132 |
| $45,000 to <$55,000 | -2.2 (-4.4, -0.1) | 0.043 |
| $55,000 to <$65,000 | -1.9 (-4.7, 0.9) | 0.182 |
| $65,000 to <$75,000 | -0.0 (-3.0, 3.0) | 0.995 |
| ≥$75,000 | -1.2 (-3.1, 0.8) | 0.238 |
| **Number of medical visits in first week of treatment (numeric)** | -0.7 (-1.1, -0.2) | 0.003 |
| **Number of distinct diagnoses in first week of treatment (numeric)** | 3.5 (2.8, 4.2) | <0.0001 |
| **Male (female as baseline, no = 0, yes = 1)** | -3.2 (-4.5, -1.9) | <0.0001 |
| **Any comorbidities (no = 0, yes = 1)** | -9.8 (-13.3, -6.3) | <0.0001 |
| **Worker has regular employment (no = 0, yes = 1)** | -1.7 (-3.1, -0.2) | 0.028 |
| **Time from injury to first medical visit (numeric)** | 0.1 (0.1, 0.2) | <0.0001 |
| **Any previous workers’ compensation claims (no = 0, yes = 1)** | 4.0 (2.7, 5.3) | <0.0001 |
