# Supplementary material for: Guideline adherence and lost workdays for acute low back pain in the California workers’ compensation system
Source: PLoS One. 2021 Jun 17;16(6):e0253268. doi: 10.1371/journal.pone.0253268 (PMC8211224; doi:10.1371/journal.pone.0253268)
Supplement: S6 Table — (DOCX) [file pone.0253268.s006.docx]

**Table S6. ACOEM Guidelines’ acute low back pain recommendation changes over time for common treatments in study ***

|  | **2008** | **2011** | **2015** | **2018** |
| --- | --- | --- | --- | --- |
| NSAID | Rec (A) | Rec (A) | Rec (A) | Rec (A) |
| Muscle relaxant** | Rec (B)*** | Rec (B)*** | Rec (B)*** | Rec (B)*** |
| Manipulation/Manual Therapy | Rec (B/C) § | Rec (B/C) § | Rec (I) | Rec (I) |
| Toradol injection¥ | Rec (A) | Rec (A) | Rec (A) | Rec (A) |
| Opioid £ | Not Rec (C) | Not Rec (C) | Not Rec (A) | Not Rec (A) |
| Carisoprodol | Rec (B)¥ | Rec (B) ¥ | Not Rec (I) | Not Rec (I) |
| Glucocorticosteroids | Not Rec (B) | Not Rec (B) | Not Rec (B) | Not Rec (B) |
| MRI or CT ¤ | Not Rec (I) | Not Rec (I) | Not Rec (I) | Not Rec (I) |
| X-ray ¤ | Not Rec (C) | Not Rec (C) | Not Rec (B) | Not Rec (B) |
| Ultrasound (therapeutic) | No Rec (I) | No Rec (I) | No Rec (I) | No Rec (I) |
| Electrical Stimulation | No Rec (I) | No Rec (I) | Not Rec (I) | Not Rec (I) |
| NSAID | Rec (A) | Rec (A) | Rec (A) | Rec (A) |
| Specific exercises of directional stretching and progressive aerobic exercise are first-line treatments, but are unable to be assessed in this study as that level of detail is not available in the dataset from California.  **Not including Carisoprodol, which is not recommended as of 2015.  ***Unless mild to moderate then not recommended.  §Categories B/C are based on meeting vs. not meeting the Clinical Prediction Rule, which was subsequently not confirmed with subsequent research.  ¥Not specifically addressed. Recommendation inferred from category of medication.  £Routine use not recommended. Recommended for severe back pain with more restrictive indications over this timeframe.  ¤ Absent red flags, including trauma. | | | | |
