# Supplementary material for: Guideline adherence and lost workdays for acute low back pain in the California workers’ compensation system
Source: PLoS One. 2021 Jun 17;16(6):e0253268. doi: 10.1371/journal.pone.0253268 (PMC8211224; doi:10.1371/journal.pone.0253268)
Supplement: S7 Table — (DOCX) [file pone.0253268.s007.docx]

**Table S7. Comparisons between select recommendation statements of the ACOEM, American College of Physicians (ACP) and United Kingdoms’ National Institute of Health and Care Excellence (NICE) guidelines for acute low back pain.^1^**

|  | **ACOEM (2018)** | **ACP (2017)^2^** | **NICE/UK (2020)**^3^ |
| --- | --- | --- | --- |
| NSAID | Rec. (A) | Rec (Mod) | Rec |
| Muscle relaxant** | Rec (B)**** | Rec (Mod) | Silent |
| Manipulation | Rec (I)# | Rec (Low) | Rec# |
| Toradol injection | Rec (A) | Silent | Silent |
| Manual Therapy | Rec (I)# | Rec (Low) | Rec# |
| Acupuncture | Not Rec (I) | Rec. (Low) | Not Rec |
| Opioid | Not Rec (A) | Silent & | Not Rec |
| Anti-depressants (TCA, SNRI) | Rec (C)$ | Silent | Not Rec |
| Anti-depressant (SSRI) | Not Rec(A) | Silent | Not Rec |
| Massage | Rec (I) | Rec (Low) | Rec# |
| Heat | Rec (C) | Rec (Mod) | Silent |
| Carisoprodol | Not Rec (I) | Rec mm relaxants/Silent | Silent |
| Glucocorticosteroids | Not Rec (B) | Silent | Silent |
| MRI or CT | Not Rec (C) | Silent | Not Rec. |
| X-ray*** | Not Rec (B) | Silent | Not Rec |
| Ultrasound (therapeutic) | No Rec (I) | Silent | Not Rec |
| Electrical Stimulation | Not Rec (I) | Silent | Not Rec |
| ^1^ These are summaries of the guidelines’ recommendations statements. Readers are advised that there also may be additional brief discussion of topics in the guidelines text without mention in guidelines’ recommendations statements. For purposes of this table, a recommendation for manipulation was entered as the same for manual therapy.  ^2^ Qaseem et al. (2017)  ^3^ NICE low back guideline (2016)  ** Not including Carisoprodol, which is not recommended.  *** Absent red flags, including trauma.  **** Unless mild to moderate then not recommended.  # Only if part of a rehabilitation program that includes active exercise.  & No formal recommendation, but mentioned as potentially harmful.  $ Not including SSRI antidepressants, which are not recommended. | | | |
